# SelCysML: a hybrid ML-BLAST pipeline for the automated identification and functional annotation of bacterial selenoproteins

**DOI:** 10.3389/fbinf.2026.2001675

**Published:** 2026-10-26

**Authors:** José L. Lavín, José A. Oguiza

**Affiliations:** 1 Applied Mathematics Department, NEIKER – Basque Institute for Agricultural Research and Development, Basque Research and Technology Alliance (BRTA), Derio, Bizkaia, Spain; 2 Genetics, Genomics and Microbiology Research Group, Institute for Multidisciplinary Research in Applied Biology (IMAB), Universidad Pública de Navarra (UPNA), Pamplona, Spain

**Keywords:** annotation, bacteria, machine learning, proteome, selenoproteins

## Abstract

Selenoproteins are characterized by the incorporation of selenocysteine (Sec) via UGA codon recoding, play vital roles in redox homeostasis and enzymatic catalysis. Despite this biological significance, their computational identification remains challenging, and recent assessments confirm that public databases suffer from widespread annotation errors, often misclassifying these proteins. Current predictive tools frequently operate as “black boxes” with limited interpretability or lack integrated functional validation layers, which contributes to high false-positive rates and persistent ambiguities in the annotation of the selenoproteome. We present SelCysML, an accessible and highly accurate computational pipeline that integrates an ensemble of machine learning (ML) models with a parallel functional validation module using BLAST. By utilizing curated physicochemical descriptors—Amino Acid Composition (AAC) and Composition, Transition, Distribution (CTDC)—the model achieves a robust performance, with an AUC-ROC of 0.968 and a Matthews Correlation Coefficient (MCC) of 0.787. SelCysML not only classifies protein sequences but also provides functional evidence through sequence similarity, effectively bridging the gap between automated prediction and experimental biological insight. SelCysML provides a user-friendly, interpretable, and precise solution for the identification of bacterial selenoproteins in proteomic datasets, serving as a reliable tool for both bioinformaticians and experimental biologists.

## 1 Introduction

The identification of selenoproteins—proteins that contain selenocysteine (Sec) as the 21st amino acid—is a vital task in functional genomics (Hatfield and Gladyshev, 2002). Because selenocysteine is encoded by the UGA codon, which typically acts as a stop signal, the computational prediction of these proteins is notoriously difficult (Kryukov et al., 2003). Recent bioinformatic assessments have highlighted that despite the growing availability of genomic resources, standard pipelines frequently misidentify selenoproteins as truncated or standard proteins, leading to widespread flaws in public database annotations (Ticó et al., 2026). As discussed by Santesmasses et al. (2020), the progress in computational tools over the last two decades has been essential to translate genomic data into insights regarding selenium biology, yet the regulation and identification of Sec-insertion mechanisms remain critical challenges.

Recent advances in deep learning have led to the development of several predictors, such as deep-Sep (Xiao and Zhang, 2025). Although these tools demonstrate high sensitivity, they typically operate under a non-transparent “black-box” paradigm and rely solely on *de novo* probabilistic classification. Lacking mechanisms to verify the biological plausibility of their outputs, existing approaches are prone to high false-positive rates, particularly when sequences share physicochemical features with non-selenoprotein redox families. To address these limitations, we developed SelCysML, an interpretable, three-layer hybrid pipeline designed for high-precision selenoprotein identification and functional annotation (Figure 1). By combining an ensemble of machine learning models trained on transparent physicochemical descriptors (AAC and CTDC) with an automated dual-BLAST validation module integrating BSepDB (Hong et al., 2026) and UniProt (UniProt Consortium, 2023), SelCysML bridges the gap between raw statistical predictions and verifiable biological evidence, providing a robust framework for selenoproteome exploration.

**FIGURE 1:**
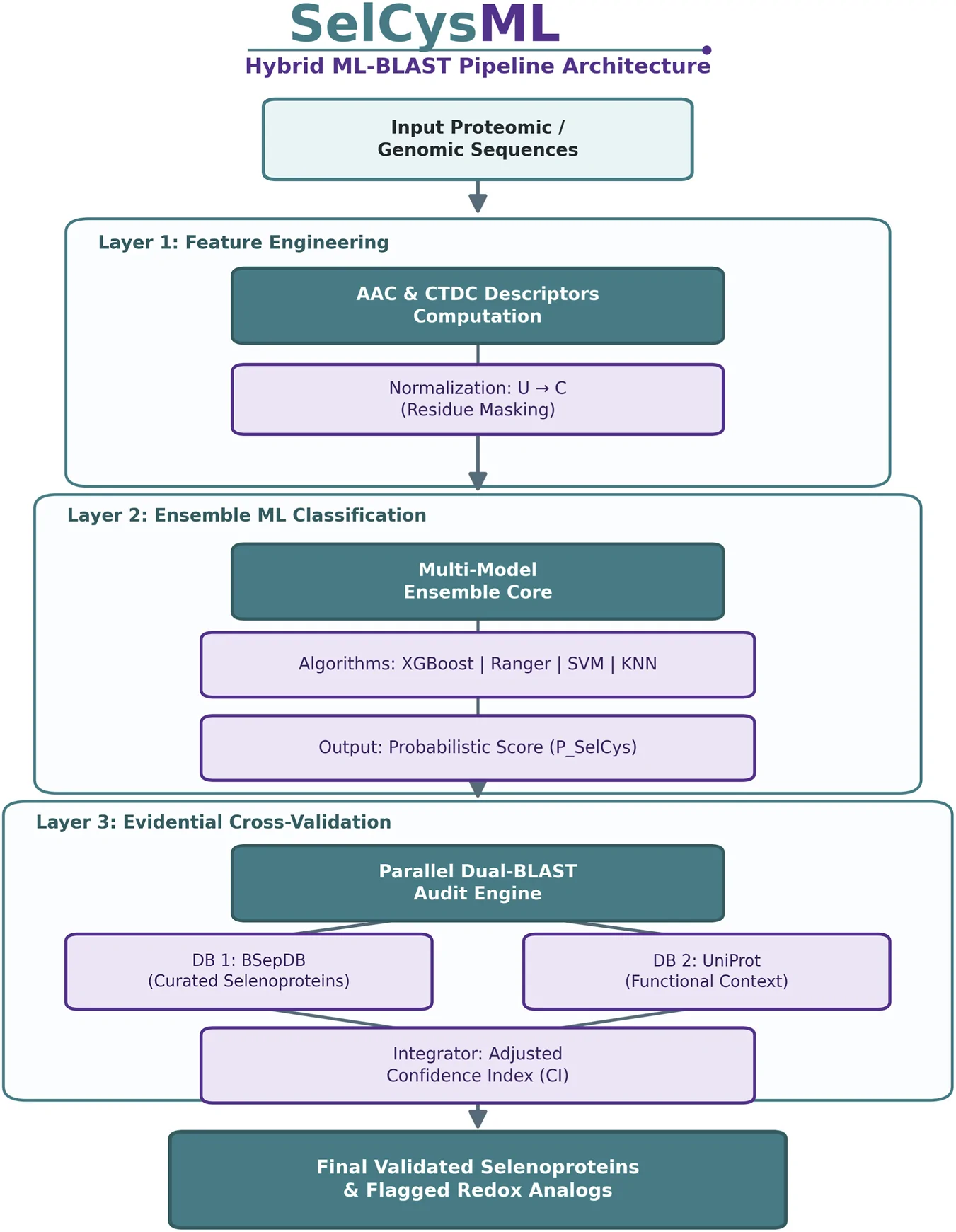
Schematic workflow of the SelCysML three-layer architecture. The pipeline processes protein or genomic input sequences through three sequential, integrated layers: (1) Feature extraction and physicochemical characterization (AAC and CTDC descriptors); (2) Multi-model Ensemble Machine Learning classification providing probabilistic scores; and (3) Evidential cross-validation via parallel BLAST searches against specialized databases (BSepDB and UniProt), culminating in an integrated Confidence Index ($CI$) and functional annotation.

## 2 Materials and methods

### 2.1 Dataset preparation and quality control

To evaluate the performance of SelCysML, we compiled a balanced, non-redundant dataset of 30,000 protein sequences, comprising 15,000 selenoproteins and 15,000 control proteins. The Generalistic model’s control dataset was composed by proteins from different phylogenetic kingdoms while the Bacterial model`s control dataset only had bacterial sequences in it.

The selenoprotein dataset was curated from BSepDB (Hong et al., 2026), a comprehensive repository encompassing over 57,000 selenoprotein entries across 111 families and 16,621 distinct bacterial organisms, ensuring a broad taxonomic representation across 88 distinct clades. To construct robust control sets, we queried the UniProt database using two different strategies, first, a multi-domain filtering strategy. We retrieved sequences covering all three domains of life (taxonomy IDs: 2, 2,157, 2,759) while strictly excluding any entries containing the keyword “Selenocysteine” (NOT keyword: “Selenocysteine”), ensuring no overlap existed between the positive and negative classes. Since the positive class is strictly bacterial, our negative set was intentionally diversified across all domains of life to ensure a robust representation of standard proteins. Consequently, while our reported metrics reflect high discriminative performance, they primarily represent the model’s ability to distinguish bacterial selenoproteins against a broad-domain background. Complementary to the previous approach we also generated a bacterial exclusive dataset to offer users the option to test their data against a specific bacterial model. To that aim, the new control was obtained by querying UniProt via a restricted phylogenetic domain for bacteria (taxonomy ID: 2), and again, excluding any entries containing the keyword “Selenocysteine” (NOT keyword: “Selenocysteine”).

To ensure rigorous evaluation and prevent data leakage, the entire dataset was subjected to a global clustering process using CD-HIT (Fu et al., 2012) with a sequence identity cut-off of 0.70 prior to splitting into training and test sets. This ensures that no sequences in the test partition share high homology (>70% identity) with those used during training. Following CD-HIT filtering, the final datasets were standardized through a cleaning pipeline where non-standard characters were removed. Selenocysteine residues (U) were normalized to cysteine (C) to ensure full compatibility with the protr feature extraction package. While explicit encoding of Selenocysteine (Sec) would theoretically provide the highest biochemical fidelity, this normalization approach mimics the real-world scenario of automated genomic annotation, where UGA codons are often prematurely annotated as stop signals. Only sequences with a minimum length of 40 amino acids were retained, ensuring the biological relevance and structural stability of the training data. To prevent artifacts arising from prematurely truncated proteins or misannotated selenoproteins, negative controls were stringently filtered against high-confidence reference proteomes (UniProt/RefSeq), excluding any entries with ambiguous stop codons or anomalous fragment lengths.

### 2.2 Feature engineering

The predictive power of SelCysML relies on two core protein descriptors: Amino Acid Composition (AAC) and Composition, Transition, Distribution (CTDC) (Figure 1). These descriptors were calculated using the *protr* package in R (Xiao et al., 2015), ensuring a standardized transformation of sequences into numerical vectors suitable for machine learning.

Amino Acid Composition (AAC): A 20-dimensional vector representing the frequency of each standard amino acid. Composition, Transition, Distribution (CTDC): Providing a granular description of the distribution of physicochemical properties (hydrophobicity, charge, and polarity) along the sequence. Numerical features were normalized using a median-imputation and scaling strategy to handle potential missing values and variance across the dataset. While nucleotide context (such as UGA recoding signals and downstream SECIS elements) is biologically definitive, SelCysML is purposely engineered to operate at the protein sequence level. This design enables high-throughput annotation and metagenomic screening where genomic assemblies or UTR regions are frequently fragmented or missing. The loss of direct nucleotide context is actively compensated for by Layer 3 (BLAST cross-validation).

### 2.3 Machine learning ensemble model

We employed an ensemble approach utilizing the *tidymodels* framework (Kuhn and Wickham, 2020). The final model aggregates predictions from multiple algorithms, including XGBoost (Chen et al., 2024), Ranger (Random Forest) (Wright and Ziegler, 2017), Kernlab (Support Vector Machines) (Karatzoglou et al., 2004), and KKNN (K-Nearest Neighbors) (Schliep and Hechenbichler, 2016). To mitigate class imbalance, a step_downsample procedure was applied within the recipe. The final ensemble model aggregated the probability outputs from the trained algorithms using a mean-averaging strategy. Performance was validated on an independent test set (30% split), with metrics including Balanced Accuracy, MCC, and AUC-ROC. Confidence intervals for performance metrics were computed using a bootstrap approach (50 iterations) to ensure statistical robustness. The models were trained to predict a probability score for the “Selenoprotein” class, with a classification threshold optimized to maximize both sensitivity and specificity.

To enhance the tool’s versatility and mitigate potential taxonomic biases, we implemented a dual-model selection framework within SelCysML. Users can select between a bacteria-specific model (trained on high-quality bacterial selenoproteomes) and a multi-kingdom model. This flexibility allows researchers to tailor the predictive approach to the specific taxonomic context of their proteomic datasets, ensuring maximum accuracy for domain-specific studies.

### 2.4 The three-layer predictive architecture

The SelCys-Predictor architecture is organized into three distinct operational layers that transform raw sequences into functionally annotated data: (i) Layer 1 (Feature Engineering), extraction of physicochemical descriptors (AAC and CTDC) to characterize the protein sequences; (ii) Layer 2 (Ensemble ML Classification), a multi-model ensemble (XGBoost, Ranger, etc.) that generates a probability-based classification (Selenoprotein vs. Standard); and (iii) Layer 3 (Evidential Cross-Validation), a parallel validation engine that utilizes a dual-BLAST strategy (Camacho et al., 2009) to compare ML predictions against local, standalone replicas of specialized databases, including BSepDB (Hong et al., 2026) and UniProt (UniProt Consortium, 2023). These databases are distributed as integrated dependencies with the SelCysML pipeline, ensuring fully offline execution and reproducible functional validation.

This hybrid ML-BLAST approach significantly enhances the reliability of the system. By integrating functional validation, this third layer assigns an “Evidential Status” to each prediction—categorizing them as “Validated,” “Potential False Positive,” or “Potential False Negative”—and calculates an “Adjusted Confidence” score ($CI$).

The final confidence of each prediction is calculated through a two-stage process. First, we define the Base Confidence ($C_b$) derived from the ML model, where:

$$C\_b=\backslash \max\left(P\_\left\{SelCys\right\},1-P\_\left\{SelCys\right\}\right)\;\backslash times\;100.$$

Subsequently, the integrated Confidence Index (CI) is computed using the following piecewise function:

$$\text{CI}=\left\{\begin{array}{cc} 99.9, & \text{if validated }\left(\text{BLAST}+\right)\\ Cb\;x\;\alpha , & \text{if not validated }\left(\text{ML only}\right)\\ Cb, & \text{if standard }\left(\text{Concordant}\right) \end{array}\right.$$

Where α represents a penalty factor (α = 0.3) applied to ML-positive predictions that lack homology in annotated databases. This mechanism effectively identifies false positives—such as proteins containing redox-active cysteine-rich motifs—, which are automatically flagged for expert manual inspection within the SelCysML interface. Consequently, this architecture ensures that every prediction is not only statistically robust but also backed by biological homology evidence, reducing manual curation efforts and clarifying the annotation of the selenoproteome.

## 3 Results

### 3.1 Performance evaluation

To assess the predictive capability of SelCysML, we evaluated both the general ensemble model and the bacteria-specific classifier on independent test sets on balanced sets of 2000 proteins in both cases (divided into 1,000 selenoproteins and 1,000 controls). The general model demonstrated high discriminative power with an AUC-ROC of 0.968 and a Mathews Correlation Coefficient (MCC) (Matthews, 1975) of 0.787. In contrast, the specialized bacterial model exhibited superior performance within prokaryotic environments, achieving an AUC-ROC of 0.977 and an MCC of 0.843, validating the strategy of utilizing domain-adjusted architectures for high-precision genomic screening.

Detailed classification metrics for the general model are summarized in Table 1. The General demonstrated excellent sensitivity, identifying 97.3% of the selenoprotein set—a critical feature for genomic screening to ensure that potential candidates are not overlooked. With a specificity of 80.3% and a balanced accuracy of 88.8%, these results confirm the model’s robustness in discriminating between selenoproteins and standard sequences.

**TABLE 1:** Summary of classification performance metrics for the SelCysML ensemble model.

Metric	General model	Bacterial model
Sensitivity	0.973	0.965
Specificity	0.803	0.875
Balanced accuracy	0.888	0.920
MCC	0.787	0.843
AUC-ROC	0.968	0.977

Performance metrics were computed on independent test sets (30% split, balanced sets of 2,000 proteins) using standard evaluation formulas: Sensitivity = TP/(TP + FN); specificity = TN/(TN + FP); balanced accuracy = (sensitivity + specificity)/2; Matthews Correlation Coefficient (MCC) = (TP × TN - FP × FN)/sqrt((TP + FP)(TP + FN)(TN + FP)(TN + FN)); and AUC-ROC representing the area under the receiver operating characteristic curve. The classification threshold was optimized to maximize both sensitivity and specificity.

### 3.2 Functional evidence and annotation

The most critical innovation of SelCys-Predictor is the integration of a third operational layer: Evidential Cross-Validation. Unlike conventional methods that terminate at the classification stage (Layer 2), our pipeline employs a parallel validation engine that utilizes a dual-BLAST strategy to contrast the ML output against specialized databases, such as BSepDB (Hong et al., 2026) and UniProt (UniProt Consortium, 2023). This third layer serves as an “automated auditor” of the ML predictions. It does not merely confirm identity; it operates through an evidential logic that categorizes outcomes into “Validated,” “Potential False Positive,” or “Potential False Negative.” By identifying biological homology to non-selenoprotein enzymes—such as the chorismate synthase example detected in our test set—this layer acts as a high-precision filter for proteins containing complex redox-active motifs that frequently deceive standard classifiers. Consequently, this architecture ensures that every prediction is not only statistically supported by the ML ensemble but is also biologically verified, transitioning the tool from a ‘black box’ classifier to a reliable platform for functional selenoprotein annotation.”

## 4 Discussion

The performance of SelCysML confirms that an interpretable ensemble approach can match or even exceed the predictive power of more complex, deep-learning-only models. With an AUC-ROC of 0.968 and an MCC of 0.787, our tool provides high discriminative capability while maintaining a clear, transparent link between protein features (AAC/CTDC) and prediction outcomes.

A key advantage of SelCysML over state-of-the-art tools like deep-Sep (Xiao and Zhang, 2025) is its three-layer predictive architecture, which culminates in an evidential cross-validation module. While deep learning models excel at identifying abstract patterns, they often lack the functional transparency required by experimental biologists. Our integrated BLAST module bridges this gap, transforming a simple binary prediction into a verifiable functional annotation. Furthermore, while recent efforts in genomic selenoprotein annotation—such as those by Ticó et al. (2026) —have significantly advanced our ability to identify selenoprotein genes through homology-based pipelines, SelCysML provides a complementary framework. By focusing on physicochemical descriptors rather than strictly on sequence similarity, our tool is uniquely positioned to identify novel or highly divergent selenoproteins that may escape conventional alignment-based detection.

Regarding our computational implementation, we adopted a U-masked regime (U→C) to normalize sequences for feature extraction. While explicit encoding of Selenocysteine (Sec) would theoretically provide higher biochemical fidelity, this normalization approach mimics the real-world scenario of automated genomic annotation, where UGA codons are often prematurely identified as stop signals. By demonstrating robust performance under this constraint, SelCysML proves that the protein sequence context flanking the Sec-residue contains sufficient structural and physicochemical information to infer selenoprotein identity, even when the explicit residue identity is obscured by standard annotation artifacts. Future iterations of SelCysML will incorporate U-aware encodings (21-AA AAC) to further quantify the specific contribution of explicit Sec-residues versus context-based sequence features.

A potential consideration in sequence-based machine learning classifiers is the risk of capturing broad phylogenetic signals rather than true functional motifs. Because our initial dataset incorporated multi-domain negative controls against an exclusively bacterial positive class, global compositional descriptors (AAC and CTDC) could theoretically reflect general proteomic differences across domains of life rather than intrinsic selenoprotein properties. To rigorously address this taxonomic confounder, we developed a bacteria-specific predictive engine utilizing domain-matched negative controls, successfully isolating local physicochemical profiles—such as thiol- and selenol-associated amino acid distributions—from broader phylogenetic backgrounds. This specialized model demonstrated superior performance in prokaryotic environments, achieving an AUC-ROC of 0.977 and an MCC of 0.843. Consequently, SelCysML implements a dual-model selection framework that allows users to choose between the bacteria-specific and general-purpose models, effectively decoupling selenoprotein-specific features from broader domain-of-life compositional trends and aligning with previous findings on prokaryotic selenoproteome constraints.

Finally, with reference to the observed 80.3% specificity, our analysis indicates that most false-positive predictions occur in proteins sharing structural motifs with selenoproteins, such as redox-active cysteine-rich domains (Supplementary Table S1). Crucially, our third-layer Evidential Cross-Validation module acts as an automated safeguard in these instances; rather than allowing these sequences to be misclassified as selenoproteins, the pipeline identifies the lack of homology to annotated selenoproteomes and flags them for expert manual review.

To evaluate the biological relevance of these flagged predictions, we performed a functional enrichment analysis using STRING-db (v12.0) (Szklarczyk et al., 2023). The results (Supplementary Table S2) revealed a marked overrepresentation of proteins involved in RNA processing, methyltransferase activity, and redox-related enzymatic functions (see Figure 2 for protein-protein interaction network). This enrichment is consistent with the evolutionary and structural analysis of selenoprotein catalytic centers described by Kryukov et al. (2003), who noted that the similarity in physicochemical properties between cysteine and selenocysteine residues—specifically within thiol-dependent motifs—often leads to bioinformatic classification challenges. Consequently, our model’s tendency to flag these sequences, supported by the evidential filtering of the third layer, highlights SelCysML’s high sensitivity for identifying structurally analogous catalytic centers of significant biological interest, rather than representing stochastic classification errors.

**FIGURE 2:**
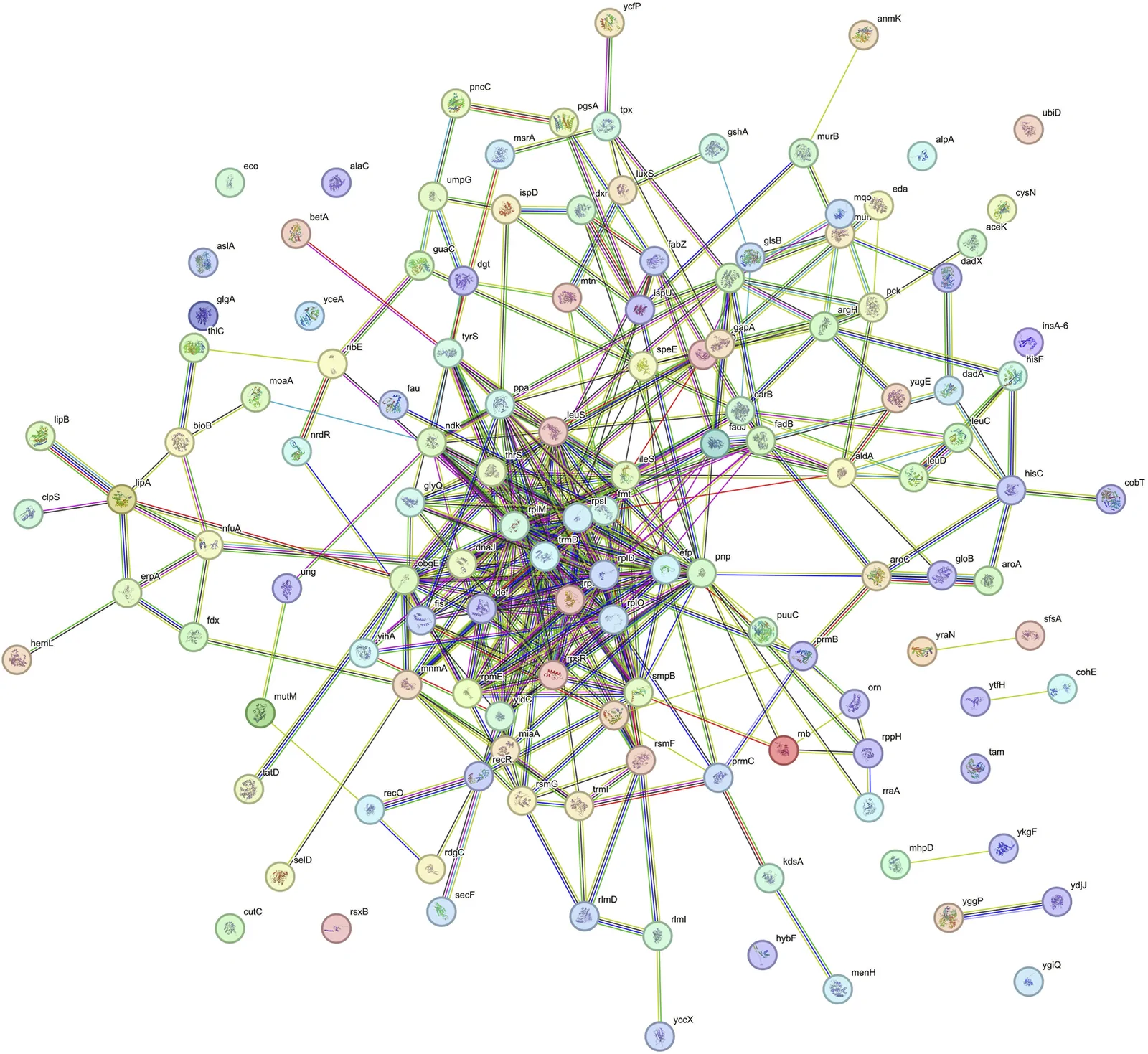
Functional interactome of SelCysML false-positive predictions. Protein-protein interaction (PPI) network generated using STRING-db (v12.0) for the sequences identified as ‘Potential False Positives’. Nodes represent the flagged proteins, and edges indicate functional associations. The network reveals a significant enrichment in proteins associated with RNA processing, methyltransferase activity, and redox-related enzymatic functions. This connectivity reflects the shared physicochemical motifs between cysteine-rich domains and selenocysteine catalytic centers, illustrating that these flagged sequences represent biologically relevant, structurally analogous proteins rather than stochastic classification artifacts.

Furthermore, the identification of structural false positives—such as cysteine-rich redox enzymes and chorismate synthase homologs—reflects an inherent biological challenge in selenoprotein prediction rather than a random statistical failure. Due to the close evolutionary and physicochemical relationship between cysteine and selenocysteine residues, proteins participating in sulfur-dependent catalysis frequently share conserved structural scaffolds and active-site environments with *bona fide* selenoproteins. Because standard probabilistic machine learning models alone cannot fully resolve this topological convergence, the implementation of our third-layer Evidential Cross-Validation module is essential. By coupling ML classification with parallel BLAST searches against specialized repositories like BSepDB (Hong et al., 2026), the pipeline actively audits these ambiguous matches, filtering out structural analogues and transforming raw probabilistic outputs into reliable, biologically verified functional annotations.

## 5 Conclusion

SelCysML provides a robust, interpretable, and reproducible R/Shiny framework for the high-precision identification and functional annotation of bacterial selenoproteins. By integrating an ensemble-based machine learning pipeline with a rigorous three-layer architecture—culminating in an evidential cross-validation module—we offer a solution that bridges the gap between statistical probability and biological insight.

By addressing the reproducibility and transparency issues often associated with ‘black-box’ deep-learning models, SelCysML represents a significant step forward in the automated exploration of the selenoproteome. The tool’s ability to flag structural analogs for expert inspection, rather than providing opaque classifications, transforms the computational screening process into a reliable platform for functional discovery. Given its user-friendly interface, local standalone validation database, and transparent evidential architecture, we anticipate that SelCysML will facilitate broad adoption and accelerate the discovery of novel selenoproteins within the scientific community.

## Data Availability

The SelCysML source code and user manuals are publicly available on GitHub (https://github.com/jllavin77/SelCys). The ensemble bundle model and the BLASTdb required for the homology based optional validation, are publicly available on Zenodo (https://zenodo.org/uploads/20625234). The code and datasets used to generate the independent testing are available upon request along with the curated non-redundant training dataset (clustered at 70% identity to match the reported performance metrics).
