# Supplementary material for: SelCysML: a hybrid ML-BLAST pipeline for the automated identification and functional annotation of bacterial selenoproteins
Source: Front Bioinform. 2026 Oct 26;6:2001675. doi: 10.3389/fbinf.2026.2001675 (PMC13745706; doi:10.3389/fbinf.2026.2001675)
Supplement: Supplementary file 1 [file Table2.docx]

```
#node	identifier		category	term ID	term description
aceK	511145.b4016	GO Process	GO:0005975	Carbohydrate metabolic process
aceK	511145.b4016	GO Process	GO:0005996	Monosaccharide metabolic process
aceK	511145.b4016	GO Process	GO:0006006	Glucose metabolic process
aceK	511145.b4016	GO Process	GO:0006081	Cellular aldehyde metabolic process
aceK	511145.b4016	GO Process	GO:0006082	Organic acid metabolic process
aceK	511145.b4016	GO Process	GO:0006091	Generation of precursor metabolites and energy
aceK	511145.b4016	GO Process	GO:0006097	Glyoxylate cycle
aceK	511145.b4016	GO Process	GO:0006099	Tricarboxylic acid cycle
aceK	511145.b4016	GO Process	GO:0006468	Protein phosphorylation
aceK	511145.b4016	GO Process	GO:0006470	Protein dephosphorylation
aceK	511145.b4016	GO Process	GO:0006793	Phosphorus metabolic process
aceK	511145.b4016	GO Process	GO:0006796	Phosphate-containing compound metabolic process
aceK	511145.b4016	GO Process	GO:0006807	Nitrogen compound metabolic process
aceK	511145.b4016	GO Process	GO:0008152	Metabolic process
aceK	511145.b4016	GO Process	GO:0009060	Aerobic respiration
aceK	511145.b4016	GO Process	GO:0009987	Cellular process
aceK	511145.b4016	GO Process	GO:0015980	Energy derivation by oxidation of organic compounds
aceK	511145.b4016	GO Process	GO:0016310	Phosphorylation
aceK	511145.b4016	GO Process	GO:0016311	Dephosphorylation
aceK	511145.b4016	GO Process	GO:0018105	Peptidyl-serine phosphorylation
aceK	511145.b4016	GO Process	GO:0018193	Peptidyl-amino acid modification
aceK	511145.b4016	GO Process	GO:0018209	Peptidyl-serine modification
aceK	511145.b4016	GO Process	GO:0019318	Hexose metabolic process
aceK	511145.b4016	GO Process	GO:0019538	Protein metabolic process
aceK	511145.b4016	GO Process	GO:0019752	Carboxylic acid metabolic process
aceK	511145.b4016	GO Process	GO:0032787	Monocarboxylic acid metabolic process
aceK	511145.b4016	GO Process	GO:0036211	Protein modification process
aceK	511145.b4016	GO Process	GO:0043170	Macromolecule metabolic process
aceK	511145.b4016	GO Process	GO:0043412	Macromolecule modification
aceK	511145.b4016	GO Process	GO:0043436	Oxoacid metabolic process
aceK	511145.b4016	GO Process	GO:0044237	Cellular metabolic process
aceK	511145.b4016	GO Process	GO:0044238	Primary metabolic process
aceK	511145.b4016	GO Process	GO:0044262	Cellular carbohydrate metabolic process
aceK	511145.b4016	GO Process	GO:0044281	Small molecule metabolic process
aceK	511145.b4016	GO Process	GO:0045333	Cellular respiration
aceK	511145.b4016	GO Process	GO:0046487	Glyoxylate metabolic process
aceK	511145.b4016	GO Process	GO:0050790	Regulation of catalytic activity
aceK	511145.b4016	GO Process	GO:0065007	Biological regulation
aceK	511145.b4016	GO Process	GO:0065009	Regulation of molecular function
aceK	511145.b4016	GO Process	GO:0070262	Peptidyl-serine dephosphorylation
aceK	511145.b4016	GO Process	GO:0071704	Organic substance metabolic process
aceK	511145.b4016	GO Process	GO:1901564	Organonitrogen compound metabolic process
aceK	511145.b4016	GO Function	GO:0000166	Nucleotide binding
aceK	511145.b4016	GO Function	GO:0003824	Catalytic activity
aceK	511145.b4016	GO Function	GO:0004672	Protein kinase activity
aceK	511145.b4016	GO Function	GO:0004674	Protein serine/threonine kinase activity
aceK	511145.b4016	GO Function	GO:0004721	Phosphoprotein phosphatase activity
aceK	511145.b4016	GO Function	GO:0004722	Protein serine/threonine phosphatase activity
aceK	511145.b4016	GO Function	GO:0005488	Binding
aceK	511145.b4016	GO Function	GO:0005524	ATP binding
aceK	511145.b4016	GO Function	GO:0008772	[Isocitrate dehydrogenase (NADP+)] kinase activity
aceK	511145.b4016	GO Function	GO:0016208	AMP binding
aceK	511145.b4016	GO Function	GO:0016301	Kinase activity
aceK	511145.b4016	GO Function	GO:0016740	Transferase activity
aceK	511145.b4016	GO Function	GO:0016772	Transferase activity, transferring phosphorus-containing groups
aceK	511145.b4016	GO Function	GO:0016773	Phosphotransferase activity, alcohol group as acceptor
aceK	511145.b4016	GO Function	GO:0016787	Hydrolase activity
aceK	511145.b4016	GO Function	GO:0016788	Hydrolase activity, acting on ester bonds
aceK	511145.b4016	GO Function	GO:0016791	Phosphatase activity
aceK	511145.b4016	GO Function	GO:0017076	Purine nucleotide binding
aceK	511145.b4016	GO Function	GO:0030554	Adenyl nucleotide binding
aceK	511145.b4016	GO Function	GO:0032553	Ribonucleotide binding
aceK	511145.b4016	GO Function	GO:0032555	Purine ribonucleotide binding
aceK	511145.b4016	GO Function	GO:0032559	Adenyl ribonucleotide binding
aceK	511145.b4016	GO Function	GO:0035639	Purine ribonucleoside triphosphate binding
aceK	511145.b4016	GO Function	GO:0036094	Small molecule binding
aceK	511145.b4016	GO Function	GO:0042578	Phosphoric ester hydrolase activity
aceK	511145.b4016	GO Function	GO:0043167	Ion binding
aceK	511145.b4016	GO Function	GO:0043168	Anion binding
aceK	511145.b4016	GO Function	GO:0043169	Cation binding
aceK	511145.b4016	GO Function	GO:0097159	Organic cyclic compound binding
aceK	511145.b4016	GO Function	GO:0097367	Carbohydrate derivative binding
aceK	511145.b4016	GO Function	GO:0140096	Catalytic activity, acting on a protein
aceK	511145.b4016	GO Function	GO:1901265	Nucleoside phosphate binding
aceK	511145.b4016	GO Function	GO:1901363	Heterocyclic compound binding
aceK	511145.b4016	GO Component	GO:0005622	Intracellular anatomical structure
aceK	511145.b4016	GO Component	GO:0005737	Cytoplasm
aceK	511145.b4016	GO Component	GO:0110165	Cellular anatomical entity
aceK	511145.b4016	STRING clusters	CL:2010	Mixed, incl. Carbon metabolism, and Fatty acid metabolic process
aceK	511145.b4016	STRING clusters	CL:2011	Carbon metabolism, and Oxidative phosphorylation
aceK	511145.b4016	STRING clusters	CL:2012	Carbon metabolism, and Pyruvate metabolism
aceK	511145.b4016	STRING clusters	CL:2013	Carbon metabolism, and Formate C-acetyltransferase activity
aceK	511145.b4016	STRING clusters	CL:2014	Citrate cycle (TCA cycle), and Pyruvate metabolism
aceK	511145.b4016	STRING clusters	CL:2016	Pyruvate metabolism, and Glyoxylate bypass
aceK	511145.b4016	COMPARTMENTS	GOCC:0005622	Intracellular
aceK	511145.b4016	COMPARTMENTS	GOCC:0005737	Cytoplasm
aceK	511145.b4016	COMPARTMENTS	GOCC:0110165	Cellular anatomical entity
aceK	511145.b4016	UniProt Keywords	KW-0067	ATP-binding
aceK	511145.b4016	UniProt Keywords	KW-0329	Glyoxylate bypass
aceK	511145.b4016	UniProt Keywords	KW-0378	Hydrolase
aceK	511145.b4016	UniProt Keywords	KW-0418	Kinase
aceK	511145.b4016	UniProt Keywords	KW-0547	Nucleotide-binding
aceK	511145.b4016	UniProt Keywords	KW-0723	Serine/threonine-protein kinase
aceK	511145.b4016	UniProt Keywords	KW-0808	Transferase
aceK	511145.b4016	UniProt Keywords	KW-0816	Tricarboxylic acid cycle
aceK	511145.b4016	UniProt Keywords	KW-0904	Protein phosphatase
aceK	511145.b4016	UniProt Keywords	KW-0963	Cytoplasm
aceK	511145.b4016	InterPro	IPR010452	Isocitrate dehydrogenase kinasephosphatase
aceK	511145.b4016	InterPro	IPR046854	Isocitrate dehydrogenase kinase/phosphatase (AceK), regulatory domain
aceK	511145.b4016	InterPro	IPR046855	Isocitrate dehydrogenase kinase/phosphatase (AceK), kinase domain
alaC	511145.b2379	GO Process	GO:0006082	Organic acid metabolic process
alaC	511145.b2379	GO Process	GO:0006090	Pyruvate metabolic process
alaC	511145.b2379	GO Process	GO:0006520	Cellular amino acid metabolic process
alaC	511145.b2379	GO Process	GO:0006522	Alanine metabolic process
alaC	511145.b2379	GO Process	GO:0006523	Alanine biosynthetic process
alaC	511145.b2379	GO Process	GO:0006807	Nitrogen compound metabolic process
alaC	511145.b2379	GO Process	GO:0008152	Metabolic process
alaC	511145.b2379	GO Process	GO:0008652	Cellular amino acid biosynthetic process
alaC	511145.b2379	GO Process	GO:0009058	Biosynthetic process
alaC	511145.b2379	GO Process	GO:0009078	Pyruvate family amino acid metabolic process
alaC	511145.b2379	GO Process	GO:0009079	Pyruvate family amino acid biosynthetic process
alaC	511145.b2379	GO Process	GO:0009987	Cellular process
alaC	511145.b2379	GO Process	GO:0016053	Organic acid biosynthetic process
alaC	511145.b2379	GO Process	GO:0019272	L-alanine biosynthetic process from pyruvate
alaC	511145.b2379	GO Process	GO:0019752	Carboxylic acid metabolic process
alaC	511145.b2379	GO Process	GO:0030632	D-alanine biosynthetic process
alaC	511145.b2379	GO Process	GO:0032787	Monocarboxylic acid metabolic process
alaC	511145.b2379	GO Process	GO:0042851	L-alanine metabolic process
alaC	511145.b2379	GO Process	GO:0042852	L-alanine biosynthetic process
alaC	511145.b2379	GO Process	GO:0043436	Oxoacid metabolic process
alaC	511145.b2379	GO Process	GO:0044237	Cellular metabolic process
alaC	511145.b2379	GO Process	GO:0044238	Primary metabolic process
alaC	511145.b2379	GO Process	GO:0044249	Cellular biosynthetic process
alaC	511145.b2379	GO Process	GO:0044281	Small molecule metabolic process
alaC	511145.b2379	GO Process	GO:0044283	Small molecule biosynthetic process
alaC	511145.b2379	GO Process	GO:0046144	D-alanine family amino acid metabolic process
alaC	511145.b2379	GO Process	GO:0046145	D-alanine family amino acid biosynthetic process
alaC	511145.b2379	GO Process	GO:0046394	Carboxylic acid biosynthetic process
alaC	511145.b2379	GO Process	GO:0046416	D-amino acid metabolic process
alaC	511145.b2379	GO Process	GO:0046436	D-alanine metabolic process
alaC	511145.b2379	GO Process	GO:0046437	D-amino acid biosynthetic process
alaC	511145.b2379	GO Process	GO:0071704	Organic substance metabolic process
alaC	511145.b2379	GO Process	GO:1901564	Organonitrogen compound metabolic process
alaC	511145.b2379	GO Process	GO:1901566	Organonitrogen compound biosynthetic process
alaC	511145.b2379	GO Process	GO:1901576	Organic substance biosynthetic process
alaC	511145.b2379	GO Process	GO:1901605	Alpha-amino acid metabolic process
alaC	511145.b2379	GO Process	GO:1901607	Alpha-amino acid biosynthetic process
alaC	511145.b2379	GO Function	GO:0003824	Catalytic activity
alaC	511145.b2379	GO Function	GO:0004021	L-alanine:2-oxoglutarate aminotransferase activity
alaC	511145.b2379	GO Function	GO:0005488	Binding
alaC	511145.b2379	GO Function	GO:0008483	Transaminase activity
alaC	511145.b2379	GO Function	GO:0016740	Transferase activity
alaC	511145.b2379	GO Function	GO:0016769	Transferase activity, transferring nitrogenous groups
alaC	511145.b2379	GO Function	GO:0019842	Vitamin binding
alaC	511145.b2379	GO Function	GO:0030170	Pyridoxal phosphate binding
alaC	511145.b2379	GO Function	GO:0036094	Small molecule binding
alaC	511145.b2379	GO Function	GO:0043167	Ion binding
alaC	511145.b2379	GO Function	GO:0043168	Anion binding
alaC	511145.b2379	GO Function	GO:0047635	Alanine-oxo-acid transaminase activity
alaC	511145.b2379	GO Function	GO:0070279	Vitamin B6 binding
alaC	511145.b2379	GO Function	GO:0097159	Organic cyclic compound binding
alaC	511145.b2379	GO Function	GO:1901363	Heterocyclic compound binding
alaC	511145.b2379	GO Component	GO:0005622	Intracellular anatomical structure
alaC	511145.b2379	GO Component	GO:0005737	Cytoplasm
alaC	511145.b2379	GO Component	GO:0110165	Cellular anatomical entity
alaC	511145.b2379	STRING clusters	CL:1426	Amino-acid biosynthesis, and Arginine biosynthesis
alaC	511145.b2379	STRING clusters	CL:1427	Amino-acid biosynthesis, and D-alanine metabolic process
alaC	511145.b2379	STRING clusters	CL:1591	L-alanine metabolic process
alaC	511145.b2379	UniProt Keywords	KW-0032	Aminotransferase
alaC	511145.b2379	UniProt Keywords	KW-0663	Pyridoxal phosphate
alaC	511145.b2379	UniProt Keywords	KW-0808	Transferase
alaC	511145.b2379	UniProt Keywords	KW-0963	Cytoplasm
alaC	511145.b2379	Pfam	PF00155	Aminotransferase class I and II
alaC	511145.b2379	InterPro	IPR004839	Aminotransferase, class I/classII
alaC	511145.b2379	InterPro	IPR015421	Pyridoxal phosphate-dependent transferase, major domain
alaC	511145.b2379	InterPro	IPR015422	Pyridoxal phosphate-dependent transferase, small domain
alaC	511145.b2379	InterPro	IPR015424	Pyridoxal phosphate-dependent transferase
aldA	511145.b1415	GO Process	GO:0005975	Carbohydrate metabolic process
aldA	511145.b1415	GO Process	GO:0005996	Monosaccharide metabolic process
aldA	511145.b1415	GO Process	GO:0006004	Fucose metabolic process
aldA	511145.b1415	GO Process	GO:0006082	Organic acid metabolic process
aldA	511145.b1415	GO Process	GO:0006520	Cellular amino acid metabolic process
aldA	511145.b1415	GO Process	GO:0006807	Nitrogen compound metabolic process
aldA	511145.b1415	GO Process	GO:0008152	Metabolic process
aldA	511145.b1415	GO Process	GO:0009056	Catabolic process
aldA	511145.b1415	GO Process	GO:0009063	Cellular amino acid catabolic process
aldA	511145.b1415	GO Process	GO:0009448	Gamma-aminobutyric acid metabolic process
aldA	511145.b1415	GO Process	GO:0009450	Gamma-aminobutyric acid catabolic process
aldA	511145.b1415	GO Process	GO:0009987	Cellular process
aldA	511145.b1415	GO Process	GO:0016052	Carbohydrate catabolic process
aldA	511145.b1415	GO Process	GO:0016054	Organic acid catabolic process
aldA	511145.b1415	GO Process	GO:0019299	Rhamnose metabolic process
aldA	511145.b1415	GO Process	GO:0019301	Rhamnose catabolic process
aldA	511145.b1415	GO Process	GO:0019317	Fucose catabolic process
aldA	511145.b1415	GO Process	GO:0019318	Hexose metabolic process
aldA	511145.b1415	GO Process	GO:0019320	Hexose catabolic process
aldA	511145.b1415	GO Process	GO:0019752	Carboxylic acid metabolic process
aldA	511145.b1415	GO Process	GO:0032787	Monocarboxylic acid metabolic process
aldA	511145.b1415	GO Process	GO:0042354	L-fucose metabolic process
aldA	511145.b1415	GO Process	GO:0042355	L-fucose catabolic process
aldA	511145.b1415	GO Process	GO:0043436	Oxoacid metabolic process
aldA	511145.b1415	GO Process	GO:0044237	Cellular metabolic process
aldA	511145.b1415	GO Process	GO:0044238	Primary metabolic process
aldA	511145.b1415	GO Process	GO:0044248	Cellular catabolic process
aldA	511145.b1415	GO Process	GO:0044281	Small molecule metabolic process
aldA	511145.b1415	GO Process	GO:0044282	Small molecule catabolic process
aldA	511145.b1415	GO Process	GO:0046365	Monosaccharide catabolic process
aldA	511145.b1415	GO Process	GO:0046395	Carboxylic acid catabolic process
aldA	511145.b1415	GO Process	GO:0071704	Organic substance metabolic process
aldA	511145.b1415	GO Process	GO:1901564	Organonitrogen compound metabolic process
aldA	511145.b1415	GO Process	GO:1901565	Organonitrogen compound catabolic process
aldA	511145.b1415	GO Process	GO:1901575	Organic substance catabolic process
aldA	511145.b1415	GO Function	GO:0003824	Catalytic activity
aldA	511145.b1415	GO Function	GO:0004029	Aldehyde dehydrogenase (NAD+) activity
aldA	511145.b1415	GO Function	GO:0004030	Aldehyde dehydrogenase [NAD(P)+] activity
aldA	511145.b1415	GO Function	GO:0004777	Succinate-semialdehyde dehydrogenase (NAD+) activity
aldA	511145.b1415	GO Function	GO:0005488	Binding
aldA	511145.b1415	GO Function	GO:0005515	Protein binding
aldA	511145.b1415	GO Function	GO:0008911	Lactaldehyde dehydrogenase activity
aldA	511145.b1415	GO Function	GO:0009013	Succinate-semialdehyde dehydrogenase [NAD(P)+] activity
aldA	511145.b1415	GO Function	GO:0016491	Oxidoreductase activity
aldA	511145.b1415	GO Function	GO:0016620	Oxidoreductase activity, acting on the aldehyde or oxo group of donors, NAD or NADP as acceptor
aldA	511145.b1415	GO Function	GO:0016903	Oxidoreductase activity, acting on the aldehyde or oxo group of donors
aldA	511145.b1415	GO Function	GO:0042802	Identical protein binding
aldA	511145.b1415	GO Function	GO:0050569	Glycolaldehyde dehydrogenase activity
aldA	511145.b1415	GO Component	GO:0005622	Intracellular anatomical structure
aldA	511145.b1415	GO Component	GO:0005737	Cytoplasm
aldA	511145.b1415	GO Component	GO:0005829	Cytosol
aldA	511145.b1415	GO Component	GO:0032991	Protein-containing complex
aldA	511145.b1415	GO Component	GO:0110165	Cellular anatomical entity
aldA	511145.b1415	STRING clusters	CL:6432	Mixed, incl. LysR, substrate-binding, and Organic acid transmembrane transporter activity
aldA	511145.b1415	STRING clusters	CL:6433	Mixed, incl. LysR, substrate-binding, and Neutral amino acid transmembrane transporter activity
aldA	511145.b1415	STRING clusters	CL:6515	Mixed, incl. Neutral amino acid transmembrane transporter activity, and Ammonia-lyase activity
aldA	511145.b1415	STRING clusters	CL:6516	Mixed, incl. Ammonia-lyase activity, and Serine transmembrane transporter activity
aldA	511145.b1415	STRING clusters	CL:6518	Mixed, incl. Serine dehydratase beta chain, and Methylglyoxal catabolic process to D-lactate via S-lactoyl-glutathione
aldA	511145.b1415	STRING clusters	CL:6519	Methylglyoxal catabolic process to D-lactate via S-lactoyl-glutathione, and Glycerate dehydrogenase activity
aldA	511145.b1415	KEGG	eco00620	Pyruvate metabolism
aldA	511145.b1415	KEGG	eco00630	Glyoxylate and dicarboxylate metabolism
aldA	511145.b1415	KEGG	eco01100	Metabolic pathways
aldA	511145.b1415	KEGG	eco01120	Microbial metabolism in diverse environments
aldA	511145.b1415	COMPARTMENTS	GOCC:0005622	Intracellular
aldA	511145.b1415	COMPARTMENTS	GOCC:0005737	Cytoplasm
aldA	511145.b1415	COMPARTMENTS	GOCC:0005829	Cytosol
aldA	511145.b1415	COMPARTMENTS	GOCC:0032991	Protein-containing complex
aldA	511145.b1415	COMPARTMENTS	GOCC:0110165	Cellular anatomical entity
aldA	511145.b1415	UniProt Keywords	KW-0520	NAD
aldA	511145.b1415	UniProt Keywords	KW-0560	Oxidoreductase
aldA	511145.b1415	Pfam	PF00171	Aldehyde dehydrogenase family
aldA	511145.b1415	InterPro	IPR015590	Aldehyde dehydrogenase domain
aldA	511145.b1415	InterPro	IPR016160	Aldehyde dehydrogenase, cysteine active site
aldA	511145.b1415	InterPro	IPR016161	Aldehyde/histidinol dehydrogenase
aldA	511145.b1415	InterPro	IPR016162	Aldehyde dehydrogenase, N-terminal
aldA	511145.b1415	InterPro	IPR016163	Aldehyde dehydrogenase, C-terminal
aldA	511145.b1415	InterPro	IPR029510	Aldehyde dehydrogenase, glutamic acid active site
alpA	511145.b2624	GO Process	GO:0006355	Regulation of transcription, DNA-templated
alpA	511145.b2624	GO Process	GO:0009889	Regulation of biosynthetic process
alpA	511145.b2624	GO Process	GO:0009891	Positive regulation of biosynthetic process
alpA	511145.b2624	GO Process	GO:0009893	Positive regulation of metabolic process
alpA	511145.b2624	GO Process	GO:0009987	Cellular process
alpA	511145.b2624	GO Process	GO:0010468	Regulation of gene expression
alpA	511145.b2624	GO Process	GO:0010556	Regulation of macromolecule biosynthetic process
alpA	511145.b2624	GO Process	GO:0010557	Positive regulation of macromolecule biosynthetic process
alpA	511145.b2624	GO Process	GO:0010604	Positive regulation of macromolecule metabolic process
alpA	511145.b2624	GO Process	GO:0019219	Regulation of nucleobase-containing compound metabolic process
alpA	511145.b2624	GO Process	GO:0019222	Regulation of metabolic process
alpA	511145.b2624	GO Process	GO:0031323	Regulation of cellular metabolic process
alpA	511145.b2624	GO Process	GO:0031325	Positive regulation of cellular metabolic process
alpA	511145.b2624	GO Process	GO:0031326	Regulation of cellular biosynthetic process
alpA	511145.b2624	GO Process	GO:0031328	Positive regulation of cellular biosynthetic process
alpA	511145.b2624	GO Process	GO:0042710	Biofilm formation
alpA	511145.b2624	GO Process	GO:0044010	Single-species biofilm formation
alpA	511145.b2624	GO Process	GO:0045893	Positive regulation of transcription, DNA-templated
alpA	511145.b2624	GO Process	GO:0045935	Positive regulation of nucleobase-containing compound metabolic process
alpA	511145.b2624	GO Process	GO:0048518	Positive regulation of biological process
alpA	511145.b2624	GO Process	GO:0048522	Positive regulation of cellular process
alpA	511145.b2624	GO Process	GO:0050789	Regulation of biological process
alpA	511145.b2624	GO Process	GO:0050794	Regulation of cellular process
alpA	511145.b2624	GO Process	GO:0051171	Regulation of nitrogen compound metabolic process
alpA	511145.b2624	GO Process	GO:0051173	Positive regulation of nitrogen compound metabolic process
alpA	511145.b2624	GO Process	GO:0051252	Regulation of RNA metabolic process
alpA	511145.b2624	GO Process	GO:0051254	Positive regulation of RNA metabolic process
alpA	511145.b2624	GO Process	GO:0051703	Biological process involved in intraspecies interaction between organisms
alpA	511145.b2624	GO Process	GO:0060255	Regulation of macromolecule metabolic process
alpA	511145.b2624	GO Process	GO:0065007	Biological regulation
alpA	511145.b2624	GO Process	GO:0080090	Regulation of primary metabolic process
alpA	511145.b2624	GO Process	GO:0098630	Aggregation of unicellular organisms
alpA	511145.b2624	GO Process	GO:0098743	Cell aggregation
alpA	511145.b2624	GO Process	GO:1902680	Positive regulation of RNA biosynthetic process
alpA	511145.b2624	GO Process	GO:1903506	Regulation of nucleic acid-templated transcription
alpA	511145.b2624	GO Process	GO:1903508	Positive regulation of nucleic acid-templated transcription
alpA	511145.b2624	GO Process	GO:2001141	Regulation of RNA biosynthetic process
alpA	511145.b2624	GO Function	GO:0003676	Nucleic acid binding
alpA	511145.b2624	GO Function	GO:0003677	DNA binding
alpA	511145.b2624	GO Function	GO:0005488	Binding
alpA	511145.b2624	GO Function	GO:0097159	Organic cyclic compound binding
alpA	511145.b2624	GO Function	GO:1901363	Heterocyclic compound binding
alpA	511145.b2624	STRING clusters	CL:7781	Mixed, incl. Toxin-antitoxin system, and Viral process
alpA	511145.b2624	STRING clusters	CL:7782	Mixed, incl. Toxin-antitoxin system, and Viral process
alpA	511145.b2624	STRING clusters	CL:7916	Mixed, incl. Viral process, and Bacteriophage T4, Gp38, tail fibre assembly
alpA	511145.b2624	STRING clusters	CL:8012	Mixed, incl. Viral genome integration, and Inovirus Gp2
alpA	511145.b2624	UniProt Keywords	KW-0010	Activator
alpA	511145.b2624	UniProt Keywords	KW-0238	DNA-binding
alpA	511145.b2624	UniProt Keywords	KW-0804	Transcription
alpA	511145.b2624	UniProt Keywords	KW-0805	Transcription regulation
alpA	511145.b2624	Pfam	PF05930	Prophage CP4-57 regulatory protein (AlpA)
alpA	511145.b2624	InterPro	IPR010260	DNA-binding transcriptional activator AlpA
anmK	511145.b1640	GO Process	GO:0000270	Peptidoglycan metabolic process
anmK	511145.b1640	GO Process	GO:0005975	Carbohydrate metabolic process
anmK	511145.b1640	GO Process	GO:0006022	Aminoglycan metabolic process
anmK	511145.b1640	GO Process	GO:0006040	Amino sugar metabolic process
anmK	511145.b1640	GO Process	GO:0006082	Organic acid metabolic process
anmK	511145.b1640	GO Process	GO:0006793	Phosphorus metabolic process
anmK	511145.b1640	GO Process	GO:0006796	Phosphate-containing compound metabolic process
anmK	511145.b1640	GO Process	GO:0006807	Nitrogen compound metabolic process
anmK	511145.b1640	GO Process	GO:0008152	Metabolic process
anmK	511145.b1640	GO Process	GO:0009056	Catabolic process
anmK	511145.b1640	GO Process	GO:0009254	Peptidoglycan turnover
anmK	511145.b1640	GO Process	GO:0009987	Cellular process
anmK	511145.b1640	GO Process	GO:0016054	Organic acid catabolic process
anmK	511145.b1640	GO Process	GO:0016310	Phosphorylation
anmK	511145.b1640	GO Process	GO:0019752	Carboxylic acid metabolic process
anmK	511145.b1640	GO Process	GO:0030203	Glycosaminoglycan metabolic process
anmK	511145.b1640	GO Process	GO:0032787	Monocarboxylic acid metabolic process
anmK	511145.b1640	GO Process	GO:0043170	Macromolecule metabolic process
anmK	511145.b1640	GO Process	GO:0043436	Oxoacid metabolic process
anmK	511145.b1640	GO Process	GO:0044237	Cellular metabolic process
anmK	511145.b1640	GO Process	GO:0044238	Primary metabolic process
anmK	511145.b1640	GO Process	GO:0044248	Cellular catabolic process
anmK	511145.b1640	GO Process	GO:0044281	Small molecule metabolic process
anmK	511145.b1640	GO Process	GO:0044282	Small molecule catabolic process
anmK	511145.b1640	GO Process	GO:0046395	Carboxylic acid catabolic process
anmK	511145.b1640	GO Process	GO:0071704	Organic substance metabolic process
anmK	511145.b1640	GO Process	GO:0072329	Monocarboxylic acid catabolic process
anmK	511145.b1640	GO Process	GO:0097174	1,6-anhydro-N-acetyl-beta-muramic acid metabolic process
anmK	511145.b1640	GO Process	GO:0097175	1,6-anhydro-N-acetyl-beta-muramic acid catabolic process
anmK	511145.b1640	GO Process	GO:1901135	Carbohydrate derivative metabolic process
anmK	511145.b1640	GO Process	GO:1901564	Organonitrogen compound metabolic process
anmK	511145.b1640	GO Process	GO:1901575	Organic substance catabolic process
anmK	511145.b1640	GO Function	GO:0000166	Nucleotide binding
anmK	511145.b1640	GO Function	GO:0003824	Catalytic activity
anmK	511145.b1640	GO Function	GO:0005488	Binding
anmK	511145.b1640	GO Function	GO:0005524	ATP binding
anmK	511145.b1640	GO Function	GO:0016301	Kinase activity
anmK	511145.b1640	GO Function	GO:0016740	Transferase activity
anmK	511145.b1640	GO Function	GO:0016772	Transferase activity, transferring phosphorus-containing groups
anmK	511145.b1640	GO Function	GO:0016773	Phosphotransferase activity, alcohol group as acceptor
anmK	511145.b1640	GO Function	GO:0017076	Purine nucleotide binding
anmK	511145.b1640	GO Function	GO:0030554	Adenyl nucleotide binding
anmK	511145.b1640	GO Function	GO:0032553	Ribonucleotide binding
anmK	511145.b1640	GO Function	GO:0032555	Purine ribonucleotide binding
anmK	511145.b1640	GO Function	GO:0032559	Adenyl ribonucleotide binding
anmK	511145.b1640	GO Function	GO:0035639	Purine ribonucleoside triphosphate binding
anmK	511145.b1640	GO Function	GO:0036094	Small molecule binding
anmK	511145.b1640	GO Function	GO:0043167	Ion binding
anmK	511145.b1640	GO Function	GO:0043168	Anion binding
anmK	511145.b1640	GO Function	GO:0097159	Organic cyclic compound binding
anmK	511145.b1640	GO Function	GO:0097367	Carbohydrate derivative binding
anmK	511145.b1640	GO Function	GO:1901265	Nucleoside phosphate binding
anmK	511145.b1640	GO Function	GO:1901363	Heterocyclic compound binding
anmK	511145.b1640	STRING clusters	CL:5082	Mixed, incl. Phosphoenolpyruvate-dependent sugar phosphotransferase system, and Carbohydrate metabolism
anmK	511145.b1640	STRING clusters	CL:5083	Mixed, incl. Phosphoenolpyruvate-dependent sugar phosphotransferase system, and Amino sugar metabolic process
anmK	511145.b1640	STRING clusters	CL:5257	Mixed, incl. Amino sugar metabolic process, and Peptidoglycan turnover
anmK	511145.b1640	STRING clusters	CL:5258	Mixed, incl. Amino sugar metabolic process, and Peptidoglycan turnover
anmK	511145.b1640	STRING clusters	CL:5259	Peptidoglycan turnover, and Chitin degradation
anmK	511145.b1640	STRING clusters	CL:5261	Peptidoglycan turnover, and Peptidoglycan transport
anmK	511145.b1640	UniProt Keywords	KW-0067	ATP-binding
anmK	511145.b1640	UniProt Keywords	KW-0119	Carbohydrate metabolism
anmK	511145.b1640	UniProt Keywords	KW-0418	Kinase
anmK	511145.b1640	UniProt Keywords	KW-0547	Nucleotide-binding
anmK	511145.b1640	UniProt Keywords	KW-0808	Transferase
anmK	511145.b1640	Pfam	PF03702	Anhydro-N-acetylmuramic acid kinase
anmK	511145.b1640	InterPro	IPR005338	Anhydro-N-acetylmuramic acid kinase
anmK	511145.b1640	InterPro	IPR043129	ATPase, nucleotide binding domain
argH	511145.b3960	GO Process	GO:0006082	Organic acid metabolic process
argH	511145.b3960	GO Process	GO:0006520	Cellular amino acid metabolic process
argH	511145.b3960	GO Process	GO:0006525	Arginine metabolic process
argH	511145.b3960	GO Process	GO:0006526	Arginine biosynthetic process
argH	511145.b3960	GO Process	GO:0006591	Ornithine metabolic process
argH	511145.b3960	GO Process	GO:0006807	Nitrogen compound metabolic process
argH	511145.b3960	GO Process	GO:0008152	Metabolic process
argH	511145.b3960	GO Process	GO:0008652	Cellular amino acid biosynthetic process
argH	511145.b3960	GO Process	GO:0009058	Biosynthetic process
argH	511145.b3960	GO Process	GO:0009064	Glutamine family amino acid metabolic process
argH	511145.b3960	GO Process	GO:0009084	Glutamine family amino acid biosynthetic process
argH	511145.b3960	GO Process	GO:0009987	Cellular process
argH	511145.b3960	GO Process	GO:0016053	Organic acid biosynthetic process
argH	511145.b3960	GO Process	GO:0019752	Carboxylic acid metabolic process
argH	511145.b3960	GO Process	GO:0042450	Arginine biosynthetic process via ornithine
argH	511145.b3960	GO Process	GO:0043436	Oxoacid metabolic process
argH	511145.b3960	GO Process	GO:0044237	Cellular metabolic process
argH	511145.b3960	GO Process	GO:0044238	Primary metabolic process
argH	511145.b3960	GO Process	GO:0044249	Cellular biosynthetic process
argH	511145.b3960	GO Process	GO:0044281	Small molecule metabolic process
argH	511145.b3960	GO Process	GO:0044283	Small molecule biosynthetic process
argH	511145.b3960	GO Process	GO:0046394	Carboxylic acid biosynthetic process
argH	511145.b3960	GO Process	GO:0071704	Organic substance metabolic process
argH	511145.b3960	GO Process	GO:1901564	Organonitrogen compound metabolic process
argH	511145.b3960	GO Process	GO:1901566	Organonitrogen compound biosynthetic process
argH	511145.b3960	GO Process	GO:1901576	Organic substance biosynthetic process
argH	511145.b3960	GO Process	GO:1901605	Alpha-amino acid metabolic process
argH	511145.b3960	GO Process	GO:1901607	Alpha-amino acid biosynthetic process
argH	511145.b3960	GO Function	GO:0003824	Catalytic activity
argH	511145.b3960	GO Function	GO:0004056	Argininosuccinate lyase activity
argH	511145.b3960	GO Function	GO:0016829	Lyase activity
argH	511145.b3960	GO Function	GO:0016840	Carbon-nitrogen lyase activity
argH	511145.b3960	GO Function	GO:0016842	Amidine-lyase activity
argH	511145.b3960	GO Component	GO:0005622	Intracellular anatomical structure
argH	511145.b3960	GO Component	GO:0005737	Cytoplasm
argH	511145.b3960	GO Component	GO:0005829	Cytosol
argH	511145.b3960	GO Component	GO:0110165	Cellular anatomical entity
argH	511145.b3960	STRING clusters	CL:1426	Amino-acid biosynthesis, and Arginine biosynthesis
argH	511145.b3960	STRING clusters	CL:1600	Mixed, incl. Glutamine family amino acid biosynthetic process, and Asparagine metabolic process
argH	511145.b3960	STRING clusters	CL:1601	Glutamine family amino acid biosynthetic process, and Regulation of nitrogen utilization
argH	511145.b3960	STRING clusters	CL:1603	Arginine biosynthesis, and Proline metabolic process
argH	511145.b3960	STRING clusters	CL:1607	Arginine biosynthesis
argH	511145.b3960	KEGG	eco00220	Arginine biosynthesis
argH	511145.b3960	KEGG	eco00250	Alanine, aspartate and glutamate metabolism
argH	511145.b3960	KEGG	eco01100	Metabolic pathways
argH	511145.b3960	KEGG	eco01110	Biosynthesis of secondary metabolites
argH	511145.b3960	KEGG	eco01230	Biosynthesis of amino acids
argH	511145.b3960	UniProt Keywords	KW-0028	Amino-acid biosynthesis
argH	511145.b3960	UniProt Keywords	KW-0055	Arginine biosynthesis
argH	511145.b3960	UniProt Keywords	KW-0456	Lyase
argH	511145.b3960	UniProt Keywords	KW-0963	Cytoplasm
argH	511145.b3960	Pfam	PF00206	Lyase
argH	511145.b3960	Pfam	PF14698	Argininosuccinate lyase C-terminal
argH	511145.b3960	InterPro	IPR000362	Fumarate lyase family
argH	511145.b3960	InterPro	IPR008948	L-Aspartase-like
argH	511145.b3960	InterPro	IPR009049	Argininosuccinate lyase
argH	511145.b3960	InterPro	IPR020557	Fumarate lyase, conserved site
argH	511145.b3960	InterPro	IPR022761	Fumarate lyase, N-terminal
argH	511145.b3960	InterPro	IPR024083	Fumarase/histidase, N-terminal
argH	511145.b3960	InterPro	IPR029419	Argininosuccinate lyase, C-terminal
aroA	511145.b0908	GO Process	GO:0006082	Organic acid metabolic process
aroA	511145.b0908	GO Process	GO:0006520	Cellular amino acid metabolic process
aroA	511145.b0908	GO Process	GO:0006725	Cellular aromatic compound metabolic process
aroA	511145.b0908	GO Process	GO:0006807	Nitrogen compound metabolic process
aroA	511145.b0908	GO Process	GO:0008152	Metabolic process
aroA	511145.b0908	GO Process	GO:0008652	Cellular amino acid biosynthetic process
aroA	511145.b0908	GO Process	GO:0009058	Biosynthetic process
aroA	511145.b0908	GO Process	GO:0009072	Aromatic amino acid family metabolic process
aroA	511145.b0908	GO Process	GO:0009073	Aromatic amino acid family biosynthetic process
aroA	511145.b0908	GO Process	GO:0009423	Chorismate biosynthetic process
aroA	511145.b0908	GO Process	GO:0009987	Cellular process
aroA	511145.b0908	GO Process	GO:0016053	Organic acid biosynthetic process
aroA	511145.b0908	GO Process	GO:0019438	Aromatic compound biosynthetic process
aroA	511145.b0908	GO Process	GO:0019752	Carboxylic acid metabolic process
aroA	511145.b0908	GO Process	GO:0043436	Oxoacid metabolic process
aroA	511145.b0908	GO Process	GO:0043648	Dicarboxylic acid metabolic process
aroA	511145.b0908	GO Process	GO:0043650	Dicarboxylic acid biosynthetic process
aroA	511145.b0908	GO Process	GO:0044237	Cellular metabolic process
aroA	511145.b0908	GO Process	GO:0044238	Primary metabolic process
aroA	511145.b0908	GO Process	GO:0044249	Cellular biosynthetic process
aroA	511145.b0908	GO Process	GO:0044281	Small molecule metabolic process
aroA	511145.b0908	GO Process	GO:0044283	Small molecule biosynthetic process
aroA	511145.b0908	GO Process	GO:0046394	Carboxylic acid biosynthetic process
aroA	511145.b0908	GO Process	GO:0046417	Chorismate metabolic process
aroA	511145.b0908	GO Process	GO:0071704	Organic substance metabolic process
aroA	511145.b0908	GO Process	GO:1901360	Organic cyclic compound metabolic process
aroA	511145.b0908	GO Process	GO:1901362	Organic cyclic compound biosynthetic process
aroA	511145.b0908	GO Process	GO:1901564	Organonitrogen compound metabolic process
aroA	511145.b0908	GO Process	GO:1901566	Organonitrogen compound biosynthetic process
aroA	511145.b0908	GO Process	GO:1901576	Organic substance biosynthetic process
aroA	511145.b0908	GO Function	GO:0003824	Catalytic activity
aroA	511145.b0908	GO Function	GO:0003866	3-phosphoshikimate 1-carboxyvinyltransferase activity
aroA	511145.b0908	GO Function	GO:0016740	Transferase activity
aroA	511145.b0908	GO Function	GO:0016765	Transferase activity, transferring alkyl or aryl (other than methyl) groups
aroA	511145.b0908	GO Component	GO:0005622	Intracellular anatomical structure
aroA	511145.b0908	GO Component	GO:0005737	Cytoplasm
aroA	511145.b0908	GO Component	GO:0005829	Cytosol
aroA	511145.b0908	GO Component	GO:0110165	Cellular anatomical entity
aroA	511145.b0908	STRING clusters	CL:1426	Amino-acid biosynthesis, and Arginine biosynthesis
aroA	511145.b0908	STRING clusters	CL:1427	Amino-acid biosynthesis, and D-alanine metabolic process
aroA	511145.b0908	STRING clusters	CL:1428	Amino-acid biosynthesis, and Aromatic amino acid transmembrane transporter activity
aroA	511145.b0908	STRING clusters	CL:1528	Aromatic amino acid family biosynthetic process, and Tryptophan/tyrosine transporter
aroA	511145.b0908	STRING clusters	CL:1530	Aromatic amino acid family biosynthetic process
aroA	511145.b0908	STRING clusters	CL:1531	Chorismate biosynthetic process
aroA	511145.b0908	STRING clusters	CL:1532	Chorismate biosynthetic process
aroA	511145.b0908	KEGG	eco00400	Phenylalanine, tyrosine and tryptophan biosynthesis
aroA	511145.b0908	KEGG	eco01100	Metabolic pathways
aroA	511145.b0908	KEGG	eco01110	Biosynthesis of secondary metabolites
aroA	511145.b0908	KEGG	eco01230	Biosynthesis of amino acids
aroA	511145.b0908	COMPARTMENTS	GOCC:0005622	Intracellular
aroA	511145.b0908	COMPARTMENTS	GOCC:0005737	Cytoplasm
aroA	511145.b0908	COMPARTMENTS	GOCC:0005829	Cytosol
aroA	511145.b0908	COMPARTMENTS	GOCC:0110165	Cellular anatomical entity
aroA	511145.b0908	UniProt Keywords	KW-0028	Amino-acid biosynthesis
aroA	511145.b0908	UniProt Keywords	KW-0057	Aromatic amino acid biosynthesis
aroA	511145.b0908	UniProt Keywords	KW-0808	Transferase
aroA	511145.b0908	UniProt Keywords	KW-0963	Cytoplasm
aroA	511145.b0908	Pfam	PF00275	EPSP synthase (3-phosphoshikimate 1-carboxyvinyltransferase)
aroA	511145.b0908	InterPro	IPR001986	Enolpyruvate transferase domain
aroA	511145.b0908	InterPro	IPR006264	3-phosphoshikimate 1-carboxyvinyltransferase
aroA	511145.b0908	InterPro	IPR013792	RNA 3-terminal phosphate cyclase/enolpyruvate transferase, alpha/beta
aroA	511145.b0908	InterPro	IPR023193	3-phosphoshikimate 1-carboxyvinyltransferase, conserved site
aroA	511145.b0908	InterPro	IPR036968	Enolpyruvate transferase domain superfamily
aroC	511145.b2329	GO Process	GO:0006082	Organic acid metabolic process
aroC	511145.b2329	GO Process	GO:0006520	Cellular amino acid metabolic process
aroC	511145.b2329	GO Process	GO:0006725	Cellular aromatic compound metabolic process
aroC	511145.b2329	GO Process	GO:0006807	Nitrogen compound metabolic process
aroC	511145.b2329	GO Process	GO:0008152	Metabolic process
aroC	511145.b2329	GO Process	GO:0008652	Cellular amino acid biosynthetic process
aroC	511145.b2329	GO Process	GO:0009058	Biosynthetic process
aroC	511145.b2329	GO Process	GO:0009072	Aromatic amino acid family metabolic process
aroC	511145.b2329	GO Process	GO:0009073	Aromatic amino acid family biosynthetic process
aroC	511145.b2329	GO Process	GO:0009423	Chorismate biosynthetic process
aroC	511145.b2329	GO Process	GO:0009987	Cellular process
aroC	511145.b2329	GO Process	GO:0016053	Organic acid biosynthetic process
aroC	511145.b2329	GO Process	GO:0019438	Aromatic compound biosynthetic process
aroC	511145.b2329	GO Process	GO:0019752	Carboxylic acid metabolic process
aroC	511145.b2329	GO Process	GO:0043436	Oxoacid metabolic process
aroC	511145.b2329	GO Process	GO:0043648	Dicarboxylic acid metabolic process
aroC	511145.b2329	GO Process	GO:0043650	Dicarboxylic acid biosynthetic process
aroC	511145.b2329	GO Process	GO:0044237	Cellular metabolic process
aroC	511145.b2329	GO Process	GO:0044238	Primary metabolic process
aroC	511145.b2329	GO Process	GO:0044249	Cellular biosynthetic process
aroC	511145.b2329	GO Process	GO:0044281	Small molecule metabolic process
aroC	511145.b2329	GO Process	GO:0044283	Small molecule biosynthetic process
aroC	511145.b2329	GO Process	GO:0046394	Carboxylic acid biosynthetic process
aroC	511145.b2329	GO Process	GO:0046417	Chorismate metabolic process
aroC	511145.b2329	GO Process	GO:0071704	Organic substance metabolic process
aroC	511145.b2329	GO Process	GO:1901360	Organic cyclic compound metabolic process
aroC	511145.b2329	GO Process	GO:1901362	Organic cyclic compound biosynthetic process
aroC	511145.b2329	GO Process	GO:1901564	Organonitrogen compound metabolic process
aroC	511145.b2329	GO Process	GO:1901566	Organonitrogen compound biosynthetic process
aroC	511145.b2329	GO Process	GO:1901576	Organic substance biosynthetic process
aroC	511145.b2329	GO Function	GO:0000166	Nucleotide binding
aroC	511145.b2329	GO Function	GO:0003824	Catalytic activity
aroC	511145.b2329	GO Function	GO:0004107	Chorismate synthase activity
aroC	511145.b2329	GO Function	GO:0005488	Binding
aroC	511145.b2329	GO Function	GO:0005515	Protein binding
aroC	511145.b2329	GO Function	GO:0010181	FMN binding
aroC	511145.b2329	GO Function	GO:0016829	Lyase activity
aroC	511145.b2329	GO Function	GO:0016835	Carbon-oxygen lyase activity
aroC	511145.b2329	GO Function	GO:0016838	Carbon-oxygen lyase activity, acting on phosphates
aroC	511145.b2329	GO Function	GO:0032553	Ribonucleotide binding
aroC	511145.b2329	GO Function	GO:0036094	Small molecule binding
aroC	511145.b2329	GO Function	GO:0042802	Identical protein binding
aroC	511145.b2329	GO Function	GO:0043167	Ion binding
aroC	511145.b2329	GO Function	GO:0043168	Anion binding
aroC	511145.b2329	GO Function	GO:0097159	Organic cyclic compound binding
aroC	511145.b2329	GO Function	GO:0097367	Carbohydrate derivative binding
aroC	511145.b2329	GO Function	GO:1901265	Nucleoside phosphate binding
aroC	511145.b2329	GO Function	GO:1901363	Heterocyclic compound binding
aroC	511145.b2329	GO Component	GO:0005622	Intracellular anatomical structure
aroC	511145.b2329	GO Component	GO:0005737	Cytoplasm
aroC	511145.b2329	GO Component	GO:0005829	Cytosol
aroC	511145.b2329	GO Component	GO:0110165	Cellular anatomical entity
aroC	511145.b2329	STRING clusters	CL:1426	Amino-acid biosynthesis, and Arginine biosynthesis
aroC	511145.b2329	STRING clusters	CL:1427	Amino-acid biosynthesis, and D-alanine metabolic process
aroC	511145.b2329	STRING clusters	CL:1428	Amino-acid biosynthesis, and Aromatic amino acid transmembrane transporter activity
aroC	511145.b2329	STRING clusters	CL:1528	Aromatic amino acid family biosynthetic process, and Tryptophan/tyrosine transporter
aroC	511145.b2329	STRING clusters	CL:1530	Aromatic amino acid family biosynthetic process
aroC	511145.b2329	STRING clusters	CL:1531	Chorismate biosynthetic process
aroC	511145.b2329	STRING clusters	CL:1532	Chorismate biosynthetic process
aroC	511145.b2329	KEGG	eco00400	Phenylalanine, tyrosine and tryptophan biosynthesis
aroC	511145.b2329	KEGG	eco01100	Metabolic pathways
aroC	511145.b2329	KEGG	eco01110	Biosynthesis of secondary metabolites
aroC	511145.b2329	KEGG	eco01230	Biosynthesis of amino acids
aroC	511145.b2329	COMPARTMENTS	GOCC:0005622	Intracellular
aroC	511145.b2329	COMPARTMENTS	GOCC:0005737	Cytoplasm
aroC	511145.b2329	COMPARTMENTS	GOCC:0005829	Cytosol
aroC	511145.b2329	COMPARTMENTS	GOCC:0110165	Cellular anatomical entity
aroC	511145.b2329	UniProt Keywords	KW-0028	Amino-acid biosynthesis
aroC	511145.b2329	UniProt Keywords	KW-0057	Aromatic amino acid biosynthesis
aroC	511145.b2329	UniProt Keywords	KW-0274	FAD
aroC	511145.b2329	UniProt Keywords	KW-0285	Flavoprotein
aroC	511145.b2329	UniProt Keywords	KW-0288	FMN
aroC	511145.b2329	UniProt Keywords	KW-0456	Lyase
aroC	511145.b2329	UniProt Keywords	KW-0521	NADP
aroC	511145.b2329	Pfam	PF01264	Chorismate synthase
aroC	511145.b2329	InterPro	IPR000453	Chorismate synthase
aroC	511145.b2329	InterPro	IPR020541	Chorismate synthase, conserved site
aroC	511145.b2329	InterPro	IPR035904	Chorismate synthase AroC superfamily
aslA	511145.b3801	GO Function	GO:0003824	Catalytic activity
aslA	511145.b3801	GO Function	GO:0004065	Arylsulfatase activity
aslA	511145.b3801	GO Function	GO:0005488	Binding
aslA	511145.b3801	GO Function	GO:0008484	Sulfuric ester hydrolase activity
aslA	511145.b3801	GO Function	GO:0016787	Hydrolase activity
aslA	511145.b3801	GO Function	GO:0016788	Hydrolase activity, acting on ester bonds
aslA	511145.b3801	GO Function	GO:0043167	Ion binding
aslA	511145.b3801	GO Function	GO:0043169	Cation binding
aslA	511145.b3801	GO Function	GO:0046872	Metal ion binding
aslA	511145.b3801	GO Component	GO:0042597	Periplasmic space
aslA	511145.b3801	GO Component	GO:0110165	Cellular anatomical entity
aslA	511145.b3801	STRING clusters	CL:6765	Mixed, incl. DNA recombination, and Hydrolase activity, acting on ester bonds
aslA	511145.b3801	STRING clusters	CL:6766	Mostly uncharacterized, incl. Restriction system, and Aldaric acid metabolic process
aslA	511145.b3801	STRING clusters	CL:6767	Mostly uncharacterized, incl. Aldaric acid metabolic process, and Purine metabolism
aslA	511145.b3801	STRING clusters	CL:6768	Mostly uncharacterized, incl. Sulfatase, N-terminal, and Anaerobic sulphatase maturase, radical SAM
aslA	511145.b3801	STRING clusters	CL:6769	Mostly uncharacterized, incl. Sulfatase, N-terminal, and Anaerobic sulphatase maturase, radical SAM
aslA	511145.b3801	STRING clusters	CL:6850	Mixed, incl. Sulfatase, conserved site, and Anaerobic sulphatase maturase, radical SAM
aslA	511145.b3801	STRING clusters	CL:6852	Anaerobic sulphatase maturase, radical SAM, and Sulfatase, conserved site
aslA	511145.b3801	UniProt Keywords	KW-0106	Calcium
aslA	511145.b3801	UniProt Keywords	KW-0378	Hydrolase
aslA	511145.b3801	UniProt Keywords	KW-0479	Metal-binding
aslA	511145.b3801	UniProt Keywords	KW-0574	Periplasm
aslA	511145.b3801	UniProt Keywords	KW-0732	Signal
aslA	511145.b3801	Pfam	PF00884	Sulfatase
aslA	511145.b3801	InterPro	IPR000917	Sulfatase, N-terminal
aslA	511145.b3801	InterPro	IPR017850	Alkaline-phosphatase-like, core domain superfamily
aslA	511145.b3801	InterPro	IPR024607	Sulfatase, conserved site
betA	511145.b0311	GO Process	GO:0006575	Cellular modified amino acid metabolic process
betA	511145.b0311	GO Process	GO:0006577	Amino-acid betaine metabolic process
betA	511145.b0311	GO Process	GO:0006578	Amino-acid betaine biosynthetic process
betA	511145.b0311	GO Process	GO:0006807	Nitrogen compound metabolic process
betA	511145.b0311	GO Process	GO:0006950	Response to stress
betA	511145.b0311	GO Process	GO:0006970	Response to osmotic stress
betA	511145.b0311	GO Process	GO:0008152	Metabolic process
betA	511145.b0311	GO Process	GO:0009058	Biosynthetic process
betA	511145.b0311	GO Process	GO:0009628	Response to abiotic stimulus
betA	511145.b0311	GO Process	GO:0009987	Cellular process
betA	511145.b0311	GO Process	GO:0019285	Glycine betaine biosynthetic process from choline
betA	511145.b0311	GO Process	GO:0019695	Choline metabolic process
betA	511145.b0311	GO Process	GO:0031455	Glycine betaine metabolic process
betA	511145.b0311	GO Process	GO:0031456	Glycine betaine biosynthetic process
betA	511145.b0311	GO Process	GO:0034641	Cellular nitrogen compound metabolic process
betA	511145.b0311	GO Process	GO:0042398	Cellular modified amino acid biosynthetic process
betA	511145.b0311	GO Process	GO:0044237	Cellular metabolic process
betA	511145.b0311	GO Process	GO:0044249	Cellular biosynthetic process
betA	511145.b0311	GO Process	GO:0044271	Cellular nitrogen compound biosynthetic process
betA	511145.b0311	GO Process	GO:0050896	Response to stimulus
betA	511145.b0311	GO Process	GO:0071704	Organic substance metabolic process
betA	511145.b0311	GO Process	GO:0097164	Ammonium ion metabolic process
betA	511145.b0311	GO Process	GO:1901564	Organonitrogen compound metabolic process
betA	511145.b0311	GO Process	GO:1901566	Organonitrogen compound biosynthetic process
betA	511145.b0311	GO Process	GO:1901576	Organic substance biosynthetic process
betA	511145.b0311	GO Function	GO:0000166	Nucleotide binding
betA	511145.b0311	GO Function	GO:0003824	Catalytic activity
betA	511145.b0311	GO Function	GO:0004029	Aldehyde dehydrogenase (NAD+) activity
betA	511145.b0311	GO Function	GO:0004030	Aldehyde dehydrogenase [NAD(P)+] activity
betA	511145.b0311	GO Function	GO:0005488	Binding
betA	511145.b0311	GO Function	GO:0008802	Betaine-aldehyde dehydrogenase activity
betA	511145.b0311	GO Function	GO:0008812	Choline dehydrogenase activity
betA	511145.b0311	GO Function	GO:0016491	Oxidoreductase activity
betA	511145.b0311	GO Function	GO:0016614	Oxidoreductase activity, acting on CH-OH group of donors
betA	511145.b0311	GO Function	GO:0016620	Oxidoreductase activity, acting on the aldehyde or oxo group of donors, NAD or NADP as acceptor
betA	511145.b0311	GO Function	GO:0016903	Oxidoreductase activity, acting on the aldehyde or oxo group of donors
betA	511145.b0311	GO Function	GO:0036094	Small molecule binding
betA	511145.b0311	GO Function	GO:0043167	Ion binding
betA	511145.b0311	GO Function	GO:0043168	Anion binding
betA	511145.b0311	GO Function	GO:0050660	Flavin adenine dinucleotide binding
betA	511145.b0311	GO Function	GO:0097159	Organic cyclic compound binding
betA	511145.b0311	GO Function	GO:1901265	Nucleoside phosphate binding
betA	511145.b0311	GO Function	GO:1901363	Heterocyclic compound binding
betA	511145.b0311	GO Component	GO:0005886	Plasma membrane
betA	511145.b0311	GO Component	GO:0016020	Membrane
betA	511145.b0311	GO Component	GO:0071944	Cell periphery
betA	511145.b0311	GO Component	GO:0110165	Cellular anatomical entity
betA	511145.b0311	STRING clusters	CL:6207	Mostly uncharacterized, incl. Degradation of aromatic compounds, and Uracil catabolic process
betA	511145.b0311	STRING clusters	CL:6403	Mixed, incl. Choline metabolic process, and Cyanate transport system protein
betA	511145.b0311	STRING clusters	CL:6419	Choline metabolic process, and Cyanate transport system protein
betA	511145.b0311	KEGG	eco00260	Glycine, serine and threonine metabolism
betA	511145.b0311	KEGG	eco01100	Metabolic pathways
betA	511145.b0311	COMPARTMENTS	GOCC:0016020	Membrane
betA	511145.b0311	COMPARTMENTS	GOCC:0110165	Cellular anatomical entity
betA	511145.b0311	UniProt Keywords	KW-0274	FAD
betA	511145.b0311	UniProt Keywords	KW-0285	Flavoprotein
betA	511145.b0311	UniProt Keywords	KW-0346	Stress response
betA	511145.b0311	UniProt Keywords	KW-0472	Membrane
betA	511145.b0311	UniProt Keywords	KW-0520	NAD
betA	511145.b0311	UniProt Keywords	KW-0560	Oxidoreductase
betA	511145.b0311	UniProt Keywords	KW-1003	Cell membrane
betA	511145.b0311	Pfam	PF00732	GMC oxidoreductase
betA	511145.b0311	Pfam	PF05199	GMC oxidoreductase
betA	511145.b0311	InterPro	IPR000172	Glucose-methanol-choline oxidoreductase, N-terminal
betA	511145.b0311	InterPro	IPR007867	Glucose-methanol-choline oxidoreductase, C-terminal
betA	511145.b0311	InterPro	IPR011533	Oxygen-dependent choline dehydrogenase
betA	511145.b0311	InterPro	IPR012132	Glucose-methanol-choline oxidoreductase
betA	511145.b0311	InterPro	IPR036188	FAD/NAD(P)-binding domain superfamily
bioB	511145.b0775	GO Process	GO:0006082	Organic acid metabolic process
bioB	511145.b0775	GO Process	GO:0006766	Vitamin metabolic process
bioB	511145.b0775	GO Process	GO:0006767	Water-soluble vitamin metabolic process
bioB	511145.b0775	GO Process	GO:0006768	Biotin metabolic process
bioB	511145.b0775	GO Process	GO:0006790	Sulfur compound metabolic process
bioB	511145.b0775	GO Process	GO:0006807	Nitrogen compound metabolic process
bioB	511145.b0775	GO Process	GO:0008152	Metabolic process
bioB	511145.b0775	GO Process	GO:0009058	Biosynthetic process
bioB	511145.b0775	GO Process	GO:0009102	Biotin biosynthetic process
bioB	511145.b0775	GO Process	GO:0009110	Vitamin biosynthetic process
bioB	511145.b0775	GO Process	GO:0009987	Cellular process
bioB	511145.b0775	GO Process	GO:0016053	Organic acid biosynthetic process
bioB	511145.b0775	GO Process	GO:0018130	Heterocycle biosynthetic process
bioB	511145.b0775	GO Process	GO:0019752	Carboxylic acid metabolic process
bioB	511145.b0775	GO Process	GO:0032787	Monocarboxylic acid metabolic process
bioB	511145.b0775	GO Process	GO:0034641	Cellular nitrogen compound metabolic process
bioB	511145.b0775	GO Process	GO:0042364	Water-soluble vitamin biosynthetic process
bioB	511145.b0775	GO Process	GO:0043436	Oxoacid metabolic process
bioB	511145.b0775	GO Process	GO:0043603	Cellular amide metabolic process
bioB	511145.b0775	GO Process	GO:0043604	Amide biosynthetic process
bioB	511145.b0775	GO Process	GO:0044237	Cellular metabolic process
bioB	511145.b0775	GO Process	GO:0044249	Cellular biosynthetic process
bioB	511145.b0775	GO Process	GO:0044271	Cellular nitrogen compound biosynthetic process
bioB	511145.b0775	GO Process	GO:0044272	Sulfur compound biosynthetic process
bioB	511145.b0775	GO Process	GO:0044281	Small molecule metabolic process
bioB	511145.b0775	GO Process	GO:0044283	Small molecule biosynthetic process
bioB	511145.b0775	GO Process	GO:0046394	Carboxylic acid biosynthetic process
bioB	511145.b0775	GO Process	GO:0046483	Heterocycle metabolic process
bioB	511145.b0775	GO Process	GO:0071704	Organic substance metabolic process
bioB	511145.b0775	GO Process	GO:0072330	Monocarboxylic acid biosynthetic process
bioB	511145.b0775	GO Process	GO:1901360	Organic cyclic compound metabolic process
bioB	511145.b0775	GO Process	GO:1901362	Organic cyclic compound biosynthetic process
bioB	511145.b0775	GO Process	GO:1901564	Organonitrogen compound metabolic process
bioB	511145.b0775	GO Process	GO:1901566	Organonitrogen compound biosynthetic process
bioB	511145.b0775	GO Process	GO:1901576	Organic substance biosynthetic process
bioB	511145.b0775	GO Function	GO:0003824	Catalytic activity
bioB	511145.b0775	GO Function	GO:0004076	Biotin synthase activity
bioB	511145.b0775	GO Function	GO:0005488	Binding
bioB	511145.b0775	GO Function	GO:0005506	Iron ion binding
bioB	511145.b0775	GO Function	GO:0016740	Transferase activity
bioB	511145.b0775	GO Function	GO:0016782	Transferase activity, transferring sulphur-containing groups
bioB	511145.b0775	GO Function	GO:0016783	Sulfurtransferase activity
bioB	511145.b0775	GO Function	GO:0043167	Ion binding
bioB	511145.b0775	GO Function	GO:0043169	Cation binding
bioB	511145.b0775	GO Function	GO:0046872	Metal ion binding
bioB	511145.b0775	GO Function	GO:0046914	Transition metal ion binding
bioB	511145.b0775	GO Function	GO:0051536	Iron-sulfur cluster binding
bioB	511145.b0775	GO Function	GO:0051537	2 iron, 2 sulfur cluster binding
bioB	511145.b0775	GO Function	GO:0051539	4 iron, 4 sulfur cluster binding
bioB	511145.b0775	GO Function	GO:0051540	Metal cluster binding
bioB	511145.b0775	STRING clusters	CL:1869	Water-soluble vitamin biosynthetic process, and Nicotinate and nicotinamide metabolism
bioB	511145.b0775	STRING clusters	CL:1870	Water-soluble vitamin biosynthetic process, and Coenzyme A biosynthetic process
bioB	511145.b0775	STRING clusters	CL:1871	Water-soluble vitamin biosynthetic process
bioB	511145.b0775	STRING clusters	CL:1872	Water-soluble vitamin biosynthetic process
bioB	511145.b0775	STRING clusters	CL:1873	Thiamine diphosphate metabolic process, and Biotin biosynthesis
bioB	511145.b0775	STRING clusters	CL:1897	Biotin biosynthesis
bioB	511145.b0775	KEGG	eco00780	Biotin metabolism
bioB	511145.b0775	KEGG	eco01100	Metabolic pathways
bioB	511145.b0775	UniProt Keywords	KW-0001	2Fe-2S
bioB	511145.b0775	UniProt Keywords	KW-0004	4Fe-4S
bioB	511145.b0775	UniProt Keywords	KW-0093	Biotin biosynthesis
bioB	511145.b0775	UniProt Keywords	KW-0408	Iron
bioB	511145.b0775	UniProt Keywords	KW-0411	Iron-sulfur
bioB	511145.b0775	UniProt Keywords	KW-0479	Metal-binding
bioB	511145.b0775	UniProt Keywords	KW-0808	Transferase
bioB	511145.b0775	UniProt Keywords	KW-0949	S-adenosyl-L-methionine
bioB	511145.b0775	InterPro	IPR002684	Biotin synthase/Biotin biosynthesis bifunctional protein BioAB
bioB	511145.b0775	InterPro	IPR006638	Elp3/MiaA/NifB-like, radical SAM core domain
bioB	511145.b0775	InterPro	IPR007197	Radical SAM
bioB	511145.b0775	InterPro	IPR010722	Biotin and thiamin synthesis-associated domain
bioB	511145.b0775	InterPro	IPR013785	Aldolase-type TIM barrel
bioB	511145.b0775	InterPro	IPR024177	Biotin synthase
bioB	511145.b0775	SMART	SM00876	Biotin and Thiamin Synthesis associated domain
carB	511145.b0033	GO Process	GO:0006082	Organic acid metabolic process
carB	511145.b0033	GO Process	GO:0006139	Nucleobase-containing compound metabolic process
carB	511145.b0033	GO Process	GO:0006206	Pyrimidine nucleobase metabolic process
carB	511145.b0033	GO Process	GO:0006220	Pyrimidine nucleotide metabolic process
carB	511145.b0033	GO Process	GO:0006221	Pyrimidine nucleotide biosynthetic process
carB	511145.b0033	GO Process	GO:0006222	UMP biosynthetic process
carB	511145.b0033	GO Process	GO:0006520	Cellular amino acid metabolic process
carB	511145.b0033	GO Process	GO:0006525	Arginine metabolic process
carB	511145.b0033	GO Process	GO:0006526	Arginine biosynthetic process
carB	511145.b0033	GO Process	GO:0006541	Glutamine metabolic process
carB	511145.b0033	GO Process	GO:0006725	Cellular aromatic compound metabolic process
carB	511145.b0033	GO Process	GO:0006753	Nucleoside phosphate metabolic process
carB	511145.b0033	GO Process	GO:0006793	Phosphorus metabolic process
carB	511145.b0033	GO Process	GO:0006796	Phosphate-containing compound metabolic process
carB	511145.b0033	GO Process	GO:0006807	Nitrogen compound metabolic process
carB	511145.b0033	GO Process	GO:0008152	Metabolic process
carB	511145.b0033	GO Process	GO:0008652	Cellular amino acid biosynthetic process
carB	511145.b0033	GO Process	GO:0009058	Biosynthetic process
carB	511145.b0033	GO Process	GO:0009064	Glutamine family amino acid metabolic process
carB	511145.b0033	GO Process	GO:0009084	Glutamine family amino acid biosynthetic process
carB	511145.b0033	GO Process	GO:0009112	Nucleobase metabolic process
carB	511145.b0033	GO Process	GO:0009117	Nucleotide metabolic process
carB	511145.b0033	GO Process	GO:0009123	Nucleoside monophosphate metabolic process
carB	511145.b0033	GO Process	GO:0009124	Nucleoside monophosphate biosynthetic process
carB	511145.b0033	GO Process	GO:0009129	Pyrimidine nucleoside monophosphate metabolic process
carB	511145.b0033	GO Process	GO:0009130	Pyrimidine nucleoside monophosphate biosynthetic process
carB	511145.b0033	GO Process	GO:0009156	Ribonucleoside monophosphate biosynthetic process
carB	511145.b0033	GO Process	GO:0009161	Ribonucleoside monophosphate metabolic process
carB	511145.b0033	GO Process	GO:0009165	Nucleotide biosynthetic process
carB	511145.b0033	GO Process	GO:0009173	Pyrimidine ribonucleoside monophosphate metabolic process
carB	511145.b0033	GO Process	GO:0009174	Pyrimidine ribonucleoside monophosphate biosynthetic process
carB	511145.b0033	GO Process	GO:0009218	Pyrimidine ribonucleotide metabolic process
carB	511145.b0033	GO Process	GO:0009220	Pyrimidine ribonucleotide biosynthetic process
carB	511145.b0033	GO Process	GO:0009259	Ribonucleotide metabolic process
carB	511145.b0033	GO Process	GO:0009260	Ribonucleotide biosynthetic process
carB	511145.b0033	GO Process	GO:0009987	Cellular process
carB	511145.b0033	GO Process	GO:0016053	Organic acid biosynthetic process
carB	511145.b0033	GO Process	GO:0018130	Heterocycle biosynthetic process
carB	511145.b0033	GO Process	GO:0019438	Aromatic compound biosynthetic process
carB	511145.b0033	GO Process	GO:0019637	Organophosphate metabolic process
carB	511145.b0033	GO Process	GO:0019693	Ribose phosphate metabolic process
carB	511145.b0033	GO Process	GO:0019752	Carboxylic acid metabolic process
carB	511145.b0033	GO Process	GO:0019856	Pyrimidine nucleobase biosynthetic process
carB	511145.b0033	GO Process	GO:0034641	Cellular nitrogen compound metabolic process
carB	511145.b0033	GO Process	GO:0034654	Nucleobase-containing compound biosynthetic process
carB	511145.b0033	GO Process	GO:0043436	Oxoacid metabolic process
carB	511145.b0033	GO Process	GO:0044205	De novo UMP biosynthetic process
carB	511145.b0033	GO Process	GO:0044237	Cellular metabolic process
carB	511145.b0033	GO Process	GO:0044238	Primary metabolic process
carB	511145.b0033	GO Process	GO:0044249	Cellular biosynthetic process
carB	511145.b0033	GO Process	GO:0044271	Cellular nitrogen compound biosynthetic process
carB	511145.b0033	GO Process	GO:0044281	Small molecule metabolic process
carB	511145.b0033	GO Process	GO:0044283	Small molecule biosynthetic process
carB	511145.b0033	GO Process	GO:0046049	UMP metabolic process
carB	511145.b0033	GO Process	GO:0046112	Nucleobase biosynthetic process
carB	511145.b0033	GO Process	GO:0046390	Ribose phosphate biosynthetic process
carB	511145.b0033	GO Process	GO:0046394	Carboxylic acid biosynthetic process
carB	511145.b0033	GO Process	GO:0046483	Heterocycle metabolic process
carB	511145.b0033	GO Process	GO:0055086	Nucleobase-containing small molecule metabolic process
carB	511145.b0033	GO Process	GO:0071704	Organic substance metabolic process
carB	511145.b0033	GO Process	GO:0072527	Pyrimidine-containing compound metabolic process
carB	511145.b0033	GO Process	GO:0072528	Pyrimidine-containing compound biosynthetic process
carB	511145.b0033	GO Process	GO:0090407	Organophosphate biosynthetic process
carB	511145.b0033	GO Process	GO:1901135	Carbohydrate derivative metabolic process
carB	511145.b0033	GO Process	GO:1901137	Carbohydrate derivative biosynthetic process
carB	511145.b0033	GO Process	GO:1901293	Nucleoside phosphate biosynthetic process
carB	511145.b0033	GO Process	GO:1901360	Organic cyclic compound metabolic process
carB	511145.b0033	GO Process	GO:1901362	Organic cyclic compound biosynthetic process
carB	511145.b0033	GO Process	GO:1901564	Organonitrogen compound metabolic process
carB	511145.b0033	GO Process	GO:1901566	Organonitrogen compound biosynthetic process
carB	511145.b0033	GO Process	GO:1901576	Organic substance biosynthetic process
carB	511145.b0033	GO Process	GO:1901605	Alpha-amino acid metabolic process
carB	511145.b0033	GO Process	GO:1901607	Alpha-amino acid biosynthetic process
carB	511145.b0033	GO Function	GO:0000166	Nucleotide binding
carB	511145.b0033	GO Function	GO:0003824	Catalytic activity
carB	511145.b0033	GO Function	GO:0004087	Carbamoyl-phosphate synthase (ammonia) activity
carB	511145.b0033	GO Function	GO:0004088	Carbamoyl-phosphate synthase (glutamine-hydrolyzing) activity
carB	511145.b0033	GO Function	GO:0005488	Binding
carB	511145.b0033	GO Function	GO:0005524	ATP binding
carB	511145.b0033	GO Function	GO:0016597	Amino acid binding
carB	511145.b0033	GO Function	GO:0016874	Ligase activity
carB	511145.b0033	GO Function	GO:0016879	Ligase activity, forming carbon-nitrogen bonds
carB	511145.b0033	GO Function	GO:0016884	Carbon-nitrogen ligase activity, with glutamine as amido-N-donor
carB	511145.b0033	GO Function	GO:0017076	Purine nucleotide binding
carB	511145.b0033	GO Function	GO:0030554	Adenyl nucleotide binding
carB	511145.b0033	GO Function	GO:0032553	Ribonucleotide binding
carB	511145.b0033	GO Function	GO:0032555	Purine ribonucleotide binding
carB	511145.b0033	GO Function	GO:0032559	Adenyl ribonucleotide binding
carB	511145.b0033	GO Function	GO:0035639	Purine ribonucleoside triphosphate binding
carB	511145.b0033	GO Function	GO:0036094	Small molecule binding
carB	511145.b0033	GO Function	GO:0043167	Ion binding
carB	511145.b0033	GO Function	GO:0043168	Anion binding
carB	511145.b0033	GO Function	GO:0043169	Cation binding
carB	511145.b0033	GO Function	GO:0043177	Organic acid binding
carB	511145.b0033	GO Function	GO:0046872	Metal ion binding
carB	511145.b0033	GO Function	GO:0097159	Organic cyclic compound binding
carB	511145.b0033	GO Function	GO:0097367	Carbohydrate derivative binding
carB	511145.b0033	GO Function	GO:1901265	Nucleoside phosphate binding
carB	511145.b0033	GO Function	GO:1901363	Heterocyclic compound binding
carB	511145.b0033	GO Component	GO:0005622	Intracellular anatomical structure
carB	511145.b0033	GO Component	GO:0005737	Cytoplasm
carB	511145.b0033	GO Component	GO:0005829	Cytosol
carB	511145.b0033	GO Component	GO:0005951	Carbamoyl-phosphate synthase complex
carB	511145.b0033	GO Component	GO:0032991	Protein-containing complex
carB	511145.b0033	GO Component	GO:0110165	Cellular anatomical entity
carB	511145.b0033	GO Component	GO:1902494	Catalytic complex
carB	511145.b0033	STRING clusters	CL:1684	Mixed, incl. Nucleoside monophosphate biosynthetic process, and Pyrimidine metabolism
carB	511145.b0033	STRING clusters	CL:1685	Nucleoside monophosphate biosynthetic process, and Nucleobase transport
carB	511145.b0033	STRING clusters	CL:1686	Ribonucleoside monophosphate biosynthetic process, and Nucleobase transport
carB	511145.b0033	STRING clusters	CL:1687	Ribonucleoside monophosphate biosynthetic process, and Nucleobase transport
carB	511145.b0033	STRING clusters	CL:1689	Ribonucleoside monophosphate biosynthetic process
carB	511145.b0033	STRING clusters	CL:1724	De novo UMP biosynthetic process
carB	511145.b0033	KEGG	eco00240	Pyrimidine metabolism
carB	511145.b0033	KEGG	eco00250	Alanine, aspartate and glutamate metabolism
carB	511145.b0033	KEGG	eco01100	Metabolic pathways
carB	511145.b0033	COMPARTMENTS	GOCC:0005622	Intracellular
carB	511145.b0033	COMPARTMENTS	GOCC:0005737	Cytoplasm
carB	511145.b0033	COMPARTMENTS	GOCC:0005829	Cytosol
carB	511145.b0033	COMPARTMENTS	GOCC:0005951	Carbamoyl-phosphate synthase complex
carB	511145.b0033	COMPARTMENTS	GOCC:0032991	Protein-containing complex
carB	511145.b0033	COMPARTMENTS	GOCC:0110165	Cellular anatomical entity
carB	511145.b0033	COMPARTMENTS	GOCC:1902494	Catalytic complex
carB	511145.b0033	UniProt Keywords	KW-0028	Amino-acid biosynthesis
carB	511145.b0033	UniProt Keywords	KW-0055	Arginine biosynthesis
carB	511145.b0033	UniProt Keywords	KW-0067	ATP-binding
carB	511145.b0033	UniProt Keywords	KW-0436	Ligase
carB	511145.b0033	UniProt Keywords	KW-0464	Manganese
carB	511145.b0033	UniProt Keywords	KW-0479	Metal-binding
carB	511145.b0033	UniProt Keywords	KW-0547	Nucleotide-binding
carB	511145.b0033	UniProt Keywords	KW-0665	Pyrimidine biosynthesis
carB	511145.b0033	UniProt Keywords	KW-0677	Repeat
carB	511145.b0033	Pfam	PF02786	Carbamoyl-phosphate synthase L chain, ATP binding domain
carB	511145.b0033	InterPro	IPR005479	Carbamoyl-phosphate synthetase large subunit-like, ATP-binding domain
carB	511145.b0033	InterPro	IPR005480	Carbamoyl-phosphate synthetase, large subunit oligomerisation domain
carB	511145.b0033	InterPro	IPR005483	Carbamoyl-phosphate synthase large subunit, CPSase domain
carB	511145.b0033	InterPro	IPR006275	Carbamoyl-phosphate synthase, large subunit
carB	511145.b0033	InterPro	IPR011607	Methylglyoxal synthase-like domain
carB	511145.b0033	InterPro	IPR011761	ATP-grasp fold
carB	511145.b0033	InterPro	IPR016185	Pre-ATP-grasp domain superfamily
carB	511145.b0033	InterPro	IPR033937	Carbamoyl-phosphate synthase large chain, methylglyoxal synthase-like domain
carB	511145.b0033	InterPro	IPR036897	Carbamoyl-phosphate synthetase, large subunit oligomerisation domain superfamily
carB	511145.b0033	InterPro	IPR036914	Methylglyoxal synthase-like domain superfamily
carB	511145.b0033	SMART	SM00851	MGS-like domain
carB	511145.b0033	SMART	SM01096	Carbamoyl-phosphate synthetase large chain, oligomerisation domain
clpS	511145.b0881	GO Process	GO:0006508	Proteolysis
clpS	511145.b0881	GO Process	GO:0006807	Nitrogen compound metabolic process
clpS	511145.b0881	GO Process	GO:0006950	Response to stress
clpS	511145.b0881	GO Process	GO:0008152	Metabolic process
clpS	511145.b0881	GO Process	GO:0009056	Catabolic process
clpS	511145.b0881	GO Process	GO:0009057	Macromolecule catabolic process
clpS	511145.b0881	GO Process	GO:0009266	Response to temperature stimulus
clpS	511145.b0881	GO Process	GO:0009408	Response to heat
clpS	511145.b0881	GO Process	GO:0009628	Response to abiotic stimulus
clpS	511145.b0881	GO Process	GO:0019538	Protein metabolic process
clpS	511145.b0881	GO Process	GO:0030163	Protein catabolic process
clpS	511145.b0881	GO Process	GO:0043170	Macromolecule metabolic process
clpS	511145.b0881	GO Process	GO:0044238	Primary metabolic process
clpS	511145.b0881	GO Process	GO:0050896	Response to stimulus
clpS	511145.b0881	GO Process	GO:0071704	Organic substance metabolic process
clpS	511145.b0881	GO Process	GO:1901564	Organonitrogen compound metabolic process
clpS	511145.b0881	GO Process	GO:1901565	Organonitrogen compound catabolic process
clpS	511145.b0881	GO Process	GO:1901575	Organic substance catabolic process
clpS	511145.b0881	GO Function	GO:0005488	Binding
clpS	511145.b0881	GO Function	GO:0005515	Protein binding
clpS	511145.b0881	GO Function	GO:0051087	Chaperone binding
clpS	511145.b0881	STRING clusters	CL:594	Mixed, incl. Response to heat, and DnaJ domain
clpS	511145.b0881	STRING clusters	CL:596	Response to heat, and DnaJ domain
clpS	511145.b0881	STRING clusters	CL:597	Mixed, incl. Protein folding chaperone, and Cellular response to heat
clpS	511145.b0881	Pfam	PF02617	ATP-dependent Clp protease adaptor protein ClpS
clpS	511145.b0881	InterPro	IPR003769	Adaptor protein ClpS, core
clpS	511145.b0881	InterPro	IPR014719	Ribosomal protein L7/L12, C-terminal/adaptor protein ClpS-like
clpS	511145.b0881	InterPro	IPR022935	ATP-dependent Clp protease adaptor protein ClpS
cobT	511145.b1991	GO Process	GO:0006725	Cellular aromatic compound metabolic process
cobT	511145.b1991	GO Process	GO:0006766	Vitamin metabolic process
cobT	511145.b1991	GO Process	GO:0006767	Water-soluble vitamin metabolic process
cobT	511145.b1991	GO Process	GO:0006807	Nitrogen compound metabolic process
cobT	511145.b1991	GO Process	GO:0008152	Metabolic process
cobT	511145.b1991	GO Process	GO:0009058	Biosynthetic process
cobT	511145.b1991	GO Process	GO:0009110	Vitamin biosynthetic process
cobT	511145.b1991	GO Process	GO:0009235	Cobalamin metabolic process
cobT	511145.b1991	GO Process	GO:0009236	Cobalamin biosynthetic process
cobT	511145.b1991	GO Process	GO:0009987	Cellular process
cobT	511145.b1991	GO Process	GO:0018130	Heterocycle biosynthetic process
cobT	511145.b1991	GO Process	GO:0019438	Aromatic compound biosynthetic process
cobT	511145.b1991	GO Process	GO:0033013	Tetrapyrrole metabolic process
cobT	511145.b1991	GO Process	GO:0033014	Tetrapyrrole biosynthetic process
cobT	511145.b1991	GO Process	GO:0034641	Cellular nitrogen compound metabolic process
cobT	511145.b1991	GO Process	GO:0042364	Water-soluble vitamin biosynthetic process
cobT	511145.b1991	GO Process	GO:0044237	Cellular metabolic process
cobT	511145.b1991	GO Process	GO:0044249	Cellular biosynthetic process
cobT	511145.b1991	GO Process	GO:0044271	Cellular nitrogen compound biosynthetic process
cobT	511145.b1991	GO Process	GO:0044281	Small molecule metabolic process
cobT	511145.b1991	GO Process	GO:0044283	Small molecule biosynthetic process
cobT	511145.b1991	GO Process	GO:0046483	Heterocycle metabolic process
cobT	511145.b1991	GO Process	GO:0071704	Organic substance metabolic process
cobT	511145.b1991	GO Process	GO:1901360	Organic cyclic compound metabolic process
cobT	511145.b1991	GO Process	GO:1901362	Organic cyclic compound biosynthetic process
cobT	511145.b1991	GO Process	GO:1901564	Organonitrogen compound metabolic process
cobT	511145.b1991	GO Process	GO:1901566	Organonitrogen compound biosynthetic process
cobT	511145.b1991	GO Process	GO:1901576	Organic substance biosynthetic process
cobT	511145.b1991	GO Function	GO:0003824	Catalytic activity
cobT	511145.b1991	GO Function	GO:0008939	Nicotinate-nucleotide-dimethylbenzimidazole phosphoribosyltransferase activity
cobT	511145.b1991	GO Function	GO:0016740	Transferase activity
cobT	511145.b1991	GO Function	GO:0016757	Glycosyltransferase activity
cobT	511145.b1991	GO Function	GO:0016763	Pentosyltransferase activity
cobT	511145.b1991	STRING clusters	CL:2534	Mixed, incl. Quinone metabolic process, and Tetrapyrrole biosynthetic process
cobT	511145.b1991	STRING clusters	CL:2535	Ubiquinone metabolic process, and Tetrapyrrole biosynthetic process
cobT	511145.b1991	STRING clusters	CL:2601	Porphyrin biosynthesis, and Cobalamin biosynthesis
cobT	511145.b1991	STRING clusters	CL:2625	Cobalamin biosynthesis, and Peptidase S49
cobT	511145.b1991	KEGG	eco00860	Porphyrin and chlorophyll metabolism
cobT	511145.b1991	KEGG	eco01100	Metabolic pathways
cobT	511145.b1991	UniProt Keywords	KW-0169	Cobalamin biosynthesis
cobT	511145.b1991	UniProt Keywords	KW-0328	Glycosyltransferase
cobT	511145.b1991	UniProt Keywords	KW-0808	Transferase
cobT	511145.b1991	Pfam	PF02277	Phosphoribosyltransferase
cobT	511145.b1991	InterPro	IPR003200	Nicotinate-nucleotide-dimethylbenzimidazole phosphoribosyltransferase
cobT	511145.b1991	InterPro	IPR017846	Nicotinate-nucleotide-dimethylbenzimidazole phosphoribosyltransferase, bacterial type
cobT	511145.b1991	InterPro	IPR023195	Nicotinate-nucleotide-dimethylbenzimidazole phosphoribosyltransferase, N-terminal
cobT	511145.b1991	InterPro	IPR036087	Nicotinate-nucleotide-dimethylbenzimidazole phosphoribosyltransferase-like superfamily
cohE	511145.b1145	GO Function	GO:0003676	Nucleic acid binding
cohE	511145.b1145	GO Function	GO:0003677	DNA binding
cohE	511145.b1145	GO Function	GO:0005488	Binding
cohE	511145.b1145	GO Function	GO:0097159	Organic cyclic compound binding
cohE	511145.b1145	GO Function	GO:1901363	Heterocyclic compound binding
cohE	511145.b1145	STRING clusters	CL:7781	Mixed, incl. Toxin-antitoxin system, and Viral process
cohE	511145.b1145	STRING clusters	CL:7782	Mixed, incl. Toxin-antitoxin system, and Viral process
cohE	511145.b1145	STRING clusters	CL:7916	Mixed, incl. Viral process, and Bacteriophage T4, Gp38, tail fibre assembly
cohE	511145.b1145	STRING clusters	CL:7917	Mostly uncharacterized, incl. Viral process, and Bacteriophage T4, Gp38, tail fibre assembly
cohE	511145.b1145	STRING clusters	CL:7978	Mostly uncharacterized, incl. Bacteriophage T4, Gp38, tail fibre assembly, and Bacterial antitoxin YdaS
cohE	511145.b1145	STRING clusters	CL:8000	Mixed, incl. LexA-like, and Regulation of translational elongation
cohE	511145.b1145	UniProt Keywords	KW-0238	DNA-binding
cohE	511145.b1145	UniProt Keywords	KW-0678	Repressor
cohE	511145.b1145	UniProt Keywords	KW-0804	Transcription
cohE	511145.b1145	UniProt Keywords	KW-0805	Transcription regulation
cohE	511145.b1145	Pfam	PF00717	Peptidase S24-like
cohE	511145.b1145	InterPro	IPR015927	Peptidase S24/S26A/S26B/S26C
cohE	511145.b1145	InterPro	IPR036286	LexA/Signal peptidase-like superfamily
cohE	511145.b1145	InterPro	IPR039418	LexA-like
cutC	511145.b1874	GO Function	GO:0005488	Binding
cutC	511145.b1874	GO Function	GO:0005507	Copper ion binding
cutC	511145.b1874	GO Function	GO:0043167	Ion binding
cutC	511145.b1874	GO Function	GO:0043169	Cation binding
cutC	511145.b1874	GO Function	GO:0046872	Metal ion binding
cutC	511145.b1874	GO Function	GO:0046914	Transition metal ion binding
cutC	511145.b1874	GO Component	GO:0005622	Intracellular anatomical structure
cutC	511145.b1874	GO Component	GO:0005737	Cytoplasm
cutC	511145.b1874	GO Component	GO:0110165	Cellular anatomical entity
cutC	511145.b1874	STRING clusters	CL:3636	Mostly uncharacterized, incl. Intermembrane lipid transfer, and Glucan biosynthetic process
cutC	511145.b1874	STRING clusters	CL:3637	Mostly uncharacterized, incl. Glucan biosynthetic process, and Fusaric acid resistance protein-like
cutC	511145.b1874	STRING clusters	CL:3638	Mostly uncharacterized, incl. Glucan biosynthetic process, and AsmA
cutC	511145.b1874	STRING clusters	CL:3732	Mixed, incl. Succinate transport, and Aminopeptidase activity
cutC	511145.b1874	STRING clusters	CL:3733	Mixed, incl. Succinate transport, and YidE/YbjL duplication
cutC	511145.b1874	STRING clusters	CL:3734	Mostly uncharacterized, incl. Protein of unknown function DUF1097, and Tat proofreading chaperone DmsD-type
cutC	511145.b1874	STRING clusters	CL:3735	Mixed, incl. Endonuclease/exonuclease/phosphatase, and Glyoxalase/Bleomycin resistance protein/Dihydroxybiphenyl dioxygenase
cutC	511145.b1874	UniProt Keywords	KW-0186	Copper
cutC	511145.b1874	UniProt Keywords	KW-0963	Cytoplasm
cutC	511145.b1874	Pfam	PF03932	CutC family
cutC	511145.b1874	InterPro	IPR005627	Copper homeostasis protein CutC
cutC	511145.b1874	InterPro	IPR036822	Copper homeostasis (CutC) domain superfamily
cysN	511145.b2751	GO Process	GO:0000103	Sulfate assimilation
cysN	511145.b2751	GO Process	GO:0006790	Sulfur compound metabolic process
cysN	511145.b2751	GO Process	GO:0008152	Metabolic process
cysN	511145.b2751	GO Process	GO:0009058	Biosynthetic process
cysN	511145.b2751	GO Process	GO:0009987	Cellular process
cysN	511145.b2751	GO Process	GO:0019419	Sulfate reduction
cysN	511145.b2751	GO Process	GO:0044237	Cellular metabolic process
cysN	511145.b2751	GO Process	GO:0044249	Cellular biosynthetic process
cysN	511145.b2751	GO Process	GO:0044272	Sulfur compound biosynthetic process
cysN	511145.b2751	GO Process	GO:0070813	Hydrogen sulfide metabolic process
cysN	511145.b2751	GO Process	GO:0070814	Hydrogen sulfide biosynthetic process
cysN	511145.b2751	GO Function	GO:0000166	Nucleotide binding
cysN	511145.b2751	GO Function	GO:0003824	Catalytic activity
cysN	511145.b2751	GO Function	GO:0003924	GTPase activity
cysN	511145.b2751	GO Function	GO:0004779	Sulfate adenylyltransferase activity
cysN	511145.b2751	GO Function	GO:0004781	Sulfate adenylyltransferase (ATP) activity
cysN	511145.b2751	GO Function	GO:0005488	Binding
cysN	511145.b2751	GO Function	GO:0005524	ATP binding
cysN	511145.b2751	GO Function	GO:0005525	GTP binding
cysN	511145.b2751	GO Function	GO:0016462	Pyrophosphatase activity
cysN	511145.b2751	GO Function	GO:0016740	Transferase activity
cysN	511145.b2751	GO Function	GO:0016772	Transferase activity, transferring phosphorus-containing groups
cysN	511145.b2751	GO Function	GO:0016779	Nucleotidyltransferase activity
cysN	511145.b2751	GO Function	GO:0016787	Hydrolase activity
cysN	511145.b2751	GO Function	GO:0016817	Hydrolase activity, acting on acid anhydrides
cysN	511145.b2751	GO Function	GO:0016818	Hydrolase activity, acting on acid anhydrides, in phosphorus-containing anhydrides
cysN	511145.b2751	GO Function	GO:0017076	Purine nucleotide binding
cysN	511145.b2751	GO Function	GO:0017111	Nucleoside-triphosphatase activity
cysN	511145.b2751	GO Function	GO:0019001	Guanyl nucleotide binding
cysN	511145.b2751	GO Function	GO:0030554	Adenyl nucleotide binding
cysN	511145.b2751	GO Function	GO:0032553	Ribonucleotide binding
cysN	511145.b2751	GO Function	GO:0032555	Purine ribonucleotide binding
cysN	511145.b2751	GO Function	GO:0032559	Adenyl ribonucleotide binding
cysN	511145.b2751	GO Function	GO:0032561	Guanyl ribonucleotide binding
cysN	511145.b2751	GO Function	GO:0035639	Purine ribonucleoside triphosphate binding
cysN	511145.b2751	GO Function	GO:0036094	Small molecule binding
cysN	511145.b2751	GO Function	GO:0043167	Ion binding
cysN	511145.b2751	GO Function	GO:0043168	Anion binding
cysN	511145.b2751	GO Function	GO:0070566	Adenylyltransferase activity
cysN	511145.b2751	GO Function	GO:0097159	Organic cyclic compound binding
cysN	511145.b2751	GO Function	GO:0097367	Carbohydrate derivative binding
cysN	511145.b2751	GO Function	GO:1901265	Nucleoside phosphate binding
cysN	511145.b2751	GO Function	GO:1901363	Heterocyclic compound binding
cysN	511145.b2751	GO Component	GO:0005622	Intracellular anatomical structure
cysN	511145.b2751	GO Component	GO:0005737	Cytoplasm
cysN	511145.b2751	GO Component	GO:0009336	Sulfate adenylyltransferase complex (ATP)
cysN	511145.b2751	GO Component	GO:0032991	Protein-containing complex
cysN	511145.b2751	GO Component	GO:0061695	Transferase complex, transferring phosphorus-containing groups
cysN	511145.b2751	GO Component	GO:0110165	Cellular anatomical entity
cysN	511145.b2751	GO Component	GO:1902494	Catalytic complex
cysN	511145.b2751	GO Component	GO:1902503	Adenylyltransferase complex
cysN	511145.b2751	GO Component	GO:1990234	Transferase complex
cysN	511145.b2751	STRING clusters	CL:4542	Sulfur metabolism
cysN	511145.b2751	STRING clusters	CL:4546	Thiosulfate transport, and Hydrogen sulfide metabolic process
cysN	511145.b2751	STRING clusters	CL:4556	Hydrogen sulfide metabolic process
cysN	511145.b2751	KEGG	eco00230	Purine metabolism
cysN	511145.b2751	KEGG	eco00261	Monobactam biosynthesis
cysN	511145.b2751	KEGG	eco00450	Selenocompound metabolism
cysN	511145.b2751	KEGG	eco00920	Sulfur metabolism
cysN	511145.b2751	KEGG	eco01100	Metabolic pathways
cysN	511145.b2751	KEGG	eco01110	Biosynthesis of secondary metabolites
cysN	511145.b2751	KEGG	eco01120	Microbial metabolism in diverse environments
cysN	511145.b2751	COMPARTMENTS	GOCC:0005622	Intracellular
cysN	511145.b2751	COMPARTMENTS	GOCC:0005737	Cytoplasm
cysN	511145.b2751	COMPARTMENTS	GOCC:0009336	Sulfate adenylyltransferase complex (ATP)
cysN	511145.b2751	COMPARTMENTS	GOCC:0032991	Protein-containing complex
cysN	511145.b2751	COMPARTMENTS	GOCC:0061695	Transferase complex, transferring phosphorus-containing groups
cysN	511145.b2751	COMPARTMENTS	GOCC:0110165	Cellular anatomical entity
cysN	511145.b2751	COMPARTMENTS	GOCC:1902494	Catalytic complex
cysN	511145.b2751	COMPARTMENTS	GOCC:1902503	Adenylyltransferase complex
cysN	511145.b2751	COMPARTMENTS	GOCC:1990234	Transferase complex
cysN	511145.b2751	UniProt Keywords	KW-0067	ATP-binding
cysN	511145.b2751	UniProt Keywords	KW-0342	GTP-binding
cysN	511145.b2751	UniProt Keywords	KW-0547	Nucleotide-binding
cysN	511145.b2751	UniProt Keywords	KW-0548	Nucleotidyltransferase
cysN	511145.b2751	UniProt Keywords	KW-0808	Transferase
cysN	511145.b2751	Pfam	PF01926	50S ribosome-binding GTPase
cysN	511145.b2751	InterPro	IPR000795	Translational (tr)-type GTP-binding domain
cysN	511145.b2751	InterPro	IPR005225	Small GTP-binding protein domain
cysN	511145.b2751	InterPro	IPR009000	Translation protein, beta-barrel domain superfamily
cysN	511145.b2751	InterPro	IPR009001	Translation elongation factor EF1A/initiation factor IF2gamma, C-terminal
cysN	511145.b2751	InterPro	IPR011779	Sulphate adenylyltransferase, large subunit
cysN	511145.b2751	InterPro	IPR027417	P-loop containing nucleoside triphosphate hydrolase
cysN	511145.b2751	InterPro	IPR031157	Tr-type G domain, conserved site
cysN	511145.b2751	InterPro	IPR041757	Sulfate adenylyltransferase subunit CysN, GTP-binding domain
cysN	511145.b2751	InterPro	IPR044138	Sulfate adenylyltransferase subunit CysN, Domain II
cysN	511145.b2751	InterPro	IPR044139	Sulfate adenylyltransferase subunit CysN, Domain III
dadA	511145.b1189	GO Process	GO:0006082	Organic acid metabolic process
dadA	511145.b1189	GO Process	GO:0006520	Cellular amino acid metabolic process
dadA	511145.b1189	GO Process	GO:0006522	Alanine metabolic process
dadA	511145.b1189	GO Process	GO:0006524	Alanine catabolic process
dadA	511145.b1189	GO Process	GO:0006807	Nitrogen compound metabolic process
dadA	511145.b1189	GO Process	GO:0008152	Metabolic process
dadA	511145.b1189	GO Process	GO:0009056	Catabolic process
dadA	511145.b1189	GO Process	GO:0009063	Cellular amino acid catabolic process
dadA	511145.b1189	GO Process	GO:0009078	Pyruvate family amino acid metabolic process
dadA	511145.b1189	GO Process	GO:0009080	Pyruvate family amino acid catabolic process
dadA	511145.b1189	GO Process	GO:0009987	Cellular process
dadA	511145.b1189	GO Process	GO:0016054	Organic acid catabolic process
dadA	511145.b1189	GO Process	GO:0019478	D-amino acid catabolic process
dadA	511145.b1189	GO Process	GO:0019480	L-alanine oxidation to pyruvate via D-alanine
dadA	511145.b1189	GO Process	GO:0019752	Carboxylic acid metabolic process
dadA	511145.b1189	GO Process	GO:0042851	L-alanine metabolic process
dadA	511145.b1189	GO Process	GO:0042853	L-alanine catabolic process
dadA	511145.b1189	GO Process	GO:0043436	Oxoacid metabolic process
dadA	511145.b1189	GO Process	GO:0044237	Cellular metabolic process
dadA	511145.b1189	GO Process	GO:0044238	Primary metabolic process
dadA	511145.b1189	GO Process	GO:0044248	Cellular catabolic process
dadA	511145.b1189	GO Process	GO:0044281	Small molecule metabolic process
dadA	511145.b1189	GO Process	GO:0044282	Small molecule catabolic process
dadA	511145.b1189	GO Process	GO:0046144	D-alanine family amino acid metabolic process
dadA	511145.b1189	GO Process	GO:0046395	Carboxylic acid catabolic process
dadA	511145.b1189	GO Process	GO:0046416	D-amino acid metabolic process
dadA	511145.b1189	GO Process	GO:0046436	D-alanine metabolic process
dadA	511145.b1189	GO Process	GO:0055130	D-alanine catabolic process
dadA	511145.b1189	GO Process	GO:0071704	Organic substance metabolic process
dadA	511145.b1189	GO Process	GO:1901564	Organonitrogen compound metabolic process
dadA	511145.b1189	GO Process	GO:1901565	Organonitrogen compound catabolic process
dadA	511145.b1189	GO Process	GO:1901575	Organic substance catabolic process
dadA	511145.b1189	GO Process	GO:1901605	Alpha-amino acid metabolic process
dadA	511145.b1189	GO Process	GO:1901606	Alpha-amino acid catabolic process
dadA	511145.b1189	GO Function	GO:0003824	Catalytic activity
dadA	511145.b1189	GO Function	GO:0008718	D-amino-acid dehydrogenase activity
dadA	511145.b1189	GO Function	GO:0016491	Oxidoreductase activity
dadA	511145.b1189	GO Function	GO:0016638	Oxidoreductase activity, acting on the CH-NH2 group of donors
dadA	511145.b1189	GO Component	GO:0005622	Intracellular anatomical structure
dadA	511145.b1189	GO Component	GO:0005737	Cytoplasm
dadA	511145.b1189	GO Component	GO:0005886	Plasma membrane
dadA	511145.b1189	GO Component	GO:0016020	Membrane
dadA	511145.b1189	GO Component	GO:0071944	Cell periphery
dadA	511145.b1189	GO Component	GO:0110165	Cellular anatomical entity
dadA	511145.b1189	STRING clusters	CL:1426	Amino-acid biosynthesis, and Arginine biosynthesis
dadA	511145.b1189	STRING clusters	CL:1427	Amino-acid biosynthesis, and D-alanine metabolic process
dadA	511145.b1189	STRING clusters	CL:1591	L-alanine metabolic process
dadA	511145.b1189	KEGG	eco00360	Phenylalanine metabolism
dadA	511145.b1189	KEGG	eco01100	Metabolic pathways
dadA	511145.b1189	COMPARTMENTS	GOCC:0005886	Plasma membrane
dadA	511145.b1189	COMPARTMENTS	GOCC:0016020	Membrane
dadA	511145.b1189	COMPARTMENTS	GOCC:0071944	Cell periphery
dadA	511145.b1189	COMPARTMENTS	GOCC:0110165	Cellular anatomical entity
dadA	511145.b1189	UniProt Keywords	KW-0274	FAD
dadA	511145.b1189	UniProt Keywords	KW-0285	Flavoprotein
dadA	511145.b1189	UniProt Keywords	KW-0472	Membrane
dadA	511145.b1189	UniProt Keywords	KW-0560	Oxidoreductase
dadA	511145.b1189	UniProt Keywords	KW-0997	Cell inner membrane
dadA	511145.b1189	UniProt Keywords	KW-1003	Cell membrane
dadA	511145.b1189	Pfam	PF00070	Pyridine nucleotide-disulphide oxidoreductase
dadA	511145.b1189	InterPro	IPR006076	FAD dependent oxidoreductase
dadA	511145.b1189	InterPro	IPR023080	D-amino acid dehydrogenase DadA
dadA	511145.b1189	InterPro	IPR036188	FAD/NAD(P)-binding domain superfamily
dadX	511145.b1190	GO Process	GO:0006082	Organic acid metabolic process
dadX	511145.b1190	GO Process	GO:0006520	Cellular amino acid metabolic process
dadX	511145.b1190	GO Process	GO:0006522	Alanine metabolic process
dadX	511145.b1190	GO Process	GO:0006523	Alanine biosynthetic process
dadX	511145.b1190	GO Process	GO:0006524	Alanine catabolic process
dadX	511145.b1190	GO Process	GO:0006807	Nitrogen compound metabolic process
dadX	511145.b1190	GO Process	GO:0008152	Metabolic process
dadX	511145.b1190	GO Process	GO:0008652	Cellular amino acid biosynthetic process
dadX	511145.b1190	GO Process	GO:0009056	Catabolic process
dadX	511145.b1190	GO Process	GO:0009058	Biosynthetic process
dadX	511145.b1190	GO Process	GO:0009063	Cellular amino acid catabolic process
dadX	511145.b1190	GO Process	GO:0009078	Pyruvate family amino acid metabolic process
dadX	511145.b1190	GO Process	GO:0009079	Pyruvate family amino acid biosynthetic process
dadX	511145.b1190	GO Process	GO:0009080	Pyruvate family amino acid catabolic process
dadX	511145.b1190	GO Process	GO:0009987	Cellular process
dadX	511145.b1190	GO Process	GO:0016053	Organic acid biosynthetic process
dadX	511145.b1190	GO Process	GO:0016054	Organic acid catabolic process
dadX	511145.b1190	GO Process	GO:0019480	L-alanine oxidation to pyruvate via D-alanine
dadX	511145.b1190	GO Process	GO:0019752	Carboxylic acid metabolic process
dadX	511145.b1190	GO Process	GO:0030632	D-alanine biosynthetic process
dadX	511145.b1190	GO Process	GO:0042851	L-alanine metabolic process
dadX	511145.b1190	GO Process	GO:0042853	L-alanine catabolic process
dadX	511145.b1190	GO Process	GO:0043436	Oxoacid metabolic process
dadX	511145.b1190	GO Process	GO:0044237	Cellular metabolic process
dadX	511145.b1190	GO Process	GO:0044238	Primary metabolic process
dadX	511145.b1190	GO Process	GO:0044248	Cellular catabolic process
dadX	511145.b1190	GO Process	GO:0044249	Cellular biosynthetic process
dadX	511145.b1190	GO Process	GO:0044281	Small molecule metabolic process
dadX	511145.b1190	GO Process	GO:0044282	Small molecule catabolic process
dadX	511145.b1190	GO Process	GO:0044283	Small molecule biosynthetic process
dadX	511145.b1190	GO Process	GO:0046144	D-alanine family amino acid metabolic process
dadX	511145.b1190	GO Process	GO:0046145	D-alanine family amino acid biosynthetic process
dadX	511145.b1190	GO Process	GO:0046394	Carboxylic acid biosynthetic process
dadX	511145.b1190	GO Process	GO:0046395	Carboxylic acid catabolic process
dadX	511145.b1190	GO Process	GO:0046416	D-amino acid metabolic process
dadX	511145.b1190	GO Process	GO:0046436	D-alanine metabolic process
dadX	511145.b1190	GO Process	GO:0046437	D-amino acid biosynthetic process
dadX	511145.b1190	GO Process	GO:0071704	Organic substance metabolic process
dadX	511145.b1190	GO Process	GO:1901564	Organonitrogen compound metabolic process
dadX	511145.b1190	GO Process	GO:1901565	Organonitrogen compound catabolic process
dadX	511145.b1190	GO Process	GO:1901566	Organonitrogen compound biosynthetic process
dadX	511145.b1190	GO Process	GO:1901575	Organic substance catabolic process
dadX	511145.b1190	GO Process	GO:1901576	Organic substance biosynthetic process
dadX	511145.b1190	GO Process	GO:1901605	Alpha-amino acid metabolic process
dadX	511145.b1190	GO Process	GO:1901606	Alpha-amino acid catabolic process
dadX	511145.b1190	GO Process	GO:1901607	Alpha-amino acid biosynthetic process
dadX	511145.b1190	GO Function	GO:0003824	Catalytic activity
dadX	511145.b1190	GO Function	GO:0005488	Binding
dadX	511145.b1190	GO Function	GO:0008784	Alanine racemase activity
dadX	511145.b1190	GO Function	GO:0016853	Isomerase activity
dadX	511145.b1190	GO Function	GO:0016854	Racemase and epimerase activity
dadX	511145.b1190	GO Function	GO:0016855	Racemase and epimerase activity, acting on amino acids and derivatives
dadX	511145.b1190	GO Function	GO:0019842	Vitamin binding
dadX	511145.b1190	GO Function	GO:0030170	Pyridoxal phosphate binding
dadX	511145.b1190	GO Function	GO:0036094	Small molecule binding
dadX	511145.b1190	GO Function	GO:0036361	Racemase activity, acting on amino acids and derivatives
dadX	511145.b1190	GO Function	GO:0043167	Ion binding
dadX	511145.b1190	GO Function	GO:0043168	Anion binding
dadX	511145.b1190	GO Function	GO:0047661	Amino-acid racemase activity
dadX	511145.b1190	GO Function	GO:0070279	Vitamin B6 binding
dadX	511145.b1190	GO Function	GO:0097159	Organic cyclic compound binding
dadX	511145.b1190	GO Function	GO:1901363	Heterocyclic compound binding
dadX	511145.b1190	GO Component	GO:0005622	Intracellular anatomical structure
dadX	511145.b1190	GO Component	GO:0005737	Cytoplasm
dadX	511145.b1190	GO Component	GO:0005829	Cytosol
dadX	511145.b1190	GO Component	GO:0110165	Cellular anatomical entity
dadX	511145.b1190	STRING clusters	CL:1426	Amino-acid biosynthesis, and Arginine biosynthesis
dadX	511145.b1190	STRING clusters	CL:1427	Amino-acid biosynthesis, and D-alanine metabolic process
dadX	511145.b1190	STRING clusters	CL:1591	L-alanine metabolic process
dadX	511145.b1190	KEGG	eco00473	D-Alanine metabolism
dadX	511145.b1190	KEGG	eco01100	Metabolic pathways
dadX	511145.b1190	KEGG	eco01502	Vancomycin resistance
dadX	511145.b1190	COMPARTMENTS	GOCC:0005622	Intracellular
dadX	511145.b1190	COMPARTMENTS	GOCC:0005737	Cytoplasm
dadX	511145.b1190	COMPARTMENTS	GOCC:0005829	Cytosol
dadX	511145.b1190	COMPARTMENTS	GOCC:0110165	Cellular anatomical entity
dadX	511145.b1190	UniProt Keywords	KW-0413	Isomerase
dadX	511145.b1190	UniProt Keywords	KW-0663	Pyridoxal phosphate
dadX	511145.b1190	Pfam	PF01168	Alanine racemase, N-terminal domain
dadX	511145.b1190	InterPro	IPR000821	Alanine racemase
dadX	511145.b1190	InterPro	IPR001608	Alanine racemase, N-terminal
dadX	511145.b1190	InterPro	IPR009006	Alanine racemase/group IV decarboxylase, C-terminal
dadX	511145.b1190	InterPro	IPR011079	Alanine racemase, C-terminal
dadX	511145.b1190	InterPro	IPR020622	Alanine racemase, pyridoxal-phosphate attachment site
dadX	511145.b1190	InterPro	IPR029066	PLP-binding barrel
dadX	511145.b1190	SMART	SM01005	Alanine racemase, C-terminal domain
def	511145.b3287	GO Process	GO:0006412	Translation
def	511145.b3287	GO Process	GO:0006518	Peptide metabolic process
def	511145.b3287	GO Process	GO:0006807	Nitrogen compound metabolic process
def	511145.b3287	GO Process	GO:0008152	Metabolic process
def	511145.b3287	GO Process	GO:0009058	Biosynthetic process
def	511145.b3287	GO Process	GO:0009059	Macromolecule biosynthetic process
def	511145.b3287	GO Process	GO:0009987	Cellular process
def	511145.b3287	GO Process	GO:0010467	Gene expression
def	511145.b3287	GO Process	GO:0018193	Peptidyl-amino acid modification
def	511145.b3287	GO Process	GO:0018206	Peptidyl-methionine modification
def	511145.b3287	GO Process	GO:0019538	Protein metabolic process
def	511145.b3287	GO Process	GO:0031365	N-terminal protein amino acid modification
def	511145.b3287	GO Process	GO:0034641	Cellular nitrogen compound metabolic process
def	511145.b3287	GO Process	GO:0034645	Cellular macromolecule biosynthetic process
def	511145.b3287	GO Process	GO:0036211	Protein modification process
def	511145.b3287	GO Process	GO:0043043	Peptide biosynthetic process
def	511145.b3287	GO Process	GO:0043170	Macromolecule metabolic process
def	511145.b3287	GO Process	GO:0043412	Macromolecule modification
def	511145.b3287	GO Process	GO:0043603	Cellular amide metabolic process
def	511145.b3287	GO Process	GO:0043604	Amide biosynthetic process
def	511145.b3287	GO Process	GO:0043686	Co-translational protein modification
def	511145.b3287	GO Process	GO:0044237	Cellular metabolic process
def	511145.b3287	GO Process	GO:0044238	Primary metabolic process
def	511145.b3287	GO Process	GO:0044249	Cellular biosynthetic process
def	511145.b3287	GO Process	GO:0044260	Cellular macromolecule metabolic process
def	511145.b3287	GO Process	GO:0044271	Cellular nitrogen compound biosynthetic process
def	511145.b3287	GO Process	GO:0071704	Organic substance metabolic process
def	511145.b3287	GO Process	GO:1901564	Organonitrogen compound metabolic process
def	511145.b3287	GO Process	GO:1901566	Organonitrogen compound biosynthetic process
def	511145.b3287	GO Process	GO:1901576	Organic substance biosynthetic process
def	511145.b3287	GO Function	GO:0003824	Catalytic activity
def	511145.b3287	GO Function	GO:0005488	Binding
def	511145.b3287	GO Function	GO:0005506	Iron ion binding
def	511145.b3287	GO Function	GO:0008198	Ferrous iron binding
def	511145.b3287	GO Function	GO:0008270	Zinc ion binding
def	511145.b3287	GO Function	GO:0016787	Hydrolase activity
def	511145.b3287	GO Function	GO:0016810	Hydrolase activity, acting on carbon-nitrogen (but not peptide) bonds
def	511145.b3287	GO Function	GO:0016811	Hydrolase activity, acting on carbon-nitrogen (but not peptide) bonds, in linear amides
def	511145.b3287	GO Function	GO:0042586	Peptide deformylase activity
def	511145.b3287	GO Function	GO:0043021	Ribonucleoprotein complex binding
def	511145.b3287	GO Function	GO:0043022	Ribosome binding
def	511145.b3287	GO Function	GO:0043167	Ion binding
def	511145.b3287	GO Function	GO:0043169	Cation binding
def	511145.b3287	GO Function	GO:0044877	Protein-containing complex binding
def	511145.b3287	GO Function	GO:0046872	Metal ion binding
def	511145.b3287	GO Function	GO:0046914	Transition metal ion binding
def	511145.b3287	GO Component	GO:0005622	Intracellular anatomical structure
def	511145.b3287	GO Component	GO:0005737	Cytoplasm
def	511145.b3287	GO Component	GO:0005829	Cytosol
def	511145.b3287	GO Component	GO:0110165	Cellular anatomical entity
def	511145.b3287	STRING clusters	CL:481	Mixed, incl. tRNA threonylcarbamoyladenosine modification, and Horizontal gene transfer
def	511145.b3287	STRING clusters	CL:482	Mixed, incl. Horizontal gene transfer, and C-methyltransferase activity
def	511145.b3287	STRING clusters	CL:483	Mixed, incl. Horizontal gene transfer, and C-methyltransferase activity
def	511145.b3287	STRING clusters	CL:484	Mixed, incl. C-methyltransferase activity, and Formyl transferase, C-terminal
def	511145.b3287	COMPARTMENTS	GOCC:0005622	Intracellular
def	511145.b3287	COMPARTMENTS	GOCC:0005737	Cytoplasm
def	511145.b3287	COMPARTMENTS	GOCC:0005829	Cytosol
def	511145.b3287	COMPARTMENTS	GOCC:0110165	Cellular anatomical entity
def	511145.b3287	UniProt Keywords	KW-0378	Hydrolase
def	511145.b3287	UniProt Keywords	KW-0408	Iron
def	511145.b3287	UniProt Keywords	KW-0479	Metal-binding
def	511145.b3287	UniProt Keywords	KW-0648	Protein biosynthesis
def	511145.b3287	UniProt Keywords	KW-0862	Zinc
def	511145.b3287	Pfam	PF01327	Polypeptide deformylase
def	511145.b3287	InterPro	IPR023635	Peptide deformylase
def	511145.b3287	InterPro	IPR036821	Peptide deformylase superfamily
dgt	511145.b0160	GO Process	GO:0006139	Nucleobase-containing compound metabolic process
dgt	511145.b0160	GO Process	GO:0006163	Purine nucleotide metabolic process
dgt	511145.b0160	GO Process	GO:0006195	Purine nucleotide catabolic process
dgt	511145.b0160	GO Process	GO:0006203	dGTP catabolic process
dgt	511145.b0160	GO Process	GO:0006725	Cellular aromatic compound metabolic process
dgt	511145.b0160	GO Process	GO:0006753	Nucleoside phosphate metabolic process
dgt	511145.b0160	GO Process	GO:0006793	Phosphorus metabolic process
dgt	511145.b0160	GO Process	GO:0006796	Phosphate-containing compound metabolic process
dgt	511145.b0160	GO Process	GO:0006807	Nitrogen compound metabolic process
dgt	511145.b0160	GO Process	GO:0008152	Metabolic process
dgt	511145.b0160	GO Process	GO:0009056	Catabolic process
dgt	511145.b0160	GO Process	GO:0009117	Nucleotide metabolic process
dgt	511145.b0160	GO Process	GO:0009141	Nucleoside triphosphate metabolic process
dgt	511145.b0160	GO Process	GO:0009143	Nucleoside triphosphate catabolic process
dgt	511145.b0160	GO Process	GO:0009144	Purine nucleoside triphosphate metabolic process
dgt	511145.b0160	GO Process	GO:0009146	Purine nucleoside triphosphate catabolic process
dgt	511145.b0160	GO Process	GO:0009151	Purine deoxyribonucleotide metabolic process
dgt	511145.b0160	GO Process	GO:0009155	Purine deoxyribonucleotide catabolic process
dgt	511145.b0160	GO Process	GO:0009166	Nucleotide catabolic process
dgt	511145.b0160	GO Process	GO:0009200	Deoxyribonucleoside triphosphate metabolic process
dgt	511145.b0160	GO Process	GO:0009204	Deoxyribonucleoside triphosphate catabolic process
dgt	511145.b0160	GO Process	GO:0009215	Purine deoxyribonucleoside triphosphate metabolic process
dgt	511145.b0160	GO Process	GO:0009217	Purine deoxyribonucleoside triphosphate catabolic process
dgt	511145.b0160	GO Process	GO:0009262	Deoxyribonucleotide metabolic process
dgt	511145.b0160	GO Process	GO:0009264	Deoxyribonucleotide catabolic process
dgt	511145.b0160	GO Process	GO:0009394	2-deoxyribonucleotide metabolic process
dgt	511145.b0160	GO Process	GO:0009987	Cellular process
dgt	511145.b0160	GO Process	GO:0015949	Nucleobase-containing small molecule interconversion
dgt	511145.b0160	GO Process	GO:0019439	Aromatic compound catabolic process
dgt	511145.b0160	GO Process	GO:0019637	Organophosphate metabolic process
dgt	511145.b0160	GO Process	GO:0019692	Deoxyribose phosphate metabolic process
dgt	511145.b0160	GO Process	GO:0034641	Cellular nitrogen compound metabolic process
dgt	511145.b0160	GO Process	GO:0034655	Nucleobase-containing compound catabolic process
dgt	511145.b0160	GO Process	GO:0044237	Cellular metabolic process
dgt	511145.b0160	GO Process	GO:0044238	Primary metabolic process
dgt	511145.b0160	GO Process	GO:0044248	Cellular catabolic process
dgt	511145.b0160	GO Process	GO:0044270	Cellular nitrogen compound catabolic process
dgt	511145.b0160	GO Process	GO:0044281	Small molecule metabolic process
dgt	511145.b0160	GO Process	GO:0046070	dGTP metabolic process
dgt	511145.b0160	GO Process	GO:0046386	Deoxyribose phosphate catabolic process
dgt	511145.b0160	GO Process	GO:0046434	Organophosphate catabolic process
dgt	511145.b0160	GO Process	GO:0046483	Heterocycle metabolic process
dgt	511145.b0160	GO Process	GO:0046700	Heterocycle catabolic process
dgt	511145.b0160	GO Process	GO:0055086	Nucleobase-containing small molecule metabolic process
dgt	511145.b0160	GO Process	GO:0071704	Organic substance metabolic process
dgt	511145.b0160	GO Process	GO:0072521	Purine-containing compound metabolic process
dgt	511145.b0160	GO Process	GO:0072523	Purine-containing compound catabolic process
dgt	511145.b0160	GO Process	GO:1901135	Carbohydrate derivative metabolic process
dgt	511145.b0160	GO Process	GO:1901136	Carbohydrate derivative catabolic process
dgt	511145.b0160	GO Process	GO:1901292	Nucleoside phosphate catabolic process
dgt	511145.b0160	GO Process	GO:1901360	Organic cyclic compound metabolic process
dgt	511145.b0160	GO Process	GO:1901361	Organic cyclic compound catabolic process
dgt	511145.b0160	GO Process	GO:1901564	Organonitrogen compound metabolic process
dgt	511145.b0160	GO Process	GO:1901565	Organonitrogen compound catabolic process
dgt	511145.b0160	GO Process	GO:1901575	Organic substance catabolic process
dgt	511145.b0160	GO Function	GO:0000287	Magnesium ion binding
dgt	511145.b0160	GO Function	GO:0003676	Nucleic acid binding
dgt	511145.b0160	GO Function	GO:0003677	DNA binding
dgt	511145.b0160	GO Function	GO:0003697	Single-stranded DNA binding
dgt	511145.b0160	GO Function	GO:0003824	Catalytic activity
dgt	511145.b0160	GO Function	GO:0003924	GTPase activity
dgt	511145.b0160	GO Function	GO:0005488	Binding
dgt	511145.b0160	GO Function	GO:0005515	Protein binding
dgt	511145.b0160	GO Function	GO:0008832	dGTPase activity
dgt	511145.b0160	GO Function	GO:0016462	Pyrophosphatase activity
dgt	511145.b0160	GO Function	GO:0016787	Hydrolase activity
dgt	511145.b0160	GO Function	GO:0016788	Hydrolase activity, acting on ester bonds
dgt	511145.b0160	GO Function	GO:0016793	Triphosphoric monoester hydrolase activity
dgt	511145.b0160	GO Function	GO:0016817	Hydrolase activity, acting on acid anhydrides
dgt	511145.b0160	GO Function	GO:0016818	Hydrolase activity, acting on acid anhydrides, in phosphorus-containing anhydrides
dgt	511145.b0160	GO Function	GO:0017111	Nucleoside-triphosphatase activity
dgt	511145.b0160	GO Function	GO:0030145	Manganese ion binding
dgt	511145.b0160	GO Function	GO:0042578	Phosphoric ester hydrolase activity
dgt	511145.b0160	GO Function	GO:0042802	Identical protein binding
dgt	511145.b0160	GO Function	GO:0043167	Ion binding
dgt	511145.b0160	GO Function	GO:0043169	Cation binding
dgt	511145.b0160	GO Function	GO:0046872	Metal ion binding
dgt	511145.b0160	GO Function	GO:0046914	Transition metal ion binding
dgt	511145.b0160	GO Function	GO:0050897	Cobalt ion binding
dgt	511145.b0160	GO Function	GO:0097159	Organic cyclic compound binding
dgt	511145.b0160	GO Function	GO:1901363	Heterocyclic compound binding
dgt	511145.b0160	STRING clusters	CL:6207	Mostly uncharacterized, incl. Degradation of aromatic compounds, and Uracil catabolic process
dgt	511145.b0160	STRING clusters	CL:6208	Mostly uncharacterized, incl. 3-phenylpropionate catabolic process, and Uracil catabolic process
dgt	511145.b0160	STRING clusters	CL:6318	Mixed, incl. LysE type translocator, and Major facilitator superfamily
dgt	511145.b0160	STRING clusters	CL:6319	Mixed, incl. LysE type translocator, and EamA-like transporter family
dgt	511145.b0160	STRING clusters	CL:6365	Glycine transporter, and dGTP catabolic process
dgt	511145.b0160	KEGG	eco00230	Purine metabolism
dgt	511145.b0160	KEGG	eco01100	Metabolic pathways
dgt	511145.b0160	UniProt Keywords	KW-0378	Hydrolase
dgt	511145.b0160	UniProt Keywords	KW-0460	Magnesium
dgt	511145.b0160	InterPro	IPR003607	HD/PDEase domain
dgt	511145.b0160	InterPro	IPR006261	dNTP triphosphohydrolase
dgt	511145.b0160	InterPro	IPR006674	HD domain
dgt	511145.b0160	InterPro	IPR020779	dNTP triphosphohydrolase, type 1
dgt	511145.b0160	InterPro	IPR023293	Deoxyguanosinetriphosphate triphosphohydrolase, central domain superfamily
dgt	511145.b0160	InterPro	IPR026875	Phosphohydrolase-associated domain
dgt	511145.b0160	SMART	SM00471	Metal dependent phosphohydrolases with conserved HD motif.
dnaJ	511145.b0015	GO Process	GO:0006139	Nucleobase-containing compound metabolic process
dnaJ	511145.b0015	GO Process	GO:0006259	DNA metabolic process
dnaJ	511145.b0015	GO Process	GO:0006260	DNA replication
dnaJ	511145.b0015	GO Process	GO:0006355	Regulation of transcription, DNA-templated
dnaJ	511145.b0015	GO Process	GO:0006457	Protein folding
dnaJ	511145.b0015	GO Process	GO:0006458	De novo protein folding
dnaJ	511145.b0015	GO Process	GO:0006725	Cellular aromatic compound metabolic process
dnaJ	511145.b0015	GO Process	GO:0006807	Nitrogen compound metabolic process
dnaJ	511145.b0015	GO Process	GO:0006950	Response to stress
dnaJ	511145.b0015	GO Process	GO:0008152	Metabolic process
dnaJ	511145.b0015	GO Process	GO:0009266	Response to temperature stimulus
dnaJ	511145.b0015	GO Process	GO:0009408	Response to heat
dnaJ	511145.b0015	GO Process	GO:0009628	Response to abiotic stimulus
dnaJ	511145.b0015	GO Process	GO:0009889	Regulation of biosynthetic process
dnaJ	511145.b0015	GO Process	GO:0009890	Negative regulation of biosynthetic process
dnaJ	511145.b0015	GO Process	GO:0009892	Negative regulation of metabolic process
dnaJ	511145.b0015	GO Process	GO:0009987	Cellular process
dnaJ	511145.b0015	GO Process	GO:0010468	Regulation of gene expression
dnaJ	511145.b0015	GO Process	GO:0010556	Regulation of macromolecule biosynthetic process
dnaJ	511145.b0015	GO Process	GO:0010558	Negative regulation of macromolecule biosynthetic process
dnaJ	511145.b0015	GO Process	GO:0010605	Negative regulation of macromolecule metabolic process
dnaJ	511145.b0015	GO Process	GO:0016032	Viral process
dnaJ	511145.b0015	GO Process	GO:0016043	Cellular component organization
dnaJ	511145.b0015	GO Process	GO:0019219	Regulation of nucleobase-containing compound metabolic process
dnaJ	511145.b0015	GO Process	GO:0019222	Regulation of metabolic process
dnaJ	511145.b0015	GO Process	GO:0022607	Cellular component assembly
dnaJ	511145.b0015	GO Process	GO:0031323	Regulation of cellular metabolic process
dnaJ	511145.b0015	GO Process	GO:0031324	Negative regulation of cellular metabolic process
dnaJ	511145.b0015	GO Process	GO:0031326	Regulation of cellular biosynthetic process
dnaJ	511145.b0015	GO Process	GO:0031327	Negative regulation of cellular biosynthetic process
dnaJ	511145.b0015	GO Process	GO:0034641	Cellular nitrogen compound metabolic process
dnaJ	511145.b0015	GO Process	GO:0042026	Protein refolding
dnaJ	511145.b0015	GO Process	GO:0043170	Macromolecule metabolic process
dnaJ	511145.b0015	GO Process	GO:0043933	Protein-containing complex organization
dnaJ	511145.b0015	GO Process	GO:0044085	Cellular component biogenesis
dnaJ	511145.b0015	GO Process	GO:0044237	Cellular metabolic process
dnaJ	511145.b0015	GO Process	GO:0044238	Primary metabolic process
dnaJ	511145.b0015	GO Process	GO:0044260	Cellular macromolecule metabolic process
dnaJ	511145.b0015	GO Process	GO:0045892	Negative regulation of transcription, DNA-templated
dnaJ	511145.b0015	GO Process	GO:0045934	Negative regulation of nucleobase-containing compound metabolic process
dnaJ	511145.b0015	GO Process	GO:0046483	Heterocycle metabolic process
dnaJ	511145.b0015	GO Process	GO:0048519	Negative regulation of biological process
dnaJ	511145.b0015	GO Process	GO:0048523	Negative regulation of cellular process
dnaJ	511145.b0015	GO Process	GO:0050789	Regulation of biological process
dnaJ	511145.b0015	GO Process	GO:0050794	Regulation of cellular process
dnaJ	511145.b0015	GO Process	GO:0050896	Response to stimulus
dnaJ	511145.b0015	GO Process	GO:0051084	De novo post-translational protein folding
dnaJ	511145.b0015	GO Process	GO:0051085	Chaperone cofactor-dependent protein refolding
dnaJ	511145.b0015	GO Process	GO:0051171	Regulation of nitrogen compound metabolic process
dnaJ	511145.b0015	GO Process	GO:0051172	Negative regulation of nitrogen compound metabolic process
dnaJ	511145.b0015	GO Process	GO:0051252	Regulation of RNA metabolic process
dnaJ	511145.b0015	GO Process	GO:0051253	Negative regulation of RNA metabolic process
dnaJ	511145.b0015	GO Process	GO:0060255	Regulation of macromolecule metabolic process
dnaJ	511145.b0015	GO Process	GO:0061077	Chaperone-mediated protein folding
dnaJ	511145.b0015	GO Process	GO:0065003	Protein-containing complex assembly
dnaJ	511145.b0015	GO Process	GO:0065007	Biological regulation
dnaJ	511145.b0015	GO Process	GO:0071704	Organic substance metabolic process
dnaJ	511145.b0015	GO Process	GO:0071840	Cellular component organization or biogenesis
dnaJ	511145.b0015	GO Process	GO:0080090	Regulation of primary metabolic process
dnaJ	511145.b0015	GO Process	GO:0090304	Nucleic acid metabolic process
dnaJ	511145.b0015	GO Process	GO:1901360	Organic cyclic compound metabolic process
dnaJ	511145.b0015	GO Process	GO:1902679	Negative regulation of RNA biosynthetic process
dnaJ	511145.b0015	GO Process	GO:1903506	Regulation of nucleic acid-templated transcription
dnaJ	511145.b0015	GO Process	GO:1903507	Negative regulation of nucleic acid-templated transcription
dnaJ	511145.b0015	GO Process	GO:2001141	Regulation of RNA biosynthetic process
dnaJ	511145.b0015	GO Function	GO:0000166	Nucleotide binding
dnaJ	511145.b0015	GO Function	GO:0003712	Transcription coregulator activity
dnaJ	511145.b0015	GO Function	GO:0003714	Transcription corepressor activity
dnaJ	511145.b0015	GO Function	GO:0003756	Protein disulfide isomerase activity
dnaJ	511145.b0015	GO Function	GO:0003824	Catalytic activity
dnaJ	511145.b0015	GO Function	GO:0005488	Binding
dnaJ	511145.b0015	GO Function	GO:0005515	Protein binding
dnaJ	511145.b0015	GO Function	GO:0005524	ATP binding
dnaJ	511145.b0015	GO Function	GO:0008270	Zinc ion binding
dnaJ	511145.b0015	GO Function	GO:0015035	Protein-disulfide reductase activity
dnaJ	511145.b0015	GO Function	GO:0015036	Disulfide oxidoreductase activity
dnaJ	511145.b0015	GO Function	GO:0016491	Oxidoreductase activity
dnaJ	511145.b0015	GO Function	GO:0016667	Oxidoreductase activity, acting on a sulfur group of donors
dnaJ	511145.b0015	GO Function	GO:0016853	Isomerase activity
dnaJ	511145.b0015	GO Function	GO:0016860	Intramolecular oxidoreductase activity
dnaJ	511145.b0015	GO Function	GO:0016864	Intramolecular oxidoreductase activity, transposing S-S bonds
dnaJ	511145.b0015	GO Function	GO:0016989	Sigma factor antagonist activity
dnaJ	511145.b0015	GO Function	GO:0017076	Purine nucleotide binding
dnaJ	511145.b0015	GO Function	GO:0030554	Adenyl nucleotide binding
dnaJ	511145.b0015	GO Function	GO:0031072	Heat shock protein binding
dnaJ	511145.b0015	GO Function	GO:0032553	Ribonucleotide binding
dnaJ	511145.b0015	GO Function	GO:0032555	Purine ribonucleotide binding
dnaJ	511145.b0015	GO Function	GO:0032559	Adenyl ribonucleotide binding
dnaJ	511145.b0015	GO Function	GO:0035639	Purine ribonucleoside triphosphate binding
dnaJ	511145.b0015	GO Function	GO:0036094	Small molecule binding
dnaJ	511145.b0015	GO Function	GO:0043167	Ion binding
dnaJ	511145.b0015	GO Function	GO:0043168	Anion binding
dnaJ	511145.b0015	GO Function	GO:0043169	Cation binding
dnaJ	511145.b0015	GO Function	GO:0046872	Metal ion binding
dnaJ	511145.b0015	GO Function	GO:0046914	Transition metal ion binding
dnaJ	511145.b0015	GO Function	GO:0051082	Unfolded protein binding
dnaJ	511145.b0015	GO Function	GO:0051087	Chaperone binding
dnaJ	511145.b0015	GO Function	GO:0097159	Organic cyclic compound binding
dnaJ	511145.b0015	GO Function	GO:0097367	Carbohydrate derivative binding
dnaJ	511145.b0015	GO Function	GO:0140096	Catalytic activity, acting on a protein
dnaJ	511145.b0015	GO Function	GO:0140110	Transcription regulator activity
dnaJ	511145.b0015	GO Function	GO:1901265	Nucleoside phosphate binding
dnaJ	511145.b0015	GO Function	GO:1901363	Heterocyclic compound binding
dnaJ	511145.b0015	GO Component	GO:0005622	Intracellular anatomical structure
dnaJ	511145.b0015	GO Component	GO:0005737	Cytoplasm
dnaJ	511145.b0015	GO Component	GO:0005829	Cytosol
dnaJ	511145.b0015	GO Component	GO:0016020	Membrane
dnaJ	511145.b0015	GO Component	GO:0032991	Protein-containing complex
dnaJ	511145.b0015	GO Component	GO:0110165	Cellular anatomical entity
dnaJ	511145.b0015	STRING clusters	CL:594	Mixed, incl. Response to heat, and DnaJ domain
dnaJ	511145.b0015	STRING clusters	CL:596	Response to heat, and DnaJ domain
dnaJ	511145.b0015	STRING clusters	CL:597	Mixed, incl. Protein folding chaperone, and Cellular response to heat
dnaJ	511145.b0015	STRING clusters	CL:599	Unfolded protein binding, and Cellular response to heat
dnaJ	511145.b0015	STRING clusters	CL:602	Proteasome complex, and De novo post-translational protein folding
dnaJ	511145.b0015	STRING clusters	CL:604	De novo post-translational protein folding, and Clp, repeat (R) domain
dnaJ	511145.b0015	COMPARTMENTS	GOCC:0005622	Intracellular
dnaJ	511145.b0015	COMPARTMENTS	GOCC:0005737	Cytoplasm
dnaJ	511145.b0015	COMPARTMENTS	GOCC:0005829	Cytosol
dnaJ	511145.b0015	COMPARTMENTS	GOCC:0110165	Cellular anatomical entity
dnaJ	511145.b0015	UniProt Keywords	KW-0143	Chaperone
dnaJ	511145.b0015	UniProt Keywords	KW-0235	DNA replication
dnaJ	511145.b0015	UniProt Keywords	KW-0346	Stress response
dnaJ	511145.b0015	UniProt Keywords	KW-0479	Metal-binding
dnaJ	511145.b0015	UniProt Keywords	KW-0677	Repeat
dnaJ	511145.b0015	UniProt Keywords	KW-0862	Zinc
dnaJ	511145.b0015	UniProt Keywords	KW-0863	Zinc-finger
dnaJ	511145.b0015	UniProt Keywords	KW-0963	Cytoplasm
dnaJ	511145.b0015	Pfam	PF00684	DnaJ central domain
dnaJ	511145.b0015	InterPro	IPR001305	Heat shock protein DnaJ, cysteine-rich domain
dnaJ	511145.b0015	InterPro	IPR001623	DnaJ domain
dnaJ	511145.b0015	InterPro	IPR002939	Chaperone DnaJ, C-terminal
dnaJ	511145.b0015	InterPro	IPR008971	HSP40/DnaJ peptide-binding
dnaJ	511145.b0015	InterPro	IPR012724	Chaperone DnaJ
dnaJ	511145.b0015	InterPro	IPR018253	DnaJ domain, conserved site
dnaJ	511145.b0015	InterPro	IPR036410	Heat shock protein DnaJ, cysteine-rich domain superfamily
dnaJ	511145.b0015	InterPro	IPR036869	Chaperone J-domain superfamily
dnaJ	511145.b0015	SMART	SM00271	DnaJ molecular chaperone homology domain
dxr	511145.b0173	GO Process	GO:0006081	Cellular aldehyde metabolic process
dxr	511145.b0173	GO Process	GO:0006082	Organic acid metabolic process
dxr	511145.b0173	GO Process	GO:0006090	Pyruvate metabolic process
dxr	511145.b0173	GO Process	GO:0006629	Lipid metabolic process
dxr	511145.b0173	GO Process	GO:0006644	Phospholipid metabolic process
dxr	511145.b0173	GO Process	GO:0006720	Isoprenoid metabolic process
dxr	511145.b0173	GO Process	GO:0006721	Terpenoid metabolic process
dxr	511145.b0173	GO Process	GO:0006793	Phosphorus metabolic process
dxr	511145.b0173	GO Process	GO:0006796	Phosphate-containing compound metabolic process
dxr	511145.b0173	GO Process	GO:0008152	Metabolic process
dxr	511145.b0173	GO Process	GO:0008299	Isoprenoid biosynthetic process
dxr	511145.b0173	GO Process	GO:0008610	Lipid biosynthetic process
dxr	511145.b0173	GO Process	GO:0008654	Phospholipid biosynthetic process
dxr	511145.b0173	GO Process	GO:0009058	Biosynthetic process
dxr	511145.b0173	GO Process	GO:0009240	Isopentenyl diphosphate biosynthetic process
dxr	511145.b0173	GO Process	GO:0009987	Cellular process
dxr	511145.b0173	GO Process	GO:0016114	Terpenoid biosynthetic process
dxr	511145.b0173	GO Process	GO:0019288	Isopentenyl diphosphate biosynthetic process, methylerythritol 4-phosphate pathway
dxr	511145.b0173	GO Process	GO:0019637	Organophosphate metabolic process
dxr	511145.b0173	GO Process	GO:0019682	glyceraldehyde-3-phosphate metabolic process
dxr	511145.b0173	GO Process	GO:0019752	Carboxylic acid metabolic process
dxr	511145.b0173	GO Process	GO:0032787	Monocarboxylic acid metabolic process
dxr	511145.b0173	GO Process	GO:0043436	Oxoacid metabolic process
dxr	511145.b0173	GO Process	GO:0044237	Cellular metabolic process
dxr	511145.b0173	GO Process	GO:0044238	Primary metabolic process
dxr	511145.b0173	GO Process	GO:0044249	Cellular biosynthetic process
dxr	511145.b0173	GO Process	GO:0044255	Cellular lipid metabolic process
dxr	511145.b0173	GO Process	GO:0044281	Small molecule metabolic process
dxr	511145.b0173	GO Process	GO:0046490	Isopentenyl diphosphate metabolic process
dxr	511145.b0173	GO Process	GO:0051483	Terpenoid biosynthetic process, mevalonate-independent
dxr	511145.b0173	GO Process	GO:0051484	Isopentenyl diphosphate biosynthetic process, methylerythritol 4-phosphate pathway involved in terpenoid biosynthetic process
dxr	511145.b0173	GO Process	GO:0071704	Organic substance metabolic process
dxr	511145.b0173	GO Process	GO:0090407	Organophosphate biosynthetic process
dxr	511145.b0173	GO Process	GO:1901135	Carbohydrate derivative metabolic process
dxr	511145.b0173	GO Process	GO:1901576	Organic substance biosynthetic process
dxr	511145.b0173	GO Function	GO:0000166	Nucleotide binding
dxr	511145.b0173	GO Function	GO:0003824	Catalytic activity
dxr	511145.b0173	GO Function	GO:0005488	Binding
dxr	511145.b0173	GO Function	GO:0005515	Protein binding
dxr	511145.b0173	GO Function	GO:0016491	Oxidoreductase activity
dxr	511145.b0173	GO Function	GO:0016614	Oxidoreductase activity, acting on CH-OH group of donors
dxr	511145.b0173	GO Function	GO:0016616	Oxidoreductase activity, acting on the CH-OH group of donors, NAD or NADP as acceptor
dxr	511145.b0173	GO Function	GO:0030145	Manganese ion binding
dxr	511145.b0173	GO Function	GO:0030604	1-deoxy-D-xylulose-5-phosphate reductoisomerase activity
dxr	511145.b0173	GO Function	GO:0036094	Small molecule binding
dxr	511145.b0173	GO Function	GO:0042802	Identical protein binding
dxr	511145.b0173	GO Function	GO:0043167	Ion binding
dxr	511145.b0173	GO Function	GO:0043168	Anion binding
dxr	511145.b0173	GO Function	GO:0043169	Cation binding
dxr	511145.b0173	GO Function	GO:0046872	Metal ion binding
dxr	511145.b0173	GO Function	GO:0046914	Transition metal ion binding
dxr	511145.b0173	GO Function	GO:0050661	NADP binding
dxr	511145.b0173	GO Function	GO:0070402	NADPH binding
dxr	511145.b0173	GO Function	GO:0097159	Organic cyclic compound binding
dxr	511145.b0173	GO Function	GO:1901265	Nucleoside phosphate binding
dxr	511145.b0173	GO Function	GO:1901363	Heterocyclic compound binding
dxr	511145.b0173	GO Component	GO:0032991	Protein-containing complex
dxr	511145.b0173	GO Component	GO:1990065	Dxr protein complex
dxr	511145.b0173	STRING clusters	CL:2534	Mixed, incl. Quinone metabolic process, and Tetrapyrrole biosynthetic process
dxr	511145.b0173	STRING clusters	CL:2638	Isoprene biosynthesis, and Menaquinone biosynthesis
dxr	511145.b0173	STRING clusters	CL:2670	Isoprene biosynthesis
dxr	511145.b0173	KEGG	eco00900	Terpenoid backbone biosynthesis
dxr	511145.b0173	KEGG	eco01100	Metabolic pathways
dxr	511145.b0173	KEGG	eco01110	Biosynthesis of secondary metabolites
dxr	511145.b0173	COMPARTMENTS	GOCC:0032991	Protein-containing complex
dxr	511145.b0173	COMPARTMENTS	GOCC:1990065	Dxr protein complex
dxr	511145.b0173	UniProt Keywords	KW-0170	Cobalt
dxr	511145.b0173	UniProt Keywords	KW-0414	Isoprene biosynthesis
dxr	511145.b0173	UniProt Keywords	KW-0460	Magnesium
dxr	511145.b0173	UniProt Keywords	KW-0464	Manganese
dxr	511145.b0173	UniProt Keywords	KW-0479	Metal-binding
dxr	511145.b0173	UniProt Keywords	KW-0521	NADP
dxr	511145.b0173	UniProt Keywords	KW-0560	Oxidoreductase
dxr	511145.b0173	Pfam	PF02670	1-deoxy-D-xylulose 5-phosphate reductoisomerase
dxr	511145.b0173	Pfam	PF08436	1-deoxy-D-xylulose 5-phosphate reductoisomerase C-terminal domain
dxr	511145.b0173	Pfam	PF13288	DXP reductoisomerase C-terminal domain
dxr	511145.b0173	InterPro	IPR003821	1-deoxy-D-xylulose 5-phosphate reductoisomerase
dxr	511145.b0173	InterPro	IPR013512	1-deoxy-D-xylulose 5-phosphate reductoisomerase, N-terminal
dxr	511145.b0173	InterPro	IPR013644	1-deoxy-D-xylulose 5-phosphate reductoisomerase, C-terminal
dxr	511145.b0173	InterPro	IPR026877	DXP reductoisomerase C-terminal domain
dxr	511145.b0173	InterPro	IPR036169	DXP reductoisomerase, C-terminal domain superfamily
dxr	511145.b0173	InterPro	IPR036291	NAD(P)-binding domain superfamily
eco	511145.b2209	GO Process	GO:0009892	Negative regulation of metabolic process
eco	511145.b2209	GO Process	GO:0010466	Negative regulation of peptidase activity
eco	511145.b2209	GO Process	GO:0010605	Negative regulation of macromolecule metabolic process
eco	511145.b2209	GO Process	GO:0010951	Negative regulation of endopeptidase activity
eco	511145.b2209	GO Process	GO:0019222	Regulation of metabolic process
eco	511145.b2209	GO Process	GO:0030162	Regulation of proteolysis
eco	511145.b2209	GO Process	GO:0043086	Negative regulation of catalytic activity
eco	511145.b2209	GO Process	GO:0044092	Negative regulation of molecular function
eco	511145.b2209	GO Process	GO:0045861	Negative regulation of proteolysis
eco	511145.b2209	GO Process	GO:0048519	Negative regulation of biological process
eco	511145.b2209	GO Process	GO:0050789	Regulation of biological process
eco	511145.b2209	GO Process	GO:0050790	Regulation of catalytic activity
eco	511145.b2209	GO Process	GO:0051171	Regulation of nitrogen compound metabolic process
eco	511145.b2209	GO Process	GO:0051172	Negative regulation of nitrogen compound metabolic process
eco	511145.b2209	GO Process	GO:0051246	Regulation of protein metabolic process
eco	511145.b2209	GO Process	GO:0051248	Negative regulation of protein metabolic process
eco	511145.b2209	GO Process	GO:0051336	Regulation of hydrolase activity
eco	511145.b2209	GO Process	GO:0051346	Negative regulation of hydrolase activity
eco	511145.b2209	GO Process	GO:0052547	Regulation of peptidase activity
eco	511145.b2209	GO Process	GO:0052548	Regulation of endopeptidase activity
eco	511145.b2209	GO Process	GO:0060255	Regulation of macromolecule metabolic process
eco	511145.b2209	GO Process	GO:0065007	Biological regulation
eco	511145.b2209	GO Process	GO:0065009	Regulation of molecular function
eco	511145.b2209	GO Process	GO:0080090	Regulation of primary metabolic process
eco	511145.b2209	GO Function	GO:0004857	Enzyme inhibitor activity
eco	511145.b2209	GO Function	GO:0004866	Endopeptidase inhibitor activity
eco	511145.b2209	GO Function	GO:0004867	Serine-type endopeptidase inhibitor activity
eco	511145.b2209	GO Function	GO:0030234	Enzyme regulator activity
eco	511145.b2209	GO Function	GO:0030414	Peptidase inhibitor activity
eco	511145.b2209	GO Function	GO:0061134	Peptidase regulator activity
eco	511145.b2209	GO Function	GO:0061135	Endopeptidase regulator activity
eco	511145.b2209	GO Function	GO:0098772	Molecular function regulator activity
eco	511145.b2209	GO Component	GO:0030288	Outer membrane-bounded periplasmic space
eco	511145.b2209	GO Component	GO:0030313	Cell envelope
eco	511145.b2209	GO Component	GO:0031975	Envelope
eco	511145.b2209	GO Component	GO:0042597	Periplasmic space
eco	511145.b2209	GO Component	GO:0110165	Cellular anatomical entity
eco	511145.b2209	STRING clusters	CL:6765	Mixed, incl. DNA recombination, and Hydrolase activity, acting on ester bonds
eco	511145.b2209	STRING clusters	CL:6766	Mostly uncharacterized, incl. Restriction system, and Aldaric acid metabolic process
eco	511145.b2209	STRING clusters	CL:6767	Mostly uncharacterized, incl. Aldaric acid metabolic process, and Purine metabolism
eco	511145.b2209	STRING clusters	CL:6768	Mostly uncharacterized, incl. Sulfatase, N-terminal, and Anaerobic sulphatase maturase, radical SAM
eco	511145.b2209	STRING clusters	CL:6769	Mostly uncharacterized, incl. Sulfatase, N-terminal, and Anaerobic sulphatase maturase, radical SAM
eco	511145.b2209	STRING clusters	CL:6770	Mostly uncharacterized, incl. Pertactin virulence factor family, and Phosphoethanolamine transferase
eco	511145.b2209	STRING clusters	CL:6771	Mostly uncharacterized, incl. Phosphoethanolamine transferase, and Tryptophan/tyrosine permease family
eco	511145.b2209	STRING clusters	CL:6805	Mixed, incl. Tryptophan/tyrosine permease family, and Protease inhibitor
eco	511145.b2209	COMPARTMENTS	GOCC:0030288	Outer membrane-bounded periplasmic space
eco	511145.b2209	COMPARTMENTS	GOCC:0030313	Cell envelope
eco	511145.b2209	COMPARTMENTS	GOCC:0031975	Envelope
eco	511145.b2209	COMPARTMENTS	GOCC:0042597	Periplasmic space
eco	511145.b2209	COMPARTMENTS	GOCC:0110165	Cellular anatomical entity
eco	511145.b2209	UniProt Keywords	KW-0574	Periplasm
eco	511145.b2209	UniProt Keywords	KW-0646	Protease inhibitor
eco	511145.b2209	UniProt Keywords	KW-0722	Serine protease inhibitor
eco	511145.b2209	UniProt Keywords	KW-0732	Signal
eco	511145.b2209	UniProt Keywords	KW-1015	Disulfide bond
eco	511145.b2209	Pfam	PF03974	Ecotin
eco	511145.b2209	InterPro	IPR005658	Proteinase inhibitor I11, ecotin
eco	511145.b2209	InterPro	IPR023084	Proteinase inhibitor I11, ecotin, gammaproteobacteria
eco	511145.b2209	InterPro	IPR027438	Ecotin, C-terminal
eco	511145.b2209	InterPro	IPR036198	Ecotin superfamily
eda	511145.b1850	GO Process	GO:0008152	Metabolic process
eda	511145.b1850	GO Function	GO:0003824	Catalytic activity
eda	511145.b1850	GO Function	GO:0005488	Binding
eda	511145.b1850	GO Function	GO:0005515	Protein binding
eda	511145.b1850	GO Function	GO:0008675	2-dehydro-3-deoxy-phosphogluconate aldolase activity
eda	511145.b1850	GO Function	GO:0008700	4-hydroxy-2-oxoglutarate aldolase activity
eda	511145.b1850	GO Function	GO:0008948	Oxaloacetate decarboxylase activity
eda	511145.b1850	GO Function	GO:0016829	Lyase activity
eda	511145.b1850	GO Function	GO:0016830	Carbon-carbon lyase activity
eda	511145.b1850	GO Function	GO:0016831	Carboxy-lyase activity
eda	511145.b1850	GO Function	GO:0016832	Aldehyde-lyase activity
eda	511145.b1850	GO Function	GO:0016833	Oxo-acid-lyase activity
eda	511145.b1850	GO Function	GO:0042802	Identical protein binding
eda	511145.b1850	GO Function	GO:0106009	(4S)-4-hydroxy-2-oxoglutarate aldolase activity
eda	511145.b1850	GO Component	GO:0005622	Intracellular anatomical structure
eda	511145.b1850	GO Component	GO:0005737	Cytoplasm
eda	511145.b1850	GO Component	GO:0005829	Cytosol
eda	511145.b1850	GO Component	GO:0016020	Membrane
eda	511145.b1850	GO Component	GO:0110165	Cellular anatomical entity
eda	511145.b1850	STRING clusters	CL:2010	Mixed, incl. Carbon metabolism, and Fatty acid metabolic process
eda	511145.b1850	STRING clusters	CL:2011	Carbon metabolism, and Oxidative phosphorylation
eda	511145.b1850	STRING clusters	CL:2012	Carbon metabolism, and Pyruvate metabolism
eda	511145.b1850	STRING clusters	CL:2013	Carbon metabolism, and Formate C-acetyltransferase activity
eda	511145.b1850	STRING clusters	CL:2109	Pentose phosphate pathway, and Glycolysis
eda	511145.b1850	STRING clusters	CL:2170	Mixed, incl. KDPG/KHG aldolase, and Glucokinase activity
eda	511145.b1850	KEGG	eco00030	Pentose phosphate pathway
eda	511145.b1850	KEGG	eco00630	Glyoxylate and dicarboxylate metabolism
eda	511145.b1850	KEGG	eco01100	Metabolic pathways
eda	511145.b1850	KEGG	eco01120	Microbial metabolism in diverse environments
eda	511145.b1850	KEGG	eco01200	Carbon metabolism
eda	511145.b1850	COMPARTMENTS	GOCC:0005622	Intracellular
eda	511145.b1850	COMPARTMENTS	GOCC:0005737	Cytoplasm
eda	511145.b1850	COMPARTMENTS	GOCC:0005829	Cytosol
eda	511145.b1850	COMPARTMENTS	GOCC:0110165	Cellular anatomical entity
eda	511145.b1850	UniProt Keywords	KW-0456	Lyase
eda	511145.b1850	UniProt Keywords	KW-0511	Multifunctional enzyme
eda	511145.b1850	UniProt Keywords	KW-0704	Schiff base
eda	511145.b1850	UniProt Keywords	KW-0963	Cytoplasm
eda	511145.b1850	Pfam	PF01081	KDPG and KHG aldolase
eda	511145.b1850	InterPro	IPR000887	KDPG/KHG aldolase
eda	511145.b1850	InterPro	IPR013785	Aldolase-type TIM barrel
eda	511145.b1850	InterPro	IPR031337	KDPG/KHG aldolase, active site 1
eda	511145.b1850	InterPro	IPR031338	KDPG/KHG aldolase, active site 2
efp	511145.b4147	GO Process	GO:0006412	Translation
efp	511145.b4147	GO Process	GO:0006414	Translational elongation
efp	511145.b4147	GO Process	GO:0006417	Regulation of translation
efp	511145.b4147	GO Process	GO:0006448	Regulation of translational elongation
efp	511145.b4147	GO Process	GO:0006518	Peptide metabolic process
efp	511145.b4147	GO Process	GO:0006807	Nitrogen compound metabolic process
efp	511145.b4147	GO Process	GO:0008152	Metabolic process
efp	511145.b4147	GO Process	GO:0009058	Biosynthetic process
efp	511145.b4147	GO Process	GO:0009059	Macromolecule biosynthetic process
efp	511145.b4147	GO Process	GO:0009889	Regulation of biosynthetic process
efp	511145.b4147	GO Process	GO:0009890	Negative regulation of biosynthetic process
efp	511145.b4147	GO Process	GO:0009892	Negative regulation of metabolic process
efp	511145.b4147	GO Process	GO:0009987	Cellular process
efp	511145.b4147	GO Process	GO:0010467	Gene expression
efp	511145.b4147	GO Process	GO:0010468	Regulation of gene expression
efp	511145.b4147	GO Process	GO:0010556	Regulation of macromolecule biosynthetic process
efp	511145.b4147	GO Process	GO:0010558	Negative regulation of macromolecule biosynthetic process
efp	511145.b4147	GO Process	GO:0010605	Negative regulation of macromolecule metabolic process
efp	511145.b4147	GO Process	GO:0010608	Post-transcriptional regulation of gene expression
efp	511145.b4147	GO Process	GO:0010629	Negative regulation of gene expression
efp	511145.b4147	GO Process	GO:0017148	Negative regulation of translation
efp	511145.b4147	GO Process	GO:0019222	Regulation of metabolic process
efp	511145.b4147	GO Process	GO:0019538	Protein metabolic process
efp	511145.b4147	GO Process	GO:0031323	Regulation of cellular metabolic process
efp	511145.b4147	GO Process	GO:0031324	Negative regulation of cellular metabolic process
efp	511145.b4147	GO Process	GO:0031326	Regulation of cellular biosynthetic process
efp	511145.b4147	GO Process	GO:0031327	Negative regulation of cellular biosynthetic process
efp	511145.b4147	GO Process	GO:0034248	Regulation of cellular amide metabolic process
efp	511145.b4147	GO Process	GO:0034249	Negative regulation of cellular amide metabolic process
efp	511145.b4147	GO Process	GO:0034641	Cellular nitrogen compound metabolic process
efp	511145.b4147	GO Process	GO:0034645	Cellular macromolecule biosynthetic process
efp	511145.b4147	GO Process	GO:0043043	Peptide biosynthetic process
efp	511145.b4147	GO Process	GO:0043170	Macromolecule metabolic process
efp	511145.b4147	GO Process	GO:0043603	Cellular amide metabolic process
efp	511145.b4147	GO Process	GO:0043604	Amide biosynthetic process
efp	511145.b4147	GO Process	GO:0044237	Cellular metabolic process
efp	511145.b4147	GO Process	GO:0044238	Primary metabolic process
efp	511145.b4147	GO Process	GO:0044249	Cellular biosynthetic process
efp	511145.b4147	GO Process	GO:0044260	Cellular macromolecule metabolic process
efp	511145.b4147	GO Process	GO:0044271	Cellular nitrogen compound biosynthetic process
efp	511145.b4147	GO Process	GO:0045900	Negative regulation of translational elongation
efp	511145.b4147	GO Process	GO:0048519	Negative regulation of biological process
efp	511145.b4147	GO Process	GO:0048523	Negative regulation of cellular process
efp	511145.b4147	GO Process	GO:0050789	Regulation of biological process
efp	511145.b4147	GO Process	GO:0050794	Regulation of cellular process
efp	511145.b4147	GO Process	GO:0051171	Regulation of nitrogen compound metabolic process
efp	511145.b4147	GO Process	GO:0051172	Negative regulation of nitrogen compound metabolic process
efp	511145.b4147	GO Process	GO:0051246	Regulation of protein metabolic process
efp	511145.b4147	GO Process	GO:0051248	Negative regulation of protein metabolic process
efp	511145.b4147	GO Process	GO:0060255	Regulation of macromolecule metabolic process
efp	511145.b4147	GO Process	GO:0065007	Biological regulation
efp	511145.b4147	GO Process	GO:0071704	Organic substance metabolic process
efp	511145.b4147	GO Process	GO:0072344	Rescue of stalled ribosome
efp	511145.b4147	GO Process	GO:0080090	Regulation of primary metabolic process
efp	511145.b4147	GO Process	GO:1901564	Organonitrogen compound metabolic process
efp	511145.b4147	GO Process	GO:1901566	Organonitrogen compound biosynthetic process
efp	511145.b4147	GO Process	GO:1901576	Organic substance biosynthetic process
efp	511145.b4147	GO Process	GO:2000112	Regulation of cellular macromolecule biosynthetic process
efp	511145.b4147	GO Process	GO:2000113	Negative regulation of cellular macromolecule biosynthetic process
efp	511145.b4147	GO Process	GO:2001124	Regulation of translational frameshifting
efp	511145.b4147	GO Process	GO:2001125	Negative regulation of translational frameshifting
efp	511145.b4147	GO Function	GO:0003676	Nucleic acid binding
efp	511145.b4147	GO Function	GO:0003746	Translation elongation factor activity
efp	511145.b4147	GO Function	GO:0005488	Binding
efp	511145.b4147	GO Function	GO:0008135	Translation factor activity, RNA binding
efp	511145.b4147	GO Function	GO:0043021	Ribonucleoprotein complex binding
efp	511145.b4147	GO Function	GO:0043022	Ribosome binding
efp	511145.b4147	GO Function	GO:0044877	Protein-containing complex binding
efp	511145.b4147	GO Function	GO:0045182	Translation regulator activity
efp	511145.b4147	GO Function	GO:0090079	Translation regulator activity, nucleic acid binding
efp	511145.b4147	GO Function	GO:0097159	Organic cyclic compound binding
efp	511145.b4147	GO Function	GO:1901363	Heterocyclic compound binding
efp	511145.b4147	GO Component	GO:0005622	Intracellular anatomical structure
efp	511145.b4147	GO Component	GO:0005737	Cytoplasm
efp	511145.b4147	GO Component	GO:0005829	Cytosol
efp	511145.b4147	GO Component	GO:0110165	Cellular anatomical entity
efp	511145.b4147	STRING clusters	CL:35	Translation, and Protein export
efp	511145.b4147	STRING clusters	CL:366	Elongation factor P, C-terminal, and Bacterial-type EF-P lysine modification
efp	511145.b4147	COMPARTMENTS	GOCC:0005622	Intracellular
efp	511145.b4147	COMPARTMENTS	GOCC:0005737	Cytoplasm
efp	511145.b4147	COMPARTMENTS	GOCC:0005829	Cytosol
efp	511145.b4147	COMPARTMENTS	GOCC:0110165	Cellular anatomical entity
efp	511145.b4147	UniProt Keywords	KW-0251	Elongation factor
efp	511145.b4147	UniProt Keywords	KW-0379	Hydroxylation
efp	511145.b4147	UniProt Keywords	KW-0648	Protein biosynthesis
efp	511145.b4147	UniProt Keywords	KW-0963	Cytoplasm
efp	511145.b4147	Pfam	PF08207	Elongation factor P (EF-P) KOW-like domain
efp	511145.b4147	InterPro	IPR001059	Translation elongation factor P/YeiP, central
efp	511145.b4147	InterPro	IPR008991	Translation protein SH3-like domain superfamily
efp	511145.b4147	InterPro	IPR011768	Translation elongation factor P
efp	511145.b4147	InterPro	IPR012340	Nucleic acid-binding, OB-fold
efp	511145.b4147	InterPro	IPR013185	Translation elongation factor, KOW-like
efp	511145.b4147	InterPro	IPR013852	Translation elongation factor P/YeiP, conserved site
efp	511145.b4147	InterPro	IPR014722	Ribosomal protein L2, domain 2
efp	511145.b4147	InterPro	IPR015365	Elongation factor P, C-terminal
efp	511145.b4147	InterPro	IPR020599	Translation elongation factor P/YeiP
efp	511145.b4147	SMART	SM00841	Elongation factor P, C-terminal
efp	511145.b4147	SMART	SM01185	Elongation factor P (EF-P) OB domain
erpA	511145.b0156	GO Process	GO:0006091	Generation of precursor metabolites and energy
erpA	511145.b0156	GO Process	GO:0006790	Sulfur compound metabolic process
erpA	511145.b0156	GO Process	GO:0006807	Nitrogen compound metabolic process
erpA	511145.b0156	GO Process	GO:0008152	Metabolic process
erpA	511145.b0156	GO Process	GO:0009060	Aerobic respiration
erpA	511145.b0156	GO Process	GO:0009061	Anaerobic respiration
erpA	511145.b0156	GO Process	GO:0009987	Cellular process
erpA	511145.b0156	GO Process	GO:0010467	Gene expression
erpA	511145.b0156	GO Process	GO:0015980	Energy derivation by oxidation of organic compounds
erpA	511145.b0156	GO Process	GO:0016043	Cellular component organization
erpA	511145.b0156	GO Process	GO:0016226	Iron-sulfur cluster assembly
erpA	511145.b0156	GO Process	GO:0019538	Protein metabolic process
erpA	511145.b0156	GO Process	GO:0022607	Cellular component assembly
erpA	511145.b0156	GO Process	GO:0031163	Metallo-sulfur cluster assembly
erpA	511145.b0156	GO Process	GO:0043170	Macromolecule metabolic process
erpA	511145.b0156	GO Process	GO:0044085	Cellular component biogenesis
erpA	511145.b0156	GO Process	GO:0044237	Cellular metabolic process
erpA	511145.b0156	GO Process	GO:0044238	Primary metabolic process
erpA	511145.b0156	GO Process	GO:0045333	Cellular respiration
erpA	511145.b0156	GO Process	GO:0051604	Protein maturation
erpA	511145.b0156	GO Process	GO:0071704	Organic substance metabolic process
erpA	511145.b0156	GO Process	GO:0071840	Cellular component organization or biogenesis
erpA	511145.b0156	GO Process	GO:0097428	Protein maturation by iron-sulfur cluster transfer
erpA	511145.b0156	GO Process	GO:0106035	Protein maturation by [4Fe-4S] cluster transfer
erpA	511145.b0156	GO Process	GO:1901564	Organonitrogen compound metabolic process
erpA	511145.b0156	GO Function	GO:0005488	Binding
erpA	511145.b0156	GO Function	GO:0005506	Iron ion binding
erpA	511145.b0156	GO Function	GO:0043167	Ion binding
erpA	511145.b0156	GO Function	GO:0043169	Cation binding
erpA	511145.b0156	GO Function	GO:0046872	Metal ion binding
erpA	511145.b0156	GO Function	GO:0046914	Transition metal ion binding
erpA	511145.b0156	GO Function	GO:0051536	Iron-sulfur cluster binding
erpA	511145.b0156	GO Function	GO:0051537	2 iron, 2 sulfur cluster binding
erpA	511145.b0156	GO Function	GO:0051539	4 iron, 4 sulfur cluster binding
erpA	511145.b0156	GO Function	GO:0051540	Metal cluster binding
erpA	511145.b0156	GO Component	GO:0005622	Intracellular anatomical structure
erpA	511145.b0156	GO Component	GO:0005737	Cytoplasm
erpA	511145.b0156	GO Component	GO:0005829	Cytosol
erpA	511145.b0156	GO Component	GO:0110165	Cellular anatomical entity
erpA	511145.b0156	STRING clusters	CL:1242	Mixed, incl. Iron-sulfur cluster assembly, and Antioxidant activity
erpA	511145.b0156	STRING clusters	CL:1243	Mixed, incl. Iron-sulfur cluster assembly, and Antioxidant activity
erpA	511145.b0156	STRING clusters	CL:1327	Iron-sulfur cluster assembly, and tRNA thio-modification
erpA	511145.b0156	STRING clusters	CL:1328	Iron-sulfur cluster assembly
erpA	511145.b0156	STRING clusters	CL:1329	Protein maturation by iron-sulfur cluster transfer, and IscS-IscU complex
erpA	511145.b0156	COMPARTMENTS	GOCC:0005622	Intracellular
erpA	511145.b0156	COMPARTMENTS	GOCC:0005737	Cytoplasm
erpA	511145.b0156	COMPARTMENTS	GOCC:0005829	Cytosol
erpA	511145.b0156	COMPARTMENTS	GOCC:0110165	Cellular anatomical entity
erpA	511145.b0156	UniProt Keywords	KW-0408	Iron
erpA	511145.b0156	UniProt Keywords	KW-0411	Iron-sulfur
erpA	511145.b0156	UniProt Keywords	KW-0479	Metal-binding
erpA	511145.b0156	Pfam	PF01521	Iron-sulphur cluster biosynthesis
erpA	511145.b0156	InterPro	IPR000361	FeS cluster biogenesis
erpA	511145.b0156	InterPro	IPR016092	FeS cluster insertion protein
erpA	511145.b0156	InterPro	IPR017870	FeS cluster insertion, C-terminal, conserved site
erpA	511145.b0156	InterPro	IPR023063	FeS cluster insertion protein ErpA
erpA	511145.b0156	InterPro	IPR035903	HesB-like domain superfamily
fabZ	511145.b0180	GO Process	GO:0005975	Carbohydrate metabolic process
fabZ	511145.b0180	GO Process	GO:0006082	Organic acid metabolic process
fabZ	511145.b0180	GO Process	GO:0006629	Lipid metabolic process
fabZ	511145.b0180	GO Process	GO:0006631	Fatty acid metabolic process
fabZ	511145.b0180	GO Process	GO:0006633	Fatty acid biosynthetic process
fabZ	511145.b0180	GO Process	GO:0006643	Membrane lipid metabolic process
fabZ	511145.b0180	GO Process	GO:0006644	Phospholipid metabolic process
fabZ	511145.b0180	GO Process	GO:0006664	Glycolipid metabolic process
fabZ	511145.b0180	GO Process	GO:0006793	Phosphorus metabolic process
fabZ	511145.b0180	GO Process	GO:0006796	Phosphate-containing compound metabolic process
fabZ	511145.b0180	GO Process	GO:0008152	Metabolic process
fabZ	511145.b0180	GO Process	GO:0008610	Lipid biosynthetic process
fabZ	511145.b0180	GO Process	GO:0008654	Phospholipid biosynthetic process
fabZ	511145.b0180	GO Process	GO:0009058	Biosynthetic process
fabZ	511145.b0180	GO Process	GO:0009245	Lipid A biosynthetic process
fabZ	511145.b0180	GO Process	GO:0009247	Glycolipid biosynthetic process
fabZ	511145.b0180	GO Process	GO:0009311	Oligosaccharide metabolic process
fabZ	511145.b0180	GO Process	GO:0009312	Oligosaccharide biosynthetic process
fabZ	511145.b0180	GO Process	GO:0009987	Cellular process
fabZ	511145.b0180	GO Process	GO:0016051	Carbohydrate biosynthetic process
fabZ	511145.b0180	GO Process	GO:0016053	Organic acid biosynthetic process
fabZ	511145.b0180	GO Process	GO:0019637	Organophosphate metabolic process
fabZ	511145.b0180	GO Process	GO:0019752	Carboxylic acid metabolic process
fabZ	511145.b0180	GO Process	GO:0032787	Monocarboxylic acid metabolic process
fabZ	511145.b0180	GO Process	GO:0043436	Oxoacid metabolic process
fabZ	511145.b0180	GO Process	GO:0044237	Cellular metabolic process
fabZ	511145.b0180	GO Process	GO:0044238	Primary metabolic process
fabZ	511145.b0180	GO Process	GO:0044249	Cellular biosynthetic process
fabZ	511145.b0180	GO Process	GO:0044255	Cellular lipid metabolic process
fabZ	511145.b0180	GO Process	GO:0044281	Small molecule metabolic process
fabZ	511145.b0180	GO Process	GO:0044283	Small molecule biosynthetic process
fabZ	511145.b0180	GO Process	GO:0046394	Carboxylic acid biosynthetic process
fabZ	511145.b0180	GO Process	GO:0046467	Membrane lipid biosynthetic process
fabZ	511145.b0180	GO Process	GO:0046493	Lipid A metabolic process
fabZ	511145.b0180	GO Process	GO:0071704	Organic substance metabolic process
fabZ	511145.b0180	GO Process	GO:0072330	Monocarboxylic acid biosynthetic process
fabZ	511145.b0180	GO Process	GO:0090407	Organophosphate biosynthetic process
fabZ	511145.b0180	GO Process	GO:1901135	Carbohydrate derivative metabolic process
fabZ	511145.b0180	GO Process	GO:1901137	Carbohydrate derivative biosynthetic process
fabZ	511145.b0180	GO Process	GO:1901269	Lipooligosaccharide metabolic process
fabZ	511145.b0180	GO Process	GO:1901271	Lipooligosaccharide biosynthetic process
fabZ	511145.b0180	GO Process	GO:1901576	Organic substance biosynthetic process
fabZ	511145.b0180	GO Process	GO:1903509	Liposaccharide metabolic process
fabZ	511145.b0180	GO Function	GO:0003824	Catalytic activity
fabZ	511145.b0180	GO Function	GO:0004312	Fatty acid synthase activity
fabZ	511145.b0180	GO Function	GO:0004317	3-hydroxypalmitoyl-[acyl-carrier-protein] dehydratase activity
fabZ	511145.b0180	GO Function	GO:0005488	Binding
fabZ	511145.b0180	GO Function	GO:0005515	Protein binding
fabZ	511145.b0180	GO Function	GO:0008659	(3R)-hydroxymyristoyl-[acyl-carrier-protein] dehydratase activity
fabZ	511145.b0180	GO Function	GO:0008693	3-hydroxydecanoyl-[acyl-carrier-protein] dehydratase activity
fabZ	511145.b0180	GO Function	GO:0016740	Transferase activity
fabZ	511145.b0180	GO Function	GO:0016746	Acyltransferase activity
fabZ	511145.b0180	GO Function	GO:0016747	Acyltransferase activity, transferring groups other than amino-acyl groups
fabZ	511145.b0180	GO Function	GO:0016829	Lyase activity
fabZ	511145.b0180	GO Function	GO:0016835	Carbon-oxygen lyase activity
fabZ	511145.b0180	GO Function	GO:0016836	Hydro-lyase activity
fabZ	511145.b0180	GO Function	GO:0019171	3-hydroxyacyl-[acyl-carrier-protein] dehydratase activity
fabZ	511145.b0180	GO Function	GO:0042802	Identical protein binding
fabZ	511145.b0180	GO Function	GO:0047451	3-hydroxyoctanoyl-[acyl-carrier-protein] dehydratase activity
fabZ	511145.b0180	GO Component	GO:0005622	Intracellular anatomical structure
fabZ	511145.b0180	GO Component	GO:0005737	Cytoplasm
fabZ	511145.b0180	GO Component	GO:0110165	Cellular anatomical entity
fabZ	511145.b0180	STRING clusters	CL:2010	Mixed, incl. Carbon metabolism, and Fatty acid metabolic process
fabZ	511145.b0180	STRING clusters	CL:2270	Fatty acid metabolic process, and Phenylacetate catabolic process
fabZ	511145.b0180	STRING clusters	CL:2271	Fatty acid metabolic process, and Acetate transport
fabZ	511145.b0180	STRING clusters	CL:2272	Fatty acid metabolic process
fabZ	511145.b0180	STRING clusters	CL:2273	Fatty acid metabolism, and Propionate metabolic process
fabZ	511145.b0180	STRING clusters	CL:2320	Fatty acid biosynthesis
fabZ	511145.b0180	STRING clusters	CL:2323	Fatty acid synthase activity
fabZ	511145.b0180	KEGG	eco00061	Fatty acid biosynthesis
fabZ	511145.b0180	KEGG	eco00780	Biotin metabolism
fabZ	511145.b0180	KEGG	eco01100	Metabolic pathways
fabZ	511145.b0180	KEGG	eco01212	Fatty acid metabolism
fabZ	511145.b0180	COMPARTMENTS	GOCC:0005622	Intracellular
fabZ	511145.b0180	COMPARTMENTS	GOCC:0005737	Cytoplasm
fabZ	511145.b0180	COMPARTMENTS	GOCC:0110165	Cellular anatomical entity
fabZ	511145.b0180	UniProt Keywords	KW-0441	Lipid A biosynthesis
fabZ	511145.b0180	UniProt Keywords	KW-0443	Lipid metabolism
fabZ	511145.b0180	UniProt Keywords	KW-0444	Lipid biosynthesis
fabZ	511145.b0180	UniProt Keywords	KW-0456	Lyase
fabZ	511145.b0180	UniProt Keywords	KW-0963	Cytoplasm
fabZ	511145.b0180	Pfam	PF07977	FabA-like domain
fabZ	511145.b0180	InterPro	IPR010084	Beta-hydroxyacyl-(acyl-carrier-protein) dehydratase FabZ
fabZ	511145.b0180	InterPro	IPR013114	Beta-hydroxydecanoyl thiol ester dehydrase, FabA/FabZ
fabZ	511145.b0180	InterPro	IPR029069	HotDog domain superfamily
fadB	511145.b3846	GO Process	GO:0006082	Organic acid metabolic process
fadB	511145.b3846	GO Process	GO:0006629	Lipid metabolic process
fadB	511145.b3846	GO Process	GO:0006631	Fatty acid metabolic process
fadB	511145.b3846	GO Process	GO:0006635	Fatty acid beta-oxidation
fadB	511145.b3846	GO Process	GO:0008152	Metabolic process
fadB	511145.b3846	GO Process	GO:0009056	Catabolic process
fadB	511145.b3846	GO Process	GO:0009062	Fatty acid catabolic process
fadB	511145.b3846	GO Process	GO:0009987	Cellular process
fadB	511145.b3846	GO Process	GO:0016042	Lipid catabolic process
fadB	511145.b3846	GO Process	GO:0016054	Organic acid catabolic process
fadB	511145.b3846	GO Process	GO:0019395	Fatty acid oxidation
fadB	511145.b3846	GO Process	GO:0019752	Carboxylic acid metabolic process
fadB	511145.b3846	GO Process	GO:0030258	Lipid modification
fadB	511145.b3846	GO Process	GO:0032787	Monocarboxylic acid metabolic process
fadB	511145.b3846	GO Process	GO:0034440	Lipid oxidation
fadB	511145.b3846	GO Process	GO:0043436	Oxoacid metabolic process
fadB	511145.b3846	GO Process	GO:0044237	Cellular metabolic process
fadB	511145.b3846	GO Process	GO:0044238	Primary metabolic process
fadB	511145.b3846	GO Process	GO:0044242	Cellular lipid catabolic process
fadB	511145.b3846	GO Process	GO:0044248	Cellular catabolic process
fadB	511145.b3846	GO Process	GO:0044255	Cellular lipid metabolic process
fadB	511145.b3846	GO Process	GO:0044281	Small molecule metabolic process
fadB	511145.b3846	GO Process	GO:0044282	Small molecule catabolic process
fadB	511145.b3846	GO Process	GO:0046395	Carboxylic acid catabolic process
fadB	511145.b3846	GO Process	GO:0071704	Organic substance metabolic process
fadB	511145.b3846	GO Process	GO:0072329	Monocarboxylic acid catabolic process
fadB	511145.b3846	GO Process	GO:1901575	Organic substance catabolic process
fadB	511145.b3846	GO Function	GO:0000166	Nucleotide binding
fadB	511145.b3846	GO Function	GO:0003824	Catalytic activity
fadB	511145.b3846	GO Function	GO:0003857	3-hydroxyacyl-CoA dehydrogenase activity
fadB	511145.b3846	GO Function	GO:0004165	delta(3)-delta(2)-enoyl-CoA isomerase activity
fadB	511145.b3846	GO Function	GO:0004300	enoyl-CoA hydratase activity
fadB	511145.b3846	GO Function	GO:0005488	Binding
fadB	511145.b3846	GO Function	GO:0008692	3-hydroxybutyryl-CoA epimerase activity
fadB	511145.b3846	GO Function	GO:0016491	Oxidoreductase activity
fadB	511145.b3846	GO Function	GO:0016509	long-chain-3-hydroxyacyl-CoA dehydrogenase activity
fadB	511145.b3846	GO Function	GO:0016614	Oxidoreductase activity, acting on CH-OH group of donors
fadB	511145.b3846	GO Function	GO:0016616	Oxidoreductase activity, acting on the CH-OH group of donors, NAD or NADP as acceptor
fadB	511145.b3846	GO Function	GO:0016829	Lyase activity
fadB	511145.b3846	GO Function	GO:0016835	Carbon-oxygen lyase activity
fadB	511145.b3846	GO Function	GO:0016836	Hydro-lyase activity
fadB	511145.b3846	GO Function	GO:0016853	Isomerase activity
fadB	511145.b3846	GO Function	GO:0016854	Racemase and epimerase activity
fadB	511145.b3846	GO Function	GO:0016856	Racemase and epimerase activity, acting on hydroxy acids and derivatives
fadB	511145.b3846	GO Function	GO:0016860	Intramolecular oxidoreductase activity
fadB	511145.b3846	GO Function	GO:0016863	Intramolecular oxidoreductase activity, transposing C=C bonds
fadB	511145.b3846	GO Function	GO:0036094	Small molecule binding
fadB	511145.b3846	GO Function	GO:0043167	Ion binding
fadB	511145.b3846	GO Function	GO:0043168	Anion binding
fadB	511145.b3846	GO Function	GO:0051287	NAD binding
fadB	511145.b3846	GO Function	GO:0070403	NAD+ binding
fadB	511145.b3846	GO Function	GO:0097159	Organic cyclic compound binding
fadB	511145.b3846	GO Function	GO:1901265	Nucleoside phosphate binding
fadB	511145.b3846	GO Function	GO:1901363	Heterocyclic compound binding
fadB	511145.b3846	GO Component	GO:0032991	Protein-containing complex
fadB	511145.b3846	GO Component	GO:0036125	Fatty acid beta-oxidation multienzyme complex
fadB	511145.b3846	STRING clusters	CL:2010	Mixed, incl. Carbon metabolism, and Fatty acid metabolic process
fadB	511145.b3846	STRING clusters	CL:2270	Fatty acid metabolic process, and Phenylacetate catabolic process
fadB	511145.b3846	STRING clusters	CL:2271	Fatty acid metabolic process, and Acetate transport
fadB	511145.b3846	STRING clusters	CL:2272	Fatty acid metabolic process
fadB	511145.b3846	STRING clusters	CL:2273	Fatty acid metabolism, and Propionate metabolic process
fadB	511145.b3846	STRING clusters	CL:2275	Fatty acid beta-oxidation, and propionate-CoA ligase activity
fadB	511145.b3846	STRING clusters	CL:2277	Fatty acid beta-oxidation
fadB	511145.b3846	STRING clusters	CL:2280	Fatty acid beta-oxidation multienzyme complex
fadB	511145.b3846	KEGG	eco00071	Fatty acid degradation
fadB	511145.b3846	KEGG	eco00280	Valine, leucine and isoleucine degradation
fadB	511145.b3846	KEGG	eco00281	Geraniol degradation
fadB	511145.b3846	KEGG	eco00310	Lysine degradation
fadB	511145.b3846	KEGG	eco00362	Benzoate degradation
fadB	511145.b3846	KEGG	eco00380	Tryptophan metabolism
fadB	511145.b3846	KEGG	eco00410	beta-Alanine metabolism
fadB	511145.b3846	KEGG	eco00640	Propanoate metabolism
fadB	511145.b3846	KEGG	eco00650	Butanoate metabolism
fadB	511145.b3846	KEGG	eco00903	Limonene and pinene degradation
fadB	511145.b3846	KEGG	eco00930	Caprolactam degradation
fadB	511145.b3846	KEGG	eco01100	Metabolic pathways
fadB	511145.b3846	KEGG	eco01110	Biosynthesis of secondary metabolites
fadB	511145.b3846	KEGG	eco01120	Microbial metabolism in diverse environments
fadB	511145.b3846	KEGG	eco01200	Carbon metabolism
fadB	511145.b3846	KEGG	eco01212	Fatty acid metabolism
fadB	511145.b3846	COMPARTMENTS	GOCC:0032991	Protein-containing complex
fadB	511145.b3846	COMPARTMENTS	GOCC:0036125	Fatty acid beta-oxidation multienzyme complex
fadB	511145.b3846	UniProt Keywords	KW-0276	Fatty acid metabolism
fadB	511145.b3846	UniProt Keywords	KW-0413	Isomerase
fadB	511145.b3846	UniProt Keywords	KW-0442	Lipid degradation
fadB	511145.b3846	UniProt Keywords	KW-0443	Lipid metabolism
fadB	511145.b3846	UniProt Keywords	KW-0456	Lyase
fadB	511145.b3846	UniProt Keywords	KW-0511	Multifunctional enzyme
fadB	511145.b3846	UniProt Keywords	KW-0520	NAD
fadB	511145.b3846	UniProt Keywords	KW-0560	Oxidoreductase
fadB	511145.b3846	Pfam	PF00725	3-hydroxyacyl-CoA dehydrogenase, C-terminal domain
fadB	511145.b3846	Pfam	PF02737	3-hydroxyacyl-CoA dehydrogenase, NAD binding domain
fadB	511145.b3846	Pfam	PF16113	Enoyl-CoA hydratase/isomerase
fadB	511145.b3846	InterPro	IPR001753	Enoyl-CoA hydratase/isomerase
fadB	511145.b3846	InterPro	IPR006108	3-hydroxyacyl-CoA dehydrogenase, C-terminal
fadB	511145.b3846	InterPro	IPR006176	3-hydroxyacyl-CoA dehydrogenase, NAD binding
fadB	511145.b3846	InterPro	IPR006180	3-hydroxyacyl-CoA dehydrogenase, conserved site
fadB	511145.b3846	InterPro	IPR008927	6-phosphogluconate dehydrogenase-like, C-terminal domain superfamily
fadB	511145.b3846	InterPro	IPR012799	Fatty oxidation complex, alpha subunit FadB
fadB	511145.b3846	InterPro	IPR018376	Enoyl-CoA hydratase/isomerase, conserved site
fadB	511145.b3846	InterPro	IPR029045	ClpP/crotonase-like domain superfamily
fadB	511145.b3846	InterPro	IPR036291	NAD(P)-binding domain superfamily
fadJ	511145.b2341	GO Process	GO:0006082	Organic acid metabolic process
fadJ	511145.b2341	GO Process	GO:0006629	Lipid metabolic process
fadJ	511145.b2341	GO Process	GO:0006631	Fatty acid metabolic process
fadJ	511145.b2341	GO Process	GO:0006635	Fatty acid beta-oxidation
fadJ	511145.b2341	GO Process	GO:0008152	Metabolic process
fadJ	511145.b2341	GO Process	GO:0009056	Catabolic process
fadJ	511145.b2341	GO Process	GO:0009062	Fatty acid catabolic process
fadJ	511145.b2341	GO Process	GO:0009987	Cellular process
fadJ	511145.b2341	GO Process	GO:0016042	Lipid catabolic process
fadJ	511145.b2341	GO Process	GO:0016054	Organic acid catabolic process
fadJ	511145.b2341	GO Process	GO:0019395	Fatty acid oxidation
fadJ	511145.b2341	GO Process	GO:0019752	Carboxylic acid metabolic process
fadJ	511145.b2341	GO Process	GO:0030258	Lipid modification
fadJ	511145.b2341	GO Process	GO:0032787	Monocarboxylic acid metabolic process
fadJ	511145.b2341	GO Process	GO:0034440	Lipid oxidation
fadJ	511145.b2341	GO Process	GO:0043436	Oxoacid metabolic process
fadJ	511145.b2341	GO Process	GO:0044237	Cellular metabolic process
fadJ	511145.b2341	GO Process	GO:0044238	Primary metabolic process
fadJ	511145.b2341	GO Process	GO:0044242	Cellular lipid catabolic process
fadJ	511145.b2341	GO Process	GO:0044248	Cellular catabolic process
fadJ	511145.b2341	GO Process	GO:0044255	Cellular lipid metabolic process
fadJ	511145.b2341	GO Process	GO:0044281	Small molecule metabolic process
fadJ	511145.b2341	GO Process	GO:0044282	Small molecule catabolic process
fadJ	511145.b2341	GO Process	GO:0046395	Carboxylic acid catabolic process
fadJ	511145.b2341	GO Process	GO:0071704	Organic substance metabolic process
fadJ	511145.b2341	GO Process	GO:0072329	Monocarboxylic acid catabolic process
fadJ	511145.b2341	GO Process	GO:1901575	Organic substance catabolic process
fadJ	511145.b2341	GO Function	GO:0000166	Nucleotide binding
fadJ	511145.b2341	GO Function	GO:0003824	Catalytic activity
fadJ	511145.b2341	GO Function	GO:0003857	3-hydroxyacyl-CoA dehydrogenase activity
fadJ	511145.b2341	GO Function	GO:0004300	enoyl-CoA hydratase activity
fadJ	511145.b2341	GO Function	GO:0005488	Binding
fadJ	511145.b2341	GO Function	GO:0008692	3-hydroxybutyryl-CoA epimerase activity
fadJ	511145.b2341	GO Function	GO:0016491	Oxidoreductase activity
fadJ	511145.b2341	GO Function	GO:0016509	long-chain-3-hydroxyacyl-CoA dehydrogenase activity
fadJ	511145.b2341	GO Function	GO:0016614	Oxidoreductase activity, acting on CH-OH group of donors
fadJ	511145.b2341	GO Function	GO:0016616	Oxidoreductase activity, acting on the CH-OH group of donors, NAD or NADP as acceptor
fadJ	511145.b2341	GO Function	GO:0016829	Lyase activity
fadJ	511145.b2341	GO Function	GO:0016835	Carbon-oxygen lyase activity
fadJ	511145.b2341	GO Function	GO:0016836	Hydro-lyase activity
fadJ	511145.b2341	GO Function	GO:0016853	Isomerase activity
fadJ	511145.b2341	GO Function	GO:0016854	Racemase and epimerase activity
fadJ	511145.b2341	GO Function	GO:0016856	Racemase and epimerase activity, acting on hydroxy acids and derivatives
fadJ	511145.b2341	GO Function	GO:0036094	Small molecule binding
fadJ	511145.b2341	GO Function	GO:0043167	Ion binding
fadJ	511145.b2341	GO Function	GO:0043168	Anion binding
fadJ	511145.b2341	GO Function	GO:0051287	NAD binding
fadJ	511145.b2341	GO Function	GO:0070403	NAD+ binding
fadJ	511145.b2341	GO Function	GO:0097159	Organic cyclic compound binding
fadJ	511145.b2341	GO Function	GO:1901265	Nucleoside phosphate binding
fadJ	511145.b2341	GO Function	GO:1901363	Heterocyclic compound binding
fadJ	511145.b2341	GO Component	GO:0005622	Intracellular anatomical structure
fadJ	511145.b2341	GO Component	GO:0005737	Cytoplasm
fadJ	511145.b2341	GO Component	GO:0032991	Protein-containing complex
fadJ	511145.b2341	GO Component	GO:0036125	Fatty acid beta-oxidation multienzyme complex
fadJ	511145.b2341	GO Component	GO:0110165	Cellular anatomical entity
fadJ	511145.b2341	STRING clusters	CL:2010	Mixed, incl. Carbon metabolism, and Fatty acid metabolic process
fadJ	511145.b2341	STRING clusters	CL:2270	Fatty acid metabolic process, and Phenylacetate catabolic process
fadJ	511145.b2341	STRING clusters	CL:2271	Fatty acid metabolic process, and Acetate transport
fadJ	511145.b2341	STRING clusters	CL:2272	Fatty acid metabolic process
fadJ	511145.b2341	STRING clusters	CL:2273	Fatty acid metabolism, and Propionate metabolic process
fadJ	511145.b2341	STRING clusters	CL:2275	Fatty acid beta-oxidation, and propionate-CoA ligase activity
fadJ	511145.b2341	STRING clusters	CL:2277	Fatty acid beta-oxidation
fadJ	511145.b2341	STRING clusters	CL:2280	Fatty acid beta-oxidation multienzyme complex
fadJ	511145.b2341	KEGG	eco00071	Fatty acid degradation
fadJ	511145.b2341	KEGG	eco00280	Valine, leucine and isoleucine degradation
fadJ	511145.b2341	KEGG	eco00281	Geraniol degradation
fadJ	511145.b2341	KEGG	eco00310	Lysine degradation
fadJ	511145.b2341	KEGG	eco00362	Benzoate degradation
fadJ	511145.b2341	KEGG	eco00380	Tryptophan metabolism
fadJ	511145.b2341	KEGG	eco00410	beta-Alanine metabolism
fadJ	511145.b2341	KEGG	eco00640	Propanoate metabolism
fadJ	511145.b2341	KEGG	eco00650	Butanoate metabolism
fadJ	511145.b2341	KEGG	eco00903	Limonene and pinene degradation
fadJ	511145.b2341	KEGG	eco00930	Caprolactam degradation
fadJ	511145.b2341	KEGG	eco01100	Metabolic pathways
fadJ	511145.b2341	KEGG	eco01110	Biosynthesis of secondary metabolites
fadJ	511145.b2341	KEGG	eco01120	Microbial metabolism in diverse environments
fadJ	511145.b2341	KEGG	eco01200	Carbon metabolism
fadJ	511145.b2341	KEGG	eco01212	Fatty acid metabolism
fadJ	511145.b2341	COMPARTMENTS	GOCC:0032991	Protein-containing complex
fadJ	511145.b2341	COMPARTMENTS	GOCC:0036125	Fatty acid beta-oxidation multienzyme complex
fadJ	511145.b2341	UniProt Keywords	KW-0276	Fatty acid metabolism
fadJ	511145.b2341	UniProt Keywords	KW-0413	Isomerase
fadJ	511145.b2341	UniProt Keywords	KW-0442	Lipid degradation
fadJ	511145.b2341	UniProt Keywords	KW-0443	Lipid metabolism
fadJ	511145.b2341	UniProt Keywords	KW-0456	Lyase
fadJ	511145.b2341	UniProt Keywords	KW-0511	Multifunctional enzyme
fadJ	511145.b2341	UniProt Keywords	KW-0520	NAD
fadJ	511145.b2341	UniProt Keywords	KW-0560	Oxidoreductase
fadJ	511145.b2341	UniProt Keywords	KW-0963	Cytoplasm
fadJ	511145.b2341	Pfam	PF00725	3-hydroxyacyl-CoA dehydrogenase, C-terminal domain
fadJ	511145.b2341	Pfam	PF02737	3-hydroxyacyl-CoA dehydrogenase, NAD binding domain
fadJ	511145.b2341	Pfam	PF16113	Enoyl-CoA hydratase/isomerase
fadJ	511145.b2341	InterPro	IPR001753	Enoyl-CoA hydratase/isomerase
fadJ	511145.b2341	InterPro	IPR006108	3-hydroxyacyl-CoA dehydrogenase, C-terminal
fadJ	511145.b2341	InterPro	IPR006176	3-hydroxyacyl-CoA dehydrogenase, NAD binding
fadJ	511145.b2341	InterPro	IPR006180	3-hydroxyacyl-CoA dehydrogenase, conserved site
fadJ	511145.b2341	InterPro	IPR008927	6-phosphogluconate dehydrogenase-like, C-terminal domain superfamily
fadJ	511145.b2341	InterPro	IPR012802	Fatty oxidation complex, alpha subunit FadJ
fadJ	511145.b2341	InterPro	IPR029045	ClpP/crotonase-like domain superfamily
fadJ	511145.b2341	InterPro	IPR036291	NAD(P)-binding domain superfamily
fau	511145.b2912	GO Process	GO:0006575	Cellular modified amino acid metabolic process
fau	511145.b2912	GO Process	GO:0006725	Cellular aromatic compound metabolic process
fau	511145.b2912	GO Process	GO:0006730	One-carbon metabolic process
fau	511145.b2912	GO Process	GO:0006760	Folic acid-containing compound metabolic process
fau	511145.b2912	GO Process	GO:0006807	Nitrogen compound metabolic process
fau	511145.b2912	GO Process	GO:0008152	Metabolic process
fau	511145.b2912	GO Process	GO:0009058	Biosynthetic process
fau	511145.b2912	GO Process	GO:0009396	Folic acid-containing compound biosynthetic process
fau	511145.b2912	GO Process	GO:0009987	Cellular process
fau	511145.b2912	GO Process	GO:0018130	Heterocycle biosynthetic process
fau	511145.b2912	GO Process	GO:0019438	Aromatic compound biosynthetic process
fau	511145.b2912	GO Process	GO:0022611	Dormancy process
fau	511145.b2912	GO Process	GO:0032502	Developmental process
fau	511145.b2912	GO Process	GO:0034641	Cellular nitrogen compound metabolic process
fau	511145.b2912	GO Process	GO:0035999	Tetrahydrofolate interconversion
fau	511145.b2912	GO Process	GO:0042398	Cellular modified amino acid biosynthetic process
fau	511145.b2912	GO Process	GO:0042558	Pteridine-containing compound metabolic process
fau	511145.b2912	GO Process	GO:0042559	Pteridine-containing compound biosynthetic process
fau	511145.b2912	GO Process	GO:0044237	Cellular metabolic process
fau	511145.b2912	GO Process	GO:0044249	Cellular biosynthetic process
fau	511145.b2912	GO Process	GO:0044271	Cellular nitrogen compound biosynthetic process
fau	511145.b2912	GO Process	GO:0044281	Small molecule metabolic process
fau	511145.b2912	GO Process	GO:0046483	Heterocycle metabolic process
fau	511145.b2912	GO Process	GO:0046653	Tetrahydrofolate metabolic process
fau	511145.b2912	GO Process	GO:0071704	Organic substance metabolic process
fau	511145.b2912	GO Process	GO:1901360	Organic cyclic compound metabolic process
fau	511145.b2912	GO Process	GO:1901362	Organic cyclic compound biosynthetic process
fau	511145.b2912	GO Process	GO:1901564	Organonitrogen compound metabolic process
fau	511145.b2912	GO Process	GO:1901566	Organonitrogen compound biosynthetic process
fau	511145.b2912	GO Process	GO:1901576	Organic substance biosynthetic process
fau	511145.b2912	GO Function	GO:0000166	Nucleotide binding
fau	511145.b2912	GO Function	GO:0003824	Catalytic activity
fau	511145.b2912	GO Function	GO:0005488	Binding
fau	511145.b2912	GO Function	GO:0005524	ATP binding
fau	511145.b2912	GO Function	GO:0016874	Ligase activity
fau	511145.b2912	GO Function	GO:0016879	Ligase activity, forming carbon-nitrogen bonds
fau	511145.b2912	GO Function	GO:0016882	Cyclo-ligase activity
fau	511145.b2912	GO Function	GO:0017076	Purine nucleotide binding
fau	511145.b2912	GO Function	GO:0030272	5-formyltetrahydrofolate cyclo-ligase activity
fau	511145.b2912	GO Function	GO:0030554	Adenyl nucleotide binding
fau	511145.b2912	GO Function	GO:0032553	Ribonucleotide binding
fau	511145.b2912	GO Function	GO:0032555	Purine ribonucleotide binding
fau	511145.b2912	GO Function	GO:0032559	Adenyl ribonucleotide binding
fau	511145.b2912	GO Function	GO:0035639	Purine ribonucleoside triphosphate binding
fau	511145.b2912	GO Function	GO:0036094	Small molecule binding
fau	511145.b2912	GO Function	GO:0043167	Ion binding
fau	511145.b2912	GO Function	GO:0043168	Anion binding
fau	511145.b2912	GO Function	GO:0097159	Organic cyclic compound binding
fau	511145.b2912	GO Function	GO:0097367	Carbohydrate derivative binding
fau	511145.b2912	GO Function	GO:1901265	Nucleoside phosphate binding
fau	511145.b2912	GO Function	GO:1901363	Heterocyclic compound binding
fau	511145.b2912	STRING clusters	CL:1684	Mixed, incl. Nucleoside monophosphate biosynthetic process, and Pyrimidine metabolism
fau	511145.b2912	STRING clusters	CL:1685	Nucleoside monophosphate biosynthetic process, and Nucleobase transport
fau	511145.b2912	STRING clusters	CL:1686	Ribonucleoside monophosphate biosynthetic process, and Nucleobase transport
fau	511145.b2912	STRING clusters	CL:1758	One-carbon metabolism, and Folic acid-containing compound biosynthetic process
fau	511145.b2912	KEGG	eco00670	One carbon pool by folate
fau	511145.b2912	KEGG	eco01100	Metabolic pathways
fau	511145.b2912	UniProt Keywords	KW-0067	ATP-binding
fau	511145.b2912	UniProt Keywords	KW-0436	Ligase
fau	511145.b2912	UniProt Keywords	KW-0547	Nucleotide-binding
fau	511145.b2912	Pfam	PF01812	5-formyltetrahydrofolate cyclo-ligase family
fau	511145.b2912	InterPro	IPR002698	5-formyltetrahydrofolate cyclo-ligase
fau	511145.b2912	InterPro	IPR024185	5-formyltetrahydrofolate cyclo-ligase-like domain superfamily
fau	511145.b2912	InterPro	IPR037171	NagB/RpiA transferase-like
fdx	511145.b2525	GO Process	GO:0006091	Generation of precursor metabolites and energy
fdx	511145.b2525	GO Process	GO:0006790	Sulfur compound metabolic process
fdx	511145.b2525	GO Process	GO:0008152	Metabolic process
fdx	511145.b2525	GO Process	GO:0009987	Cellular process
fdx	511145.b2525	GO Process	GO:0016043	Cellular component organization
fdx	511145.b2525	GO Process	GO:0016226	Iron-sulfur cluster assembly
fdx	511145.b2525	GO Process	GO:0022607	Cellular component assembly
fdx	511145.b2525	GO Process	GO:0022900	Electron transport chain
fdx	511145.b2525	GO Process	GO:0031163	Metallo-sulfur cluster assembly
fdx	511145.b2525	GO Process	GO:0044085	Cellular component biogenesis
fdx	511145.b2525	GO Process	GO:0044237	Cellular metabolic process
fdx	511145.b2525	GO Process	GO:0071840	Cellular component organization or biogenesis
fdx	511145.b2525	GO Process	GO:0140647	P450-containing electron transport chain
fdx	511145.b2525	GO Function	GO:0003824	Catalytic activity
fdx	511145.b2525	GO Function	GO:0005488	Binding
fdx	511145.b2525	GO Function	GO:0009055	Electron transfer activity
fdx	511145.b2525	GO Function	GO:0016491	Oxidoreductase activity
fdx	511145.b2525	GO Function	GO:0043167	Ion binding
fdx	511145.b2525	GO Function	GO:0043169	Cation binding
fdx	511145.b2525	GO Function	GO:0046872	Metal ion binding
fdx	511145.b2525	GO Function	GO:0051536	Iron-sulfur cluster binding
fdx	511145.b2525	GO Function	GO:0051537	2 iron, 2 sulfur cluster binding
fdx	511145.b2525	GO Function	GO:0051540	Metal cluster binding
fdx	511145.b2525	GO Component	GO:0005622	Intracellular anatomical structure
fdx	511145.b2525	GO Component	GO:0005737	Cytoplasm
fdx	511145.b2525	GO Component	GO:0005829	Cytosol
fdx	511145.b2525	GO Component	GO:0110165	Cellular anatomical entity
fdx	511145.b2525	STRING clusters	CL:1242	Mixed, incl. Iron-sulfur cluster assembly, and Antioxidant activity
fdx	511145.b2525	STRING clusters	CL:1243	Mixed, incl. Iron-sulfur cluster assembly, and Antioxidant activity
fdx	511145.b2525	STRING clusters	CL:1327	Iron-sulfur cluster assembly, and tRNA thio-modification
fdx	511145.b2525	STRING clusters	CL:1328	Iron-sulfur cluster assembly
fdx	511145.b2525	STRING clusters	CL:1329	Protein maturation by iron-sulfur cluster transfer, and IscS-IscU complex
fdx	511145.b2525	STRING clusters	CL:1333	Iron-sulfur cluster transfer complex, and IscS-IscU complex
fdx	511145.b2525	COMPARTMENTS	GOCC:0005622	Intracellular
fdx	511145.b2525	COMPARTMENTS	GOCC:0005737	Cytoplasm
fdx	511145.b2525	COMPARTMENTS	GOCC:0005829	Cytosol
fdx	511145.b2525	COMPARTMENTS	GOCC:0110165	Cellular anatomical entity
fdx	511145.b2525	UniProt Keywords	KW-0001	2Fe-2S
fdx	511145.b2525	UniProt Keywords	KW-0249	Electron transport
fdx	511145.b2525	UniProt Keywords	KW-0408	Iron
fdx	511145.b2525	UniProt Keywords	KW-0411	Iron-sulfur
fdx	511145.b2525	UniProt Keywords	KW-0479	Metal-binding
fdx	511145.b2525	UniProt Keywords	KW-0813	Transport
fdx	511145.b2525	Pfam	PF00111	2Fe-2S iron-sulfur cluster binding domain
fdx	511145.b2525	InterPro	IPR001041	2Fe-2S ferredoxin-type iron-sulfur binding domain
fdx	511145.b2525	InterPro	IPR001055	Adrenodoxin
fdx	511145.b2525	InterPro	IPR011536	Ferredoxin 2Fe-2S type, proteobacteria
fdx	511145.b2525	InterPro	IPR012675	Beta-grasp domain superfamily
fdx	511145.b2525	InterPro	IPR018298	Adrenodoxin, iron-sulphur binding site
fdx	511145.b2525	InterPro	IPR036010	2Fe-2S ferredoxin-like superfamily
fis	511145.b3261	GO Process	GO:0000018	Regulation of DNA recombination
fis	511145.b3261	GO Process	GO:0006139	Nucleobase-containing compound metabolic process
fis	511145.b3261	GO Process	GO:0006351	Transcription, DNA-templated
fis	511145.b3261	GO Process	GO:0006355	Regulation of transcription, DNA-templated
fis	511145.b3261	GO Process	GO:0006725	Cellular aromatic compound metabolic process
fis	511145.b3261	GO Process	GO:0006807	Nitrogen compound metabolic process
fis	511145.b3261	GO Process	GO:0008152	Metabolic process
fis	511145.b3261	GO Process	GO:0009058	Biosynthetic process
fis	511145.b3261	GO Process	GO:0009059	Macromolecule biosynthetic process
fis	511145.b3261	GO Process	GO:0009314	Response to radiation
fis	511145.b3261	GO Process	GO:0009628	Response to abiotic stimulus
fis	511145.b3261	GO Process	GO:0009889	Regulation of biosynthetic process
fis	511145.b3261	GO Process	GO:0009890	Negative regulation of biosynthetic process
fis	511145.b3261	GO Process	GO:0009891	Positive regulation of biosynthetic process
fis	511145.b3261	GO Process	GO:0009892	Negative regulation of metabolic process
fis	511145.b3261	GO Process	GO:0009893	Positive regulation of metabolic process
fis	511145.b3261	GO Process	GO:0009987	Cellular process
fis	511145.b3261	GO Process	GO:0010467	Gene expression
fis	511145.b3261	GO Process	GO:0010468	Regulation of gene expression
fis	511145.b3261	GO Process	GO:0010556	Regulation of macromolecule biosynthetic process
fis	511145.b3261	GO Process	GO:0010557	Positive regulation of macromolecule biosynthetic process
fis	511145.b3261	GO Process	GO:0010558	Negative regulation of macromolecule biosynthetic process
fis	511145.b3261	GO Process	GO:0010604	Positive regulation of macromolecule metabolic process
fis	511145.b3261	GO Process	GO:0010605	Negative regulation of macromolecule metabolic process
fis	511145.b3261	GO Process	GO:0016032	Viral process
fis	511145.b3261	GO Process	GO:0016070	RNA metabolic process
fis	511145.b3261	GO Process	GO:0018130	Heterocycle biosynthetic process
fis	511145.b3261	GO Process	GO:0019042	Viral latency
fis	511145.b3261	GO Process	GO:0019045	Latent virus replication
fis	511145.b3261	GO Process	GO:0019046	Release from viral latency
fis	511145.b3261	GO Process	GO:0019219	Regulation of nucleobase-containing compound metabolic process
fis	511145.b3261	GO Process	GO:0019222	Regulation of metabolic process
fis	511145.b3261	GO Process	GO:0019438	Aromatic compound biosynthetic process
fis	511145.b3261	GO Process	GO:0031323	Regulation of cellular metabolic process
fis	511145.b3261	GO Process	GO:0031324	Negative regulation of cellular metabolic process
fis	511145.b3261	GO Process	GO:0031325	Positive regulation of cellular metabolic process
fis	511145.b3261	GO Process	GO:0031326	Regulation of cellular biosynthetic process
fis	511145.b3261	GO Process	GO:0031327	Negative regulation of cellular biosynthetic process
fis	511145.b3261	GO Process	GO:0031328	Positive regulation of cellular biosynthetic process
fis	511145.b3261	GO Process	GO:0032359	Provirus excision
fis	511145.b3261	GO Process	GO:0032774	RNA biosynthetic process
fis	511145.b3261	GO Process	GO:0034641	Cellular nitrogen compound metabolic process
fis	511145.b3261	GO Process	GO:0034654	Nucleobase-containing compound biosynthetic process
fis	511145.b3261	GO Process	GO:0043170	Macromolecule metabolic process
fis	511145.b3261	GO Process	GO:0044237	Cellular metabolic process
fis	511145.b3261	GO Process	GO:0044238	Primary metabolic process
fis	511145.b3261	GO Process	GO:0044249	Cellular biosynthetic process
fis	511145.b3261	GO Process	GO:0044271	Cellular nitrogen compound biosynthetic process
fis	511145.b3261	GO Process	GO:0045892	Negative regulation of transcription, DNA-templated
fis	511145.b3261	GO Process	GO:0045893	Positive regulation of transcription, DNA-templated
fis	511145.b3261	GO Process	GO:0045911	Positive regulation of DNA recombination
fis	511145.b3261	GO Process	GO:0045934	Negative regulation of nucleobase-containing compound metabolic process
fis	511145.b3261	GO Process	GO:0045935	Positive regulation of nucleobase-containing compound metabolic process
fis	511145.b3261	GO Process	GO:0046483	Heterocycle metabolic process
fis	511145.b3261	GO Process	GO:0048518	Positive regulation of biological process
fis	511145.b3261	GO Process	GO:0048519	Negative regulation of biological process
fis	511145.b3261	GO Process	GO:0048522	Positive regulation of cellular process
fis	511145.b3261	GO Process	GO:0048523	Negative regulation of cellular process
fis	511145.b3261	GO Process	GO:0050789	Regulation of biological process
fis	511145.b3261	GO Process	GO:0050794	Regulation of cellular process
fis	511145.b3261	GO Process	GO:0050896	Response to stimulus
fis	511145.b3261	GO Process	GO:0051052	Regulation of DNA metabolic process
fis	511145.b3261	GO Process	GO:0051054	Positive regulation of DNA metabolic process
fis	511145.b3261	GO Process	GO:0051171	Regulation of nitrogen compound metabolic process
fis	511145.b3261	GO Process	GO:0051172	Negative regulation of nitrogen compound metabolic process
fis	511145.b3261	GO Process	GO:0051173	Positive regulation of nitrogen compound metabolic process
fis	511145.b3261	GO Process	GO:0051252	Regulation of RNA metabolic process
fis	511145.b3261	GO Process	GO:0051253	Negative regulation of RNA metabolic process
fis	511145.b3261	GO Process	GO:0051254	Positive regulation of RNA metabolic process
fis	511145.b3261	GO Process	GO:0060255	Regulation of macromolecule metabolic process
fis	511145.b3261	GO Process	GO:0065007	Biological regulation
fis	511145.b3261	GO Process	GO:0071704	Organic substance metabolic process
fis	511145.b3261	GO Process	GO:0080090	Regulation of primary metabolic process
fis	511145.b3261	GO Process	GO:0090304	Nucleic acid metabolic process
fis	511145.b3261	GO Process	GO:0097659	Nucleic acid-templated transcription
fis	511145.b3261	GO Process	GO:1901360	Organic cyclic compound metabolic process
fis	511145.b3261	GO Process	GO:1901362	Organic cyclic compound biosynthetic process
fis	511145.b3261	GO Process	GO:1901576	Organic substance biosynthetic process
fis	511145.b3261	GO Process	GO:1902679	Negative regulation of RNA biosynthetic process
fis	511145.b3261	GO Process	GO:1902680	Positive regulation of RNA biosynthetic process
fis	511145.b3261	GO Process	GO:1903506	Regulation of nucleic acid-templated transcription
fis	511145.b3261	GO Process	GO:1903507	Negative regulation of nucleic acid-templated transcription
fis	511145.b3261	GO Process	GO:1903508	Positive regulation of nucleic acid-templated transcription
fis	511145.b3261	GO Process	GO:2001141	Regulation of RNA biosynthetic process
fis	511145.b3261	GO Function	GO:0000976	Transcription cis-regulatory region binding
fis	511145.b3261	GO Function	GO:0001046	Core promoter sequence-specific DNA binding
fis	511145.b3261	GO Function	GO:0001067	Transcription regulatory region nucleic acid binding
fis	511145.b3261	GO Function	GO:0001216	DNA-binding transcription activator activity
fis	511145.b3261	GO Function	GO:0001217	DNA-binding transcription repressor activity
fis	511145.b3261	GO Function	GO:0003676	Nucleic acid binding
fis	511145.b3261	GO Function	GO:0003677	DNA binding
fis	511145.b3261	GO Function	GO:0003690	Double-stranded DNA binding
fis	511145.b3261	GO Function	GO:0003700	DNA-binding transcription factor activity
fis	511145.b3261	GO Function	GO:0005488	Binding
fis	511145.b3261	GO Function	GO:0005515	Protein binding
fis	511145.b3261	GO Function	GO:0008301	DNA binding, bending
fis	511145.b3261	GO Function	GO:0042802	Identical protein binding
fis	511145.b3261	GO Function	GO:0042803	Protein homodimerization activity
fis	511145.b3261	GO Function	GO:0043565	Sequence-specific DNA binding
fis	511145.b3261	GO Function	GO:0044374	Sequence-specific DNA binding, bending
fis	511145.b3261	GO Function	GO:0046983	Protein dimerization activity
fis	511145.b3261	GO Function	GO:0097159	Organic cyclic compound binding
fis	511145.b3261	GO Function	GO:0140110	Transcription regulator activity
fis	511145.b3261	GO Function	GO:1901363	Heterocyclic compound binding
fis	511145.b3261	GO Function	GO:1990837	Sequence-specific double-stranded DNA binding
fis	511145.b3261	GO Component	GO:0000785	Chromatin
fis	511145.b3261	GO Component	GO:0000786	Nucleosome
fis	511145.b3261	GO Component	GO:0005622	Intracellular anatomical structure
fis	511145.b3261	GO Component	GO:0005694	Chromosome
fis	511145.b3261	GO Component	GO:0005737	Cytoplasm
fis	511145.b3261	GO Component	GO:0005829	Cytosol
fis	511145.b3261	GO Component	GO:0009295	Nucleoid
fis	511145.b3261	GO Component	GO:0031421	Invertasome
fis	511145.b3261	GO Component	GO:0032991	Protein-containing complex
fis	511145.b3261	GO Component	GO:0032993	protein-DNA complex
fis	511145.b3261	GO Component	GO:0043226	Organelle
fis	511145.b3261	GO Component	GO:0043228	Non-membrane-bounded organelle
fis	511145.b3261	GO Component	GO:0043229	Intracellular organelle
fis	511145.b3261	GO Component	GO:0043232	Intracellular non-membrane-bounded organelle
fis	511145.b3261	GO Component	GO:0044815	DNA packaging complex
fis	511145.b3261	GO Component	GO:0110165	Cellular anatomical entity
fis	511145.b3261	GO Component	GO:0140535	Intracellular protein-containing complex
fis	511145.b3261	STRING clusters	CL:1208	Mostly uncharacterized, incl. N-methyltransferase activity, and Invertasome
fis	511145.b3261	STRING clusters	CL:1211	Mixed, incl. Invertasome, and Sodium ion export across plasma membrane
fis	511145.b3261	COMPARTMENTS	GOCC:0000785	Chromatin
fis	511145.b3261	COMPARTMENTS	GOCC:0000786	Nucleosome
fis	511145.b3261	COMPARTMENTS	GOCC:0005622	Intracellular
fis	511145.b3261	COMPARTMENTS	GOCC:0005694	Chromosome
fis	511145.b3261	COMPARTMENTS	GOCC:0005737	Cytoplasm
fis	511145.b3261	COMPARTMENTS	GOCC:0005829	Cytosol
fis	511145.b3261	COMPARTMENTS	GOCC:0031421	Invertasome
fis	511145.b3261	COMPARTMENTS	GOCC:0032991	Protein-containing complex
fis	511145.b3261	COMPARTMENTS	GOCC:0032993	protein-DNA complex
fis	511145.b3261	COMPARTMENTS	GOCC:0043226	Organelle
fis	511145.b3261	COMPARTMENTS	GOCC:0043228	Non-membrane-bounded organelle
fis	511145.b3261	COMPARTMENTS	GOCC:0043229	Intracellular organelle
fis	511145.b3261	COMPARTMENTS	GOCC:0043232	Intracellular non-membrane-bounded organelle
fis	511145.b3261	COMPARTMENTS	GOCC:0044815	DNA packaging complex
fis	511145.b3261	COMPARTMENTS	GOCC:0110165	Cellular anatomical entity
fis	511145.b3261	UniProt Keywords	KW-0010	Activator
fis	511145.b3261	UniProt Keywords	KW-0238	DNA-binding
fis	511145.b3261	UniProt Keywords	KW-0804	Transcription
fis	511145.b3261	UniProt Keywords	KW-0805	Transcription regulation
fis	511145.b3261	UniProt Keywords	KW-0963	Cytoplasm
fis	511145.b3261	Pfam	PF02954	Bacterial regulatory protein, Fis family
fis	511145.b3261	InterPro	IPR002197	DNA binding HTH domain, Fis-type
fis	511145.b3261	InterPro	IPR005412	DNA-binding protein Fis
fis	511145.b3261	InterPro	IPR009057	Homeobox-like domain superfamily
fmt	511145.b3288	GO Process	GO:0006139	Nucleobase-containing compound metabolic process
fmt	511145.b3288	GO Process	GO:0006396	RNA processing
fmt	511145.b3288	GO Process	GO:0006399	tRNA metabolic process
fmt	511145.b3288	GO Process	GO:0006400	tRNA modification
fmt	511145.b3288	GO Process	GO:0006412	Translation
fmt	511145.b3288	GO Process	GO:0006413	Translational initiation
fmt	511145.b3288	GO Process	GO:0006518	Peptide metabolic process
fmt	511145.b3288	GO Process	GO:0006725	Cellular aromatic compound metabolic process
fmt	511145.b3288	GO Process	GO:0006807	Nitrogen compound metabolic process
fmt	511145.b3288	GO Process	GO:0008033	tRNA processing
fmt	511145.b3288	GO Process	GO:0008152	Metabolic process
fmt	511145.b3288	GO Process	GO:0009058	Biosynthetic process
fmt	511145.b3288	GO Process	GO:0009059	Macromolecule biosynthetic process
fmt	511145.b3288	GO Process	GO:0009451	RNA modification
fmt	511145.b3288	GO Process	GO:0009987	Cellular process
fmt	511145.b3288	GO Process	GO:0010467	Gene expression
fmt	511145.b3288	GO Process	GO:0016070	RNA metabolic process
fmt	511145.b3288	GO Process	GO:0019538	Protein metabolic process
fmt	511145.b3288	GO Process	GO:0019988	charged-tRNA amino acid modification
fmt	511145.b3288	GO Process	GO:0034470	ncRNA processing
fmt	511145.b3288	GO Process	GO:0034641	Cellular nitrogen compound metabolic process
fmt	511145.b3288	GO Process	GO:0034645	Cellular macromolecule biosynthetic process
fmt	511145.b3288	GO Process	GO:0034660	ncRNA metabolic process
fmt	511145.b3288	GO Process	GO:0043043	Peptide biosynthetic process
fmt	511145.b3288	GO Process	GO:0043170	Macromolecule metabolic process
fmt	511145.b3288	GO Process	GO:0043412	Macromolecule modification
fmt	511145.b3288	GO Process	GO:0043603	Cellular amide metabolic process
fmt	511145.b3288	GO Process	GO:0043604	Amide biosynthetic process
fmt	511145.b3288	GO Process	GO:0044237	Cellular metabolic process
fmt	511145.b3288	GO Process	GO:0044238	Primary metabolic process
fmt	511145.b3288	GO Process	GO:0044249	Cellular biosynthetic process
fmt	511145.b3288	GO Process	GO:0044260	Cellular macromolecule metabolic process
fmt	511145.b3288	GO Process	GO:0044271	Cellular nitrogen compound biosynthetic process
fmt	511145.b3288	GO Process	GO:0046483	Heterocycle metabolic process
fmt	511145.b3288	GO Process	GO:0071704	Organic substance metabolic process
fmt	511145.b3288	GO Process	GO:0071951	Conversion of methionyl-tRNA to N-formyl-methionyl-tRNA
fmt	511145.b3288	GO Process	GO:0090304	Nucleic acid metabolic process
fmt	511145.b3288	GO Process	GO:1901360	Organic cyclic compound metabolic process
fmt	511145.b3288	GO Process	GO:1901564	Organonitrogen compound metabolic process
fmt	511145.b3288	GO Process	GO:1901566	Organonitrogen compound biosynthetic process
fmt	511145.b3288	GO Process	GO:1901576	Organic substance biosynthetic process
fmt	511145.b3288	GO Function	GO:0003824	Catalytic activity
fmt	511145.b3288	GO Function	GO:0004479	methionyl-tRNA formyltransferase activity
fmt	511145.b3288	GO Function	GO:0016740	Transferase activity
fmt	511145.b3288	GO Function	GO:0016741	Transferase activity, transferring one-carbon groups
fmt	511145.b3288	GO Function	GO:0016742	Hydroxymethyl-, formyl- and related transferase activity
fmt	511145.b3288	GO Function	GO:0140098	Catalytic activity, acting on RNA
fmt	511145.b3288	GO Function	GO:0140101	Catalytic activity, acting on a tRNA
fmt	511145.b3288	GO Function	GO:0140640	Catalytic activity, acting on a nucleic acid
fmt	511145.b3288	GO Component	GO:0005622	Intracellular anatomical structure
fmt	511145.b3288	GO Component	GO:0005737	Cytoplasm
fmt	511145.b3288	GO Component	GO:0005829	Cytosol
fmt	511145.b3288	GO Component	GO:0110165	Cellular anatomical entity
fmt	511145.b3288	STRING clusters	CL:481	Mixed, incl. tRNA threonylcarbamoyladenosine modification, and Horizontal gene transfer
fmt	511145.b3288	STRING clusters	CL:482	Mixed, incl. Horizontal gene transfer, and C-methyltransferase activity
fmt	511145.b3288	STRING clusters	CL:483	Mixed, incl. Horizontal gene transfer, and C-methyltransferase activity
fmt	511145.b3288	STRING clusters	CL:484	Mixed, incl. C-methyltransferase activity, and Formyl transferase, C-terminal
fmt	511145.b3288	KEGG	eco00670	One carbon pool by folate
fmt	511145.b3288	KEGG	eco00970	Aminoacyl-tRNA biosynthesis
fmt	511145.b3288	COMPARTMENTS	GOCC:0005622	Intracellular
fmt	511145.b3288	COMPARTMENTS	GOCC:0005737	Cytoplasm
fmt	511145.b3288	COMPARTMENTS	GOCC:0005829	Cytosol
fmt	511145.b3288	COMPARTMENTS	GOCC:0110165	Cellular anatomical entity
fmt	511145.b3288	UniProt Keywords	KW-0648	Protein biosynthesis
fmt	511145.b3288	UniProt Keywords	KW-0808	Transferase
fmt	511145.b3288	Pfam	PF00551	Formyl transferase
fmt	511145.b3288	Pfam	PF02911	Formyl transferase, C-terminal domain
fmt	511145.b3288	InterPro	IPR001555	Phosphoribosylglycinamide formyltransferase, active site
fmt	511145.b3288	InterPro	IPR002376	Formyl transferase, N-terminal
fmt	511145.b3288	InterPro	IPR005793	Formyl transferase, C-terminal
fmt	511145.b3288	InterPro	IPR005794	Methionyl-tRNA formyltransferase
fmt	511145.b3288	InterPro	IPR011034	Formyl transferase-like, C-terminal domain superfamily
fmt	511145.b3288	InterPro	IPR036477	Formyl transferase, N-terminal domain superfamily
fmt	511145.b3288	InterPro	IPR037022	Formyl transferase, C-terminal domain superfamily
fmt	511145.b3288	InterPro	IPR041711	Methionyl-tRNA formyltransferase, N-terminal domain
fmt	511145.b3288	InterPro	IPR044135	Methionyl-tRNA formyltransferase, C-terminal domain
gapA	511145.b1779	GO Process	GO:0005975	Carbohydrate metabolic process
gapA	511145.b1779	GO Process	GO:0005996	Monosaccharide metabolic process
gapA	511145.b1779	GO Process	GO:0006006	Glucose metabolic process
gapA	511145.b1779	GO Process	GO:0006082	Organic acid metabolic process
gapA	511145.b1779	GO Process	GO:0006090	Pyruvate metabolic process
gapA	511145.b1779	GO Process	GO:0006091	Generation of precursor metabolites and energy
gapA	511145.b1779	GO Process	GO:0006096	Glycolytic process
gapA	511145.b1779	GO Process	GO:0006139	Nucleobase-containing compound metabolic process
gapA	511145.b1779	GO Process	GO:0006163	Purine nucleotide metabolic process
gapA	511145.b1779	GO Process	GO:0006165	Nucleoside diphosphate phosphorylation
gapA	511145.b1779	GO Process	GO:0006725	Cellular aromatic compound metabolic process
gapA	511145.b1779	GO Process	GO:0006753	Nucleoside phosphate metabolic process
gapA	511145.b1779	GO Process	GO:0006757	ATP generation from ADP
gapA	511145.b1779	GO Process	GO:0006793	Phosphorus metabolic process
gapA	511145.b1779	GO Process	GO:0006796	Phosphate-containing compound metabolic process
gapA	511145.b1779	GO Process	GO:0006807	Nitrogen compound metabolic process
gapA	511145.b1779	GO Process	GO:0008152	Metabolic process
gapA	511145.b1779	GO Process	GO:0009056	Catabolic process
gapA	511145.b1779	GO Process	GO:0009117	Nucleotide metabolic process
gapA	511145.b1779	GO Process	GO:0009132	Nucleoside diphosphate metabolic process
gapA	511145.b1779	GO Process	GO:0009135	Purine nucleoside diphosphate metabolic process
gapA	511145.b1779	GO Process	GO:0009141	Nucleoside triphosphate metabolic process
gapA	511145.b1779	GO Process	GO:0009144	Purine nucleoside triphosphate metabolic process
gapA	511145.b1779	GO Process	GO:0009150	Purine ribonucleotide metabolic process
gapA	511145.b1779	GO Process	GO:0009179	Purine ribonucleoside diphosphate metabolic process
gapA	511145.b1779	GO Process	GO:0009185	Ribonucleoside diphosphate metabolic process
gapA	511145.b1779	GO Process	GO:0009199	Ribonucleoside triphosphate metabolic process
gapA	511145.b1779	GO Process	GO:0009205	Purine ribonucleoside triphosphate metabolic process
gapA	511145.b1779	GO Process	GO:0009259	Ribonucleotide metabolic process
gapA	511145.b1779	GO Process	GO:0009987	Cellular process
gapA	511145.b1779	GO Process	GO:0016052	Carbohydrate catabolic process
gapA	511145.b1779	GO Process	GO:0016310	Phosphorylation
gapA	511145.b1779	GO Process	GO:0019318	Hexose metabolic process
gapA	511145.b1779	GO Process	GO:0019637	Organophosphate metabolic process
gapA	511145.b1779	GO Process	GO:0019693	Ribose phosphate metabolic process
gapA	511145.b1779	GO Process	GO:0019752	Carboxylic acid metabolic process
gapA	511145.b1779	GO Process	GO:0032787	Monocarboxylic acid metabolic process
gapA	511145.b1779	GO Process	GO:0034641	Cellular nitrogen compound metabolic process
gapA	511145.b1779	GO Process	GO:0043436	Oxoacid metabolic process
gapA	511145.b1779	GO Process	GO:0044237	Cellular metabolic process
gapA	511145.b1779	GO Process	GO:0044238	Primary metabolic process
gapA	511145.b1779	GO Process	GO:0044281	Small molecule metabolic process
gapA	511145.b1779	GO Process	GO:0046031	ADP metabolic process
gapA	511145.b1779	GO Process	GO:0046034	ATP metabolic process
gapA	511145.b1779	GO Process	GO:0046483	Heterocycle metabolic process
gapA	511145.b1779	GO Process	GO:0046939	Nucleotide phosphorylation
gapA	511145.b1779	GO Process	GO:0055086	Nucleobase-containing small molecule metabolic process
gapA	511145.b1779	GO Process	GO:0071704	Organic substance metabolic process
gapA	511145.b1779	GO Process	GO:0072521	Purine-containing compound metabolic process
gapA	511145.b1779	GO Process	GO:1901135	Carbohydrate derivative metabolic process
gapA	511145.b1779	GO Process	GO:1901360	Organic cyclic compound metabolic process
gapA	511145.b1779	GO Process	GO:1901564	Organonitrogen compound metabolic process
gapA	511145.b1779	GO Process	GO:1901575	Organic substance catabolic process
gapA	511145.b1779	GO Function	GO:0000166	Nucleotide binding
gapA	511145.b1779	GO Function	GO:0003824	Catalytic activity
gapA	511145.b1779	GO Function	GO:0004365	glyceraldehyde-3-phosphate dehydrogenase (NAD+) (phosphorylating) activity
gapA	511145.b1779	GO Function	GO:0005488	Binding
gapA	511145.b1779	GO Function	GO:0005515	Protein binding
gapA	511145.b1779	GO Function	GO:0016491	Oxidoreductase activity
gapA	511145.b1779	GO Function	GO:0016620	Oxidoreductase activity, acting on the aldehyde or oxo group of donors, NAD or NADP as acceptor
gapA	511145.b1779	GO Function	GO:0016903	Oxidoreductase activity, acting on the aldehyde or oxo group of donors
gapA	511145.b1779	GO Function	GO:0036094	Small molecule binding
gapA	511145.b1779	GO Function	GO:0042802	Identical protein binding
gapA	511145.b1779	GO Function	GO:0043891	glyceraldehyde-3-phosphate dehydrogenase (NAD(P)+) (phosphorylating) activity
gapA	511145.b1779	GO Function	GO:0050661	NADP binding
gapA	511145.b1779	GO Function	GO:0051287	NAD binding
gapA	511145.b1779	GO Function	GO:0097159	Organic cyclic compound binding
gapA	511145.b1779	GO Function	GO:1901265	Nucleoside phosphate binding
gapA	511145.b1779	GO Function	GO:1901363	Heterocyclic compound binding
gapA	511145.b1779	GO Component	GO:0005622	Intracellular anatomical structure
gapA	511145.b1779	GO Component	GO:0005737	Cytoplasm
gapA	511145.b1779	GO Component	GO:0005829	Cytosol
gapA	511145.b1779	GO Component	GO:0016020	Membrane
gapA	511145.b1779	GO Component	GO:0110165	Cellular anatomical entity
gapA	511145.b1779	STRING clusters	CL:2010	Mixed, incl. Carbon metabolism, and Fatty acid metabolic process
gapA	511145.b1779	STRING clusters	CL:2011	Carbon metabolism, and Oxidative phosphorylation
gapA	511145.b1779	STRING clusters	CL:2012	Carbon metabolism, and Pyruvate metabolism
gapA	511145.b1779	STRING clusters	CL:2013	Carbon metabolism, and Formate C-acetyltransferase activity
gapA	511145.b1779	STRING clusters	CL:2109	Pentose phosphate pathway, and Glycolysis
gapA	511145.b1779	STRING clusters	CL:2111	Pentose phosphate pathway, and Gluconeogenesis
gapA	511145.b1779	STRING clusters	CL:2112	Gluconeogenesis, and Glycolysis
gapA	511145.b1779	STRING clusters	CL:2114	Glycolysis
gapA	511145.b1779	KEGG	eco00010	Glycolysis / Gluconeogenesis
gapA	511145.b1779	KEGG	eco01100	Metabolic pathways
gapA	511145.b1779	KEGG	eco01110	Biosynthesis of secondary metabolites
gapA	511145.b1779	KEGG	eco01120	Microbial metabolism in diverse environments
gapA	511145.b1779	KEGG	eco01200	Carbon metabolism
gapA	511145.b1779	KEGG	eco01230	Biosynthesis of amino acids
gapA	511145.b1779	COMPARTMENTS	GOCC:0005622	Intracellular
gapA	511145.b1779	COMPARTMENTS	GOCC:0005737	Cytoplasm
gapA	511145.b1779	COMPARTMENTS	GOCC:0005829	Cytosol
gapA	511145.b1779	COMPARTMENTS	GOCC:0110165	Cellular anatomical entity
gapA	511145.b1779	UniProt Keywords	KW-0007	Acetylation
gapA	511145.b1779	UniProt Keywords	KW-0324	Glycolysis
gapA	511145.b1779	UniProt Keywords	KW-0520	NAD
gapA	511145.b1779	UniProt Keywords	KW-0547	Nucleotide-binding
gapA	511145.b1779	UniProt Keywords	KW-0560	Oxidoreductase
gapA	511145.b1779	UniProt Keywords	KW-0963	Cytoplasm
gapA	511145.b1779	Pfam	PF02800	Glyceraldehyde 3-phosphate dehydrogenase, C-terminal domain
gapA	511145.b1779	InterPro	IPR006424	Glyceraldehyde-3-phosphate dehydrogenase, type I
gapA	511145.b1779	InterPro	IPR020828	Glyceraldehyde 3-phosphate dehydrogenase, NAD(P) binding domain
gapA	511145.b1779	InterPro	IPR020829	Glyceraldehyde 3-phosphate dehydrogenase, catalytic domain
gapA	511145.b1779	InterPro	IPR020830	Glyceraldehyde 3-phosphate dehydrogenase, active site
gapA	511145.b1779	InterPro	IPR020831	Glyceraldehyde/Erythrose phosphate dehydrogenase family
gapA	511145.b1779	InterPro	IPR036291	NAD(P)-binding domain superfamily
gapA	511145.b1779	SMART	SM00846	Glyceraldehyde 3-phosphate dehydrogenase, NAD binding domain
glgA	511145.b3429	GO Process	GO:0000271	Polysaccharide biosynthetic process
glgA	511145.b3429	GO Process	GO:0005975	Carbohydrate metabolic process
glgA	511145.b3429	GO Process	GO:0005976	Polysaccharide metabolic process
glgA	511145.b3429	GO Process	GO:0005977	Glycogen metabolic process
glgA	511145.b3429	GO Process	GO:0005978	Glycogen biosynthetic process
glgA	511145.b3429	GO Process	GO:0006073	Cellular glucan metabolic process
glgA	511145.b3429	GO Process	GO:0006091	Generation of precursor metabolites and energy
glgA	511145.b3429	GO Process	GO:0006112	Energy reserve metabolic process
glgA	511145.b3429	GO Process	GO:0006950	Response to stress
glgA	511145.b3429	GO Process	GO:0006974	Cellular response to DNA damage stimulus
glgA	511145.b3429	GO Process	GO:0008152	Metabolic process
glgA	511145.b3429	GO Process	GO:0009058	Biosynthetic process
glgA	511145.b3429	GO Process	GO:0009059	Macromolecule biosynthetic process
glgA	511145.b3429	GO Process	GO:0009250	Glucan biosynthetic process
glgA	511145.b3429	GO Process	GO:0009987	Cellular process
glgA	511145.b3429	GO Process	GO:0015980	Energy derivation by oxidation of organic compounds
glgA	511145.b3429	GO Process	GO:0016051	Carbohydrate biosynthetic process
glgA	511145.b3429	GO Process	GO:0033554	Cellular response to stress
glgA	511145.b3429	GO Process	GO:0033692	Cellular polysaccharide biosynthetic process
glgA	511145.b3429	GO Process	GO:0034637	Cellular carbohydrate biosynthetic process
glgA	511145.b3429	GO Process	GO:0034645	Cellular macromolecule biosynthetic process
glgA	511145.b3429	GO Process	GO:0043170	Macromolecule metabolic process
glgA	511145.b3429	GO Process	GO:0044042	Glucan metabolic process
glgA	511145.b3429	GO Process	GO:0044237	Cellular metabolic process
glgA	511145.b3429	GO Process	GO:0044238	Primary metabolic process
glgA	511145.b3429	GO Process	GO:0044249	Cellular biosynthetic process
glgA	511145.b3429	GO Process	GO:0044260	Cellular macromolecule metabolic process
glgA	511145.b3429	GO Process	GO:0044262	Cellular carbohydrate metabolic process
glgA	511145.b3429	GO Process	GO:0044264	Cellular polysaccharide metabolic process
glgA	511145.b3429	GO Process	GO:0050896	Response to stimulus
glgA	511145.b3429	GO Process	GO:0051716	Cellular response to stimulus
glgA	511145.b3429	GO Process	GO:0071704	Organic substance metabolic process
glgA	511145.b3429	GO Process	GO:1901576	Organic substance biosynthetic process
glgA	511145.b3429	GO Function	GO:0003824	Catalytic activity
glgA	511145.b3429	GO Function	GO:0004373	Glycogen (starch) synthase activity
glgA	511145.b3429	GO Function	GO:0008194	UDP-glycosyltransferase activity
glgA	511145.b3429	GO Function	GO:0009011	Starch synthase activity
glgA	511145.b3429	GO Function	GO:0016740	Transferase activity
glgA	511145.b3429	GO Function	GO:0016757	Glycosyltransferase activity
glgA	511145.b3429	GO Function	GO:0016758	Hexosyltransferase activity
glgA	511145.b3429	GO Function	GO:0033201	alpha-1,4-glucan synthase activity
glgA	511145.b3429	GO Function	GO:0035251	UDP-glucosyltransferase activity
glgA	511145.b3429	GO Function	GO:0046527	Glucosyltransferase activity
glgA	511145.b3429	GO Component	GO:0005622	Intracellular anatomical structure
glgA	511145.b3429	GO Component	GO:0005737	Cytoplasm
glgA	511145.b3429	GO Component	GO:0005829	Cytosol
glgA	511145.b3429	GO Component	GO:0110165	Cellular anatomical entity
glgA	511145.b3429	STRING clusters	CL:5082	Mixed, incl. Phosphoenolpyruvate-dependent sugar phosphotransferase system, and Carbohydrate metabolism
glgA	511145.b3429	STRING clusters	CL:5322	Mixed, incl. Starch and sucrose metabolism, and 6-sulfoquinovose(1-) catabolic process
glgA	511145.b3429	STRING clusters	CL:5323	Starch and sucrose metabolism, and Galactose catabolic process
glgA	511145.b3429	STRING clusters	CL:5324	Starch and sucrose metabolism, and Galactose catabolic process
glgA	511145.b3429	STRING clusters	CL:5325	Glycogen metabolic process, and Galactose catabolic process
glgA	511145.b3429	STRING clusters	CL:5326	Glycogen metabolic process, and Alpha-amylase domain
glgA	511145.b3429	STRING clusters	CL:5328	Glycogen metabolism
glgA	511145.b3429	KEGG	eco00500	Starch and sucrose metabolism
glgA	511145.b3429	KEGG	eco01100	Metabolic pathways
glgA	511145.b3429	KEGG	eco01110	Biosynthesis of secondary metabolites
glgA	511145.b3429	KEGG	eco02026	Biofilm formation - Escherichia coli
glgA	511145.b3429	COMPARTMENTS	GOCC:0005622	Intracellular
glgA	511145.b3429	COMPARTMENTS	GOCC:0005737	Cytoplasm
glgA	511145.b3429	COMPARTMENTS	GOCC:0005829	Cytosol
glgA	511145.b3429	COMPARTMENTS	GOCC:0110165	Cellular anatomical entity
glgA	511145.b3429	UniProt Keywords	KW-0320	Glycogen biosynthesis
glgA	511145.b3429	UniProt Keywords	KW-0328	Glycosyltransferase
glgA	511145.b3429	UniProt Keywords	KW-0808	Transferase
glgA	511145.b3429	Pfam	PF00534	Glycosyl transferases group 1
glgA	511145.b3429	Pfam	PF13439	Glycosyltransferase Family 4
glgA	511145.b3429	InterPro	IPR001296	Glycosyl transferase, family 1
glgA	511145.b3429	InterPro	IPR011835	Bacterial/plant glycogen synthase
glgA	511145.b3429	InterPro	IPR013534	Starch synthase, catalytic domain
gloB	511145.b0212	GO Process	GO:0006081	Cellular aldehyde metabolic process
gloB	511145.b0212	GO Process	GO:0006082	Organic acid metabolic process
gloB	511145.b0212	GO Process	GO:0006089	Lactate metabolic process
gloB	511145.b0212	GO Process	GO:0006950	Response to stress
gloB	511145.b0212	GO Process	GO:0008152	Metabolic process
gloB	511145.b0212	GO Process	GO:0009056	Catabolic process
gloB	511145.b0212	GO Process	GO:0009266	Response to temperature stimulus
gloB	511145.b0212	GO Process	GO:0009408	Response to heat
gloB	511145.b0212	GO Process	GO:0009438	Methylglyoxal metabolic process
gloB	511145.b0212	GO Process	GO:0009628	Response to abiotic stimulus
gloB	511145.b0212	GO Process	GO:0009636	Response to toxic substance
gloB	511145.b0212	GO Process	GO:0009987	Cellular process
gloB	511145.b0212	GO Process	GO:0019243	Methylglyoxal catabolic process to D-lactate via S-lactoyl-glutathione
gloB	511145.b0212	GO Process	GO:0019752	Carboxylic acid metabolic process
gloB	511145.b0212	GO Process	GO:0032787	Monocarboxylic acid metabolic process
gloB	511145.b0212	GO Process	GO:0042180	Cellular ketone metabolic process
gloB	511145.b0212	GO Process	GO:0042182	Ketone catabolic process
gloB	511145.b0212	GO Process	GO:0042221	Response to chemical
gloB	511145.b0212	GO Process	GO:0043436	Oxoacid metabolic process
gloB	511145.b0212	GO Process	GO:0044237	Cellular metabolic process
gloB	511145.b0212	GO Process	GO:0044248	Cellular catabolic process
gloB	511145.b0212	GO Process	GO:0044281	Small molecule metabolic process
gloB	511145.b0212	GO Process	GO:0044282	Small molecule catabolic process
gloB	511145.b0212	GO Process	GO:0046185	Aldehyde catabolic process
gloB	511145.b0212	GO Process	GO:0050896	Response to stimulus
gloB	511145.b0212	GO Process	GO:0051596	Methylglyoxal catabolic process
gloB	511145.b0212	GO Process	GO:0061727	Methylglyoxal catabolic process to lactate
gloB	511145.b0212	GO Process	GO:0071704	Organic substance metabolic process
gloB	511145.b0212	GO Process	GO:1901575	Organic substance catabolic process
gloB	511145.b0212	GO Process	GO:1901615	Organic hydroxy compound metabolic process
gloB	511145.b0212	GO Function	GO:0003824	Catalytic activity
gloB	511145.b0212	GO Function	GO:0004416	Hydroxyacylglutathione hydrolase activity
gloB	511145.b0212	GO Function	GO:0005488	Binding
gloB	511145.b0212	GO Function	GO:0008270	Zinc ion binding
gloB	511145.b0212	GO Function	GO:0016787	Hydrolase activity
gloB	511145.b0212	GO Function	GO:0016788	Hydrolase activity, acting on ester bonds
gloB	511145.b0212	GO Function	GO:0016790	Thiolester hydrolase activity
gloB	511145.b0212	GO Function	GO:0043167	Ion binding
gloB	511145.b0212	GO Function	GO:0043169	Cation binding
gloB	511145.b0212	GO Function	GO:0046872	Metal ion binding
gloB	511145.b0212	GO Function	GO:0046914	Transition metal ion binding
gloB	511145.b0212	STRING clusters	CL:6432	Mixed, incl. LysR, substrate-binding, and Organic acid transmembrane transporter activity
gloB	511145.b0212	STRING clusters	CL:6433	Mixed, incl. LysR, substrate-binding, and Neutral amino acid transmembrane transporter activity
gloB	511145.b0212	STRING clusters	CL:6515	Mixed, incl. Neutral amino acid transmembrane transporter activity, and Ammonia-lyase activity
gloB	511145.b0212	STRING clusters	CL:6516	Mixed, incl. Ammonia-lyase activity, and Serine transmembrane transporter activity
gloB	511145.b0212	STRING clusters	CL:6518	Mixed, incl. Serine dehydratase beta chain, and Methylglyoxal catabolic process to D-lactate via S-lactoyl-glutathione
gloB	511145.b0212	STRING clusters	CL:6519	Methylglyoxal catabolic process to D-lactate via S-lactoyl-glutathione, and Glycerate dehydrogenase activity
gloB	511145.b0212	KEGG	eco00620	Pyruvate metabolism
gloB	511145.b0212	KEGG	eco01100	Metabolic pathways
gloB	511145.b0212	UniProt Keywords	KW-0216	Detoxification
gloB	511145.b0212	UniProt Keywords	KW-0378	Hydrolase
gloB	511145.b0212	UniProt Keywords	KW-0479	Metal-binding
gloB	511145.b0212	UniProt Keywords	KW-0862	Zinc
gloB	511145.b0212	Pfam	PF16123	Hydroxyacylglutathione hydrolase C-terminus
gloB	511145.b0212	InterPro	IPR001279	Metallo-beta-lactamase
gloB	511145.b0212	InterPro	IPR017782	Hydroxyacylglutathione hydrolase
gloB	511145.b0212	InterPro	IPR032282	Hydroxyacylglutathione hydrolase, C-terminal domain
gloB	511145.b0212	InterPro	IPR035680	Hydroxyacylglutathione hydrolase, MBL domain
gloB	511145.b0212	InterPro	IPR036866	Ribonuclease Z/Hydroxyacylglutathione hydrolase-like
gloB	511145.b0212	SMART	SM00849	Metallo-beta-lactamase superfamily
glsB	511145.b1524	GO Process	GO:0006082	Organic acid metabolic process
glsB	511145.b1524	GO Process	GO:0006520	Cellular amino acid metabolic process
glsB	511145.b1524	GO Process	GO:0006536	Glutamate metabolic process
glsB	511145.b1524	GO Process	GO:0006537	Glutamate biosynthetic process
glsB	511145.b1524	GO Process	GO:0006541	Glutamine metabolic process
glsB	511145.b1524	GO Process	GO:0006543	Glutamine catabolic process
glsB	511145.b1524	GO Process	GO:0006807	Nitrogen compound metabolic process
glsB	511145.b1524	GO Process	GO:0008152	Metabolic process
glsB	511145.b1524	GO Process	GO:0008652	Cellular amino acid biosynthetic process
glsB	511145.b1524	GO Process	GO:0009056	Catabolic process
glsB	511145.b1524	GO Process	GO:0009058	Biosynthetic process
glsB	511145.b1524	GO Process	GO:0009063	Cellular amino acid catabolic process
glsB	511145.b1524	GO Process	GO:0009064	Glutamine family amino acid metabolic process
glsB	511145.b1524	GO Process	GO:0009065	Glutamine family amino acid catabolic process
glsB	511145.b1524	GO Process	GO:0009084	Glutamine family amino acid biosynthetic process
glsB	511145.b1524	GO Process	GO:0009987	Cellular process
glsB	511145.b1524	GO Process	GO:0016053	Organic acid biosynthetic process
glsB	511145.b1524	GO Process	GO:0016054	Organic acid catabolic process
glsB	511145.b1524	GO Process	GO:0019752	Carboxylic acid metabolic process
glsB	511145.b1524	GO Process	GO:0040008	Regulation of growth
glsB	511145.b1524	GO Process	GO:0043436	Oxoacid metabolic process
glsB	511145.b1524	GO Process	GO:0043648	Dicarboxylic acid metabolic process
glsB	511145.b1524	GO Process	GO:0043650	Dicarboxylic acid biosynthetic process
glsB	511145.b1524	GO Process	GO:0044237	Cellular metabolic process
glsB	511145.b1524	GO Process	GO:0044238	Primary metabolic process
glsB	511145.b1524	GO Process	GO:0044248	Cellular catabolic process
glsB	511145.b1524	GO Process	GO:0044249	Cellular biosynthetic process
glsB	511145.b1524	GO Process	GO:0044281	Small molecule metabolic process
glsB	511145.b1524	GO Process	GO:0044282	Small molecule catabolic process
glsB	511145.b1524	GO Process	GO:0044283	Small molecule biosynthetic process
glsB	511145.b1524	GO Process	GO:0045926	Negative regulation of growth
glsB	511145.b1524	GO Process	GO:0046394	Carboxylic acid biosynthetic process
glsB	511145.b1524	GO Process	GO:0046395	Carboxylic acid catabolic process
glsB	511145.b1524	GO Process	GO:0048519	Negative regulation of biological process
glsB	511145.b1524	GO Process	GO:0050789	Regulation of biological process
glsB	511145.b1524	GO Process	GO:0065007	Biological regulation
glsB	511145.b1524	GO Process	GO:0071704	Organic substance metabolic process
glsB	511145.b1524	GO Process	GO:1901564	Organonitrogen compound metabolic process
glsB	511145.b1524	GO Process	GO:1901565	Organonitrogen compound catabolic process
glsB	511145.b1524	GO Process	GO:1901566	Organonitrogen compound biosynthetic process
glsB	511145.b1524	GO Process	GO:1901575	Organic substance catabolic process
glsB	511145.b1524	GO Process	GO:1901576	Organic substance biosynthetic process
glsB	511145.b1524	GO Process	GO:1901605	Alpha-amino acid metabolic process
glsB	511145.b1524	GO Process	GO:1901606	Alpha-amino acid catabolic process
glsB	511145.b1524	GO Process	GO:1901607	Alpha-amino acid biosynthetic process
glsB	511145.b1524	GO Function	GO:0003824	Catalytic activity
glsB	511145.b1524	GO Function	GO:0004359	Glutaminase activity
glsB	511145.b1524	GO Function	GO:0016787	Hydrolase activity
glsB	511145.b1524	GO Function	GO:0016810	Hydrolase activity, acting on carbon-nitrogen (but not peptide) bonds
glsB	511145.b1524	GO Function	GO:0016811	Hydrolase activity, acting on carbon-nitrogen (but not peptide) bonds, in linear amides
glsB	511145.b1524	STRING clusters	CL:1426	Amino-acid biosynthesis, and Arginine biosynthesis
glsB	511145.b1524	STRING clusters	CL:1600	Mixed, incl. Glutamine family amino acid biosynthetic process, and Asparagine metabolic process
glsB	511145.b1524	STRING clusters	CL:1601	Glutamine family amino acid biosynthetic process, and Regulation of nitrogen utilization
glsB	511145.b1524	STRING clusters	CL:1639	Mixed, incl. Regulation of nitrogen utilization, and Glutamate biosynthetic process
glsB	511145.b1524	STRING clusters	CL:1640	Glutamate biosynthetic process, and Glycine cleavage complex
glsB	511145.b1524	KEGG	eco00220	Arginine biosynthesis
glsB	511145.b1524	KEGG	eco00250	Alanine, aspartate and glutamate metabolism
glsB	511145.b1524	KEGG	eco00471	D-Glutamine and D-glutamate metabolism
glsB	511145.b1524	KEGG	eco01100	Metabolic pathways
glsB	511145.b1524	KEGG	eco02020	Two-component system
glsB	511145.b1524	UniProt Keywords	KW-0378	Hydrolase
glsB	511145.b1524	Pfam	PF04960	Glutaminase
glsB	511145.b1524	InterPro	IPR012338	Beta-lactamase/transpeptidase-like
glsB	511145.b1524	InterPro	IPR015868	Glutaminase
glyQ	511145.b3560	GO Process	GO:0006082	Organic acid metabolic process
glyQ	511145.b3560	GO Process	GO:0006139	Nucleobase-containing compound metabolic process
glyQ	511145.b3560	GO Process	GO:0006399	tRNA metabolic process
glyQ	511145.b3560	GO Process	GO:0006412	Translation
glyQ	511145.b3560	GO Process	GO:0006418	tRNA aminoacylation for protein translation
glyQ	511145.b3560	GO Process	GO:0006426	glycyl-tRNA aminoacylation
glyQ	511145.b3560	GO Process	GO:0006518	Peptide metabolic process
glyQ	511145.b3560	GO Process	GO:0006520	Cellular amino acid metabolic process
glyQ	511145.b3560	GO Process	GO:0006725	Cellular aromatic compound metabolic process
glyQ	511145.b3560	GO Process	GO:0006807	Nitrogen compound metabolic process
glyQ	511145.b3560	GO Process	GO:0008152	Metabolic process
glyQ	511145.b3560	GO Process	GO:0009058	Biosynthetic process
glyQ	511145.b3560	GO Process	GO:0009059	Macromolecule biosynthetic process
glyQ	511145.b3560	GO Process	GO:0009987	Cellular process
glyQ	511145.b3560	GO Process	GO:0010467	Gene expression
glyQ	511145.b3560	GO Process	GO:0016070	RNA metabolic process
glyQ	511145.b3560	GO Process	GO:0019538	Protein metabolic process
glyQ	511145.b3560	GO Process	GO:0019752	Carboxylic acid metabolic process
glyQ	511145.b3560	GO Process	GO:0034641	Cellular nitrogen compound metabolic process
glyQ	511145.b3560	GO Process	GO:0034645	Cellular macromolecule biosynthetic process
glyQ	511145.b3560	GO Process	GO:0034660	ncRNA metabolic process
glyQ	511145.b3560	GO Process	GO:0043038	Amino acid activation
glyQ	511145.b3560	GO Process	GO:0043039	tRNA aminoacylation
glyQ	511145.b3560	GO Process	GO:0043043	Peptide biosynthetic process
glyQ	511145.b3560	GO Process	GO:0043170	Macromolecule metabolic process
glyQ	511145.b3560	GO Process	GO:0043436	Oxoacid metabolic process
glyQ	511145.b3560	GO Process	GO:0043603	Cellular amide metabolic process
glyQ	511145.b3560	GO Process	GO:0043604	Amide biosynthetic process
glyQ	511145.b3560	GO Process	GO:0044237	Cellular metabolic process
glyQ	511145.b3560	GO Process	GO:0044238	Primary metabolic process
glyQ	511145.b3560	GO Process	GO:0044249	Cellular biosynthetic process
glyQ	511145.b3560	GO Process	GO:0044260	Cellular macromolecule metabolic process
glyQ	511145.b3560	GO Process	GO:0044271	Cellular nitrogen compound biosynthetic process
glyQ	511145.b3560	GO Process	GO:0044281	Small molecule metabolic process
glyQ	511145.b3560	GO Process	GO:0046483	Heterocycle metabolic process
glyQ	511145.b3560	GO Process	GO:0071704	Organic substance metabolic process
glyQ	511145.b3560	GO Process	GO:0090304	Nucleic acid metabolic process
glyQ	511145.b3560	GO Process	GO:1901360	Organic cyclic compound metabolic process
glyQ	511145.b3560	GO Process	GO:1901564	Organonitrogen compound metabolic process
glyQ	511145.b3560	GO Process	GO:1901566	Organonitrogen compound biosynthetic process
glyQ	511145.b3560	GO Process	GO:1901576	Organic substance biosynthetic process
glyQ	511145.b3560	GO Function	GO:0000166	Nucleotide binding
glyQ	511145.b3560	GO Function	GO:0003824	Catalytic activity
glyQ	511145.b3560	GO Function	GO:0004812	aminoacyl-tRNA ligase activity
glyQ	511145.b3560	GO Function	GO:0004820	glycine-tRNA ligase activity
glyQ	511145.b3560	GO Function	GO:0005488	Binding
glyQ	511145.b3560	GO Function	GO:0005524	ATP binding
glyQ	511145.b3560	GO Function	GO:0016874	Ligase activity
glyQ	511145.b3560	GO Function	GO:0016875	Ligase activity, forming carbon-oxygen bonds
glyQ	511145.b3560	GO Function	GO:0017076	Purine nucleotide binding
glyQ	511145.b3560	GO Function	GO:0030554	Adenyl nucleotide binding
glyQ	511145.b3560	GO Function	GO:0032553	Ribonucleotide binding
glyQ	511145.b3560	GO Function	GO:0032555	Purine ribonucleotide binding
glyQ	511145.b3560	GO Function	GO:0032559	Adenyl ribonucleotide binding
glyQ	511145.b3560	GO Function	GO:0035639	Purine ribonucleoside triphosphate binding
glyQ	511145.b3560	GO Function	GO:0036094	Small molecule binding
glyQ	511145.b3560	GO Function	GO:0043167	Ion binding
glyQ	511145.b3560	GO Function	GO:0043168	Anion binding
glyQ	511145.b3560	GO Function	GO:0097159	Organic cyclic compound binding
glyQ	511145.b3560	GO Function	GO:0097367	Carbohydrate derivative binding
glyQ	511145.b3560	GO Function	GO:0140098	Catalytic activity, acting on RNA
glyQ	511145.b3560	GO Function	GO:0140101	Catalytic activity, acting on a tRNA
glyQ	511145.b3560	GO Function	GO:0140640	Catalytic activity, acting on a nucleic acid
glyQ	511145.b3560	GO Function	GO:1901265	Nucleoside phosphate binding
glyQ	511145.b3560	GO Function	GO:1901363	Heterocyclic compound binding
glyQ	511145.b3560	GO Component	GO:0005622	Intracellular anatomical structure
glyQ	511145.b3560	GO Component	GO:0005737	Cytoplasm
glyQ	511145.b3560	GO Component	GO:0005829	Cytosol
glyQ	511145.b3560	GO Component	GO:0009345	glycine-tRNA ligase complex
glyQ	511145.b3560	GO Component	GO:0032991	Protein-containing complex
glyQ	511145.b3560	GO Component	GO:0110165	Cellular anatomical entity
glyQ	511145.b3560	GO Component	GO:1902494	Catalytic complex
glyQ	511145.b3560	STRING clusters	CL:309	Aminoacyl-tRNA synthetase
glyQ	511145.b3560	STRING clusters	CL:311	Aminoacyl-tRNA synthetase
glyQ	511145.b3560	STRING clusters	CL:35	Translation, and Protein export
glyQ	511145.b3560	STRING clusters	CL:37	Translation, and Protein export
glyQ	511145.b3560	KEGG	eco00970	Aminoacyl-tRNA biosynthesis
glyQ	511145.b3560	COMPARTMENTS	GOCC:0005622	Intracellular
glyQ	511145.b3560	COMPARTMENTS	GOCC:0005737	Cytoplasm
glyQ	511145.b3560	COMPARTMENTS	GOCC:0005829	Cytosol
glyQ	511145.b3560	COMPARTMENTS	GOCC:0009345	glycine-tRNA ligase complex
glyQ	511145.b3560	COMPARTMENTS	GOCC:0032991	Protein-containing complex
glyQ	511145.b3560	COMPARTMENTS	GOCC:0110165	Cellular anatomical entity
glyQ	511145.b3560	COMPARTMENTS	GOCC:1902494	Catalytic complex
glyQ	511145.b3560	UniProt Keywords	KW-0030	Aminoacyl-tRNA synthetase
glyQ	511145.b3560	UniProt Keywords	KW-0067	ATP-binding
glyQ	511145.b3560	UniProt Keywords	KW-0436	Ligase
glyQ	511145.b3560	UniProt Keywords	KW-0547	Nucleotide-binding
glyQ	511145.b3560	UniProt Keywords	KW-0648	Protein biosynthesis
glyQ	511145.b3560	UniProt Keywords	KW-0963	Cytoplasm
glyQ	511145.b3560	Pfam	PF02091	Glycyl-tRNA synthetase alpha subunit
glyQ	511145.b3560	InterPro	IPR002310	Glycine-tRNA ligase, alpha subunit
glyQ	511145.b3560	InterPro	IPR006194	Glycine-tRNA synthetase, heterodimeric
glyQ	511145.b3560	InterPro	IPR045864	Class II Aminoacyl-tRNA synthetase/Biotinyl protein ligase (BPL) and lipoyl protein ligase (LPL)
gshA	511145.b2688	GO Process	GO:0006518	Peptide metabolic process
gshA	511145.b2688	GO Process	GO:0006575	Cellular modified amino acid metabolic process
gshA	511145.b2688	GO Process	GO:0006749	Glutathione metabolic process
gshA	511145.b2688	GO Process	GO:0006750	Glutathione biosynthetic process
gshA	511145.b2688	GO Process	GO:0006790	Sulfur compound metabolic process
gshA	511145.b2688	GO Process	GO:0006807	Nitrogen compound metabolic process
gshA	511145.b2688	GO Process	GO:0006950	Response to stress
gshA	511145.b2688	GO Process	GO:0006970	Response to osmotic stress
gshA	511145.b2688	GO Process	GO:0006972	Hyperosmotic response
gshA	511145.b2688	GO Process	GO:0008152	Metabolic process
gshA	511145.b2688	GO Process	GO:0009058	Biosynthetic process
gshA	511145.b2688	GO Process	GO:0009628	Response to abiotic stimulus
gshA	511145.b2688	GO Process	GO:0009987	Cellular process
gshA	511145.b2688	GO Process	GO:0010035	Response to inorganic substance
gshA	511145.b2688	GO Process	GO:0010038	Response to metal ion
gshA	511145.b2688	GO Process	GO:0019184	Nonribosomal peptide biosynthetic process
gshA	511145.b2688	GO Process	GO:0034641	Cellular nitrogen compound metabolic process
gshA	511145.b2688	GO Process	GO:0042221	Response to chemical
gshA	511145.b2688	GO Process	GO:0042398	Cellular modified amino acid biosynthetic process
gshA	511145.b2688	GO Process	GO:0043043	Peptide biosynthetic process
gshA	511145.b2688	GO Process	GO:0043603	Cellular amide metabolic process
gshA	511145.b2688	GO Process	GO:0043604	Amide biosynthetic process
gshA	511145.b2688	GO Process	GO:0044237	Cellular metabolic process
gshA	511145.b2688	GO Process	GO:0044249	Cellular biosynthetic process
gshA	511145.b2688	GO Process	GO:0044271	Cellular nitrogen compound biosynthetic process
gshA	511145.b2688	GO Process	GO:0044272	Sulfur compound biosynthetic process
gshA	511145.b2688	GO Process	GO:0046685	Response to arsenic-containing substance
gshA	511145.b2688	GO Process	GO:0046689	Response to mercury ion
gshA	511145.b2688	GO Process	GO:0050896	Response to stimulus
gshA	511145.b2688	GO Process	GO:0051716	Cellular response to stimulus
gshA	511145.b2688	GO Process	GO:0070887	Cellular response to chemical stimulus
gshA	511145.b2688	GO Process	GO:0071241	Cellular response to inorganic substance
gshA	511145.b2688	GO Process	GO:0071243	Cellular response to arsenic-containing substance
gshA	511145.b2688	GO Process	GO:0071248	Cellular response to metal ion
gshA	511145.b2688	GO Process	GO:0071288	Cellular response to mercury ion
gshA	511145.b2688	GO Process	GO:0071704	Organic substance metabolic process
gshA	511145.b2688	GO Process	GO:1901564	Organonitrogen compound metabolic process
gshA	511145.b2688	GO Process	GO:1901566	Organonitrogen compound biosynthetic process
gshA	511145.b2688	GO Process	GO:1901576	Organic substance biosynthetic process
gshA	511145.b2688	GO Function	GO:0000166	Nucleotide binding
gshA	511145.b2688	GO Function	GO:0003824	Catalytic activity
gshA	511145.b2688	GO Function	GO:0004357	Glutamate-cysteine ligase activity
gshA	511145.b2688	GO Function	GO:0005488	Binding
gshA	511145.b2688	GO Function	GO:0005524	ATP binding
gshA	511145.b2688	GO Function	GO:0016874	Ligase activity
gshA	511145.b2688	GO Function	GO:0016879	Ligase activity, forming carbon-nitrogen bonds
gshA	511145.b2688	GO Function	GO:0016881	Acid-amino acid ligase activity
gshA	511145.b2688	GO Function	GO:0017076	Purine nucleotide binding
gshA	511145.b2688	GO Function	GO:0030554	Adenyl nucleotide binding
gshA	511145.b2688	GO Function	GO:0032553	Ribonucleotide binding
gshA	511145.b2688	GO Function	GO:0032555	Purine ribonucleotide binding
gshA	511145.b2688	GO Function	GO:0032559	Adenyl ribonucleotide binding
gshA	511145.b2688	GO Function	GO:0035639	Purine ribonucleoside triphosphate binding
gshA	511145.b2688	GO Function	GO:0036094	Small molecule binding
gshA	511145.b2688	GO Function	GO:0043167	Ion binding
gshA	511145.b2688	GO Function	GO:0043168	Anion binding
gshA	511145.b2688	GO Function	GO:0043169	Cation binding
gshA	511145.b2688	GO Function	GO:0046872	Metal ion binding
gshA	511145.b2688	GO Function	GO:0097159	Organic cyclic compound binding
gshA	511145.b2688	GO Function	GO:0097367	Carbohydrate derivative binding
gshA	511145.b2688	GO Function	GO:1901265	Nucleoside phosphate binding
gshA	511145.b2688	GO Function	GO:1901363	Heterocyclic compound binding
gshA	511145.b2688	GO Component	GO:0005622	Intracellular anatomical structure
gshA	511145.b2688	GO Component	GO:0005737	Cytoplasm
gshA	511145.b2688	GO Component	GO:0005829	Cytosol
gshA	511145.b2688	GO Component	GO:0110165	Cellular anatomical entity
gshA	511145.b2688	STRING clusters	CL:1242	Mixed, incl. Iron-sulfur cluster assembly, and Antioxidant activity
gshA	511145.b2688	STRING clusters	CL:1243	Mixed, incl. Iron-sulfur cluster assembly, and Antioxidant activity
gshA	511145.b2688	STRING clusters	CL:1244	Antioxidant activity, and Glutathione metabolism
gshA	511145.b2688	STRING clusters	CL:1245	Antioxidant activity, and Glutathione metabolism
gshA	511145.b2688	STRING clusters	CL:1289	Glutathione metabolism
gshA	511145.b2688	STRING clusters	CL:1290	Glutathione biosynthesis, and Peptidase M17, leucyl aminopeptidase, C-terminal
gshA	511145.b2688	KEGG	eco00270	Cysteine and methionine metabolism
gshA	511145.b2688	KEGG	eco00480	Glutathione metabolism
gshA	511145.b2688	KEGG	eco01100	Metabolic pathways
gshA	511145.b2688	COMPARTMENTS	GOCC:0005622	Intracellular
gshA	511145.b2688	COMPARTMENTS	GOCC:0005737	Cytoplasm
gshA	511145.b2688	COMPARTMENTS	GOCC:0005829	Cytosol
gshA	511145.b2688	COMPARTMENTS	GOCC:0110165	Cellular anatomical entity
gshA	511145.b2688	UniProt Keywords	KW-0067	ATP-binding
gshA	511145.b2688	UniProt Keywords	KW-0317	Glutathione biosynthesis
gshA	511145.b2688	UniProt Keywords	KW-0436	Ligase
gshA	511145.b2688	UniProt Keywords	KW-0547	Nucleotide-binding
gshA	511145.b2688	Pfam	PF04262	Glutamate-cysteine ligase
gshA	511145.b2688	InterPro	IPR006334	Glutamate--cysteine ligase, monofunctional
gshA	511145.b2688	InterPro	IPR007370	Glutamate--cysteine ligase
gshA	511145.b2688	InterPro	IPR014746	Glutamine synthetase/guanido kinase, catalytic domain
guaC	511145.b0104	GO Process	GO:0006139	Nucleobase-containing compound metabolic process
guaC	511145.b0104	GO Process	GO:0006163	Purine nucleotide metabolic process
guaC	511145.b0104	GO Process	GO:0006164	Purine nucleotide biosynthetic process
guaC	511145.b0104	GO Process	GO:0006188	IMP biosynthetic process
guaC	511145.b0104	GO Process	GO:0006725	Cellular aromatic compound metabolic process
guaC	511145.b0104	GO Process	GO:0006753	Nucleoside phosphate metabolic process
guaC	511145.b0104	GO Process	GO:0006793	Phosphorus metabolic process
guaC	511145.b0104	GO Process	GO:0006796	Phosphate-containing compound metabolic process
guaC	511145.b0104	GO Process	GO:0006807	Nitrogen compound metabolic process
guaC	511145.b0104	GO Process	GO:0008152	Metabolic process
guaC	511145.b0104	GO Process	GO:0009058	Biosynthetic process
guaC	511145.b0104	GO Process	GO:0009117	Nucleotide metabolic process
guaC	511145.b0104	GO Process	GO:0009123	Nucleoside monophosphate metabolic process
guaC	511145.b0104	GO Process	GO:0009124	Nucleoside monophosphate biosynthetic process
guaC	511145.b0104	GO Process	GO:0009126	Purine nucleoside monophosphate metabolic process
guaC	511145.b0104	GO Process	GO:0009127	Purine nucleoside monophosphate biosynthetic process
guaC	511145.b0104	GO Process	GO:0009150	Purine ribonucleotide metabolic process
guaC	511145.b0104	GO Process	GO:0009152	Purine ribonucleotide biosynthetic process
guaC	511145.b0104	GO Process	GO:0009156	Ribonucleoside monophosphate biosynthetic process
guaC	511145.b0104	GO Process	GO:0009161	Ribonucleoside monophosphate metabolic process
guaC	511145.b0104	GO Process	GO:0009165	Nucleotide biosynthetic process
guaC	511145.b0104	GO Process	GO:0009167	Purine ribonucleoside monophosphate metabolic process
guaC	511145.b0104	GO Process	GO:0009168	Purine ribonucleoside monophosphate biosynthetic process
guaC	511145.b0104	GO Process	GO:0009259	Ribonucleotide metabolic process
guaC	511145.b0104	GO Process	GO:0009260	Ribonucleotide biosynthetic process
guaC	511145.b0104	GO Process	GO:0009987	Cellular process
guaC	511145.b0104	GO Process	GO:0015949	Nucleobase-containing small molecule interconversion
guaC	511145.b0104	GO Process	GO:0015950	Purine nucleotide interconversion
guaC	511145.b0104	GO Process	GO:0015951	Purine ribonucleotide interconversion
guaC	511145.b0104	GO Process	GO:0018130	Heterocycle biosynthetic process
guaC	511145.b0104	GO Process	GO:0019438	Aromatic compound biosynthetic process
guaC	511145.b0104	GO Process	GO:0019637	Organophosphate metabolic process
guaC	511145.b0104	GO Process	GO:0019693	Ribose phosphate metabolic process
guaC	511145.b0104	GO Process	GO:0032261	Purine nucleotide salvage
guaC	511145.b0104	GO Process	GO:0032264	IMP salvage
guaC	511145.b0104	GO Process	GO:0034641	Cellular nitrogen compound metabolic process
guaC	511145.b0104	GO Process	GO:0034654	Nucleobase-containing compound biosynthetic process
guaC	511145.b0104	GO Process	GO:0043094	Cellular metabolic compound salvage
guaC	511145.b0104	GO Process	GO:0043101	Purine-containing compound salvage
guaC	511145.b0104	GO Process	GO:0043173	Nucleotide salvage
guaC	511145.b0104	GO Process	GO:0044237	Cellular metabolic process
guaC	511145.b0104	GO Process	GO:0044238	Primary metabolic process
guaC	511145.b0104	GO Process	GO:0044249	Cellular biosynthetic process
guaC	511145.b0104	GO Process	GO:0044271	Cellular nitrogen compound biosynthetic process
guaC	511145.b0104	GO Process	GO:0044281	Small molecule metabolic process
guaC	511145.b0104	GO Process	GO:0046040	IMP metabolic process
guaC	511145.b0104	GO Process	GO:0046390	Ribose phosphate biosynthetic process
guaC	511145.b0104	GO Process	GO:0046483	Heterocycle metabolic process
guaC	511145.b0104	GO Process	GO:0055086	Nucleobase-containing small molecule metabolic process
guaC	511145.b0104	GO Process	GO:0071704	Organic substance metabolic process
guaC	511145.b0104	GO Process	GO:0072521	Purine-containing compound metabolic process
guaC	511145.b0104	GO Process	GO:0072522	Purine-containing compound biosynthetic process
guaC	511145.b0104	GO Process	GO:0090407	Organophosphate biosynthetic process
guaC	511145.b0104	GO Process	GO:0106380	Purine ribonucleotide salvage
guaC	511145.b0104	GO Process	GO:1901135	Carbohydrate derivative metabolic process
guaC	511145.b0104	GO Process	GO:1901137	Carbohydrate derivative biosynthetic process
guaC	511145.b0104	GO Process	GO:1901293	Nucleoside phosphate biosynthetic process
guaC	511145.b0104	GO Process	GO:1901360	Organic cyclic compound metabolic process
guaC	511145.b0104	GO Process	GO:1901362	Organic cyclic compound biosynthetic process
guaC	511145.b0104	GO Process	GO:1901564	Organonitrogen compound metabolic process
guaC	511145.b0104	GO Process	GO:1901566	Organonitrogen compound biosynthetic process
guaC	511145.b0104	GO Process	GO:1901576	Organic substance biosynthetic process
guaC	511145.b0104	GO Function	GO:0003824	Catalytic activity
guaC	511145.b0104	GO Function	GO:0003920	GMP reductase activity
guaC	511145.b0104	GO Function	GO:0005488	Binding
guaC	511145.b0104	GO Function	GO:0005515	Protein binding
guaC	511145.b0104	GO Function	GO:0016491	Oxidoreductase activity
guaC	511145.b0104	GO Function	GO:0016651	Oxidoreductase activity, acting on NAD(P)H
guaC	511145.b0104	GO Function	GO:0016657	Oxidoreductase activity, acting on NAD(P)H, nitrogenous group as acceptor
guaC	511145.b0104	GO Function	GO:0042802	Identical protein binding
guaC	511145.b0104	GO Function	GO:0043167	Ion binding
guaC	511145.b0104	GO Function	GO:0043169	Cation binding
guaC	511145.b0104	GO Function	GO:0046872	Metal ion binding
guaC	511145.b0104	GO Component	GO:0005622	Intracellular anatomical structure
guaC	511145.b0104	GO Component	GO:0005737	Cytoplasm
guaC	511145.b0104	GO Component	GO:0005829	Cytosol
guaC	511145.b0104	GO Component	GO:0032991	Protein-containing complex
guaC	511145.b0104	GO Component	GO:0110165	Cellular anatomical entity
guaC	511145.b0104	GO Component	GO:1902494	Catalytic complex
guaC	511145.b0104	GO Component	GO:1902560	GMP reductase complex
guaC	511145.b0104	GO Component	GO:1990204	Oxidoreductase complex
guaC	511145.b0104	STRING clusters	CL:1684	Mixed, incl. Nucleoside monophosphate biosynthetic process, and Pyrimidine metabolism
guaC	511145.b0104	STRING clusters	CL:1685	Nucleoside monophosphate biosynthetic process, and Nucleobase transport
guaC	511145.b0104	STRING clusters	CL:1686	Ribonucleoside monophosphate biosynthetic process, and Nucleobase transport
guaC	511145.b0104	STRING clusters	CL:1687	Ribonucleoside monophosphate biosynthetic process, and Nucleobase transport
guaC	511145.b0104	STRING clusters	CL:1689	Ribonucleoside monophosphate biosynthetic process
guaC	511145.b0104	STRING clusters	CL:1690	IMP metabolic process
guaC	511145.b0104	STRING clusters	CL:1712	GMP biosynthetic process, and Adenosine metabolic process
guaC	511145.b0104	KEGG	eco00230	Purine metabolism
guaC	511145.b0104	KEGG	eco01100	Metabolic pathways
guaC	511145.b0104	COMPARTMENTS	GOCC:0005622	Intracellular
guaC	511145.b0104	COMPARTMENTS	GOCC:0005737	Cytoplasm
guaC	511145.b0104	COMPARTMENTS	GOCC:0005829	Cytosol
guaC	511145.b0104	COMPARTMENTS	GOCC:0032991	Protein-containing complex
guaC	511145.b0104	COMPARTMENTS	GOCC:0110165	Cellular anatomical entity
guaC	511145.b0104	COMPARTMENTS	GOCC:1902494	Catalytic complex
guaC	511145.b0104	COMPARTMENTS	GOCC:1902560	GMP reductase complex
guaC	511145.b0104	COMPARTMENTS	GOCC:1990204	Oxidoreductase complex
guaC	511145.b0104	UniProt Keywords	KW-0479	Metal-binding
guaC	511145.b0104	UniProt Keywords	KW-0521	NADP
guaC	511145.b0104	UniProt Keywords	KW-0560	Oxidoreductase
guaC	511145.b0104	UniProt Keywords	KW-0630	Potassium
guaC	511145.b0104	InterPro	IPR001093	IMP dehydrogenase/GMP reductase
guaC	511145.b0104	InterPro	IPR005993	GMP reductase
guaC	511145.b0104	InterPro	IPR013785	Aldolase-type TIM barrel
guaC	511145.b0104	InterPro	IPR015875	IMP dehydrogenase / GMP reductase, conserved site
guaC	511145.b0104	SMART	SM01240	IMP dehydrogenase / GMP reductase domain
hemL	511145.b0154	GO Process	GO:0006725	Cellular aromatic compound metabolic process
hemL	511145.b0154	GO Process	GO:0006778	Porphyrin-containing compound metabolic process
hemL	511145.b0154	GO Process	GO:0006779	Porphyrin-containing compound biosynthetic process
hemL	511145.b0154	GO Process	GO:0006782	Protoporphyrinogen IX biosynthetic process
hemL	511145.b0154	GO Process	GO:0006783	Heme biosynthetic process
hemL	511145.b0154	GO Process	GO:0006807	Nitrogen compound metabolic process
hemL	511145.b0154	GO Process	GO:0008152	Metabolic process
hemL	511145.b0154	GO Process	GO:0009058	Biosynthetic process
hemL	511145.b0154	GO Process	GO:0009987	Cellular process
hemL	511145.b0154	GO Process	GO:0018130	Heterocycle biosynthetic process
hemL	511145.b0154	GO Process	GO:0019438	Aromatic compound biosynthetic process
hemL	511145.b0154	GO Process	GO:0033013	Tetrapyrrole metabolic process
hemL	511145.b0154	GO Process	GO:0033014	Tetrapyrrole biosynthetic process
hemL	511145.b0154	GO Process	GO:0034641	Cellular nitrogen compound metabolic process
hemL	511145.b0154	GO Process	GO:0042168	Heme metabolic process
hemL	511145.b0154	GO Process	GO:0042440	Pigment metabolic process
hemL	511145.b0154	GO Process	GO:0044237	Cellular metabolic process
hemL	511145.b0154	GO Process	GO:0044249	Cellular biosynthetic process
hemL	511145.b0154	GO Process	GO:0044271	Cellular nitrogen compound biosynthetic process
hemL	511145.b0154	GO Process	GO:0046148	Pigment biosynthetic process
hemL	511145.b0154	GO Process	GO:0046483	Heterocycle metabolic process
hemL	511145.b0154	GO Process	GO:0046501	Protoporphyrinogen IX metabolic process
hemL	511145.b0154	GO Process	GO:0071704	Organic substance metabolic process
hemL	511145.b0154	GO Process	GO:1901360	Organic cyclic compound metabolic process
hemL	511145.b0154	GO Process	GO:1901362	Organic cyclic compound biosynthetic process
hemL	511145.b0154	GO Process	GO:1901564	Organonitrogen compound metabolic process
hemL	511145.b0154	GO Process	GO:1901566	Organonitrogen compound biosynthetic process
hemL	511145.b0154	GO Process	GO:1901576	Organic substance biosynthetic process
hemL	511145.b0154	GO Function	GO:0003824	Catalytic activity
hemL	511145.b0154	GO Function	GO:0005488	Binding
hemL	511145.b0154	GO Function	GO:0008483	Transaminase activity
hemL	511145.b0154	GO Function	GO:0016740	Transferase activity
hemL	511145.b0154	GO Function	GO:0016769	Transferase activity, transferring nitrogenous groups
hemL	511145.b0154	GO Function	GO:0016853	Isomerase activity
hemL	511145.b0154	GO Function	GO:0016866	Intramolecular transferase activity
hemL	511145.b0154	GO Function	GO:0016869	Intramolecular transferase activity, transferring amino groups
hemL	511145.b0154	GO Function	GO:0019842	Vitamin binding
hemL	511145.b0154	GO Function	GO:0030170	Pyridoxal phosphate binding
hemL	511145.b0154	GO Function	GO:0036094	Small molecule binding
hemL	511145.b0154	GO Function	GO:0042286	glutamate-1-semialdehyde 2,1-aminomutase activity
hemL	511145.b0154	GO Function	GO:0043167	Ion binding
hemL	511145.b0154	GO Function	GO:0043168	Anion binding
hemL	511145.b0154	GO Function	GO:0070279	Vitamin B6 binding
hemL	511145.b0154	GO Function	GO:0097159	Organic cyclic compound binding
hemL	511145.b0154	GO Function	GO:1901363	Heterocyclic compound binding
hemL	511145.b0154	GO Component	GO:0005622	Intracellular anatomical structure
hemL	511145.b0154	GO Component	GO:0005737	Cytoplasm
hemL	511145.b0154	GO Component	GO:0005829	Cytosol
hemL	511145.b0154	GO Component	GO:0110165	Cellular anatomical entity
hemL	511145.b0154	STRING clusters	CL:2534	Mixed, incl. Quinone metabolic process, and Tetrapyrrole biosynthetic process
hemL	511145.b0154	STRING clusters	CL:2535	Ubiquinone metabolic process, and Tetrapyrrole biosynthetic process
hemL	511145.b0154	STRING clusters	CL:2601	Porphyrin biosynthesis, and Cobalamin biosynthesis
hemL	511145.b0154	STRING clusters	CL:2602	Protoporphyrinogen IX metabolic process
hemL	511145.b0154	KEGG	eco00860	Porphyrin and chlorophyll metabolism
hemL	511145.b0154	KEGG	eco01100	Metabolic pathways
hemL	511145.b0154	KEGG	eco01110	Biosynthesis of secondary metabolites
hemL	511145.b0154	KEGG	eco01120	Microbial metabolism in diverse environments
hemL	511145.b0154	UniProt Keywords	KW-0413	Isomerase
hemL	511145.b0154	UniProt Keywords	KW-0627	Porphyrin biosynthesis
hemL	511145.b0154	UniProt Keywords	KW-0663	Pyridoxal phosphate
hemL	511145.b0154	UniProt Keywords	KW-0963	Cytoplasm
hemL	511145.b0154	Pfam	PF00202	Aminotransferase class-III
hemL	511145.b0154	InterPro	IPR004639	Tetrapyrrole biosynthesis, glutamate-1-semialdehyde aminotransferase
hemL	511145.b0154	InterPro	IPR005814	Aminotransferase class-III
hemL	511145.b0154	InterPro	IPR015421	Pyridoxal phosphate-dependent transferase, major domain
hemL	511145.b0154	InterPro	IPR015422	Pyridoxal phosphate-dependent transferase, small domain
hemL	511145.b0154	InterPro	IPR015424	Pyridoxal phosphate-dependent transferase
hisC	511145.b2021	GO Process	GO:0000105	Histidine biosynthetic process
hisC	511145.b2021	GO Process	GO:0006082	Organic acid metabolic process
hisC	511145.b2021	GO Process	GO:0006520	Cellular amino acid metabolic process
hisC	511145.b2021	GO Process	GO:0006547	Histidine metabolic process
hisC	511145.b2021	GO Process	GO:0006807	Nitrogen compound metabolic process
hisC	511145.b2021	GO Process	GO:0008152	Metabolic process
hisC	511145.b2021	GO Process	GO:0008652	Cellular amino acid biosynthetic process
hisC	511145.b2021	GO Process	GO:0009058	Biosynthetic process
hisC	511145.b2021	GO Process	GO:0009987	Cellular process
hisC	511145.b2021	GO Process	GO:0016053	Organic acid biosynthetic process
hisC	511145.b2021	GO Process	GO:0019752	Carboxylic acid metabolic process
hisC	511145.b2021	GO Process	GO:0043436	Oxoacid metabolic process
hisC	511145.b2021	GO Process	GO:0044237	Cellular metabolic process
hisC	511145.b2021	GO Process	GO:0044238	Primary metabolic process
hisC	511145.b2021	GO Process	GO:0044249	Cellular biosynthetic process
hisC	511145.b2021	GO Process	GO:0044281	Small molecule metabolic process
hisC	511145.b2021	GO Process	GO:0044283	Small molecule biosynthetic process
hisC	511145.b2021	GO Process	GO:0046394	Carboxylic acid biosynthetic process
hisC	511145.b2021	GO Process	GO:0071704	Organic substance metabolic process
hisC	511145.b2021	GO Process	GO:1901564	Organonitrogen compound metabolic process
hisC	511145.b2021	GO Process	GO:1901566	Organonitrogen compound biosynthetic process
hisC	511145.b2021	GO Process	GO:1901576	Organic substance biosynthetic process
hisC	511145.b2021	GO Function	GO:0003824	Catalytic activity
hisC	511145.b2021	GO Function	GO:0004400	Histidinol-phosphate transaminase activity
hisC	511145.b2021	GO Function	GO:0005488	Binding
hisC	511145.b2021	GO Function	GO:0005515	Protein binding
hisC	511145.b2021	GO Function	GO:0008483	Transaminase activity
hisC	511145.b2021	GO Function	GO:0016740	Transferase activity
hisC	511145.b2021	GO Function	GO:0016769	Transferase activity, transferring nitrogenous groups
hisC	511145.b2021	GO Function	GO:0019842	Vitamin binding
hisC	511145.b2021	GO Function	GO:0030170	Pyridoxal phosphate binding
hisC	511145.b2021	GO Function	GO:0036094	Small molecule binding
hisC	511145.b2021	GO Function	GO:0042802	Identical protein binding
hisC	511145.b2021	GO Function	GO:0043167	Ion binding
hisC	511145.b2021	GO Function	GO:0043168	Anion binding
hisC	511145.b2021	GO Function	GO:0070279	Vitamin B6 binding
hisC	511145.b2021	GO Function	GO:0097159	Organic cyclic compound binding
hisC	511145.b2021	GO Function	GO:1901363	Heterocyclic compound binding
hisC	511145.b2021	GO Component	GO:0005622	Intracellular anatomical structure
hisC	511145.b2021	GO Component	GO:0005737	Cytoplasm
hisC	511145.b2021	GO Component	GO:0005829	Cytosol
hisC	511145.b2021	GO Component	GO:0110165	Cellular anatomical entity
hisC	511145.b2021	STRING clusters	CL:1426	Amino-acid biosynthesis, and Arginine biosynthesis
hisC	511145.b2021	STRING clusters	CL:1427	Amino-acid biosynthesis, and D-alanine metabolic process
hisC	511145.b2021	STRING clusters	CL:1428	Amino-acid biosynthesis, and Aromatic amino acid transmembrane transporter activity
hisC	511145.b2021	STRING clusters	CL:1429	Amino-acid biosynthesis
hisC	511145.b2021	STRING clusters	CL:1430	Valine, leucine and isoleucine biosynthesis, and Histidine metabolism
hisC	511145.b2021	STRING clusters	CL:1431	Histidine metabolism, and Lysine biosynthesis
hisC	511145.b2021	STRING clusters	CL:1455	Histidine metabolism
hisC	511145.b2021	KEGG	eco00340	Histidine metabolism
hisC	511145.b2021	KEGG	eco00350	Tyrosine metabolism
hisC	511145.b2021	KEGG	eco00360	Phenylalanine metabolism
hisC	511145.b2021	KEGG	eco00400	Phenylalanine, tyrosine and tryptophan biosynthesis
hisC	511145.b2021	KEGG	eco00401	Novobiocin biosynthesis
hisC	511145.b2021	KEGG	eco01100	Metabolic pathways
hisC	511145.b2021	KEGG	eco01110	Biosynthesis of secondary metabolites
hisC	511145.b2021	KEGG	eco01230	Biosynthesis of amino acids
hisC	511145.b2021	COMPARTMENTS	GOCC:0005622	Intracellular
hisC	511145.b2021	COMPARTMENTS	GOCC:0005737	Cytoplasm
hisC	511145.b2021	COMPARTMENTS	GOCC:0005829	Cytosol
hisC	511145.b2021	COMPARTMENTS	GOCC:0110165	Cellular anatomical entity
hisC	511145.b2021	UniProt Keywords	KW-0028	Amino-acid biosynthesis
hisC	511145.b2021	UniProt Keywords	KW-0032	Aminotransferase
hisC	511145.b2021	UniProt Keywords	KW-0368	Histidine biosynthesis
hisC	511145.b2021	UniProt Keywords	KW-0663	Pyridoxal phosphate
hisC	511145.b2021	UniProt Keywords	KW-0808	Transferase
hisC	511145.b2021	Pfam	PF00155	Aminotransferase class I and II
hisC	511145.b2021	InterPro	IPR001917	Aminotransferase, class-II, pyridoxal-phosphate binding site
hisC	511145.b2021	InterPro	IPR004839	Aminotransferase, class I/classII
hisC	511145.b2021	InterPro	IPR005861	Histidinol-phosphate aminotransferase family
hisC	511145.b2021	InterPro	IPR015421	Pyridoxal phosphate-dependent transferase, major domain
hisC	511145.b2021	InterPro	IPR015422	Pyridoxal phosphate-dependent transferase, small domain
hisC	511145.b2021	InterPro	IPR015424	Pyridoxal phosphate-dependent transferase
hisF	511145.b2025	GO Process	GO:0000105	Histidine biosynthetic process
hisF	511145.b2025	GO Process	GO:0006082	Organic acid metabolic process
hisF	511145.b2025	GO Process	GO:0006520	Cellular amino acid metabolic process
hisF	511145.b2025	GO Process	GO:0006547	Histidine metabolic process
hisF	511145.b2025	GO Process	GO:0006807	Nitrogen compound metabolic process
hisF	511145.b2025	GO Process	GO:0008152	Metabolic process
hisF	511145.b2025	GO Process	GO:0008652	Cellular amino acid biosynthetic process
hisF	511145.b2025	GO Process	GO:0009058	Biosynthetic process
hisF	511145.b2025	GO Process	GO:0009987	Cellular process
hisF	511145.b2025	GO Process	GO:0016053	Organic acid biosynthetic process
hisF	511145.b2025	GO Process	GO:0019752	Carboxylic acid metabolic process
hisF	511145.b2025	GO Process	GO:0043436	Oxoacid metabolic process
hisF	511145.b2025	GO Process	GO:0044237	Cellular metabolic process
hisF	511145.b2025	GO Process	GO:0044238	Primary metabolic process
hisF	511145.b2025	GO Process	GO:0044249	Cellular biosynthetic process
hisF	511145.b2025	GO Process	GO:0044281	Small molecule metabolic process
hisF	511145.b2025	GO Process	GO:0044283	Small molecule biosynthetic process
hisF	511145.b2025	GO Process	GO:0046394	Carboxylic acid biosynthetic process
hisF	511145.b2025	GO Process	GO:0071704	Organic substance metabolic process
hisF	511145.b2025	GO Process	GO:1901564	Organonitrogen compound metabolic process
hisF	511145.b2025	GO Process	GO:1901566	Organonitrogen compound biosynthetic process
hisF	511145.b2025	GO Process	GO:1901576	Organic substance biosynthetic process
hisF	511145.b2025	GO Function	GO:0000107	Imidazoleglycerol-phosphate synthase activity
hisF	511145.b2025	GO Function	GO:0003824	Catalytic activity
hisF	511145.b2025	GO Function	GO:0016740	Transferase activity
hisF	511145.b2025	GO Function	GO:0016757	Glycosyltransferase activity
hisF	511145.b2025	GO Function	GO:0016763	Pentosyltransferase activity
hisF	511145.b2025	GO Function	GO:0016829	Lyase activity
hisF	511145.b2025	GO Component	GO:0005622	Intracellular anatomical structure
hisF	511145.b2025	GO Component	GO:0005737	Cytoplasm
hisF	511145.b2025	GO Component	GO:0005829	Cytosol
hisF	511145.b2025	GO Component	GO:0009382	Imidazoleglycerol-phosphate synthase complex
hisF	511145.b2025	GO Component	GO:0032991	Protein-containing complex
hisF	511145.b2025	GO Component	GO:0110165	Cellular anatomical entity
hisF	511145.b2025	GO Component	GO:1902494	Catalytic complex
hisF	511145.b2025	GO Component	GO:1990234	Transferase complex
hisF	511145.b2025	STRING clusters	CL:1426	Amino-acid biosynthesis, and Arginine biosynthesis
hisF	511145.b2025	STRING clusters	CL:1427	Amino-acid biosynthesis, and D-alanine metabolic process
hisF	511145.b2025	STRING clusters	CL:1428	Amino-acid biosynthesis, and Aromatic amino acid transmembrane transporter activity
hisF	511145.b2025	STRING clusters	CL:1429	Amino-acid biosynthesis
hisF	511145.b2025	STRING clusters	CL:1430	Valine, leucine and isoleucine biosynthesis, and Histidine metabolism
hisF	511145.b2025	STRING clusters	CL:1431	Histidine metabolism, and Lysine biosynthesis
hisF	511145.b2025	STRING clusters	CL:1455	Histidine metabolism
hisF	511145.b2025	KEGG	eco00340	Histidine metabolism
hisF	511145.b2025	KEGG	eco01100	Metabolic pathways
hisF	511145.b2025	KEGG	eco01110	Biosynthesis of secondary metabolites
hisF	511145.b2025	KEGG	eco01230	Biosynthesis of amino acids
hisF	511145.b2025	COMPARTMENTS	GOCC:0005622	Intracellular
hisF	511145.b2025	COMPARTMENTS	GOCC:0005737	Cytoplasm
hisF	511145.b2025	COMPARTMENTS	GOCC:0005829	Cytosol
hisF	511145.b2025	COMPARTMENTS	GOCC:0009382	Imidazoleglycerol-phosphate synthase complex
hisF	511145.b2025	COMPARTMENTS	GOCC:0032991	Protein-containing complex
hisF	511145.b2025	COMPARTMENTS	GOCC:0110165	Cellular anatomical entity
hisF	511145.b2025	COMPARTMENTS	GOCC:1902494	Catalytic complex
hisF	511145.b2025	COMPARTMENTS	GOCC:1990234	Transferase complex
hisF	511145.b2025	UniProt Keywords	KW-0028	Amino-acid biosynthesis
hisF	511145.b2025	UniProt Keywords	KW-0368	Histidine biosynthesis
hisF	511145.b2025	UniProt Keywords	KW-0456	Lyase
hisF	511145.b2025	UniProt Keywords	KW-0963	Cytoplasm
hisF	511145.b2025	Pfam	PF00977	Histidine biosynthesis protein
hisF	511145.b2025	InterPro	IPR004651	Histidine biosynthesis, HisF
hisF	511145.b2025	InterPro	IPR006062	Histidine biosynthesis protein
hisF	511145.b2025	InterPro	IPR011060	Ribulose-phosphate binding barrel
hisF	511145.b2025	InterPro	IPR013785	Aldolase-type TIM barrel
hybF	511145.b2991	GO Process	GO:0006807	Nitrogen compound metabolic process
hybF	511145.b2991	GO Process	GO:0008152	Metabolic process
hybF	511145.b2991	GO Process	GO:0010467	Gene expression
hybF	511145.b2991	GO Process	GO:0019538	Protein metabolic process
hybF	511145.b2991	GO Process	GO:0043170	Macromolecule metabolic process
hybF	511145.b2991	GO Process	GO:0044238	Primary metabolic process
hybF	511145.b2991	GO Process	GO:0051604	Protein maturation
hybF	511145.b2991	GO Process	GO:0071704	Organic substance metabolic process
hybF	511145.b2991	GO Process	GO:1901564	Organonitrogen compound metabolic process
hybF	511145.b2991	GO Function	GO:0005488	Binding
hybF	511145.b2991	GO Function	GO:0008270	Zinc ion binding
hybF	511145.b2991	GO Function	GO:0016151	Nickel cation binding
hybF	511145.b2991	GO Function	GO:0043167	Ion binding
hybF	511145.b2991	GO Function	GO:0043169	Cation binding
hybF	511145.b2991	GO Function	GO:0046872	Metal ion binding
hybF	511145.b2991	GO Function	GO:0046914	Transition metal ion binding
hybF	511145.b2991	STRING clusters	CL:4810	Mixed, incl. Anaerobic respiration, and Molybdenum cofactor biosynthesis
hybF	511145.b2991	STRING clusters	CL:4956	Anaerobic electron transport chain, and Protein maturation
hybF	511145.b2991	STRING clusters	CL:5030	[Ni-Fe] hydrogenase complex, and Protein maturation
hybF	511145.b2991	STRING clusters	CL:5031	[Ni-Fe] hydrogenase complex, and Protein maturation
hybF	511145.b2991	UniProt Keywords	KW-0479	Metal-binding
hybF	511145.b2991	UniProt Keywords	KW-0533	Nickel
hybF	511145.b2991	UniProt Keywords	KW-0862	Zinc
hybF	511145.b2991	Pfam	PF01155	Hydrogenase/urease nickel incorporation, metallochaperone, hypA
hybF	511145.b2991	InterPro	IPR000688	Hydrogenase maturation factor HypA/HybF
hybF	511145.b2991	InterPro	IPR020538	Hydrogenase nickel incorporation protein HypA/HybF, conserved site
hybF	511145.b2991	InterPro	IPR039002	Hydrogenase maturation factor HybF
ileS	511145.b0026	GO Process	GO:0006082	Organic acid metabolic process
ileS	511145.b0026	GO Process	GO:0006139	Nucleobase-containing compound metabolic process
ileS	511145.b0026	GO Process	GO:0006399	tRNA metabolic process
ileS	511145.b0026	GO Process	GO:0006412	Translation
ileS	511145.b0026	GO Process	GO:0006418	tRNA aminoacylation for protein translation
ileS	511145.b0026	GO Process	GO:0006428	isoleucyl-tRNA aminoacylation
ileS	511145.b0026	GO Process	GO:0006450	Regulation of translational fidelity
ileS	511145.b0026	GO Process	GO:0006518	Peptide metabolic process
ileS	511145.b0026	GO Process	GO:0006520	Cellular amino acid metabolic process
ileS	511145.b0026	GO Process	GO:0006725	Cellular aromatic compound metabolic process
ileS	511145.b0026	GO Process	GO:0006807	Nitrogen compound metabolic process
ileS	511145.b0026	GO Process	GO:0008152	Metabolic process
ileS	511145.b0026	GO Process	GO:0009058	Biosynthetic process
ileS	511145.b0026	GO Process	GO:0009059	Macromolecule biosynthetic process
ileS	511145.b0026	GO Process	GO:0009987	Cellular process
ileS	511145.b0026	GO Process	GO:0010467	Gene expression
ileS	511145.b0026	GO Process	GO:0016070	RNA metabolic process
ileS	511145.b0026	GO Process	GO:0019538	Protein metabolic process
ileS	511145.b0026	GO Process	GO:0019752	Carboxylic acid metabolic process
ileS	511145.b0026	GO Process	GO:0034641	Cellular nitrogen compound metabolic process
ileS	511145.b0026	GO Process	GO:0034645	Cellular macromolecule biosynthetic process
ileS	511145.b0026	GO Process	GO:0034660	ncRNA metabolic process
ileS	511145.b0026	GO Process	GO:0042221	Response to chemical
ileS	511145.b0026	GO Process	GO:0043038	Amino acid activation
ileS	511145.b0026	GO Process	GO:0043039	tRNA aminoacylation
ileS	511145.b0026	GO Process	GO:0043043	Peptide biosynthetic process
ileS	511145.b0026	GO Process	GO:0043170	Macromolecule metabolic process
ileS	511145.b0026	GO Process	GO:0043436	Oxoacid metabolic process
ileS	511145.b0026	GO Process	GO:0043603	Cellular amide metabolic process
ileS	511145.b0026	GO Process	GO:0043604	Amide biosynthetic process
ileS	511145.b0026	GO Process	GO:0044237	Cellular metabolic process
ileS	511145.b0026	GO Process	GO:0044238	Primary metabolic process
ileS	511145.b0026	GO Process	GO:0044249	Cellular biosynthetic process
ileS	511145.b0026	GO Process	GO:0044260	Cellular macromolecule metabolic process
ileS	511145.b0026	GO Process	GO:0044271	Cellular nitrogen compound biosynthetic process
ileS	511145.b0026	GO Process	GO:0044281	Small molecule metabolic process
ileS	511145.b0026	GO Process	GO:0046483	Heterocycle metabolic process
ileS	511145.b0026	GO Process	GO:0046677	Response to antibiotic
ileS	511145.b0026	GO Process	GO:0050896	Response to stimulus
ileS	511145.b0026	GO Process	GO:0065007	Biological regulation
ileS	511145.b0026	GO Process	GO:0065008	Regulation of biological quality
ileS	511145.b0026	GO Process	GO:0071704	Organic substance metabolic process
ileS	511145.b0026	GO Process	GO:0090304	Nucleic acid metabolic process
ileS	511145.b0026	GO Process	GO:0106074	aminoacyl-tRNA metabolism involved in translational fidelity
ileS	511145.b0026	GO Process	GO:1901360	Organic cyclic compound metabolic process
ileS	511145.b0026	GO Process	GO:1901564	Organonitrogen compound metabolic process
ileS	511145.b0026	GO Process	GO:1901566	Organonitrogen compound biosynthetic process
ileS	511145.b0026	GO Process	GO:1901576	Organic substance biosynthetic process
ileS	511145.b0026	GO Function	GO:0000049	tRNA binding
ileS	511145.b0026	GO Function	GO:0000166	Nucleotide binding
ileS	511145.b0026	GO Function	GO:0002161	aminoacyl-tRNA editing activity
ileS	511145.b0026	GO Function	GO:0003676	Nucleic acid binding
ileS	511145.b0026	GO Function	GO:0003723	RNA binding
ileS	511145.b0026	GO Function	GO:0003824	Catalytic activity
ileS	511145.b0026	GO Function	GO:0004812	aminoacyl-tRNA ligase activity
ileS	511145.b0026	GO Function	GO:0004822	isoleucine-tRNA ligase activity
ileS	511145.b0026	GO Function	GO:0005488	Binding
ileS	511145.b0026	GO Function	GO:0005524	ATP binding
ileS	511145.b0026	GO Function	GO:0008270	Zinc ion binding
ileS	511145.b0026	GO Function	GO:0016787	Hydrolase activity
ileS	511145.b0026	GO Function	GO:0016788	Hydrolase activity, acting on ester bonds
ileS	511145.b0026	GO Function	GO:0016874	Ligase activity
ileS	511145.b0026	GO Function	GO:0016875	Ligase activity, forming carbon-oxygen bonds
ileS	511145.b0026	GO Function	GO:0017076	Purine nucleotide binding
ileS	511145.b0026	GO Function	GO:0030554	Adenyl nucleotide binding
ileS	511145.b0026	GO Function	GO:0032553	Ribonucleotide binding
ileS	511145.b0026	GO Function	GO:0032555	Purine ribonucleotide binding
ileS	511145.b0026	GO Function	GO:0032559	Adenyl ribonucleotide binding
ileS	511145.b0026	GO Function	GO:0035639	Purine ribonucleoside triphosphate binding
ileS	511145.b0026	GO Function	GO:0036094	Small molecule binding
ileS	511145.b0026	GO Function	GO:0043167	Ion binding
ileS	511145.b0026	GO Function	GO:0043168	Anion binding
ileS	511145.b0026	GO Function	GO:0043169	Cation binding
ileS	511145.b0026	GO Function	GO:0046872	Metal ion binding
ileS	511145.b0026	GO Function	GO:0046914	Transition metal ion binding
ileS	511145.b0026	GO Function	GO:0052689	Carboxylic ester hydrolase activity
ileS	511145.b0026	GO Function	GO:0097159	Organic cyclic compound binding
ileS	511145.b0026	GO Function	GO:0097367	Carbohydrate derivative binding
ileS	511145.b0026	GO Function	GO:0140098	Catalytic activity, acting on RNA
ileS	511145.b0026	GO Function	GO:0140101	Catalytic activity, acting on a tRNA
ileS	511145.b0026	GO Function	GO:0140640	Catalytic activity, acting on a nucleic acid
ileS	511145.b0026	GO Function	GO:1901265	Nucleoside phosphate binding
ileS	511145.b0026	GO Function	GO:1901363	Heterocyclic compound binding
ileS	511145.b0026	GO Component	GO:0005622	Intracellular anatomical structure
ileS	511145.b0026	GO Component	GO:0005737	Cytoplasm
ileS	511145.b0026	GO Component	GO:0005829	Cytosol
ileS	511145.b0026	GO Component	GO:0110165	Cellular anatomical entity
ileS	511145.b0026	STRING clusters	CL:309	Aminoacyl-tRNA synthetase
ileS	511145.b0026	STRING clusters	CL:311	Aminoacyl-tRNA synthetase
ileS	511145.b0026	STRING clusters	CL:314	Aminoacyl-tRNA synthetase
ileS	511145.b0026	STRING clusters	CL:318	Aminoacyl-tRNA synthetase, class Ia, anticodon-binding, and Lysine-tRNA ligase, class II
ileS	511145.b0026	STRING clusters	CL:35	Translation, and Protein export
ileS	511145.b0026	STRING clusters	CL:37	Translation, and Protein export
ileS	511145.b0026	KEGG	eco00970	Aminoacyl-tRNA biosynthesis
ileS	511145.b0026	COMPARTMENTS	GOCC:0005622	Intracellular
ileS	511145.b0026	COMPARTMENTS	GOCC:0005737	Cytoplasm
ileS	511145.b0026	COMPARTMENTS	GOCC:0005829	Cytosol
ileS	511145.b0026	COMPARTMENTS	GOCC:0110165	Cellular anatomical entity
ileS	511145.b0026	UniProt Keywords	KW-0007	Acetylation
ileS	511145.b0026	UniProt Keywords	KW-0030	Aminoacyl-tRNA synthetase
ileS	511145.b0026	UniProt Keywords	KW-0046	Antibiotic resistance
ileS	511145.b0026	UniProt Keywords	KW-0067	ATP-binding
ileS	511145.b0026	UniProt Keywords	KW-0436	Ligase
ileS	511145.b0026	UniProt Keywords	KW-0479	Metal-binding
ileS	511145.b0026	UniProt Keywords	KW-0547	Nucleotide-binding
ileS	511145.b0026	UniProt Keywords	KW-0648	Protein biosynthesis
ileS	511145.b0026	UniProt Keywords	KW-0862	Zinc
ileS	511145.b0026	UniProt Keywords	KW-0963	Cytoplasm
ileS	511145.b0026	Pfam	PF01406	tRNA synthetases class I (C) catalytic domain
ileS	511145.b0026	Pfam	PF06827	Zinc finger found in FPG and IleRS
ileS	511145.b0026	Pfam	PF08264	Anticodon-binding domain of tRNA ligase
ileS	511145.b0026	Pfam	PF09334	tRNA synthetases class I (M)
ileS	511145.b0026	InterPro	IPR001412	Aminoacyl-tRNA synthetase, class I, conserved site
ileS	511145.b0026	InterPro	IPR002300	Aminoacyl-tRNA synthetase, class Ia
ileS	511145.b0026	InterPro	IPR002301	Isoleucine-tRNA ligase
ileS	511145.b0026	InterPro	IPR009008	Valyl/Leucyl/Isoleucyl-tRNA synthetase, editing domain
ileS	511145.b0026	InterPro	IPR009080	Aminoacyl-tRNA synthetase, class Ia, anticodon-binding
ileS	511145.b0026	InterPro	IPR010663	Zinc finger, FPG/IleRS-type
ileS	511145.b0026	InterPro	IPR013155	Methionyl/Valyl/Leucyl/Isoleucyl-tRNA synthetase, anticodon-binding
ileS	511145.b0026	InterPro	IPR014729	Rossmann-like alpha/beta/alpha sandwich fold
ileS	511145.b0026	InterPro	IPR023585	Isoleucine-tRNA ligase, type 1
ileS	511145.b0026	InterPro	IPR033708	Isoleucyl tRNA synthetase type 1, anticodon-binding domain
insA-6	511145.b4294	GO Process	GO:0006139	Nucleobase-containing compound metabolic process
insA-6	511145.b4294	GO Process	GO:0006259	DNA metabolic process
insA-6	511145.b4294	GO Process	GO:0006310	DNA recombination
insA-6	511145.b4294	GO Process	GO:0006313	Transposition, DNA-mediated
insA-6	511145.b4294	GO Process	GO:0006725	Cellular aromatic compound metabolic process
insA-6	511145.b4294	GO Process	GO:0006807	Nitrogen compound metabolic process
insA-6	511145.b4294	GO Process	GO:0008152	Metabolic process
insA-6	511145.b4294	GO Process	GO:0009987	Cellular process
insA-6	511145.b4294	GO Process	GO:0032196	Transposition
insA-6	511145.b4294	GO Process	GO:0034641	Cellular nitrogen compound metabolic process
insA-6	511145.b4294	GO Process	GO:0043170	Macromolecule metabolic process
insA-6	511145.b4294	GO Process	GO:0044237	Cellular metabolic process
insA-6	511145.b4294	GO Process	GO:0044238	Primary metabolic process
insA-6	511145.b4294	GO Process	GO:0044260	Cellular macromolecule metabolic process
insA-6	511145.b4294	GO Process	GO:0046483	Heterocycle metabolic process
insA-6	511145.b4294	GO Process	GO:0071704	Organic substance metabolic process
insA-6	511145.b4294	GO Process	GO:0090304	Nucleic acid metabolic process
insA-6	511145.b4294	GO Process	GO:1901360	Organic cyclic compound metabolic process
insA-6	511145.b4294	STRING clusters	CL:7138	Mixed, incl. Pilus, and Fimbrium biogenesis
insA-6	511145.b4294	STRING clusters	CL:7307	Transposition, DNA-mediated, and HNH endonuclease
insA-6	511145.b4294	STRING clusters	CL:7309	Transposition, DNA-mediated, and InsA N-terminal domain
insA-6	511145.b4294	STRING clusters	CL:7311	Mixed, incl. Transposase, IS1, and InsA N-terminal domain
insA-6	511145.b4294	UniProt Keywords	KW-0233	DNA recombination
insA-6	511145.b4294	UniProt Keywords	KW-0815	Transposition
insA-6	511145.b4294	Pfam	PF03811	InsA N-terminal domain
insA-6	511145.b4294	Pfam	PF12759	InsA C-terminal domain
insA-6	511145.b4294	InterPro	IPR003220	InsA N-terminal domain
insA-6	511145.b4294	InterPro	IPR024431	Insertion element IS1 protein InsA, helix-turn-helix domain
ispD	511145.b2747	GO Process	GO:0006081	Cellular aldehyde metabolic process
ispD	511145.b2747	GO Process	GO:0006082	Organic acid metabolic process
ispD	511145.b2747	GO Process	GO:0006090	Pyruvate metabolic process
ispD	511145.b2747	GO Process	GO:0006629	Lipid metabolic process
ispD	511145.b2747	GO Process	GO:0006644	Phospholipid metabolic process
ispD	511145.b2747	GO Process	GO:0006720	Isoprenoid metabolic process
ispD	511145.b2747	GO Process	GO:0006721	Terpenoid metabolic process
ispD	511145.b2747	GO Process	GO:0006793	Phosphorus metabolic process
ispD	511145.b2747	GO Process	GO:0006796	Phosphate-containing compound metabolic process
ispD	511145.b2747	GO Process	GO:0008152	Metabolic process
ispD	511145.b2747	GO Process	GO:0008299	Isoprenoid biosynthetic process
ispD	511145.b2747	GO Process	GO:0008610	Lipid biosynthetic process
ispD	511145.b2747	GO Process	GO:0008654	Phospholipid biosynthetic process
ispD	511145.b2747	GO Process	GO:0009058	Biosynthetic process
ispD	511145.b2747	GO Process	GO:0009240	Isopentenyl diphosphate biosynthetic process
ispD	511145.b2747	GO Process	GO:0009987	Cellular process
ispD	511145.b2747	GO Process	GO:0016114	Terpenoid biosynthetic process
ispD	511145.b2747	GO Process	GO:0019288	Isopentenyl diphosphate biosynthetic process, methylerythritol 4-phosphate pathway
ispD	511145.b2747	GO Process	GO:0019637	Organophosphate metabolic process
ispD	511145.b2747	GO Process	GO:0019682	glyceraldehyde-3-phosphate metabolic process
ispD	511145.b2747	GO Process	GO:0019752	Carboxylic acid metabolic process
ispD	511145.b2747	GO Process	GO:0032787	Monocarboxylic acid metabolic process
ispD	511145.b2747	GO Process	GO:0043436	Oxoacid metabolic process
ispD	511145.b2747	GO Process	GO:0044237	Cellular metabolic process
ispD	511145.b2747	GO Process	GO:0044238	Primary metabolic process
ispD	511145.b2747	GO Process	GO:0044249	Cellular biosynthetic process
ispD	511145.b2747	GO Process	GO:0044255	Cellular lipid metabolic process
ispD	511145.b2747	GO Process	GO:0044281	Small molecule metabolic process
ispD	511145.b2747	GO Process	GO:0046490	Isopentenyl diphosphate metabolic process
ispD	511145.b2747	GO Process	GO:0071704	Organic substance metabolic process
ispD	511145.b2747	GO Process	GO:0090407	Organophosphate biosynthetic process
ispD	511145.b2747	GO Process	GO:1901135	Carbohydrate derivative metabolic process
ispD	511145.b2747	GO Process	GO:1901576	Organic substance biosynthetic process
ispD	511145.b2747	GO Function	GO:0000287	Magnesium ion binding
ispD	511145.b2747	GO Function	GO:0003824	Catalytic activity
ispD	511145.b2747	GO Function	GO:0005488	Binding
ispD	511145.b2747	GO Function	GO:0005515	Protein binding
ispD	511145.b2747	GO Function	GO:0016740	Transferase activity
ispD	511145.b2747	GO Function	GO:0016772	Transferase activity, transferring phosphorus-containing groups
ispD	511145.b2747	GO Function	GO:0016779	Nucleotidyltransferase activity
ispD	511145.b2747	GO Function	GO:0042802	Identical protein binding
ispD	511145.b2747	GO Function	GO:0043167	Ion binding
ispD	511145.b2747	GO Function	GO:0043169	Cation binding
ispD	511145.b2747	GO Function	GO:0046872	Metal ion binding
ispD	511145.b2747	GO Function	GO:0050518	2-C-methyl-D-erythritol 4-phosphate cytidylyltransferase activity
ispD	511145.b2747	GO Function	GO:0070567	Cytidylyltransferase activity
ispD	511145.b2747	GO Component	GO:0005622	Intracellular anatomical structure
ispD	511145.b2747	GO Component	GO:0005737	Cytoplasm
ispD	511145.b2747	GO Component	GO:0005829	Cytosol
ispD	511145.b2747	GO Component	GO:0110165	Cellular anatomical entity
ispD	511145.b2747	STRING clusters	CL:2534	Mixed, incl. Quinone metabolic process, and Tetrapyrrole biosynthetic process
ispD	511145.b2747	STRING clusters	CL:2638	Isoprene biosynthesis, and Menaquinone biosynthesis
ispD	511145.b2747	STRING clusters	CL:2670	Isoprene biosynthesis
ispD	511145.b2747	STRING clusters	CL:2673	Isopentenyl diphosphate metabolic process
ispD	511145.b2747	KEGG	eco00900	Terpenoid backbone biosynthesis
ispD	511145.b2747	KEGG	eco01100	Metabolic pathways
ispD	511145.b2747	KEGG	eco01110	Biosynthesis of secondary metabolites
ispD	511145.b2747	COMPARTMENTS	GOCC:0005622	Intracellular
ispD	511145.b2747	COMPARTMENTS	GOCC:0005737	Cytoplasm
ispD	511145.b2747	COMPARTMENTS	GOCC:0005829	Cytosol
ispD	511145.b2747	COMPARTMENTS	GOCC:0110165	Cellular anatomical entity
ispD	511145.b2747	UniProt Keywords	KW-0170	Cobalt
ispD	511145.b2747	UniProt Keywords	KW-0414	Isoprene biosynthesis
ispD	511145.b2747	UniProt Keywords	KW-0460	Magnesium
ispD	511145.b2747	UniProt Keywords	KW-0464	Manganese
ispD	511145.b2747	UniProt Keywords	KW-0548	Nucleotidyltransferase
ispD	511145.b2747	UniProt Keywords	KW-0808	Transferase
ispD	511145.b2747	Pfam	PF12804	MobA-like NTP transferase domain
ispD	511145.b2747	InterPro	IPR001228	2-C-methyl-D-erythritol 4-phosphate cytidylyltransferase
ispD	511145.b2747	InterPro	IPR018294	4-diphosphocytidyl-2C-methyl-D-erythritol synthase, conserved site
ispD	511145.b2747	InterPro	IPR029044	Nucleotide-diphospho-sugar transferases
ispD	511145.b2747	InterPro	IPR034683	Cytidylyltransferase IspD/TarI
ispU	511145.b0174	GO Process	GO:0000270	Peptidoglycan metabolic process
ispU	511145.b0174	GO Process	GO:0006022	Aminoglycan metabolic process
ispU	511145.b0174	GO Process	GO:0006023	Aminoglycan biosynthetic process
ispU	511145.b0174	GO Process	GO:0006024	Glycosaminoglycan biosynthetic process
ispU	511145.b0174	GO Process	GO:0006066	Alcohol metabolic process
ispU	511145.b0174	GO Process	GO:0006629	Lipid metabolic process
ispU	511145.b0174	GO Process	GO:0006720	Isoprenoid metabolic process
ispU	511145.b0174	GO Process	GO:0006807	Nitrogen compound metabolic process
ispU	511145.b0174	GO Process	GO:0007049	Cell cycle
ispU	511145.b0174	GO Process	GO:0008152	Metabolic process
ispU	511145.b0174	GO Process	GO:0008299	Isoprenoid biosynthetic process
ispU	511145.b0174	GO Process	GO:0008360	Regulation of cell shape
ispU	511145.b0174	GO Process	GO:0008610	Lipid biosynthetic process
ispU	511145.b0174	GO Process	GO:0009058	Biosynthetic process
ispU	511145.b0174	GO Process	GO:0009059	Macromolecule biosynthetic process
ispU	511145.b0174	GO Process	GO:0009252	Peptidoglycan biosynthetic process
ispU	511145.b0174	GO Process	GO:0009273	Peptidoglycan-based cell wall biogenesis
ispU	511145.b0174	GO Process	GO:0009987	Cellular process
ispU	511145.b0174	GO Process	GO:0016043	Cellular component organization
ispU	511145.b0174	GO Process	GO:0016093	Polyprenol metabolic process
ispU	511145.b0174	GO Process	GO:0016094	Polyprenol biosynthetic process
ispU	511145.b0174	GO Process	GO:0022603	Regulation of anatomical structure morphogenesis
ispU	511145.b0174	GO Process	GO:0022604	Regulation of cell morphogenesis
ispU	511145.b0174	GO Process	GO:0030203	Glycosaminoglycan metabolic process
ispU	511145.b0174	GO Process	GO:0034645	Cellular macromolecule biosynthetic process
ispU	511145.b0174	GO Process	GO:0042546	Cell wall biogenesis
ispU	511145.b0174	GO Process	GO:0043164	Gram-negative-bacterium-type cell wall biogenesis
ispU	511145.b0174	GO Process	GO:0043170	Macromolecule metabolic process
ispU	511145.b0174	GO Process	GO:0044036	Cell wall macromolecule metabolic process
ispU	511145.b0174	GO Process	GO:0044038	Cell wall macromolecule biosynthetic process
ispU	511145.b0174	GO Process	GO:0044085	Cellular component biogenesis
ispU	511145.b0174	GO Process	GO:0044237	Cellular metabolic process
ispU	511145.b0174	GO Process	GO:0044238	Primary metabolic process
ispU	511145.b0174	GO Process	GO:0044249	Cellular biosynthetic process
ispU	511145.b0174	GO Process	GO:0044255	Cellular lipid metabolic process
ispU	511145.b0174	GO Process	GO:0044260	Cellular macromolecule metabolic process
ispU	511145.b0174	GO Process	GO:0044281	Small molecule metabolic process
ispU	511145.b0174	GO Process	GO:0044283	Small molecule biosynthetic process
ispU	511145.b0174	GO Process	GO:0045229	External encapsulating structure organization
ispU	511145.b0174	GO Process	GO:0046165	Alcohol biosynthetic process
ispU	511145.b0174	GO Process	GO:0050789	Regulation of biological process
ispU	511145.b0174	GO Process	GO:0050793	Regulation of developmental process
ispU	511145.b0174	GO Process	GO:0051301	Cell division
ispU	511145.b0174	GO Process	GO:0065007	Biological regulation
ispU	511145.b0174	GO Process	GO:0070589	Cellular component macromolecule biosynthetic process
ispU	511145.b0174	GO Process	GO:0071554	Cell wall organization or biogenesis
ispU	511145.b0174	GO Process	GO:0071555	Cell wall organization
ispU	511145.b0174	GO Process	GO:0071704	Organic substance metabolic process
ispU	511145.b0174	GO Process	GO:0071840	Cellular component organization or biogenesis
ispU	511145.b0174	GO Process	GO:1901135	Carbohydrate derivative metabolic process
ispU	511145.b0174	GO Process	GO:1901137	Carbohydrate derivative biosynthetic process
ispU	511145.b0174	GO Process	GO:1901564	Organonitrogen compound metabolic process
ispU	511145.b0174	GO Process	GO:1901566	Organonitrogen compound biosynthetic process
ispU	511145.b0174	GO Process	GO:1901576	Organic substance biosynthetic process
ispU	511145.b0174	GO Process	GO:1901615	Organic hydroxy compound metabolic process
ispU	511145.b0174	GO Process	GO:1901617	Organic hydroxy compound biosynthetic process
ispU	511145.b0174	GO Function	GO:0000287	Magnesium ion binding
ispU	511145.b0174	GO Function	GO:0002094	Polyprenyltransferase activity
ispU	511145.b0174	GO Function	GO:0003824	Catalytic activity
ispU	511145.b0174	GO Function	GO:0004659	Prenyltransferase activity
ispU	511145.b0174	GO Function	GO:0005488	Binding
ispU	511145.b0174	GO Function	GO:0008834	Di-trans,poly-cis-decaprenylcistransferase activity
ispU	511145.b0174	GO Function	GO:0016740	Transferase activity
ispU	511145.b0174	GO Function	GO:0016765	Transferase activity, transferring alkyl or aryl (other than methyl) groups
ispU	511145.b0174	GO Function	GO:0030145	Manganese ion binding
ispU	511145.b0174	GO Function	GO:0043167	Ion binding
ispU	511145.b0174	GO Function	GO:0043169	Cation binding
ispU	511145.b0174	GO Function	GO:0046872	Metal ion binding
ispU	511145.b0174	GO Function	GO:0046914	Transition metal ion binding
ispU	511145.b0174	GO Component	GO:0005622	Intracellular anatomical structure
ispU	511145.b0174	GO Component	GO:0005737	Cytoplasm
ispU	511145.b0174	GO Component	GO:0005829	Cytosol
ispU	511145.b0174	GO Component	GO:0110165	Cellular anatomical entity
ispU	511145.b0174	STRING clusters	CL:254	Pyrimidine nucleoside triphosphate biosynthetic process, and Adenylate kinase activity
ispU	511145.b0174	STRING clusters	CL:35	Translation, and Protein export
ispU	511145.b0174	STRING clusters	CL:37	Translation, and Protein export
ispU	511145.b0174	STRING clusters	CL:39	Mixed, incl. Ribonucleoprotein, and Translation regulator activity
ispU	511145.b0174	STRING clusters	CL:40	Ribonucleoprotein, and Translation factor activity, RNA binding
ispU	511145.b0174	KEGG	eco00550	Peptidoglycan biosynthesis
ispU	511145.b0174	KEGG	eco00900	Terpenoid backbone biosynthesis
ispU	511145.b0174	KEGG	eco01110	Biosynthesis of secondary metabolites
ispU	511145.b0174	COMPARTMENTS	GOCC:0005622	Intracellular
ispU	511145.b0174	COMPARTMENTS	GOCC:0005737	Cytoplasm
ispU	511145.b0174	COMPARTMENTS	GOCC:0005829	Cytosol
ispU	511145.b0174	COMPARTMENTS	GOCC:0110165	Cellular anatomical entity
ispU	511145.b0174	UniProt Keywords	KW-0131	Cell cycle
ispU	511145.b0174	UniProt Keywords	KW-0132	Cell division
ispU	511145.b0174	UniProt Keywords	KW-0133	Cell shape
ispU	511145.b0174	UniProt Keywords	KW-0460	Magnesium
ispU	511145.b0174	UniProt Keywords	KW-0479	Metal-binding
ispU	511145.b0174	UniProt Keywords	KW-0573	Peptidoglycan synthesis
ispU	511145.b0174	UniProt Keywords	KW-0808	Transferase
ispU	511145.b0174	UniProt Keywords	KW-0961	Cell wall biogenesis/degradation
ispU	511145.b0174	Pfam	PF01255	Putative undecaprenyl diphosphate synthase
ispU	511145.b0174	InterPro	IPR001441	Decaprenyl diphosphate synthase-like
ispU	511145.b0174	InterPro	IPR018520	Di-trans-poly-cis-decaprenylcistransferase-like, conserved site
ispU	511145.b0174	InterPro	IPR036424	Decaprenyl diphosphate synthase-like superfamily
kdsA	511145.b1215	GO Process	GO:0000271	Polysaccharide biosynthetic process
kdsA	511145.b1215	GO Process	GO:0005975	Carbohydrate metabolic process
kdsA	511145.b1215	GO Process	GO:0005976	Polysaccharide metabolic process
kdsA	511145.b1215	GO Process	GO:0005996	Monosaccharide metabolic process
kdsA	511145.b1215	GO Process	GO:0006082	Organic acid metabolic process
kdsA	511145.b1215	GO Process	GO:0006629	Lipid metabolic process
kdsA	511145.b1215	GO Process	GO:0008152	Metabolic process
kdsA	511145.b1215	GO Process	GO:0008610	Lipid biosynthetic process
kdsA	511145.b1215	GO Process	GO:0008653	Lipopolysaccharide metabolic process
kdsA	511145.b1215	GO Process	GO:0009058	Biosynthetic process
kdsA	511145.b1215	GO Process	GO:0009059	Macromolecule biosynthetic process
kdsA	511145.b1215	GO Process	GO:0009103	Lipopolysaccharide biosynthetic process
kdsA	511145.b1215	GO Process	GO:0009987	Cellular process
kdsA	511145.b1215	GO Process	GO:0016051	Carbohydrate biosynthetic process
kdsA	511145.b1215	GO Process	GO:0016053	Organic acid biosynthetic process
kdsA	511145.b1215	GO Process	GO:0019294	keto-3-deoxy-D-manno-octulosonic acid biosynthetic process
kdsA	511145.b1215	GO Process	GO:0019752	Carboxylic acid metabolic process
kdsA	511145.b1215	GO Process	GO:0033692	Cellular polysaccharide biosynthetic process
kdsA	511145.b1215	GO Process	GO:0034637	Cellular carbohydrate biosynthetic process
kdsA	511145.b1215	GO Process	GO:0034645	Cellular macromolecule biosynthetic process
kdsA	511145.b1215	GO Process	GO:0043170	Macromolecule metabolic process
kdsA	511145.b1215	GO Process	GO:0043436	Oxoacid metabolic process
kdsA	511145.b1215	GO Process	GO:0044237	Cellular metabolic process
kdsA	511145.b1215	GO Process	GO:0044238	Primary metabolic process
kdsA	511145.b1215	GO Process	GO:0044249	Cellular biosynthetic process
kdsA	511145.b1215	GO Process	GO:0044255	Cellular lipid metabolic process
kdsA	511145.b1215	GO Process	GO:0044260	Cellular macromolecule metabolic process
kdsA	511145.b1215	GO Process	GO:0044262	Cellular carbohydrate metabolic process
kdsA	511145.b1215	GO Process	GO:0044264	Cellular polysaccharide metabolic process
kdsA	511145.b1215	GO Process	GO:0044281	Small molecule metabolic process
kdsA	511145.b1215	GO Process	GO:0044283	Small molecule biosynthetic process
kdsA	511145.b1215	GO Process	GO:0046364	Monosaccharide biosynthetic process
kdsA	511145.b1215	GO Process	GO:0046394	Carboxylic acid biosynthetic process
kdsA	511145.b1215	GO Process	GO:0046400	keto-3-deoxy-D-manno-octulosonic acid metabolic process
kdsA	511145.b1215	GO Process	GO:0071704	Organic substance metabolic process
kdsA	511145.b1215	GO Process	GO:1901135	Carbohydrate derivative metabolic process
kdsA	511145.b1215	GO Process	GO:1901137	Carbohydrate derivative biosynthetic process
kdsA	511145.b1215	GO Process	GO:1901576	Organic substance biosynthetic process
kdsA	511145.b1215	GO Process	GO:1903509	Liposaccharide metabolic process
kdsA	511145.b1215	GO Function	GO:0003824	Catalytic activity
kdsA	511145.b1215	GO Function	GO:0005488	Binding
kdsA	511145.b1215	GO Function	GO:0005515	Protein binding
kdsA	511145.b1215	GO Function	GO:0008676	3-deoxy-8-phosphooctulonate synthase activity
kdsA	511145.b1215	GO Function	GO:0016740	Transferase activity
kdsA	511145.b1215	GO Function	GO:0016765	Transferase activity, transferring alkyl or aryl (other than methyl) groups
kdsA	511145.b1215	GO Function	GO:0042802	Identical protein binding
kdsA	511145.b1215	GO Component	GO:0005622	Intracellular anatomical structure
kdsA	511145.b1215	GO Component	GO:0005737	Cytoplasm
kdsA	511145.b1215	GO Component	GO:0005829	Cytosol
kdsA	511145.b1215	GO Component	GO:0032991	Protein-containing complex
kdsA	511145.b1215	GO Component	GO:0110165	Cellular anatomical entity
kdsA	511145.b1215	STRING clusters	CL:3397	Polysaccharide biosynthetic process, and Lipopolysaccharide transport
kdsA	511145.b1215	STRING clusters	CL:3519	Lipopolysaccharide biosynthesis, and Lipopolysaccharide transport
kdsA	511145.b1215	STRING clusters	CL:3520	Lipopolysaccharide biosynthesis, and Lipopolysaccharide transport
kdsA	511145.b1215	STRING clusters	CL:3521	Lipopolysaccharide transport, and Lipopolysaccharide biosynthesis
kdsA	511145.b1215	KEGG	eco00540	Lipopolysaccharide biosynthesis
kdsA	511145.b1215	KEGG	eco01100	Metabolic pathways
kdsA	511145.b1215	COMPARTMENTS	GOCC:0005622	Intracellular
kdsA	511145.b1215	COMPARTMENTS	GOCC:0005737	Cytoplasm
kdsA	511145.b1215	COMPARTMENTS	GOCC:0005829	Cytosol
kdsA	511145.b1215	COMPARTMENTS	GOCC:0032991	Protein-containing complex
kdsA	511145.b1215	COMPARTMENTS	GOCC:0110165	Cellular anatomical entity
kdsA	511145.b1215	UniProt Keywords	KW-0448	Lipopolysaccharide biosynthesis
kdsA	511145.b1215	UniProt Keywords	KW-0808	Transferase
kdsA	511145.b1215	UniProt Keywords	KW-0963	Cytoplasm
kdsA	511145.b1215	Pfam	PF00793	DAHP synthetase I family
kdsA	511145.b1215	InterPro	IPR006218	DAHP synthetase I/KDSA
kdsA	511145.b1215	InterPro	IPR006269	3-deoxy-8-phosphooctulonate synthase
kdsA	511145.b1215	InterPro	IPR013785	Aldolase-type TIM barrel
leuC	511145.b0072	GO Process	GO:0006082	Organic acid metabolic process
leuC	511145.b0072	GO Process	GO:0006520	Cellular amino acid metabolic process
leuC	511145.b0072	GO Process	GO:0006551	Leucine metabolic process
leuC	511145.b0072	GO Process	GO:0006807	Nitrogen compound metabolic process
leuC	511145.b0072	GO Process	GO:0008152	Metabolic process
leuC	511145.b0072	GO Process	GO:0008652	Cellular amino acid biosynthetic process
leuC	511145.b0072	GO Process	GO:0009058	Biosynthetic process
leuC	511145.b0072	GO Process	GO:0009081	Branched-chain amino acid metabolic process
leuC	511145.b0072	GO Process	GO:0009082	Branched-chain amino acid biosynthetic process
leuC	511145.b0072	GO Process	GO:0009098	Leucine biosynthetic process
leuC	511145.b0072	GO Process	GO:0009987	Cellular process
leuC	511145.b0072	GO Process	GO:0016053	Organic acid biosynthetic process
leuC	511145.b0072	GO Process	GO:0019752	Carboxylic acid metabolic process
leuC	511145.b0072	GO Process	GO:0043436	Oxoacid metabolic process
leuC	511145.b0072	GO Process	GO:0044237	Cellular metabolic process
leuC	511145.b0072	GO Process	GO:0044238	Primary metabolic process
leuC	511145.b0072	GO Process	GO:0044249	Cellular biosynthetic process
leuC	511145.b0072	GO Process	GO:0044281	Small molecule metabolic process
leuC	511145.b0072	GO Process	GO:0044283	Small molecule biosynthetic process
leuC	511145.b0072	GO Process	GO:0046394	Carboxylic acid biosynthetic process
leuC	511145.b0072	GO Process	GO:0071704	Organic substance metabolic process
leuC	511145.b0072	GO Process	GO:1901564	Organonitrogen compound metabolic process
leuC	511145.b0072	GO Process	GO:1901566	Organonitrogen compound biosynthetic process
leuC	511145.b0072	GO Process	GO:1901576	Organic substance biosynthetic process
leuC	511145.b0072	GO Process	GO:1901605	Alpha-amino acid metabolic process
leuC	511145.b0072	GO Process	GO:1901607	Alpha-amino acid biosynthetic process
leuC	511145.b0072	GO Function	GO:0003824	Catalytic activity
leuC	511145.b0072	GO Function	GO:0003861	3-isopropylmalate dehydratase activity
leuC	511145.b0072	GO Function	GO:0005488	Binding
leuC	511145.b0072	GO Function	GO:0016829	Lyase activity
leuC	511145.b0072	GO Function	GO:0016835	Carbon-oxygen lyase activity
leuC	511145.b0072	GO Function	GO:0016836	Hydro-lyase activity
leuC	511145.b0072	GO Function	GO:0043167	Ion binding
leuC	511145.b0072	GO Function	GO:0043169	Cation binding
leuC	511145.b0072	GO Function	GO:0046872	Metal ion binding
leuC	511145.b0072	GO Function	GO:0051536	Iron-sulfur cluster binding
leuC	511145.b0072	GO Function	GO:0051539	4 iron, 4 sulfur cluster binding
leuC	511145.b0072	GO Function	GO:0051540	Metal cluster binding
leuC	511145.b0072	GO Component	GO:0005622	Intracellular anatomical structure
leuC	511145.b0072	GO Component	GO:0005737	Cytoplasm
leuC	511145.b0072	GO Component	GO:0005829	Cytosol
leuC	511145.b0072	GO Component	GO:0009316	3-isopropylmalate dehydratase complex
leuC	511145.b0072	GO Component	GO:0032991	Protein-containing complex
leuC	511145.b0072	GO Component	GO:0110165	Cellular anatomical entity
leuC	511145.b0072	GO Component	GO:1902494	Catalytic complex
leuC	511145.b0072	STRING clusters	CL:1426	Amino-acid biosynthesis, and Arginine biosynthesis
leuC	511145.b0072	STRING clusters	CL:1427	Amino-acid biosynthesis, and D-alanine metabolic process
leuC	511145.b0072	STRING clusters	CL:1428	Amino-acid biosynthesis, and Aromatic amino acid transmembrane transporter activity
leuC	511145.b0072	STRING clusters	CL:1429	Amino-acid biosynthesis
leuC	511145.b0072	STRING clusters	CL:1430	Valine, leucine and isoleucine biosynthesis, and Histidine metabolism
leuC	511145.b0072	KEGG	eco00290	Valine, leucine and isoleucine biosynthesis
leuC	511145.b0072	KEGG	eco00660	C5-Branched dibasic acid metabolism
leuC	511145.b0072	KEGG	eco01100	Metabolic pathways
leuC	511145.b0072	KEGG	eco01110	Biosynthesis of secondary metabolites
leuC	511145.b0072	KEGG	eco01210	2-Oxocarboxylic acid metabolism
leuC	511145.b0072	KEGG	eco01230	Biosynthesis of amino acids
leuC	511145.b0072	COMPARTMENTS	GOCC:0005622	Intracellular
leuC	511145.b0072	COMPARTMENTS	GOCC:0005737	Cytoplasm
leuC	511145.b0072	COMPARTMENTS	GOCC:0005829	Cytosol
leuC	511145.b0072	COMPARTMENTS	GOCC:0009316	3-isopropylmalate dehydratase complex
leuC	511145.b0072	COMPARTMENTS	GOCC:0032991	Protein-containing complex
leuC	511145.b0072	COMPARTMENTS	GOCC:0110165	Cellular anatomical entity
leuC	511145.b0072	COMPARTMENTS	GOCC:1902494	Catalytic complex
leuC	511145.b0072	UniProt Keywords	KW-0004	4Fe-4S
leuC	511145.b0072	UniProt Keywords	KW-0028	Amino-acid biosynthesis
leuC	511145.b0072	UniProt Keywords	KW-0100	Branched-chain amino acid biosynthesis
leuC	511145.b0072	UniProt Keywords	KW-0408	Iron
leuC	511145.b0072	UniProt Keywords	KW-0411	Iron-sulfur
leuC	511145.b0072	UniProt Keywords	KW-0432	Leucine biosynthesis
leuC	511145.b0072	UniProt Keywords	KW-0456	Lyase
leuC	511145.b0072	UniProt Keywords	KW-0479	Metal-binding
leuC	511145.b0072	Pfam	PF00330	Aconitase family (aconitate hydratase)
leuC	511145.b0072	InterPro	IPR001030	Aconitase/3-isopropylmalate dehydratase large subunit, alpha/beta/alpha domain
leuC	511145.b0072	InterPro	IPR004430	3-isopropylmalate dehydratase, large subunit
leuC	511145.b0072	InterPro	IPR015931	Aconitase/3-isopropylmalate dehydratase large subunit, alpha/beta/alpha, subdomain 1/3
leuC	511145.b0072	InterPro	IPR018136	Aconitase family, 4Fe-4S cluster binding site
leuC	511145.b0072	InterPro	IPR033941	3-Isopropylmalate dehydratase, catalytic domain
leuC	511145.b0072	InterPro	IPR036008	Aconitase, iron-sulfur domain
leuD	511145.b0071	GO Process	GO:0006082	Organic acid metabolic process
leuD	511145.b0071	GO Process	GO:0006520	Cellular amino acid metabolic process
leuD	511145.b0071	GO Process	GO:0006551	Leucine metabolic process
leuD	511145.b0071	GO Process	GO:0006807	Nitrogen compound metabolic process
leuD	511145.b0071	GO Process	GO:0008152	Metabolic process
leuD	511145.b0071	GO Process	GO:0008652	Cellular amino acid biosynthetic process
leuD	511145.b0071	GO Process	GO:0009058	Biosynthetic process
leuD	511145.b0071	GO Process	GO:0009081	Branched-chain amino acid metabolic process
leuD	511145.b0071	GO Process	GO:0009082	Branched-chain amino acid biosynthetic process
leuD	511145.b0071	GO Process	GO:0009098	Leucine biosynthetic process
leuD	511145.b0071	GO Process	GO:0009987	Cellular process
leuD	511145.b0071	GO Process	GO:0016053	Organic acid biosynthetic process
leuD	511145.b0071	GO Process	GO:0019752	Carboxylic acid metabolic process
leuD	511145.b0071	GO Process	GO:0043436	Oxoacid metabolic process
leuD	511145.b0071	GO Process	GO:0044237	Cellular metabolic process
leuD	511145.b0071	GO Process	GO:0044238	Primary metabolic process
leuD	511145.b0071	GO Process	GO:0044249	Cellular biosynthetic process
leuD	511145.b0071	GO Process	GO:0044281	Small molecule metabolic process
leuD	511145.b0071	GO Process	GO:0044283	Small molecule biosynthetic process
leuD	511145.b0071	GO Process	GO:0046394	Carboxylic acid biosynthetic process
leuD	511145.b0071	GO Process	GO:0071704	Organic substance metabolic process
leuD	511145.b0071	GO Process	GO:1901564	Organonitrogen compound metabolic process
leuD	511145.b0071	GO Process	GO:1901566	Organonitrogen compound biosynthetic process
leuD	511145.b0071	GO Process	GO:1901576	Organic substance biosynthetic process
leuD	511145.b0071	GO Process	GO:1901605	Alpha-amino acid metabolic process
leuD	511145.b0071	GO Process	GO:1901607	Alpha-amino acid biosynthetic process
leuD	511145.b0071	GO Function	GO:0003824	Catalytic activity
leuD	511145.b0071	GO Function	GO:0003861	3-isopropylmalate dehydratase activity
leuD	511145.b0071	GO Function	GO:0016829	Lyase activity
leuD	511145.b0071	GO Function	GO:0016835	Carbon-oxygen lyase activity
leuD	511145.b0071	GO Function	GO:0016836	Hydro-lyase activity
leuD	511145.b0071	GO Component	GO:0005622	Intracellular anatomical structure
leuD	511145.b0071	GO Component	GO:0005737	Cytoplasm
leuD	511145.b0071	GO Component	GO:0005829	Cytosol
leuD	511145.b0071	GO Component	GO:0009316	3-isopropylmalate dehydratase complex
leuD	511145.b0071	GO Component	GO:0032991	Protein-containing complex
leuD	511145.b0071	GO Component	GO:0110165	Cellular anatomical entity
leuD	511145.b0071	GO Component	GO:1902494	Catalytic complex
leuD	511145.b0071	STRING clusters	CL:1426	Amino-acid biosynthesis, and Arginine biosynthesis
leuD	511145.b0071	STRING clusters	CL:1427	Amino-acid biosynthesis, and D-alanine metabolic process
leuD	511145.b0071	STRING clusters	CL:1428	Amino-acid biosynthesis, and Aromatic amino acid transmembrane transporter activity
leuD	511145.b0071	STRING clusters	CL:1429	Amino-acid biosynthesis
leuD	511145.b0071	STRING clusters	CL:1430	Valine, leucine and isoleucine biosynthesis, and Histidine metabolism
leuD	511145.b0071	KEGG	eco00290	Valine, leucine and isoleucine biosynthesis
leuD	511145.b0071	KEGG	eco00660	C5-Branched dibasic acid metabolism
leuD	511145.b0071	KEGG	eco01100	Metabolic pathways
leuD	511145.b0071	KEGG	eco01110	Biosynthesis of secondary metabolites
leuD	511145.b0071	KEGG	eco01210	2-Oxocarboxylic acid metabolism
leuD	511145.b0071	KEGG	eco01230	Biosynthesis of amino acids
leuD	511145.b0071	COMPARTMENTS	GOCC:0005622	Intracellular
leuD	511145.b0071	COMPARTMENTS	GOCC:0005737	Cytoplasm
leuD	511145.b0071	COMPARTMENTS	GOCC:0005829	Cytosol
leuD	511145.b0071	COMPARTMENTS	GOCC:0009316	3-isopropylmalate dehydratase complex
leuD	511145.b0071	COMPARTMENTS	GOCC:0032991	Protein-containing complex
leuD	511145.b0071	COMPARTMENTS	GOCC:0110165	Cellular anatomical entity
leuD	511145.b0071	COMPARTMENTS	GOCC:1902494	Catalytic complex
leuD	511145.b0071	UniProt Keywords	KW-0028	Amino-acid biosynthesis
leuD	511145.b0071	UniProt Keywords	KW-0100	Branched-chain amino acid biosynthesis
leuD	511145.b0071	UniProt Keywords	KW-0432	Leucine biosynthesis
leuD	511145.b0071	UniProt Keywords	KW-0456	Lyase
leuD	511145.b0071	Pfam	PF00694	Aconitase C-terminal domain
leuD	511145.b0071	InterPro	IPR000573	Aconitase A/isopropylmalate dehydratase small subunit, swivel domain
leuD	511145.b0071	InterPro	IPR004431	3-isopropylmalate dehydratase, small subunit
leuD	511145.b0071	InterPro	IPR015928	Aconitase/3-isopropylmalate dehydratase, swivel
leuD	511145.b0071	InterPro	IPR033940	3-isopropylmalate dehydratase, swivel domain
leuS	511145.b0642	GO Process	GO:0006082	Organic acid metabolic process
leuS	511145.b0642	GO Process	GO:0006139	Nucleobase-containing compound metabolic process
leuS	511145.b0642	GO Process	GO:0006399	tRNA metabolic process
leuS	511145.b0642	GO Process	GO:0006412	Translation
leuS	511145.b0642	GO Process	GO:0006418	tRNA aminoacylation for protein translation
leuS	511145.b0642	GO Process	GO:0006429	leucyl-tRNA aminoacylation
leuS	511145.b0642	GO Process	GO:0006450	Regulation of translational fidelity
leuS	511145.b0642	GO Process	GO:0006518	Peptide metabolic process
leuS	511145.b0642	GO Process	GO:0006520	Cellular amino acid metabolic process
leuS	511145.b0642	GO Process	GO:0006725	Cellular aromatic compound metabolic process
leuS	511145.b0642	GO Process	GO:0006807	Nitrogen compound metabolic process
leuS	511145.b0642	GO Process	GO:0008152	Metabolic process
leuS	511145.b0642	GO Process	GO:0009058	Biosynthetic process
leuS	511145.b0642	GO Process	GO:0009059	Macromolecule biosynthetic process
leuS	511145.b0642	GO Process	GO:0009987	Cellular process
leuS	511145.b0642	GO Process	GO:0010467	Gene expression
leuS	511145.b0642	GO Process	GO:0016070	RNA metabolic process
leuS	511145.b0642	GO Process	GO:0019538	Protein metabolic process
leuS	511145.b0642	GO Process	GO:0019752	Carboxylic acid metabolic process
leuS	511145.b0642	GO Process	GO:0034641	Cellular nitrogen compound metabolic process
leuS	511145.b0642	GO Process	GO:0034645	Cellular macromolecule biosynthetic process
leuS	511145.b0642	GO Process	GO:0034660	ncRNA metabolic process
leuS	511145.b0642	GO Process	GO:0043038	Amino acid activation
leuS	511145.b0642	GO Process	GO:0043039	tRNA aminoacylation
leuS	511145.b0642	GO Process	GO:0043043	Peptide biosynthetic process
leuS	511145.b0642	GO Process	GO:0043170	Macromolecule metabolic process
leuS	511145.b0642	GO Process	GO:0043436	Oxoacid metabolic process
leuS	511145.b0642	GO Process	GO:0043603	Cellular amide metabolic process
leuS	511145.b0642	GO Process	GO:0043604	Amide biosynthetic process
leuS	511145.b0642	GO Process	GO:0044237	Cellular metabolic process
leuS	511145.b0642	GO Process	GO:0044238	Primary metabolic process
leuS	511145.b0642	GO Process	GO:0044249	Cellular biosynthetic process
leuS	511145.b0642	GO Process	GO:0044260	Cellular macromolecule metabolic process
leuS	511145.b0642	GO Process	GO:0044271	Cellular nitrogen compound biosynthetic process
leuS	511145.b0642	GO Process	GO:0044281	Small molecule metabolic process
leuS	511145.b0642	GO Process	GO:0046483	Heterocycle metabolic process
leuS	511145.b0642	GO Process	GO:0065007	Biological regulation
leuS	511145.b0642	GO Process	GO:0065008	Regulation of biological quality
leuS	511145.b0642	GO Process	GO:0071704	Organic substance metabolic process
leuS	511145.b0642	GO Process	GO:0090304	Nucleic acid metabolic process
leuS	511145.b0642	GO Process	GO:0106074	aminoacyl-tRNA metabolism involved in translational fidelity
leuS	511145.b0642	GO Process	GO:1901360	Organic cyclic compound metabolic process
leuS	511145.b0642	GO Process	GO:1901564	Organonitrogen compound metabolic process
leuS	511145.b0642	GO Process	GO:1901566	Organonitrogen compound biosynthetic process
leuS	511145.b0642	GO Process	GO:1901576	Organic substance biosynthetic process
leuS	511145.b0642	GO Function	GO:0000166	Nucleotide binding
leuS	511145.b0642	GO Function	GO:0002161	aminoacyl-tRNA editing activity
leuS	511145.b0642	GO Function	GO:0003824	Catalytic activity
leuS	511145.b0642	GO Function	GO:0004812	aminoacyl-tRNA ligase activity
leuS	511145.b0642	GO Function	GO:0004823	leucine-tRNA ligase activity
leuS	511145.b0642	GO Function	GO:0005488	Binding
leuS	511145.b0642	GO Function	GO:0005524	ATP binding
leuS	511145.b0642	GO Function	GO:0016787	Hydrolase activity
leuS	511145.b0642	GO Function	GO:0016788	Hydrolase activity, acting on ester bonds
leuS	511145.b0642	GO Function	GO:0016874	Ligase activity
leuS	511145.b0642	GO Function	GO:0016875	Ligase activity, forming carbon-oxygen bonds
leuS	511145.b0642	GO Function	GO:0017076	Purine nucleotide binding
leuS	511145.b0642	GO Function	GO:0030554	Adenyl nucleotide binding
leuS	511145.b0642	GO Function	GO:0032553	Ribonucleotide binding
leuS	511145.b0642	GO Function	GO:0032555	Purine ribonucleotide binding
leuS	511145.b0642	GO Function	GO:0032559	Adenyl ribonucleotide binding
leuS	511145.b0642	GO Function	GO:0035639	Purine ribonucleoside triphosphate binding
leuS	511145.b0642	GO Function	GO:0036094	Small molecule binding
leuS	511145.b0642	GO Function	GO:0043167	Ion binding
leuS	511145.b0642	GO Function	GO:0043168	Anion binding
leuS	511145.b0642	GO Function	GO:0052689	Carboxylic ester hydrolase activity
leuS	511145.b0642	GO Function	GO:0097159	Organic cyclic compound binding
leuS	511145.b0642	GO Function	GO:0097367	Carbohydrate derivative binding
leuS	511145.b0642	GO Function	GO:0140098	Catalytic activity, acting on RNA
leuS	511145.b0642	GO Function	GO:0140101	Catalytic activity, acting on a tRNA
leuS	511145.b0642	GO Function	GO:0140640	Catalytic activity, acting on a nucleic acid
leuS	511145.b0642	GO Function	GO:1901265	Nucleoside phosphate binding
leuS	511145.b0642	GO Function	GO:1901363	Heterocyclic compound binding
leuS	511145.b0642	GO Component	GO:0005622	Intracellular anatomical structure
leuS	511145.b0642	GO Component	GO:0005737	Cytoplasm
leuS	511145.b0642	GO Component	GO:0005829	Cytosol
leuS	511145.b0642	GO Component	GO:0110165	Cellular anatomical entity
leuS	511145.b0642	STRING clusters	CL:309	Aminoacyl-tRNA synthetase
leuS	511145.b0642	STRING clusters	CL:311	Aminoacyl-tRNA synthetase
leuS	511145.b0642	STRING clusters	CL:314	Aminoacyl-tRNA synthetase
leuS	511145.b0642	STRING clusters	CL:318	Aminoacyl-tRNA synthetase, class Ia, anticodon-binding, and Lysine-tRNA ligase, class II
leuS	511145.b0642	STRING clusters	CL:35	Translation, and Protein export
leuS	511145.b0642	STRING clusters	CL:37	Translation, and Protein export
leuS	511145.b0642	KEGG	eco00970	Aminoacyl-tRNA biosynthesis
leuS	511145.b0642	COMPARTMENTS	GOCC:0005622	Intracellular
leuS	511145.b0642	COMPARTMENTS	GOCC:0005737	Cytoplasm
leuS	511145.b0642	COMPARTMENTS	GOCC:0005829	Cytosol
leuS	511145.b0642	COMPARTMENTS	GOCC:0110165	Cellular anatomical entity
leuS	511145.b0642	UniProt Keywords	KW-0030	Aminoacyl-tRNA synthetase
leuS	511145.b0642	UniProt Keywords	KW-0067	ATP-binding
leuS	511145.b0642	UniProt Keywords	KW-0436	Ligase
leuS	511145.b0642	UniProt Keywords	KW-0547	Nucleotide-binding
leuS	511145.b0642	UniProt Keywords	KW-0648	Protein biosynthesis
leuS	511145.b0642	UniProt Keywords	KW-0963	Cytoplasm
leuS	511145.b0642	Pfam	PF00133	tRNA synthetases class I (I, L, M and V)
leuS	511145.b0642	Pfam	PF08264	Anticodon-binding domain of tRNA ligase
leuS	511145.b0642	Pfam	PF13603	Leucyl-tRNA synthetase, Domain 2
leuS	511145.b0642	InterPro	IPR001412	Aminoacyl-tRNA synthetase, class I, conserved site
leuS	511145.b0642	InterPro	IPR002300	Aminoacyl-tRNA synthetase, class Ia
leuS	511145.b0642	InterPro	IPR002302	Leucine-tRNA ligase
leuS	511145.b0642	InterPro	IPR009008	Valyl/Leucyl/Isoleucyl-tRNA synthetase, editing domain
leuS	511145.b0642	InterPro	IPR009080	Aminoacyl-tRNA synthetase, class Ia, anticodon-binding
leuS	511145.b0642	InterPro	IPR013155	Methionyl/Valyl/Leucyl/Isoleucyl-tRNA synthetase, anticodon-binding
leuS	511145.b0642	InterPro	IPR014729	Rossmann-like alpha/beta/alpha sandwich fold
leuS	511145.b0642	InterPro	IPR025709	Leucyl-tRNA synthetase, editing domain
lipA	511145.b0628	GO Process	GO:0006082	Organic acid metabolic process
lipA	511145.b0628	GO Process	GO:0006629	Lipid metabolic process
lipA	511145.b0628	GO Process	GO:0006631	Fatty acid metabolic process
lipA	511145.b0628	GO Process	GO:0006633	Fatty acid biosynthetic process
lipA	511145.b0628	GO Process	GO:0006790	Sulfur compound metabolic process
lipA	511145.b0628	GO Process	GO:0006807	Nitrogen compound metabolic process
lipA	511145.b0628	GO Process	GO:0008152	Metabolic process
lipA	511145.b0628	GO Process	GO:0008610	Lipid biosynthetic process
lipA	511145.b0628	GO Process	GO:0009058	Biosynthetic process
lipA	511145.b0628	GO Process	GO:0009106	Lipoate metabolic process
lipA	511145.b0628	GO Process	GO:0009107	Lipoate biosynthetic process
lipA	511145.b0628	GO Process	GO:0009249	Protein lipoylation
lipA	511145.b0628	GO Process	GO:0009987	Cellular process
lipA	511145.b0628	GO Process	GO:0010467	Gene expression
lipA	511145.b0628	GO Process	GO:0016053	Organic acid biosynthetic process
lipA	511145.b0628	GO Process	GO:0018130	Heterocycle biosynthetic process
lipA	511145.b0628	GO Process	GO:0018193	Peptidyl-amino acid modification
lipA	511145.b0628	GO Process	GO:0018205	Peptidyl-lysine modification
lipA	511145.b0628	GO Process	GO:0019538	Protein metabolic process
lipA	511145.b0628	GO Process	GO:0019752	Carboxylic acid metabolic process
lipA	511145.b0628	GO Process	GO:0032787	Monocarboxylic acid metabolic process
lipA	511145.b0628	GO Process	GO:0036211	Protein modification process
lipA	511145.b0628	GO Process	GO:0043170	Macromolecule metabolic process
lipA	511145.b0628	GO Process	GO:0043412	Macromolecule modification
lipA	511145.b0628	GO Process	GO:0043436	Oxoacid metabolic process
lipA	511145.b0628	GO Process	GO:0044237	Cellular metabolic process
lipA	511145.b0628	GO Process	GO:0044238	Primary metabolic process
lipA	511145.b0628	GO Process	GO:0044249	Cellular biosynthetic process
lipA	511145.b0628	GO Process	GO:0044255	Cellular lipid metabolic process
lipA	511145.b0628	GO Process	GO:0044272	Sulfur compound biosynthetic process
lipA	511145.b0628	GO Process	GO:0044281	Small molecule metabolic process
lipA	511145.b0628	GO Process	GO:0044283	Small molecule biosynthetic process
lipA	511145.b0628	GO Process	GO:0046394	Carboxylic acid biosynthetic process
lipA	511145.b0628	GO Process	GO:0046483	Heterocycle metabolic process
lipA	511145.b0628	GO Process	GO:0051604	Protein maturation
lipA	511145.b0628	GO Process	GO:0071704	Organic substance metabolic process
lipA	511145.b0628	GO Process	GO:0072330	Monocarboxylic acid biosynthetic process
lipA	511145.b0628	GO Process	GO:1901360	Organic cyclic compound metabolic process
lipA	511145.b0628	GO Process	GO:1901362	Organic cyclic compound biosynthetic process
lipA	511145.b0628	GO Process	GO:1901564	Organonitrogen compound metabolic process
lipA	511145.b0628	GO Process	GO:1901576	Organic substance biosynthetic process
lipA	511145.b0628	GO Function	GO:0003824	Catalytic activity
lipA	511145.b0628	GO Function	GO:0005488	Binding
lipA	511145.b0628	GO Function	GO:0016740	Transferase activity
lipA	511145.b0628	GO Function	GO:0016782	Transferase activity, transferring sulphur-containing groups
lipA	511145.b0628	GO Function	GO:0016783	Sulfurtransferase activity
lipA	511145.b0628	GO Function	GO:0016992	Lipoate synthase activity
lipA	511145.b0628	GO Function	GO:0043167	Ion binding
lipA	511145.b0628	GO Function	GO:0043169	Cation binding
lipA	511145.b0628	GO Function	GO:0046872	Metal ion binding
lipA	511145.b0628	GO Function	GO:0051536	Iron-sulfur cluster binding
lipA	511145.b0628	GO Function	GO:0051539	4 iron, 4 sulfur cluster binding
lipA	511145.b0628	GO Function	GO:0051540	Metal cluster binding
lipA	511145.b0628	GO Function	GO:0102552	Lipoyl synthase activity (acting on glycine-cleavage complex H protein
lipA	511145.b0628	GO Function	GO:0102553	Lipoyl synthase activity (acting on pyruvate dehydrogenase E2 protein)
lipA	511145.b0628	GO Component	GO:0005622	Intracellular anatomical structure
lipA	511145.b0628	GO Component	GO:0005737	Cytoplasm
lipA	511145.b0628	GO Component	GO:0005829	Cytosol
lipA	511145.b0628	GO Component	GO:0110165	Cellular anatomical entity
lipA	511145.b0628	KEGG	eco00785	Lipoic acid metabolism
lipA	511145.b0628	KEGG	eco01100	Metabolic pathways
lipA	511145.b0628	COMPARTMENTS	GOCC:0005622	Intracellular
lipA	511145.b0628	COMPARTMENTS	GOCC:0005737	Cytoplasm
lipA	511145.b0628	COMPARTMENTS	GOCC:0005829	Cytosol
lipA	511145.b0628	COMPARTMENTS	GOCC:0110165	Cellular anatomical entity
lipA	511145.b0628	UniProt Keywords	KW-0004	4Fe-4S
lipA	511145.b0628	UniProt Keywords	KW-0408	Iron
lipA	511145.b0628	UniProt Keywords	KW-0411	Iron-sulfur
lipA	511145.b0628	UniProt Keywords	KW-0479	Metal-binding
lipA	511145.b0628	UniProt Keywords	KW-0808	Transferase
lipA	511145.b0628	UniProt Keywords	KW-0949	S-adenosyl-L-methionine
lipA	511145.b0628	UniProt Keywords	KW-0963	Cytoplasm
lipA	511145.b0628	Pfam	PF16881	N-terminal domain of lipoyl synthase of Radical_SAM family
lipA	511145.b0628	InterPro	IPR003698	Lipoyl synthase
lipA	511145.b0628	InterPro	IPR006638	Elp3/MiaA/NifB-like, radical SAM core domain
lipA	511145.b0628	InterPro	IPR007197	Radical SAM
lipA	511145.b0628	InterPro	IPR013785	Aldolase-type TIM barrel
lipA	511145.b0628	InterPro	IPR031691	Lipoyl synthase, N-terminal
lipA	511145.b0628	SMART	SM00729	Elongator protein 3, MiaB family, Radical SAM
lipB	511145.b0630	GO Process	GO:0006082	Organic acid metabolic process
lipB	511145.b0630	GO Process	GO:0006629	Lipid metabolic process
lipB	511145.b0630	GO Process	GO:0006631	Fatty acid metabolic process
lipB	511145.b0630	GO Process	GO:0006633	Fatty acid biosynthetic process
lipB	511145.b0630	GO Process	GO:0006790	Sulfur compound metabolic process
lipB	511145.b0630	GO Process	GO:0006807	Nitrogen compound metabolic process
lipB	511145.b0630	GO Process	GO:0008152	Metabolic process
lipB	511145.b0630	GO Process	GO:0008610	Lipid biosynthetic process
lipB	511145.b0630	GO Process	GO:0009058	Biosynthetic process
lipB	511145.b0630	GO Process	GO:0009106	Lipoate metabolic process
lipB	511145.b0630	GO Process	GO:0009107	Lipoate biosynthetic process
lipB	511145.b0630	GO Process	GO:0009249	Protein lipoylation
lipB	511145.b0630	GO Process	GO:0009892	Negative regulation of metabolic process
lipB	511145.b0630	GO Process	GO:0009987	Cellular process
lipB	511145.b0630	GO Process	GO:0010467	Gene expression
lipB	511145.b0630	GO Process	GO:0010468	Regulation of gene expression
lipB	511145.b0630	GO Process	GO:0010605	Negative regulation of macromolecule metabolic process
lipB	511145.b0630	GO Process	GO:0010629	Negative regulation of gene expression
lipB	511145.b0630	GO Process	GO:0016053	Organic acid biosynthetic process
lipB	511145.b0630	GO Process	GO:0018130	Heterocycle biosynthetic process
lipB	511145.b0630	GO Process	GO:0018193	Peptidyl-amino acid modification
lipB	511145.b0630	GO Process	GO:0018205	Peptidyl-lysine modification
lipB	511145.b0630	GO Process	GO:0019222	Regulation of metabolic process
lipB	511145.b0630	GO Process	GO:0019538	Protein metabolic process
lipB	511145.b0630	GO Process	GO:0019752	Carboxylic acid metabolic process
lipB	511145.b0630	GO Process	GO:0032787	Monocarboxylic acid metabolic process
lipB	511145.b0630	GO Process	GO:0036211	Protein modification process
lipB	511145.b0630	GO Process	GO:0043170	Macromolecule metabolic process
lipB	511145.b0630	GO Process	GO:0043412	Macromolecule modification
lipB	511145.b0630	GO Process	GO:0043436	Oxoacid metabolic process
lipB	511145.b0630	GO Process	GO:0044237	Cellular metabolic process
lipB	511145.b0630	GO Process	GO:0044238	Primary metabolic process
lipB	511145.b0630	GO Process	GO:0044249	Cellular biosynthetic process
lipB	511145.b0630	GO Process	GO:0044255	Cellular lipid metabolic process
lipB	511145.b0630	GO Process	GO:0044272	Sulfur compound biosynthetic process
lipB	511145.b0630	GO Process	GO:0044281	Small molecule metabolic process
lipB	511145.b0630	GO Process	GO:0044283	Small molecule biosynthetic process
lipB	511145.b0630	GO Process	GO:0046394	Carboxylic acid biosynthetic process
lipB	511145.b0630	GO Process	GO:0046483	Heterocycle metabolic process
lipB	511145.b0630	GO Process	GO:0048519	Negative regulation of biological process
lipB	511145.b0630	GO Process	GO:0050789	Regulation of biological process
lipB	511145.b0630	GO Process	GO:0051604	Protein maturation
lipB	511145.b0630	GO Process	GO:0060255	Regulation of macromolecule metabolic process
lipB	511145.b0630	GO Process	GO:0065007	Biological regulation
lipB	511145.b0630	GO Process	GO:0071704	Organic substance metabolic process
lipB	511145.b0630	GO Process	GO:0072330	Monocarboxylic acid biosynthetic process
lipB	511145.b0630	GO Process	GO:1901360	Organic cyclic compound metabolic process
lipB	511145.b0630	GO Process	GO:1901362	Organic cyclic compound biosynthetic process
lipB	511145.b0630	GO Process	GO:1901564	Organonitrogen compound metabolic process
lipB	511145.b0630	GO Process	GO:1901576	Organic substance biosynthetic process
lipB	511145.b0630	GO Function	GO:0003824	Catalytic activity
lipB	511145.b0630	GO Function	GO:0016415	Octanoyltransferase activity
lipB	511145.b0630	GO Function	GO:0016740	Transferase activity
lipB	511145.b0630	GO Function	GO:0016746	Acyltransferase activity
lipB	511145.b0630	GO Function	GO:0016747	Acyltransferase activity, transferring groups other than amino-acyl groups
lipB	511145.b0630	GO Function	GO:0033819	Lipoyl(octanoyl) transferase activity
lipB	511145.b0630	GO Function	GO:0140096	Catalytic activity, acting on a protein
lipB	511145.b0630	GO Component	GO:0005622	Intracellular anatomical structure
lipB	511145.b0630	GO Component	GO:0005737	Cytoplasm
lipB	511145.b0630	GO Component	GO:0110165	Cellular anatomical entity
lipB	511145.b0630	KEGG	eco00785	Lipoic acid metabolism
lipB	511145.b0630	KEGG	eco01100	Metabolic pathways
lipB	511145.b0630	UniProt Keywords	KW-0012	Acyltransferase
lipB	511145.b0630	UniProt Keywords	KW-0808	Transferase
lipB	511145.b0630	UniProt Keywords	KW-0963	Cytoplasm
lipB	511145.b0630	Pfam	PF03099	Biotin/lipoate A/B protein ligase family
lipB	511145.b0630	InterPro	IPR000544	Octanoyltransferase
lipB	511145.b0630	InterPro	IPR004143	Biotinyl protein ligase (BPL) and lipoyl protein ligase (LPL), catalytic domain
lipB	511145.b0630	InterPro	IPR020605	Octanoyltransferase, conserved site
lipB	511145.b0630	InterPro	IPR045864	Class II Aminoacyl-tRNA synthetase/Biotinyl protein ligase (BPL) and lipoyl protein ligase (LPL)
luxS	511145.b2687	GO Process	GO:0000096	Sulfur amino acid metabolic process
luxS	511145.b2687	GO Process	GO:0000097	Sulfur amino acid biosynthetic process
luxS	511145.b2687	GO Process	GO:0006082	Organic acid metabolic process
luxS	511145.b2687	GO Process	GO:0006520	Cellular amino acid metabolic process
luxS	511145.b2687	GO Process	GO:0006555	Methionine metabolic process
luxS	511145.b2687	GO Process	GO:0006790	Sulfur compound metabolic process
luxS	511145.b2687	GO Process	GO:0006807	Nitrogen compound metabolic process
luxS	511145.b2687	GO Process	GO:0007154	Cell communication
luxS	511145.b2687	GO Process	GO:0007267	Cell-cell signaling
luxS	511145.b2687	GO Process	GO:0008152	Metabolic process
luxS	511145.b2687	GO Process	GO:0008652	Cellular amino acid biosynthetic process
luxS	511145.b2687	GO Process	GO:0009058	Biosynthetic process
luxS	511145.b2687	GO Process	GO:0009066	Aspartate family amino acid metabolic process
luxS	511145.b2687	GO Process	GO:0009067	Aspartate family amino acid biosynthetic process
luxS	511145.b2687	GO Process	GO:0009086	Methionine biosynthetic process
luxS	511145.b2687	GO Process	GO:0009372	Quorum sensing
luxS	511145.b2687	GO Process	GO:0009595	Detection of biotic stimulus
luxS	511145.b2687	GO Process	GO:0009607	Response to biotic stimulus
luxS	511145.b2687	GO Process	GO:0009987	Cellular process
luxS	511145.b2687	GO Process	GO:0016053	Organic acid biosynthetic process
luxS	511145.b2687	GO Process	GO:0019284	L-methionine salvage from S-adenosylmethionine
luxS	511145.b2687	GO Process	GO:0019752	Carboxylic acid metabolic process
luxS	511145.b2687	GO Process	GO:0023052	Signaling
luxS	511145.b2687	GO Process	GO:0033353	S-adenosylmethionine cycle
luxS	511145.b2687	GO Process	GO:0043094	Cellular metabolic compound salvage
luxS	511145.b2687	GO Process	GO:0043102	Amino acid salvage
luxS	511145.b2687	GO Process	GO:0043436	Oxoacid metabolic process
luxS	511145.b2687	GO Process	GO:0044237	Cellular metabolic process
luxS	511145.b2687	GO Process	GO:0044238	Primary metabolic process
luxS	511145.b2687	GO Process	GO:0044249	Cellular biosynthetic process
luxS	511145.b2687	GO Process	GO:0044272	Sulfur compound biosynthetic process
luxS	511145.b2687	GO Process	GO:0044281	Small molecule metabolic process
luxS	511145.b2687	GO Process	GO:0044283	Small molecule biosynthetic process
luxS	511145.b2687	GO Process	GO:0046394	Carboxylic acid biosynthetic process
luxS	511145.b2687	GO Process	GO:0046500	S-adenosylmethionine metabolic process
luxS	511145.b2687	GO Process	GO:0050896	Response to stimulus
luxS	511145.b2687	GO Process	GO:0051606	Detection of stimulus
luxS	511145.b2687	GO Process	GO:0060245	Detection of cell density
luxS	511145.b2687	GO Process	GO:0071265	L-methionine biosynthetic process
luxS	511145.b2687	GO Process	GO:0071267	L-methionine salvage
luxS	511145.b2687	GO Process	GO:0071704	Organic substance metabolic process
luxS	511145.b2687	GO Process	GO:1901564	Organonitrogen compound metabolic process
luxS	511145.b2687	GO Process	GO:1901566	Organonitrogen compound biosynthetic process
luxS	511145.b2687	GO Process	GO:1901576	Organic substance biosynthetic process
luxS	511145.b2687	GO Process	GO:1901605	Alpha-amino acid metabolic process
luxS	511145.b2687	GO Process	GO:1901607	Alpha-amino acid biosynthetic process
luxS	511145.b2687	GO Function	GO:0003824	Catalytic activity
luxS	511145.b2687	GO Function	GO:0005488	Binding
luxS	511145.b2687	GO Function	GO:0005506	Iron ion binding
luxS	511145.b2687	GO Function	GO:0016829	Lyase activity
luxS	511145.b2687	GO Function	GO:0016846	Carbon-sulfur lyase activity
luxS	511145.b2687	GO Function	GO:0043167	Ion binding
luxS	511145.b2687	GO Function	GO:0043169	Cation binding
luxS	511145.b2687	GO Function	GO:0043768	S-ribosylhomocysteine lyase activity
luxS	511145.b2687	GO Function	GO:0046872	Metal ion binding
luxS	511145.b2687	GO Function	GO:0046914	Transition metal ion binding
luxS	511145.b2687	GO Component	GO:0005622	Intracellular anatomical structure
luxS	511145.b2687	GO Component	GO:0005737	Cytoplasm
luxS	511145.b2687	GO Component	GO:0005829	Cytosol
luxS	511145.b2687	GO Component	GO:0110165	Cellular anatomical entity
luxS	511145.b2687	STRING clusters	CL:4178	ATP-binding cassette (ABC) transporter complex
luxS	511145.b2687	STRING clusters	CL:4179	ATP-binding cassette (ABC) transporter complex
luxS	511145.b2687	STRING clusters	CL:4493	Mixed, incl. Quorum sensing, and Metabolite repair
luxS	511145.b2687	KEGG	eco00270	Cysteine and methionine metabolism
luxS	511145.b2687	KEGG	eco01100	Metabolic pathways
luxS	511145.b2687	KEGG	eco01230	Biosynthesis of amino acids
luxS	511145.b2687	KEGG	eco02024	Quorum sensing
luxS	511145.b2687	KEGG	eco02026	Biofilm formation - Escherichia coli
luxS	511145.b2687	COMPARTMENTS	GOCC:0005622	Intracellular
luxS	511145.b2687	COMPARTMENTS	GOCC:0005737	Cytoplasm
luxS	511145.b2687	COMPARTMENTS	GOCC:0005829	Cytosol
luxS	511145.b2687	COMPARTMENTS	GOCC:0110165	Cellular anatomical entity
luxS	511145.b2687	UniProt Keywords	KW-0071	Autoinducer synthesis
luxS	511145.b2687	UniProt Keywords	KW-0408	Iron
luxS	511145.b2687	UniProt Keywords	KW-0456	Lyase
luxS	511145.b2687	UniProt Keywords	KW-0479	Metal-binding
luxS	511145.b2687	UniProt Keywords	KW-0673	Quorum sensing
luxS	511145.b2687	Pfam	PF02664	S-Ribosylhomocysteinase (LuxS)
luxS	511145.b2687	InterPro	IPR003815	S-ribosylhomocysteinase (LuxS)
luxS	511145.b2687	InterPro	IPR011249	Metalloenzyme, LuxS/M16 peptidase-like
luxS	511145.b2687	InterPro	IPR037005	S-ribosylhomocysteinase (LuxS) superfamily
mdh	511145.b3236	GO Process	GO:0005975	Carbohydrate metabolic process
mdh	511145.b3236	GO Process	GO:0006082	Organic acid metabolic process
mdh	511145.b3236	GO Process	GO:0006090	Pyruvate metabolic process
mdh	511145.b3236	GO Process	GO:0006091	Generation of precursor metabolites and energy
mdh	511145.b3236	GO Process	GO:0006096	Glycolytic process
mdh	511145.b3236	GO Process	GO:0006099	Tricarboxylic acid cycle
mdh	511145.b3236	GO Process	GO:0006108	Malate metabolic process
mdh	511145.b3236	GO Process	GO:0006113	Fermentation
mdh	511145.b3236	GO Process	GO:0006139	Nucleobase-containing compound metabolic process
mdh	511145.b3236	GO Process	GO:0006163	Purine nucleotide metabolic process
mdh	511145.b3236	GO Process	GO:0006165	Nucleoside diphosphate phosphorylation
mdh	511145.b3236	GO Process	GO:0006725	Cellular aromatic compound metabolic process
mdh	511145.b3236	GO Process	GO:0006753	Nucleoside phosphate metabolic process
mdh	511145.b3236	GO Process	GO:0006757	ATP generation from ADP
mdh	511145.b3236	GO Process	GO:0006793	Phosphorus metabolic process
mdh	511145.b3236	GO Process	GO:0006796	Phosphate-containing compound metabolic process
mdh	511145.b3236	GO Process	GO:0006807	Nitrogen compound metabolic process
mdh	511145.b3236	GO Process	GO:0008152	Metabolic process
mdh	511145.b3236	GO Process	GO:0009056	Catabolic process
mdh	511145.b3236	GO Process	GO:0009060	Aerobic respiration
mdh	511145.b3236	GO Process	GO:0009061	Anaerobic respiration
mdh	511145.b3236	GO Process	GO:0009117	Nucleotide metabolic process
mdh	511145.b3236	GO Process	GO:0009132	Nucleoside diphosphate metabolic process
mdh	511145.b3236	GO Process	GO:0009135	Purine nucleoside diphosphate metabolic process
mdh	511145.b3236	GO Process	GO:0009141	Nucleoside triphosphate metabolic process
mdh	511145.b3236	GO Process	GO:0009144	Purine nucleoside triphosphate metabolic process
mdh	511145.b3236	GO Process	GO:0009150	Purine ribonucleotide metabolic process
mdh	511145.b3236	GO Process	GO:0009179	Purine ribonucleoside diphosphate metabolic process
mdh	511145.b3236	GO Process	GO:0009185	Ribonucleoside diphosphate metabolic process
mdh	511145.b3236	GO Process	GO:0009199	Ribonucleoside triphosphate metabolic process
mdh	511145.b3236	GO Process	GO:0009205	Purine ribonucleoside triphosphate metabolic process
mdh	511145.b3236	GO Process	GO:0009259	Ribonucleotide metabolic process
mdh	511145.b3236	GO Process	GO:0009987	Cellular process
mdh	511145.b3236	GO Process	GO:0015980	Energy derivation by oxidation of organic compounds
mdh	511145.b3236	GO Process	GO:0016052	Carbohydrate catabolic process
mdh	511145.b3236	GO Process	GO:0016310	Phosphorylation
mdh	511145.b3236	GO Process	GO:0019637	Organophosphate metabolic process
mdh	511145.b3236	GO Process	GO:0019693	Ribose phosphate metabolic process
mdh	511145.b3236	GO Process	GO:0019752	Carboxylic acid metabolic process
mdh	511145.b3236	GO Process	GO:0032787	Monocarboxylic acid metabolic process
mdh	511145.b3236	GO Process	GO:0034641	Cellular nitrogen compound metabolic process
mdh	511145.b3236	GO Process	GO:0043436	Oxoacid metabolic process
mdh	511145.b3236	GO Process	GO:0043648	Dicarboxylic acid metabolic process
mdh	511145.b3236	GO Process	GO:0044237	Cellular metabolic process
mdh	511145.b3236	GO Process	GO:0044238	Primary metabolic process
mdh	511145.b3236	GO Process	GO:0044281	Small molecule metabolic process
mdh	511145.b3236	GO Process	GO:0045333	Cellular respiration
mdh	511145.b3236	GO Process	GO:0046031	ADP metabolic process
mdh	511145.b3236	GO Process	GO:0046034	ATP metabolic process
mdh	511145.b3236	GO Process	GO:0046483	Heterocycle metabolic process
mdh	511145.b3236	GO Process	GO:0046939	Nucleotide phosphorylation
mdh	511145.b3236	GO Process	GO:0055086	Nucleobase-containing small molecule metabolic process
mdh	511145.b3236	GO Process	GO:0071704	Organic substance metabolic process
mdh	511145.b3236	GO Process	GO:0072521	Purine-containing compound metabolic process
mdh	511145.b3236	GO Process	GO:1901135	Carbohydrate derivative metabolic process
mdh	511145.b3236	GO Process	GO:1901360	Organic cyclic compound metabolic process
mdh	511145.b3236	GO Process	GO:1901564	Organonitrogen compound metabolic process
mdh	511145.b3236	GO Process	GO:1901575	Organic substance catabolic process
mdh	511145.b3236	GO Function	GO:0003824	Catalytic activity
mdh	511145.b3236	GO Function	GO:0016491	Oxidoreductase activity
mdh	511145.b3236	GO Function	GO:0016614	Oxidoreductase activity, acting on CH-OH group of donors
mdh	511145.b3236	GO Function	GO:0016615	Malate dehydrogenase activity
mdh	511145.b3236	GO Function	GO:0016616	Oxidoreductase activity, acting on the CH-OH group of donors, NAD or NADP as acceptor
mdh	511145.b3236	GO Function	GO:0030060	L-malate dehydrogenase activity
mdh	511145.b3236	GO Component	GO:0005622	Intracellular anatomical structure
mdh	511145.b3236	GO Component	GO:0005737	Cytoplasm
mdh	511145.b3236	GO Component	GO:0005829	Cytosol
mdh	511145.b3236	GO Component	GO:0016020	Membrane
mdh	511145.b3236	GO Component	GO:0019898	Extrinsic component of membrane
mdh	511145.b3236	GO Component	GO:0110165	Cellular anatomical entity
mdh	511145.b3236	STRING clusters	CL:2010	Mixed, incl. Carbon metabolism, and Fatty acid metabolic process
mdh	511145.b3236	STRING clusters	CL:2011	Carbon metabolism, and Oxidative phosphorylation
mdh	511145.b3236	STRING clusters	CL:2012	Carbon metabolism, and Pyruvate metabolism
mdh	511145.b3236	STRING clusters	CL:2013	Carbon metabolism, and Formate C-acetyltransferase activity
mdh	511145.b3236	STRING clusters	CL:2014	Citrate cycle (TCA cycle), and Pyruvate metabolism
mdh	511145.b3236	STRING clusters	CL:2062	Citrate cycle (TCA cycle)
mdh	511145.b3236	STRING clusters	CL:2097	Malate dehydrogenase activity, and Fumarate hydratase activity
mdh	511145.b3236	KEGG	eco00020	Citrate cycle (TCA cycle)
mdh	511145.b3236	KEGG	eco00270	Cysteine and methionine metabolism
mdh	511145.b3236	KEGG	eco00620	Pyruvate metabolism
mdh	511145.b3236	KEGG	eco00630	Glyoxylate and dicarboxylate metabolism
mdh	511145.b3236	KEGG	eco00680	Methane metabolism
mdh	511145.b3236	KEGG	eco01100	Metabolic pathways
mdh	511145.b3236	KEGG	eco01110	Biosynthesis of secondary metabolites
mdh	511145.b3236	KEGG	eco01120	Microbial metabolism in diverse environments
mdh	511145.b3236	KEGG	eco01200	Carbon metabolism
mdh	511145.b3236	COMPARTMENTS	GOCC:0005622	Intracellular
mdh	511145.b3236	COMPARTMENTS	GOCC:0005737	Cytoplasm
mdh	511145.b3236	COMPARTMENTS	GOCC:0005829	Cytosol
mdh	511145.b3236	COMPARTMENTS	GOCC:0016020	Membrane
mdh	511145.b3236	COMPARTMENTS	GOCC:0019898	Extrinsic component of membrane
mdh	511145.b3236	COMPARTMENTS	GOCC:0110165	Cellular anatomical entity
mdh	511145.b3236	UniProt Keywords	KW-0520	NAD
mdh	511145.b3236	UniProt Keywords	KW-0560	Oxidoreductase
mdh	511145.b3236	UniProt Keywords	KW-0816	Tricarboxylic acid cycle
mdh	511145.b3236	Pfam	PF00056	Lactate/malate dehydrogenase, NAD binding domain
mdh	511145.b3236	Pfam	PF02866	Lactate/malate dehydrogenase, alpha/beta C-terminal domain
mdh	511145.b3236	InterPro	IPR001236	Lactate/malate dehydrogenase, N-terminal
mdh	511145.b3236	InterPro	IPR001252	Malate dehydrogenase, active site
mdh	511145.b3236	InterPro	IPR001557	L-lactate/malate dehydrogenase
mdh	511145.b3236	InterPro	IPR010097	Malate dehydrogenase, type 1
mdh	511145.b3236	InterPro	IPR015955	Lactate dehydrogenase/glycoside hydrolase, family 4, C-terminal
mdh	511145.b3236	InterPro	IPR022383	Lactate/malate dehydrogenase, C-terminal
mdh	511145.b3236	InterPro	IPR023958	Malate dehydrogenase, type 1, bacterial
mdh	511145.b3236	InterPro	IPR036291	NAD(P)-binding domain superfamily
menH	511145.b2263	GO Process	GO:0008152	Metabolic process
menH	511145.b2263	GO Process	GO:0009058	Biosynthetic process
menH	511145.b2263	GO Process	GO:0009233	Menaquinone metabolic process
menH	511145.b2263	GO Process	GO:0009234	Menaquinone biosynthetic process
menH	511145.b2263	GO Process	GO:0009987	Cellular process
menH	511145.b2263	GO Process	GO:0042180	Cellular ketone metabolic process
menH	511145.b2263	GO Process	GO:0042181	Ketone biosynthetic process
menH	511145.b2263	GO Process	GO:0044237	Cellular metabolic process
menH	511145.b2263	GO Process	GO:0044249	Cellular biosynthetic process
menH	511145.b2263	GO Process	GO:0044281	Small molecule metabolic process
menH	511145.b2263	GO Process	GO:0044283	Small molecule biosynthetic process
menH	511145.b2263	GO Process	GO:0071704	Organic substance metabolic process
menH	511145.b2263	GO Process	GO:1901576	Organic substance biosynthetic process
menH	511145.b2263	GO Process	GO:1901661	Quinone metabolic process
menH	511145.b2263	GO Process	GO:1901663	Quinone biosynthetic process
menH	511145.b2263	GO Function	GO:0003824	Catalytic activity
menH	511145.b2263	GO Function	GO:0016829	Lyase activity
menH	511145.b2263	GO Function	GO:0016835	Carbon-oxygen lyase activity
menH	511145.b2263	GO Function	GO:0070205	2-succinyl-6-hydroxy-2,4-cyclohexadiene-1-carboxylate synthase activity
menH	511145.b2263	GO Component	GO:0005622	Intracellular anatomical structure
menH	511145.b2263	GO Component	GO:0005737	Cytoplasm
menH	511145.b2263	GO Component	GO:0005829	Cytosol
menH	511145.b2263	GO Component	GO:0110165	Cellular anatomical entity
menH	511145.b2263	STRING clusters	CL:2534	Mixed, incl. Quinone metabolic process, and Tetrapyrrole biosynthetic process
menH	511145.b2263	STRING clusters	CL:2638	Isoprene biosynthesis, and Menaquinone biosynthesis
menH	511145.b2263	STRING clusters	CL:2639	Mixed, incl. Menaquinone biosynthesis, and Peptidase M28
menH	511145.b2263	STRING clusters	CL:2640	Menaquinone biosynthesis
menH	511145.b2263	STRING clusters	CL:2643	Menaquinone biosynthesis
menH	511145.b2263	KEGG	eco00130	Ubiquinone and other terpenoid-quinone biosynthesis
menH	511145.b2263	KEGG	eco01100	Metabolic pathways
menH	511145.b2263	KEGG	eco01110	Biosynthesis of secondary metabolites
menH	511145.b2263	COMPARTMENTS	GOCC:0005622	Intracellular
menH	511145.b2263	COMPARTMENTS	GOCC:0005737	Cytoplasm
menH	511145.b2263	COMPARTMENTS	GOCC:0005829	Cytosol
menH	511145.b2263	COMPARTMENTS	GOCC:0110165	Cellular anatomical entity
menH	511145.b2263	UniProt Keywords	KW-0456	Lyase
menH	511145.b2263	UniProt Keywords	KW-0474	Menaquinone biosynthesis
menH	511145.b2263	Pfam	PF12146	Serine aminopeptidase, S33
menH	511145.b2263	InterPro	IPR000073	Alpha/beta hydrolase fold-1
menH	511145.b2263	InterPro	IPR022485	2-succinyl-6-hydroxy-2,4-cyclohexadiene-1-carboxylate synthase
menH	511145.b2263	InterPro	IPR029058	Alpha/Beta hydrolase fold
mhpD	511145.b0350	GO Process	GO:0006082	Organic acid metabolic process
mhpD	511145.b0350	GO Process	GO:0006725	Cellular aromatic compound metabolic process
mhpD	511145.b0350	GO Process	GO:0006805	Xenobiotic metabolic process
mhpD	511145.b0350	GO Process	GO:0008152	Metabolic process
mhpD	511145.b0350	GO Process	GO:0009056	Catabolic process
mhpD	511145.b0350	GO Process	GO:0009410	Response to xenobiotic stimulus
mhpD	511145.b0350	GO Process	GO:0009987	Cellular process
mhpD	511145.b0350	GO Process	GO:0016054	Organic acid catabolic process
mhpD	511145.b0350	GO Process	GO:0018962	3-phenylpropionate metabolic process
mhpD	511145.b0350	GO Process	GO:0019380	3-phenylpropionate catabolic process
mhpD	511145.b0350	GO Process	GO:0019439	Aromatic compound catabolic process
mhpD	511145.b0350	GO Process	GO:0019752	Carboxylic acid metabolic process
mhpD	511145.b0350	GO Process	GO:0032787	Monocarboxylic acid metabolic process
mhpD	511145.b0350	GO Process	GO:0042178	Xenobiotic catabolic process
mhpD	511145.b0350	GO Process	GO:0042221	Response to chemical
mhpD	511145.b0350	GO Process	GO:0042537	Benzene-containing compound metabolic process
mhpD	511145.b0350	GO Process	GO:0043436	Oxoacid metabolic process
mhpD	511145.b0350	GO Process	GO:0044237	Cellular metabolic process
mhpD	511145.b0350	GO Process	GO:0044248	Cellular catabolic process
mhpD	511145.b0350	GO Process	GO:0044281	Small molecule metabolic process
mhpD	511145.b0350	GO Process	GO:0044282	Small molecule catabolic process
mhpD	511145.b0350	GO Process	GO:0046395	Carboxylic acid catabolic process
mhpD	511145.b0350	GO Process	GO:0050896	Response to stimulus
mhpD	511145.b0350	GO Process	GO:0051716	Cellular response to stimulus
mhpD	511145.b0350	GO Process	GO:0070887	Cellular response to chemical stimulus
mhpD	511145.b0350	GO Process	GO:0071466	Cellular response to xenobiotic stimulus
mhpD	511145.b0350	GO Process	GO:0071704	Organic substance metabolic process
mhpD	511145.b0350	GO Process	GO:0072329	Monocarboxylic acid catabolic process
mhpD	511145.b0350	GO Process	GO:1901360	Organic cyclic compound metabolic process
mhpD	511145.b0350	GO Process	GO:1901361	Organic cyclic compound catabolic process
mhpD	511145.b0350	GO Process	GO:1901575	Organic substance catabolic process
mhpD	511145.b0350	GO Function	GO:0003824	Catalytic activity
mhpD	511145.b0350	GO Function	GO:0005488	Binding
mhpD	511145.b0350	GO Function	GO:0005515	Protein binding
mhpD	511145.b0350	GO Function	GO:0008684	2-oxopent-4-enoate hydratase activity
mhpD	511145.b0350	GO Function	GO:0016829	Lyase activity
mhpD	511145.b0350	GO Function	GO:0016835	Carbon-oxygen lyase activity
mhpD	511145.b0350	GO Function	GO:0016836	Hydro-lyase activity
mhpD	511145.b0350	GO Function	GO:0030145	Manganese ion binding
mhpD	511145.b0350	GO Function	GO:0042802	Identical protein binding
mhpD	511145.b0350	GO Function	GO:0043167	Ion binding
mhpD	511145.b0350	GO Function	GO:0043169	Cation binding
mhpD	511145.b0350	GO Function	GO:0046872	Metal ion binding
mhpD	511145.b0350	GO Function	GO:0046914	Transition metal ion binding
mhpD	511145.b0350	GO Component	GO:0005622	Intracellular anatomical structure
mhpD	511145.b0350	GO Component	GO:0005737	Cytoplasm
mhpD	511145.b0350	GO Component	GO:0110165	Cellular anatomical entity
mhpD	511145.b0350	STRING clusters	CL:6207	Mostly uncharacterized, incl. Degradation of aromatic compounds, and Uracil catabolic process
mhpD	511145.b0350	STRING clusters	CL:6208	Mostly uncharacterized, incl. 3-phenylpropionate catabolic process, and Uracil catabolic process
mhpD	511145.b0350	STRING clusters	CL:6209	Mixed, incl. 3-phenylpropionate catabolic process, and Uracil catabolic process
mhpD	511145.b0350	STRING clusters	CL:6210	Mixed, incl. 3-phenylpropionate catabolic process, and DNA dealkylation
mhpD	511145.b0350	STRING clusters	CL:6211	Mixed, incl. 3-phenylpropionate catabolic process, and Nitric oxide metabolic process
mhpD	511145.b0350	STRING clusters	CL:6212	3-phenylpropionate catabolic process
mhpD	511145.b0350	STRING clusters	CL:6226	3-phenylpropionate catabolic process
mhpD	511145.b0350	KEGG	eco00360	Phenylalanine metabolism
mhpD	511145.b0350	KEGG	eco00362	Benzoate degradation
mhpD	511145.b0350	KEGG	eco00621	Dioxin degradation
mhpD	511145.b0350	KEGG	eco00622	Xylene degradation
mhpD	511145.b0350	KEGG	eco01100	Metabolic pathways
mhpD	511145.b0350	KEGG	eco01120	Microbial metabolism in diverse environments
mhpD	511145.b0350	KEGG	eco01220	Degradation of aromatic compounds
mhpD	511145.b0350	UniProt Keywords	KW-0058	Aromatic hydrocarbons catabolism
mhpD	511145.b0350	UniProt Keywords	KW-0456	Lyase
mhpD	511145.b0350	Pfam	PF01557	Fumarylacetoacetate (FAA) hydrolase family
mhpD	511145.b0350	InterPro	IPR011234	Fumarylacetoacetase-like, C-terminal
mhpD	511145.b0350	InterPro	IPR023793	2-keto-4-pentenoate hydratase
mhpD	511145.b0350	InterPro	IPR036663	Fumarylacetoacetase-like, C-terminal domain superfamily
miaA	511145.b4171	GO Process	GO:0006139	Nucleobase-containing compound metabolic process
miaA	511145.b4171	GO Process	GO:0006396	RNA processing
miaA	511145.b4171	GO Process	GO:0006399	tRNA metabolic process
miaA	511145.b4171	GO Process	GO:0006400	tRNA modification
miaA	511145.b4171	GO Process	GO:0006417	Regulation of translation
miaA	511145.b4171	GO Process	GO:0006725	Cellular aromatic compound metabolic process
miaA	511145.b4171	GO Process	GO:0006807	Nitrogen compound metabolic process
miaA	511145.b4171	GO Process	GO:0006950	Response to stress
miaA	511145.b4171	GO Process	GO:0008033	tRNA processing
miaA	511145.b4171	GO Process	GO:0008152	Metabolic process
miaA	511145.b4171	GO Process	GO:0009266	Response to temperature stimulus
miaA	511145.b4171	GO Process	GO:0009408	Response to heat
miaA	511145.b4171	GO Process	GO:0009451	RNA modification
miaA	511145.b4171	GO Process	GO:0009628	Response to abiotic stimulus
miaA	511145.b4171	GO Process	GO:0009889	Regulation of biosynthetic process
miaA	511145.b4171	GO Process	GO:0009987	Cellular process
miaA	511145.b4171	GO Process	GO:0010467	Gene expression
miaA	511145.b4171	GO Process	GO:0010468	Regulation of gene expression
miaA	511145.b4171	GO Process	GO:0010556	Regulation of macromolecule biosynthetic process
miaA	511145.b4171	GO Process	GO:0010608	Post-transcriptional regulation of gene expression
miaA	511145.b4171	GO Process	GO:0016070	RNA metabolic process
miaA	511145.b4171	GO Process	GO:0019222	Regulation of metabolic process
miaA	511145.b4171	GO Process	GO:0031323	Regulation of cellular metabolic process
miaA	511145.b4171	GO Process	GO:0031326	Regulation of cellular biosynthetic process
miaA	511145.b4171	GO Process	GO:0033554	Cellular response to stress
miaA	511145.b4171	GO Process	GO:0034248	Regulation of cellular amide metabolic process
miaA	511145.b4171	GO Process	GO:0034470	ncRNA processing
miaA	511145.b4171	GO Process	GO:0034605	Cellular response to heat
miaA	511145.b4171	GO Process	GO:0034641	Cellular nitrogen compound metabolic process
miaA	511145.b4171	GO Process	GO:0034660	ncRNA metabolic process
miaA	511145.b4171	GO Process	GO:0043170	Macromolecule metabolic process
miaA	511145.b4171	GO Process	GO:0043412	Macromolecule modification
miaA	511145.b4171	GO Process	GO:0043555	Regulation of translation in response to stress
miaA	511145.b4171	GO Process	GO:0044237	Cellular metabolic process
miaA	511145.b4171	GO Process	GO:0044238	Primary metabolic process
miaA	511145.b4171	GO Process	GO:0046483	Heterocycle metabolic process
miaA	511145.b4171	GO Process	GO:0050789	Regulation of biological process
miaA	511145.b4171	GO Process	GO:0050794	Regulation of cellular process
miaA	511145.b4171	GO Process	GO:0050896	Response to stimulus
miaA	511145.b4171	GO Process	GO:0051171	Regulation of nitrogen compound metabolic process
miaA	511145.b4171	GO Process	GO:0051246	Regulation of protein metabolic process
miaA	511145.b4171	GO Process	GO:0051716	Cellular response to stimulus
miaA	511145.b4171	GO Process	GO:0060255	Regulation of macromolecule metabolic process
miaA	511145.b4171	GO Process	GO:0065007	Biological regulation
miaA	511145.b4171	GO Process	GO:0071704	Organic substance metabolic process
miaA	511145.b4171	GO Process	GO:0080090	Regulation of primary metabolic process
miaA	511145.b4171	GO Process	GO:0090304	Nucleic acid metabolic process
miaA	511145.b4171	GO Process	GO:1901360	Organic cyclic compound metabolic process
miaA	511145.b4171	GO Process	GO:1990497	Regulation of cytoplasmic translation in response to stress
miaA	511145.b4171	GO Process	GO:2000112	Regulation of cellular macromolecule biosynthetic process
miaA	511145.b4171	GO Process	GO:2000765	Regulation of cytoplasmic translation
miaA	511145.b4171	GO Function	GO:0000166	Nucleotide binding
miaA	511145.b4171	GO Function	GO:0003824	Catalytic activity
miaA	511145.b4171	GO Function	GO:0005488	Binding
miaA	511145.b4171	GO Function	GO:0005524	ATP binding
miaA	511145.b4171	GO Function	GO:0016740	Transferase activity
miaA	511145.b4171	GO Function	GO:0016765	Transferase activity, transferring alkyl or aryl (other than methyl) groups
miaA	511145.b4171	GO Function	GO:0017076	Purine nucleotide binding
miaA	511145.b4171	GO Function	GO:0030554	Adenyl nucleotide binding
miaA	511145.b4171	GO Function	GO:0032553	Ribonucleotide binding
miaA	511145.b4171	GO Function	GO:0032555	Purine ribonucleotide binding
miaA	511145.b4171	GO Function	GO:0032559	Adenyl ribonucleotide binding
miaA	511145.b4171	GO Function	GO:0035639	Purine ribonucleoside triphosphate binding
miaA	511145.b4171	GO Function	GO:0036094	Small molecule binding
miaA	511145.b4171	GO Function	GO:0043167	Ion binding
miaA	511145.b4171	GO Function	GO:0043168	Anion binding
miaA	511145.b4171	GO Function	GO:0052381	tRNA dimethylallyltransferase activity
miaA	511145.b4171	GO Function	GO:0097159	Organic cyclic compound binding
miaA	511145.b4171	GO Function	GO:0097367	Carbohydrate derivative binding
miaA	511145.b4171	GO Function	GO:0140098	Catalytic activity, acting on RNA
miaA	511145.b4171	GO Function	GO:0140101	Catalytic activity, acting on a tRNA
miaA	511145.b4171	GO Function	GO:0140640	Catalytic activity, acting on a nucleic acid
miaA	511145.b4171	GO Function	GO:1901265	Nucleoside phosphate binding
miaA	511145.b4171	GO Function	GO:1901363	Heterocyclic compound binding
miaA	511145.b4171	STRING clusters	CL:594	Mixed, incl. Response to heat, and DnaJ domain
miaA	511145.b4171	STRING clusters	CL:596	Response to heat, and DnaJ domain
miaA	511145.b4171	STRING clusters	CL:654	Mixed, incl. Regulation of proteolysis, and AAA domain
miaA	511145.b4171	STRING clusters	CL:668	Mixed, incl. AAA domain, and TRAM domain
miaA	511145.b4171	KEGG	eco01100	Metabolic pathways
miaA	511145.b4171	KEGG	eco01110	Biosynthesis of secondary metabolites
miaA	511145.b4171	UniProt Keywords	KW-0067	ATP-binding
miaA	511145.b4171	UniProt Keywords	KW-0460	Magnesium
miaA	511145.b4171	UniProt Keywords	KW-0547	Nucleotide-binding
miaA	511145.b4171	UniProt Keywords	KW-0808	Transferase
miaA	511145.b4171	UniProt Keywords	KW-0819	tRNA processing
miaA	511145.b4171	Pfam	PF01715	IPP transferase
miaA	511145.b4171	InterPro	IPR018022	IPP transferase
miaA	511145.b4171	InterPro	IPR027417	P-loop containing nucleoside triphosphate hydrolase
miaA	511145.b4171	InterPro	IPR039657	Dimethylallyltransferase
mnmA	511145.b1133	GO Process	GO:0002097	tRNA wobble base modification
mnmA	511145.b1133	GO Process	GO:0002098	tRNA wobble uridine modification
mnmA	511145.b1133	GO Process	GO:0002143	tRNA wobble position uridine thiolation
mnmA	511145.b1133	GO Process	GO:0006139	Nucleobase-containing compound metabolic process
mnmA	511145.b1133	GO Process	GO:0006396	RNA processing
mnmA	511145.b1133	GO Process	GO:0006399	tRNA metabolic process
mnmA	511145.b1133	GO Process	GO:0006400	tRNA modification
mnmA	511145.b1133	GO Process	GO:0006725	Cellular aromatic compound metabolic process
mnmA	511145.b1133	GO Process	GO:0006807	Nitrogen compound metabolic process
mnmA	511145.b1133	GO Process	GO:0008033	tRNA processing
mnmA	511145.b1133	GO Process	GO:0008152	Metabolic process
mnmA	511145.b1133	GO Process	GO:0009451	RNA modification
mnmA	511145.b1133	GO Process	GO:0009987	Cellular process
mnmA	511145.b1133	GO Process	GO:0010467	Gene expression
mnmA	511145.b1133	GO Process	GO:0016070	RNA metabolic process
mnmA	511145.b1133	GO Process	GO:0034227	tRNA thio-modification
mnmA	511145.b1133	GO Process	GO:0034470	ncRNA processing
mnmA	511145.b1133	GO Process	GO:0034641	Cellular nitrogen compound metabolic process
mnmA	511145.b1133	GO Process	GO:0034660	ncRNA metabolic process
mnmA	511145.b1133	GO Process	GO:0043170	Macromolecule metabolic process
mnmA	511145.b1133	GO Process	GO:0043412	Macromolecule modification
mnmA	511145.b1133	GO Process	GO:0044237	Cellular metabolic process
mnmA	511145.b1133	GO Process	GO:0044238	Primary metabolic process
mnmA	511145.b1133	GO Process	GO:0046483	Heterocycle metabolic process
mnmA	511145.b1133	GO Process	GO:0071704	Organic substance metabolic process
mnmA	511145.b1133	GO Process	GO:0090304	Nucleic acid metabolic process
mnmA	511145.b1133	GO Process	GO:1901360	Organic cyclic compound metabolic process
mnmA	511145.b1133	GO Function	GO:0000049	tRNA binding
mnmA	511145.b1133	GO Function	GO:0000166	Nucleotide binding
mnmA	511145.b1133	GO Function	GO:0003676	Nucleic acid binding
mnmA	511145.b1133	GO Function	GO:0003723	RNA binding
mnmA	511145.b1133	GO Function	GO:0003824	Catalytic activity
mnmA	511145.b1133	GO Function	GO:0005488	Binding
mnmA	511145.b1133	GO Function	GO:0005524	ATP binding
mnmA	511145.b1133	GO Function	GO:0016740	Transferase activity
mnmA	511145.b1133	GO Function	GO:0016782	Transferase activity, transferring sulphur-containing groups
mnmA	511145.b1133	GO Function	GO:0016783	Sulfurtransferase activity
mnmA	511145.b1133	GO Function	GO:0017076	Purine nucleotide binding
mnmA	511145.b1133	GO Function	GO:0030554	Adenyl nucleotide binding
mnmA	511145.b1133	GO Function	GO:0032553	Ribonucleotide binding
mnmA	511145.b1133	GO Function	GO:0032555	Purine ribonucleotide binding
mnmA	511145.b1133	GO Function	GO:0032559	Adenyl ribonucleotide binding
mnmA	511145.b1133	GO Function	GO:0035639	Purine ribonucleoside triphosphate binding
mnmA	511145.b1133	GO Function	GO:0036094	Small molecule binding
mnmA	511145.b1133	GO Function	GO:0043167	Ion binding
mnmA	511145.b1133	GO Function	GO:0043168	Anion binding
mnmA	511145.b1133	GO Function	GO:0097159	Organic cyclic compound binding
mnmA	511145.b1133	GO Function	GO:0097367	Carbohydrate derivative binding
mnmA	511145.b1133	GO Function	GO:1901265	Nucleoside phosphate binding
mnmA	511145.b1133	GO Function	GO:1901363	Heterocyclic compound binding
mnmA	511145.b1133	GO Component	GO:0005622	Intracellular anatomical structure
mnmA	511145.b1133	GO Component	GO:0005737	Cytoplasm
mnmA	511145.b1133	GO Component	GO:0005829	Cytosol
mnmA	511145.b1133	GO Component	GO:0032991	Protein-containing complex
mnmA	511145.b1133	GO Component	GO:0110165	Cellular anatomical entity
mnmA	511145.b1133	GO Component	GO:1902494	Catalytic complex
mnmA	511145.b1133	GO Component	GO:1990228	Sulfurtransferase complex
mnmA	511145.b1133	GO Component	GO:1990234	Transferase complex
mnmA	511145.b1133	STRING clusters	CL:1242	Mixed, incl. Iron-sulfur cluster assembly, and Antioxidant activity
mnmA	511145.b1133	STRING clusters	CL:1243	Mixed, incl. Iron-sulfur cluster assembly, and Antioxidant activity
mnmA	511145.b1133	STRING clusters	CL:1327	Iron-sulfur cluster assembly, and tRNA thio-modification
mnmA	511145.b1133	STRING clusters	CL:1368	tRNA wobble position uridine thiolation
mnmA	511145.b1133	KEGG	eco04122	Sulfur relay system
mnmA	511145.b1133	COMPARTMENTS	GOCC:0005622	Intracellular
mnmA	511145.b1133	COMPARTMENTS	GOCC:0005737	Cytoplasm
mnmA	511145.b1133	COMPARTMENTS	GOCC:0005829	Cytosol
mnmA	511145.b1133	COMPARTMENTS	GOCC:0110165	Cellular anatomical entity
mnmA	511145.b1133	UniProt Keywords	KW-0067	ATP-binding
mnmA	511145.b1133	UniProt Keywords	KW-0547	Nucleotide-binding
mnmA	511145.b1133	UniProt Keywords	KW-0694	RNA-binding
mnmA	511145.b1133	UniProt Keywords	KW-0808	Transferase
mnmA	511145.b1133	UniProt Keywords	KW-0819	tRNA processing
mnmA	511145.b1133	UniProt Keywords	KW-0820	tRNA-binding
mnmA	511145.b1133	UniProt Keywords	KW-0963	Cytoplasm
mnmA	511145.b1133	UniProt Keywords	KW-1015	Disulfide bond
mnmA	511145.b1133	Pfam	PF02568	Thiamine biosynthesis protein (ThiI)
mnmA	511145.b1133	InterPro	IPR004506	tRNA-specific 2-thiouridylase
mnmA	511145.b1133	InterPro	IPR014729	Rossmann-like alpha/beta/alpha sandwich fold
mnmA	511145.b1133	InterPro	IPR023382	tRNA-specific 2-thiouridylase MnmA-like, central domain superfamily
mnmA	511145.b1133	InterPro	IPR046884	tRNA-specific 2-thiouridylase MnmA-like, central domain
mnmA	511145.b1133	InterPro	IPR046885	tRNA-specific 2-thiouridylase MnmA-like, C-terminal domain
moaA	511145.b0781	GO Process	GO:0006777	Mo-molybdopterin cofactor biosynthetic process
moaA	511145.b0781	GO Process	GO:0006793	Phosphorus metabolic process
moaA	511145.b0781	GO Process	GO:0006796	Phosphate-containing compound metabolic process
moaA	511145.b0781	GO Process	GO:0006807	Nitrogen compound metabolic process
moaA	511145.b0781	GO Process	GO:0006950	Response to stress
moaA	511145.b0781	GO Process	GO:0008152	Metabolic process
moaA	511145.b0781	GO Process	GO:0009058	Biosynthetic process
moaA	511145.b0781	GO Process	GO:0009266	Response to temperature stimulus
moaA	511145.b0781	GO Process	GO:0009408	Response to heat
moaA	511145.b0781	GO Process	GO:0009628	Response to abiotic stimulus
moaA	511145.b0781	GO Process	GO:0009987	Cellular process
moaA	511145.b0781	GO Process	GO:0018130	Heterocycle biosynthetic process
moaA	511145.b0781	GO Process	GO:0019538	Protein metabolic process
moaA	511145.b0781	GO Process	GO:0019637	Organophosphate metabolic process
moaA	511145.b0781	GO Process	GO:0019720	Mo-molybdopterin cofactor metabolic process
moaA	511145.b0781	GO Process	GO:0043170	Macromolecule metabolic process
moaA	511145.b0781	GO Process	GO:0043545	Molybdopterin cofactor metabolic process
moaA	511145.b0781	GO Process	GO:0044237	Cellular metabolic process
moaA	511145.b0781	GO Process	GO:0044238	Primary metabolic process
moaA	511145.b0781	GO Process	GO:0044249	Cellular biosynthetic process
moaA	511145.b0781	GO Process	GO:0046483	Heterocycle metabolic process
moaA	511145.b0781	GO Process	GO:0050896	Response to stimulus
moaA	511145.b0781	GO Process	GO:0051189	Prosthetic group metabolic process
moaA	511145.b0781	GO Process	GO:0071704	Organic substance metabolic process
moaA	511145.b0781	GO Process	GO:0090407	Organophosphate biosynthetic process
moaA	511145.b0781	GO Process	GO:1901360	Organic cyclic compound metabolic process
moaA	511145.b0781	GO Process	GO:1901362	Organic cyclic compound biosynthetic process
moaA	511145.b0781	GO Process	GO:1901564	Organonitrogen compound metabolic process
moaA	511145.b0781	GO Process	GO:1901566	Organonitrogen compound biosynthetic process
moaA	511145.b0781	GO Process	GO:1901576	Organic substance biosynthetic process
moaA	511145.b0781	GO Function	GO:0000166	Nucleotide binding
moaA	511145.b0781	GO Function	GO:0003824	Catalytic activity
moaA	511145.b0781	GO Function	GO:0005488	Binding
moaA	511145.b0781	GO Function	GO:0005525	GTP binding
moaA	511145.b0781	GO Function	GO:0016829	Lyase activity
moaA	511145.b0781	GO Function	GO:0016830	Carbon-carbon lyase activity
moaA	511145.b0781	GO Function	GO:0016849	Phosphorus-oxygen lyase activity
moaA	511145.b0781	GO Function	GO:0017076	Purine nucleotide binding
moaA	511145.b0781	GO Function	GO:0019001	Guanyl nucleotide binding
moaA	511145.b0781	GO Function	GO:0032553	Ribonucleotide binding
moaA	511145.b0781	GO Function	GO:0032555	Purine ribonucleotide binding
moaA	511145.b0781	GO Function	GO:0032561	Guanyl ribonucleotide binding
moaA	511145.b0781	GO Function	GO:0035639	Purine ribonucleoside triphosphate binding
moaA	511145.b0781	GO Function	GO:0036094	Small molecule binding
moaA	511145.b0781	GO Function	GO:0043167	Ion binding
moaA	511145.b0781	GO Function	GO:0043168	Anion binding
moaA	511145.b0781	GO Function	GO:0043169	Cation binding
moaA	511145.b0781	GO Function	GO:0046872	Metal ion binding
moaA	511145.b0781	GO Function	GO:0051536	Iron-sulfur cluster binding
moaA	511145.b0781	GO Function	GO:0051539	4 iron, 4 sulfur cluster binding
moaA	511145.b0781	GO Function	GO:0051540	Metal cluster binding
moaA	511145.b0781	GO Function	GO:0061798	GTP 3,8-cyclase activity
moaA	511145.b0781	GO Function	GO:0061799	Cyclic pyranopterin monophosphate synthase activity
moaA	511145.b0781	GO Function	GO:0097159	Organic cyclic compound binding
moaA	511145.b0781	GO Function	GO:0097367	Carbohydrate derivative binding
moaA	511145.b0781	GO Function	GO:1901265	Nucleoside phosphate binding
moaA	511145.b0781	GO Function	GO:1901363	Heterocyclic compound binding
moaA	511145.b0781	GO Function	GO:1901681	Sulfur compound binding
moaA	511145.b0781	GO Function	GO:1904047	S-adenosyl-L-methionine binding
moaA	511145.b0781	GO Component	GO:0019008	Molybdopterin synthase complex
moaA	511145.b0781	GO Component	GO:0032991	Protein-containing complex
moaA	511145.b0781	STRING clusters	CL:4810	Mixed, incl. Anaerobic respiration, and Molybdenum cofactor biosynthesis
moaA	511145.b0781	STRING clusters	CL:4811	Mixed, incl. Anaerobic respiration, and Cytochrome complex assembly
moaA	511145.b0781	STRING clusters	CL:4812	Mixed, incl. Anaerobic respiration, and Cytochrome complex assembly
moaA	511145.b0781	STRING clusters	CL:4884	Mixed, incl. Molybdenum cofactor biosynthesis, and Dimethyl sulfoxide reductase activity
moaA	511145.b0781	STRING clusters	CL:4917	Molybdenum cofactor biosynthesis
moaA	511145.b0781	STRING clusters	CL:4919	Molybdenum cofactor biosynthesis
moaA	511145.b0781	KEGG	eco00790	Folate biosynthesis
moaA	511145.b0781	KEGG	eco01100	Metabolic pathways
moaA	511145.b0781	KEGG	eco04122	Sulfur relay system
moaA	511145.b0781	COMPARTMENTS	GOCC:0019008	Molybdopterin synthase complex
moaA	511145.b0781	COMPARTMENTS	GOCC:0032991	Protein-containing complex
moaA	511145.b0781	UniProt Keywords	KW-0004	4Fe-4S
moaA	511145.b0781	UniProt Keywords	KW-0342	GTP-binding
moaA	511145.b0781	UniProt Keywords	KW-0408	Iron
moaA	511145.b0781	UniProt Keywords	KW-0411	Iron-sulfur
moaA	511145.b0781	UniProt Keywords	KW-0456	Lyase
moaA	511145.b0781	UniProt Keywords	KW-0479	Metal-binding
moaA	511145.b0781	UniProt Keywords	KW-0501	Molybdenum cofactor biosynthesis
moaA	511145.b0781	UniProt Keywords	KW-0547	Nucleotide-binding
moaA	511145.b0781	UniProt Keywords	KW-0949	S-adenosyl-L-methionine
moaA	511145.b0781	InterPro	IPR000385	MoaA/NifB/PqqE, iron-sulphur binding, conserved site
moaA	511145.b0781	InterPro	IPR006638	Elp3/MiaA/NifB-like, radical SAM core domain
moaA	511145.b0781	InterPro	IPR007197	Radical SAM
moaA	511145.b0781	InterPro	IPR010505	Molybdenum cofactor biosynthesis protein A-like, twitch domain
moaA	511145.b0781	InterPro	IPR013483	Molybdenum cofactor biosynthesis protein A
moaA	511145.b0781	InterPro	IPR013785	Aldolase-type TIM barrel
moaA	511145.b0781	SMART	SM00729	Elongator protein 3, MiaB family, Radical SAM
mqo	511145.b2210	GO Process	GO:0006091	Generation of precursor metabolites and energy
mqo	511145.b2210	GO Process	GO:0006099	Tricarboxylic acid cycle
mqo	511145.b2210	GO Process	GO:0008152	Metabolic process
mqo	511145.b2210	GO Process	GO:0009060	Aerobic respiration
mqo	511145.b2210	GO Process	GO:0009987	Cellular process
mqo	511145.b2210	GO Process	GO:0015980	Energy derivation by oxidation of organic compounds
mqo	511145.b2210	GO Process	GO:0044237	Cellular metabolic process
mqo	511145.b2210	GO Process	GO:0044238	Primary metabolic process
mqo	511145.b2210	GO Process	GO:0045333	Cellular respiration
mqo	511145.b2210	GO Function	GO:0000166	Nucleotide binding
mqo	511145.b2210	GO Function	GO:0003824	Catalytic activity
mqo	511145.b2210	GO Function	GO:0005488	Binding
mqo	511145.b2210	GO Function	GO:0008924	Malate dehydrogenase (quinone) activity
mqo	511145.b2210	GO Function	GO:0016491	Oxidoreductase activity
mqo	511145.b2210	GO Function	GO:0016614	Oxidoreductase activity, acting on CH-OH group of donors
mqo	511145.b2210	GO Function	GO:0016615	Malate dehydrogenase activity
mqo	511145.b2210	GO Function	GO:0016901	Oxidoreductase activity, acting on the CH-OH group of donors, quinone or similar compound as acceptor
mqo	511145.b2210	GO Function	GO:0036094	Small molecule binding
mqo	511145.b2210	GO Function	GO:0043167	Ion binding
mqo	511145.b2210	GO Function	GO:0043168	Anion binding
mqo	511145.b2210	GO Function	GO:0050660	Flavin adenine dinucleotide binding
mqo	511145.b2210	GO Function	GO:0052589	Malate dehydrogenase (menaquinone) activity
mqo	511145.b2210	GO Function	GO:0097159	Organic cyclic compound binding
mqo	511145.b2210	GO Function	GO:1901265	Nucleoside phosphate binding
mqo	511145.b2210	GO Function	GO:1901363	Heterocyclic compound binding
mqo	511145.b2210	GO Component	GO:0005622	Intracellular anatomical structure
mqo	511145.b2210	GO Component	GO:0005737	Cytoplasm
mqo	511145.b2210	GO Component	GO:0005829	Cytosol
mqo	511145.b2210	GO Component	GO:0005886	Plasma membrane
mqo	511145.b2210	GO Component	GO:0009898	Cytoplasmic side of plasma membrane
mqo	511145.b2210	GO Component	GO:0016020	Membrane
mqo	511145.b2210	GO Component	GO:0071944	Cell periphery
mqo	511145.b2210	GO Component	GO:0098552	Side of membrane
mqo	511145.b2210	GO Component	GO:0098562	Cytoplasmic side of membrane
mqo	511145.b2210	GO Component	GO:0110165	Cellular anatomical entity
mqo	511145.b2210	STRING clusters	CL:2010	Mixed, incl. Carbon metabolism, and Fatty acid metabolic process
mqo	511145.b2210	STRING clusters	CL:2011	Carbon metabolism, and Oxidative phosphorylation
mqo	511145.b2210	STRING clusters	CL:2012	Carbon metabolism, and Pyruvate metabolism
mqo	511145.b2210	STRING clusters	CL:2013	Carbon metabolism, and Formate C-acetyltransferase activity
mqo	511145.b2210	STRING clusters	CL:2014	Citrate cycle (TCA cycle), and Pyruvate metabolism
mqo	511145.b2210	STRING clusters	CL:2062	Citrate cycle (TCA cycle)
mqo	511145.b2210	STRING clusters	CL:2097	Malate dehydrogenase activity, and Fumarate hydratase activity
mqo	511145.b2210	KEGG	eco00020	Citrate cycle (TCA cycle)
mqo	511145.b2210	KEGG	eco00620	Pyruvate metabolism
mqo	511145.b2210	KEGG	eco01100	Metabolic pathways
mqo	511145.b2210	KEGG	eco01110	Biosynthesis of secondary metabolites
mqo	511145.b2210	KEGG	eco01120	Microbial metabolism in diverse environments
mqo	511145.b2210	KEGG	eco01200	Carbon metabolism
mqo	511145.b2210	COMPARTMENTS	GOCC:0005622	Intracellular
mqo	511145.b2210	COMPARTMENTS	GOCC:0005737	Cytoplasm
mqo	511145.b2210	COMPARTMENTS	GOCC:0005829	Cytosol
mqo	511145.b2210	COMPARTMENTS	GOCC:0005886	Plasma membrane
mqo	511145.b2210	COMPARTMENTS	GOCC:0009898	Cytoplasmic side of plasma membrane
mqo	511145.b2210	COMPARTMENTS	GOCC:0016020	Membrane
mqo	511145.b2210	COMPARTMENTS	GOCC:0071944	Cell periphery
mqo	511145.b2210	COMPARTMENTS	GOCC:0098552	Side of membrane
mqo	511145.b2210	COMPARTMENTS	GOCC:0098562	Cytoplasmic side of membrane
mqo	511145.b2210	COMPARTMENTS	GOCC:0110165	Cellular anatomical entity
mqo	511145.b2210	UniProt Keywords	KW-0274	FAD
mqo	511145.b2210	UniProt Keywords	KW-0285	Flavoprotein
mqo	511145.b2210	UniProt Keywords	KW-0560	Oxidoreductase
mqo	511145.b2210	UniProt Keywords	KW-0816	Tricarboxylic acid cycle
mqo	511145.b2210	Pfam	PF01266	FAD dependent oxidoreductase
mqo	511145.b2210	InterPro	IPR006231	Malate:quinone-oxidoreductase
mqo	511145.b2210	InterPro	IPR036188	FAD/NAD(P)-binding domain superfamily
msrA	511145.b4219	GO Process	GO:0006807	Nitrogen compound metabolic process
msrA	511145.b4219	GO Process	GO:0006950	Response to stress
msrA	511145.b4219	GO Process	GO:0006979	Response to oxidative stress
msrA	511145.b4219	GO Process	GO:0008152	Metabolic process
msrA	511145.b4219	GO Process	GO:0009987	Cellular process
msrA	511145.b4219	GO Process	GO:0019538	Protein metabolic process
msrA	511145.b4219	GO Process	GO:0030091	Protein repair
msrA	511145.b4219	GO Process	GO:0033554	Cellular response to stress
msrA	511145.b4219	GO Process	GO:0034599	Cellular response to oxidative stress
msrA	511145.b4219	GO Process	GO:0042221	Response to chemical
msrA	511145.b4219	GO Process	GO:0043170	Macromolecule metabolic process
msrA	511145.b4219	GO Process	GO:0044238	Primary metabolic process
msrA	511145.b4219	GO Process	GO:0050896	Response to stimulus
msrA	511145.b4219	GO Process	GO:0051716	Cellular response to stimulus
msrA	511145.b4219	GO Process	GO:0062197	Cellular response to chemical stress
msrA	511145.b4219	GO Process	GO:0070887	Cellular response to chemical stimulus
msrA	511145.b4219	GO Process	GO:0071704	Organic substance metabolic process
msrA	511145.b4219	GO Process	GO:1901564	Organonitrogen compound metabolic process
msrA	511145.b4219	GO Function	GO:0003824	Catalytic activity
msrA	511145.b4219	GO Function	GO:0008113	Peptide-methionine (S)-S-oxide reductase activity
msrA	511145.b4219	GO Function	GO:0016491	Oxidoreductase activity
msrA	511145.b4219	GO Function	GO:0016667	Oxidoreductase activity, acting on a sulfur group of donors
msrA	511145.b4219	GO Function	GO:0016671	Oxidoreductase activity, acting on a sulfur group of donors, disulfide as acceptor
msrA	511145.b4219	GO Function	GO:0033744	L-methionine:thioredoxin-disulfide S-oxidoreductase activity
msrA	511145.b4219	GO Function	GO:0036456	L-methionine-(S)-S-oxide reductase activity
msrA	511145.b4219	GO Component	GO:0005622	Intracellular anatomical structure
msrA	511145.b4219	GO Component	GO:0005737	Cytoplasm
msrA	511145.b4219	GO Component	GO:0005829	Cytosol
msrA	511145.b4219	GO Component	GO:0110165	Cellular anatomical entity
msrA	511145.b4219	STRING clusters	CL:3861	Mostly uncharacterized, incl. Protein repair, and Peptidase M48
msrA	511145.b4219	STRING clusters	CL:3862	Mixed, incl. Protein repair, and Peptidase M48
msrA	511145.b4219	STRING clusters	CL:3863	Mixed, incl. Protein repair, and TAM protein secretion complex
msrA	511145.b4219	COMPARTMENTS	GOCC:0005622	Intracellular
msrA	511145.b4219	COMPARTMENTS	GOCC:0005737	Cytoplasm
msrA	511145.b4219	COMPARTMENTS	GOCC:0005829	Cytosol
msrA	511145.b4219	COMPARTMENTS	GOCC:0110165	Cellular anatomical entity
msrA	511145.b4219	UniProt Keywords	KW-0560	Oxidoreductase
msrA	511145.b4219	UniProt Keywords	KW-0676	Redox-active center
msrA	511145.b4219	UniProt Keywords	KW-1015	Disulfide bond
msrA	511145.b4219	Pfam	PF01625	Peptide methionine sulfoxide reductase
msrA	511145.b4219	InterPro	IPR002569	Peptide methionine sulphoxide reductase MsrA domain
msrA	511145.b4219	InterPro	IPR036509	Peptide methionine sulphoxide reductase MsrA superfamily
mtn	511145.b0159	GO Process	GO:0000096	Sulfur amino acid metabolic process
mtn	511145.b0159	GO Process	GO:0000097	Sulfur amino acid biosynthetic process
mtn	511145.b0159	GO Process	GO:0006082	Organic acid metabolic process
mtn	511145.b0159	GO Process	GO:0006139	Nucleobase-containing compound metabolic process
mtn	511145.b0159	GO Process	GO:0006152	Purine nucleoside catabolic process
mtn	511145.b0159	GO Process	GO:0006520	Cellular amino acid metabolic process
mtn	511145.b0159	GO Process	GO:0006555	Methionine metabolic process
mtn	511145.b0159	GO Process	GO:0006725	Cellular aromatic compound metabolic process
mtn	511145.b0159	GO Process	GO:0006790	Sulfur compound metabolic process
mtn	511145.b0159	GO Process	GO:0006807	Nitrogen compound metabolic process
mtn	511145.b0159	GO Process	GO:0008152	Metabolic process
mtn	511145.b0159	GO Process	GO:0008652	Cellular amino acid biosynthetic process
mtn	511145.b0159	GO Process	GO:0009056	Catabolic process
mtn	511145.b0159	GO Process	GO:0009058	Biosynthetic process
mtn	511145.b0159	GO Process	GO:0009066	Aspartate family amino acid metabolic process
mtn	511145.b0159	GO Process	GO:0009067	Aspartate family amino acid biosynthetic process
mtn	511145.b0159	GO Process	GO:0009086	Methionine biosynthetic process
mtn	511145.b0159	GO Process	GO:0009116	Nucleoside metabolic process
mtn	511145.b0159	GO Process	GO:0009120	Deoxyribonucleoside metabolic process
mtn	511145.b0159	GO Process	GO:0009164	Nucleoside catabolic process
mtn	511145.b0159	GO Process	GO:0009636	Response to toxic substance
mtn	511145.b0159	GO Process	GO:0009987	Cellular process
mtn	511145.b0159	GO Process	GO:0016053	Organic acid biosynthetic process
mtn	511145.b0159	GO Process	GO:0019284	L-methionine salvage from S-adenosylmethionine
mtn	511145.b0159	GO Process	GO:0019439	Aromatic compound catabolic process
mtn	511145.b0159	GO Process	GO:0019509	L-methionine salvage from methylthioadenosine
mtn	511145.b0159	GO Process	GO:0019752	Carboxylic acid metabolic process
mtn	511145.b0159	GO Process	GO:0033353	S-adenosylmethionine cycle
mtn	511145.b0159	GO Process	GO:0034641	Cellular nitrogen compound metabolic process
mtn	511145.b0159	GO Process	GO:0034655	Nucleobase-containing compound catabolic process
mtn	511145.b0159	GO Process	GO:0034656	Nucleobase-containing small molecule catabolic process
mtn	511145.b0159	GO Process	GO:0042221	Response to chemical
mtn	511145.b0159	GO Process	GO:0042278	Purine nucleoside metabolic process
mtn	511145.b0159	GO Process	GO:0043094	Cellular metabolic compound salvage
mtn	511145.b0159	GO Process	GO:0043102	Amino acid salvage
mtn	511145.b0159	GO Process	GO:0043436	Oxoacid metabolic process
mtn	511145.b0159	GO Process	GO:0044237	Cellular metabolic process
mtn	511145.b0159	GO Process	GO:0044238	Primary metabolic process
mtn	511145.b0159	GO Process	GO:0044248	Cellular catabolic process
mtn	511145.b0159	GO Process	GO:0044249	Cellular biosynthetic process
mtn	511145.b0159	GO Process	GO:0044270	Cellular nitrogen compound catabolic process
mtn	511145.b0159	GO Process	GO:0044272	Sulfur compound biosynthetic process
mtn	511145.b0159	GO Process	GO:0044281	Small molecule metabolic process
mtn	511145.b0159	GO Process	GO:0044282	Small molecule catabolic process
mtn	511145.b0159	GO Process	GO:0044283	Small molecule biosynthetic process
mtn	511145.b0159	GO Process	GO:0046121	Deoxyribonucleoside catabolic process
mtn	511145.b0159	GO Process	GO:0046122	Purine deoxyribonucleoside metabolic process
mtn	511145.b0159	GO Process	GO:0046124	Purine deoxyribonucleoside catabolic process
mtn	511145.b0159	GO Process	GO:0046394	Carboxylic acid biosynthetic process
mtn	511145.b0159	GO Process	GO:0046483	Heterocycle metabolic process
mtn	511145.b0159	GO Process	GO:0046500	S-adenosylmethionine metabolic process
mtn	511145.b0159	GO Process	GO:0046700	Heterocycle catabolic process
mtn	511145.b0159	GO Process	GO:0050896	Response to stimulus
mtn	511145.b0159	GO Process	GO:0051716	Cellular response to stimulus
mtn	511145.b0159	GO Process	GO:0055086	Nucleobase-containing small molecule metabolic process
mtn	511145.b0159	GO Process	GO:0070887	Cellular response to chemical stimulus
mtn	511145.b0159	GO Process	GO:0071265	L-methionine biosynthetic process
mtn	511145.b0159	GO Process	GO:0071267	L-methionine salvage
mtn	511145.b0159	GO Process	GO:0071704	Organic substance metabolic process
mtn	511145.b0159	GO Process	GO:0072521	Purine-containing compound metabolic process
mtn	511145.b0159	GO Process	GO:0072523	Purine-containing compound catabolic process
mtn	511145.b0159	GO Process	GO:0097237	Cellular response to toxic substance
mtn	511145.b0159	GO Process	GO:0098754	Detoxification
mtn	511145.b0159	GO Process	GO:0110051	Metabolite repair
mtn	511145.b0159	GO Process	GO:0110052	Toxic metabolite repair
mtn	511145.b0159	GO Process	GO:1901135	Carbohydrate derivative metabolic process
mtn	511145.b0159	GO Process	GO:1901136	Carbohydrate derivative catabolic process
mtn	511145.b0159	GO Process	GO:1901360	Organic cyclic compound metabolic process
mtn	511145.b0159	GO Process	GO:1901361	Organic cyclic compound catabolic process
mtn	511145.b0159	GO Process	GO:1901564	Organonitrogen compound metabolic process
mtn	511145.b0159	GO Process	GO:1901565	Organonitrogen compound catabolic process
mtn	511145.b0159	GO Process	GO:1901566	Organonitrogen compound biosynthetic process
mtn	511145.b0159	GO Process	GO:1901575	Organic substance catabolic process
mtn	511145.b0159	GO Process	GO:1901576	Organic substance biosynthetic process
mtn	511145.b0159	GO Process	GO:1901605	Alpha-amino acid metabolic process
mtn	511145.b0159	GO Process	GO:1901607	Alpha-amino acid biosynthetic process
mtn	511145.b0159	GO Process	GO:1901657	Glycosyl compound metabolic process
mtn	511145.b0159	GO Process	GO:1901658	Glycosyl compound catabolic process
mtn	511145.b0159	GO Process	GO:1990748	Cellular detoxification
mtn	511145.b0159	GO Function	GO:0003824	Catalytic activity
mtn	511145.b0159	GO Function	GO:0005488	Binding
mtn	511145.b0159	GO Function	GO:0005515	Protein binding
mtn	511145.b0159	GO Function	GO:0008477	Purine nucleosidase activity
mtn	511145.b0159	GO Function	GO:0008782	Adenosylhomocysteine nucleosidase activity
mtn	511145.b0159	GO Function	GO:0008930	Methylthioadenosine nucleosidase activity
mtn	511145.b0159	GO Function	GO:0016787	Hydrolase activity
mtn	511145.b0159	GO Function	GO:0016798	Hydrolase activity, acting on glycosyl bonds
mtn	511145.b0159	GO Function	GO:0016799	Hydrolase activity, hydrolyzing N-glycosyl compounds
mtn	511145.b0159	GO Function	GO:0042802	Identical protein binding
mtn	511145.b0159	GO Component	GO:0005622	Intracellular anatomical structure
mtn	511145.b0159	GO Component	GO:0005737	Cytoplasm
mtn	511145.b0159	GO Component	GO:0005829	Cytosol
mtn	511145.b0159	GO Component	GO:0110165	Cellular anatomical entity
mtn	511145.b0159	STRING clusters	CL:4178	ATP-binding cassette (ABC) transporter complex
mtn	511145.b0159	STRING clusters	CL:4179	ATP-binding cassette (ABC) transporter complex
mtn	511145.b0159	STRING clusters	CL:4493	Mixed, incl. Quorum sensing, and Metabolite repair
mtn	511145.b0159	KEGG	eco00270	Cysteine and methionine metabolism
mtn	511145.b0159	KEGG	eco01100	Metabolic pathways
mtn	511145.b0159	KEGG	eco01230	Biosynthesis of amino acids
mtn	511145.b0159	COMPARTMENTS	GOCC:0005622	Intracellular
mtn	511145.b0159	COMPARTMENTS	GOCC:0005737	Cytoplasm
mtn	511145.b0159	COMPARTMENTS	GOCC:0005829	Cytosol
mtn	511145.b0159	COMPARTMENTS	GOCC:0110165	Cellular anatomical entity
mtn	511145.b0159	UniProt Keywords	KW-0028	Amino-acid biosynthesis
mtn	511145.b0159	UniProt Keywords	KW-0378	Hydrolase
mtn	511145.b0159	UniProt Keywords	KW-0486	Methionine biosynthesis
mtn	511145.b0159	Pfam	PF01048	Phosphorylase superfamily
mtn	511145.b0159	InterPro	IPR000845	Nucleoside phosphorylase domain
mtn	511145.b0159	InterPro	IPR010049	MTA/SAH nucleosidase
mtn	511145.b0159	InterPro	IPR035994	Nucleoside phosphorylase superfamily
murB	511145.b3972	GO Process	GO:0000270	Peptidoglycan metabolic process
murB	511145.b3972	GO Process	GO:0006022	Aminoglycan metabolic process
murB	511145.b3972	GO Process	GO:0006023	Aminoglycan biosynthetic process
murB	511145.b3972	GO Process	GO:0006024	Glycosaminoglycan biosynthetic process
murB	511145.b3972	GO Process	GO:0006807	Nitrogen compound metabolic process
murB	511145.b3972	GO Process	GO:0007049	Cell cycle
murB	511145.b3972	GO Process	GO:0008152	Metabolic process
murB	511145.b3972	GO Process	GO:0008360	Regulation of cell shape
murB	511145.b3972	GO Process	GO:0009058	Biosynthetic process
murB	511145.b3972	GO Process	GO:0009059	Macromolecule biosynthetic process
murB	511145.b3972	GO Process	GO:0009252	Peptidoglycan biosynthetic process
murB	511145.b3972	GO Process	GO:0009273	Peptidoglycan-based cell wall biogenesis
murB	511145.b3972	GO Process	GO:0009987	Cellular process
murB	511145.b3972	GO Process	GO:0016043	Cellular component organization
murB	511145.b3972	GO Process	GO:0022603	Regulation of anatomical structure morphogenesis
murB	511145.b3972	GO Process	GO:0022604	Regulation of cell morphogenesis
murB	511145.b3972	GO Process	GO:0030203	Glycosaminoglycan metabolic process
murB	511145.b3972	GO Process	GO:0034645	Cellular macromolecule biosynthetic process
murB	511145.b3972	GO Process	GO:0042546	Cell wall biogenesis
murB	511145.b3972	GO Process	GO:0043170	Macromolecule metabolic process
murB	511145.b3972	GO Process	GO:0044036	Cell wall macromolecule metabolic process
murB	511145.b3972	GO Process	GO:0044038	Cell wall macromolecule biosynthetic process
murB	511145.b3972	GO Process	GO:0044085	Cellular component biogenesis
murB	511145.b3972	GO Process	GO:0044237	Cellular metabolic process
murB	511145.b3972	GO Process	GO:0044249	Cellular biosynthetic process
murB	511145.b3972	GO Process	GO:0044260	Cellular macromolecule metabolic process
murB	511145.b3972	GO Process	GO:0045229	External encapsulating structure organization
murB	511145.b3972	GO Process	GO:0050789	Regulation of biological process
murB	511145.b3972	GO Process	GO:0050793	Regulation of developmental process
murB	511145.b3972	GO Process	GO:0051301	Cell division
murB	511145.b3972	GO Process	GO:0065007	Biological regulation
murB	511145.b3972	GO Process	GO:0070589	Cellular component macromolecule biosynthetic process
murB	511145.b3972	GO Process	GO:0071554	Cell wall organization or biogenesis
murB	511145.b3972	GO Process	GO:0071555	Cell wall organization
murB	511145.b3972	GO Process	GO:0071704	Organic substance metabolic process
murB	511145.b3972	GO Process	GO:0071840	Cellular component organization or biogenesis
murB	511145.b3972	GO Process	GO:1901135	Carbohydrate derivative metabolic process
murB	511145.b3972	GO Process	GO:1901137	Carbohydrate derivative biosynthetic process
murB	511145.b3972	GO Process	GO:1901564	Organonitrogen compound metabolic process
murB	511145.b3972	GO Process	GO:1901566	Organonitrogen compound biosynthetic process
murB	511145.b3972	GO Process	GO:1901576	Organic substance biosynthetic process
murB	511145.b3972	GO Function	GO:0000166	Nucleotide binding
murB	511145.b3972	GO Function	GO:0003824	Catalytic activity
murB	511145.b3972	GO Function	GO:0005488	Binding
murB	511145.b3972	GO Function	GO:0008762	UDP-N-acetylmuramate dehydrogenase activity
murB	511145.b3972	GO Function	GO:0016491	Oxidoreductase activity
murB	511145.b3972	GO Function	GO:0016614	Oxidoreductase activity, acting on CH-OH group of donors
murB	511145.b3972	GO Function	GO:0016616	Oxidoreductase activity, acting on the CH-OH group of donors, NAD or NADP as acceptor
murB	511145.b3972	GO Function	GO:0036094	Small molecule binding
murB	511145.b3972	GO Function	GO:0043167	Ion binding
murB	511145.b3972	GO Function	GO:0043168	Anion binding
murB	511145.b3972	GO Function	GO:0050660	Flavin adenine dinucleotide binding
murB	511145.b3972	GO Function	GO:0071949	FAD binding
murB	511145.b3972	GO Function	GO:0097159	Organic cyclic compound binding
murB	511145.b3972	GO Function	GO:1901265	Nucleoside phosphate binding
murB	511145.b3972	GO Function	GO:1901363	Heterocyclic compound binding
murB	511145.b3972	GO Component	GO:0005622	Intracellular anatomical structure
murB	511145.b3972	GO Component	GO:0005737	Cytoplasm
murB	511145.b3972	GO Component	GO:0005829	Cytosol
murB	511145.b3972	GO Component	GO:0110165	Cellular anatomical entity
murB	511145.b3972	STRING clusters	CL:897	Mixed, incl. Peptidoglycan metabolic process, and Cell division site
murB	511145.b3972	STRING clusters	CL:898	Mixed, incl. Peptidoglycan metabolic process, and Cell division site
murB	511145.b3972	STRING clusters	CL:899	Peptidoglycan metabolic process, and Cell septum
murB	511145.b3972	STRING clusters	CL:901	Peptidoglycan metabolic process, and Cell septum
murB	511145.b3972	STRING clusters	CL:902	Peptidoglycan metabolic process, and Cell septum
murB	511145.b3972	STRING clusters	CL:903	Cell shape, and Divisome complex
murB	511145.b3972	STRING clusters	CL:904	Cell shape, and Divisome complex
murB	511145.b3972	STRING clusters	CL:946	Vancomycin resistance, and Mur ligase, central
murB	511145.b3972	KEGG	eco00520	Amino sugar and nucleotide sugar metabolism
murB	511145.b3972	KEGG	eco00550	Peptidoglycan biosynthesis
murB	511145.b3972	KEGG	eco01100	Metabolic pathways
murB	511145.b3972	COMPARTMENTS	GOCC:0005622	Intracellular
murB	511145.b3972	COMPARTMENTS	GOCC:0005737	Cytoplasm
murB	511145.b3972	COMPARTMENTS	GOCC:0005829	Cytosol
murB	511145.b3972	COMPARTMENTS	GOCC:0110165	Cellular anatomical entity
murB	511145.b3972	UniProt Keywords	KW-0131	Cell cycle
murB	511145.b3972	UniProt Keywords	KW-0132	Cell division
murB	511145.b3972	UniProt Keywords	KW-0133	Cell shape
murB	511145.b3972	UniProt Keywords	KW-0274	FAD
murB	511145.b3972	UniProt Keywords	KW-0285	Flavoprotein
murB	511145.b3972	UniProt Keywords	KW-0521	NADP
murB	511145.b3972	UniProt Keywords	KW-0560	Oxidoreductase
murB	511145.b3972	UniProt Keywords	KW-0573	Peptidoglycan synthesis
murB	511145.b3972	UniProt Keywords	KW-0961	Cell wall biogenesis/degradation
murB	511145.b3972	UniProt Keywords	KW-0963	Cytoplasm
murB	511145.b3972	Pfam	PF01565	FAD binding domain
murB	511145.b3972	Pfam	PF02873	UDP-N-acetylenolpyruvoylglucosamine reductase, C-terminal domain
murB	511145.b3972	InterPro	IPR003170	UDP-N-acetylenolpyruvoylglucosamine reductase
murB	511145.b3972	InterPro	IPR006094	FAD linked oxidase, N-terminal
murB	511145.b3972	InterPro	IPR011601	UDP-N-acetylenolpyruvoylglucosamine reductase, C-terminal
murB	511145.b3972	InterPro	IPR016166	FAD-binding domain, PCMH-type
murB	511145.b3972	InterPro	IPR016167	FAD-binding, type PCMH, subdomain 1
murB	511145.b3972	InterPro	IPR016169	FAD-binding, type PCMH, subdomain 2
murB	511145.b3972	InterPro	IPR036318	FAD-binding, type PCMH-like superfamily
murB	511145.b3972	InterPro	IPR036635	UDP-N-acetylenolpyruvoylglucosamine reductase, C-terminal domain superfamily
murI	511145.b3967	GO Process	GO:0000270	Peptidoglycan metabolic process
murI	511145.b3967	GO Process	GO:0006022	Aminoglycan metabolic process
murI	511145.b3967	GO Process	GO:0006023	Aminoglycan biosynthetic process
murI	511145.b3967	GO Process	GO:0006024	Glycosaminoglycan biosynthetic process
murI	511145.b3967	GO Process	GO:0006807	Nitrogen compound metabolic process
murI	511145.b3967	GO Process	GO:0008152	Metabolic process
murI	511145.b3967	GO Process	GO:0008360	Regulation of cell shape
murI	511145.b3967	GO Process	GO:0009058	Biosynthetic process
murI	511145.b3967	GO Process	GO:0009059	Macromolecule biosynthetic process
murI	511145.b3967	GO Process	GO:0009252	Peptidoglycan biosynthetic process
murI	511145.b3967	GO Process	GO:0009273	Peptidoglycan-based cell wall biogenesis
murI	511145.b3967	GO Process	GO:0009987	Cellular process
murI	511145.b3967	GO Process	GO:0016043	Cellular component organization
murI	511145.b3967	GO Process	GO:0022603	Regulation of anatomical structure morphogenesis
murI	511145.b3967	GO Process	GO:0022604	Regulation of cell morphogenesis
murI	511145.b3967	GO Process	GO:0030203	Glycosaminoglycan metabolic process
murI	511145.b3967	GO Process	GO:0034645	Cellular macromolecule biosynthetic process
murI	511145.b3967	GO Process	GO:0042546	Cell wall biogenesis
murI	511145.b3967	GO Process	GO:0043170	Macromolecule metabolic process
murI	511145.b3967	GO Process	GO:0044036	Cell wall macromolecule metabolic process
murI	511145.b3967	GO Process	GO:0044038	Cell wall macromolecule biosynthetic process
murI	511145.b3967	GO Process	GO:0044085	Cellular component biogenesis
murI	511145.b3967	GO Process	GO:0044237	Cellular metabolic process
murI	511145.b3967	GO Process	GO:0044249	Cellular biosynthetic process
murI	511145.b3967	GO Process	GO:0044260	Cellular macromolecule metabolic process
murI	511145.b3967	GO Process	GO:0045229	External encapsulating structure organization
murI	511145.b3967	GO Process	GO:0050789	Regulation of biological process
murI	511145.b3967	GO Process	GO:0050793	Regulation of developmental process
murI	511145.b3967	GO Process	GO:0065007	Biological regulation
murI	511145.b3967	GO Process	GO:0070589	Cellular component macromolecule biosynthetic process
murI	511145.b3967	GO Process	GO:0071554	Cell wall organization or biogenesis
murI	511145.b3967	GO Process	GO:0071555	Cell wall organization
murI	511145.b3967	GO Process	GO:0071704	Organic substance metabolic process
murI	511145.b3967	GO Process	GO:0071840	Cellular component organization or biogenesis
murI	511145.b3967	GO Process	GO:1901135	Carbohydrate derivative metabolic process
murI	511145.b3967	GO Process	GO:1901137	Carbohydrate derivative biosynthetic process
murI	511145.b3967	GO Process	GO:1901564	Organonitrogen compound metabolic process
murI	511145.b3967	GO Process	GO:1901566	Organonitrogen compound biosynthetic process
murI	511145.b3967	GO Process	GO:1901576	Organic substance biosynthetic process
murI	511145.b3967	GO Function	GO:0003824	Catalytic activity
murI	511145.b3967	GO Function	GO:0008881	Glutamate racemase activity
murI	511145.b3967	GO Function	GO:0016853	Isomerase activity
murI	511145.b3967	GO Function	GO:0016854	Racemase and epimerase activity
murI	511145.b3967	GO Function	GO:0016855	Racemase and epimerase activity, acting on amino acids and derivatives
murI	511145.b3967	GO Function	GO:0036361	Racemase activity, acting on amino acids and derivatives
murI	511145.b3967	GO Function	GO:0047661	Amino-acid racemase activity
murI	511145.b3967	STRING clusters	CL:897	Mixed, incl. Peptidoglycan metabolic process, and Cell division site
murI	511145.b3967	STRING clusters	CL:898	Mixed, incl. Peptidoglycan metabolic process, and Cell division site
murI	511145.b3967	STRING clusters	CL:899	Peptidoglycan metabolic process, and Cell septum
murI	511145.b3967	STRING clusters	CL:901	Peptidoglycan metabolic process, and Cell septum
murI	511145.b3967	STRING clusters	CL:902	Peptidoglycan metabolic process, and Cell septum
murI	511145.b3967	STRING clusters	CL:903	Cell shape, and Divisome complex
murI	511145.b3967	STRING clusters	CL:904	Cell shape, and Divisome complex
murI	511145.b3967	KEGG	eco00471	D-Glutamine and D-glutamate metabolism
murI	511145.b3967	KEGG	eco01100	Metabolic pathways
murI	511145.b3967	UniProt Keywords	KW-0133	Cell shape
murI	511145.b3967	UniProt Keywords	KW-0413	Isomerase
murI	511145.b3967	UniProt Keywords	KW-0573	Peptidoglycan synthesis
murI	511145.b3967	UniProt Keywords	KW-0961	Cell wall biogenesis/degradation
murI	511145.b3967	Pfam	PF01177	Asp/Glu/Hydantoin racemase
murI	511145.b3967	InterPro	IPR001920	Asp/Glu racemase
murI	511145.b3967	InterPro	IPR004391	Glutamate racemase
murI	511145.b3967	InterPro	IPR015942	Asp/Glu/hydantoin racemase
murI	511145.b3967	InterPro	IPR018187	Asp/Glu racemase, active site 1
murI	511145.b3967	InterPro	IPR033134	Asp/Glu racemase, active site 2
mutM	511145.b3635	GO Process	GO:0006139	Nucleobase-containing compound metabolic process
mutM	511145.b3635	GO Process	GO:0006259	DNA metabolic process
mutM	511145.b3635	GO Process	GO:0006281	DNA repair
mutM	511145.b3635	GO Process	GO:0006284	Base-excision repair
mutM	511145.b3635	GO Process	GO:0006725	Cellular aromatic compound metabolic process
mutM	511145.b3635	GO Process	GO:0006807	Nitrogen compound metabolic process
mutM	511145.b3635	GO Process	GO:0006950	Response to stress
mutM	511145.b3635	GO Process	GO:0006974	Cellular response to DNA damage stimulus
mutM	511145.b3635	GO Process	GO:0008152	Metabolic process
mutM	511145.b3635	GO Process	GO:0009987	Cellular process
mutM	511145.b3635	GO Process	GO:0033554	Cellular response to stress
mutM	511145.b3635	GO Process	GO:0034641	Cellular nitrogen compound metabolic process
mutM	511145.b3635	GO Process	GO:0043170	Macromolecule metabolic process
mutM	511145.b3635	GO Process	GO:0044237	Cellular metabolic process
mutM	511145.b3635	GO Process	GO:0044238	Primary metabolic process
mutM	511145.b3635	GO Process	GO:0044260	Cellular macromolecule metabolic process
mutM	511145.b3635	GO Process	GO:0046483	Heterocycle metabolic process
mutM	511145.b3635	GO Process	GO:0050896	Response to stimulus
mutM	511145.b3635	GO Process	GO:0051716	Cellular response to stimulus
mutM	511145.b3635	GO Process	GO:0071704	Organic substance metabolic process
mutM	511145.b3635	GO Process	GO:0090304	Nucleic acid metabolic process
mutM	511145.b3635	GO Process	GO:0090305	Nucleic acid phosphodiester bond hydrolysis
mutM	511145.b3635	GO Process	GO:1901360	Organic cyclic compound metabolic process
mutM	511145.b3635	GO Function	GO:0000702	Oxidized base lesion DNA N-glycosylase activity
mutM	511145.b3635	GO Function	GO:0000703	Oxidized pyrimidine nucleobase lesion DNA N-glycosylase activity
mutM	511145.b3635	GO Function	GO:0003676	Nucleic acid binding
mutM	511145.b3635	GO Function	GO:0003677	DNA binding
mutM	511145.b3635	GO Function	GO:0003684	Damaged DNA binding
mutM	511145.b3635	GO Function	GO:0003824	Catalytic activity
mutM	511145.b3635	GO Function	GO:0003906	DNA-(apurinic or apyrimidinic site) endonuclease activity
mutM	511145.b3635	GO Function	GO:0004518	Nuclease activity
mutM	511145.b3635	GO Function	GO:0004519	Endonuclease activity
mutM	511145.b3635	GO Function	GO:0005488	Binding
mutM	511145.b3635	GO Function	GO:0008270	Zinc ion binding
mutM	511145.b3635	GO Function	GO:0008534	Oxidized purine nucleobase lesion DNA N-glycosylase activity
mutM	511145.b3635	GO Function	GO:0016787	Hydrolase activity
mutM	511145.b3635	GO Function	GO:0016788	Hydrolase activity, acting on ester bonds
mutM	511145.b3635	GO Function	GO:0016798	Hydrolase activity, acting on glycosyl bonds
mutM	511145.b3635	GO Function	GO:0016799	Hydrolase activity, hydrolyzing N-glycosyl compounds
mutM	511145.b3635	GO Function	GO:0016829	Lyase activity
mutM	511145.b3635	GO Function	GO:0016835	Carbon-oxygen lyase activity
mutM	511145.b3635	GO Function	GO:0019104	DNA N-glycosylase activity
mutM	511145.b3635	GO Function	GO:0034039	8-oxo-7,8-dihydroguanine DNA N-glycosylase activity
mutM	511145.b3635	GO Function	GO:0043167	Ion binding
mutM	511145.b3635	GO Function	GO:0043169	Cation binding
mutM	511145.b3635	GO Function	GO:0046872	Metal ion binding
mutM	511145.b3635	GO Function	GO:0046914	Transition metal ion binding
mutM	511145.b3635	GO Function	GO:0097159	Organic cyclic compound binding
mutM	511145.b3635	GO Function	GO:0140078	Class I DNA-(apurinic or apyrimidinic site) endonuclease activity
mutM	511145.b3635	GO Function	GO:0140097	Catalytic activity, acting on DNA
mutM	511145.b3635	GO Function	GO:0140640	Catalytic activity, acting on a nucleic acid
mutM	511145.b3635	GO Function	GO:1901363	Heterocyclic compound binding
mutM	511145.b3635	STRING clusters	CL:689	DNA repair, and Chromosome
mutM	511145.b3635	STRING clusters	CL:690	DNA repair, and Chromosome
mutM	511145.b3635	STRING clusters	CL:691	DNA repair, and Chromosome
mutM	511145.b3635	STRING clusters	CL:693	DNA damage, and Exodeoxyribonuclease activity
mutM	511145.b3635	STRING clusters	CL:772	Base excision repair
mutM	511145.b3635	KEGG	eco03410	Base excision repair
mutM	511145.b3635	UniProt Keywords	KW-0227	DNA damage
mutM	511145.b3635	UniProt Keywords	KW-0234	DNA repair
mutM	511145.b3635	UniProt Keywords	KW-0238	DNA-binding
mutM	511145.b3635	UniProt Keywords	KW-0326	Glycosidase
mutM	511145.b3635	UniProt Keywords	KW-0378	Hydrolase
mutM	511145.b3635	UniProt Keywords	KW-0456	Lyase
mutM	511145.b3635	UniProt Keywords	KW-0479	Metal-binding
mutM	511145.b3635	UniProt Keywords	KW-0511	Multifunctional enzyme
mutM	511145.b3635	UniProt Keywords	KW-0862	Zinc
mutM	511145.b3635	UniProt Keywords	KW-0863	Zinc-finger
mutM	511145.b3635	Pfam	PF06827	Zinc finger found in FPG and IleRS
mutM	511145.b3635	InterPro	IPR000214	Zinc finger, DNA glycosylase/AP lyase-type
mutM	511145.b3635	InterPro	IPR010663	Zinc finger, FPG/IleRS-type
mutM	511145.b3635	InterPro	IPR010979	Ribosomal protein S13-like, H2TH
mutM	511145.b3635	InterPro	IPR012319	Formamidopyrimidine-DNA glycosylase, catalytic domain
mutM	511145.b3635	InterPro	IPR015886	DNA glycosylase/AP lyase, H2TH DNA-binding
mutM	511145.b3635	InterPro	IPR015887	DNA glycosylase/AP lyase, zinc finger domain, DNA-binding site
mutM	511145.b3635	InterPro	IPR020629	Formamidopyrimidine-DNA glycosylase
mutM	511145.b3635	InterPro	IPR035937	MutM-like, N-terminal
mutM	511145.b3635	SMART	SM00898	Formamidopyrimidine-DNA glycosylase N-terminal domain
mutM	511145.b3635	SMART	SM01232	Formamidopyrimidine-DNA glycosylase H2TH domain
ndk	511145.b2518	GO Process	GO:0006139	Nucleobase-containing compound metabolic process
ndk	511145.b2518	GO Process	GO:0006163	Purine nucleotide metabolic process
ndk	511145.b2518	GO Process	GO:0006164	Purine nucleotide biosynthetic process
ndk	511145.b2518	GO Process	GO:0006165	Nucleoside diphosphate phosphorylation
ndk	511145.b2518	GO Process	GO:0006183	GTP biosynthetic process
ndk	511145.b2518	GO Process	GO:0006220	Pyrimidine nucleotide metabolic process
ndk	511145.b2518	GO Process	GO:0006221	Pyrimidine nucleotide biosynthetic process
ndk	511145.b2518	GO Process	GO:0006228	UTP biosynthetic process
ndk	511145.b2518	GO Process	GO:0006241	CTP biosynthetic process
ndk	511145.b2518	GO Process	GO:0006725	Cellular aromatic compound metabolic process
ndk	511145.b2518	GO Process	GO:0006753	Nucleoside phosphate metabolic process
ndk	511145.b2518	GO Process	GO:0006793	Phosphorus metabolic process
ndk	511145.b2518	GO Process	GO:0006796	Phosphate-containing compound metabolic process
ndk	511145.b2518	GO Process	GO:0006807	Nitrogen compound metabolic process
ndk	511145.b2518	GO Process	GO:0008152	Metabolic process
ndk	511145.b2518	GO Process	GO:0009058	Biosynthetic process
ndk	511145.b2518	GO Process	GO:0009117	Nucleotide metabolic process
ndk	511145.b2518	GO Process	GO:0009132	Nucleoside diphosphate metabolic process
ndk	511145.b2518	GO Process	GO:0009141	Nucleoside triphosphate metabolic process
ndk	511145.b2518	GO Process	GO:0009142	Nucleoside triphosphate biosynthetic process
ndk	511145.b2518	GO Process	GO:0009144	Purine nucleoside triphosphate metabolic process
ndk	511145.b2518	GO Process	GO:0009145	Purine nucleoside triphosphate biosynthetic process
ndk	511145.b2518	GO Process	GO:0009147	Pyrimidine nucleoside triphosphate metabolic process
ndk	511145.b2518	GO Process	GO:0009148	Pyrimidine nucleoside triphosphate biosynthetic process
ndk	511145.b2518	GO Process	GO:0009150	Purine ribonucleotide metabolic process
ndk	511145.b2518	GO Process	GO:0009152	Purine ribonucleotide biosynthetic process
ndk	511145.b2518	GO Process	GO:0009165	Nucleotide biosynthetic process
ndk	511145.b2518	GO Process	GO:0009199	Ribonucleoside triphosphate metabolic process
ndk	511145.b2518	GO Process	GO:0009201	Ribonucleoside triphosphate biosynthetic process
ndk	511145.b2518	GO Process	GO:0009205	Purine ribonucleoside triphosphate metabolic process
ndk	511145.b2518	GO Process	GO:0009206	Purine ribonucleoside triphosphate biosynthetic process
ndk	511145.b2518	GO Process	GO:0009208	Pyrimidine ribonucleoside triphosphate metabolic process
ndk	511145.b2518	GO Process	GO:0009209	Pyrimidine ribonucleoside triphosphate biosynthetic process
ndk	511145.b2518	GO Process	GO:0009218	Pyrimidine ribonucleotide metabolic process
ndk	511145.b2518	GO Process	GO:0009220	Pyrimidine ribonucleotide biosynthetic process
ndk	511145.b2518	GO Process	GO:0009259	Ribonucleotide metabolic process
ndk	511145.b2518	GO Process	GO:0009260	Ribonucleotide biosynthetic process
ndk	511145.b2518	GO Process	GO:0009987	Cellular process
ndk	511145.b2518	GO Process	GO:0016310	Phosphorylation
ndk	511145.b2518	GO Process	GO:0018130	Heterocycle biosynthetic process
ndk	511145.b2518	GO Process	GO:0019438	Aromatic compound biosynthetic process
ndk	511145.b2518	GO Process	GO:0019637	Organophosphate metabolic process
ndk	511145.b2518	GO Process	GO:0019693	Ribose phosphate metabolic process
ndk	511145.b2518	GO Process	GO:0034641	Cellular nitrogen compound metabolic process
ndk	511145.b2518	GO Process	GO:0034654	Nucleobase-containing compound biosynthetic process
ndk	511145.b2518	GO Process	GO:0044237	Cellular metabolic process
ndk	511145.b2518	GO Process	GO:0044238	Primary metabolic process
ndk	511145.b2518	GO Process	GO:0044249	Cellular biosynthetic process
ndk	511145.b2518	GO Process	GO:0044271	Cellular nitrogen compound biosynthetic process
ndk	511145.b2518	GO Process	GO:0044281	Small molecule metabolic process
ndk	511145.b2518	GO Process	GO:0046036	CTP metabolic process
ndk	511145.b2518	GO Process	GO:0046039	GTP metabolic process
ndk	511145.b2518	GO Process	GO:0046051	UTP metabolic process
ndk	511145.b2518	GO Process	GO:0046390	Ribose phosphate biosynthetic process
ndk	511145.b2518	GO Process	GO:0046483	Heterocycle metabolic process
ndk	511145.b2518	GO Process	GO:0046939	Nucleotide phosphorylation
ndk	511145.b2518	GO Process	GO:0055086	Nucleobase-containing small molecule metabolic process
ndk	511145.b2518	GO Process	GO:0071704	Organic substance metabolic process
ndk	511145.b2518	GO Process	GO:0072521	Purine-containing compound metabolic process
ndk	511145.b2518	GO Process	GO:0072522	Purine-containing compound biosynthetic process
ndk	511145.b2518	GO Process	GO:0072527	Pyrimidine-containing compound metabolic process
ndk	511145.b2518	GO Process	GO:0072528	Pyrimidine-containing compound biosynthetic process
ndk	511145.b2518	GO Process	GO:0090407	Organophosphate biosynthetic process
ndk	511145.b2518	GO Process	GO:1901135	Carbohydrate derivative metabolic process
ndk	511145.b2518	GO Process	GO:1901137	Carbohydrate derivative biosynthetic process
ndk	511145.b2518	GO Process	GO:1901293	Nucleoside phosphate biosynthetic process
ndk	511145.b2518	GO Process	GO:1901360	Organic cyclic compound metabolic process
ndk	511145.b2518	GO Process	GO:1901362	Organic cyclic compound biosynthetic process
ndk	511145.b2518	GO Process	GO:1901564	Organonitrogen compound metabolic process
ndk	511145.b2518	GO Process	GO:1901566	Organonitrogen compound biosynthetic process
ndk	511145.b2518	GO Process	GO:1901576	Organic substance biosynthetic process
ndk	511145.b2518	GO Function	GO:0000166	Nucleotide binding
ndk	511145.b2518	GO Function	GO:0003824	Catalytic activity
ndk	511145.b2518	GO Function	GO:0004550	Nucleoside diphosphate kinase activity
ndk	511145.b2518	GO Function	GO:0005488	Binding
ndk	511145.b2518	GO Function	GO:0005524	ATP binding
ndk	511145.b2518	GO Function	GO:0016301	Kinase activity
ndk	511145.b2518	GO Function	GO:0016740	Transferase activity
ndk	511145.b2518	GO Function	GO:0016772	Transferase activity, transferring phosphorus-containing groups
ndk	511145.b2518	GO Function	GO:0016776	Phosphotransferase activity, phosphate group as acceptor
ndk	511145.b2518	GO Function	GO:0017076	Purine nucleotide binding
ndk	511145.b2518	GO Function	GO:0019205	Nucleobase-containing compound kinase activity
ndk	511145.b2518	GO Function	GO:0030554	Adenyl nucleotide binding
ndk	511145.b2518	GO Function	GO:0032553	Ribonucleotide binding
ndk	511145.b2518	GO Function	GO:0032555	Purine ribonucleotide binding
ndk	511145.b2518	GO Function	GO:0032559	Adenyl ribonucleotide binding
ndk	511145.b2518	GO Function	GO:0035639	Purine ribonucleoside triphosphate binding
ndk	511145.b2518	GO Function	GO:0036094	Small molecule binding
ndk	511145.b2518	GO Function	GO:0043167	Ion binding
ndk	511145.b2518	GO Function	GO:0043168	Anion binding
ndk	511145.b2518	GO Function	GO:0043169	Cation binding
ndk	511145.b2518	GO Function	GO:0046872	Metal ion binding
ndk	511145.b2518	GO Function	GO:0097159	Organic cyclic compound binding
ndk	511145.b2518	GO Function	GO:0097367	Carbohydrate derivative binding
ndk	511145.b2518	GO Function	GO:1901265	Nucleoside phosphate binding
ndk	511145.b2518	GO Function	GO:1901363	Heterocyclic compound binding
ndk	511145.b2518	GO Component	GO:0005622	Intracellular anatomical structure
ndk	511145.b2518	GO Component	GO:0005737	Cytoplasm
ndk	511145.b2518	GO Component	GO:0005829	Cytosol
ndk	511145.b2518	GO Component	GO:0110165	Cellular anatomical entity
ndk	511145.b2518	STRING clusters	CL:254	Pyrimidine nucleoside triphosphate biosynthetic process, and Adenylate kinase activity
ndk	511145.b2518	STRING clusters	CL:35	Translation, and Protein export
ndk	511145.b2518	STRING clusters	CL:37	Translation, and Protein export
ndk	511145.b2518	STRING clusters	CL:39	Mixed, incl. Ribonucleoprotein, and Translation regulator activity
ndk	511145.b2518	STRING clusters	CL:40	Ribonucleoprotein, and Translation factor activity, RNA binding
ndk	511145.b2518	KEGG	eco00230	Purine metabolism
ndk	511145.b2518	KEGG	eco00240	Pyrimidine metabolism
ndk	511145.b2518	KEGG	eco01100	Metabolic pathways
ndk	511145.b2518	KEGG	eco01110	Biosynthesis of secondary metabolites
ndk	511145.b2518	COMPARTMENTS	GOCC:0005622	Intracellular
ndk	511145.b2518	COMPARTMENTS	GOCC:0005737	Cytoplasm
ndk	511145.b2518	COMPARTMENTS	GOCC:0005829	Cytosol
ndk	511145.b2518	COMPARTMENTS	GOCC:0110165	Cellular anatomical entity
ndk	511145.b2518	UniProt Keywords	KW-0067	ATP-binding
ndk	511145.b2518	UniProt Keywords	KW-0418	Kinase
ndk	511145.b2518	UniProt Keywords	KW-0460	Magnesium
ndk	511145.b2518	UniProt Keywords	KW-0479	Metal-binding
ndk	511145.b2518	UniProt Keywords	KW-0546	Nucleotide metabolism
ndk	511145.b2518	UniProt Keywords	KW-0547	Nucleotide-binding
ndk	511145.b2518	UniProt Keywords	KW-0597	Phosphoprotein
ndk	511145.b2518	UniProt Keywords	KW-0808	Transferase
ndk	511145.b2518	UniProt Keywords	KW-0963	Cytoplasm
ndk	511145.b2518	InterPro	IPR001564	Nucleoside diphosphate kinase
ndk	511145.b2518	InterPro	IPR023005	Nucleoside diphosphate kinase, active site
ndk	511145.b2518	InterPro	IPR034907	Nucleoside diphosphate kinase-like domain
ndk	511145.b2518	InterPro	IPR036850	Nucleoside diphosphate kinase-like domain superfamily
ndk	511145.b2518	SMART	SM00562	These are enzymes that catalyze nonsubstrate specific conversions of nucleoside diphosphates to nucleoside triphosphates. These enzymes play important roles in bacterial growth, signal transduction and pathogenicity.
nfuA	511145.b3414	GO Process	GO:0006790	Sulfur compound metabolic process
nfuA	511145.b3414	GO Process	GO:0006807	Nitrogen compound metabolic process
nfuA	511145.b3414	GO Process	GO:0006873	Cellular ion homeostasis
nfuA	511145.b3414	GO Process	GO:0006875	Cellular metal ion homeostasis
nfuA	511145.b3414	GO Process	GO:0006879	Cellular iron ion homeostasis
nfuA	511145.b3414	GO Process	GO:0006950	Response to stress
nfuA	511145.b3414	GO Process	GO:0006979	Response to oxidative stress
nfuA	511145.b3414	GO Process	GO:0007154	Cell communication
nfuA	511145.b3414	GO Process	GO:0008152	Metabolic process
nfuA	511145.b3414	GO Process	GO:0009267	Cellular response to starvation
nfuA	511145.b3414	GO Process	GO:0009605	Response to external stimulus
nfuA	511145.b3414	GO Process	GO:0009987	Cellular process
nfuA	511145.b3414	GO Process	GO:0009991	Response to extracellular stimulus
nfuA	511145.b3414	GO Process	GO:0010106	Cellular response to iron ion starvation
nfuA	511145.b3414	GO Process	GO:0010467	Gene expression
nfuA	511145.b3414	GO Process	GO:0015976	Carbon utilization
nfuA	511145.b3414	GO Process	GO:0016043	Cellular component organization
nfuA	511145.b3414	GO Process	GO:0016226	Iron-sulfur cluster assembly
nfuA	511145.b3414	GO Process	GO:0019538	Protein metabolic process
nfuA	511145.b3414	GO Process	GO:0019725	Cellular homeostasis
nfuA	511145.b3414	GO Process	GO:0022607	Cellular component assembly
nfuA	511145.b3414	GO Process	GO:0030003	Cellular cation homeostasis
nfuA	511145.b3414	GO Process	GO:0031163	Metallo-sulfur cluster assembly
nfuA	511145.b3414	GO Process	GO:0031667	Response to nutrient levels
nfuA	511145.b3414	GO Process	GO:0031668	Cellular response to extracellular stimulus
nfuA	511145.b3414	GO Process	GO:0031669	Cellular response to nutrient levels
nfuA	511145.b3414	GO Process	GO:0033554	Cellular response to stress
nfuA	511145.b3414	GO Process	GO:0042592	Homeostatic process
nfuA	511145.b3414	GO Process	GO:0042594	Response to starvation
nfuA	511145.b3414	GO Process	GO:0043170	Macromolecule metabolic process
nfuA	511145.b3414	GO Process	GO:0044085	Cellular component biogenesis
nfuA	511145.b3414	GO Process	GO:0044237	Cellular metabolic process
nfuA	511145.b3414	GO Process	GO:0044238	Primary metabolic process
nfuA	511145.b3414	GO Process	GO:0046916	Cellular transition metal ion homeostasis
nfuA	511145.b3414	GO Process	GO:0048878	Chemical homeostasis
nfuA	511145.b3414	GO Process	GO:0050801	Ion homeostasis
nfuA	511145.b3414	GO Process	GO:0050896	Response to stimulus
nfuA	511145.b3414	GO Process	GO:0051604	Protein maturation
nfuA	511145.b3414	GO Process	GO:0051716	Cellular response to stimulus
nfuA	511145.b3414	GO Process	GO:0055065	Metal ion homeostasis
nfuA	511145.b3414	GO Process	GO:0055072	Iron ion homeostasis
nfuA	511145.b3414	GO Process	GO:0055076	Transition metal ion homeostasis
nfuA	511145.b3414	GO Process	GO:0055080	Cation homeostasis
nfuA	511145.b3414	GO Process	GO:0055082	Cellular chemical homeostasis
nfuA	511145.b3414	GO Process	GO:0065007	Biological regulation
nfuA	511145.b3414	GO Process	GO:0065008	Regulation of biological quality
nfuA	511145.b3414	GO Process	GO:0071496	Cellular response to external stimulus
nfuA	511145.b3414	GO Process	GO:0071704	Organic substance metabolic process
nfuA	511145.b3414	GO Process	GO:0071840	Cellular component organization or biogenesis
nfuA	511145.b3414	GO Process	GO:0097428	Protein maturation by iron-sulfur cluster transfer
nfuA	511145.b3414	GO Process	GO:0098771	Inorganic ion homeostasis
nfuA	511145.b3414	GO Process	GO:0106035	Protein maturation by [4Fe-4S] cluster transfer
nfuA	511145.b3414	GO Process	GO:1901564	Organonitrogen compound metabolic process
nfuA	511145.b3414	GO Function	GO:0005488	Binding
nfuA	511145.b3414	GO Function	GO:0005506	Iron ion binding
nfuA	511145.b3414	GO Function	GO:0043167	Ion binding
nfuA	511145.b3414	GO Function	GO:0043169	Cation binding
nfuA	511145.b3414	GO Function	GO:0046872	Metal ion binding
nfuA	511145.b3414	GO Function	GO:0046914	Transition metal ion binding
nfuA	511145.b3414	GO Function	GO:0051536	Iron-sulfur cluster binding
nfuA	511145.b3414	GO Function	GO:0051539	4 iron, 4 sulfur cluster binding
nfuA	511145.b3414	GO Function	GO:0051540	Metal cluster binding
nfuA	511145.b3414	GO Function	GO:0140104	Molecular carrier activity
nfuA	511145.b3414	GO Function	GO:0140132	Iron-sulfur cluster carrier activity
nfuA	511145.b3414	GO Component	GO:0005622	Intracellular anatomical structure
nfuA	511145.b3414	GO Component	GO:0005737	Cytoplasm
nfuA	511145.b3414	GO Component	GO:0005829	Cytosol
nfuA	511145.b3414	GO Component	GO:0110165	Cellular anatomical entity
nfuA	511145.b3414	STRING clusters	CL:1242	Mixed, incl. Iron-sulfur cluster assembly, and Antioxidant activity
nfuA	511145.b3414	STRING clusters	CL:1243	Mixed, incl. Iron-sulfur cluster assembly, and Antioxidant activity
nfuA	511145.b3414	STRING clusters	CL:1327	Iron-sulfur cluster assembly, and tRNA thio-modification
nfuA	511145.b3414	STRING clusters	CL:1328	Iron-sulfur cluster assembly
nfuA	511145.b3414	STRING clusters	CL:1329	Protein maturation by iron-sulfur cluster transfer, and IscS-IscU complex
nfuA	511145.b3414	COMPARTMENTS	GOCC:0005622	Intracellular
nfuA	511145.b3414	COMPARTMENTS	GOCC:0005737	Cytoplasm
nfuA	511145.b3414	COMPARTMENTS	GOCC:0005829	Cytosol
nfuA	511145.b3414	COMPARTMENTS	GOCC:0110165	Cellular anatomical entity
nfuA	511145.b3414	UniProt Keywords	KW-0004	4Fe-4S
nfuA	511145.b3414	UniProt Keywords	KW-0346	Stress response
nfuA	511145.b3414	UniProt Keywords	KW-0408	Iron
nfuA	511145.b3414	UniProt Keywords	KW-0411	Iron-sulfur
nfuA	511145.b3414	UniProt Keywords	KW-0479	Metal-binding
nfuA	511145.b3414	Pfam	PF01106	NifU-like domain
nfuA	511145.b3414	Pfam	PF01521	Iron-sulphur cluster biosynthesis
nfuA	511145.b3414	InterPro	IPR000361	FeS cluster biogenesis
nfuA	511145.b3414	InterPro	IPR001075	NIF system FeS cluster assembly, NifU, C-terminal
nfuA	511145.b3414	InterPro	IPR017726	Fe/S biogenesis protein NfuA
nfuA	511145.b3414	InterPro	IPR034904	Fe-S cluster assembly domain superfamily
nfuA	511145.b3414	InterPro	IPR035903	HesB-like domain superfamily
nrdR	511145.b0413	GO Process	GO:0006355	Regulation of transcription, DNA-templated
nrdR	511145.b0413	GO Process	GO:0009889	Regulation of biosynthetic process
nrdR	511145.b0413	GO Process	GO:0009890	Negative regulation of biosynthetic process
nrdR	511145.b0413	GO Process	GO:0009892	Negative regulation of metabolic process
nrdR	511145.b0413	GO Process	GO:0010468	Regulation of gene expression
nrdR	511145.b0413	GO Process	GO:0010556	Regulation of macromolecule biosynthetic process
nrdR	511145.b0413	GO Process	GO:0010558	Negative regulation of macromolecule biosynthetic process
nrdR	511145.b0413	GO Process	GO:0010605	Negative regulation of macromolecule metabolic process
nrdR	511145.b0413	GO Process	GO:0019219	Regulation of nucleobase-containing compound metabolic process
nrdR	511145.b0413	GO Process	GO:0019222	Regulation of metabolic process
nrdR	511145.b0413	GO Process	GO:0031323	Regulation of cellular metabolic process
nrdR	511145.b0413	GO Process	GO:0031324	Negative regulation of cellular metabolic process
nrdR	511145.b0413	GO Process	GO:0031326	Regulation of cellular biosynthetic process
nrdR	511145.b0413	GO Process	GO:0031327	Negative regulation of cellular biosynthetic process
nrdR	511145.b0413	GO Process	GO:0045892	Negative regulation of transcription, DNA-templated
nrdR	511145.b0413	GO Process	GO:0045934	Negative regulation of nucleobase-containing compound metabolic process
nrdR	511145.b0413	GO Process	GO:0048519	Negative regulation of biological process
nrdR	511145.b0413	GO Process	GO:0048523	Negative regulation of cellular process
nrdR	511145.b0413	GO Process	GO:0050789	Regulation of biological process
nrdR	511145.b0413	GO Process	GO:0050794	Regulation of cellular process
nrdR	511145.b0413	GO Process	GO:0051171	Regulation of nitrogen compound metabolic process
nrdR	511145.b0413	GO Process	GO:0051172	Negative regulation of nitrogen compound metabolic process
nrdR	511145.b0413	GO Process	GO:0051252	Regulation of RNA metabolic process
nrdR	511145.b0413	GO Process	GO:0051253	Negative regulation of RNA metabolic process
nrdR	511145.b0413	GO Process	GO:0060255	Regulation of macromolecule metabolic process
nrdR	511145.b0413	GO Process	GO:0065007	Biological regulation
nrdR	511145.b0413	GO Process	GO:0080090	Regulation of primary metabolic process
nrdR	511145.b0413	GO Process	GO:1902679	Negative regulation of RNA biosynthetic process
nrdR	511145.b0413	GO Process	GO:1903506	Regulation of nucleic acid-templated transcription
nrdR	511145.b0413	GO Process	GO:1903507	Negative regulation of nucleic acid-templated transcription
nrdR	511145.b0413	GO Process	GO:2001141	Regulation of RNA biosynthetic process
nrdR	511145.b0413	GO Function	GO:0000166	Nucleotide binding
nrdR	511145.b0413	GO Function	GO:0003676	Nucleic acid binding
nrdR	511145.b0413	GO Function	GO:0003677	DNA binding
nrdR	511145.b0413	GO Function	GO:0003690	Double-stranded DNA binding
nrdR	511145.b0413	GO Function	GO:0005488	Binding
nrdR	511145.b0413	GO Function	GO:0005524	ATP binding
nrdR	511145.b0413	GO Function	GO:0008270	Zinc ion binding
nrdR	511145.b0413	GO Function	GO:0017076	Purine nucleotide binding
nrdR	511145.b0413	GO Function	GO:0030554	Adenyl nucleotide binding
nrdR	511145.b0413	GO Function	GO:0032553	Ribonucleotide binding
nrdR	511145.b0413	GO Function	GO:0032555	Purine ribonucleotide binding
nrdR	511145.b0413	GO Function	GO:0032559	Adenyl ribonucleotide binding
nrdR	511145.b0413	GO Function	GO:0035639	Purine ribonucleoside triphosphate binding
nrdR	511145.b0413	GO Function	GO:0036094	Small molecule binding
nrdR	511145.b0413	GO Function	GO:0043167	Ion binding
nrdR	511145.b0413	GO Function	GO:0043168	Anion binding
nrdR	511145.b0413	GO Function	GO:0043169	Cation binding
nrdR	511145.b0413	GO Function	GO:0046872	Metal ion binding
nrdR	511145.b0413	GO Function	GO:0046914	Transition metal ion binding
nrdR	511145.b0413	GO Function	GO:0097159	Organic cyclic compound binding
nrdR	511145.b0413	GO Function	GO:0097367	Carbohydrate derivative binding
nrdR	511145.b0413	GO Function	GO:1901265	Nucleoside phosphate binding
nrdR	511145.b0413	GO Function	GO:1901363	Heterocyclic compound binding
nrdR	511145.b0413	STRING clusters	CL:1242	Mixed, incl. Iron-sulfur cluster assembly, and Antioxidant activity
nrdR	511145.b0413	STRING clusters	CL:1382	Mixed, incl. Oxidoreductase activity, acting on CH or CH2 groups, disulfide as acceptor, and Flavodoxin, long chain
nrdR	511145.b0413	STRING clusters	CL:1383	Mixed, incl. Oxidoreductase activity, acting on CH or CH2 groups, disulfide as acceptor, and Regulation of oxidoreductase activity
nrdR	511145.b0413	UniProt Keywords	KW-0067	ATP-binding
nrdR	511145.b0413	UniProt Keywords	KW-0238	DNA-binding
nrdR	511145.b0413	UniProt Keywords	KW-0479	Metal-binding
nrdR	511145.b0413	UniProt Keywords	KW-0547	Nucleotide-binding
nrdR	511145.b0413	UniProt Keywords	KW-0678	Repressor
nrdR	511145.b0413	UniProt Keywords	KW-0804	Transcription
nrdR	511145.b0413	UniProt Keywords	KW-0805	Transcription regulation
nrdR	511145.b0413	UniProt Keywords	KW-0862	Zinc
nrdR	511145.b0413	UniProt Keywords	KW-0863	Zinc-finger
nrdR	511145.b0413	Pfam	PF03477	ATP cone domain
nrdR	511145.b0413	InterPro	IPR003796	Ribonucleotide reductase regulator NrdR-like
nrdR	511145.b0413	InterPro	IPR005144	ATP-cone domain
obgE	511145.b3183	GO Process	GO:0006996	Organelle organization
obgE	511145.b3183	GO Process	GO:0007059	Chromosome segregation
obgE	511145.b3183	GO Process	GO:0009987	Cellular process
obgE	511145.b3183	GO Process	GO:0016043	Cellular component organization
obgE	511145.b3183	GO Process	GO:0022607	Cellular component assembly
obgE	511145.b3183	GO Process	GO:0022611	Dormancy process
obgE	511145.b3183	GO Process	GO:0022613	Ribonucleoprotein complex biogenesis
obgE	511145.b3183	GO Process	GO:0032502	Developmental process
obgE	511145.b3183	GO Process	GO:0042254	Ribosome biogenesis
obgE	511145.b3183	GO Process	GO:0042255	Ribosome assembly
obgE	511145.b3183	GO Process	GO:0044085	Cellular component biogenesis
obgE	511145.b3183	GO Process	GO:0044087	Regulation of cellular component biogenesis
obgE	511145.b3183	GO Process	GO:0048519	Negative regulation of biological process
obgE	511145.b3183	GO Process	GO:0048523	Negative regulation of cellular process
obgE	511145.b3183	GO Process	GO:0050789	Regulation of biological process
obgE	511145.b3183	GO Process	GO:0050794	Regulation of cellular process
obgE	511145.b3183	GO Process	GO:0065007	Biological regulation
obgE	511145.b3183	GO Process	GO:0070925	Organelle assembly
obgE	511145.b3183	GO Process	GO:0071840	Cellular component organization or biogenesis
obgE	511145.b3183	GO Process	GO:0090069	Regulation of ribosome biogenesis
obgE	511145.b3183	GO Process	GO:0090071	Negative regulation of ribosome biogenesis
obgE	511145.b3183	GO Process	GO:0140694	Non-membrane-bounded organelle assembly
obgE	511145.b3183	GO Function	GO:0000166	Nucleotide binding
obgE	511145.b3183	GO Function	GO:0000287	Magnesium ion binding
obgE	511145.b3183	GO Function	GO:0003676	Nucleic acid binding
obgE	511145.b3183	GO Function	GO:0003677	DNA binding
obgE	511145.b3183	GO Function	GO:0003723	RNA binding
obgE	511145.b3183	GO Function	GO:0003824	Catalytic activity
obgE	511145.b3183	GO Function	GO:0003924	GTPase activity
obgE	511145.b3183	GO Function	GO:0005488	Binding
obgE	511145.b3183	GO Function	GO:0005525	GTP binding
obgE	511145.b3183	GO Function	GO:0016462	Pyrophosphatase activity
obgE	511145.b3183	GO Function	GO:0016787	Hydrolase activity
obgE	511145.b3183	GO Function	GO:0016817	Hydrolase activity, acting on acid anhydrides
obgE	511145.b3183	GO Function	GO:0016818	Hydrolase activity, acting on acid anhydrides, in phosphorus-containing anhydrides
obgE	511145.b3183	GO Function	GO:0017076	Purine nucleotide binding
obgE	511145.b3183	GO Function	GO:0017111	Nucleoside-triphosphatase activity
obgE	511145.b3183	GO Function	GO:0019001	Guanyl nucleotide binding
obgE	511145.b3183	GO Function	GO:0019003	GDP binding
obgE	511145.b3183	GO Function	GO:0019843	rRNA binding
obgE	511145.b3183	GO Function	GO:0032553	Ribonucleotide binding
obgE	511145.b3183	GO Function	GO:0032555	Purine ribonucleotide binding
obgE	511145.b3183	GO Function	GO:0032561	Guanyl ribonucleotide binding
obgE	511145.b3183	GO Function	GO:0035639	Purine ribonucleoside triphosphate binding
obgE	511145.b3183	GO Function	GO:0036094	Small molecule binding
obgE	511145.b3183	GO Function	GO:0043021	Ribonucleoprotein complex binding
obgE	511145.b3183	GO Function	GO:0043023	Ribosomal large subunit binding
obgE	511145.b3183	GO Function	GO:0043167	Ion binding
obgE	511145.b3183	GO Function	GO:0043168	Anion binding
obgE	511145.b3183	GO Function	GO:0043169	Cation binding
obgE	511145.b3183	GO Function	GO:0044877	Protein-containing complex binding
obgE	511145.b3183	GO Function	GO:0046872	Metal ion binding
obgE	511145.b3183	GO Function	GO:0097159	Organic cyclic compound binding
obgE	511145.b3183	GO Function	GO:0097216	Guanosine tetraphosphate binding
obgE	511145.b3183	GO Function	GO:0097367	Carbohydrate derivative binding
obgE	511145.b3183	GO Function	GO:1901265	Nucleoside phosphate binding
obgE	511145.b3183	GO Function	GO:1901363	Heterocyclic compound binding
obgE	511145.b3183	GO Component	GO:0005622	Intracellular anatomical structure
obgE	511145.b3183	GO Component	GO:0005737	Cytoplasm
obgE	511145.b3183	GO Component	GO:0005829	Cytosol
obgE	511145.b3183	GO Component	GO:0110165	Cellular anatomical entity
obgE	511145.b3183	STRING clusters	CL:378	Mixed, incl. rRNA modification, and tRNA methylation
obgE	511145.b3183	STRING clusters	CL:379	rRNA modification, and tRNA methylation
obgE	511145.b3183	STRING clusters	CL:380	RNA methylation, and GTP binding domain
obgE	511145.b3183	STRING clusters	CL:416	Mixed, incl. GTP binding domain, and S4 RNA-binding domain
obgE	511145.b3183	STRING clusters	CL:419	Negative regulation of ribosome biogenesis, and Pseudouridine synthase, RluC/RluD
obgE	511145.b3183	COMPARTMENTS	GOCC:0005622	Intracellular
obgE	511145.b3183	COMPARTMENTS	GOCC:0005737	Cytoplasm
obgE	511145.b3183	COMPARTMENTS	GOCC:0005829	Cytosol
obgE	511145.b3183	COMPARTMENTS	GOCC:0110165	Cellular anatomical entity
obgE	511145.b3183	UniProt Keywords	KW-0238	DNA-binding
obgE	511145.b3183	UniProt Keywords	KW-0342	GTP-binding
obgE	511145.b3183	UniProt Keywords	KW-0378	Hydrolase
obgE	511145.b3183	UniProt Keywords	KW-0460	Magnesium
obgE	511145.b3183	UniProt Keywords	KW-0479	Metal-binding
obgE	511145.b3183	UniProt Keywords	KW-0547	Nucleotide-binding
obgE	511145.b3183	UniProt Keywords	KW-0694	RNA-binding
obgE	511145.b3183	UniProt Keywords	KW-0699	rRNA-binding
obgE	511145.b3183	UniProt Keywords	KW-0963	Cytoplasm
obgE	511145.b3183	Pfam	PF01018	GTP1/OBG
obgE	511145.b3183	Pfam	PF01926	50S ribosome-binding GTPase
obgE	511145.b3183	InterPro	IPR006073	GTP binding domain
obgE	511145.b3183	InterPro	IPR006074	GTP1/OBG, conserved site
obgE	511145.b3183	InterPro	IPR006169	GTP1/OBG domain
obgE	511145.b3183	InterPro	IPR014100	GTP-binding protein Obg/CgtA
obgE	511145.b3183	InterPro	IPR027417	P-loop containing nucleoside triphosphate hydrolase
obgE	511145.b3183	InterPro	IPR031167	OBG-type guanine nucleotide-binding (G) domain
obgE	511145.b3183	InterPro	IPR036726	GTP1/OBG domain superfamily
obgE	511145.b3183	InterPro	IPR045086	OBG-type GTPase
orn	511145.b4162	GO Process	GO:0006139	Nucleobase-containing compound metabolic process
orn	511145.b4162	GO Process	GO:0006259	DNA metabolic process
orn	511145.b4162	GO Process	GO:0006401	RNA catabolic process
orn	511145.b4162	GO Process	GO:0006725	Cellular aromatic compound metabolic process
orn	511145.b4162	GO Process	GO:0006807	Nitrogen compound metabolic process
orn	511145.b4162	GO Process	GO:0008152	Metabolic process
orn	511145.b4162	GO Process	GO:0009056	Catabolic process
orn	511145.b4162	GO Process	GO:0009057	Macromolecule catabolic process
orn	511145.b4162	GO Process	GO:0009987	Cellular process
orn	511145.b4162	GO Process	GO:0016070	RNA metabolic process
orn	511145.b4162	GO Process	GO:0019439	Aromatic compound catabolic process
orn	511145.b4162	GO Process	GO:0034641	Cellular nitrogen compound metabolic process
orn	511145.b4162	GO Process	GO:0034655	Nucleobase-containing compound catabolic process
orn	511145.b4162	GO Process	GO:0043170	Macromolecule metabolic process
orn	511145.b4162	GO Process	GO:0044237	Cellular metabolic process
orn	511145.b4162	GO Process	GO:0044238	Primary metabolic process
orn	511145.b4162	GO Process	GO:0044248	Cellular catabolic process
orn	511145.b4162	GO Process	GO:0044260	Cellular macromolecule metabolic process
orn	511145.b4162	GO Process	GO:0044265	Cellular macromolecule catabolic process
orn	511145.b4162	GO Process	GO:0044270	Cellular nitrogen compound catabolic process
orn	511145.b4162	GO Process	GO:0046483	Heterocycle metabolic process
orn	511145.b4162	GO Process	GO:0046700	Heterocycle catabolic process
orn	511145.b4162	GO Process	GO:0071704	Organic substance metabolic process
orn	511145.b4162	GO Process	GO:0090304	Nucleic acid metabolic process
orn	511145.b4162	GO Process	GO:0090305	Nucleic acid phosphodiester bond hydrolysis
orn	511145.b4162	GO Process	GO:0090501	RNA phosphodiester bond hydrolysis
orn	511145.b4162	GO Process	GO:0090503	RNA phosphodiester bond hydrolysis, exonucleolytic
orn	511145.b4162	GO Process	GO:1901360	Organic cyclic compound metabolic process
orn	511145.b4162	GO Process	GO:1901361	Organic cyclic compound catabolic process
orn	511145.b4162	GO Process	GO:1901575	Organic substance catabolic process
orn	511145.b4162	GO Function	GO:0000175	3-5-exoribonuclease activity
orn	511145.b4162	GO Function	GO:0003676	Nucleic acid binding
orn	511145.b4162	GO Function	GO:0003824	Catalytic activity
orn	511145.b4162	GO Function	GO:0004518	Nuclease activity
orn	511145.b4162	GO Function	GO:0004527	Exonuclease activity
orn	511145.b4162	GO Function	GO:0004529	Exodeoxyribonuclease activity
orn	511145.b4162	GO Function	GO:0004532	Exoribonuclease activity
orn	511145.b4162	GO Function	GO:0004536	Deoxyribonuclease activity
orn	511145.b4162	GO Function	GO:0004540	Ribonuclease activity
orn	511145.b4162	GO Function	GO:0005488	Binding
orn	511145.b4162	GO Function	GO:0008296	3-5-exodeoxyribonuclease activity
orn	511145.b4162	GO Function	GO:0008297	Single-stranded DNA exodeoxyribonuclease activity
orn	511145.b4162	GO Function	GO:0008310	Single-stranded DNA 3-5 exodeoxyribonuclease activity
orn	511145.b4162	GO Function	GO:0008408	3-5 exonuclease activity
orn	511145.b4162	GO Function	GO:0008946	Oligonucleotidase activity
orn	511145.b4162	GO Function	GO:0016787	Hydrolase activity
orn	511145.b4162	GO Function	GO:0016788	Hydrolase activity, acting on ester bonds
orn	511145.b4162	GO Function	GO:0016796	Exonuclease activity, active with either ribo- or deoxyribonucleic acids and producing 5-phosphomonoesters
orn	511145.b4162	GO Function	GO:0016895	Exodeoxyribonuclease activity, producing 5-phosphomonoesters
orn	511145.b4162	GO Function	GO:0016896	Exoribonuclease activity, producing 5-phosphomonoesters
orn	511145.b4162	GO Function	GO:0034611	Oligoribonucleotidase activity
orn	511145.b4162	GO Function	GO:0043167	Ion binding
orn	511145.b4162	GO Function	GO:0043169	Cation binding
orn	511145.b4162	GO Function	GO:0046872	Metal ion binding
orn	511145.b4162	GO Function	GO:0097159	Organic cyclic compound binding
orn	511145.b4162	GO Function	GO:0140097	Catalytic activity, acting on DNA
orn	511145.b4162	GO Function	GO:0140098	Catalytic activity, acting on RNA
orn	511145.b4162	GO Function	GO:0140640	Catalytic activity, acting on a nucleic acid
orn	511145.b4162	GO Function	GO:1901363	Heterocyclic compound binding
orn	511145.b4162	GO Component	GO:0005622	Intracellular anatomical structure
orn	511145.b4162	GO Component	GO:0005737	Cytoplasm
orn	511145.b4162	GO Component	GO:0005829	Cytosol
orn	511145.b4162	GO Component	GO:0110165	Cellular anatomical entity
orn	511145.b4162	STRING clusters	CL:531	Mixed, incl. RNA helicase activity, and Exoribonuclease activity
orn	511145.b4162	STRING clusters	CL:533	Exoribonuclease activity, and RNA degradation
orn	511145.b4162	STRING clusters	CL:553	Exoribonuclease activity
orn	511145.b4162	COMPARTMENTS	GOCC:0005622	Intracellular
orn	511145.b4162	COMPARTMENTS	GOCC:0005737	Cytoplasm
orn	511145.b4162	COMPARTMENTS	GOCC:0005829	Cytosol
orn	511145.b4162	COMPARTMENTS	GOCC:0110165	Cellular anatomical entity
orn	511145.b4162	UniProt Keywords	KW-0269	Exonuclease
orn	511145.b4162	UniProt Keywords	KW-0378	Hydrolase
orn	511145.b4162	UniProt Keywords	KW-0479	Metal-binding
orn	511145.b4162	UniProt Keywords	KW-0540	Nuclease
orn	511145.b4162	UniProt Keywords	KW-0862	Zinc
orn	511145.b4162	UniProt Keywords	KW-0963	Cytoplasm
orn	511145.b4162	InterPro	IPR012337	Ribonuclease H-like superfamily
orn	511145.b4162	InterPro	IPR013520	Exonuclease, RNase T/DNA polymerase III
orn	511145.b4162	InterPro	IPR022894	Oligoribonuclease
orn	511145.b4162	InterPro	IPR036397	Ribonuclease H superfamily
orn	511145.b4162	SMART	SM00479	Exonuclease domain in DNA-polymerase alpha and epsilon chain, ribonuclease T and other exonucleases
patD	511145.b1444	GO Process	GO:0006082	Organic acid metabolic process
patD	511145.b1444	GO Process	GO:0006520	Cellular amino acid metabolic process
patD	511145.b1444	GO Process	GO:0006553	Lysine metabolic process
patD	511145.b1444	GO Process	GO:0006554	Lysine catabolic process
patD	511145.b1444	GO Process	GO:0006576	Cellular biogenic amine metabolic process
patD	511145.b1444	GO Process	GO:0006595	Polyamine metabolic process
patD	511145.b1444	GO Process	GO:0006598	Polyamine catabolic process
patD	511145.b1444	GO Process	GO:0006807	Nitrogen compound metabolic process
patD	511145.b1444	GO Process	GO:0008152	Metabolic process
patD	511145.b1444	GO Process	GO:0009056	Catabolic process
patD	511145.b1444	GO Process	GO:0009063	Cellular amino acid catabolic process
patD	511145.b1444	GO Process	GO:0009066	Aspartate family amino acid metabolic process
patD	511145.b1444	GO Process	GO:0009068	Aspartate family amino acid catabolic process
patD	511145.b1444	GO Process	GO:0009308	Amine metabolic process
patD	511145.b1444	GO Process	GO:0009310	Amine catabolic process
patD	511145.b1444	GO Process	GO:0009445	Putrescine metabolic process
patD	511145.b1444	GO Process	GO:0009447	Putrescine catabolic process
patD	511145.b1444	GO Process	GO:0009987	Cellular process
patD	511145.b1444	GO Process	GO:0016054	Organic acid catabolic process
patD	511145.b1444	GO Process	GO:0019477	L-lysine catabolic process
patD	511145.b1444	GO Process	GO:0019752	Carboxylic acid metabolic process
patD	511145.b1444	GO Process	GO:0034641	Cellular nitrogen compound metabolic process
patD	511145.b1444	GO Process	GO:0042402	Cellular biogenic amine catabolic process
patD	511145.b1444	GO Process	GO:0043436	Oxoacid metabolic process
patD	511145.b1444	GO Process	GO:0044106	Cellular amine metabolic process
patD	511145.b1444	GO Process	GO:0044237	Cellular metabolic process
patD	511145.b1444	GO Process	GO:0044238	Primary metabolic process
patD	511145.b1444	GO Process	GO:0044248	Cellular catabolic process
patD	511145.b1444	GO Process	GO:0044281	Small molecule metabolic process
patD	511145.b1444	GO Process	GO:0044282	Small molecule catabolic process
patD	511145.b1444	GO Process	GO:0046395	Carboxylic acid catabolic process
patD	511145.b1444	GO Process	GO:0046440	L-lysine metabolic process
patD	511145.b1444	GO Process	GO:0071704	Organic substance metabolic process
patD	511145.b1444	GO Process	GO:1901564	Organonitrogen compound metabolic process
patD	511145.b1444	GO Process	GO:1901565	Organonitrogen compound catabolic process
patD	511145.b1444	GO Process	GO:1901575	Organic substance catabolic process
patD	511145.b1444	GO Process	GO:1901605	Alpha-amino acid metabolic process
patD	511145.b1444	GO Process	GO:1901606	Alpha-amino acid catabolic process
patD	511145.b1444	GO Function	GO:0000166	Nucleotide binding
patD	511145.b1444	GO Function	GO:0003824	Catalytic activity
patD	511145.b1444	GO Function	GO:0004029	Aldehyde dehydrogenase (NAD+) activity
patD	511145.b1444	GO Function	GO:0004030	Aldehyde dehydrogenase [NAD(P)+] activity
patD	511145.b1444	GO Function	GO:0005488	Binding
patD	511145.b1444	GO Function	GO:0016491	Oxidoreductase activity
patD	511145.b1444	GO Function	GO:0016620	Oxidoreductase activity, acting on the aldehyde or oxo group of donors, NAD or NADP as acceptor
patD	511145.b1444	GO Function	GO:0016903	Oxidoreductase activity, acting on the aldehyde or oxo group of donors
patD	511145.b1444	GO Function	GO:0019145	Aminobutyraldehyde dehydrogenase activity
patD	511145.b1444	GO Function	GO:0033737	1-pyrroline dehydrogenase activity
patD	511145.b1444	GO Function	GO:0036094	Small molecule binding
patD	511145.b1444	GO Function	GO:0051287	NAD binding
patD	511145.b1444	GO Function	GO:0097159	Organic cyclic compound binding
patD	511145.b1444	GO Function	GO:1901265	Nucleoside phosphate binding
patD	511145.b1444	GO Function	GO:1901363	Heterocyclic compound binding
patD	511145.b1444	GO Component	GO:0005622	Intracellular anatomical structure
patD	511145.b1444	GO Component	GO:0005737	Cytoplasm
patD	511145.b1444	GO Component	GO:0005829	Cytosol
patD	511145.b1444	GO Component	GO:0032991	Protein-containing complex
patD	511145.b1444	GO Component	GO:0110165	Cellular anatomical entity
patD	511145.b1444	STRING clusters	CL:5950	Mixed, incl. Amine metabolic process, and Alcohol dehydrogenase-like, N-terminal
patD	511145.b1444	STRING clusters	CL:6087	Mixed, incl. Polyamine metabolic process, and Arginine catabolic process to succinate
patD	511145.b1444	STRING clusters	CL:6088	Polyamine metabolic process, and Arginine catabolic process to succinate
patD	511145.b1444	STRING clusters	CL:6089	Polyamine metabolic process, and Gamma-aminobutyric acid catabolic process
patD	511145.b1444	STRING clusters	CL:6090	Putrescine catabolic process, and Gamma-aminobutyric acid catabolic process
patD	511145.b1444	STRING clusters	CL:6091	Gamma-aminobutyric acid catabolic process, and Lysine catabolic process
patD	511145.b1444	KEGG	eco00310	Lysine degradation
patD	511145.b1444	KEGG	eco00330	Arginine and proline metabolism
patD	511145.b1444	KEGG	eco00410	beta-Alanine metabolism
patD	511145.b1444	KEGG	eco01100	Metabolic pathways
patD	511145.b1444	KEGG	eco01120	Microbial metabolism in diverse environments
patD	511145.b1444	COMPARTMENTS	GOCC:0005622	Intracellular
patD	511145.b1444	COMPARTMENTS	GOCC:0005737	Cytoplasm
patD	511145.b1444	COMPARTMENTS	GOCC:0005829	Cytosol
patD	511145.b1444	COMPARTMENTS	GOCC:0032991	Protein-containing complex
patD	511145.b1444	COMPARTMENTS	GOCC:0110165	Cellular anatomical entity
patD	511145.b1444	UniProt Keywords	KW-0520	NAD
patD	511145.b1444	UniProt Keywords	KW-0560	Oxidoreductase
patD	511145.b1444	Pfam	PF00171	Aldehyde dehydrogenase family
patD	511145.b1444	InterPro	IPR015590	Aldehyde dehydrogenase domain
patD	511145.b1444	InterPro	IPR015657	Aminobutyraldehyde dehydrogenase
patD	511145.b1444	InterPro	IPR016161	Aldehyde/histidinol dehydrogenase
patD	511145.b1444	InterPro	IPR016162	Aldehyde dehydrogenase, N-terminal
patD	511145.b1444	InterPro	IPR016163	Aldehyde dehydrogenase, C-terminal
patD	511145.b1444	InterPro	IPR017749	Gamma-aminobutyraldehyde dehydrogenase
patD	511145.b1444	InterPro	IPR029510	Aldehyde dehydrogenase, glutamic acid active site
pck	511145.b3403	GO Process	GO:0005975	Carbohydrate metabolic process
pck	511145.b3403	GO Process	GO:0005996	Monosaccharide metabolic process
pck	511145.b3403	GO Process	GO:0006006	Glucose metabolic process
pck	511145.b3403	GO Process	GO:0006094	Gluconeogenesis
pck	511145.b3403	GO Process	GO:0008152	Metabolic process
pck	511145.b3403	GO Process	GO:0009058	Biosynthetic process
pck	511145.b3403	GO Process	GO:0016051	Carbohydrate biosynthetic process
pck	511145.b3403	GO Process	GO:0019318	Hexose metabolic process
pck	511145.b3403	GO Process	GO:0019319	Hexose biosynthetic process
pck	511145.b3403	GO Process	GO:0044238	Primary metabolic process
pck	511145.b3403	GO Process	GO:0044281	Small molecule metabolic process
pck	511145.b3403	GO Process	GO:0044283	Small molecule biosynthetic process
pck	511145.b3403	GO Process	GO:0046364	Monosaccharide biosynthetic process
pck	511145.b3403	GO Process	GO:0071704	Organic substance metabolic process
pck	511145.b3403	GO Process	GO:1901576	Organic substance biosynthetic process
pck	511145.b3403	GO Function	GO:0000166	Nucleotide binding
pck	511145.b3403	GO Function	GO:0000287	Magnesium ion binding
pck	511145.b3403	GO Function	GO:0003824	Catalytic activity
pck	511145.b3403	GO Function	GO:0004611	Phosphoenolpyruvate carboxykinase activity
pck	511145.b3403	GO Function	GO:0004612	Phosphoenolpyruvate carboxykinase (ATP) activity
pck	511145.b3403	GO Function	GO:0005488	Binding
pck	511145.b3403	GO Function	GO:0005509	Calcium ion binding
pck	511145.b3403	GO Function	GO:0005524	ATP binding
pck	511145.b3403	GO Function	GO:0016829	Lyase activity
pck	511145.b3403	GO Function	GO:0016830	Carbon-carbon lyase activity
pck	511145.b3403	GO Function	GO:0016831	Carboxy-lyase activity
pck	511145.b3403	GO Function	GO:0017076	Purine nucleotide binding
pck	511145.b3403	GO Function	GO:0030554	Adenyl nucleotide binding
pck	511145.b3403	GO Function	GO:0032553	Ribonucleotide binding
pck	511145.b3403	GO Function	GO:0032555	Purine ribonucleotide binding
pck	511145.b3403	GO Function	GO:0032559	Adenyl ribonucleotide binding
pck	511145.b3403	GO Function	GO:0035639	Purine ribonucleoside triphosphate binding
pck	511145.b3403	GO Function	GO:0036094	Small molecule binding
pck	511145.b3403	GO Function	GO:0043167	Ion binding
pck	511145.b3403	GO Function	GO:0043168	Anion binding
pck	511145.b3403	GO Function	GO:0043169	Cation binding
pck	511145.b3403	GO Function	GO:0046872	Metal ion binding
pck	511145.b3403	GO Function	GO:0097159	Organic cyclic compound binding
pck	511145.b3403	GO Function	GO:0097367	Carbohydrate derivative binding
pck	511145.b3403	GO Function	GO:1901265	Nucleoside phosphate binding
pck	511145.b3403	GO Function	GO:1901363	Heterocyclic compound binding
pck	511145.b3403	GO Component	GO:0005622	Intracellular anatomical structure
pck	511145.b3403	GO Component	GO:0005737	Cytoplasm
pck	511145.b3403	GO Component	GO:0005829	Cytosol
pck	511145.b3403	GO Component	GO:0110165	Cellular anatomical entity
pck	511145.b3403	STRING clusters	CL:2010	Mixed, incl. Carbon metabolism, and Fatty acid metabolic process
pck	511145.b3403	STRING clusters	CL:2011	Carbon metabolism, and Oxidative phosphorylation
pck	511145.b3403	STRING clusters	CL:2012	Carbon metabolism, and Pyruvate metabolism
pck	511145.b3403	STRING clusters	CL:2013	Carbon metabolism, and Formate C-acetyltransferase activity
pck	511145.b3403	STRING clusters	CL:2109	Pentose phosphate pathway, and Glycolysis
pck	511145.b3403	STRING clusters	CL:2111	Pentose phosphate pathway, and Gluconeogenesis
pck	511145.b3403	STRING clusters	CL:2112	Gluconeogenesis, and Glycolysis
pck	511145.b3403	KEGG	eco00010	Glycolysis / Gluconeogenesis
pck	511145.b3403	KEGG	eco00020	Citrate cycle (TCA cycle)
pck	511145.b3403	KEGG	eco00620	Pyruvate metabolism
pck	511145.b3403	KEGG	eco01100	Metabolic pathways
pck	511145.b3403	KEGG	eco01110	Biosynthesis of secondary metabolites
pck	511145.b3403	KEGG	eco01120	Microbial metabolism in diverse environments
pck	511145.b3403	KEGG	eco01200	Carbon metabolism
pck	511145.b3403	COMPARTMENTS	GOCC:0005622	Intracellular
pck	511145.b3403	COMPARTMENTS	GOCC:0005737	Cytoplasm
pck	511145.b3403	COMPARTMENTS	GOCC:0005829	Cytosol
pck	511145.b3403	COMPARTMENTS	GOCC:0110165	Cellular anatomical entity
pck	511145.b3403	UniProt Keywords	KW-0007	Acetylation
pck	511145.b3403	UniProt Keywords	KW-0021	Allosteric enzyme
pck	511145.b3403	UniProt Keywords	KW-0067	ATP-binding
pck	511145.b3403	UniProt Keywords	KW-0106	Calcium
pck	511145.b3403	UniProt Keywords	KW-0210	Decarboxylase
pck	511145.b3403	UniProt Keywords	KW-0312	Gluconeogenesis
pck	511145.b3403	UniProt Keywords	KW-0456	Lyase
pck	511145.b3403	UniProt Keywords	KW-0460	Magnesium
pck	511145.b3403	UniProt Keywords	KW-0464	Manganese
pck	511145.b3403	UniProt Keywords	KW-0479	Metal-binding
pck	511145.b3403	UniProt Keywords	KW-0547	Nucleotide-binding
pck	511145.b3403	UniProt Keywords	KW-0963	Cytoplasm
pck	511145.b3403	Pfam	PF01293	Phosphoenolpyruvate carboxykinase
pck	511145.b3403	InterPro	IPR001272	Phosphoenolpyruvate carboxykinase, ATP-utilising
pck	511145.b3403	InterPro	IPR008210	Phosphoenolpyruvate carboxykinase, N-terminal
pck	511145.b3403	InterPro	IPR013035	Phosphoenolpyruvate carboxykinase, C-terminal
pck	511145.b3403	InterPro	IPR015994	Phosphoenolpyruvate carboxykinase (ATP), conserved site
pgsA	511145.b1912	GO Process	GO:0006629	Lipid metabolic process
pgsA	511145.b1912	GO Process	GO:0006644	Phospholipid metabolic process
pgsA	511145.b1912	GO Process	GO:0006650	Glycerophospholipid metabolic process
pgsA	511145.b1912	GO Process	GO:0006655	Phosphatidylglycerol biosynthetic process
pgsA	511145.b1912	GO Process	GO:0006793	Phosphorus metabolic process
pgsA	511145.b1912	GO Process	GO:0006796	Phosphate-containing compound metabolic process
pgsA	511145.b1912	GO Process	GO:0008152	Metabolic process
pgsA	511145.b1912	GO Process	GO:0008610	Lipid biosynthetic process
pgsA	511145.b1912	GO Process	GO:0008654	Phospholipid biosynthetic process
pgsA	511145.b1912	GO Process	GO:0009058	Biosynthetic process
pgsA	511145.b1912	GO Process	GO:0009987	Cellular process
pgsA	511145.b1912	GO Process	GO:0019637	Organophosphate metabolic process
pgsA	511145.b1912	GO Process	GO:0044237	Cellular metabolic process
pgsA	511145.b1912	GO Process	GO:0044238	Primary metabolic process
pgsA	511145.b1912	GO Process	GO:0044249	Cellular biosynthetic process
pgsA	511145.b1912	GO Process	GO:0044255	Cellular lipid metabolic process
pgsA	511145.b1912	GO Process	GO:0045017	Glycerolipid biosynthetic process
pgsA	511145.b1912	GO Process	GO:0046471	Phosphatidylglycerol metabolic process
pgsA	511145.b1912	GO Process	GO:0046474	Glycerophospholipid biosynthetic process
pgsA	511145.b1912	GO Process	GO:0046486	Glycerolipid metabolic process
pgsA	511145.b1912	GO Process	GO:0071704	Organic substance metabolic process
pgsA	511145.b1912	GO Process	GO:0090407	Organophosphate biosynthetic process
pgsA	511145.b1912	GO Process	GO:1901576	Organic substance biosynthetic process
pgsA	511145.b1912	GO Function	GO:0003824	Catalytic activity
pgsA	511145.b1912	GO Function	GO:0008444	CDP-diacylglycerol-glycerol-3-phosphate 3-phosphatidyltransferase activity
pgsA	511145.b1912	GO Function	GO:0016740	Transferase activity
pgsA	511145.b1912	GO Function	GO:0016772	Transferase activity, transferring phosphorus-containing groups
pgsA	511145.b1912	GO Function	GO:0016780	Phosphotransferase activity, for other substituted phosphate groups
pgsA	511145.b1912	GO Function	GO:0017169	CDP-alcohol phosphatidyltransferase activity
pgsA	511145.b1912	GO Component	GO:0005886	Plasma membrane
pgsA	511145.b1912	GO Component	GO:0016020	Membrane
pgsA	511145.b1912	GO Component	GO:0016021	Integral component of membrane
pgsA	511145.b1912	GO Component	GO:0031224	Intrinsic component of membrane
pgsA	511145.b1912	GO Component	GO:0071944	Cell periphery
pgsA	511145.b1912	GO Component	GO:0110165	Cellular anatomical entity
pgsA	511145.b1912	STRING clusters	CL:2443	Glycerophospholipid metabolism, and Mechanosensitive ion channel
pgsA	511145.b1912	STRING clusters	CL:2444	Glycerophospholipid metabolism, and Glycerol metabolism
pgsA	511145.b1912	STRING clusters	CL:2445	Glycerolipid metabolic process, and Undecaprenyl-diphosphatase activity
pgsA	511145.b1912	STRING clusters	CL:2447	Phosphatidylglycerol metabolic process, and Macro domain-like
pgsA	511145.b1912	STRING clusters	CL:2449	Phosphatidylglycerol biosynthetic process
pgsA	511145.b1912	KEGG	eco00564	Glycerophospholipid metabolism
pgsA	511145.b1912	KEGG	eco01100	Metabolic pathways
pgsA	511145.b1912	COMPARTMENTS	GOCC:0005886	Plasma membrane
pgsA	511145.b1912	COMPARTMENTS	GOCC:0016020	Membrane
pgsA	511145.b1912	COMPARTMENTS	GOCC:0071944	Cell periphery
pgsA	511145.b1912	COMPARTMENTS	GOCC:0110165	Cellular anatomical entity
pgsA	511145.b1912	UniProt Keywords	KW-0443	Lipid metabolism
pgsA	511145.b1912	UniProt Keywords	KW-0444	Lipid biosynthesis
pgsA	511145.b1912	UniProt Keywords	KW-0472	Membrane
pgsA	511145.b1912	UniProt Keywords	KW-0594	Phospholipid biosynthesis
pgsA	511145.b1912	UniProt Keywords	KW-0808	Transferase
pgsA	511145.b1912	UniProt Keywords	KW-0812	Transmembrane
pgsA	511145.b1912	UniProt Keywords	KW-0997	Cell inner membrane
pgsA	511145.b1912	UniProt Keywords	KW-1003	Cell membrane
pgsA	511145.b1912	UniProt Keywords	KW-1133	Transmembrane helix
pgsA	511145.b1912	UniProt Keywords	KW-1208	Phospholipid metabolism
pgsA	511145.b1912	Pfam	PF01066	CDP-alcohol phosphatidyltransferase
pgsA	511145.b1912	InterPro	IPR000462	CDP-alcohol phosphatidyltransferase
pgsA	511145.b1912	InterPro	IPR004570	CDP-diacylglycerol--glycerol-3-phosphate 3-phosphatidyltransferase
pgsA	511145.b1912	InterPro	IPR023762	CDP-diacylglycerol--glycerol-3-phosphate 3-phosphatidyltransferase, bacterial
pgsA	511145.b1912	InterPro	IPR043130	CDP-alcohol phosphatidyltransferase, transmembrane domain
pncC	511145.b2700	GO Process	GO:0006139	Nucleobase-containing compound metabolic process
pncC	511145.b2700	GO Process	GO:0006725	Cellular aromatic compound metabolic process
pncC	511145.b2700	GO Process	GO:0006753	Nucleoside phosphate metabolic process
pncC	511145.b2700	GO Process	GO:0006793	Phosphorus metabolic process
pncC	511145.b2700	GO Process	GO:0006796	Phosphate-containing compound metabolic process
pncC	511145.b2700	GO Process	GO:0006807	Nitrogen compound metabolic process
pncC	511145.b2700	GO Process	GO:0008152	Metabolic process
pncC	511145.b2700	GO Process	GO:0009058	Biosynthetic process
pncC	511145.b2700	GO Process	GO:0009117	Nucleotide metabolic process
pncC	511145.b2700	GO Process	GO:0009165	Nucleotide biosynthetic process
pncC	511145.b2700	GO Process	GO:0009987	Cellular process
pncC	511145.b2700	GO Process	GO:0018130	Heterocycle biosynthetic process
pncC	511145.b2700	GO Process	GO:0019362	Pyridine nucleotide metabolic process
pncC	511145.b2700	GO Process	GO:0019363	Pyridine nucleotide biosynthetic process
pncC	511145.b2700	GO Process	GO:0019438	Aromatic compound biosynthetic process
pncC	511145.b2700	GO Process	GO:0019637	Organophosphate metabolic process
pncC	511145.b2700	GO Process	GO:0034641	Cellular nitrogen compound metabolic process
pncC	511145.b2700	GO Process	GO:0034654	Nucleobase-containing compound biosynthetic process
pncC	511145.b2700	GO Process	GO:0044237	Cellular metabolic process
pncC	511145.b2700	GO Process	GO:0044238	Primary metabolic process
pncC	511145.b2700	GO Process	GO:0044249	Cellular biosynthetic process
pncC	511145.b2700	GO Process	GO:0044271	Cellular nitrogen compound biosynthetic process
pncC	511145.b2700	GO Process	GO:0044281	Small molecule metabolic process
pncC	511145.b2700	GO Process	GO:0046483	Heterocycle metabolic process
pncC	511145.b2700	GO Process	GO:0055086	Nucleobase-containing small molecule metabolic process
pncC	511145.b2700	GO Process	GO:0071704	Organic substance metabolic process
pncC	511145.b2700	GO Process	GO:0072524	Pyridine-containing compound metabolic process
pncC	511145.b2700	GO Process	GO:0072525	Pyridine-containing compound biosynthetic process
pncC	511145.b2700	GO Process	GO:0090407	Organophosphate biosynthetic process
pncC	511145.b2700	GO Process	GO:1901293	Nucleoside phosphate biosynthetic process
pncC	511145.b2700	GO Process	GO:1901360	Organic cyclic compound metabolic process
pncC	511145.b2700	GO Process	GO:1901362	Organic cyclic compound biosynthetic process
pncC	511145.b2700	GO Process	GO:1901564	Organonitrogen compound metabolic process
pncC	511145.b2700	GO Process	GO:1901566	Organonitrogen compound biosynthetic process
pncC	511145.b2700	GO Process	GO:1901576	Organic substance biosynthetic process
pncC	511145.b2700	GO Function	GO:0003824	Catalytic activity
pncC	511145.b2700	GO Function	GO:0016787	Hydrolase activity
pncC	511145.b2700	GO Function	GO:0016810	Hydrolase activity, acting on carbon-nitrogen (but not peptide) bonds
pncC	511145.b2700	GO Function	GO:0016811	Hydrolase activity, acting on carbon-nitrogen (but not peptide) bonds, in linear amides
pncC	511145.b2700	GO Function	GO:0019159	Nicotinamide-nucleotide amidase activity
pncC	511145.b2700	STRING clusters	CL:1869	Water-soluble vitamin biosynthetic process, and Nicotinate and nicotinamide metabolism
pncC	511145.b2700	STRING clusters	CL:1974	Nicotinate and nicotinamide metabolism
pncC	511145.b2700	STRING clusters	CL:1976	Pyridine nucleotide salvage
pncC	511145.b2700	KEGG	eco00760	Nicotinate and nicotinamide metabolism
pncC	511145.b2700	UniProt Keywords	KW-0378	Hydrolase
pncC	511145.b2700	UniProt Keywords	KW-0662	Pyridine nucleotide biosynthesis
pncC	511145.b2700	Pfam	PF02464	Competence-damaged protein
pncC	511145.b2700	InterPro	IPR008136	CinA, C-terminal
pncC	511145.b2700	InterPro	IPR036653	CinA-like, C-terminal
pnp	511145.b3164	GO Process	GO:0006139	Nucleobase-containing compound metabolic process
pnp	511145.b3164	GO Process	GO:0006396	RNA processing
pnp	511145.b3164	GO Process	GO:0006401	RNA catabolic process
pnp	511145.b3164	GO Process	GO:0006402	mRNA catabolic process
pnp	511145.b3164	GO Process	GO:0006725	Cellular aromatic compound metabolic process
pnp	511145.b3164	GO Process	GO:0006807	Nitrogen compound metabolic process
pnp	511145.b3164	GO Process	GO:0006950	Response to stress
pnp	511145.b3164	GO Process	GO:0008152	Metabolic process
pnp	511145.b3164	GO Process	GO:0009056	Catabolic process
pnp	511145.b3164	GO Process	GO:0009057	Macromolecule catabolic process
pnp	511145.b3164	GO Process	GO:0009266	Response to temperature stimulus
pnp	511145.b3164	GO Process	GO:0009408	Response to heat
pnp	511145.b3164	GO Process	GO:0009628	Response to abiotic stimulus
pnp	511145.b3164	GO Process	GO:0009892	Negative regulation of metabolic process
pnp	511145.b3164	GO Process	GO:0009987	Cellular process
pnp	511145.b3164	GO Process	GO:0010467	Gene expression
pnp	511145.b3164	GO Process	GO:0010468	Regulation of gene expression
pnp	511145.b3164	GO Process	GO:0010605	Negative regulation of macromolecule metabolic process
pnp	511145.b3164	GO Process	GO:0010629	Negative regulation of gene expression
pnp	511145.b3164	GO Process	GO:0016070	RNA metabolic process
pnp	511145.b3164	GO Process	GO:0016071	mRNA metabolic process
pnp	511145.b3164	GO Process	GO:0019222	Regulation of metabolic process
pnp	511145.b3164	GO Process	GO:0019439	Aromatic compound catabolic process
pnp	511145.b3164	GO Process	GO:0034641	Cellular nitrogen compound metabolic process
pnp	511145.b3164	GO Process	GO:0034655	Nucleobase-containing compound catabolic process
pnp	511145.b3164	GO Process	GO:0043170	Macromolecule metabolic process
pnp	511145.b3164	GO Process	GO:0044237	Cellular metabolic process
pnp	511145.b3164	GO Process	GO:0044238	Primary metabolic process
pnp	511145.b3164	GO Process	GO:0044248	Cellular catabolic process
pnp	511145.b3164	GO Process	GO:0044260	Cellular macromolecule metabolic process
pnp	511145.b3164	GO Process	GO:0044265	Cellular macromolecule catabolic process
pnp	511145.b3164	GO Process	GO:0044270	Cellular nitrogen compound catabolic process
pnp	511145.b3164	GO Process	GO:0046483	Heterocycle metabolic process
pnp	511145.b3164	GO Process	GO:0046700	Heterocycle catabolic process
pnp	511145.b3164	GO Process	GO:0048519	Negative regulation of biological process
pnp	511145.b3164	GO Process	GO:0050789	Regulation of biological process
pnp	511145.b3164	GO Process	GO:0050896	Response to stimulus
pnp	511145.b3164	GO Process	GO:0060255	Regulation of macromolecule metabolic process
pnp	511145.b3164	GO Process	GO:0065007	Biological regulation
pnp	511145.b3164	GO Process	GO:0071704	Organic substance metabolic process
pnp	511145.b3164	GO Process	GO:0090304	Nucleic acid metabolic process
pnp	511145.b3164	GO Process	GO:0090305	Nucleic acid phosphodiester bond hydrolysis
pnp	511145.b3164	GO Process	GO:0090501	RNA phosphodiester bond hydrolysis
pnp	511145.b3164	GO Process	GO:0090503	RNA phosphodiester bond hydrolysis, exonucleolytic
pnp	511145.b3164	GO Process	GO:1901360	Organic cyclic compound metabolic process
pnp	511145.b3164	GO Process	GO:1901361	Organic cyclic compound catabolic process
pnp	511145.b3164	GO Process	GO:1901575	Organic substance catabolic process
pnp	511145.b3164	GO Function	GO:0000166	Nucleotide binding
pnp	511145.b3164	GO Function	GO:0000175	3-5-exoribonuclease activity
pnp	511145.b3164	GO Function	GO:0000287	Magnesium ion binding
pnp	511145.b3164	GO Function	GO:0003676	Nucleic acid binding
pnp	511145.b3164	GO Function	GO:0003723	RNA binding
pnp	511145.b3164	GO Function	GO:0003824	Catalytic activity
pnp	511145.b3164	GO Function	GO:0004518	Nuclease activity
pnp	511145.b3164	GO Function	GO:0004527	Exonuclease activity
pnp	511145.b3164	GO Function	GO:0004532	Exoribonuclease activity
pnp	511145.b3164	GO Function	GO:0004540	Ribonuclease activity
pnp	511145.b3164	GO Function	GO:0004654	Polyribonucleotide nucleotidyltransferase activity
pnp	511145.b3164	GO Function	GO:0005488	Binding
pnp	511145.b3164	GO Function	GO:0005515	Protein binding
pnp	511145.b3164	GO Function	GO:0008408	3-5 exonuclease activity
pnp	511145.b3164	GO Function	GO:0016740	Transferase activity
pnp	511145.b3164	GO Function	GO:0016772	Transferase activity, transferring phosphorus-containing groups
pnp	511145.b3164	GO Function	GO:0016779	Nucleotidyltransferase activity
pnp	511145.b3164	GO Function	GO:0016787	Hydrolase activity
pnp	511145.b3164	GO Function	GO:0016788	Hydrolase activity, acting on ester bonds
pnp	511145.b3164	GO Function	GO:0016796	Exonuclease activity, active with either ribo- or deoxyribonucleic acids and producing 5-phosphomonoesters
pnp	511145.b3164	GO Function	GO:0016896	Exoribonuclease activity, producing 5-phosphomonoesters
pnp	511145.b3164	GO Function	GO:0017076	Purine nucleotide binding
pnp	511145.b3164	GO Function	GO:0019001	Guanyl nucleotide binding
pnp	511145.b3164	GO Function	GO:0030551	Cyclic nucleotide binding
pnp	511145.b3164	GO Function	GO:0032553	Ribonucleotide binding
pnp	511145.b3164	GO Function	GO:0032555	Purine ribonucleotide binding
pnp	511145.b3164	GO Function	GO:0032561	Guanyl ribonucleotide binding
pnp	511145.b3164	GO Function	GO:0035438	cyclic-di-GMP binding
pnp	511145.b3164	GO Function	GO:0036094	Small molecule binding
pnp	511145.b3164	GO Function	GO:0042802	Identical protein binding
pnp	511145.b3164	GO Function	GO:0043167	Ion binding
pnp	511145.b3164	GO Function	GO:0043168	Anion binding
pnp	511145.b3164	GO Function	GO:0043169	Cation binding
pnp	511145.b3164	GO Function	GO:0046872	Metal ion binding
pnp	511145.b3164	GO Function	GO:0097159	Organic cyclic compound binding
pnp	511145.b3164	GO Function	GO:0097367	Carbohydrate derivative binding
pnp	511145.b3164	GO Function	GO:0140098	Catalytic activity, acting on RNA
pnp	511145.b3164	GO Function	GO:0140640	Catalytic activity, acting on a nucleic acid
pnp	511145.b3164	GO Function	GO:1901265	Nucleoside phosphate binding
pnp	511145.b3164	GO Function	GO:1901363	Heterocyclic compound binding
pnp	511145.b3164	GO Component	GO:0000177	Cytoplasmic exosome (RNase complex)
pnp	511145.b3164	GO Component	GO:0000178	Exosome (RNase complex)
pnp	511145.b3164	GO Component	GO:0005622	Intracellular anatomical structure
pnp	511145.b3164	GO Component	GO:0005737	Cytoplasm
pnp	511145.b3164	GO Component	GO:0005829	Cytosol
pnp	511145.b3164	GO Component	GO:0016020	Membrane
pnp	511145.b3164	GO Component	GO:0032991	Protein-containing complex
pnp	511145.b3164	GO Component	GO:0110165	Cellular anatomical entity
pnp	511145.b3164	GO Component	GO:1902494	Catalytic complex
pnp	511145.b3164	GO Component	GO:1905354	Exoribonuclease complex
pnp	511145.b3164	GO Component	GO:1990061	Bacterial degradosome
pnp	511145.b3164	STRING clusters	CL:531	Mixed, incl. RNA helicase activity, and Exoribonuclease activity
pnp	511145.b3164	STRING clusters	CL:533	Exoribonuclease activity, and RNA degradation
pnp	511145.b3164	STRING clusters	CL:534	RNA degradation, and Nuclease inhibitor activity
pnp	511145.b3164	STRING clusters	CL:538	Bacterial degradosome, and mRNA processing
pnp	511145.b3164	KEGG	eco03018	RNA degradation
pnp	511145.b3164	COMPARTMENTS	GOCC:0000177	Cytoplasmic exosome (RNase complex)
pnp	511145.b3164	COMPARTMENTS	GOCC:0000178	Exosome (RNase complex)
pnp	511145.b3164	COMPARTMENTS	GOCC:0005622	Intracellular
pnp	511145.b3164	COMPARTMENTS	GOCC:0005737	Cytoplasm
pnp	511145.b3164	COMPARTMENTS	GOCC:0005829	Cytosol
pnp	511145.b3164	COMPARTMENTS	GOCC:0032991	Protein-containing complex
pnp	511145.b3164	COMPARTMENTS	GOCC:0110165	Cellular anatomical entity
pnp	511145.b3164	COMPARTMENTS	GOCC:1902494	Catalytic complex
pnp	511145.b3164	COMPARTMENTS	GOCC:1905354	Exoribonuclease complex
pnp	511145.b3164	COMPARTMENTS	GOCC:1990061	Bacterial degradosome
pnp	511145.b3164	UniProt Keywords	KW-0346	Stress response
pnp	511145.b3164	UniProt Keywords	KW-0460	Magnesium
pnp	511145.b3164	UniProt Keywords	KW-0479	Metal-binding
pnp	511145.b3164	UniProt Keywords	KW-0548	Nucleotidyltransferase
pnp	511145.b3164	UniProt Keywords	KW-0694	RNA-binding
pnp	511145.b3164	UniProt Keywords	KW-0808	Transferase
pnp	511145.b3164	UniProt Keywords	KW-0963	Cytoplasm
pnp	511145.b3164	Pfam	PF01138	3 exoribonuclease family, domain 1
pnp	511145.b3164	Pfam	PF03725	3 exoribonuclease family, domain 2
pnp	511145.b3164	Pfam	PF03726	Polyribonucleotide nucleotidyltransferase, RNA binding domain
pnp	511145.b3164	InterPro	IPR001247	Exoribonuclease, phosphorolytic domain 1
pnp	511145.b3164	InterPro	IPR003029	S1 domain
pnp	511145.b3164	InterPro	IPR004087	K Homology domain
pnp	511145.b3164	InterPro	IPR004088	K Homology domain, type 1
pnp	511145.b3164	InterPro	IPR012162	Polyribonucleotide nucleotidyltransferase
pnp	511145.b3164	InterPro	IPR012340	Nucleic acid-binding, OB-fold
pnp	511145.b3164	InterPro	IPR015847	Exoribonuclease, phosphorolytic domain 2
pnp	511145.b3164	InterPro	IPR015848	Polyribonucleotide nucleotidyltransferase, RNA-binding domain
pnp	511145.b3164	InterPro	IPR020568	Ribosomal protein S5 domain 2-type fold
pnp	511145.b3164	InterPro	IPR022967	RNA-binding domain, S1
pnp	511145.b3164	InterPro	IPR027408	PNPase/RNase PH domain superfamily
pnp	511145.b3164	InterPro	IPR036345	Exoribonuclease, PH domain 2 superfamily
pnp	511145.b3164	InterPro	IPR036456	Polyribonucleotide nucleotidyltransferase, RNA-binding domain superfamily
pnp	511145.b3164	InterPro	IPR036612	K Homology domain, type 1 superfamily
pnp	511145.b3164	SMART	SM00316	Ribosomal protein S1-like RNA-binding domain
pnp	511145.b3164	SMART	SM00322	K homology RNA-binding domain
ppa	511145.b4226	GO Process	GO:0006793	Phosphorus metabolic process
ppa	511145.b4226	GO Process	GO:0006796	Phosphate-containing compound metabolic process
ppa	511145.b4226	GO Process	GO:0008152	Metabolic process
ppa	511145.b4226	GO Process	GO:0009987	Cellular process
ppa	511145.b4226	GO Process	GO:0044237	Cellular metabolic process
ppa	511145.b4226	GO Function	GO:0000287	Magnesium ion binding
ppa	511145.b4226	GO Function	GO:0003824	Catalytic activity
ppa	511145.b4226	GO Function	GO:0004427	Inorganic diphosphatase activity
ppa	511145.b4226	GO Function	GO:0005488	Binding
ppa	511145.b4226	GO Function	GO:0008270	Zinc ion binding
ppa	511145.b4226	GO Function	GO:0016462	Pyrophosphatase activity
ppa	511145.b4226	GO Function	GO:0016787	Hydrolase activity
ppa	511145.b4226	GO Function	GO:0016817	Hydrolase activity, acting on acid anhydrides
ppa	511145.b4226	GO Function	GO:0016818	Hydrolase activity, acting on acid anhydrides, in phosphorus-containing anhydrides
ppa	511145.b4226	GO Function	GO:0043167	Ion binding
ppa	511145.b4226	GO Function	GO:0043169	Cation binding
ppa	511145.b4226	GO Function	GO:0046872	Metal ion binding
ppa	511145.b4226	GO Function	GO:0046914	Transition metal ion binding
ppa	511145.b4226	GO Function	GO:0050355	Triphosphatase activity
ppa	511145.b4226	GO Component	GO:0005622	Intracellular anatomical structure
ppa	511145.b4226	GO Component	GO:0005737	Cytoplasm
ppa	511145.b4226	GO Component	GO:0005829	Cytosol
ppa	511145.b4226	GO Component	GO:0016020	Membrane
ppa	511145.b4226	GO Component	GO:0110165	Cellular anatomical entity
ppa	511145.b4226	STRING clusters	CL:35	Translation, and Protein export
ppa	511145.b4226	STRING clusters	CL:37	Translation, and Protein export
ppa	511145.b4226	STRING clusters	CL:39	Mixed, incl. Ribonucleoprotein, and Translation regulator activity
ppa	511145.b4226	STRING clusters	CL:40	Ribonucleoprotein, and Translation factor activity, RNA binding
ppa	511145.b4226	STRING clusters	CL:41	Ribonucleoprotein, and Translation factor activity, RNA binding
ppa	511145.b4226	STRING clusters	CL:42	Ribosome, and Translation factor activity, RNA binding
ppa	511145.b4226	KEGG	eco00190	Oxidative phosphorylation
ppa	511145.b4226	COMPARTMENTS	GOCC:0005622	Intracellular
ppa	511145.b4226	COMPARTMENTS	GOCC:0005737	Cytoplasm
ppa	511145.b4226	COMPARTMENTS	GOCC:0005829	Cytosol
ppa	511145.b4226	COMPARTMENTS	GOCC:0110165	Cellular anatomical entity
ppa	511145.b4226	UniProt Keywords	KW-0378	Hydrolase
ppa	511145.b4226	UniProt Keywords	KW-0460	Magnesium
ppa	511145.b4226	UniProt Keywords	KW-0479	Metal-binding
ppa	511145.b4226	UniProt Keywords	KW-0963	Cytoplasm
ppa	511145.b4226	Pfam	PF00719	Inorganic pyrophosphatase
ppa	511145.b4226	InterPro	IPR008162	Inorganic pyrophosphatase
ppa	511145.b4226	InterPro	IPR036649	Inorganic pyrophosphatase superfamily
prmA	511145.b3259	GO Process	GO:0006479	Protein methylation
prmA	511145.b3259	GO Process	GO:0006480	N-terminal protein amino acid methylation
prmA	511145.b3259	GO Process	GO:0006807	Nitrogen compound metabolic process
prmA	511145.b3259	GO Process	GO:0008152	Metabolic process
prmA	511145.b3259	GO Process	GO:0008213	Protein alkylation
prmA	511145.b3259	GO Process	GO:0009987	Cellular process
prmA	511145.b3259	GO Process	GO:0018011	N-terminal peptidyl-alanine methylation
prmA	511145.b3259	GO Process	GO:0018012	N-terminal peptidyl-alanine trimethylation
prmA	511145.b3259	GO Process	GO:0018022	Peptidyl-lysine methylation
prmA	511145.b3259	GO Process	GO:0018023	Peptidyl-lysine trimethylation
prmA	511145.b3259	GO Process	GO:0018193	Peptidyl-amino acid modification
prmA	511145.b3259	GO Process	GO:0018194	Peptidyl-alanine modification
prmA	511145.b3259	GO Process	GO:0018205	Peptidyl-lysine modification
prmA	511145.b3259	GO Process	GO:0019538	Protein metabolic process
prmA	511145.b3259	GO Process	GO:0031365	N-terminal protein amino acid modification
prmA	511145.b3259	GO Process	GO:0032259	Methylation
prmA	511145.b3259	GO Process	GO:0036211	Protein modification process
prmA	511145.b3259	GO Process	GO:0043170	Macromolecule metabolic process
prmA	511145.b3259	GO Process	GO:0043412	Macromolecule modification
prmA	511145.b3259	GO Process	GO:0043414	Macromolecule methylation
prmA	511145.b3259	GO Process	GO:0044237	Cellular metabolic process
prmA	511145.b3259	GO Process	GO:0044238	Primary metabolic process
prmA	511145.b3259	GO Process	GO:0044260	Cellular macromolecule metabolic process
prmA	511145.b3259	GO Process	GO:0071704	Organic substance metabolic process
prmA	511145.b3259	GO Process	GO:1901564	Organonitrogen compound metabolic process
prmA	511145.b3259	GO Function	GO:0003824	Catalytic activity
prmA	511145.b3259	GO Function	GO:0008168	Methyltransferase activity
prmA	511145.b3259	GO Function	GO:0008170	N-methyltransferase activity
prmA	511145.b3259	GO Function	GO:0008276	Protein methyltransferase activity
prmA	511145.b3259	GO Function	GO:0008757	S-adenosylmethionine-dependent methyltransferase activity
prmA	511145.b3259	GO Function	GO:0016278	Lysine N-methyltransferase activity
prmA	511145.b3259	GO Function	GO:0016279	Protein-lysine N-methyltransferase activity
prmA	511145.b3259	GO Function	GO:0016740	Transferase activity
prmA	511145.b3259	GO Function	GO:0016741	Transferase activity, transferring one-carbon groups
prmA	511145.b3259	GO Function	GO:0071885	N-terminal protein N-methyltransferase activity
prmA	511145.b3259	GO Function	GO:0140096	Catalytic activity, acting on a protein
prmA	511145.b3259	GO Component	GO:0005622	Intracellular anatomical structure
prmA	511145.b3259	GO Component	GO:0005737	Cytoplasm
prmA	511145.b3259	GO Component	GO:0005829	Cytosol
prmA	511145.b3259	GO Component	GO:0110165	Cellular anatomical entity
prmA	511145.b3259	STRING clusters	CL:1208	Mostly uncharacterized, incl. N-methyltransferase activity, and Invertasome
prmA	511145.b3259	STRING clusters	CL:1211	Mixed, incl. Invertasome, and Sodium ion export across plasma membrane
prmA	511145.b3259	COMPARTMENTS	GOCC:0005622	Intracellular
prmA	511145.b3259	COMPARTMENTS	GOCC:0005737	Cytoplasm
prmA	511145.b3259	COMPARTMENTS	GOCC:0005829	Cytosol
prmA	511145.b3259	COMPARTMENTS	GOCC:0110165	Cellular anatomical entity
prmA	511145.b3259	UniProt Keywords	KW-0489	Methyltransferase
prmA	511145.b3259	UniProt Keywords	KW-0808	Transferase
prmA	511145.b3259	UniProt Keywords	KW-0963	Cytoplasm
prmA	511145.b3259	Pfam	PF05175	Methyltransferase small domain
prmA	511145.b3259	InterPro	IPR004498	Ribosomal protein L11 methyltransferase
prmA	511145.b3259	InterPro	IPR029063	S-adenosyl-L-methionine-dependent methyltransferase superfamily
prmB	511145.b2330	GO Process	GO:0006139	Nucleobase-containing compound metabolic process
prmB	511145.b2330	GO Process	GO:0006259	DNA metabolic process
prmB	511145.b2330	GO Process	GO:0006304	DNA modification
prmB	511145.b2330	GO Process	GO:0006305	DNA alkylation
prmB	511145.b2330	GO Process	GO:0006306	DNA methylation
prmB	511145.b2330	GO Process	GO:0006479	Protein methylation
prmB	511145.b2330	GO Process	GO:0006725	Cellular aromatic compound metabolic process
prmB	511145.b2330	GO Process	GO:0006807	Nitrogen compound metabolic process
prmB	511145.b2330	GO Process	GO:0008152	Metabolic process
prmB	511145.b2330	GO Process	GO:0008213	Protein alkylation
prmB	511145.b2330	GO Process	GO:0009987	Cellular process
prmB	511145.b2330	GO Process	GO:0018364	Peptidyl-glutamine methylation
prmB	511145.b2330	GO Process	GO:0019538	Protein metabolic process
prmB	511145.b2330	GO Process	GO:0032259	Methylation
prmB	511145.b2330	GO Process	GO:0032775	DNA methylation on adenine
prmB	511145.b2330	GO Process	GO:0034641	Cellular nitrogen compound metabolic process
prmB	511145.b2330	GO Process	GO:0036211	Protein modification process
prmB	511145.b2330	GO Process	GO:0043170	Macromolecule metabolic process
prmB	511145.b2330	GO Process	GO:0043412	Macromolecule modification
prmB	511145.b2330	GO Process	GO:0043414	Macromolecule methylation
prmB	511145.b2330	GO Process	GO:0044237	Cellular metabolic process
prmB	511145.b2330	GO Process	GO:0044238	Primary metabolic process
prmB	511145.b2330	GO Process	GO:0044260	Cellular macromolecule metabolic process
prmB	511145.b2330	GO Process	GO:0044728	DNA methylation or demethylation
prmB	511145.b2330	GO Process	GO:0046483	Heterocycle metabolic process
prmB	511145.b2330	GO Process	GO:0071704	Organic substance metabolic process
prmB	511145.b2330	GO Process	GO:0090304	Nucleic acid metabolic process
prmB	511145.b2330	GO Process	GO:1901360	Organic cyclic compound metabolic process
prmB	511145.b2330	GO Process	GO:1901564	Organonitrogen compound metabolic process
prmB	511145.b2330	GO Function	GO:0003676	Nucleic acid binding
prmB	511145.b2330	GO Function	GO:0003824	Catalytic activity
prmB	511145.b2330	GO Function	GO:0005488	Binding
prmB	511145.b2330	GO Function	GO:0008168	Methyltransferase activity
prmB	511145.b2330	GO Function	GO:0008170	N-methyltransferase activity
prmB	511145.b2330	GO Function	GO:0008276	Protein methyltransferase activity
prmB	511145.b2330	GO Function	GO:0008757	S-adenosylmethionine-dependent methyltransferase activity
prmB	511145.b2330	GO Function	GO:0009007	Site-specific DNA-methyltransferase (adenine-specific) activity
prmB	511145.b2330	GO Function	GO:0009008	DNA-methyltransferase activity
prmB	511145.b2330	GO Function	GO:0016740	Transferase activity
prmB	511145.b2330	GO Function	GO:0016741	Transferase activity, transferring one-carbon groups
prmB	511145.b2330	GO Function	GO:0036009	Protein-glutamine N-methyltransferase activity
prmB	511145.b2330	GO Function	GO:0097159	Organic cyclic compound binding
prmB	511145.b2330	GO Function	GO:0140096	Catalytic activity, acting on a protein
prmB	511145.b2330	GO Function	GO:0140097	Catalytic activity, acting on DNA
prmB	511145.b2330	GO Function	GO:0140640	Catalytic activity, acting on a nucleic acid
prmB	511145.b2330	GO Function	GO:1901363	Heterocyclic compound binding
prmB	511145.b2330	GO Component	GO:0005622	Intracellular anatomical structure
prmB	511145.b2330	GO Component	GO:0005737	Cytoplasm
prmB	511145.b2330	GO Component	GO:0005829	Cytosol
prmB	511145.b2330	GO Component	GO:0110165	Cellular anatomical entity
prmB	511145.b2330	STRING clusters	CL:2689	Mixed, incl. Methyltransferase activity, and Condensin complex
prmB	511145.b2330	STRING clusters	CL:2745	Mixed, incl. Smr domain, and peptidyl-L-beta-methylthioaspartic acid biosynthetic process from peptidyl-aspartic acid
prmB	511145.b2330	STRING clusters	CL:2768	Mixed, incl. Met-10+ like-protein, and Bacterial-type EF-P lysine modification
prmB	511145.b2330	COMPARTMENTS	GOCC:0005622	Intracellular
prmB	511145.b2330	COMPARTMENTS	GOCC:0005737	Cytoplasm
prmB	511145.b2330	COMPARTMENTS	GOCC:0005829	Cytosol
prmB	511145.b2330	COMPARTMENTS	GOCC:0110165	Cellular anatomical entity
prmB	511145.b2330	UniProt Keywords	KW-0489	Methyltransferase
prmB	511145.b2330	UniProt Keywords	KW-0808	Transferase
prmB	511145.b2330	UniProt Keywords	KW-0949	S-adenosyl-L-methionine
prmB	511145.b2330	Pfam	PF02475	Met-10+ like-protein
prmB	511145.b2330	InterPro	IPR002052	DNA methylase, N-6 adenine-specific, conserved site
prmB	511145.b2330	InterPro	IPR004556	Methyltransferase HemK-like
prmB	511145.b2330	InterPro	IPR007848	Methyltransferase small domain
prmB	511145.b2330	InterPro	IPR017127	Ribosomal protein L3-specific, glutamine-N5-methyltransferase
prmB	511145.b2330	InterPro	IPR029063	S-adenosyl-L-methionine-dependent methyltransferase superfamily
prmC	511145.b1212	GO Process	GO:0006412	Translation
prmC	511145.b1212	GO Process	GO:0006415	Translational termination
prmC	511145.b1212	GO Process	GO:0006479	Protein methylation
prmC	511145.b1212	GO Process	GO:0006518	Peptide metabolic process
prmC	511145.b1212	GO Process	GO:0006807	Nitrogen compound metabolic process
prmC	511145.b1212	GO Process	GO:0008152	Metabolic process
prmC	511145.b1212	GO Process	GO:0008213	Protein alkylation
prmC	511145.b1212	GO Process	GO:0009058	Biosynthetic process
prmC	511145.b1212	GO Process	GO:0009059	Macromolecule biosynthetic process
prmC	511145.b1212	GO Process	GO:0009987	Cellular process
prmC	511145.b1212	GO Process	GO:0010467	Gene expression
prmC	511145.b1212	GO Process	GO:0010468	Regulation of gene expression
prmC	511145.b1212	GO Process	GO:0016043	Cellular component organization
prmC	511145.b1212	GO Process	GO:0018364	Peptidyl-glutamine methylation
prmC	511145.b1212	GO Process	GO:0019222	Regulation of metabolic process
prmC	511145.b1212	GO Process	GO:0019538	Protein metabolic process
prmC	511145.b1212	GO Process	GO:0022411	Cellular component disassembly
prmC	511145.b1212	GO Process	GO:0032259	Methylation
prmC	511145.b1212	GO Process	GO:0032984	Protein-containing complex disassembly
prmC	511145.b1212	GO Process	GO:0034641	Cellular nitrogen compound metabolic process
prmC	511145.b1212	GO Process	GO:0034645	Cellular macromolecule biosynthetic process
prmC	511145.b1212	GO Process	GO:0036211	Protein modification process
prmC	511145.b1212	GO Process	GO:0043043	Peptide biosynthetic process
prmC	511145.b1212	GO Process	GO:0043170	Macromolecule metabolic process
prmC	511145.b1212	GO Process	GO:0043412	Macromolecule modification
prmC	511145.b1212	GO Process	GO:0043414	Macromolecule methylation
prmC	511145.b1212	GO Process	GO:0043603	Cellular amide metabolic process
prmC	511145.b1212	GO Process	GO:0043604	Amide biosynthetic process
prmC	511145.b1212	GO Process	GO:0043933	Protein-containing complex organization
prmC	511145.b1212	GO Process	GO:0044237	Cellular metabolic process
prmC	511145.b1212	GO Process	GO:0044238	Primary metabolic process
prmC	511145.b1212	GO Process	GO:0044249	Cellular biosynthetic process
prmC	511145.b1212	GO Process	GO:0044260	Cellular macromolecule metabolic process
prmC	511145.b1212	GO Process	GO:0044271	Cellular nitrogen compound biosynthetic process
prmC	511145.b1212	GO Process	GO:0050789	Regulation of biological process
prmC	511145.b1212	GO Process	GO:0060255	Regulation of macromolecule metabolic process
prmC	511145.b1212	GO Process	GO:0065007	Biological regulation
prmC	511145.b1212	GO Process	GO:0071704	Organic substance metabolic process
prmC	511145.b1212	GO Process	GO:0071840	Cellular component organization or biogenesis
prmC	511145.b1212	GO Process	GO:1901564	Organonitrogen compound metabolic process
prmC	511145.b1212	GO Process	GO:1901566	Organonitrogen compound biosynthetic process
prmC	511145.b1212	GO Process	GO:1901576	Organic substance biosynthetic process
prmC	511145.b1212	GO Function	GO:0003676	Nucleic acid binding
prmC	511145.b1212	GO Function	GO:0003824	Catalytic activity
prmC	511145.b1212	GO Function	GO:0005488	Binding
prmC	511145.b1212	GO Function	GO:0008168	Methyltransferase activity
prmC	511145.b1212	GO Function	GO:0008170	N-methyltransferase activity
prmC	511145.b1212	GO Function	GO:0008276	Protein methyltransferase activity
prmC	511145.b1212	GO Function	GO:0008757	S-adenosylmethionine-dependent methyltransferase activity
prmC	511145.b1212	GO Function	GO:0016740	Transferase activity
prmC	511145.b1212	GO Function	GO:0016741	Transferase activity, transferring one-carbon groups
prmC	511145.b1212	GO Function	GO:0036009	Protein-glutamine N-methyltransferase activity
prmC	511145.b1212	GO Function	GO:0097159	Organic cyclic compound binding
prmC	511145.b1212	GO Function	GO:0102559	protein-(glutamine-N5) methyltransferase activity
prmC	511145.b1212	GO Function	GO:0140096	Catalytic activity, acting on a protein
prmC	511145.b1212	GO Function	GO:1901363	Heterocyclic compound binding
prmC	511145.b1212	STRING clusters	CL:35	Translation, and Protein export
prmC	511145.b1212	UniProt Keywords	KW-0489	Methyltransferase
prmC	511145.b1212	UniProt Keywords	KW-0808	Transferase
prmC	511145.b1212	UniProt Keywords	KW-0949	S-adenosyl-L-methionine
prmC	511145.b1212	Pfam	PF06325	Ribosomal protein L11 methyltransferase (PrmA)
prmC	511145.b1212	InterPro	IPR002052	DNA methylase, N-6 adenine-specific, conserved site
prmC	511145.b1212	InterPro	IPR004556	Methyltransferase HemK-like
prmC	511145.b1212	InterPro	IPR019874	Protein-(glutamine-N5) methyltransferase, release factor-specific
prmC	511145.b1212	InterPro	IPR025714	Methyltransferase domain
prmC	511145.b1212	InterPro	IPR029063	S-adenosyl-L-methionine-dependent methyltransferase superfamily
prmC	511145.b1212	InterPro	IPR040758	Release factor glutamine methyltransferase, N-terminal domain
puuC	511145.b1300	GO Process	GO:0006576	Cellular biogenic amine metabolic process
puuC	511145.b1300	GO Process	GO:0006595	Polyamine metabolic process
puuC	511145.b1300	GO Process	GO:0006598	Polyamine catabolic process
puuC	511145.b1300	GO Process	GO:0006807	Nitrogen compound metabolic process
puuC	511145.b1300	GO Process	GO:0008152	Metabolic process
puuC	511145.b1300	GO Process	GO:0009056	Catabolic process
puuC	511145.b1300	GO Process	GO:0009308	Amine metabolic process
puuC	511145.b1300	GO Process	GO:0009310	Amine catabolic process
puuC	511145.b1300	GO Process	GO:0009445	Putrescine metabolic process
puuC	511145.b1300	GO Process	GO:0009447	Putrescine catabolic process
puuC	511145.b1300	GO Process	GO:0009987	Cellular process
puuC	511145.b1300	GO Process	GO:0034641	Cellular nitrogen compound metabolic process
puuC	511145.b1300	GO Process	GO:0042402	Cellular biogenic amine catabolic process
puuC	511145.b1300	GO Process	GO:0044106	Cellular amine metabolic process
puuC	511145.b1300	GO Process	GO:0044237	Cellular metabolic process
puuC	511145.b1300	GO Process	GO:0071704	Organic substance metabolic process
puuC	511145.b1300	GO Process	GO:1901564	Organonitrogen compound metabolic process
puuC	511145.b1300	GO Process	GO:1901565	Organonitrogen compound catabolic process
puuC	511145.b1300	GO Process	GO:1901575	Organic substance catabolic process
puuC	511145.b1300	GO Function	GO:0003824	Catalytic activity
puuC	511145.b1300	GO Function	GO:0004029	Aldehyde dehydrogenase (NAD+) activity
puuC	511145.b1300	GO Function	GO:0004030	Aldehyde dehydrogenase [NAD(P)+] activity
puuC	511145.b1300	GO Function	GO:0016491	Oxidoreductase activity
puuC	511145.b1300	GO Function	GO:0016620	Oxidoreductase activity, acting on the aldehyde or oxo group of donors, NAD or NADP as acceptor
puuC	511145.b1300	GO Function	GO:0016903	Oxidoreductase activity, acting on the aldehyde or oxo group of donors
puuC	511145.b1300	GO Function	GO:0033721	Aldehyde dehydrogenase (NADP+) activity
puuC	511145.b1300	STRING clusters	CL:5950	Mixed, incl. Amine metabolic process, and Alcohol dehydrogenase-like, N-terminal
puuC	511145.b1300	STRING clusters	CL:6087	Mixed, incl. Polyamine metabolic process, and Arginine catabolic process to succinate
puuC	511145.b1300	STRING clusters	CL:6088	Polyamine metabolic process, and Arginine catabolic process to succinate
puuC	511145.b1300	STRING clusters	CL:6089	Polyamine metabolic process, and Gamma-aminobutyric acid catabolic process
puuC	511145.b1300	STRING clusters	CL:6090	Putrescine catabolic process, and Gamma-aminobutyric acid catabolic process
puuC	511145.b1300	STRING clusters	CL:6113	Putrescine catabolic process
puuC	511145.b1300	KEGG	eco00330	Arginine and proline metabolism
puuC	511145.b1300	KEGG	eco01100	Metabolic pathways
puuC	511145.b1300	UniProt Keywords	KW-0520	NAD
puuC	511145.b1300	UniProt Keywords	KW-0521	NADP
puuC	511145.b1300	UniProt Keywords	KW-0560	Oxidoreductase
puuC	511145.b1300	Pfam	PF00171	Aldehyde dehydrogenase family
puuC	511145.b1300	InterPro	IPR015590	Aldehyde dehydrogenase domain
puuC	511145.b1300	InterPro	IPR016160	Aldehyde dehydrogenase, cysteine active site
puuC	511145.b1300	InterPro	IPR016161	Aldehyde/histidinol dehydrogenase
puuC	511145.b1300	InterPro	IPR016162	Aldehyde dehydrogenase, N-terminal
puuC	511145.b1300	InterPro	IPR016163	Aldehyde dehydrogenase, C-terminal
puuC	511145.b1300	InterPro	IPR029510	Aldehyde dehydrogenase, glutamic acid active site
rdgC	511145.b0393	GO Process	GO:0000018	Regulation of DNA recombination
rdgC	511145.b0393	GO Process	GO:0006139	Nucleobase-containing compound metabolic process
rdgC	511145.b0393	GO Process	GO:0006259	DNA metabolic process
rdgC	511145.b0393	GO Process	GO:0006310	DNA recombination
rdgC	511145.b0393	GO Process	GO:0006725	Cellular aromatic compound metabolic process
rdgC	511145.b0393	GO Process	GO:0006807	Nitrogen compound metabolic process
rdgC	511145.b0393	GO Process	GO:0008152	Metabolic process
rdgC	511145.b0393	GO Process	GO:0009987	Cellular process
rdgC	511145.b0393	GO Process	GO:0019219	Regulation of nucleobase-containing compound metabolic process
rdgC	511145.b0393	GO Process	GO:0019222	Regulation of metabolic process
rdgC	511145.b0393	GO Process	GO:0031323	Regulation of cellular metabolic process
rdgC	511145.b0393	GO Process	GO:0034641	Cellular nitrogen compound metabolic process
rdgC	511145.b0393	GO Process	GO:0043170	Macromolecule metabolic process
rdgC	511145.b0393	GO Process	GO:0044237	Cellular metabolic process
rdgC	511145.b0393	GO Process	GO:0044238	Primary metabolic process
rdgC	511145.b0393	GO Process	GO:0044260	Cellular macromolecule metabolic process
rdgC	511145.b0393	GO Process	GO:0046483	Heterocycle metabolic process
rdgC	511145.b0393	GO Process	GO:0050789	Regulation of biological process
rdgC	511145.b0393	GO Process	GO:0050794	Regulation of cellular process
rdgC	511145.b0393	GO Process	GO:0051052	Regulation of DNA metabolic process
rdgC	511145.b0393	GO Process	GO:0051171	Regulation of nitrogen compound metabolic process
rdgC	511145.b0393	GO Process	GO:0060255	Regulation of macromolecule metabolic process
rdgC	511145.b0393	GO Process	GO:0065007	Biological regulation
rdgC	511145.b0393	GO Process	GO:0071704	Organic substance metabolic process
rdgC	511145.b0393	GO Process	GO:0080090	Regulation of primary metabolic process
rdgC	511145.b0393	GO Process	GO:0090304	Nucleic acid metabolic process
rdgC	511145.b0393	GO Process	GO:1901360	Organic cyclic compound metabolic process
rdgC	511145.b0393	GO Function	GO:0003676	Nucleic acid binding
rdgC	511145.b0393	GO Function	GO:0003677	DNA binding
rdgC	511145.b0393	GO Function	GO:0003690	Double-stranded DNA binding
rdgC	511145.b0393	GO Function	GO:0003697	Single-stranded DNA binding
rdgC	511145.b0393	GO Function	GO:0005488	Binding
rdgC	511145.b0393	GO Function	GO:0097159	Organic cyclic compound binding
rdgC	511145.b0393	GO Function	GO:1901363	Heterocyclic compound binding
rdgC	511145.b0393	GO Component	GO:0005622	Intracellular anatomical structure
rdgC	511145.b0393	GO Component	GO:0005737	Cytoplasm
rdgC	511145.b0393	GO Component	GO:0005829	Cytosol
rdgC	511145.b0393	GO Component	GO:0009295	Nucleoid
rdgC	511145.b0393	GO Component	GO:0043226	Organelle
rdgC	511145.b0393	GO Component	GO:0043228	Non-membrane-bounded organelle
rdgC	511145.b0393	GO Component	GO:0043229	Intracellular organelle
rdgC	511145.b0393	GO Component	GO:0043232	Intracellular non-membrane-bounded organelle
rdgC	511145.b0393	GO Component	GO:0043590	Bacterial nucleoid
rdgC	511145.b0393	GO Component	GO:0110165	Cellular anatomical entity
rdgC	511145.b0393	STRING clusters	CL:689	DNA repair, and Chromosome
rdgC	511145.b0393	STRING clusters	CL:690	DNA repair, and Chromosome
rdgC	511145.b0393	STRING clusters	CL:691	DNA repair, and Chromosome
rdgC	511145.b0393	COMPARTMENTS	GOCC:0005622	Intracellular
rdgC	511145.b0393	COMPARTMENTS	GOCC:0005737	Cytoplasm
rdgC	511145.b0393	COMPARTMENTS	GOCC:0005829	Cytosol
rdgC	511145.b0393	COMPARTMENTS	GOCC:0009295	Nucleoid
rdgC	511145.b0393	COMPARTMENTS	GOCC:0043226	Organelle
rdgC	511145.b0393	COMPARTMENTS	GOCC:0043228	Non-membrane-bounded organelle
rdgC	511145.b0393	COMPARTMENTS	GOCC:0043229	Intracellular organelle
rdgC	511145.b0393	COMPARTMENTS	GOCC:0043232	Intracellular non-membrane-bounded organelle
rdgC	511145.b0393	COMPARTMENTS	GOCC:0043590	Bacterial nucleoid
rdgC	511145.b0393	COMPARTMENTS	GOCC:0110165	Cellular anatomical entity
rdgC	511145.b0393	UniProt Keywords	KW-0233	DNA recombination
rdgC	511145.b0393	UniProt Keywords	KW-0963	Cytoplasm
rdgC	511145.b0393	Pfam	PF04381	Putative exonuclease, RdgC
rdgC	511145.b0393	InterPro	IPR007476	Putative exonuclease, RdgC
recO	511145.b2565	GO Process	GO:0006139	Nucleobase-containing compound metabolic process
recO	511145.b2565	GO Process	GO:0006259	DNA metabolic process
recO	511145.b2565	GO Process	GO:0006281	DNA repair
recO	511145.b2565	GO Process	GO:0006302	Double-strand break repair
recO	511145.b2565	GO Process	GO:0006310	DNA recombination
recO	511145.b2565	GO Process	GO:0006725	Cellular aromatic compound metabolic process
recO	511145.b2565	GO Process	GO:0006807	Nitrogen compound metabolic process
recO	511145.b2565	GO Process	GO:0006950	Response to stress
recO	511145.b2565	GO Process	GO:0006974	Cellular response to DNA damage stimulus
recO	511145.b2565	GO Process	GO:0008152	Metabolic process
recO	511145.b2565	GO Process	GO:0009314	Response to radiation
recO	511145.b2565	GO Process	GO:0009628	Response to abiotic stimulus
recO	511145.b2565	GO Process	GO:0009987	Cellular process
recO	511145.b2565	GO Process	GO:0033554	Cellular response to stress
recO	511145.b2565	GO Process	GO:0034641	Cellular nitrogen compound metabolic process
recO	511145.b2565	GO Process	GO:0043170	Macromolecule metabolic process
recO	511145.b2565	GO Process	GO:0044237	Cellular metabolic process
recO	511145.b2565	GO Process	GO:0044238	Primary metabolic process
recO	511145.b2565	GO Process	GO:0044260	Cellular macromolecule metabolic process
recO	511145.b2565	GO Process	GO:0046483	Heterocycle metabolic process
recO	511145.b2565	GO Process	GO:0050896	Response to stimulus
recO	511145.b2565	GO Process	GO:0051716	Cellular response to stimulus
recO	511145.b2565	GO Process	GO:0071704	Organic substance metabolic process
recO	511145.b2565	GO Process	GO:0090304	Nucleic acid metabolic process
recO	511145.b2565	GO Process	GO:1901360	Organic cyclic compound metabolic process
recO	511145.b2565	GO Component	GO:0005622	Intracellular anatomical structure
recO	511145.b2565	GO Component	GO:0009295	Nucleoid
recO	511145.b2565	GO Component	GO:0043226	Organelle
recO	511145.b2565	GO Component	GO:0043228	Non-membrane-bounded organelle
recO	511145.b2565	GO Component	GO:0043229	Intracellular organelle
recO	511145.b2565	GO Component	GO:0043232	Intracellular non-membrane-bounded organelle
recO	511145.b2565	GO Component	GO:0043590	Bacterial nucleoid
recO	511145.b2565	GO Component	GO:0110165	Cellular anatomical entity
recO	511145.b2565	STRING clusters	CL:689	DNA repair, and Chromosome
recO	511145.b2565	STRING clusters	CL:690	DNA repair, and Chromosome
recO	511145.b2565	STRING clusters	CL:691	DNA repair, and Chromosome
recO	511145.b2565	STRING clusters	CL:693	DNA damage, and Exodeoxyribonuclease activity
recO	511145.b2565	STRING clusters	CL:695	SOS response, and Exodeoxyribonuclease activity
recO	511145.b2565	STRING clusters	CL:697	DNA repair complex, and DNA helicase complex
recO	511145.b2565	STRING clusters	CL:699	Endodeoxyribonuclease complex, and Single-stranded DNA-dependent ATP-dependent DNA helicase complex
recO	511145.b2565	STRING clusters	CL:713	Single-stranded DNA exodeoxyribonuclease activity, and HRDC domain
recO	511145.b2565	KEGG	eco03440	Homologous recombination
recO	511145.b2565	UniProt Keywords	KW-0227	DNA damage
recO	511145.b2565	UniProt Keywords	KW-0233	DNA recombination
recO	511145.b2565	UniProt Keywords	KW-0234	DNA repair
recO	511145.b2565	Pfam	PF02565	Recombination protein O C terminal
recO	511145.b2565	Pfam	PF11967	Recombination protein O N terminal
recO	511145.b2565	InterPro	IPR003717	Recombination protein O, RecO
recO	511145.b2565	InterPro	IPR012340	Nucleic acid-binding, OB-fold
recO	511145.b2565	InterPro	IPR022572	DNA replication/recombination mediator RecO, N-terminal
recO	511145.b2565	InterPro	IPR037278	ARFGAP/RecO-like zinc finger
recO	511145.b2565	InterPro	IPR042242	Recombination protein O, C-terminal
recR	511145.b0472	GO Process	GO:0000731	DNA synthesis involved in DNA repair
recR	511145.b0472	GO Process	GO:0006139	Nucleobase-containing compound metabolic process
recR	511145.b0472	GO Process	GO:0006259	DNA metabolic process
recR	511145.b0472	GO Process	GO:0006281	DNA repair
recR	511145.b0472	GO Process	GO:0006310	DNA recombination
recR	511145.b0472	GO Process	GO:0006725	Cellular aromatic compound metabolic process
recR	511145.b0472	GO Process	GO:0006807	Nitrogen compound metabolic process
recR	511145.b0472	GO Process	GO:0006950	Response to stress
recR	511145.b0472	GO Process	GO:0006974	Cellular response to DNA damage stimulus
recR	511145.b0472	GO Process	GO:0008152	Metabolic process
recR	511145.b0472	GO Process	GO:0009058	Biosynthetic process
recR	511145.b0472	GO Process	GO:0009059	Macromolecule biosynthetic process
recR	511145.b0472	GO Process	GO:0009314	Response to radiation
recR	511145.b0472	GO Process	GO:0009411	Response to UV
recR	511145.b0472	GO Process	GO:0009416	Response to light stimulus
recR	511145.b0472	GO Process	GO:0009628	Response to abiotic stimulus
recR	511145.b0472	GO Process	GO:0009987	Cellular process
recR	511145.b0472	GO Process	GO:0018130	Heterocycle biosynthetic process
recR	511145.b0472	GO Process	GO:0019438	Aromatic compound biosynthetic process
recR	511145.b0472	GO Process	GO:0033554	Cellular response to stress
recR	511145.b0472	GO Process	GO:0034641	Cellular nitrogen compound metabolic process
recR	511145.b0472	GO Process	GO:0034654	Nucleobase-containing compound biosynthetic process
recR	511145.b0472	GO Process	GO:0043170	Macromolecule metabolic process
recR	511145.b0472	GO Process	GO:0044237	Cellular metabolic process
recR	511145.b0472	GO Process	GO:0044238	Primary metabolic process
recR	511145.b0472	GO Process	GO:0044249	Cellular biosynthetic process
recR	511145.b0472	GO Process	GO:0044260	Cellular macromolecule metabolic process
recR	511145.b0472	GO Process	GO:0044271	Cellular nitrogen compound biosynthetic process
recR	511145.b0472	GO Process	GO:0046483	Heterocycle metabolic process
recR	511145.b0472	GO Process	GO:0050896	Response to stimulus
recR	511145.b0472	GO Process	GO:0051716	Cellular response to stimulus
recR	511145.b0472	GO Process	GO:0071704	Organic substance metabolic process
recR	511145.b0472	GO Process	GO:0071897	DNA biosynthetic process
recR	511145.b0472	GO Process	GO:0090304	Nucleic acid metabolic process
recR	511145.b0472	GO Process	GO:1901360	Organic cyclic compound metabolic process
recR	511145.b0472	GO Process	GO:1901362	Organic cyclic compound biosynthetic process
recR	511145.b0472	GO Process	GO:1901576	Organic substance biosynthetic process
recR	511145.b0472	GO Function	GO:0003676	Nucleic acid binding
recR	511145.b0472	GO Function	GO:0003677	DNA binding
recR	511145.b0472	GO Function	GO:0005488	Binding
recR	511145.b0472	GO Function	GO:0043167	Ion binding
recR	511145.b0472	GO Function	GO:0043169	Cation binding
recR	511145.b0472	GO Function	GO:0046872	Metal ion binding
recR	511145.b0472	GO Function	GO:0097159	Organic cyclic compound binding
recR	511145.b0472	GO Function	GO:1901363	Heterocyclic compound binding
recR	511145.b0472	STRING clusters	CL:689	DNA repair, and Chromosome
recR	511145.b0472	STRING clusters	CL:690	DNA repair, and Chromosome
recR	511145.b0472	STRING clusters	CL:691	DNA repair, and Chromosome
recR	511145.b0472	KEGG	eco03440	Homologous recombination
recR	511145.b0472	UniProt Keywords	KW-0227	DNA damage
recR	511145.b0472	UniProt Keywords	KW-0233	DNA recombination
recR	511145.b0472	UniProt Keywords	KW-0234	DNA repair
recR	511145.b0472	UniProt Keywords	KW-0479	Metal-binding
recR	511145.b0472	UniProt Keywords	KW-0862	Zinc
recR	511145.b0472	UniProt Keywords	KW-0863	Zinc-finger
recR	511145.b0472	Pfam	PF02132	RecR protein
recR	511145.b0472	InterPro	IPR000093	DNA recombination protein RecR
recR	511145.b0472	InterPro	IPR006171	TOPRIM domain
recR	511145.b0472	InterPro	IPR015967	Recombination protein RecR, conserved site
recR	511145.b0472	InterPro	IPR023627	Recombination protein RecR
recR	511145.b0472	InterPro	IPR023628	Recombination protein RecR, C4-type zinc finger
recR	511145.b0472	InterPro	IPR034137	RecR, TOPRIM domain
recR	511145.b0472	SMART	SM00278	Helix-hairpin-helix DNA-binding motif class 1
recR	511145.b0472	SMART	SM00493	Topoisomerases, DnaG-type primases, OLD family nucleases and RecR proteins
ribE	511145.b0415	GO Process	GO:0006766	Vitamin metabolic process
ribE	511145.b0415	GO Process	GO:0006767	Water-soluble vitamin metabolic process
ribE	511145.b0415	GO Process	GO:0006771	Riboflavin metabolic process
ribE	511145.b0415	GO Process	GO:0006807	Nitrogen compound metabolic process
ribE	511145.b0415	GO Process	GO:0008152	Metabolic process
ribE	511145.b0415	GO Process	GO:0009058	Biosynthetic process
ribE	511145.b0415	GO Process	GO:0009110	Vitamin biosynthetic process
ribE	511145.b0415	GO Process	GO:0009231	Riboflavin biosynthetic process
ribE	511145.b0415	GO Process	GO:0009987	Cellular process
ribE	511145.b0415	GO Process	GO:0018130	Heterocycle biosynthetic process
ribE	511145.b0415	GO Process	GO:0034641	Cellular nitrogen compound metabolic process
ribE	511145.b0415	GO Process	GO:0042364	Water-soluble vitamin biosynthetic process
ribE	511145.b0415	GO Process	GO:0042726	Flavin-containing compound metabolic process
ribE	511145.b0415	GO Process	GO:0042727	Flavin-containing compound biosynthetic process
ribE	511145.b0415	GO Process	GO:0044237	Cellular metabolic process
ribE	511145.b0415	GO Process	GO:0044249	Cellular biosynthetic process
ribE	511145.b0415	GO Process	GO:0044271	Cellular nitrogen compound biosynthetic process
ribE	511145.b0415	GO Process	GO:0044281	Small molecule metabolic process
ribE	511145.b0415	GO Process	GO:0044283	Small molecule biosynthetic process
ribE	511145.b0415	GO Process	GO:0046483	Heterocycle metabolic process
ribE	511145.b0415	GO Process	GO:0071704	Organic substance metabolic process
ribE	511145.b0415	GO Process	GO:1901360	Organic cyclic compound metabolic process
ribE	511145.b0415	GO Process	GO:1901362	Organic cyclic compound biosynthetic process
ribE	511145.b0415	GO Process	GO:1901564	Organonitrogen compound metabolic process
ribE	511145.b0415	GO Process	GO:1901566	Organonitrogen compound biosynthetic process
ribE	511145.b0415	GO Process	GO:1901576	Organic substance biosynthetic process
ribE	511145.b0415	GO Function	GO:0000906	6,7-dimethyl-8-ribityllumazine synthase activity
ribE	511145.b0415	GO Function	GO:0003824	Catalytic activity
ribE	511145.b0415	GO Function	GO:0016740	Transferase activity
ribE	511145.b0415	GO Function	GO:0016765	Transferase activity, transferring alkyl or aryl (other than methyl) groups
ribE	511145.b0415	GO Component	GO:0005622	Intracellular anatomical structure
ribE	511145.b0415	GO Component	GO:0005737	Cytoplasm
ribE	511145.b0415	GO Component	GO:0005829	Cytosol
ribE	511145.b0415	GO Component	GO:0009349	Riboflavin synthase complex
ribE	511145.b0415	GO Component	GO:0032991	Protein-containing complex
ribE	511145.b0415	GO Component	GO:0110165	Cellular anatomical entity
ribE	511145.b0415	GO Component	GO:1902494	Catalytic complex
ribE	511145.b0415	GO Component	GO:1990234	Transferase complex
ribE	511145.b0415	STRING clusters	CL:1869	Water-soluble vitamin biosynthetic process, and Nicotinate and nicotinamide metabolism
ribE	511145.b0415	STRING clusters	CL:1870	Water-soluble vitamin biosynthetic process, and Coenzyme A biosynthetic process
ribE	511145.b0415	STRING clusters	CL:1871	Water-soluble vitamin biosynthetic process
ribE	511145.b0415	STRING clusters	CL:1872	Water-soluble vitamin biosynthetic process
ribE	511145.b0415	STRING clusters	CL:1908	Riboflavin metabolism
ribE	511145.b0415	STRING clusters	CL:1910	Riboflavin biosynthesis
ribE	511145.b0415	KEGG	eco00740	Riboflavin metabolism
ribE	511145.b0415	KEGG	eco01100	Metabolic pathways
ribE	511145.b0415	KEGG	eco01110	Biosynthesis of secondary metabolites
ribE	511145.b0415	COMPARTMENTS	GOCC:0005622	Intracellular
ribE	511145.b0415	COMPARTMENTS	GOCC:0005737	Cytoplasm
ribE	511145.b0415	COMPARTMENTS	GOCC:0005829	Cytosol
ribE	511145.b0415	COMPARTMENTS	GOCC:0009349	Riboflavin synthase complex
ribE	511145.b0415	COMPARTMENTS	GOCC:0032991	Protein-containing complex
ribE	511145.b0415	COMPARTMENTS	GOCC:0110165	Cellular anatomical entity
ribE	511145.b0415	COMPARTMENTS	GOCC:1902494	Catalytic complex
ribE	511145.b0415	COMPARTMENTS	GOCC:1990234	Transferase complex
ribE	511145.b0415	UniProt Keywords	KW-0686	Riboflavin biosynthesis
ribE	511145.b0415	UniProt Keywords	KW-0808	Transferase
ribE	511145.b0415	Pfam	PF00885	6,7-dimethyl-8-ribityllumazine synthase
ribE	511145.b0415	InterPro	IPR002180	Lumazine/riboflavin synthase
ribE	511145.b0415	InterPro	IPR034964	Lumazine synthase
ribE	511145.b0415	InterPro	IPR036467	Lumazine/riboflavin synthase superfamily
rlmD	511145.b2785	GO Process	GO:0000154	rRNA modification
rlmD	511145.b2785	GO Process	GO:0001510	RNA methylation
rlmD	511145.b2785	GO Process	GO:0006139	Nucleobase-containing compound metabolic process
rlmD	511145.b2785	GO Process	GO:0006364	rRNA processing
rlmD	511145.b2785	GO Process	GO:0006396	RNA processing
rlmD	511145.b2785	GO Process	GO:0006725	Cellular aromatic compound metabolic process
rlmD	511145.b2785	GO Process	GO:0006807	Nitrogen compound metabolic process
rlmD	511145.b2785	GO Process	GO:0008152	Metabolic process
rlmD	511145.b2785	GO Process	GO:0009451	RNA modification
rlmD	511145.b2785	GO Process	GO:0009987	Cellular process
rlmD	511145.b2785	GO Process	GO:0010467	Gene expression
rlmD	511145.b2785	GO Process	GO:0016070	RNA metabolic process
rlmD	511145.b2785	GO Process	GO:0016072	rRNA metabolic process
rlmD	511145.b2785	GO Process	GO:0022613	Ribonucleoprotein complex biogenesis
rlmD	511145.b2785	GO Process	GO:0031167	rRNA methylation
rlmD	511145.b2785	GO Process	GO:0032259	Methylation
rlmD	511145.b2785	GO Process	GO:0034470	ncRNA processing
rlmD	511145.b2785	GO Process	GO:0034641	Cellular nitrogen compound metabolic process
rlmD	511145.b2785	GO Process	GO:0034660	ncRNA metabolic process
rlmD	511145.b2785	GO Process	GO:0042254	Ribosome biogenesis
rlmD	511145.b2785	GO Process	GO:0043170	Macromolecule metabolic process
rlmD	511145.b2785	GO Process	GO:0043412	Macromolecule modification
rlmD	511145.b2785	GO Process	GO:0043414	Macromolecule methylation
rlmD	511145.b2785	GO Process	GO:0044085	Cellular component biogenesis
rlmD	511145.b2785	GO Process	GO:0044237	Cellular metabolic process
rlmD	511145.b2785	GO Process	GO:0044238	Primary metabolic process
rlmD	511145.b2785	GO Process	GO:0044260	Cellular macromolecule metabolic process
rlmD	511145.b2785	GO Process	GO:0046483	Heterocycle metabolic process
rlmD	511145.b2785	GO Process	GO:0070475	rRNA base methylation
rlmD	511145.b2785	GO Process	GO:0071704	Organic substance metabolic process
rlmD	511145.b2785	GO Process	GO:0071840	Cellular component organization or biogenesis
rlmD	511145.b2785	GO Process	GO:0090304	Nucleic acid metabolic process
rlmD	511145.b2785	GO Process	GO:1901360	Organic cyclic compound metabolic process
rlmD	511145.b2785	GO Function	GO:0003676	Nucleic acid binding
rlmD	511145.b2785	GO Function	GO:0003723	RNA binding
rlmD	511145.b2785	GO Function	GO:0003824	Catalytic activity
rlmD	511145.b2785	GO Function	GO:0005488	Binding
rlmD	511145.b2785	GO Function	GO:0005506	Iron ion binding
rlmD	511145.b2785	GO Function	GO:0008168	Methyltransferase activity
rlmD	511145.b2785	GO Function	GO:0008169	C-methyltransferase activity
rlmD	511145.b2785	GO Function	GO:0008173	RNA methyltransferase activity
rlmD	511145.b2785	GO Function	GO:0008649	rRNA methyltransferase activity
rlmD	511145.b2785	GO Function	GO:0008757	S-adenosylmethionine-dependent methyltransferase activity
rlmD	511145.b2785	GO Function	GO:0016436	rRNA (uridine) methyltransferase activity
rlmD	511145.b2785	GO Function	GO:0016740	Transferase activity
rlmD	511145.b2785	GO Function	GO:0016741	Transferase activity, transferring one-carbon groups
rlmD	511145.b2785	GO Function	GO:0043167	Ion binding
rlmD	511145.b2785	GO Function	GO:0043169	Cation binding
rlmD	511145.b2785	GO Function	GO:0046872	Metal ion binding
rlmD	511145.b2785	GO Function	GO:0046914	Transition metal ion binding
rlmD	511145.b2785	GO Function	GO:0051536	Iron-sulfur cluster binding
rlmD	511145.b2785	GO Function	GO:0051539	4 iron, 4 sulfur cluster binding
rlmD	511145.b2785	GO Function	GO:0051540	Metal cluster binding
rlmD	511145.b2785	GO Function	GO:0070041	rRNA (uridine-C5-)-methyltransferase activity
rlmD	511145.b2785	GO Function	GO:0097159	Organic cyclic compound binding
rlmD	511145.b2785	GO Function	GO:0140098	Catalytic activity, acting on RNA
rlmD	511145.b2785	GO Function	GO:0140102	Catalytic activity, acting on a rRNA
rlmD	511145.b2785	GO Function	GO:0140640	Catalytic activity, acting on a nucleic acid
rlmD	511145.b2785	GO Function	GO:1901363	Heterocyclic compound binding
rlmD	511145.b2785	STRING clusters	CL:378	Mixed, incl. rRNA modification, and tRNA methylation
rlmD	511145.b2785	STRING clusters	CL:379	rRNA modification, and tRNA methylation
rlmD	511145.b2785	STRING clusters	CL:380	RNA methylation, and GTP binding domain
rlmD	511145.b2785	STRING clusters	CL:381	RNA methylation
rlmD	511145.b2785	STRING clusters	CL:382	RNA methyltransferase activity
rlmD	511145.b2785	STRING clusters	CL:394	rRNA (cytosine) methyltransferase activity, and rRNA (uridine) methyltransferase activity
rlmD	511145.b2785	UniProt Keywords	KW-0004	4Fe-4S
rlmD	511145.b2785	UniProt Keywords	KW-0408	Iron
rlmD	511145.b2785	UniProt Keywords	KW-0411	Iron-sulfur
rlmD	511145.b2785	UniProt Keywords	KW-0479	Metal-binding
rlmD	511145.b2785	UniProt Keywords	KW-0489	Methyltransferase
rlmD	511145.b2785	UniProt Keywords	KW-0698	rRNA processing
rlmD	511145.b2785	UniProt Keywords	KW-0808	Transferase
rlmD	511145.b2785	UniProt Keywords	KW-0949	S-adenosyl-L-methionine
rlmD	511145.b2785	Pfam	PF01135	Protein-L-isoaspartate(D-aspartate) O-methyltransferase (PCMT)
rlmD	511145.b2785	Pfam	PF01938	TRAM domain
rlmD	511145.b2785	InterPro	IPR001566	23S rRNA (uracil(1939)-C(5))-methyltransferase RlmD
rlmD	511145.b2785	InterPro	IPR002792	TRAM domain
rlmD	511145.b2785	InterPro	IPR010280	(Uracil-5)-methyltransferase family
rlmD	511145.b2785	InterPro	IPR012340	Nucleic acid-binding, OB-fold
rlmD	511145.b2785	InterPro	IPR029063	S-adenosyl-L-methionine-dependent methyltransferase superfamily
rlmD	511145.b2785	InterPro	IPR030390	RNA methyltransferase TrmA, active site
rlmD	511145.b2785	InterPro	IPR030391	RNA methyltransferase TrmA, conserved site
rlmI	511145.b0967	GO Process	GO:0000154	rRNA modification
rlmI	511145.b0967	GO Process	GO:0001510	RNA methylation
rlmI	511145.b0967	GO Process	GO:0006139	Nucleobase-containing compound metabolic process
rlmI	511145.b0967	GO Process	GO:0006364	rRNA processing
rlmI	511145.b0967	GO Process	GO:0006396	RNA processing
rlmI	511145.b0967	GO Process	GO:0006725	Cellular aromatic compound metabolic process
rlmI	511145.b0967	GO Process	GO:0006807	Nitrogen compound metabolic process
rlmI	511145.b0967	GO Process	GO:0008152	Metabolic process
rlmI	511145.b0967	GO Process	GO:0009451	RNA modification
rlmI	511145.b0967	GO Process	GO:0009987	Cellular process
rlmI	511145.b0967	GO Process	GO:0010467	Gene expression
rlmI	511145.b0967	GO Process	GO:0016070	RNA metabolic process
rlmI	511145.b0967	GO Process	GO:0016072	rRNA metabolic process
rlmI	511145.b0967	GO Process	GO:0022613	Ribonucleoprotein complex biogenesis
rlmI	511145.b0967	GO Process	GO:0031167	rRNA methylation
rlmI	511145.b0967	GO Process	GO:0032259	Methylation
rlmI	511145.b0967	GO Process	GO:0034470	ncRNA processing
rlmI	511145.b0967	GO Process	GO:0034641	Cellular nitrogen compound metabolic process
rlmI	511145.b0967	GO Process	GO:0034660	ncRNA metabolic process
rlmI	511145.b0967	GO Process	GO:0042254	Ribosome biogenesis
rlmI	511145.b0967	GO Process	GO:0042710	Biofilm formation
rlmI	511145.b0967	GO Process	GO:0043170	Macromolecule metabolic process
rlmI	511145.b0967	GO Process	GO:0043412	Macromolecule modification
rlmI	511145.b0967	GO Process	GO:0043414	Macromolecule methylation
rlmI	511145.b0967	GO Process	GO:0044010	Single-species biofilm formation
rlmI	511145.b0967	GO Process	GO:0044085	Cellular component biogenesis
rlmI	511145.b0967	GO Process	GO:0044237	Cellular metabolic process
rlmI	511145.b0967	GO Process	GO:0044238	Primary metabolic process
rlmI	511145.b0967	GO Process	GO:0044260	Cellular macromolecule metabolic process
rlmI	511145.b0967	GO Process	GO:0046483	Heterocycle metabolic process
rlmI	511145.b0967	GO Process	GO:0051703	Biological process involved in intraspecies interaction between organisms
rlmI	511145.b0967	GO Process	GO:0070475	rRNA base methylation
rlmI	511145.b0967	GO Process	GO:0071704	Organic substance metabolic process
rlmI	511145.b0967	GO Process	GO:0071840	Cellular component organization or biogenesis
rlmI	511145.b0967	GO Process	GO:0090304	Nucleic acid metabolic process
rlmI	511145.b0967	GO Process	GO:0098630	Aggregation of unicellular organisms
rlmI	511145.b0967	GO Process	GO:0098743	Cell aggregation
rlmI	511145.b0967	GO Process	GO:1901360	Organic cyclic compound metabolic process
rlmI	511145.b0967	GO Function	GO:0003676	Nucleic acid binding
rlmI	511145.b0967	GO Function	GO:0003723	RNA binding
rlmI	511145.b0967	GO Function	GO:0003824	Catalytic activity
rlmI	511145.b0967	GO Function	GO:0005488	Binding
rlmI	511145.b0967	GO Function	GO:0008168	Methyltransferase activity
rlmI	511145.b0967	GO Function	GO:0008169	C-methyltransferase activity
rlmI	511145.b0967	GO Function	GO:0008173	RNA methyltransferase activity
rlmI	511145.b0967	GO Function	GO:0008649	rRNA methyltransferase activity
rlmI	511145.b0967	GO Function	GO:0008757	S-adenosylmethionine-dependent methyltransferase activity
rlmI	511145.b0967	GO Function	GO:0009383	rRNA (cytosine-C5-)-methyltransferase activity
rlmI	511145.b0967	GO Function	GO:0016434	rRNA (cytosine) methyltransferase activity
rlmI	511145.b0967	GO Function	GO:0016740	Transferase activity
rlmI	511145.b0967	GO Function	GO:0016741	Transferase activity, transferring one-carbon groups
rlmI	511145.b0967	GO Function	GO:0097159	Organic cyclic compound binding
rlmI	511145.b0967	GO Function	GO:0140098	Catalytic activity, acting on RNA
rlmI	511145.b0967	GO Function	GO:0140102	Catalytic activity, acting on a rRNA
rlmI	511145.b0967	GO Function	GO:0140640	Catalytic activity, acting on a nucleic acid
rlmI	511145.b0967	GO Function	GO:1901363	Heterocyclic compound binding
rlmI	511145.b0967	GO Component	GO:0005622	Intracellular anatomical structure
rlmI	511145.b0967	GO Component	GO:0005737	Cytoplasm
rlmI	511145.b0967	GO Component	GO:0005829	Cytosol
rlmI	511145.b0967	GO Component	GO:0110165	Cellular anatomical entity
rlmI	511145.b0967	STRING clusters	CL:378	Mixed, incl. rRNA modification, and tRNA methylation
rlmI	511145.b0967	STRING clusters	CL:379	rRNA modification, and tRNA methylation
rlmI	511145.b0967	STRING clusters	CL:380	RNA methylation, and GTP binding domain
rlmI	511145.b0967	STRING clusters	CL:381	RNA methylation
rlmI	511145.b0967	STRING clusters	CL:382	RNA methyltransferase activity
rlmI	511145.b0967	STRING clusters	CL:394	rRNA (cytosine) methyltransferase activity, and rRNA (uridine) methyltransferase activity
rlmI	511145.b0967	COMPARTMENTS	GOCC:0005622	Intracellular
rlmI	511145.b0967	COMPARTMENTS	GOCC:0005737	Cytoplasm
rlmI	511145.b0967	COMPARTMENTS	GOCC:0005829	Cytosol
rlmI	511145.b0967	COMPARTMENTS	GOCC:0110165	Cellular anatomical entity
rlmI	511145.b0967	UniProt Keywords	KW-0489	Methyltransferase
rlmI	511145.b0967	UniProt Keywords	KW-0694	RNA-binding
rlmI	511145.b0967	UniProt Keywords	KW-0698	rRNA processing
rlmI	511145.b0967	UniProt Keywords	KW-0808	Transferase
rlmI	511145.b0967	UniProt Keywords	KW-0949	S-adenosyl-L-methionine
rlmI	511145.b0967	UniProt Keywords	KW-0963	Cytoplasm
rlmI	511145.b0967	Pfam	PF02475	Met-10+ like-protein
rlmI	511145.b0967	InterPro	IPR002478	PUA domain
rlmI	511145.b0967	InterPro	IPR015947	PUA-like superfamily
rlmI	511145.b0967	InterPro	IPR019614	S-adenosylmethionine-dependent methyltransferase
rlmI	511145.b0967	InterPro	IPR023542	Ribosomal RNA large subunit methyltransferase I
rlmI	511145.b0967	InterPro	IPR029063	S-adenosyl-L-methionine-dependent methyltransferase superfamily
rlmI	511145.b0967	InterPro	IPR036974	PUA domain superfamily
rlmI	511145.b0967	InterPro	IPR041532	RlmI-like, PUA domain
rlmI	511145.b0967	SMART	SM00359	Putative RNA-binding Domain in PseudoUridine synthase and Archaeosine transglycosylase
rnb	511145.b1286	GO Process	GO:0006139	Nucleobase-containing compound metabolic process
rnb	511145.b1286	GO Process	GO:0006401	RNA catabolic process
rnb	511145.b1286	GO Process	GO:0006402	mRNA catabolic process
rnb	511145.b1286	GO Process	GO:0006725	Cellular aromatic compound metabolic process
rnb	511145.b1286	GO Process	GO:0006807	Nitrogen compound metabolic process
rnb	511145.b1286	GO Process	GO:0008152	Metabolic process
rnb	511145.b1286	GO Process	GO:0009056	Catabolic process
rnb	511145.b1286	GO Process	GO:0009057	Macromolecule catabolic process
rnb	511145.b1286	GO Process	GO:0009892	Negative regulation of metabolic process
rnb	511145.b1286	GO Process	GO:0009987	Cellular process
rnb	511145.b1286	GO Process	GO:0010468	Regulation of gene expression
rnb	511145.b1286	GO Process	GO:0010605	Negative regulation of macromolecule metabolic process
rnb	511145.b1286	GO Process	GO:0010629	Negative regulation of gene expression
rnb	511145.b1286	GO Process	GO:0016070	RNA metabolic process
rnb	511145.b1286	GO Process	GO:0016071	mRNA metabolic process
rnb	511145.b1286	GO Process	GO:0019222	Regulation of metabolic process
rnb	511145.b1286	GO Process	GO:0019439	Aromatic compound catabolic process
rnb	511145.b1286	GO Process	GO:0034641	Cellular nitrogen compound metabolic process
rnb	511145.b1286	GO Process	GO:0034655	Nucleobase-containing compound catabolic process
rnb	511145.b1286	GO Process	GO:0043170	Macromolecule metabolic process
rnb	511145.b1286	GO Process	GO:0044237	Cellular metabolic process
rnb	511145.b1286	GO Process	GO:0044238	Primary metabolic process
rnb	511145.b1286	GO Process	GO:0044248	Cellular catabolic process
rnb	511145.b1286	GO Process	GO:0044260	Cellular macromolecule metabolic process
rnb	511145.b1286	GO Process	GO:0044265	Cellular macromolecule catabolic process
rnb	511145.b1286	GO Process	GO:0044270	Cellular nitrogen compound catabolic process
rnb	511145.b1286	GO Process	GO:0046483	Heterocycle metabolic process
rnb	511145.b1286	GO Process	GO:0046700	Heterocycle catabolic process
rnb	511145.b1286	GO Process	GO:0048519	Negative regulation of biological process
rnb	511145.b1286	GO Process	GO:0050789	Regulation of biological process
rnb	511145.b1286	GO Process	GO:0060255	Regulation of macromolecule metabolic process
rnb	511145.b1286	GO Process	GO:0065007	Biological regulation
rnb	511145.b1286	GO Process	GO:0071704	Organic substance metabolic process
rnb	511145.b1286	GO Process	GO:0090304	Nucleic acid metabolic process
rnb	511145.b1286	GO Process	GO:0090305	Nucleic acid phosphodiester bond hydrolysis
rnb	511145.b1286	GO Process	GO:0090501	RNA phosphodiester bond hydrolysis
rnb	511145.b1286	GO Process	GO:0090503	RNA phosphodiester bond hydrolysis, exonucleolytic
rnb	511145.b1286	GO Process	GO:1901360	Organic cyclic compound metabolic process
rnb	511145.b1286	GO Process	GO:1901361	Organic cyclic compound catabolic process
rnb	511145.b1286	GO Process	GO:1901575	Organic substance catabolic process
rnb	511145.b1286	GO Function	GO:0003676	Nucleic acid binding
rnb	511145.b1286	GO Function	GO:0003723	RNA binding
rnb	511145.b1286	GO Function	GO:0003824	Catalytic activity
rnb	511145.b1286	GO Function	GO:0004518	Nuclease activity
rnb	511145.b1286	GO Function	GO:0004527	Exonuclease activity
rnb	511145.b1286	GO Function	GO:0004532	Exoribonuclease activity
rnb	511145.b1286	GO Function	GO:0004540	Ribonuclease activity
rnb	511145.b1286	GO Function	GO:0005488	Binding
rnb	511145.b1286	GO Function	GO:0008408	3-5 exonuclease activity
rnb	511145.b1286	GO Function	GO:0008859	Exoribonuclease II activity
rnb	511145.b1286	GO Function	GO:0016787	Hydrolase activity
rnb	511145.b1286	GO Function	GO:0016788	Hydrolase activity, acting on ester bonds
rnb	511145.b1286	GO Function	GO:0016796	Exonuclease activity, active with either ribo- or deoxyribonucleic acids and producing 5-phosphomonoesters
rnb	511145.b1286	GO Function	GO:0016896	Exoribonuclease activity, producing 5-phosphomonoesters
rnb	511145.b1286	GO Function	GO:0097159	Organic cyclic compound binding
rnb	511145.b1286	GO Function	GO:0140098	Catalytic activity, acting on RNA
rnb	511145.b1286	GO Function	GO:0140640	Catalytic activity, acting on a nucleic acid
rnb	511145.b1286	GO Function	GO:1901363	Heterocyclic compound binding
rnb	511145.b1286	GO Component	GO:0005622	Intracellular anatomical structure
rnb	511145.b1286	GO Component	GO:0005737	Cytoplasm
rnb	511145.b1286	GO Component	GO:0005829	Cytosol
rnb	511145.b1286	GO Component	GO:0110165	Cellular anatomical entity
rnb	511145.b1286	STRING clusters	CL:531	Mixed, incl. RNA helicase activity, and Exoribonuclease activity
rnb	511145.b1286	STRING clusters	CL:533	Exoribonuclease activity, and RNA degradation
rnb	511145.b1286	STRING clusters	CL:553	Exoribonuclease activity
rnb	511145.b1286	COMPARTMENTS	GOCC:0005622	Intracellular
rnb	511145.b1286	COMPARTMENTS	GOCC:0005737	Cytoplasm
rnb	511145.b1286	COMPARTMENTS	GOCC:0005829	Cytosol
rnb	511145.b1286	COMPARTMENTS	GOCC:0110165	Cellular anatomical entity
rnb	511145.b1286	UniProt Keywords	KW-0007	Acetylation
rnb	511145.b1286	UniProt Keywords	KW-0269	Exonuclease
rnb	511145.b1286	UniProt Keywords	KW-0378	Hydrolase
rnb	511145.b1286	UniProt Keywords	KW-0540	Nuclease
rnb	511145.b1286	UniProt Keywords	KW-0694	RNA-binding
rnb	511145.b1286	UniProt Keywords	KW-0963	Cytoplasm
rnb	511145.b1286	InterPro	IPR001900	Ribonuclease II/R
rnb	511145.b1286	InterPro	IPR003029	S1 domain
rnb	511145.b1286	InterPro	IPR004476	Ribonuclease II/ribonuclease R
rnb	511145.b1286	InterPro	IPR011129	Cold shock domain
rnb	511145.b1286	InterPro	IPR011804	Ribonuclease II
rnb	511145.b1286	InterPro	IPR012340	Nucleic acid-binding, OB-fold
rnb	511145.b1286	InterPro	IPR013223	Ribonuclease B, N-terminal OB domain
rnb	511145.b1286	InterPro	IPR022966	Ribonuclease II/R, conserved site
rnb	511145.b1286	InterPro	IPR022967	RNA-binding domain, S1
rnb	511145.b1286	InterPro	IPR040476	RNase II/RNase R, cold shock domain
rnb	511145.b1286	SMART	SM00316	Ribosomal protein S1-like RNA-binding domain
rnb	511145.b1286	SMART	SM00357	Cold shock protein domain
rnb	511145.b1286	SMART	SM00955	This domain is the catalytic domain of ribonuclease II.PUBMED:16806266
rplD	511145.b3319	GO Process	GO:0002181	Cytoplasmic translation
rplD	511145.b3319	GO Process	GO:0006139	Nucleobase-containing compound metabolic process
rplD	511145.b3319	GO Process	GO:0006351	Transcription, DNA-templated
rplD	511145.b3319	GO Process	GO:0006353	DNA-templated transcription, termination
rplD	511145.b3319	GO Process	GO:0006355	Regulation of transcription, DNA-templated
rplD	511145.b3319	GO Process	GO:0006412	Translation
rplD	511145.b3319	GO Process	GO:0006417	Regulation of translation
rplD	511145.b3319	GO Process	GO:0006518	Peptide metabolic process
rplD	511145.b3319	GO Process	GO:0006725	Cellular aromatic compound metabolic process
rplD	511145.b3319	GO Process	GO:0006807	Nitrogen compound metabolic process
rplD	511145.b3319	GO Process	GO:0006996	Organelle organization
rplD	511145.b3319	GO Process	GO:0008152	Metabolic process
rplD	511145.b3319	GO Process	GO:0009058	Biosynthetic process
rplD	511145.b3319	GO Process	GO:0009059	Macromolecule biosynthetic process
rplD	511145.b3319	GO Process	GO:0009889	Regulation of biosynthetic process
rplD	511145.b3319	GO Process	GO:0009890	Negative regulation of biosynthetic process
rplD	511145.b3319	GO Process	GO:0009891	Positive regulation of biosynthetic process
rplD	511145.b3319	GO Process	GO:0009892	Negative regulation of metabolic process
rplD	511145.b3319	GO Process	GO:0009893	Positive regulation of metabolic process
rplD	511145.b3319	GO Process	GO:0009987	Cellular process
rplD	511145.b3319	GO Process	GO:0010467	Gene expression
rplD	511145.b3319	GO Process	GO:0010468	Regulation of gene expression
rplD	511145.b3319	GO Process	GO:0010556	Regulation of macromolecule biosynthetic process
rplD	511145.b3319	GO Process	GO:0010557	Positive regulation of macromolecule biosynthetic process
rplD	511145.b3319	GO Process	GO:0010558	Negative regulation of macromolecule biosynthetic process
rplD	511145.b3319	GO Process	GO:0010604	Positive regulation of macromolecule metabolic process
rplD	511145.b3319	GO Process	GO:0010605	Negative regulation of macromolecule metabolic process
rplD	511145.b3319	GO Process	GO:0010608	Post-transcriptional regulation of gene expression
rplD	511145.b3319	GO Process	GO:0010629	Negative regulation of gene expression
rplD	511145.b3319	GO Process	GO:0016043	Cellular component organization
rplD	511145.b3319	GO Process	GO:0016070	RNA metabolic process
rplD	511145.b3319	GO Process	GO:0017148	Negative regulation of translation
rplD	511145.b3319	GO Process	GO:0018130	Heterocycle biosynthetic process
rplD	511145.b3319	GO Process	GO:0019219	Regulation of nucleobase-containing compound metabolic process
rplD	511145.b3319	GO Process	GO:0019222	Regulation of metabolic process
rplD	511145.b3319	GO Process	GO:0019438	Aromatic compound biosynthetic process
rplD	511145.b3319	GO Process	GO:0019538	Protein metabolic process
rplD	511145.b3319	GO Process	GO:0022607	Cellular component assembly
rplD	511145.b3319	GO Process	GO:0022613	Ribonucleoprotein complex biogenesis
rplD	511145.b3319	GO Process	GO:0031323	Regulation of cellular metabolic process
rplD	511145.b3319	GO Process	GO:0031324	Negative regulation of cellular metabolic process
rplD	511145.b3319	GO Process	GO:0031325	Positive regulation of cellular metabolic process
rplD	511145.b3319	GO Process	GO:0031326	Regulation of cellular biosynthetic process
rplD	511145.b3319	GO Process	GO:0031327	Negative regulation of cellular biosynthetic process
rplD	511145.b3319	GO Process	GO:0031328	Positive regulation of cellular biosynthetic process
rplD	511145.b3319	GO Process	GO:0031554	Regulation of DNA-templated transcription, termination
rplD	511145.b3319	GO Process	GO:0031555	Transcriptional attenuation
rplD	511145.b3319	GO Process	GO:0032069	Regulation of nuclease activity
rplD	511145.b3319	GO Process	GO:0032074	Negative regulation of nuclease activity
rplD	511145.b3319	GO Process	GO:0032774	RNA biosynthetic process
rplD	511145.b3319	GO Process	GO:0034248	Regulation of cellular amide metabolic process
rplD	511145.b3319	GO Process	GO:0034249	Negative regulation of cellular amide metabolic process
rplD	511145.b3319	GO Process	GO:0034641	Cellular nitrogen compound metabolic process
rplD	511145.b3319	GO Process	GO:0034645	Cellular macromolecule biosynthetic process
rplD	511145.b3319	GO Process	GO:0034654	Nucleobase-containing compound biosynthetic process
rplD	511145.b3319	GO Process	GO:0042221	Response to chemical
rplD	511145.b3319	GO Process	GO:0042254	Ribosome biogenesis
rplD	511145.b3319	GO Process	GO:0042255	Ribosome assembly
rplD	511145.b3319	GO Process	GO:0043043	Peptide biosynthetic process
rplD	511145.b3319	GO Process	GO:0043086	Negative regulation of catalytic activity
rplD	511145.b3319	GO Process	GO:0043170	Macromolecule metabolic process
rplD	511145.b3319	GO Process	GO:0043244	Regulation of protein-containing complex disassembly
rplD	511145.b3319	GO Process	GO:0043603	Cellular amide metabolic process
rplD	511145.b3319	GO Process	GO:0043604	Amide biosynthetic process
rplD	511145.b3319	GO Process	GO:0044085	Cellular component biogenesis
rplD	511145.b3319	GO Process	GO:0044092	Negative regulation of molecular function
rplD	511145.b3319	GO Process	GO:0044237	Cellular metabolic process
rplD	511145.b3319	GO Process	GO:0044238	Primary metabolic process
rplD	511145.b3319	GO Process	GO:0044249	Cellular biosynthetic process
rplD	511145.b3319	GO Process	GO:0044260	Cellular macromolecule metabolic process
rplD	511145.b3319	GO Process	GO:0044271	Cellular nitrogen compound biosynthetic process
rplD	511145.b3319	GO Process	GO:0045892	Negative regulation of transcription, DNA-templated
rplD	511145.b3319	GO Process	GO:0045893	Positive regulation of transcription, DNA-templated
rplD	511145.b3319	GO Process	GO:0045934	Negative regulation of nucleobase-containing compound metabolic process
rplD	511145.b3319	GO Process	GO:0045935	Positive regulation of nucleobase-containing compound metabolic process
rplD	511145.b3319	GO Process	GO:0046483	Heterocycle metabolic process
rplD	511145.b3319	GO Process	GO:0046677	Response to antibiotic
rplD	511145.b3319	GO Process	GO:0048518	Positive regulation of biological process
rplD	511145.b3319	GO Process	GO:0048519	Negative regulation of biological process
rplD	511145.b3319	GO Process	GO:0048522	Positive regulation of cellular process
rplD	511145.b3319	GO Process	GO:0048523	Negative regulation of cellular process
rplD	511145.b3319	GO Process	GO:0050789	Regulation of biological process
rplD	511145.b3319	GO Process	GO:0050790	Regulation of catalytic activity
rplD	511145.b3319	GO Process	GO:0050794	Regulation of cellular process
rplD	511145.b3319	GO Process	GO:0050896	Response to stimulus
rplD	511145.b3319	GO Process	GO:0051128	Regulation of cellular component organization
rplD	511145.b3319	GO Process	GO:0051171	Regulation of nitrogen compound metabolic process
rplD	511145.b3319	GO Process	GO:0051172	Negative regulation of nitrogen compound metabolic process
rplD	511145.b3319	GO Process	GO:0051173	Positive regulation of nitrogen compound metabolic process
rplD	511145.b3319	GO Process	GO:0051246	Regulation of protein metabolic process
rplD	511145.b3319	GO Process	GO:0051248	Negative regulation of protein metabolic process
rplD	511145.b3319	GO Process	GO:0051252	Regulation of RNA metabolic process
rplD	511145.b3319	GO Process	GO:0051253	Negative regulation of RNA metabolic process
rplD	511145.b3319	GO Process	GO:0051254	Positive regulation of RNA metabolic process
rplD	511145.b3319	GO Process	GO:0051336	Regulation of hydrolase activity
rplD	511145.b3319	GO Process	GO:0051346	Negative regulation of hydrolase activity
rplD	511145.b3319	GO Process	GO:0060255	Regulation of macromolecule metabolic process
rplD	511145.b3319	GO Process	GO:0060699	Regulation of endoribonuclease activity
rplD	511145.b3319	GO Process	GO:0060700	Regulation of ribonuclease activity
rplD	511145.b3319	GO Process	GO:0060701	Negative regulation of ribonuclease activity
rplD	511145.b3319	GO Process	GO:0060702	Negative regulation of endoribonuclease activity
rplD	511145.b3319	GO Process	GO:0065007	Biological regulation
rplD	511145.b3319	GO Process	GO:0065009	Regulation of molecular function
rplD	511145.b3319	GO Process	GO:0070925	Organelle assembly
rplD	511145.b3319	GO Process	GO:0071704	Organic substance metabolic process
rplD	511145.b3319	GO Process	GO:0071840	Cellular component organization or biogenesis
rplD	511145.b3319	GO Process	GO:0080090	Regulation of primary metabolic process
rplD	511145.b3319	GO Process	GO:0090304	Nucleic acid metabolic process
rplD	511145.b3319	GO Process	GO:0097659	Nucleic acid-templated transcription
rplD	511145.b3319	GO Process	GO:0140694	Non-membrane-bounded organelle assembly
rplD	511145.b3319	GO Process	GO:1901360	Organic cyclic compound metabolic process
rplD	511145.b3319	GO Process	GO:1901362	Organic cyclic compound biosynthetic process
rplD	511145.b3319	GO Process	GO:1901564	Organonitrogen compound metabolic process
rplD	511145.b3319	GO Process	GO:1901566	Organonitrogen compound biosynthetic process
rplD	511145.b3319	GO Process	GO:1901576	Organic substance biosynthetic process
rplD	511145.b3319	GO Process	GO:1902679	Negative regulation of RNA biosynthetic process
rplD	511145.b3319	GO Process	GO:1902680	Positive regulation of RNA biosynthetic process
rplD	511145.b3319	GO Process	GO:1903506	Regulation of nucleic acid-templated transcription
rplD	511145.b3319	GO Process	GO:1903507	Negative regulation of nucleic acid-templated transcription
rplD	511145.b3319	GO Process	GO:1903508	Positive regulation of nucleic acid-templated transcription
rplD	511145.b3319	GO Process	GO:2000112	Regulation of cellular macromolecule biosynthetic process
rplD	511145.b3319	GO Process	GO:2000113	Negative regulation of cellular macromolecule biosynthetic process
rplD	511145.b3319	GO Process	GO:2000765	Regulation of cytoplasmic translation
rplD	511145.b3319	GO Process	GO:2000766	Negative regulation of cytoplasmic translation
rplD	511145.b3319	GO Process	GO:2001141	Regulation of RNA biosynthetic process
rplD	511145.b3319	GO Function	GO:0001070	RNA-binding transcription regulator activity
rplD	511145.b3319	GO Function	GO:0003676	Nucleic acid binding
rplD	511145.b3319	GO Function	GO:0003723	RNA binding
rplD	511145.b3319	GO Function	GO:0003729	mRNA binding
rplD	511145.b3319	GO Function	GO:0003735	Structural constituent of ribosome
rplD	511145.b3319	GO Function	GO:0004857	Enzyme inhibitor activity
rplD	511145.b3319	GO Function	GO:0005198	Structural molecule activity
rplD	511145.b3319	GO Function	GO:0005488	Binding
rplD	511145.b3319	GO Function	GO:0008428	Ribonuclease inhibitor activity
rplD	511145.b3319	GO Function	GO:0019843	rRNA binding
rplD	511145.b3319	GO Function	GO:0030234	Enzyme regulator activity
rplD	511145.b3319	GO Function	GO:0030371	Translation repressor activity
rplD	511145.b3319	GO Function	GO:0045182	Translation regulator activity
rplD	511145.b3319	GO Function	GO:0048027	mRNA 5-UTR binding
rplD	511145.b3319	GO Function	GO:0060698	Endoribonuclease inhibitor activity
rplD	511145.b3319	GO Function	GO:0097159	Organic cyclic compound binding
rplD	511145.b3319	GO Function	GO:0098772	Molecular function regulator activity
rplD	511145.b3319	GO Function	GO:0140110	Transcription regulator activity
rplD	511145.b3319	GO Function	GO:0140721	Nuclease inhibitor activity
rplD	511145.b3319	GO Function	GO:1901363	Heterocyclic compound binding
rplD	511145.b3319	GO Component	GO:0005622	Intracellular anatomical structure
rplD	511145.b3319	GO Component	GO:0005737	Cytoplasm
rplD	511145.b3319	GO Component	GO:0005829	Cytosol
rplD	511145.b3319	GO Component	GO:0005840	Ribosome
rplD	511145.b3319	GO Component	GO:0005844	Polysome
rplD	511145.b3319	GO Component	GO:0015934	Large ribosomal subunit
rplD	511145.b3319	GO Component	GO:0022625	Cytosolic large ribosomal subunit
rplD	511145.b3319	GO Component	GO:0022626	Cytosolic ribosome
rplD	511145.b3319	GO Component	GO:0032991	Protein-containing complex
rplD	511145.b3319	GO Component	GO:0042788	Polysomal ribosome
rplD	511145.b3319	GO Component	GO:0043226	Organelle
rplD	511145.b3319	GO Component	GO:0043228	Non-membrane-bounded organelle
rplD	511145.b3319	GO Component	GO:0043229	Intracellular organelle
rplD	511145.b3319	GO Component	GO:0043232	Intracellular non-membrane-bounded organelle
rplD	511145.b3319	GO Component	GO:0044391	Ribosomal subunit
rplD	511145.b3319	GO Component	GO:0110165	Cellular anatomical entity
rplD	511145.b3319	GO Component	GO:1990904	Ribonucleoprotein complex
rplD	511145.b3319	STRING clusters	CL:110	Ribosome
rplD	511145.b3319	STRING clusters	CL:112	Large ribosomal subunit rRNA binding, and Methylation
rplD	511145.b3319	STRING clusters	CL:114	Large ribosomal subunit rRNA binding, and Polysome
rplD	511145.b3319	STRING clusters	CL:35	Translation, and Protein export
rplD	511145.b3319	STRING clusters	CL:37	Translation, and Protein export
rplD	511145.b3319	STRING clusters	CL:39	Mixed, incl. Ribonucleoprotein, and Translation regulator activity
rplD	511145.b3319	STRING clusters	CL:40	Ribonucleoprotein, and Translation factor activity, RNA binding
rplD	511145.b3319	STRING clusters	CL:41	Ribonucleoprotein, and Translation factor activity, RNA binding
rplD	511145.b3319	STRING clusters	CL:42	Ribosome, and Translation factor activity, RNA binding
rplD	511145.b3319	STRING clusters	CL:45	Ribosome, and Translation factor activity, RNA binding
rplD	511145.b3319	STRING clusters	CL:47	Ribosome, and Translation factor activity, RNA binding
rplD	511145.b3319	STRING clusters	CL:49	Ribosome
rplD	511145.b3319	STRING clusters	CL:51	Ribosome
rplD	511145.b3319	STRING clusters	CL:54	Ribosome
rplD	511145.b3319	KEGG	eco03010	Ribosome
rplD	511145.b3319	COMPARTMENTS	GOCC:0005622	Intracellular
rplD	511145.b3319	COMPARTMENTS	GOCC:0005737	Cytoplasm
rplD	511145.b3319	COMPARTMENTS	GOCC:0005829	Cytosol
rplD	511145.b3319	COMPARTMENTS	GOCC:0005840	Ribosome
rplD	511145.b3319	COMPARTMENTS	GOCC:0005844	Polysome
rplD	511145.b3319	COMPARTMENTS	GOCC:0015934	Large ribosomal subunit
rplD	511145.b3319	COMPARTMENTS	GOCC:0022625	Cytosolic large ribosomal subunit
rplD	511145.b3319	COMPARTMENTS	GOCC:0022626	Cytosolic ribosome
rplD	511145.b3319	COMPARTMENTS	GOCC:0032991	Protein-containing complex
rplD	511145.b3319	COMPARTMENTS	GOCC:0042788	Polysomal ribosome
rplD	511145.b3319	COMPARTMENTS	GOCC:0043226	Organelle
rplD	511145.b3319	COMPARTMENTS	GOCC:0043228	Non-membrane-bounded organelle
rplD	511145.b3319	COMPARTMENTS	GOCC:0043229	Intracellular organelle
rplD	511145.b3319	COMPARTMENTS	GOCC:0043232	Intracellular non-membrane-bounded organelle
rplD	511145.b3319	COMPARTMENTS	GOCC:0044391	Ribosomal subunit
rplD	511145.b3319	COMPARTMENTS	GOCC:0110165	Cellular anatomical entity
rplD	511145.b3319	COMPARTMENTS	GOCC:1990904	Ribonucleoprotein complex
rplD	511145.b3319	UniProt Keywords	KW-0046	Antibiotic resistance
rplD	511145.b3319	UniProt Keywords	KW-0678	Repressor
rplD	511145.b3319	UniProt Keywords	KW-0687	Ribonucleoprotein
rplD	511145.b3319	UniProt Keywords	KW-0689	Ribosomal protein
rplD	511145.b3319	UniProt Keywords	KW-0694	RNA-binding
rplD	511145.b3319	UniProt Keywords	KW-0699	rRNA-binding
rplD	511145.b3319	UniProt Keywords	KW-0804	Transcription
rplD	511145.b3319	UniProt Keywords	KW-0805	Transcription regulation
rplD	511145.b3319	UniProt Keywords	KW-0806	Transcription termination
rplD	511145.b3319	UniProt Keywords	KW-0810	Translation regulation
rplD	511145.b3319	Pfam	PF00573	Ribosomal protein L4/L1 family
rplD	511145.b3319	InterPro	IPR002136	Ribosomal protein L4/L1e
rplD	511145.b3319	InterPro	IPR013005	50S ribosomal protein uL4
rplD	511145.b3319	InterPro	IPR023574	Ribosomal protein L4 domain superfamily
rplM	511145.b3231	GO Process	GO:0002181	Cytoplasmic translation
rplM	511145.b3231	GO Process	GO:0006412	Translation
rplM	511145.b3231	GO Process	GO:0006417	Regulation of translation
rplM	511145.b3231	GO Process	GO:0006518	Peptide metabolic process
rplM	511145.b3231	GO Process	GO:0006807	Nitrogen compound metabolic process
rplM	511145.b3231	GO Process	GO:0008152	Metabolic process
rplM	511145.b3231	GO Process	GO:0009058	Biosynthetic process
rplM	511145.b3231	GO Process	GO:0009059	Macromolecule biosynthetic process
rplM	511145.b3231	GO Process	GO:0009889	Regulation of biosynthetic process
rplM	511145.b3231	GO Process	GO:0009890	Negative regulation of biosynthetic process
rplM	511145.b3231	GO Process	GO:0009892	Negative regulation of metabolic process
rplM	511145.b3231	GO Process	GO:0009987	Cellular process
rplM	511145.b3231	GO Process	GO:0010467	Gene expression
rplM	511145.b3231	GO Process	GO:0010468	Regulation of gene expression
rplM	511145.b3231	GO Process	GO:0010556	Regulation of macromolecule biosynthetic process
rplM	511145.b3231	GO Process	GO:0010558	Negative regulation of macromolecule biosynthetic process
rplM	511145.b3231	GO Process	GO:0010605	Negative regulation of macromolecule metabolic process
rplM	511145.b3231	GO Process	GO:0010608	Post-transcriptional regulation of gene expression
rplM	511145.b3231	GO Process	GO:0010629	Negative regulation of gene expression
rplM	511145.b3231	GO Process	GO:0017148	Negative regulation of translation
rplM	511145.b3231	GO Process	GO:0019222	Regulation of metabolic process
rplM	511145.b3231	GO Process	GO:0019538	Protein metabolic process
rplM	511145.b3231	GO Process	GO:0031323	Regulation of cellular metabolic process
rplM	511145.b3231	GO Process	GO:0031324	Negative regulation of cellular metabolic process
rplM	511145.b3231	GO Process	GO:0031326	Regulation of cellular biosynthetic process
rplM	511145.b3231	GO Process	GO:0031327	Negative regulation of cellular biosynthetic process
rplM	511145.b3231	GO Process	GO:0034248	Regulation of cellular amide metabolic process
rplM	511145.b3231	GO Process	GO:0034249	Negative regulation of cellular amide metabolic process
rplM	511145.b3231	GO Process	GO:0034641	Cellular nitrogen compound metabolic process
rplM	511145.b3231	GO Process	GO:0034645	Cellular macromolecule biosynthetic process
rplM	511145.b3231	GO Process	GO:0043043	Peptide biosynthetic process
rplM	511145.b3231	GO Process	GO:0043170	Macromolecule metabolic process
rplM	511145.b3231	GO Process	GO:0043603	Cellular amide metabolic process
rplM	511145.b3231	GO Process	GO:0043604	Amide biosynthetic process
rplM	511145.b3231	GO Process	GO:0044237	Cellular metabolic process
rplM	511145.b3231	GO Process	GO:0044238	Primary metabolic process
rplM	511145.b3231	GO Process	GO:0044249	Cellular biosynthetic process
rplM	511145.b3231	GO Process	GO:0044260	Cellular macromolecule metabolic process
rplM	511145.b3231	GO Process	GO:0044271	Cellular nitrogen compound biosynthetic process
rplM	511145.b3231	GO Process	GO:0048519	Negative regulation of biological process
rplM	511145.b3231	GO Process	GO:0048523	Negative regulation of cellular process
rplM	511145.b3231	GO Process	GO:0050789	Regulation of biological process
rplM	511145.b3231	GO Process	GO:0050794	Regulation of cellular process
rplM	511145.b3231	GO Process	GO:0051171	Regulation of nitrogen compound metabolic process
rplM	511145.b3231	GO Process	GO:0051172	Negative regulation of nitrogen compound metabolic process
rplM	511145.b3231	GO Process	GO:0051246	Regulation of protein metabolic process
rplM	511145.b3231	GO Process	GO:0051248	Negative regulation of protein metabolic process
rplM	511145.b3231	GO Process	GO:0060255	Regulation of macromolecule metabolic process
rplM	511145.b3231	GO Process	GO:0065007	Biological regulation
rplM	511145.b3231	GO Process	GO:0071704	Organic substance metabolic process
rplM	511145.b3231	GO Process	GO:0080090	Regulation of primary metabolic process
rplM	511145.b3231	GO Process	GO:1901564	Organonitrogen compound metabolic process
rplM	511145.b3231	GO Process	GO:1901566	Organonitrogen compound biosynthetic process
rplM	511145.b3231	GO Process	GO:1901576	Organic substance biosynthetic process
rplM	511145.b3231	GO Process	GO:2000112	Regulation of cellular macromolecule biosynthetic process
rplM	511145.b3231	GO Process	GO:2000113	Negative regulation of cellular macromolecule biosynthetic process
rplM	511145.b3231	GO Function	GO:0003676	Nucleic acid binding
rplM	511145.b3231	GO Function	GO:0003723	RNA binding
rplM	511145.b3231	GO Function	GO:0003729	mRNA binding
rplM	511145.b3231	GO Function	GO:0003735	Structural constituent of ribosome
rplM	511145.b3231	GO Function	GO:0005198	Structural molecule activity
rplM	511145.b3231	GO Function	GO:0005488	Binding
rplM	511145.b3231	GO Function	GO:0008270	Zinc ion binding
rplM	511145.b3231	GO Function	GO:0019843	rRNA binding
rplM	511145.b3231	GO Function	GO:0043167	Ion binding
rplM	511145.b3231	GO Function	GO:0043169	Cation binding
rplM	511145.b3231	GO Function	GO:0046872	Metal ion binding
rplM	511145.b3231	GO Function	GO:0046914	Transition metal ion binding
rplM	511145.b3231	GO Function	GO:0048027	mRNA 5-UTR binding
rplM	511145.b3231	GO Function	GO:0070180	Large ribosomal subunit rRNA binding
rplM	511145.b3231	GO Function	GO:0097159	Organic cyclic compound binding
rplM	511145.b3231	GO Function	GO:1901363	Heterocyclic compound binding
rplM	511145.b3231	GO Component	GO:0005622	Intracellular anatomical structure
rplM	511145.b3231	GO Component	GO:0005737	Cytoplasm
rplM	511145.b3231	GO Component	GO:0005829	Cytosol
rplM	511145.b3231	GO Component	GO:0005840	Ribosome
rplM	511145.b3231	GO Component	GO:0015934	Large ribosomal subunit
rplM	511145.b3231	GO Component	GO:0022625	Cytosolic large ribosomal subunit
rplM	511145.b3231	GO Component	GO:0022626	Cytosolic ribosome
rplM	511145.b3231	GO Component	GO:0032991	Protein-containing complex
rplM	511145.b3231	GO Component	GO:0043226	Organelle
rplM	511145.b3231	GO Component	GO:0043228	Non-membrane-bounded organelle
rplM	511145.b3231	GO Component	GO:0043229	Intracellular organelle
rplM	511145.b3231	GO Component	GO:0043232	Intracellular non-membrane-bounded organelle
rplM	511145.b3231	GO Component	GO:0044391	Ribosomal subunit
rplM	511145.b3231	GO Component	GO:0110165	Cellular anatomical entity
rplM	511145.b3231	GO Component	GO:1990904	Ribonucleoprotein complex
rplM	511145.b3231	STRING clusters	CL:110	Ribosome
rplM	511145.b3231	STRING clusters	CL:112	Large ribosomal subunit rRNA binding, and Methylation
rplM	511145.b3231	STRING clusters	CL:114	Large ribosomal subunit rRNA binding, and Polysome
rplM	511145.b3231	STRING clusters	CL:35	Translation, and Protein export
rplM	511145.b3231	STRING clusters	CL:37	Translation, and Protein export
rplM	511145.b3231	STRING clusters	CL:39	Mixed, incl. Ribonucleoprotein, and Translation regulator activity
rplM	511145.b3231	STRING clusters	CL:40	Ribonucleoprotein, and Translation factor activity, RNA binding
rplM	511145.b3231	STRING clusters	CL:41	Ribonucleoprotein, and Translation factor activity, RNA binding
rplM	511145.b3231	STRING clusters	CL:42	Ribosome, and Translation factor activity, RNA binding
rplM	511145.b3231	STRING clusters	CL:45	Ribosome, and Translation factor activity, RNA binding
rplM	511145.b3231	STRING clusters	CL:47	Ribosome, and Translation factor activity, RNA binding
rplM	511145.b3231	STRING clusters	CL:49	Ribosome
rplM	511145.b3231	STRING clusters	CL:51	Ribosome
rplM	511145.b3231	STRING clusters	CL:54	Ribosome
rplM	511145.b3231	KEGG	eco03010	Ribosome
rplM	511145.b3231	COMPARTMENTS	GOCC:0005622	Intracellular
rplM	511145.b3231	COMPARTMENTS	GOCC:0005737	Cytoplasm
rplM	511145.b3231	COMPARTMENTS	GOCC:0005829	Cytosol
rplM	511145.b3231	COMPARTMENTS	GOCC:0005840	Ribosome
rplM	511145.b3231	COMPARTMENTS	GOCC:0015934	Large ribosomal subunit
rplM	511145.b3231	COMPARTMENTS	GOCC:0022625	Cytosolic large ribosomal subunit
rplM	511145.b3231	COMPARTMENTS	GOCC:0022626	Cytosolic ribosome
rplM	511145.b3231	COMPARTMENTS	GOCC:0032991	Protein-containing complex
rplM	511145.b3231	COMPARTMENTS	GOCC:0043226	Organelle
rplM	511145.b3231	COMPARTMENTS	GOCC:0043228	Non-membrane-bounded organelle
rplM	511145.b3231	COMPARTMENTS	GOCC:0043229	Intracellular organelle
rplM	511145.b3231	COMPARTMENTS	GOCC:0043232	Intracellular non-membrane-bounded organelle
rplM	511145.b3231	COMPARTMENTS	GOCC:0044391	Ribosomal subunit
rplM	511145.b3231	COMPARTMENTS	GOCC:0110165	Cellular anatomical entity
rplM	511145.b3231	COMPARTMENTS	GOCC:1990904	Ribonucleoprotein complex
rplM	511145.b3231	UniProt Keywords	KW-0687	Ribonucleoprotein
rplM	511145.b3231	UniProt Keywords	KW-0689	Ribosomal protein
rplM	511145.b3231	Pfam	PF00572	Ribosomal protein L13
rplM	511145.b3231	InterPro	IPR005822	Ribosomal protein L13
rplM	511145.b3231	InterPro	IPR005823	Ribosomal protein L13, bacterial-type
rplM	511145.b3231	InterPro	IPR023563	Ribosomal protein L13, conserved site
rplM	511145.b3231	InterPro	IPR036899	Ribosomal protein L13 superfamily
rplO	511145.b3301	GO Process	GO:0002181	Cytoplasmic translation
rplO	511145.b3301	GO Process	GO:0006412	Translation
rplO	511145.b3301	GO Process	GO:0006518	Peptide metabolic process
rplO	511145.b3301	GO Process	GO:0006807	Nitrogen compound metabolic process
rplO	511145.b3301	GO Process	GO:0008152	Metabolic process
rplO	511145.b3301	GO Process	GO:0009058	Biosynthetic process
rplO	511145.b3301	GO Process	GO:0009059	Macromolecule biosynthetic process
rplO	511145.b3301	GO Process	GO:0009987	Cellular process
rplO	511145.b3301	GO Process	GO:0010467	Gene expression
rplO	511145.b3301	GO Process	GO:0019538	Protein metabolic process
rplO	511145.b3301	GO Process	GO:0034641	Cellular nitrogen compound metabolic process
rplO	511145.b3301	GO Process	GO:0034645	Cellular macromolecule biosynthetic process
rplO	511145.b3301	GO Process	GO:0043043	Peptide biosynthetic process
rplO	511145.b3301	GO Process	GO:0043170	Macromolecule metabolic process
rplO	511145.b3301	GO Process	GO:0043603	Cellular amide metabolic process
rplO	511145.b3301	GO Process	GO:0043604	Amide biosynthetic process
rplO	511145.b3301	GO Process	GO:0044237	Cellular metabolic process
rplO	511145.b3301	GO Process	GO:0044238	Primary metabolic process
rplO	511145.b3301	GO Process	GO:0044249	Cellular biosynthetic process
rplO	511145.b3301	GO Process	GO:0044260	Cellular macromolecule metabolic process
rplO	511145.b3301	GO Process	GO:0044271	Cellular nitrogen compound biosynthetic process
rplO	511145.b3301	GO Process	GO:0071704	Organic substance metabolic process
rplO	511145.b3301	GO Process	GO:1901564	Organonitrogen compound metabolic process
rplO	511145.b3301	GO Process	GO:1901566	Organonitrogen compound biosynthetic process
rplO	511145.b3301	GO Process	GO:1901576	Organic substance biosynthetic process
rplO	511145.b3301	GO Function	GO:0003676	Nucleic acid binding
rplO	511145.b3301	GO Function	GO:0003723	RNA binding
rplO	511145.b3301	GO Function	GO:0003735	Structural constituent of ribosome
rplO	511145.b3301	GO Function	GO:0005198	Structural molecule activity
rplO	511145.b3301	GO Function	GO:0005488	Binding
rplO	511145.b3301	GO Function	GO:0019843	rRNA binding
rplO	511145.b3301	GO Function	GO:0097159	Organic cyclic compound binding
rplO	511145.b3301	GO Function	GO:1901363	Heterocyclic compound binding
rplO	511145.b3301	GO Component	GO:0005622	Intracellular anatomical structure
rplO	511145.b3301	GO Component	GO:0005737	Cytoplasm
rplO	511145.b3301	GO Component	GO:0005829	Cytosol
rplO	511145.b3301	GO Component	GO:0005840	Ribosome
rplO	511145.b3301	GO Component	GO:0015934	Large ribosomal subunit
rplO	511145.b3301	GO Component	GO:0022625	Cytosolic large ribosomal subunit
rplO	511145.b3301	GO Component	GO:0022626	Cytosolic ribosome
rplO	511145.b3301	GO Component	GO:0032991	Protein-containing complex
rplO	511145.b3301	GO Component	GO:0043226	Organelle
rplO	511145.b3301	GO Component	GO:0043228	Non-membrane-bounded organelle
rplO	511145.b3301	GO Component	GO:0043229	Intracellular organelle
rplO	511145.b3301	GO Component	GO:0043232	Intracellular non-membrane-bounded organelle
rplO	511145.b3301	GO Component	GO:0044391	Ribosomal subunit
rplO	511145.b3301	GO Component	GO:0110165	Cellular anatomical entity
rplO	511145.b3301	GO Component	GO:1990904	Ribonucleoprotein complex
rplO	511145.b3301	STRING clusters	CL:35	Translation, and Protein export
rplO	511145.b3301	STRING clusters	CL:37	Translation, and Protein export
rplO	511145.b3301	STRING clusters	CL:39	Mixed, incl. Ribonucleoprotein, and Translation regulator activity
rplO	511145.b3301	STRING clusters	CL:40	Ribonucleoprotein, and Translation factor activity, RNA binding
rplO	511145.b3301	STRING clusters	CL:41	Ribonucleoprotein, and Translation factor activity, RNA binding
rplO	511145.b3301	STRING clusters	CL:42	Ribosome, and Translation factor activity, RNA binding
rplO	511145.b3301	STRING clusters	CL:45	Ribosome, and Translation factor activity, RNA binding
rplO	511145.b3301	STRING clusters	CL:47	Ribosome, and Translation factor activity, RNA binding
rplO	511145.b3301	STRING clusters	CL:49	Ribosome
rplO	511145.b3301	STRING clusters	CL:51	Ribosome
rplO	511145.b3301	STRING clusters	CL:54	Ribosome
rplO	511145.b3301	STRING clusters	CL:56	Ribosome
rplO	511145.b3301	STRING clusters	CL:57	Small ribosomal subunit, and Rescue of stalled ribosome
rplO	511145.b3301	KEGG	eco03010	Ribosome
rplO	511145.b3301	COMPARTMENTS	GOCC:0005622	Intracellular
rplO	511145.b3301	COMPARTMENTS	GOCC:0005737	Cytoplasm
rplO	511145.b3301	COMPARTMENTS	GOCC:0005829	Cytosol
rplO	511145.b3301	COMPARTMENTS	GOCC:0005840	Ribosome
rplO	511145.b3301	COMPARTMENTS	GOCC:0015934	Large ribosomal subunit
rplO	511145.b3301	COMPARTMENTS	GOCC:0022625	Cytosolic large ribosomal subunit
rplO	511145.b3301	COMPARTMENTS	GOCC:0022626	Cytosolic ribosome
rplO	511145.b3301	COMPARTMENTS	GOCC:0032991	Protein-containing complex
rplO	511145.b3301	COMPARTMENTS	GOCC:0043226	Organelle
rplO	511145.b3301	COMPARTMENTS	GOCC:0043228	Non-membrane-bounded organelle
rplO	511145.b3301	COMPARTMENTS	GOCC:0043229	Intracellular organelle
rplO	511145.b3301	COMPARTMENTS	GOCC:0043232	Intracellular non-membrane-bounded organelle
rplO	511145.b3301	COMPARTMENTS	GOCC:0044391	Ribosomal subunit
rplO	511145.b3301	COMPARTMENTS	GOCC:0110165	Cellular anatomical entity
rplO	511145.b3301	COMPARTMENTS	GOCC:1990904	Ribonucleoprotein complex
rplO	511145.b3301	UniProt Keywords	KW-0687	Ribonucleoprotein
rplO	511145.b3301	UniProt Keywords	KW-0689	Ribosomal protein
rplO	511145.b3301	UniProt Keywords	KW-0694	RNA-binding
rplO	511145.b3301	UniProt Keywords	KW-0699	rRNA-binding
rplO	511145.b3301	Pfam	PF00828	Ribosomal proteins 50S-L15, 50S-L18e, 60S-L27A
rplO	511145.b3301	InterPro	IPR001196	Ribosomal protein L15, conserved site
rplO	511145.b3301	InterPro	IPR005749	Ribosomal protein L15, bacterial-type
rplO	511145.b3301	InterPro	IPR021131	Ribosomal protein L18e/L15P
rplO	511145.b3301	InterPro	IPR030878	Ribosomal protein L15
rplO	511145.b3301	InterPro	IPR036227	Ribosomal L18e/L15P superfamily
rpmE	511145.b3936	GO Process	GO:0002181	Cytoplasmic translation
rpmE	511145.b3936	GO Process	GO:0006412	Translation
rpmE	511145.b3936	GO Process	GO:0006413	Translational initiation
rpmE	511145.b3936	GO Process	GO:0006417	Regulation of translation
rpmE	511145.b3936	GO Process	GO:0006446	Regulation of translational initiation
rpmE	511145.b3936	GO Process	GO:0006518	Peptide metabolic process
rpmE	511145.b3936	GO Process	GO:0006807	Nitrogen compound metabolic process
rpmE	511145.b3936	GO Process	GO:0008152	Metabolic process
rpmE	511145.b3936	GO Process	GO:0009058	Biosynthetic process
rpmE	511145.b3936	GO Process	GO:0009059	Macromolecule biosynthetic process
rpmE	511145.b3936	GO Process	GO:0009889	Regulation of biosynthetic process
rpmE	511145.b3936	GO Process	GO:0009890	Negative regulation of biosynthetic process
rpmE	511145.b3936	GO Process	GO:0009892	Negative regulation of metabolic process
rpmE	511145.b3936	GO Process	GO:0009987	Cellular process
rpmE	511145.b3936	GO Process	GO:0010467	Gene expression
rpmE	511145.b3936	GO Process	GO:0010468	Regulation of gene expression
rpmE	511145.b3936	GO Process	GO:0010556	Regulation of macromolecule biosynthetic process
rpmE	511145.b3936	GO Process	GO:0010558	Negative regulation of macromolecule biosynthetic process
rpmE	511145.b3936	GO Process	GO:0010605	Negative regulation of macromolecule metabolic process
rpmE	511145.b3936	GO Process	GO:0010608	Post-transcriptional regulation of gene expression
rpmE	511145.b3936	GO Process	GO:0010629	Negative regulation of gene expression
rpmE	511145.b3936	GO Process	GO:0017148	Negative regulation of translation
rpmE	511145.b3936	GO Process	GO:0019222	Regulation of metabolic process
rpmE	511145.b3936	GO Process	GO:0019538	Protein metabolic process
rpmE	511145.b3936	GO Process	GO:0031323	Regulation of cellular metabolic process
rpmE	511145.b3936	GO Process	GO:0031324	Negative regulation of cellular metabolic process
rpmE	511145.b3936	GO Process	GO:0031326	Regulation of cellular biosynthetic process
rpmE	511145.b3936	GO Process	GO:0031327	Negative regulation of cellular biosynthetic process
rpmE	511145.b3936	GO Process	GO:0034248	Regulation of cellular amide metabolic process
rpmE	511145.b3936	GO Process	GO:0034249	Negative regulation of cellular amide metabolic process
rpmE	511145.b3936	GO Process	GO:0034641	Cellular nitrogen compound metabolic process
rpmE	511145.b3936	GO Process	GO:0034645	Cellular macromolecule biosynthetic process
rpmE	511145.b3936	GO Process	GO:0043043	Peptide biosynthetic process
rpmE	511145.b3936	GO Process	GO:0043170	Macromolecule metabolic process
rpmE	511145.b3936	GO Process	GO:0043603	Cellular amide metabolic process
rpmE	511145.b3936	GO Process	GO:0043604	Amide biosynthetic process
rpmE	511145.b3936	GO Process	GO:0044237	Cellular metabolic process
rpmE	511145.b3936	GO Process	GO:0044238	Primary metabolic process
rpmE	511145.b3936	GO Process	GO:0044249	Cellular biosynthetic process
rpmE	511145.b3936	GO Process	GO:0044260	Cellular macromolecule metabolic process
rpmE	511145.b3936	GO Process	GO:0044271	Cellular nitrogen compound biosynthetic process
rpmE	511145.b3936	GO Process	GO:0045947	Negative regulation of translational initiation
rpmE	511145.b3936	GO Process	GO:0048519	Negative regulation of biological process
rpmE	511145.b3936	GO Process	GO:0048523	Negative regulation of cellular process
rpmE	511145.b3936	GO Process	GO:0050789	Regulation of biological process
rpmE	511145.b3936	GO Process	GO:0050794	Regulation of cellular process
rpmE	511145.b3936	GO Process	GO:0051171	Regulation of nitrogen compound metabolic process
rpmE	511145.b3936	GO Process	GO:0051172	Negative regulation of nitrogen compound metabolic process
rpmE	511145.b3936	GO Process	GO:0051246	Regulation of protein metabolic process
rpmE	511145.b3936	GO Process	GO:0051248	Negative regulation of protein metabolic process
rpmE	511145.b3936	GO Process	GO:0060255	Regulation of macromolecule metabolic process
rpmE	511145.b3936	GO Process	GO:0065007	Biological regulation
rpmE	511145.b3936	GO Process	GO:0071704	Organic substance metabolic process
rpmE	511145.b3936	GO Process	GO:0080090	Regulation of primary metabolic process
rpmE	511145.b3936	GO Process	GO:1901564	Organonitrogen compound metabolic process
rpmE	511145.b3936	GO Process	GO:1901566	Organonitrogen compound biosynthetic process
rpmE	511145.b3936	GO Process	GO:1901576	Organic substance biosynthetic process
rpmE	511145.b3936	GO Process	GO:1904688	Regulation of cytoplasmic translational initiation
rpmE	511145.b3936	GO Process	GO:1904689	Negative regulation of cytoplasmic translational initiation
rpmE	511145.b3936	GO Process	GO:2000112	Regulation of cellular macromolecule biosynthetic process
rpmE	511145.b3936	GO Process	GO:2000113	Negative regulation of cellular macromolecule biosynthetic process
rpmE	511145.b3936	GO Process	GO:2000765	Regulation of cytoplasmic translation
rpmE	511145.b3936	GO Function	GO:0003676	Nucleic acid binding
rpmE	511145.b3936	GO Function	GO:0003723	RNA binding
rpmE	511145.b3936	GO Function	GO:0003735	Structural constituent of ribosome
rpmE	511145.b3936	GO Function	GO:0005198	Structural molecule activity
rpmE	511145.b3936	GO Function	GO:0005488	Binding
rpmE	511145.b3936	GO Function	GO:0008270	Zinc ion binding
rpmE	511145.b3936	GO Function	GO:0019843	rRNA binding
rpmE	511145.b3936	GO Function	GO:0043167	Ion binding
rpmE	511145.b3936	GO Function	GO:0043169	Cation binding
rpmE	511145.b3936	GO Function	GO:0046872	Metal ion binding
rpmE	511145.b3936	GO Function	GO:0046914	Transition metal ion binding
rpmE	511145.b3936	GO Function	GO:0097159	Organic cyclic compound binding
rpmE	511145.b3936	GO Function	GO:1901363	Heterocyclic compound binding
rpmE	511145.b3936	GO Component	GO:0005622	Intracellular anatomical structure
rpmE	511145.b3936	GO Component	GO:0005737	Cytoplasm
rpmE	511145.b3936	GO Component	GO:0005829	Cytosol
rpmE	511145.b3936	GO Component	GO:0005840	Ribosome
rpmE	511145.b3936	GO Component	GO:0015934	Large ribosomal subunit
rpmE	511145.b3936	GO Component	GO:0022625	Cytosolic large ribosomal subunit
rpmE	511145.b3936	GO Component	GO:0022626	Cytosolic ribosome
rpmE	511145.b3936	GO Component	GO:0032991	Protein-containing complex
rpmE	511145.b3936	GO Component	GO:0043226	Organelle
rpmE	511145.b3936	GO Component	GO:0043228	Non-membrane-bounded organelle
rpmE	511145.b3936	GO Component	GO:0043229	Intracellular organelle
rpmE	511145.b3936	GO Component	GO:0043232	Intracellular non-membrane-bounded organelle
rpmE	511145.b3936	GO Component	GO:0044391	Ribosomal subunit
rpmE	511145.b3936	GO Component	GO:0110165	Cellular anatomical entity
rpmE	511145.b3936	GO Component	GO:1990904	Ribonucleoprotein complex
rpmE	511145.b3936	STRING clusters	CL:110	Ribosome
rpmE	511145.b3936	STRING clusters	CL:142	Large ribosomal subunit, and Small ribosomal subunit rRNA binding
rpmE	511145.b3936	STRING clusters	CL:35	Translation, and Protein export
rpmE	511145.b3936	STRING clusters	CL:37	Translation, and Protein export
rpmE	511145.b3936	STRING clusters	CL:39	Mixed, incl. Ribonucleoprotein, and Translation regulator activity
rpmE	511145.b3936	STRING clusters	CL:40	Ribonucleoprotein, and Translation factor activity, RNA binding
rpmE	511145.b3936	STRING clusters	CL:41	Ribonucleoprotein, and Translation factor activity, RNA binding
rpmE	511145.b3936	STRING clusters	CL:42	Ribosome, and Translation factor activity, RNA binding
rpmE	511145.b3936	STRING clusters	CL:45	Ribosome, and Translation factor activity, RNA binding
rpmE	511145.b3936	STRING clusters	CL:47	Ribosome, and Translation factor activity, RNA binding
rpmE	511145.b3936	STRING clusters	CL:49	Ribosome
rpmE	511145.b3936	STRING clusters	CL:51	Ribosome
rpmE	511145.b3936	STRING clusters	CL:54	Ribosome
rpmE	511145.b3936	KEGG	eco03010	Ribosome
rpmE	511145.b3936	COMPARTMENTS	GOCC:0005622	Intracellular
rpmE	511145.b3936	COMPARTMENTS	GOCC:0005737	Cytoplasm
rpmE	511145.b3936	COMPARTMENTS	GOCC:0005829	Cytosol
rpmE	511145.b3936	COMPARTMENTS	GOCC:0005840	Ribosome
rpmE	511145.b3936	COMPARTMENTS	GOCC:0015934	Large ribosomal subunit
rpmE	511145.b3936	COMPARTMENTS	GOCC:0022625	Cytosolic large ribosomal subunit
rpmE	511145.b3936	COMPARTMENTS	GOCC:0022626	Cytosolic ribosome
rpmE	511145.b3936	COMPARTMENTS	GOCC:0032991	Protein-containing complex
rpmE	511145.b3936	COMPARTMENTS	GOCC:0043226	Organelle
rpmE	511145.b3936	COMPARTMENTS	GOCC:0043228	Non-membrane-bounded organelle
rpmE	511145.b3936	COMPARTMENTS	GOCC:0043229	Intracellular organelle
rpmE	511145.b3936	COMPARTMENTS	GOCC:0043232	Intracellular non-membrane-bounded organelle
rpmE	511145.b3936	COMPARTMENTS	GOCC:0044391	Ribosomal subunit
rpmE	511145.b3936	COMPARTMENTS	GOCC:0110165	Cellular anatomical entity
rpmE	511145.b3936	COMPARTMENTS	GOCC:1990904	Ribonucleoprotein complex
rpmE	511145.b3936	UniProt Keywords	KW-0007	Acetylation
rpmE	511145.b3936	UniProt Keywords	KW-0479	Metal-binding
rpmE	511145.b3936	UniProt Keywords	KW-0687	Ribonucleoprotein
rpmE	511145.b3936	UniProt Keywords	KW-0689	Ribosomal protein
rpmE	511145.b3936	UniProt Keywords	KW-0694	RNA-binding
rpmE	511145.b3936	UniProt Keywords	KW-0699	rRNA-binding
rpmE	511145.b3936	UniProt Keywords	KW-0862	Zinc
rpmE	511145.b3936	Pfam	PF01197	Ribosomal protein L31
rpmE	511145.b3936	InterPro	IPR002150	Ribosomal protein L31
rpmE	511145.b3936	InterPro	IPR027491	Ribosomal protein L31 type A
rpmE	511145.b3936	InterPro	IPR034704	L28p-like
rpmE	511145.b3936	InterPro	IPR042105	Ribosomal protein L31 superfamily
rppH	511145.b2830	GO Process	GO:0006139	Nucleobase-containing compound metabolic process
rppH	511145.b2830	GO Process	GO:0006396	RNA processing
rppH	511145.b2830	GO Process	GO:0006399	tRNA metabolic process
rppH	511145.b2830	GO Process	GO:0006401	RNA catabolic process
rppH	511145.b2830	GO Process	GO:0006402	mRNA catabolic process
rppH	511145.b2830	GO Process	GO:0006725	Cellular aromatic compound metabolic process
rppH	511145.b2830	GO Process	GO:0006807	Nitrogen compound metabolic process
rppH	511145.b2830	GO Process	GO:0008033	tRNA processing
rppH	511145.b2830	GO Process	GO:0008152	Metabolic process
rppH	511145.b2830	GO Process	GO:0009056	Catabolic process
rppH	511145.b2830	GO Process	GO:0009057	Macromolecule catabolic process
rppH	511145.b2830	GO Process	GO:0009892	Negative regulation of metabolic process
rppH	511145.b2830	GO Process	GO:0009893	Positive regulation of metabolic process
rppH	511145.b2830	GO Process	GO:0009894	Regulation of catabolic process
rppH	511145.b2830	GO Process	GO:0009896	Positive regulation of catabolic process
rppH	511145.b2830	GO Process	GO:0009987	Cellular process
rppH	511145.b2830	GO Process	GO:0010467	Gene expression
rppH	511145.b2830	GO Process	GO:0010468	Regulation of gene expression
rppH	511145.b2830	GO Process	GO:0010604	Positive regulation of macromolecule metabolic process
rppH	511145.b2830	GO Process	GO:0010605	Negative regulation of macromolecule metabolic process
rppH	511145.b2830	GO Process	GO:0010608	Post-transcriptional regulation of gene expression
rppH	511145.b2830	GO Process	GO:0010629	Negative regulation of gene expression
rppH	511145.b2830	GO Process	GO:0016070	RNA metabolic process
rppH	511145.b2830	GO Process	GO:0016071	mRNA metabolic process
rppH	511145.b2830	GO Process	GO:0019219	Regulation of nucleobase-containing compound metabolic process
rppH	511145.b2830	GO Process	GO:0019222	Regulation of metabolic process
rppH	511145.b2830	GO Process	GO:0019439	Aromatic compound catabolic process
rppH	511145.b2830	GO Process	GO:0031323	Regulation of cellular metabolic process
rppH	511145.b2830	GO Process	GO:0031325	Positive regulation of cellular metabolic process
rppH	511145.b2830	GO Process	GO:0031329	Regulation of cellular catabolic process
rppH	511145.b2830	GO Process	GO:0031331	Positive regulation of cellular catabolic process
rppH	511145.b2830	GO Process	GO:0034470	ncRNA processing
rppH	511145.b2830	GO Process	GO:0034641	Cellular nitrogen compound metabolic process
rppH	511145.b2830	GO Process	GO:0034655	Nucleobase-containing compound catabolic process
rppH	511145.b2830	GO Process	GO:0034660	ncRNA metabolic process
rppH	511145.b2830	GO Process	GO:0043170	Macromolecule metabolic process
rppH	511145.b2830	GO Process	GO:0043487	Regulation of RNA stability
rppH	511145.b2830	GO Process	GO:0044237	Cellular metabolic process
rppH	511145.b2830	GO Process	GO:0044238	Primary metabolic process
rppH	511145.b2830	GO Process	GO:0044248	Cellular catabolic process
rppH	511145.b2830	GO Process	GO:0044260	Cellular macromolecule metabolic process
rppH	511145.b2830	GO Process	GO:0044265	Cellular macromolecule catabolic process
rppH	511145.b2830	GO Process	GO:0044270	Cellular nitrogen compound catabolic process
rppH	511145.b2830	GO Process	GO:0045935	Positive regulation of nucleobase-containing compound metabolic process
rppH	511145.b2830	GO Process	GO:0046483	Heterocycle metabolic process
rppH	511145.b2830	GO Process	GO:0046700	Heterocycle catabolic process
rppH	511145.b2830	GO Process	GO:0048518	Positive regulation of biological process
rppH	511145.b2830	GO Process	GO:0048519	Negative regulation of biological process
rppH	511145.b2830	GO Process	GO:0048522	Positive regulation of cellular process
rppH	511145.b2830	GO Process	GO:0050779	RNA destabilization
rppH	511145.b2830	GO Process	GO:0050789	Regulation of biological process
rppH	511145.b2830	GO Process	GO:0050794	Regulation of cellular process
rppH	511145.b2830	GO Process	GO:0051171	Regulation of nitrogen compound metabolic process
rppH	511145.b2830	GO Process	GO:0051173	Positive regulation of nitrogen compound metabolic process
rppH	511145.b2830	GO Process	GO:0051252	Regulation of RNA metabolic process
rppH	511145.b2830	GO Process	GO:0051254	Positive regulation of RNA metabolic process
rppH	511145.b2830	GO Process	GO:0060255	Regulation of macromolecule metabolic process
rppH	511145.b2830	GO Process	GO:0065007	Biological regulation
rppH	511145.b2830	GO Process	GO:0065008	Regulation of biological quality
rppH	511145.b2830	GO Process	GO:0071704	Organic substance metabolic process
rppH	511145.b2830	GO Process	GO:0080090	Regulation of primary metabolic process
rppH	511145.b2830	GO Process	GO:0090304	Nucleic acid metabolic process
rppH	511145.b2830	GO Process	GO:0110154	RNA decapping
rppH	511145.b2830	GO Process	GO:0110155	NAD-cap decapping
rppH	511145.b2830	GO Process	GO:1901360	Organic cyclic compound metabolic process
rppH	511145.b2830	GO Process	GO:1901361	Organic cyclic compound catabolic process
rppH	511145.b2830	GO Process	GO:1901575	Organic substance catabolic process
rppH	511145.b2830	GO Function	GO:0000287	Magnesium ion binding
rppH	511145.b2830	GO Function	GO:0003824	Catalytic activity
rppH	511145.b2830	GO Function	GO:0005488	Binding
rppH	511145.b2830	GO Function	GO:0016462	Pyrophosphatase activity
rppH	511145.b2830	GO Function	GO:0016787	Hydrolase activity
rppH	511145.b2830	GO Function	GO:0016817	Hydrolase activity, acting on acid anhydrides
rppH	511145.b2830	GO Function	GO:0016818	Hydrolase activity, acting on acid anhydrides, in phosphorus-containing anhydrides
rppH	511145.b2830	GO Function	GO:0034353	RNA pyrophosphohydrolase activity
rppH	511145.b2830	GO Function	GO:0043167	Ion binding
rppH	511145.b2830	GO Function	GO:0043169	Cation binding
rppH	511145.b2830	GO Function	GO:0046872	Metal ion binding
rppH	511145.b2830	GO Function	GO:0110153	RNA NAD-cap (NMN-forming) hydrolase activity
rppH	511145.b2830	GO Function	GO:0140098	Catalytic activity, acting on RNA
rppH	511145.b2830	GO Function	GO:0140640	Catalytic activity, acting on a nucleic acid
rppH	511145.b2830	GO Component	GO:0005622	Intracellular anatomical structure
rppH	511145.b2830	GO Component	GO:0005737	Cytoplasm
rppH	511145.b2830	GO Component	GO:0110165	Cellular anatomical entity
rppH	511145.b2830	STRING clusters	CL:531	Mixed, incl. RNA helicase activity, and Exoribonuclease activity
rppH	511145.b2830	STRING clusters	CL:533	Exoribonuclease activity, and RNA degradation
rppH	511145.b2830	STRING clusters	CL:534	RNA degradation, and Nuclease inhibitor activity
rppH	511145.b2830	KEGG	eco03018	RNA degradation
rppH	511145.b2830	UniProt Keywords	KW-0378	Hydrolase
rppH	511145.b2830	UniProt Keywords	KW-0460	Magnesium
rppH	511145.b2830	UniProt Keywords	KW-0464	Manganese
rppH	511145.b2830	UniProt Keywords	KW-0819	tRNA processing
rppH	511145.b2830	UniProt Keywords	KW-0862	Zinc
rppH	511145.b2830	Pfam	PF00293	NUDIX domain
rppH	511145.b2830	InterPro	IPR000086	NUDIX hydrolase domain
rppH	511145.b2830	InterPro	IPR015797	NUDIX hydrolase-like domain superfamily
rppH	511145.b2830	InterPro	IPR020084	NUDIX hydrolase, conserved site
rppH	511145.b2830	InterPro	IPR020476	NUDIX hydrolase
rppH	511145.b2830	InterPro	IPR022927	RNA pyrophosphohydrolase RppH
rpsH	511145.b3306	GO Process	GO:0000028	Ribosomal small subunit assembly
rpsH	511145.b3306	GO Process	GO:0002181	Cytoplasmic translation
rpsH	511145.b3306	GO Process	GO:0006412	Translation
rpsH	511145.b3306	GO Process	GO:0006417	Regulation of translation
rpsH	511145.b3306	GO Process	GO:0006518	Peptide metabolic process
rpsH	511145.b3306	GO Process	GO:0006807	Nitrogen compound metabolic process
rpsH	511145.b3306	GO Process	GO:0006996	Organelle organization
rpsH	511145.b3306	GO Process	GO:0008152	Metabolic process
rpsH	511145.b3306	GO Process	GO:0009058	Biosynthetic process
rpsH	511145.b3306	GO Process	GO:0009059	Macromolecule biosynthetic process
rpsH	511145.b3306	GO Process	GO:0009889	Regulation of biosynthetic process
rpsH	511145.b3306	GO Process	GO:0009894	Regulation of catabolic process
rpsH	511145.b3306	GO Process	GO:0009987	Cellular process
rpsH	511145.b3306	GO Process	GO:0010467	Gene expression
rpsH	511145.b3306	GO Process	GO:0010468	Regulation of gene expression
rpsH	511145.b3306	GO Process	GO:0010556	Regulation of macromolecule biosynthetic process
rpsH	511145.b3306	GO Process	GO:0010608	Post-transcriptional regulation of gene expression
rpsH	511145.b3306	GO Process	GO:0016043	Cellular component organization
rpsH	511145.b3306	GO Process	GO:0019219	Regulation of nucleobase-containing compound metabolic process
rpsH	511145.b3306	GO Process	GO:0019222	Regulation of metabolic process
rpsH	511145.b3306	GO Process	GO:0019538	Protein metabolic process
rpsH	511145.b3306	GO Process	GO:0022607	Cellular component assembly
rpsH	511145.b3306	GO Process	GO:0022613	Ribonucleoprotein complex biogenesis
rpsH	511145.b3306	GO Process	GO:0022618	Ribonucleoprotein complex assembly
rpsH	511145.b3306	GO Process	GO:0031323	Regulation of cellular metabolic process
rpsH	511145.b3306	GO Process	GO:0031326	Regulation of cellular biosynthetic process
rpsH	511145.b3306	GO Process	GO:0031329	Regulation of cellular catabolic process
rpsH	511145.b3306	GO Process	GO:0034248	Regulation of cellular amide metabolic process
rpsH	511145.b3306	GO Process	GO:0034641	Cellular nitrogen compound metabolic process
rpsH	511145.b3306	GO Process	GO:0034645	Cellular macromolecule biosynthetic process
rpsH	511145.b3306	GO Process	GO:0042254	Ribosome biogenesis
rpsH	511145.b3306	GO Process	GO:0042255	Ribosome assembly
rpsH	511145.b3306	GO Process	GO:0042274	Ribosomal small subunit biogenesis
rpsH	511145.b3306	GO Process	GO:0043043	Peptide biosynthetic process
rpsH	511145.b3306	GO Process	GO:0043170	Macromolecule metabolic process
rpsH	511145.b3306	GO Process	GO:0043487	Regulation of RNA stability
rpsH	511145.b3306	GO Process	GO:0043488	Regulation of mRNA stability
rpsH	511145.b3306	GO Process	GO:0043603	Cellular amide metabolic process
rpsH	511145.b3306	GO Process	GO:0043604	Amide biosynthetic process
rpsH	511145.b3306	GO Process	GO:0043933	Protein-containing complex organization
rpsH	511145.b3306	GO Process	GO:0044085	Cellular component biogenesis
rpsH	511145.b3306	GO Process	GO:0044237	Cellular metabolic process
rpsH	511145.b3306	GO Process	GO:0044238	Primary metabolic process
rpsH	511145.b3306	GO Process	GO:0044249	Cellular biosynthetic process
rpsH	511145.b3306	GO Process	GO:0044260	Cellular macromolecule metabolic process
rpsH	511145.b3306	GO Process	GO:0044271	Cellular nitrogen compound biosynthetic process
rpsH	511145.b3306	GO Process	GO:0050789	Regulation of biological process
rpsH	511145.b3306	GO Process	GO:0050794	Regulation of cellular process
rpsH	511145.b3306	GO Process	GO:0051171	Regulation of nitrogen compound metabolic process
rpsH	511145.b3306	GO Process	GO:0051246	Regulation of protein metabolic process
rpsH	511145.b3306	GO Process	GO:0051252	Regulation of RNA metabolic process
rpsH	511145.b3306	GO Process	GO:0060255	Regulation of macromolecule metabolic process
rpsH	511145.b3306	GO Process	GO:0061013	Regulation of mRNA catabolic process
rpsH	511145.b3306	GO Process	GO:0065003	Protein-containing complex assembly
rpsH	511145.b3306	GO Process	GO:0065007	Biological regulation
rpsH	511145.b3306	GO Process	GO:0065008	Regulation of biological quality
rpsH	511145.b3306	GO Process	GO:0070925	Organelle assembly
rpsH	511145.b3306	GO Process	GO:0071704	Organic substance metabolic process
rpsH	511145.b3306	GO Process	GO:0071826	Ribonucleoprotein complex subunit organization
rpsH	511145.b3306	GO Process	GO:0071840	Cellular component organization or biogenesis
rpsH	511145.b3306	GO Process	GO:0080090	Regulation of primary metabolic process
rpsH	511145.b3306	GO Process	GO:0140694	Non-membrane-bounded organelle assembly
rpsH	511145.b3306	GO Process	GO:1901564	Organonitrogen compound metabolic process
rpsH	511145.b3306	GO Process	GO:1901566	Organonitrogen compound biosynthetic process
rpsH	511145.b3306	GO Process	GO:1901576	Organic substance biosynthetic process
rpsH	511145.b3306	GO Process	GO:1903311	Regulation of mRNA metabolic process
rpsH	511145.b3306	GO Process	GO:2000112	Regulation of cellular macromolecule biosynthetic process
rpsH	511145.b3306	GO Function	GO:0003676	Nucleic acid binding
rpsH	511145.b3306	GO Function	GO:0003723	RNA binding
rpsH	511145.b3306	GO Function	GO:0003735	Structural constituent of ribosome
rpsH	511145.b3306	GO Function	GO:0005198	Structural molecule activity
rpsH	511145.b3306	GO Function	GO:0005488	Binding
rpsH	511145.b3306	GO Function	GO:0019843	rRNA binding
rpsH	511145.b3306	GO Function	GO:0097159	Organic cyclic compound binding
rpsH	511145.b3306	GO Function	GO:1901363	Heterocyclic compound binding
rpsH	511145.b3306	GO Component	GO:0005622	Intracellular anatomical structure
rpsH	511145.b3306	GO Component	GO:0005737	Cytoplasm
rpsH	511145.b3306	GO Component	GO:0005829	Cytosol
rpsH	511145.b3306	GO Component	GO:0005840	Ribosome
rpsH	511145.b3306	GO Component	GO:0015935	Small ribosomal subunit
rpsH	511145.b3306	GO Component	GO:0022626	Cytosolic ribosome
rpsH	511145.b3306	GO Component	GO:0022627	Cytosolic small ribosomal subunit
rpsH	511145.b3306	GO Component	GO:0032991	Protein-containing complex
rpsH	511145.b3306	GO Component	GO:0043226	Organelle
rpsH	511145.b3306	GO Component	GO:0043228	Non-membrane-bounded organelle
rpsH	511145.b3306	GO Component	GO:0043229	Intracellular organelle
rpsH	511145.b3306	GO Component	GO:0043232	Intracellular non-membrane-bounded organelle
rpsH	511145.b3306	GO Component	GO:0044391	Ribosomal subunit
rpsH	511145.b3306	GO Component	GO:0110165	Cellular anatomical entity
rpsH	511145.b3306	GO Component	GO:1990904	Ribonucleoprotein complex
rpsH	511145.b3306	STRING clusters	CL:35	Translation, and Protein export
rpsH	511145.b3306	STRING clusters	CL:37	Translation, and Protein export
rpsH	511145.b3306	STRING clusters	CL:39	Mixed, incl. Ribonucleoprotein, and Translation regulator activity
rpsH	511145.b3306	STRING clusters	CL:40	Ribonucleoprotein, and Translation factor activity, RNA binding
rpsH	511145.b3306	STRING clusters	CL:41	Ribonucleoprotein, and Translation factor activity, RNA binding
rpsH	511145.b3306	STRING clusters	CL:42	Ribosome, and Translation factor activity, RNA binding
rpsH	511145.b3306	STRING clusters	CL:45	Ribosome, and Translation factor activity, RNA binding
rpsH	511145.b3306	STRING clusters	CL:47	Ribosome, and Translation factor activity, RNA binding
rpsH	511145.b3306	STRING clusters	CL:49	Ribosome
rpsH	511145.b3306	STRING clusters	CL:51	Ribosome
rpsH	511145.b3306	STRING clusters	CL:54	Ribosome
rpsH	511145.b3306	STRING clusters	CL:56	Ribosome
rpsH	511145.b3306	STRING clusters	CL:57	Small ribosomal subunit, and Rescue of stalled ribosome
rpsH	511145.b3306	KEGG	eco03010	Ribosome
rpsH	511145.b3306	COMPARTMENTS	GOCC:0005622	Intracellular
rpsH	511145.b3306	COMPARTMENTS	GOCC:0005737	Cytoplasm
rpsH	511145.b3306	COMPARTMENTS	GOCC:0005829	Cytosol
rpsH	511145.b3306	COMPARTMENTS	GOCC:0005840	Ribosome
rpsH	511145.b3306	COMPARTMENTS	GOCC:0015935	Small ribosomal subunit
rpsH	511145.b3306	COMPARTMENTS	GOCC:0022626	Cytosolic ribosome
rpsH	511145.b3306	COMPARTMENTS	GOCC:0022627	Cytosolic small ribosomal subunit
rpsH	511145.b3306	COMPARTMENTS	GOCC:0032991	Protein-containing complex
rpsH	511145.b3306	COMPARTMENTS	GOCC:0043226	Organelle
rpsH	511145.b3306	COMPARTMENTS	GOCC:0043228	Non-membrane-bounded organelle
rpsH	511145.b3306	COMPARTMENTS	GOCC:0043229	Intracellular organelle
rpsH	511145.b3306	COMPARTMENTS	GOCC:0043232	Intracellular non-membrane-bounded organelle
rpsH	511145.b3306	COMPARTMENTS	GOCC:0044391	Ribosomal subunit
rpsH	511145.b3306	COMPARTMENTS	GOCC:0110165	Cellular anatomical entity
rpsH	511145.b3306	COMPARTMENTS	GOCC:1990904	Ribonucleoprotein complex
rpsH	511145.b3306	UniProt Keywords	KW-0678	Repressor
rpsH	511145.b3306	UniProt Keywords	KW-0687	Ribonucleoprotein
rpsH	511145.b3306	UniProt Keywords	KW-0689	Ribosomal protein
rpsH	511145.b3306	UniProt Keywords	KW-0694	RNA-binding
rpsH	511145.b3306	UniProt Keywords	KW-0699	rRNA-binding
rpsH	511145.b3306	UniProt Keywords	KW-0810	Translation regulation
rpsH	511145.b3306	Pfam	PF00410	Ribosomal protein S8
rpsH	511145.b3306	InterPro	IPR000630	Ribosomal protein S8
rpsH	511145.b3306	InterPro	IPR035987	Ribosomal protein S8 superfamily
rpsI	511145.b3230	GO Process	GO:0002181	Cytoplasmic translation
rpsI	511145.b3230	GO Process	GO:0006412	Translation
rpsI	511145.b3230	GO Process	GO:0006518	Peptide metabolic process
rpsI	511145.b3230	GO Process	GO:0006807	Nitrogen compound metabolic process
rpsI	511145.b3230	GO Process	GO:0008152	Metabolic process
rpsI	511145.b3230	GO Process	GO:0009058	Biosynthetic process
rpsI	511145.b3230	GO Process	GO:0009059	Macromolecule biosynthetic process
rpsI	511145.b3230	GO Process	GO:0009987	Cellular process
rpsI	511145.b3230	GO Process	GO:0010467	Gene expression
rpsI	511145.b3230	GO Process	GO:0019538	Protein metabolic process
rpsI	511145.b3230	GO Process	GO:0034641	Cellular nitrogen compound metabolic process
rpsI	511145.b3230	GO Process	GO:0034645	Cellular macromolecule biosynthetic process
rpsI	511145.b3230	GO Process	GO:0043043	Peptide biosynthetic process
rpsI	511145.b3230	GO Process	GO:0043170	Macromolecule metabolic process
rpsI	511145.b3230	GO Process	GO:0043603	Cellular amide metabolic process
rpsI	511145.b3230	GO Process	GO:0043604	Amide biosynthetic process
rpsI	511145.b3230	GO Process	GO:0044237	Cellular metabolic process
rpsI	511145.b3230	GO Process	GO:0044238	Primary metabolic process
rpsI	511145.b3230	GO Process	GO:0044249	Cellular biosynthetic process
rpsI	511145.b3230	GO Process	GO:0044260	Cellular macromolecule metabolic process
rpsI	511145.b3230	GO Process	GO:0044271	Cellular nitrogen compound biosynthetic process
rpsI	511145.b3230	GO Process	GO:0071704	Organic substance metabolic process
rpsI	511145.b3230	GO Process	GO:1901564	Organonitrogen compound metabolic process
rpsI	511145.b3230	GO Process	GO:1901566	Organonitrogen compound biosynthetic process
rpsI	511145.b3230	GO Process	GO:1901576	Organic substance biosynthetic process
rpsI	511145.b3230	GO Function	GO:0000049	tRNA binding
rpsI	511145.b3230	GO Function	GO:0003676	Nucleic acid binding
rpsI	511145.b3230	GO Function	GO:0003723	RNA binding
rpsI	511145.b3230	GO Function	GO:0003735	Structural constituent of ribosome
rpsI	511145.b3230	GO Function	GO:0005198	Structural molecule activity
rpsI	511145.b3230	GO Function	GO:0005488	Binding
rpsI	511145.b3230	GO Function	GO:0097159	Organic cyclic compound binding
rpsI	511145.b3230	GO Function	GO:1901363	Heterocyclic compound binding
rpsI	511145.b3230	GO Component	GO:0005622	Intracellular anatomical structure
rpsI	511145.b3230	GO Component	GO:0005737	Cytoplasm
rpsI	511145.b3230	GO Component	GO:0005829	Cytosol
rpsI	511145.b3230	GO Component	GO:0005840	Ribosome
rpsI	511145.b3230	GO Component	GO:0015935	Small ribosomal subunit
rpsI	511145.b3230	GO Component	GO:0022626	Cytosolic ribosome
rpsI	511145.b3230	GO Component	GO:0022627	Cytosolic small ribosomal subunit
rpsI	511145.b3230	GO Component	GO:0032991	Protein-containing complex
rpsI	511145.b3230	GO Component	GO:0043226	Organelle
rpsI	511145.b3230	GO Component	GO:0043228	Non-membrane-bounded organelle
rpsI	511145.b3230	GO Component	GO:0043229	Intracellular organelle
rpsI	511145.b3230	GO Component	GO:0043232	Intracellular non-membrane-bounded organelle
rpsI	511145.b3230	GO Component	GO:0044391	Ribosomal subunit
rpsI	511145.b3230	GO Component	GO:0110165	Cellular anatomical entity
rpsI	511145.b3230	GO Component	GO:1990904	Ribonucleoprotein complex
rpsI	511145.b3230	STRING clusters	CL:35	Translation, and Protein export
rpsI	511145.b3230	STRING clusters	CL:37	Translation, and Protein export
rpsI	511145.b3230	STRING clusters	CL:39	Mixed, incl. Ribonucleoprotein, and Translation regulator activity
rpsI	511145.b3230	STRING clusters	CL:40	Ribonucleoprotein, and Translation factor activity, RNA binding
rpsI	511145.b3230	STRING clusters	CL:41	Ribonucleoprotein, and Translation factor activity, RNA binding
rpsI	511145.b3230	STRING clusters	CL:42	Ribosome, and Translation factor activity, RNA binding
rpsI	511145.b3230	STRING clusters	CL:45	Ribosome, and Translation factor activity, RNA binding
rpsI	511145.b3230	STRING clusters	CL:47	Ribosome, and Translation factor activity, RNA binding
rpsI	511145.b3230	STRING clusters	CL:49	Ribosome
rpsI	511145.b3230	STRING clusters	CL:51	Ribosome
rpsI	511145.b3230	STRING clusters	CL:54	Ribosome
rpsI	511145.b3230	STRING clusters	CL:56	Ribosome
rpsI	511145.b3230	STRING clusters	CL:57	Small ribosomal subunit, and Rescue of stalled ribosome
rpsI	511145.b3230	KEGG	eco03010	Ribosome
rpsI	511145.b3230	COMPARTMENTS	GOCC:0005622	Intracellular
rpsI	511145.b3230	COMPARTMENTS	GOCC:0005737	Cytoplasm
rpsI	511145.b3230	COMPARTMENTS	GOCC:0005829	Cytosol
rpsI	511145.b3230	COMPARTMENTS	GOCC:0005840	Ribosome
rpsI	511145.b3230	COMPARTMENTS	GOCC:0015935	Small ribosomal subunit
rpsI	511145.b3230	COMPARTMENTS	GOCC:0022626	Cytosolic ribosome
rpsI	511145.b3230	COMPARTMENTS	GOCC:0022627	Cytosolic small ribosomal subunit
rpsI	511145.b3230	COMPARTMENTS	GOCC:0032991	Protein-containing complex
rpsI	511145.b3230	COMPARTMENTS	GOCC:0043226	Organelle
rpsI	511145.b3230	COMPARTMENTS	GOCC:0043228	Non-membrane-bounded organelle
rpsI	511145.b3230	COMPARTMENTS	GOCC:0043229	Intracellular organelle
rpsI	511145.b3230	COMPARTMENTS	GOCC:0043232	Intracellular non-membrane-bounded organelle
rpsI	511145.b3230	COMPARTMENTS	GOCC:0044391	Ribosomal subunit
rpsI	511145.b3230	COMPARTMENTS	GOCC:0110165	Cellular anatomical entity
rpsI	511145.b3230	COMPARTMENTS	GOCC:1990904	Ribonucleoprotein complex
rpsI	511145.b3230	UniProt Keywords	KW-0687	Ribonucleoprotein
rpsI	511145.b3230	UniProt Keywords	KW-0689	Ribosomal protein
rpsI	511145.b3230	UniProt Keywords	KW-0694	RNA-binding
rpsI	511145.b3230	UniProt Keywords	KW-0820	tRNA-binding
rpsI	511145.b3230	Pfam	PF00380	Ribosomal protein S9/S16
rpsI	511145.b3230	InterPro	IPR000754	Ribosomal protein S9
rpsI	511145.b3230	InterPro	IPR014721	Ribosomal protein S5 domain 2-type fold, subgroup
rpsI	511145.b3230	InterPro	IPR020568	Ribosomal protein S5 domain 2-type fold
rpsI	511145.b3230	InterPro	IPR020574	Ribosomal protein S9, conserved site
rpsI	511145.b3230	InterPro	IPR023035	Ribosomal protein S9, bacterial/plastid
rpsR	511145.b4202	GO Process	GO:0002181	Cytoplasmic translation
rpsR	511145.b4202	GO Process	GO:0006412	Translation
rpsR	511145.b4202	GO Process	GO:0006518	Peptide metabolic process
rpsR	511145.b4202	GO Process	GO:0006807	Nitrogen compound metabolic process
rpsR	511145.b4202	GO Process	GO:0008152	Metabolic process
rpsR	511145.b4202	GO Process	GO:0009058	Biosynthetic process
rpsR	511145.b4202	GO Process	GO:0009059	Macromolecule biosynthetic process
rpsR	511145.b4202	GO Process	GO:0009987	Cellular process
rpsR	511145.b4202	GO Process	GO:0010467	Gene expression
rpsR	511145.b4202	GO Process	GO:0019538	Protein metabolic process
rpsR	511145.b4202	GO Process	GO:0034641	Cellular nitrogen compound metabolic process
rpsR	511145.b4202	GO Process	GO:0034645	Cellular macromolecule biosynthetic process
rpsR	511145.b4202	GO Process	GO:0043043	Peptide biosynthetic process
rpsR	511145.b4202	GO Process	GO:0043170	Macromolecule metabolic process
rpsR	511145.b4202	GO Process	GO:0043603	Cellular amide metabolic process
rpsR	511145.b4202	GO Process	GO:0043604	Amide biosynthetic process
rpsR	511145.b4202	GO Process	GO:0044237	Cellular metabolic process
rpsR	511145.b4202	GO Process	GO:0044238	Primary metabolic process
rpsR	511145.b4202	GO Process	GO:0044249	Cellular biosynthetic process
rpsR	511145.b4202	GO Process	GO:0044260	Cellular macromolecule metabolic process
rpsR	511145.b4202	GO Process	GO:0044271	Cellular nitrogen compound biosynthetic process
rpsR	511145.b4202	GO Process	GO:0071704	Organic substance metabolic process
rpsR	511145.b4202	GO Process	GO:1901564	Organonitrogen compound metabolic process
rpsR	511145.b4202	GO Process	GO:1901566	Organonitrogen compound biosynthetic process
rpsR	511145.b4202	GO Process	GO:1901576	Organic substance biosynthetic process
rpsR	511145.b4202	GO Function	GO:0003676	Nucleic acid binding
rpsR	511145.b4202	GO Function	GO:0003723	RNA binding
rpsR	511145.b4202	GO Function	GO:0003729	mRNA binding
rpsR	511145.b4202	GO Function	GO:0003735	Structural constituent of ribosome
rpsR	511145.b4202	GO Function	GO:0005198	Structural molecule activity
rpsR	511145.b4202	GO Function	GO:0005488	Binding
rpsR	511145.b4202	GO Function	GO:0019843	rRNA binding
rpsR	511145.b4202	GO Function	GO:0048027	mRNA 5-UTR binding
rpsR	511145.b4202	GO Function	GO:0070181	Small ribosomal subunit rRNA binding
rpsR	511145.b4202	GO Function	GO:0097159	Organic cyclic compound binding
rpsR	511145.b4202	GO Function	GO:1901363	Heterocyclic compound binding
rpsR	511145.b4202	GO Component	GO:0005622	Intracellular anatomical structure
rpsR	511145.b4202	GO Component	GO:0005737	Cytoplasm
rpsR	511145.b4202	GO Component	GO:0005829	Cytosol
rpsR	511145.b4202	GO Component	GO:0005840	Ribosome
rpsR	511145.b4202	GO Component	GO:0015935	Small ribosomal subunit
rpsR	511145.b4202	GO Component	GO:0022626	Cytosolic ribosome
rpsR	511145.b4202	GO Component	GO:0022627	Cytosolic small ribosomal subunit
rpsR	511145.b4202	GO Component	GO:0032991	Protein-containing complex
rpsR	511145.b4202	GO Component	GO:0043226	Organelle
rpsR	511145.b4202	GO Component	GO:0043228	Non-membrane-bounded organelle
rpsR	511145.b4202	GO Component	GO:0043229	Intracellular organelle
rpsR	511145.b4202	GO Component	GO:0043232	Intracellular non-membrane-bounded organelle
rpsR	511145.b4202	GO Component	GO:0044391	Ribosomal subunit
rpsR	511145.b4202	GO Component	GO:0110165	Cellular anatomical entity
rpsR	511145.b4202	GO Component	GO:1990904	Ribonucleoprotein complex
rpsR	511145.b4202	STRING clusters	CL:35	Translation, and Protein export
rpsR	511145.b4202	STRING clusters	CL:37	Translation, and Protein export
rpsR	511145.b4202	STRING clusters	CL:39	Mixed, incl. Ribonucleoprotein, and Translation regulator activity
rpsR	511145.b4202	STRING clusters	CL:40	Ribonucleoprotein, and Translation factor activity, RNA binding
rpsR	511145.b4202	STRING clusters	CL:41	Ribonucleoprotein, and Translation factor activity, RNA binding
rpsR	511145.b4202	STRING clusters	CL:42	Ribosome, and Translation factor activity, RNA binding
rpsR	511145.b4202	STRING clusters	CL:45	Ribosome, and Translation factor activity, RNA binding
rpsR	511145.b4202	STRING clusters	CL:47	Ribosome, and Translation factor activity, RNA binding
rpsR	511145.b4202	STRING clusters	CL:49	Ribosome
rpsR	511145.b4202	STRING clusters	CL:51	Ribosome
rpsR	511145.b4202	STRING clusters	CL:54	Ribosome
rpsR	511145.b4202	STRING clusters	CL:56	Ribosome
rpsR	511145.b4202	STRING clusters	CL:88	Mixed, incl. 5S rRNA binding, and RNA splicing
rpsR	511145.b4202	KEGG	eco03010	Ribosome
rpsR	511145.b4202	COMPARTMENTS	GOCC:0005622	Intracellular
rpsR	511145.b4202	COMPARTMENTS	GOCC:0005737	Cytoplasm
rpsR	511145.b4202	COMPARTMENTS	GOCC:0005829	Cytosol
rpsR	511145.b4202	COMPARTMENTS	GOCC:0005840	Ribosome
rpsR	511145.b4202	COMPARTMENTS	GOCC:0015935	Small ribosomal subunit
rpsR	511145.b4202	COMPARTMENTS	GOCC:0022626	Cytosolic ribosome
rpsR	511145.b4202	COMPARTMENTS	GOCC:0022627	Cytosolic small ribosomal subunit
rpsR	511145.b4202	COMPARTMENTS	GOCC:0032991	Protein-containing complex
rpsR	511145.b4202	COMPARTMENTS	GOCC:0043226	Organelle
rpsR	511145.b4202	COMPARTMENTS	GOCC:0043228	Non-membrane-bounded organelle
rpsR	511145.b4202	COMPARTMENTS	GOCC:0043229	Intracellular organelle
rpsR	511145.b4202	COMPARTMENTS	GOCC:0043232	Intracellular non-membrane-bounded organelle
rpsR	511145.b4202	COMPARTMENTS	GOCC:0044391	Ribosomal subunit
rpsR	511145.b4202	COMPARTMENTS	GOCC:0110165	Cellular anatomical entity
rpsR	511145.b4202	COMPARTMENTS	GOCC:1990904	Ribonucleoprotein complex
rpsR	511145.b4202	UniProt Keywords	KW-0007	Acetylation
rpsR	511145.b4202	UniProt Keywords	KW-0687	Ribonucleoprotein
rpsR	511145.b4202	UniProt Keywords	KW-0689	Ribosomal protein
rpsR	511145.b4202	UniProt Keywords	KW-0694	RNA-binding
rpsR	511145.b4202	UniProt Keywords	KW-0699	rRNA-binding
rpsR	511145.b4202	Pfam	PF01084	Ribosomal protein S18
rpsR	511145.b4202	InterPro	IPR001648	Ribosomal protein S18
rpsR	511145.b4202	InterPro	IPR018275	Ribosomal protein S18, conserved site
rpsR	511145.b4202	InterPro	IPR036870	Ribosomal protein S18 superfamily
rraA	511145.b3929	GO Process	GO:0009892	Negative regulation of metabolic process
rraA	511145.b3929	GO Process	GO:0009894	Regulation of catabolic process
rraA	511145.b3929	GO Process	GO:0009895	Negative regulation of catabolic process
rraA	511145.b3929	GO Process	GO:0010605	Negative regulation of macromolecule metabolic process
rraA	511145.b3929	GO Process	GO:0019219	Regulation of nucleobase-containing compound metabolic process
rraA	511145.b3929	GO Process	GO:0019222	Regulation of metabolic process
rraA	511145.b3929	GO Process	GO:0031323	Regulation of cellular metabolic process
rraA	511145.b3929	GO Process	GO:0031324	Negative regulation of cellular metabolic process
rraA	511145.b3929	GO Process	GO:0031329	Regulation of cellular catabolic process
rraA	511145.b3929	GO Process	GO:0031330	Negative regulation of cellular catabolic process
rraA	511145.b3929	GO Process	GO:0032069	Regulation of nuclease activity
rraA	511145.b3929	GO Process	GO:0032074	Negative regulation of nuclease activity
rraA	511145.b3929	GO Process	GO:0043086	Negative regulation of catalytic activity
rraA	511145.b3929	GO Process	GO:0044092	Negative regulation of molecular function
rraA	511145.b3929	GO Process	GO:0045934	Negative regulation of nucleobase-containing compound metabolic process
rraA	511145.b3929	GO Process	GO:0048519	Negative regulation of biological process
rraA	511145.b3929	GO Process	GO:0048523	Negative regulation of cellular process
rraA	511145.b3929	GO Process	GO:0050789	Regulation of biological process
rraA	511145.b3929	GO Process	GO:0050790	Regulation of catalytic activity
rraA	511145.b3929	GO Process	GO:0050794	Regulation of cellular process
rraA	511145.b3929	GO Process	GO:0051171	Regulation of nitrogen compound metabolic process
rraA	511145.b3929	GO Process	GO:0051172	Negative regulation of nitrogen compound metabolic process
rraA	511145.b3929	GO Process	GO:0051252	Regulation of RNA metabolic process
rraA	511145.b3929	GO Process	GO:0051253	Negative regulation of RNA metabolic process
rraA	511145.b3929	GO Process	GO:0051336	Regulation of hydrolase activity
rraA	511145.b3929	GO Process	GO:0051346	Negative regulation of hydrolase activity
rraA	511145.b3929	GO Process	GO:0060255	Regulation of macromolecule metabolic process
rraA	511145.b3929	GO Process	GO:0060699	Regulation of endoribonuclease activity
rraA	511145.b3929	GO Process	GO:0060700	Regulation of ribonuclease activity
rraA	511145.b3929	GO Process	GO:0060701	Negative regulation of ribonuclease activity
rraA	511145.b3929	GO Process	GO:0060702	Negative regulation of endoribonuclease activity
rraA	511145.b3929	GO Process	GO:0065007	Biological regulation
rraA	511145.b3929	GO Process	GO:0065009	Regulation of molecular function
rraA	511145.b3929	GO Process	GO:0080090	Regulation of primary metabolic process
rraA	511145.b3929	GO Process	GO:1902369	Negative regulation of RNA catabolic process
rraA	511145.b3929	GO Function	GO:0004857	Enzyme inhibitor activity
rraA	511145.b3929	GO Function	GO:0005488	Binding
rraA	511145.b3929	GO Function	GO:0005515	Protein binding
rraA	511145.b3929	GO Function	GO:0008428	Ribonuclease inhibitor activity
rraA	511145.b3929	GO Function	GO:0019899	Enzyme binding
rraA	511145.b3929	GO Function	GO:0030234	Enzyme regulator activity
rraA	511145.b3929	GO Function	GO:0060698	Endoribonuclease inhibitor activity
rraA	511145.b3929	GO Function	GO:0098772	Molecular function regulator activity
rraA	511145.b3929	GO Function	GO:0140721	Nuclease inhibitor activity
rraA	511145.b3929	GO Component	GO:0005622	Intracellular anatomical structure
rraA	511145.b3929	GO Component	GO:0005737	Cytoplasm
rraA	511145.b3929	GO Component	GO:0005829	Cytosol
rraA	511145.b3929	GO Component	GO:0032991	Protein-containing complex
rraA	511145.b3929	GO Component	GO:0110165	Cellular anatomical entity
rraA	511145.b3929	STRING clusters	CL:531	Mixed, incl. RNA helicase activity, and Exoribonuclease activity
rraA	511145.b3929	STRING clusters	CL:533	Exoribonuclease activity, and RNA degradation
rraA	511145.b3929	STRING clusters	CL:534	RNA degradation, and Nuclease inhibitor activity
rraA	511145.b3929	COMPARTMENTS	GOCC:0005622	Intracellular
rraA	511145.b3929	COMPARTMENTS	GOCC:0005737	Cytoplasm
rraA	511145.b3929	COMPARTMENTS	GOCC:0005829	Cytosol
rraA	511145.b3929	COMPARTMENTS	GOCC:0032991	Protein-containing complex
rraA	511145.b3929	COMPARTMENTS	GOCC:0110165	Cellular anatomical entity
rraA	511145.b3929	UniProt Keywords	KW-0963	Cytoplasm
rraA	511145.b3929	Pfam	PF03737	Aldolase/RraA
rraA	511145.b3929	InterPro	IPR005493	Ribonuclease E inhibitor RraA/RraA-like protein
rraA	511145.b3929	InterPro	IPR010203	Regulator of ribonuclease activity A
rraA	511145.b3929	InterPro	IPR014339	Regulator of ribonuclease activity A, gammaproteobacteria
rraA	511145.b3929	InterPro	IPR036704	Ribonuclease E inhibitor RraA/RraA-like superfamily
rsmF	511145.b1835	GO Process	GO:0000154	rRNA modification
rsmF	511145.b1835	GO Process	GO:0001510	RNA methylation
rsmF	511145.b1835	GO Process	GO:0006139	Nucleobase-containing compound metabolic process
rsmF	511145.b1835	GO Process	GO:0006364	rRNA processing
rsmF	511145.b1835	GO Process	GO:0006396	RNA processing
rsmF	511145.b1835	GO Process	GO:0006725	Cellular aromatic compound metabolic process
rsmF	511145.b1835	GO Process	GO:0006807	Nitrogen compound metabolic process
rsmF	511145.b1835	GO Process	GO:0008152	Metabolic process
rsmF	511145.b1835	GO Process	GO:0009451	RNA modification
rsmF	511145.b1835	GO Process	GO:0009987	Cellular process
rsmF	511145.b1835	GO Process	GO:0010467	Gene expression
rsmF	511145.b1835	GO Process	GO:0016070	RNA metabolic process
rsmF	511145.b1835	GO Process	GO:0016072	rRNA metabolic process
rsmF	511145.b1835	GO Process	GO:0022613	Ribonucleoprotein complex biogenesis
rsmF	511145.b1835	GO Process	GO:0031167	rRNA methylation
rsmF	511145.b1835	GO Process	GO:0032259	Methylation
rsmF	511145.b1835	GO Process	GO:0034470	ncRNA processing
rsmF	511145.b1835	GO Process	GO:0034641	Cellular nitrogen compound metabolic process
rsmF	511145.b1835	GO Process	GO:0034660	ncRNA metabolic process
rsmF	511145.b1835	GO Process	GO:0042254	Ribosome biogenesis
rsmF	511145.b1835	GO Process	GO:0043170	Macromolecule metabolic process
rsmF	511145.b1835	GO Process	GO:0043412	Macromolecule modification
rsmF	511145.b1835	GO Process	GO:0043414	Macromolecule methylation
rsmF	511145.b1835	GO Process	GO:0044085	Cellular component biogenesis
rsmF	511145.b1835	GO Process	GO:0044237	Cellular metabolic process
rsmF	511145.b1835	GO Process	GO:0044238	Primary metabolic process
rsmF	511145.b1835	GO Process	GO:0044260	Cellular macromolecule metabolic process
rsmF	511145.b1835	GO Process	GO:0046483	Heterocycle metabolic process
rsmF	511145.b1835	GO Process	GO:0070475	rRNA base methylation
rsmF	511145.b1835	GO Process	GO:0071704	Organic substance metabolic process
rsmF	511145.b1835	GO Process	GO:0071840	Cellular component organization or biogenesis
rsmF	511145.b1835	GO Process	GO:0090304	Nucleic acid metabolic process
rsmF	511145.b1835	GO Process	GO:1901360	Organic cyclic compound metabolic process
rsmF	511145.b1835	GO Function	GO:0003676	Nucleic acid binding
rsmF	511145.b1835	GO Function	GO:0003723	RNA binding
rsmF	511145.b1835	GO Function	GO:0003824	Catalytic activity
rsmF	511145.b1835	GO Function	GO:0005488	Binding
rsmF	511145.b1835	GO Function	GO:0008168	Methyltransferase activity
rsmF	511145.b1835	GO Function	GO:0008169	C-methyltransferase activity
rsmF	511145.b1835	GO Function	GO:0008173	RNA methyltransferase activity
rsmF	511145.b1835	GO Function	GO:0008649	rRNA methyltransferase activity
rsmF	511145.b1835	GO Function	GO:0008757	S-adenosylmethionine-dependent methyltransferase activity
rsmF	511145.b1835	GO Function	GO:0009383	rRNA (cytosine-C5-)-methyltransferase activity
rsmF	511145.b1835	GO Function	GO:0016434	rRNA (cytosine) methyltransferase activity
rsmF	511145.b1835	GO Function	GO:0016740	Transferase activity
rsmF	511145.b1835	GO Function	GO:0016741	Transferase activity, transferring one-carbon groups
rsmF	511145.b1835	GO Function	GO:0097159	Organic cyclic compound binding
rsmF	511145.b1835	GO Function	GO:0140098	Catalytic activity, acting on RNA
rsmF	511145.b1835	GO Function	GO:0140102	Catalytic activity, acting on a rRNA
rsmF	511145.b1835	GO Function	GO:0140640	Catalytic activity, acting on a nucleic acid
rsmF	511145.b1835	GO Function	GO:1901363	Heterocyclic compound binding
rsmF	511145.b1835	GO Component	GO:0005622	Intracellular anatomical structure
rsmF	511145.b1835	GO Component	GO:0005737	Cytoplasm
rsmF	511145.b1835	GO Component	GO:0110165	Cellular anatomical entity
rsmF	511145.b1835	STRING clusters	CL:378	Mixed, incl. rRNA modification, and tRNA methylation
rsmF	511145.b1835	STRING clusters	CL:379	rRNA modification, and tRNA methylation
rsmF	511145.b1835	STRING clusters	CL:380	RNA methylation, and GTP binding domain
rsmF	511145.b1835	STRING clusters	CL:381	RNA methylation
rsmF	511145.b1835	STRING clusters	CL:382	RNA methyltransferase activity
rsmF	511145.b1835	STRING clusters	CL:394	rRNA (cytosine) methyltransferase activity, and rRNA (uridine) methyltransferase activity
rsmF	511145.b1835	UniProt Keywords	KW-0489	Methyltransferase
rsmF	511145.b1835	UniProt Keywords	KW-0694	RNA-binding
rsmF	511145.b1835	UniProt Keywords	KW-0698	rRNA processing
rsmF	511145.b1835	UniProt Keywords	KW-0808	Transferase
rsmF	511145.b1835	UniProt Keywords	KW-0949	S-adenosyl-L-methionine
rsmF	511145.b1835	UniProt Keywords	KW-0963	Cytoplasm
rsmF	511145.b1835	Pfam	PF01189	16S rRNA methyltransferase RsmB/F
rsmF	511145.b1835	Pfam	PF13636	RNA-binding PUA-like domain of methyltransferase RsmF
rsmF	511145.b1835	Pfam	PF17125	N-terminal domain of 16S rRNA methyltransferase RsmF
rsmF	511145.b1835	InterPro	IPR001678	SAM-dependent methyltransferase RsmB/NOP2-type
rsmF	511145.b1835	InterPro	IPR011023	Nop2p
rsmF	511145.b1835	InterPro	IPR018314	Bacterial Fmu (Sun)/eukaryotic nucleolar NOL1/Nop2p, conserved site
rsmF	511145.b1835	InterPro	IPR023267	RNA (C5-cytosine) methyltransferase
rsmF	511145.b1835	InterPro	IPR023545	rRNA small subunit methyltransferase F
rsmF	511145.b1835	InterPro	IPR027391	rRNA small subunit methyltransferase F, RNA-binding PUA-like domain
rsmF	511145.b1835	InterPro	IPR029063	S-adenosyl-L-methionine-dependent methyltransferase superfamily
rsmF	511145.b1835	InterPro	IPR031341	Ribosomal RNA small subunit methyltransferase F, N-terminal
rsmG	511145.b3740	GO Process	GO:0000154	rRNA modification
rsmG	511145.b3740	GO Process	GO:0001510	RNA methylation
rsmG	511145.b3740	GO Process	GO:0006139	Nucleobase-containing compound metabolic process
rsmG	511145.b3740	GO Process	GO:0006364	rRNA processing
rsmG	511145.b3740	GO Process	GO:0006396	RNA processing
rsmG	511145.b3740	GO Process	GO:0006725	Cellular aromatic compound metabolic process
rsmG	511145.b3740	GO Process	GO:0006807	Nitrogen compound metabolic process
rsmG	511145.b3740	GO Process	GO:0008152	Metabolic process
rsmG	511145.b3740	GO Process	GO:0009451	RNA modification
rsmG	511145.b3740	GO Process	GO:0009987	Cellular process
rsmG	511145.b3740	GO Process	GO:0010467	Gene expression
rsmG	511145.b3740	GO Process	GO:0016070	RNA metabolic process
rsmG	511145.b3740	GO Process	GO:0016072	rRNA metabolic process
rsmG	511145.b3740	GO Process	GO:0022613	Ribonucleoprotein complex biogenesis
rsmG	511145.b3740	GO Process	GO:0031167	rRNA methylation
rsmG	511145.b3740	GO Process	GO:0032259	Methylation
rsmG	511145.b3740	GO Process	GO:0034470	ncRNA processing
rsmG	511145.b3740	GO Process	GO:0034641	Cellular nitrogen compound metabolic process
rsmG	511145.b3740	GO Process	GO:0034660	ncRNA metabolic process
rsmG	511145.b3740	GO Process	GO:0036265	RNA (guanine-N7)-methylation
rsmG	511145.b3740	GO Process	GO:0042254	Ribosome biogenesis
rsmG	511145.b3740	GO Process	GO:0043170	Macromolecule metabolic process
rsmG	511145.b3740	GO Process	GO:0043412	Macromolecule modification
rsmG	511145.b3740	GO Process	GO:0043414	Macromolecule methylation
rsmG	511145.b3740	GO Process	GO:0044085	Cellular component biogenesis
rsmG	511145.b3740	GO Process	GO:0044237	Cellular metabolic process
rsmG	511145.b3740	GO Process	GO:0044238	Primary metabolic process
rsmG	511145.b3740	GO Process	GO:0044260	Cellular macromolecule metabolic process
rsmG	511145.b3740	GO Process	GO:0046483	Heterocycle metabolic process
rsmG	511145.b3740	GO Process	GO:0070475	rRNA base methylation
rsmG	511145.b3740	GO Process	GO:0070476	rRNA (guanine-N7)-methylation
rsmG	511145.b3740	GO Process	GO:0071704	Organic substance metabolic process
rsmG	511145.b3740	GO Process	GO:0071840	Cellular component organization or biogenesis
rsmG	511145.b3740	GO Process	GO:0090304	Nucleic acid metabolic process
rsmG	511145.b3740	GO Process	GO:1901360	Organic cyclic compound metabolic process
rsmG	511145.b3740	GO Function	GO:0003824	Catalytic activity
rsmG	511145.b3740	GO Function	GO:0008168	Methyltransferase activity
rsmG	511145.b3740	GO Function	GO:0008170	N-methyltransferase activity
rsmG	511145.b3740	GO Function	GO:0008173	RNA methyltransferase activity
rsmG	511145.b3740	GO Function	GO:0008649	rRNA methyltransferase activity
rsmG	511145.b3740	GO Function	GO:0008757	S-adenosylmethionine-dependent methyltransferase activity
rsmG	511145.b3740	GO Function	GO:0016435	rRNA (guanine) methyltransferase activity
rsmG	511145.b3740	GO Function	GO:0016740	Transferase activity
rsmG	511145.b3740	GO Function	GO:0016741	Transferase activity, transferring one-carbon groups
rsmG	511145.b3740	GO Function	GO:0070043	rRNA (guanine-N7-)-methyltransferase activity
rsmG	511145.b3740	GO Function	GO:0140098	Catalytic activity, acting on RNA
rsmG	511145.b3740	GO Function	GO:0140102	Catalytic activity, acting on a rRNA
rsmG	511145.b3740	GO Function	GO:0140640	Catalytic activity, acting on a nucleic acid
rsmG	511145.b3740	GO Component	GO:0005622	Intracellular anatomical structure
rsmG	511145.b3740	GO Component	GO:0005737	Cytoplasm
rsmG	511145.b3740	GO Component	GO:0005829	Cytosol
rsmG	511145.b3740	GO Component	GO:0110165	Cellular anatomical entity
rsmG	511145.b3740	STRING clusters	CL:378	Mixed, incl. rRNA modification, and tRNA methylation
rsmG	511145.b3740	STRING clusters	CL:379	rRNA modification, and tRNA methylation
rsmG	511145.b3740	STRING clusters	CL:380	RNA methylation, and GTP binding domain
rsmG	511145.b3740	STRING clusters	CL:381	RNA methylation
rsmG	511145.b3740	STRING clusters	CL:405	Mixed, incl. Regulation of cytoplasmic translational fidelity, and tRNA 3-trailer cleavage
rsmG	511145.b3740	COMPARTMENTS	GOCC:0005622	Intracellular
rsmG	511145.b3740	COMPARTMENTS	GOCC:0005737	Cytoplasm
rsmG	511145.b3740	COMPARTMENTS	GOCC:0005829	Cytosol
rsmG	511145.b3740	COMPARTMENTS	GOCC:0110165	Cellular anatomical entity
rsmG	511145.b3740	UniProt Keywords	KW-0489	Methyltransferase
rsmG	511145.b3740	UniProt Keywords	KW-0698	rRNA processing
rsmG	511145.b3740	UniProt Keywords	KW-0808	Transferase
rsmG	511145.b3740	UniProt Keywords	KW-0949	S-adenosyl-L-methionine
rsmG	511145.b3740	UniProt Keywords	KW-0963	Cytoplasm
rsmG	511145.b3740	Pfam	PF02527	rRNA small subunit methyltransferase G
rsmG	511145.b3740	InterPro	IPR003682	rRNA small subunit methyltransferase G
rsmG	511145.b3740	InterPro	IPR029063	S-adenosyl-L-methionine-dependent methyltransferase superfamily
rsxB	511145.b1628	GO Process	GO:0006091	Generation of precursor metabolites and energy
rsxB	511145.b1628	GO Process	GO:0008152	Metabolic process
rsxB	511145.b1628	GO Process	GO:0009987	Cellular process
rsxB	511145.b1628	GO Process	GO:0022900	Electron transport chain
rsxB	511145.b1628	GO Process	GO:0044237	Cellular metabolic process
rsxB	511145.b1628	GO Function	GO:0003824	Catalytic activity
rsxB	511145.b1628	GO Function	GO:0005488	Binding
rsxB	511145.b1628	GO Function	GO:0009055	Electron transfer activity
rsxB	511145.b1628	GO Function	GO:0016491	Oxidoreductase activity
rsxB	511145.b1628	GO Function	GO:0043167	Ion binding
rsxB	511145.b1628	GO Function	GO:0043169	Cation binding
rsxB	511145.b1628	GO Function	GO:0046872	Metal ion binding
rsxB	511145.b1628	GO Function	GO:0051536	Iron-sulfur cluster binding
rsxB	511145.b1628	GO Function	GO:0051539	4 iron, 4 sulfur cluster binding
rsxB	511145.b1628	GO Function	GO:0051540	Metal cluster binding
rsxB	511145.b1628	GO Component	GO:0005886	Plasma membrane
rsxB	511145.b1628	GO Component	GO:0016020	Membrane
rsxB	511145.b1628	GO Component	GO:0071944	Cell periphery
rsxB	511145.b1628	GO Component	GO:0110165	Cellular anatomical entity
rsxB	511145.b1628	STRING clusters	CL:2782	Mixed, incl. Metal ion:proton antiporter activity, and NqrDE/RnfAE
rsxB	511145.b1628	STRING clusters	CL:2813	Mixed, incl. NqrDE/RnfAE, and Respiratory-chain NADH dehydrogenase 51 Kd subunit
rsxB	511145.b1628	UniProt Keywords	KW-0004	4Fe-4S
rsxB	511145.b1628	UniProt Keywords	KW-0249	Electron transport
rsxB	511145.b1628	UniProt Keywords	KW-0408	Iron
rsxB	511145.b1628	UniProt Keywords	KW-0411	Iron-sulfur
rsxB	511145.b1628	UniProt Keywords	KW-0472	Membrane
rsxB	511145.b1628	UniProt Keywords	KW-0479	Metal-binding
rsxB	511145.b1628	UniProt Keywords	KW-0677	Repeat
rsxB	511145.b1628	UniProt Keywords	KW-0813	Transport
rsxB	511145.b1628	UniProt Keywords	KW-0997	Cell inner membrane
rsxB	511145.b1628	UniProt Keywords	KW-1003	Cell membrane
rsxB	511145.b1628	UniProt Keywords	KW-1278	Translocase
rsxB	511145.b1628	Pfam	PF00037	4Fe-4S binding domain
rsxB	511145.b1628	Pfam	PF04060	Putative Fe-S cluster
rsxB	511145.b1628	InterPro	IPR007202	4Fe-4S domain
rsxB	511145.b1628	InterPro	IPR010207	Ion-translocating oxidoreductase complex, RnfB/RsxB
rsxB	511145.b1628	InterPro	IPR016463	Electron transport complex, RnfB/RsxB, Proteobacteria
rsxB	511145.b1628	InterPro	IPR017896	4Fe-4S ferredoxin-type, iron-sulphur binding domain
rsxB	511145.b1628	InterPro	IPR017900	4Fe-4S ferredoxin, iron-sulphur binding, conserved site
secF	511145.b0409	GO Process	GO:0006605	Protein targeting
secF	511145.b0409	GO Process	GO:0006810	Transport
secF	511145.b0409	GO Process	GO:0006886	Intracellular protein transport
secF	511145.b0409	GO Process	GO:0008104	Protein localization
secF	511145.b0409	GO Process	GO:0009987	Cellular process
secF	511145.b0409	GO Process	GO:0015031	Protein transport
secF	511145.b0409	GO Process	GO:0016043	Cellular component organization
secF	511145.b0409	GO Process	GO:0032978	Protein insertion into membrane from inner side
secF	511145.b0409	GO Process	GO:0033036	Macromolecule localization
secF	511145.b0409	GO Process	GO:0043952	Protein transport by the Sec complex
secF	511145.b0409	GO Process	GO:0045184	Establishment of protein localization
secF	511145.b0409	GO Process	GO:0046907	Intracellular transport
secF	511145.b0409	GO Process	GO:0051179	Localization
secF	511145.b0409	GO Process	GO:0051205	Protein insertion into membrane
secF	511145.b0409	GO Process	GO:0051234	Establishment of localization
secF	511145.b0409	GO Process	GO:0051641	Cellular localization
secF	511145.b0409	GO Process	GO:0051649	Establishment of localization in cell
secF	511145.b0409	GO Process	GO:0051668	Localization within membrane
secF	511145.b0409	GO Process	GO:0055085	Transmembrane transport
secF	511145.b0409	GO Process	GO:0061024	Membrane organization
secF	511145.b0409	GO Process	GO:0065002	Intracellular protein transmembrane transport
secF	511145.b0409	GO Process	GO:0070727	Cellular macromolecule localization
secF	511145.b0409	GO Process	GO:0071702	Organic substance transport
secF	511145.b0409	GO Process	GO:0071705	Nitrogen compound transport
secF	511145.b0409	GO Process	GO:0071806	Protein transmembrane transport
secF	511145.b0409	GO Process	GO:0071840	Cellular component organization or biogenesis
secF	511145.b0409	GO Process	GO:0072657	Protein localization to membrane
secF	511145.b0409	GO Process	GO:0090150	Establishment of protein localization to membrane
secF	511145.b0409	GO Function	GO:0005215	Transporter activity
secF	511145.b0409	GO Function	GO:0008320	Protein transmembrane transporter activity
secF	511145.b0409	GO Function	GO:0015399	Primary active transmembrane transporter activity
secF	511145.b0409	GO Function	GO:0015450	Protein-transporting ATPase activity
secF	511145.b0409	GO Function	GO:0022804	Active transmembrane transporter activity
secF	511145.b0409	GO Function	GO:0022857	Transmembrane transporter activity
secF	511145.b0409	GO Function	GO:0022884	Macromolecule transmembrane transporter activity
secF	511145.b0409	GO Function	GO:0042626	ATPase-coupled transmembrane transporter activity
secF	511145.b0409	GO Function	GO:0140318	Protein transporter activity
secF	511145.b0409	GO Function	GO:0140657	ATP-dependent activity
secF	511145.b0409	GO Component	GO:0005886	Plasma membrane
secF	511145.b0409	GO Component	GO:0016020	Membrane
secF	511145.b0409	GO Component	GO:0016021	Integral component of membrane
secF	511145.b0409	GO Component	GO:0031224	Intrinsic component of membrane
secF	511145.b0409	GO Component	GO:0031522	Cell envelope Sec protein transport complex
secF	511145.b0409	GO Component	GO:0032991	Protein-containing complex
secF	511145.b0409	GO Component	GO:0071944	Cell periphery
secF	511145.b0409	GO Component	GO:0110165	Cellular anatomical entity
secF	511145.b0409	STRING clusters	CL:221	Protein transport by the Sec complex, and SRP54-type protein, GTPase domain
secF	511145.b0409	STRING clusters	CL:223	Protein transport by the Sec complex
secF	511145.b0409	STRING clusters	CL:225	Cell envelope Sec protein transport complex
secF	511145.b0409	STRING clusters	CL:35	Translation, and Protein export
secF	511145.b0409	STRING clusters	CL:37	Translation, and Protein export
secF	511145.b0409	STRING clusters	CL:39	Mixed, incl. Ribonucleoprotein, and Translation regulator activity
secF	511145.b0409	STRING clusters	CL:40	Ribonucleoprotein, and Translation factor activity, RNA binding
secF	511145.b0409	STRING clusters	CL:41	Ribonucleoprotein, and Translation factor activity, RNA binding
secF	511145.b0409	KEGG	eco03060	Protein export
secF	511145.b0409	KEGG	eco03070	Bacterial secretion system
secF	511145.b0409	COMPARTMENTS	GOCC:0005886	Plasma membrane
secF	511145.b0409	COMPARTMENTS	GOCC:0016020	Membrane
secF	511145.b0409	COMPARTMENTS	GOCC:0071944	Cell periphery
secF	511145.b0409	COMPARTMENTS	GOCC:0110165	Cellular anatomical entity
secF	511145.b0409	UniProt Keywords	KW-0472	Membrane
secF	511145.b0409	UniProt Keywords	KW-0653	Protein transport
secF	511145.b0409	UniProt Keywords	KW-0811	Translocation
secF	511145.b0409	UniProt Keywords	KW-0812	Transmembrane
secF	511145.b0409	UniProt Keywords	KW-0813	Transport
secF	511145.b0409	UniProt Keywords	KW-0997	Cell inner membrane
secF	511145.b0409	UniProt Keywords	KW-1003	Cell membrane
secF	511145.b0409	UniProt Keywords	KW-1133	Transmembrane helix
secF	511145.b0409	Pfam	PF02355	Protein export membrane protein
secF	511145.b0409	Pfam	PF07549	SecD/SecF GG Motif
secF	511145.b0409	InterPro	IPR005665	Protein-export membrane protein SecF, bacterial
secF	511145.b0409	InterPro	IPR022645	Protein-export membrane protein SecD/SecF, bacterial
secF	511145.b0409	InterPro	IPR022646	Protein-export membrane protein SecD/SecF/SecDF, conserved site
secF	511145.b0409	InterPro	IPR022813	Protein-export membrane protein SecD/SecF, archaeal and bacterial
selD	511145.b1764	GO Process	GO:0001887	Selenium compound metabolic process
selD	511145.b1764	GO Process	GO:0006082	Organic acid metabolic process
selD	511145.b1764	GO Process	GO:0006139	Nucleobase-containing compound metabolic process
selD	511145.b1764	GO Process	GO:0006396	RNA processing
selD	511145.b1764	GO Process	GO:0006399	tRNA metabolic process
selD	511145.b1764	GO Process	GO:0006400	tRNA modification
selD	511145.b1764	GO Process	GO:0006520	Cellular amino acid metabolic process
selD	511145.b1764	GO Process	GO:0006725	Cellular aromatic compound metabolic process
selD	511145.b1764	GO Process	GO:0006793	Phosphorus metabolic process
selD	511145.b1764	GO Process	GO:0006796	Phosphate-containing compound metabolic process
selD	511145.b1764	GO Process	GO:0006807	Nitrogen compound metabolic process
selD	511145.b1764	GO Process	GO:0008033	tRNA processing
selD	511145.b1764	GO Process	GO:0008152	Metabolic process
selD	511145.b1764	GO Process	GO:0008652	Cellular amino acid biosynthetic process
selD	511145.b1764	GO Process	GO:0009058	Biosynthetic process
selD	511145.b1764	GO Process	GO:0009069	Serine family amino acid metabolic process
selD	511145.b1764	GO Process	GO:0009070	Serine family amino acid biosynthetic process
selD	511145.b1764	GO Process	GO:0009451	RNA modification
selD	511145.b1764	GO Process	GO:0009987	Cellular process
selD	511145.b1764	GO Process	GO:0010467	Gene expression
selD	511145.b1764	GO Process	GO:0016053	Organic acid biosynthetic process
selD	511145.b1764	GO Process	GO:0016070	RNA metabolic process
selD	511145.b1764	GO Process	GO:0016259	Selenocysteine metabolic process
selD	511145.b1764	GO Process	GO:0016260	Selenocysteine biosynthetic process
selD	511145.b1764	GO Process	GO:0016310	Phosphorylation
selD	511145.b1764	GO Process	GO:0019752	Carboxylic acid metabolic process
selD	511145.b1764	GO Process	GO:0034470	ncRNA processing
selD	511145.b1764	GO Process	GO:0034641	Cellular nitrogen compound metabolic process
selD	511145.b1764	GO Process	GO:0034660	ncRNA metabolic process
selD	511145.b1764	GO Process	GO:0043170	Macromolecule metabolic process
selD	511145.b1764	GO Process	GO:0043412	Macromolecule modification
selD	511145.b1764	GO Process	GO:0043436	Oxoacid metabolic process
selD	511145.b1764	GO Process	GO:0044237	Cellular metabolic process
selD	511145.b1764	GO Process	GO:0044238	Primary metabolic process
selD	511145.b1764	GO Process	GO:0044249	Cellular biosynthetic process
selD	511145.b1764	GO Process	GO:0044281	Small molecule metabolic process
selD	511145.b1764	GO Process	GO:0044283	Small molecule biosynthetic process
selD	511145.b1764	GO Process	GO:0046394	Carboxylic acid biosynthetic process
selD	511145.b1764	GO Process	GO:0046483	Heterocycle metabolic process
selD	511145.b1764	GO Process	GO:0070329	tRNA seleno-modification
selD	511145.b1764	GO Process	GO:0071704	Organic substance metabolic process
selD	511145.b1764	GO Process	GO:0090304	Nucleic acid metabolic process
selD	511145.b1764	GO Process	GO:1901360	Organic cyclic compound metabolic process
selD	511145.b1764	GO Process	GO:1901564	Organonitrogen compound metabolic process
selD	511145.b1764	GO Process	GO:1901566	Organonitrogen compound biosynthetic process
selD	511145.b1764	GO Process	GO:1901576	Organic substance biosynthetic process
selD	511145.b1764	GO Process	GO:1901605	Alpha-amino acid metabolic process
selD	511145.b1764	GO Process	GO:1901607	Alpha-amino acid biosynthetic process
selD	511145.b1764	GO Function	GO:0000166	Nucleotide binding
selD	511145.b1764	GO Function	GO:0000287	Magnesium ion binding
selD	511145.b1764	GO Function	GO:0003824	Catalytic activity
selD	511145.b1764	GO Function	GO:0004756	Selenide, water dikinase activity
selD	511145.b1764	GO Function	GO:0005488	Binding
selD	511145.b1764	GO Function	GO:0005515	Protein binding
selD	511145.b1764	GO Function	GO:0005524	ATP binding
selD	511145.b1764	GO Function	GO:0016301	Kinase activity
selD	511145.b1764	GO Function	GO:0016740	Transferase activity
selD	511145.b1764	GO Function	GO:0016772	Transferase activity, transferring phosphorus-containing groups
selD	511145.b1764	GO Function	GO:0016781	Phosphotransferase activity, paired acceptors
selD	511145.b1764	GO Function	GO:0017076	Purine nucleotide binding
selD	511145.b1764	GO Function	GO:0030554	Adenyl nucleotide binding
selD	511145.b1764	GO Function	GO:0032553	Ribonucleotide binding
selD	511145.b1764	GO Function	GO:0032555	Purine ribonucleotide binding
selD	511145.b1764	GO Function	GO:0032559	Adenyl ribonucleotide binding
selD	511145.b1764	GO Function	GO:0035639	Purine ribonucleoside triphosphate binding
selD	511145.b1764	GO Function	GO:0036094	Small molecule binding
selD	511145.b1764	GO Function	GO:0042802	Identical protein binding
selD	511145.b1764	GO Function	GO:0043167	Ion binding
selD	511145.b1764	GO Function	GO:0043168	Anion binding
selD	511145.b1764	GO Function	GO:0043169	Cation binding
selD	511145.b1764	GO Function	GO:0046872	Metal ion binding
selD	511145.b1764	GO Function	GO:0097159	Organic cyclic compound binding
selD	511145.b1764	GO Function	GO:0097367	Carbohydrate derivative binding
selD	511145.b1764	GO Function	GO:1901265	Nucleoside phosphate binding
selD	511145.b1764	GO Function	GO:1901363	Heterocyclic compound binding
selD	511145.b1764	GO Component	GO:0005622	Intracellular anatomical structure
selD	511145.b1764	GO Component	GO:0005737	Cytoplasm
selD	511145.b1764	GO Component	GO:0005829	Cytosol
selD	511145.b1764	GO Component	GO:0110165	Cellular anatomical entity
selD	511145.b1764	STRING clusters	CL:4810	Mixed, incl. Anaerobic respiration, and Molybdenum cofactor biosynthesis
selD	511145.b1764	STRING clusters	CL:4956	Anaerobic electron transport chain, and Protein maturation
selD	511145.b1764	STRING clusters	CL:4958	Formate oxidation, and Selenium
selD	511145.b1764	STRING clusters	CL:5004	Heme oxidation, and Selenium
selD	511145.b1764	KEGG	eco00450	Selenocompound metabolism
selD	511145.b1764	KEGG	eco01100	Metabolic pathways
selD	511145.b1764	COMPARTMENTS	GOCC:0005622	Intracellular
selD	511145.b1764	COMPARTMENTS	GOCC:0005737	Cytoplasm
selD	511145.b1764	COMPARTMENTS	GOCC:0005829	Cytosol
selD	511145.b1764	COMPARTMENTS	GOCC:0110165	Cellular anatomical entity
selD	511145.b1764	UniProt Keywords	KW-0067	ATP-binding
selD	511145.b1764	UniProt Keywords	KW-0418	Kinase
selD	511145.b1764	UniProt Keywords	KW-0460	Magnesium
selD	511145.b1764	UniProt Keywords	KW-0547	Nucleotide-binding
selD	511145.b1764	UniProt Keywords	KW-0711	Selenium
selD	511145.b1764	UniProt Keywords	KW-0808	Transferase
selD	511145.b1764	Pfam	PF00586	AIR synthase related protein, N-terminal domain
selD	511145.b1764	Pfam	PF02769	AIR synthase related protein, C-terminal domain
selD	511145.b1764	InterPro	IPR004536	Selenophosphate synthetase
selD	511145.b1764	InterPro	IPR010918	PurM-like, C-terminal domain
selD	511145.b1764	InterPro	IPR016188	PurM-like, N-terminal domain
selD	511145.b1764	InterPro	IPR023061	Selenophosphate synthetase, class I
selD	511145.b1764	InterPro	IPR036676	PurM-like, C-terminal domain superfamily
selD	511145.b1764	InterPro	IPR036921	PurM-like, N-terminal domain superfamily
sfsA	511145.b0146	GO Process	GO:0009889	Regulation of biosynthetic process
sfsA	511145.b0146	GO Process	GO:0009891	Positive regulation of biosynthetic process
sfsA	511145.b0146	GO Process	GO:0009893	Positive regulation of metabolic process
sfsA	511145.b0146	GO Process	GO:0019222	Regulation of metabolic process
sfsA	511145.b0146	GO Process	GO:0048518	Positive regulation of biological process
sfsA	511145.b0146	GO Process	GO:0050789	Regulation of biological process
sfsA	511145.b0146	GO Process	GO:0065007	Biological regulation
sfsA	511145.b0146	GO Function	GO:0003676	Nucleic acid binding
sfsA	511145.b0146	GO Function	GO:0003677	DNA binding
sfsA	511145.b0146	GO Function	GO:0005488	Binding
sfsA	511145.b0146	GO Function	GO:0097159	Organic cyclic compound binding
sfsA	511145.b0146	GO Function	GO:1901363	Heterocyclic compound binding
sfsA	511145.b0146	STRING clusters	CL:582	Mixed, incl. glutamate-tRNA ligase activity, and RNA ligase activity
sfsA	511145.b0146	UniProt Keywords	KW-0238	DNA-binding
sfsA	511145.b0146	Pfam	PF03749	Sugar fermentation stimulation protein RE domain
sfsA	511145.b0146	InterPro	IPR005224	Sugar fermentation stimulation protein
sfsA	511145.b0146	InterPro	IPR040452	Sugar fermentation stimulation protein, C-terminal
sfsA	511145.b0146	InterPro	IPR041465	SfsA, N-terminal OB domain
smpB	511145.b2620	GO Process	GO:0006412	Translation
smpB	511145.b2620	GO Process	GO:0006414	Translational elongation
smpB	511145.b2620	GO Process	GO:0006518	Peptide metabolic process
smpB	511145.b2620	GO Process	GO:0006807	Nitrogen compound metabolic process
smpB	511145.b2620	GO Process	GO:0008152	Metabolic process
smpB	511145.b2620	GO Process	GO:0009058	Biosynthetic process
smpB	511145.b2620	GO Process	GO:0009059	Macromolecule biosynthetic process
smpB	511145.b2620	GO Process	GO:0009987	Cellular process
smpB	511145.b2620	GO Process	GO:0010467	Gene expression
smpB	511145.b2620	GO Process	GO:0019538	Protein metabolic process
smpB	511145.b2620	GO Process	GO:0034641	Cellular nitrogen compound metabolic process
smpB	511145.b2620	GO Process	GO:0034645	Cellular macromolecule biosynthetic process
smpB	511145.b2620	GO Process	GO:0036211	Protein modification process
smpB	511145.b2620	GO Process	GO:0043043	Peptide biosynthetic process
smpB	511145.b2620	GO Process	GO:0043170	Macromolecule metabolic process
smpB	511145.b2620	GO Process	GO:0043412	Macromolecule modification
smpB	511145.b2620	GO Process	GO:0043603	Cellular amide metabolic process
smpB	511145.b2620	GO Process	GO:0043604	Amide biosynthetic process
smpB	511145.b2620	GO Process	GO:0044237	Cellular metabolic process
smpB	511145.b2620	GO Process	GO:0044238	Primary metabolic process
smpB	511145.b2620	GO Process	GO:0044249	Cellular biosynthetic process
smpB	511145.b2620	GO Process	GO:0044260	Cellular macromolecule metabolic process
smpB	511145.b2620	GO Process	GO:0044271	Cellular nitrogen compound biosynthetic process
smpB	511145.b2620	GO Process	GO:0070929	Trans-translation
smpB	511145.b2620	GO Process	GO:0070930	Trans-translation-dependent protein tagging
smpB	511145.b2620	GO Process	GO:0071704	Organic substance metabolic process
smpB	511145.b2620	GO Process	GO:1901564	Organonitrogen compound metabolic process
smpB	511145.b2620	GO Process	GO:1901566	Organonitrogen compound biosynthetic process
smpB	511145.b2620	GO Process	GO:1901576	Organic substance biosynthetic process
smpB	511145.b2620	GO Function	GO:0003676	Nucleic acid binding
smpB	511145.b2620	GO Function	GO:0003723	RNA binding
smpB	511145.b2620	GO Function	GO:0005488	Binding
smpB	511145.b2620	GO Function	GO:0097159	Organic cyclic compound binding
smpB	511145.b2620	GO Function	GO:1901363	Heterocyclic compound binding
smpB	511145.b2620	GO Component	GO:0005622	Intracellular anatomical structure
smpB	511145.b2620	GO Component	GO:0005737	Cytoplasm
smpB	511145.b2620	GO Component	GO:0005829	Cytosol
smpB	511145.b2620	GO Component	GO:0110165	Cellular anatomical entity
smpB	511145.b2620	STRING clusters	CL:35	Translation, and Protein export
smpB	511145.b2620	STRING clusters	CL:37	Translation, and Protein export
smpB	511145.b2620	STRING clusters	CL:39	Mixed, incl. Ribonucleoprotein, and Translation regulator activity
smpB	511145.b2620	STRING clusters	CL:40	Ribonucleoprotein, and Translation factor activity, RNA binding
smpB	511145.b2620	STRING clusters	CL:41	Ribonucleoprotein, and Translation factor activity, RNA binding
smpB	511145.b2620	STRING clusters	CL:42	Ribosome, and Translation factor activity, RNA binding
smpB	511145.b2620	COMPARTMENTS	GOCC:0005622	Intracellular
smpB	511145.b2620	COMPARTMENTS	GOCC:0005737	Cytoplasm
smpB	511145.b2620	COMPARTMENTS	GOCC:0005829	Cytosol
smpB	511145.b2620	COMPARTMENTS	GOCC:0110165	Cellular anatomical entity
smpB	511145.b2620	UniProt Keywords	KW-0694	RNA-binding
smpB	511145.b2620	UniProt Keywords	KW-0963	Cytoplasm
smpB	511145.b2620	Pfam	PF01668	SmpB protein
smpB	511145.b2620	InterPro	IPR000037	SsrA-binding protein
smpB	511145.b2620	InterPro	IPR020081	SsrA-binding protein, conserved site
smpB	511145.b2620	InterPro	IPR023620	Small protein B
speE	511145.b0121	GO Process	GO:0006576	Cellular biogenic amine metabolic process
speE	511145.b0121	GO Process	GO:0006595	Polyamine metabolic process
speE	511145.b0121	GO Process	GO:0006596	Polyamine biosynthetic process
speE	511145.b0121	GO Process	GO:0006807	Nitrogen compound metabolic process
speE	511145.b0121	GO Process	GO:0008152	Metabolic process
speE	511145.b0121	GO Process	GO:0008216	Spermidine metabolic process
speE	511145.b0121	GO Process	GO:0008295	Spermidine biosynthetic process
speE	511145.b0121	GO Process	GO:0009058	Biosynthetic process
speE	511145.b0121	GO Process	GO:0009308	Amine metabolic process
speE	511145.b0121	GO Process	GO:0009309	Amine biosynthetic process
speE	511145.b0121	GO Process	GO:0009987	Cellular process
speE	511145.b0121	GO Process	GO:0034641	Cellular nitrogen compound metabolic process
speE	511145.b0121	GO Process	GO:0042401	Cellular biogenic amine biosynthetic process
speE	511145.b0121	GO Process	GO:0044106	Cellular amine metabolic process
speE	511145.b0121	GO Process	GO:0044237	Cellular metabolic process
speE	511145.b0121	GO Process	GO:0044249	Cellular biosynthetic process
speE	511145.b0121	GO Process	GO:0044271	Cellular nitrogen compound biosynthetic process
speE	511145.b0121	GO Process	GO:0071704	Organic substance metabolic process
speE	511145.b0121	GO Process	GO:1901564	Organonitrogen compound metabolic process
speE	511145.b0121	GO Process	GO:1901566	Organonitrogen compound biosynthetic process
speE	511145.b0121	GO Process	GO:1901576	Organic substance biosynthetic process
speE	511145.b0121	GO Function	GO:0003824	Catalytic activity
speE	511145.b0121	GO Function	GO:0004766	Spermidine synthase activity
speE	511145.b0121	GO Function	GO:0010487	Thermospermine synthase activity
speE	511145.b0121	GO Function	GO:0016740	Transferase activity
speE	511145.b0121	GO Function	GO:0016765	Transferase activity, transferring alkyl or aryl (other than methyl) groups
speE	511145.b0121	GO Function	GO:0016768	Spermine synthase activity
speE	511145.b0121	GO Function	GO:0043918	Cadaverine aminopropyltransferase activity
speE	511145.b0121	GO Component	GO:0005622	Intracellular anatomical structure
speE	511145.b0121	GO Component	GO:0005737	Cytoplasm
speE	511145.b0121	GO Component	GO:0005829	Cytosol
speE	511145.b0121	GO Component	GO:0110165	Cellular anatomical entity
speE	511145.b0121	STRING clusters	CL:5950	Mixed, incl. Amine metabolic process, and Alcohol dehydrogenase-like, N-terminal
speE	511145.b0121	STRING clusters	CL:6087	Mixed, incl. Polyamine metabolic process, and Arginine catabolic process to succinate
speE	511145.b0121	STRING clusters	CL:6088	Polyamine metabolic process, and Arginine catabolic process to succinate
speE	511145.b0121	STRING clusters	CL:6089	Polyamine metabolic process, and Gamma-aminobutyric acid catabolic process
speE	511145.b0121	STRING clusters	CL:6124	Spermidine biosynthesis
speE	511145.b0121	KEGG	eco00270	Cysteine and methionine metabolism
speE	511145.b0121	KEGG	eco00330	Arginine and proline metabolism
speE	511145.b0121	KEGG	eco00480	Glutathione metabolism
speE	511145.b0121	KEGG	eco01100	Metabolic pathways
speE	511145.b0121	COMPARTMENTS	GOCC:0005622	Intracellular
speE	511145.b0121	COMPARTMENTS	GOCC:0005737	Cytoplasm
speE	511145.b0121	COMPARTMENTS	GOCC:0005829	Cytosol
speE	511145.b0121	COMPARTMENTS	GOCC:0110165	Cellular anatomical entity
speE	511145.b0121	UniProt Keywords	KW-0620	Polyamine biosynthesis
speE	511145.b0121	UniProt Keywords	KW-0745	Spermidine biosynthesis
speE	511145.b0121	UniProt Keywords	KW-0808	Transferase
speE	511145.b0121	UniProt Keywords	KW-0963	Cytoplasm
speE	511145.b0121	Pfam	PF01564	Spermine/spermidine synthase domain
speE	511145.b0121	Pfam	PF17284	Spermidine synthase tetramerisation domain
speE	511145.b0121	InterPro	IPR001045	Spermidine/spermine synthases
speE	511145.b0121	InterPro	IPR029063	S-adenosyl-L-methionine-dependent methyltransferase superfamily
speE	511145.b0121	InterPro	IPR030373	Polyamine biosynthesis domain, conserved site
speE	511145.b0121	InterPro	IPR030374	Polyamine biosynthesis domain
speE	511145.b0121	InterPro	IPR035246	Spermidine synthase, tetramerisation domain
speE	511145.b0121	InterPro	IPR037163	Spermidine synthase, tetramerisation domain superfamily
tam	511145.b1519	GO Process	GO:0008152	Metabolic process
tam	511145.b1519	GO Process	GO:0032259	Methylation
tam	511145.b1519	GO Function	GO:0003824	Catalytic activity
tam	511145.b1519	GO Function	GO:0008168	Methyltransferase activity
tam	511145.b1519	GO Function	GO:0008757	S-adenosylmethionine-dependent methyltransferase activity
tam	511145.b1519	GO Function	GO:0016740	Transferase activity
tam	511145.b1519	GO Function	GO:0016741	Transferase activity, transferring one-carbon groups
tam	511145.b1519	GO Function	GO:0030798	Trans-aconitate 2-methyltransferase activity
tam	511145.b1519	GO Component	GO:0005622	Intracellular anatomical structure
tam	511145.b1519	GO Component	GO:0005737	Cytoplasm
tam	511145.b1519	GO Component	GO:0005829	Cytosol
tam	511145.b1519	GO Component	GO:0110165	Cellular anatomical entity
tam	511145.b1519	STRING clusters	CL:4178	ATP-binding cassette (ABC) transporter complex
tam	511145.b1519	STRING clusters	CL:4179	ATP-binding cassette (ABC) transporter complex
tam	511145.b1519	STRING clusters	CL:4493	Mixed, incl. Quorum sensing, and Metabolite repair
tam	511145.b1519	COMPARTMENTS	GOCC:0005622	Intracellular
tam	511145.b1519	COMPARTMENTS	GOCC:0005737	Cytoplasm
tam	511145.b1519	COMPARTMENTS	GOCC:0005829	Cytosol
tam	511145.b1519	COMPARTMENTS	GOCC:0110165	Cellular anatomical entity
tam	511145.b1519	UniProt Keywords	KW-0489	Methyltransferase
tam	511145.b1519	UniProt Keywords	KW-0808	Transferase
tam	511145.b1519	UniProt Keywords	KW-0949	S-adenosyl-L-methionine
tam	511145.b1519	UniProt Keywords	KW-0963	Cytoplasm
tam	511145.b1519	Pfam	PF05175	Methyltransferase small domain
tam	511145.b1519	InterPro	IPR023149	Trans-aconitate 2-methyltransferase, C-terminal
tam	511145.b1519	InterPro	IPR023506	Trans-aconitate 2-methyltransferase
tam	511145.b1519	InterPro	IPR029063	S-adenosyl-L-methionine-dependent methyltransferase superfamily
tam	511145.b1519	InterPro	IPR041698	Methyltransferase domain 25
tatD	511145.b4483	GO Process	GO:0000302	Response to reactive oxygen species
tatD	511145.b4483	GO Process	GO:0000738	DNA catabolic process, exonucleolytic
tatD	511145.b4483	GO Process	GO:0006139	Nucleobase-containing compound metabolic process
tatD	511145.b4483	GO Process	GO:0006259	DNA metabolic process
tatD	511145.b4483	GO Process	GO:0006308	DNA catabolic process
tatD	511145.b4483	GO Process	GO:0006725	Cellular aromatic compound metabolic process
tatD	511145.b4483	GO Process	GO:0006807	Nitrogen compound metabolic process
tatD	511145.b4483	GO Process	GO:0006950	Response to stress
tatD	511145.b4483	GO Process	GO:0006979	Response to oxidative stress
tatD	511145.b4483	GO Process	GO:0008152	Metabolic process
tatD	511145.b4483	GO Process	GO:0009056	Catabolic process
tatD	511145.b4483	GO Process	GO:0009057	Macromolecule catabolic process
tatD	511145.b4483	GO Process	GO:0009987	Cellular process
tatD	511145.b4483	GO Process	GO:0010035	Response to inorganic substance
tatD	511145.b4483	GO Process	GO:0016070	RNA metabolic process
tatD	511145.b4483	GO Process	GO:0019439	Aromatic compound catabolic process
tatD	511145.b4483	GO Process	GO:0034641	Cellular nitrogen compound metabolic process
tatD	511145.b4483	GO Process	GO:0034655	Nucleobase-containing compound catabolic process
tatD	511145.b4483	GO Process	GO:0042221	Response to chemical
tatD	511145.b4483	GO Process	GO:0042542	Response to hydrogen peroxide
tatD	511145.b4483	GO Process	GO:0043170	Macromolecule metabolic process
tatD	511145.b4483	GO Process	GO:0044237	Cellular metabolic process
tatD	511145.b4483	GO Process	GO:0044238	Primary metabolic process
tatD	511145.b4483	GO Process	GO:0044248	Cellular catabolic process
tatD	511145.b4483	GO Process	GO:0044260	Cellular macromolecule metabolic process
tatD	511145.b4483	GO Process	GO:0044265	Cellular macromolecule catabolic process
tatD	511145.b4483	GO Process	GO:0044270	Cellular nitrogen compound catabolic process
tatD	511145.b4483	GO Process	GO:0046483	Heterocycle metabolic process
tatD	511145.b4483	GO Process	GO:0046700	Heterocycle catabolic process
tatD	511145.b4483	GO Process	GO:0050896	Response to stimulus
tatD	511145.b4483	GO Process	GO:0071704	Organic substance metabolic process
tatD	511145.b4483	GO Process	GO:0090304	Nucleic acid metabolic process
tatD	511145.b4483	GO Process	GO:0090305	Nucleic acid phosphodiester bond hydrolysis
tatD	511145.b4483	GO Process	GO:0090501	RNA phosphodiester bond hydrolysis
tatD	511145.b4483	GO Process	GO:0090503	RNA phosphodiester bond hydrolysis, exonucleolytic
tatD	511145.b4483	GO Process	GO:1901360	Organic cyclic compound metabolic process
tatD	511145.b4483	GO Process	GO:1901361	Organic cyclic compound catabolic process
tatD	511145.b4483	GO Process	GO:1901575	Organic substance catabolic process
tatD	511145.b4483	GO Process	GO:1901700	Response to oxygen-containing compound
tatD	511145.b4483	GO Function	GO:0000175	3-5-exoribonuclease activity
tatD	511145.b4483	GO Function	GO:0000287	Magnesium ion binding
tatD	511145.b4483	GO Function	GO:0003824	Catalytic activity
tatD	511145.b4483	GO Function	GO:0004518	Nuclease activity
tatD	511145.b4483	GO Function	GO:0004527	Exonuclease activity
tatD	511145.b4483	GO Function	GO:0004529	Exodeoxyribonuclease activity
tatD	511145.b4483	GO Function	GO:0004532	Exoribonuclease activity
tatD	511145.b4483	GO Function	GO:0004536	Deoxyribonuclease activity
tatD	511145.b4483	GO Function	GO:0004540	Ribonuclease activity
tatD	511145.b4483	GO Function	GO:0005488	Binding
tatD	511145.b4483	GO Function	GO:0008296	3-5-exodeoxyribonuclease activity
tatD	511145.b4483	GO Function	GO:0008297	Single-stranded DNA exodeoxyribonuclease activity
tatD	511145.b4483	GO Function	GO:0008310	Single-stranded DNA 3-5 exodeoxyribonuclease activity
tatD	511145.b4483	GO Function	GO:0008408	3-5 exonuclease activity
tatD	511145.b4483	GO Function	GO:0016787	Hydrolase activity
tatD	511145.b4483	GO Function	GO:0016788	Hydrolase activity, acting on ester bonds
tatD	511145.b4483	GO Function	GO:0016796	Exonuclease activity, active with either ribo- or deoxyribonucleic acids and producing 5-phosphomonoesters
tatD	511145.b4483	GO Function	GO:0016895	Exodeoxyribonuclease activity, producing 5-phosphomonoesters
tatD	511145.b4483	GO Function	GO:0016896	Exoribonuclease activity, producing 5-phosphomonoesters
tatD	511145.b4483	GO Function	GO:0043167	Ion binding
tatD	511145.b4483	GO Function	GO:0043169	Cation binding
tatD	511145.b4483	GO Function	GO:0046872	Metal ion binding
tatD	511145.b4483	GO Function	GO:0140097	Catalytic activity, acting on DNA
tatD	511145.b4483	GO Function	GO:0140098	Catalytic activity, acting on RNA
tatD	511145.b4483	GO Function	GO:0140640	Catalytic activity, acting on a nucleic acid
tatD	511145.b4483	GO Component	GO:0005622	Intracellular anatomical structure
tatD	511145.b4483	GO Component	GO:0005737	Cytoplasm
tatD	511145.b4483	GO Component	GO:0005829	Cytosol
tatD	511145.b4483	GO Component	GO:0110165	Cellular anatomical entity
tatD	511145.b4483	STRING clusters	CL:2534	Mixed, incl. Quinone metabolic process, and Tetrapyrrole biosynthetic process
tatD	511145.b4483	STRING clusters	CL:2535	Ubiquinone metabolic process, and Tetrapyrrole biosynthetic process
tatD	511145.b4483	STRING clusters	CL:2536	Ubiquinone metabolic process, and TAT protein transport complex
tatD	511145.b4483	STRING clusters	CL:2537	Ubiquinone metabolic process, and TAT protein transport complex
tatD	511145.b4483	STRING clusters	CL:2571	TAT protein transport complex, and Multicopper oxidase
tatD	511145.b4483	COMPARTMENTS	GOCC:0005622	Intracellular
tatD	511145.b4483	COMPARTMENTS	GOCC:0005737	Cytoplasm
tatD	511145.b4483	COMPARTMENTS	GOCC:0005829	Cytosol
tatD	511145.b4483	COMPARTMENTS	GOCC:0110165	Cellular anatomical entity
tatD	511145.b4483	UniProt Keywords	KW-0269	Exonuclease
tatD	511145.b4483	UniProt Keywords	KW-0378	Hydrolase
tatD	511145.b4483	UniProt Keywords	KW-0460	Magnesium
tatD	511145.b4483	UniProt Keywords	KW-0464	Manganese
tatD	511145.b4483	UniProt Keywords	KW-0479	Metal-binding
tatD	511145.b4483	UniProt Keywords	KW-0540	Nuclease
tatD	511145.b4483	UniProt Keywords	KW-0963	Cytoplasm
tatD	511145.b4483	Pfam	PF01026	TatD related DNase
tatD	511145.b4483	InterPro	IPR001130	3-5 ssDNA/RNA exonuclease TatD-like
tatD	511145.b4483	InterPro	IPR018228	Deoxyribonuclease, TatD-related, conserved site
tatD	511145.b4483	InterPro	IPR024918	3-5 ssDNA/RNA exonuclease TatD
tatD	511145.b4483	InterPro	IPR032466	Metal-dependent hydrolase
thiC	511145.b3994	GO Process	GO:0006066	Alcohol metabolic process
thiC	511145.b3994	GO Process	GO:0006725	Cellular aromatic compound metabolic process
thiC	511145.b3994	GO Process	GO:0006766	Vitamin metabolic process
thiC	511145.b3994	GO Process	GO:0006767	Water-soluble vitamin metabolic process
thiC	511145.b3994	GO Process	GO:0006772	Thiamine metabolic process
thiC	511145.b3994	GO Process	GO:0006790	Sulfur compound metabolic process
thiC	511145.b3994	GO Process	GO:0006793	Phosphorus metabolic process
thiC	511145.b3994	GO Process	GO:0006796	Phosphate-containing compound metabolic process
thiC	511145.b3994	GO Process	GO:0006807	Nitrogen compound metabolic process
thiC	511145.b3994	GO Process	GO:0008152	Metabolic process
thiC	511145.b3994	GO Process	GO:0009058	Biosynthetic process
thiC	511145.b3994	GO Process	GO:0009110	Vitamin biosynthetic process
thiC	511145.b3994	GO Process	GO:0009228	Thiamine biosynthetic process
thiC	511145.b3994	GO Process	GO:0009229	Thiamine diphosphate biosynthetic process
thiC	511145.b3994	GO Process	GO:0009987	Cellular process
thiC	511145.b3994	GO Process	GO:0018130	Heterocycle biosynthetic process
thiC	511145.b3994	GO Process	GO:0019438	Aromatic compound biosynthetic process
thiC	511145.b3994	GO Process	GO:0019637	Organophosphate metabolic process
thiC	511145.b3994	GO Process	GO:0034308	Primary alcohol metabolic process
thiC	511145.b3994	GO Process	GO:0034309	Primary alcohol biosynthetic process
thiC	511145.b3994	GO Process	GO:0034641	Cellular nitrogen compound metabolic process
thiC	511145.b3994	GO Process	GO:0042357	Thiamine diphosphate metabolic process
thiC	511145.b3994	GO Process	GO:0042364	Water-soluble vitamin biosynthetic process
thiC	511145.b3994	GO Process	GO:0042723	Thiamine-containing compound metabolic process
thiC	511145.b3994	GO Process	GO:0042724	Thiamine-containing compound biosynthetic process
thiC	511145.b3994	GO Process	GO:0044237	Cellular metabolic process
thiC	511145.b3994	GO Process	GO:0044249	Cellular biosynthetic process
thiC	511145.b3994	GO Process	GO:0044271	Cellular nitrogen compound biosynthetic process
thiC	511145.b3994	GO Process	GO:0044272	Sulfur compound biosynthetic process
thiC	511145.b3994	GO Process	GO:0044281	Small molecule metabolic process
thiC	511145.b3994	GO Process	GO:0044283	Small molecule biosynthetic process
thiC	511145.b3994	GO Process	GO:0046165	Alcohol biosynthetic process
thiC	511145.b3994	GO Process	GO:0046483	Heterocycle metabolic process
thiC	511145.b3994	GO Process	GO:0071704	Organic substance metabolic process
thiC	511145.b3994	GO Process	GO:0072527	Pyrimidine-containing compound metabolic process
thiC	511145.b3994	GO Process	GO:0072528	Pyrimidine-containing compound biosynthetic process
thiC	511145.b3994	GO Process	GO:0090407	Organophosphate biosynthetic process
thiC	511145.b3994	GO Process	GO:1901360	Organic cyclic compound metabolic process
thiC	511145.b3994	GO Process	GO:1901362	Organic cyclic compound biosynthetic process
thiC	511145.b3994	GO Process	GO:1901564	Organonitrogen compound metabolic process
thiC	511145.b3994	GO Process	GO:1901566	Organonitrogen compound biosynthetic process
thiC	511145.b3994	GO Process	GO:1901576	Organic substance biosynthetic process
thiC	511145.b3994	GO Process	GO:1901615	Organic hydroxy compound metabolic process
thiC	511145.b3994	GO Process	GO:1901617	Organic hydroxy compound biosynthetic process
thiC	511145.b3994	GO Function	GO:0003824	Catalytic activity
thiC	511145.b3994	GO Function	GO:0005488	Binding
thiC	511145.b3994	GO Function	GO:0008270	Zinc ion binding
thiC	511145.b3994	GO Function	GO:0016829	Lyase activity
thiC	511145.b3994	GO Function	GO:0016830	Carbon-carbon lyase activity
thiC	511145.b3994	GO Function	GO:0043167	Ion binding
thiC	511145.b3994	GO Function	GO:0043169	Cation binding
thiC	511145.b3994	GO Function	GO:0046872	Metal ion binding
thiC	511145.b3994	GO Function	GO:0046914	Transition metal ion binding
thiC	511145.b3994	GO Function	GO:0051536	Iron-sulfur cluster binding
thiC	511145.b3994	GO Function	GO:0051539	4 iron, 4 sulfur cluster binding
thiC	511145.b3994	GO Function	GO:0051540	Metal cluster binding
thiC	511145.b3994	GO Component	GO:0005622	Intracellular anatomical structure
thiC	511145.b3994	GO Component	GO:0005737	Cytoplasm
thiC	511145.b3994	GO Component	GO:0005829	Cytosol
thiC	511145.b3994	GO Component	GO:0110165	Cellular anatomical entity
thiC	511145.b3994	STRING clusters	CL:1869	Water-soluble vitamin biosynthetic process, and Nicotinate and nicotinamide metabolism
thiC	511145.b3994	STRING clusters	CL:1870	Water-soluble vitamin biosynthetic process, and Coenzyme A biosynthetic process
thiC	511145.b3994	STRING clusters	CL:1871	Water-soluble vitamin biosynthetic process
thiC	511145.b3994	STRING clusters	CL:1872	Water-soluble vitamin biosynthetic process
thiC	511145.b3994	STRING clusters	CL:1873	Thiamine diphosphate metabolic process, and Biotin biosynthesis
thiC	511145.b3994	STRING clusters	CL:1874	Thiamine diphosphate metabolic process
thiC	511145.b3994	STRING clusters	CL:1876	Thiamine biosynthesis
thiC	511145.b3994	KEGG	eco00730	Thiamine metabolism
thiC	511145.b3994	KEGG	eco01100	Metabolic pathways
thiC	511145.b3994	COMPARTMENTS	GOCC:0005622	Intracellular
thiC	511145.b3994	COMPARTMENTS	GOCC:0005737	Cytoplasm
thiC	511145.b3994	COMPARTMENTS	GOCC:0005829	Cytosol
thiC	511145.b3994	COMPARTMENTS	GOCC:0110165	Cellular anatomical entity
thiC	511145.b3994	UniProt Keywords	KW-0004	4Fe-4S
thiC	511145.b3994	UniProt Keywords	KW-0408	Iron
thiC	511145.b3994	UniProt Keywords	KW-0411	Iron-sulfur
thiC	511145.b3994	UniProt Keywords	KW-0456	Lyase
thiC	511145.b3994	UniProt Keywords	KW-0479	Metal-binding
thiC	511145.b3994	UniProt Keywords	KW-0784	Thiamine biosynthesis
thiC	511145.b3994	UniProt Keywords	KW-0949	S-adenosyl-L-methionine
thiC	511145.b3994	Pfam	PF01964	Radical SAM ThiC family
thiC	511145.b3994	Pfam	PF13667	ThiC-associated domain
thiC	511145.b3994	InterPro	IPR002817	Phosphomethylpyrimidine synthase ThiC/5-hydroxybenzimidazole synthase BzaA/B
thiC	511145.b3994	InterPro	IPR025747	ThiC-associated domain
thiC	511145.b3994	InterPro	IPR037509	Phosphomethylpyrimidine synthase
thiC	511145.b3994	InterPro	IPR038521	ThiC/Bza, core domain
thrS	511145.b1719	GO Process	GO:0006082	Organic acid metabolic process
thrS	511145.b1719	GO Process	GO:0006139	Nucleobase-containing compound metabolic process
thrS	511145.b1719	GO Process	GO:0006399	tRNA metabolic process
thrS	511145.b1719	GO Process	GO:0006412	Translation
thrS	511145.b1719	GO Process	GO:0006417	Regulation of translation
thrS	511145.b1719	GO Process	GO:0006418	tRNA aminoacylation for protein translation
thrS	511145.b1719	GO Process	GO:0006435	threonyl-tRNA aminoacylation
thrS	511145.b1719	GO Process	GO:0006446	Regulation of translational initiation
thrS	511145.b1719	GO Process	GO:0006450	Regulation of translational fidelity
thrS	511145.b1719	GO Process	GO:0006518	Peptide metabolic process
thrS	511145.b1719	GO Process	GO:0006520	Cellular amino acid metabolic process
thrS	511145.b1719	GO Process	GO:0006725	Cellular aromatic compound metabolic process
thrS	511145.b1719	GO Process	GO:0006807	Nitrogen compound metabolic process
thrS	511145.b1719	GO Process	GO:0008152	Metabolic process
thrS	511145.b1719	GO Process	GO:0009058	Biosynthetic process
thrS	511145.b1719	GO Process	GO:0009059	Macromolecule biosynthetic process
thrS	511145.b1719	GO Process	GO:0009889	Regulation of biosynthetic process
thrS	511145.b1719	GO Process	GO:0009890	Negative regulation of biosynthetic process
thrS	511145.b1719	GO Process	GO:0009892	Negative regulation of metabolic process
thrS	511145.b1719	GO Process	GO:0009987	Cellular process
thrS	511145.b1719	GO Process	GO:0010467	Gene expression
thrS	511145.b1719	GO Process	GO:0010468	Regulation of gene expression
thrS	511145.b1719	GO Process	GO:0010556	Regulation of macromolecule biosynthetic process
thrS	511145.b1719	GO Process	GO:0010558	Negative regulation of macromolecule biosynthetic process
thrS	511145.b1719	GO Process	GO:0010605	Negative regulation of macromolecule metabolic process
thrS	511145.b1719	GO Process	GO:0010608	Post-transcriptional regulation of gene expression
thrS	511145.b1719	GO Process	GO:0010629	Negative regulation of gene expression
thrS	511145.b1719	GO Process	GO:0016070	RNA metabolic process
thrS	511145.b1719	GO Process	GO:0017148	Negative regulation of translation
thrS	511145.b1719	GO Process	GO:0019222	Regulation of metabolic process
thrS	511145.b1719	GO Process	GO:0019538	Protein metabolic process
thrS	511145.b1719	GO Process	GO:0019752	Carboxylic acid metabolic process
thrS	511145.b1719	GO Process	GO:0031323	Regulation of cellular metabolic process
thrS	511145.b1719	GO Process	GO:0031324	Negative regulation of cellular metabolic process
thrS	511145.b1719	GO Process	GO:0031326	Regulation of cellular biosynthetic process
thrS	511145.b1719	GO Process	GO:0031327	Negative regulation of cellular biosynthetic process
thrS	511145.b1719	GO Process	GO:0034248	Regulation of cellular amide metabolic process
thrS	511145.b1719	GO Process	GO:0034249	Negative regulation of cellular amide metabolic process
thrS	511145.b1719	GO Process	GO:0034641	Cellular nitrogen compound metabolic process
thrS	511145.b1719	GO Process	GO:0034645	Cellular macromolecule biosynthetic process
thrS	511145.b1719	GO Process	GO:0034660	ncRNA metabolic process
thrS	511145.b1719	GO Process	GO:0042221	Response to chemical
thrS	511145.b1719	GO Process	GO:0043038	Amino acid activation
thrS	511145.b1719	GO Process	GO:0043039	tRNA aminoacylation
thrS	511145.b1719	GO Process	GO:0043043	Peptide biosynthetic process
thrS	511145.b1719	GO Process	GO:0043170	Macromolecule metabolic process
thrS	511145.b1719	GO Process	GO:0043436	Oxoacid metabolic process
thrS	511145.b1719	GO Process	GO:0043603	Cellular amide metabolic process
thrS	511145.b1719	GO Process	GO:0043604	Amide biosynthetic process
thrS	511145.b1719	GO Process	GO:0044237	Cellular metabolic process
thrS	511145.b1719	GO Process	GO:0044238	Primary metabolic process
thrS	511145.b1719	GO Process	GO:0044249	Cellular biosynthetic process
thrS	511145.b1719	GO Process	GO:0044260	Cellular macromolecule metabolic process
thrS	511145.b1719	GO Process	GO:0044271	Cellular nitrogen compound biosynthetic process
thrS	511145.b1719	GO Process	GO:0044281	Small molecule metabolic process
thrS	511145.b1719	GO Process	GO:0045947	Negative regulation of translational initiation
thrS	511145.b1719	GO Process	GO:0046483	Heterocycle metabolic process
thrS	511145.b1719	GO Process	GO:0046677	Response to antibiotic
thrS	511145.b1719	GO Process	GO:0048519	Negative regulation of biological process
thrS	511145.b1719	GO Process	GO:0048523	Negative regulation of cellular process
thrS	511145.b1719	GO Process	GO:0050789	Regulation of biological process
thrS	511145.b1719	GO Process	GO:0050794	Regulation of cellular process
thrS	511145.b1719	GO Process	GO:0050896	Response to stimulus
thrS	511145.b1719	GO Process	GO:0051171	Regulation of nitrogen compound metabolic process
thrS	511145.b1719	GO Process	GO:0051172	Negative regulation of nitrogen compound metabolic process
thrS	511145.b1719	GO Process	GO:0051246	Regulation of protein metabolic process
thrS	511145.b1719	GO Process	GO:0051248	Negative regulation of protein metabolic process
thrS	511145.b1719	GO Process	GO:0060255	Regulation of macromolecule metabolic process
thrS	511145.b1719	GO Process	GO:0065007	Biological regulation
thrS	511145.b1719	GO Process	GO:0065008	Regulation of biological quality
thrS	511145.b1719	GO Process	GO:0071704	Organic substance metabolic process
thrS	511145.b1719	GO Process	GO:0080090	Regulation of primary metabolic process
thrS	511145.b1719	GO Process	GO:0090304	Nucleic acid metabolic process
thrS	511145.b1719	GO Process	GO:0106074	aminoacyl-tRNA metabolism involved in translational fidelity
thrS	511145.b1719	GO Process	GO:1901360	Organic cyclic compound metabolic process
thrS	511145.b1719	GO Process	GO:1901564	Organonitrogen compound metabolic process
thrS	511145.b1719	GO Process	GO:1901566	Organonitrogen compound biosynthetic process
thrS	511145.b1719	GO Process	GO:1901576	Organic substance biosynthetic process
thrS	511145.b1719	GO Process	GO:2000112	Regulation of cellular macromolecule biosynthetic process
thrS	511145.b1719	GO Process	GO:2000113	Negative regulation of cellular macromolecule biosynthetic process
thrS	511145.b1719	GO Function	GO:0000049	tRNA binding
thrS	511145.b1719	GO Function	GO:0000166	Nucleotide binding
thrS	511145.b1719	GO Function	GO:0000900	mRNA regulatory element binding translation repressor activity
thrS	511145.b1719	GO Function	GO:0002161	aminoacyl-tRNA editing activity
thrS	511145.b1719	GO Function	GO:0003676	Nucleic acid binding
thrS	511145.b1719	GO Function	GO:0003723	RNA binding
thrS	511145.b1719	GO Function	GO:0003729	mRNA binding
thrS	511145.b1719	GO Function	GO:0003824	Catalytic activity
thrS	511145.b1719	GO Function	GO:0004812	aminoacyl-tRNA ligase activity
thrS	511145.b1719	GO Function	GO:0004829	threonine-tRNA ligase activity
thrS	511145.b1719	GO Function	GO:0005488	Binding
thrS	511145.b1719	GO Function	GO:0005524	ATP binding
thrS	511145.b1719	GO Function	GO:0008270	Zinc ion binding
thrS	511145.b1719	GO Function	GO:0016787	Hydrolase activity
thrS	511145.b1719	GO Function	GO:0016788	Hydrolase activity, acting on ester bonds
thrS	511145.b1719	GO Function	GO:0016874	Ligase activity
thrS	511145.b1719	GO Function	GO:0016875	Ligase activity, forming carbon-oxygen bonds
thrS	511145.b1719	GO Function	GO:0017076	Purine nucleotide binding
thrS	511145.b1719	GO Function	GO:0030371	Translation repressor activity
thrS	511145.b1719	GO Function	GO:0030554	Adenyl nucleotide binding
thrS	511145.b1719	GO Function	GO:0032553	Ribonucleotide binding
thrS	511145.b1719	GO Function	GO:0032555	Purine ribonucleotide binding
thrS	511145.b1719	GO Function	GO:0032559	Adenyl ribonucleotide binding
thrS	511145.b1719	GO Function	GO:0035639	Purine ribonucleoside triphosphate binding
thrS	511145.b1719	GO Function	GO:0036094	Small molecule binding
thrS	511145.b1719	GO Function	GO:0043167	Ion binding
thrS	511145.b1719	GO Function	GO:0043168	Anion binding
thrS	511145.b1719	GO Function	GO:0043169	Cation binding
thrS	511145.b1719	GO Function	GO:0045182	Translation regulator activity
thrS	511145.b1719	GO Function	GO:0046872	Metal ion binding
thrS	511145.b1719	GO Function	GO:0046914	Transition metal ion binding
thrS	511145.b1719	GO Function	GO:0048027	mRNA 5-UTR binding
thrS	511145.b1719	GO Function	GO:0052689	Carboxylic ester hydrolase activity
thrS	511145.b1719	GO Function	GO:0090079	Translation regulator activity, nucleic acid binding
thrS	511145.b1719	GO Function	GO:0097159	Organic cyclic compound binding
thrS	511145.b1719	GO Function	GO:0097367	Carbohydrate derivative binding
thrS	511145.b1719	GO Function	GO:0140098	Catalytic activity, acting on RNA
thrS	511145.b1719	GO Function	GO:0140101	Catalytic activity, acting on a tRNA
thrS	511145.b1719	GO Function	GO:0140640	Catalytic activity, acting on a nucleic acid
thrS	511145.b1719	GO Function	GO:1901265	Nucleoside phosphate binding
thrS	511145.b1719	GO Function	GO:1901363	Heterocyclic compound binding
thrS	511145.b1719	GO Component	GO:0005622	Intracellular anatomical structure
thrS	511145.b1719	GO Component	GO:0005737	Cytoplasm
thrS	511145.b1719	GO Component	GO:0005829	Cytosol
thrS	511145.b1719	GO Component	GO:0110165	Cellular anatomical entity
thrS	511145.b1719	STRING clusters	CL:309	Aminoacyl-tRNA synthetase
thrS	511145.b1719	STRING clusters	CL:311	Aminoacyl-tRNA synthetase
thrS	511145.b1719	STRING clusters	CL:314	Aminoacyl-tRNA synthetase
thrS	511145.b1719	STRING clusters	CL:332	Threonyl/alanyl tRNA synthetase, SAD, and tRNA synthetases class I (M)
thrS	511145.b1719	STRING clusters	CL:35	Translation, and Protein export
thrS	511145.b1719	STRING clusters	CL:37	Translation, and Protein export
thrS	511145.b1719	KEGG	eco00970	Aminoacyl-tRNA biosynthesis
thrS	511145.b1719	COMPARTMENTS	GOCC:0005622	Intracellular
thrS	511145.b1719	COMPARTMENTS	GOCC:0005737	Cytoplasm
thrS	511145.b1719	COMPARTMENTS	GOCC:0005829	Cytosol
thrS	511145.b1719	COMPARTMENTS	GOCC:0110165	Cellular anatomical entity
thrS	511145.b1719	UniProt Keywords	KW-0007	Acetylation
thrS	511145.b1719	UniProt Keywords	KW-0030	Aminoacyl-tRNA synthetase
thrS	511145.b1719	UniProt Keywords	KW-0046	Antibiotic resistance
thrS	511145.b1719	UniProt Keywords	KW-0067	ATP-binding
thrS	511145.b1719	UniProt Keywords	KW-0436	Ligase
thrS	511145.b1719	UniProt Keywords	KW-0479	Metal-binding
thrS	511145.b1719	UniProt Keywords	KW-0547	Nucleotide-binding
thrS	511145.b1719	UniProt Keywords	KW-0648	Protein biosynthesis
thrS	511145.b1719	UniProt Keywords	KW-0678	Repressor
thrS	511145.b1719	UniProt Keywords	KW-0694	RNA-binding
thrS	511145.b1719	UniProt Keywords	KW-0810	Translation regulation
thrS	511145.b1719	UniProt Keywords	KW-0820	tRNA-binding
thrS	511145.b1719	UniProt Keywords	KW-0862	Zinc
thrS	511145.b1719	UniProt Keywords	KW-0963	Cytoplasm
thrS	511145.b1719	Pfam	PF00587	tRNA synthetase class II core domain (G, H, P, S and T)
thrS	511145.b1719	Pfam	PF02824	TGS domain
thrS	511145.b1719	Pfam	PF03129	Anticodon binding domain
thrS	511145.b1719	InterPro	IPR002314	Aminoacyl-tRNA synthetase, class II (G/ P/ S/T)
thrS	511145.b1719	InterPro	IPR002320	Threonine-tRNA ligase, class IIa
thrS	511145.b1719	InterPro	IPR004095	TGS
thrS	511145.b1719	InterPro	IPR004154	Anticodon-binding
thrS	511145.b1719	InterPro	IPR006195	Aminoacyl-tRNA synthetase, class II
thrS	511145.b1719	InterPro	IPR012675	Beta-grasp domain superfamily
thrS	511145.b1719	InterPro	IPR012676	TGS-like
thrS	511145.b1719	InterPro	IPR012947	Threonyl/alanyl tRNA synthetase, SAD
thrS	511145.b1719	InterPro	IPR018163	Threonyl/alanyl tRNA synthetase, class II-like, putative editing domain superfamily
thrS	511145.b1719	InterPro	IPR033728	Threonine-tRNA ligase catalytic core domain
thrS	511145.b1719	InterPro	IPR036621	Anticodon-binding domain superfamily
thrS	511145.b1719	InterPro	IPR045864	Class II Aminoacyl-tRNA synthetase/Biotinyl protein ligase (BPL) and lipoyl protein ligase (LPL)
thrS	511145.b1719	InterPro	IPR047246	Threonine-tRNA ligase, class IIa, anticodon-binding domain
thrS	511145.b1719	SMART	SM00863	Threonyl and Alanyl tRNA synthetase second additional domain
tpx	511145.b1324	GO Process	GO:0006950	Response to stress
tpx	511145.b1324	GO Process	GO:0006979	Response to oxidative stress
tpx	511145.b1324	GO Process	GO:0009636	Response to toxic substance
tpx	511145.b1324	GO Process	GO:0009987	Cellular process
tpx	511145.b1324	GO Process	GO:0033554	Cellular response to stress
tpx	511145.b1324	GO Process	GO:0034599	Cellular response to oxidative stress
tpx	511145.b1324	GO Process	GO:0042221	Response to chemical
tpx	511145.b1324	GO Process	GO:0050896	Response to stimulus
tpx	511145.b1324	GO Process	GO:0051716	Cellular response to stimulus
tpx	511145.b1324	GO Process	GO:0062197	Cellular response to chemical stress
tpx	511145.b1324	GO Process	GO:0070887	Cellular response to chemical stimulus
tpx	511145.b1324	GO Process	GO:0097237	Cellular response to toxic substance
tpx	511145.b1324	GO Process	GO:0098754	Detoxification
tpx	511145.b1324	GO Process	GO:0098869	Cellular oxidant detoxification
tpx	511145.b1324	GO Process	GO:1990748	Cellular detoxification
tpx	511145.b1324	GO Function	GO:0003824	Catalytic activity
tpx	511145.b1324	GO Function	GO:0004601	Peroxidase activity
tpx	511145.b1324	GO Function	GO:0008379	Thioredoxin peroxidase activity
tpx	511145.b1324	GO Function	GO:0016209	Antioxidant activity
tpx	511145.b1324	GO Function	GO:0016491	Oxidoreductase activity
tpx	511145.b1324	GO Function	GO:0016667	Oxidoreductase activity, acting on a sulfur group of donors
tpx	511145.b1324	GO Function	GO:0016671	Oxidoreductase activity, acting on a sulfur group of donors, disulfide as acceptor
tpx	511145.b1324	GO Function	GO:0016684	Oxidoreductase activity, acting on peroxide as acceptor
tpx	511145.b1324	GO Function	GO:0032843	Hydroperoxide reductase activity
tpx	511145.b1324	GO Function	GO:0051920	Peroxiredoxin activity
tpx	511145.b1324	GO Component	GO:0005622	Intracellular anatomical structure
tpx	511145.b1324	GO Component	GO:0005737	Cytoplasm
tpx	511145.b1324	GO Component	GO:0005829	Cytosol
tpx	511145.b1324	GO Component	GO:0042597	Periplasmic space
tpx	511145.b1324	GO Component	GO:0110165	Cellular anatomical entity
tpx	511145.b1324	STRING clusters	CL:1242	Mixed, incl. Iron-sulfur cluster assembly, and Antioxidant activity
tpx	511145.b1324	STRING clusters	CL:1243	Mixed, incl. Iron-sulfur cluster assembly, and Antioxidant activity
tpx	511145.b1324	STRING clusters	CL:1244	Antioxidant activity, and Glutathione metabolism
tpx	511145.b1324	STRING clusters	CL:1245	Antioxidant activity, and Glutathione metabolism
tpx	511145.b1324	STRING clusters	CL:1246	Antioxidant activity, and Iron-sulfur cluster assembly complex
tpx	511145.b1324	STRING clusters	CL:1247	Reactive oxygen species metabolic process, and Thioredoxin domain
tpx	511145.b1324	STRING clusters	CL:1249	Reactive oxygen species metabolic process, and Thioredoxin peroxidase activity
tpx	511145.b1324	COMPARTMENTS	GOCC:0005622	Intracellular
tpx	511145.b1324	COMPARTMENTS	GOCC:0005737	Cytoplasm
tpx	511145.b1324	COMPARTMENTS	GOCC:0005829	Cytosol
tpx	511145.b1324	COMPARTMENTS	GOCC:0110165	Cellular anatomical entity
tpx	511145.b1324	UniProt Keywords	KW-0049	Antioxidant
tpx	511145.b1324	UniProt Keywords	KW-0560	Oxidoreductase
tpx	511145.b1324	UniProt Keywords	KW-0574	Periplasm
tpx	511145.b1324	UniProt Keywords	KW-0575	Peroxidase
tpx	511145.b1324	UniProt Keywords	KW-0676	Redox-active center
tpx	511145.b1324	UniProt Keywords	KW-0963	Cytoplasm
tpx	511145.b1324	UniProt Keywords	KW-1015	Disulfide bond
tpx	511145.b1324	Pfam	PF00578	AhpC/TSA family
tpx	511145.b1324	InterPro	IPR002065	Thiol peroxidase Tpx
tpx	511145.b1324	InterPro	IPR013740	Redoxin
tpx	511145.b1324	InterPro	IPR013766	Thioredoxin domain
tpx	511145.b1324	InterPro	IPR018219	Thiol peroxidase conserved site
tpx	511145.b1324	InterPro	IPR036249	Thioredoxin-like superfamily
trmD	511145.b2607	GO Process	GO:0001510	RNA methylation
trmD	511145.b2607	GO Process	GO:0002939	tRNA N1-guanine methylation
trmD	511145.b2607	GO Process	GO:0006139	Nucleobase-containing compound metabolic process
trmD	511145.b2607	GO Process	GO:0006396	RNA processing
trmD	511145.b2607	GO Process	GO:0006399	tRNA metabolic process
trmD	511145.b2607	GO Process	GO:0006400	tRNA modification
trmD	511145.b2607	GO Process	GO:0006725	Cellular aromatic compound metabolic process
trmD	511145.b2607	GO Process	GO:0006807	Nitrogen compound metabolic process
trmD	511145.b2607	GO Process	GO:0008033	tRNA processing
trmD	511145.b2607	GO Process	GO:0008152	Metabolic process
trmD	511145.b2607	GO Process	GO:0009451	RNA modification
trmD	511145.b2607	GO Process	GO:0009987	Cellular process
trmD	511145.b2607	GO Process	GO:0010467	Gene expression
trmD	511145.b2607	GO Process	GO:0016070	RNA metabolic process
trmD	511145.b2607	GO Process	GO:0030488	tRNA methylation
trmD	511145.b2607	GO Process	GO:0032259	Methylation
trmD	511145.b2607	GO Process	GO:0034470	ncRNA processing
trmD	511145.b2607	GO Process	GO:0034641	Cellular nitrogen compound metabolic process
trmD	511145.b2607	GO Process	GO:0034660	ncRNA metabolic process
trmD	511145.b2607	GO Process	GO:0043170	Macromolecule metabolic process
trmD	511145.b2607	GO Process	GO:0043412	Macromolecule modification
trmD	511145.b2607	GO Process	GO:0043414	Macromolecule methylation
trmD	511145.b2607	GO Process	GO:0044237	Cellular metabolic process
trmD	511145.b2607	GO Process	GO:0044238	Primary metabolic process
trmD	511145.b2607	GO Process	GO:0044260	Cellular macromolecule metabolic process
trmD	511145.b2607	GO Process	GO:0046483	Heterocycle metabolic process
trmD	511145.b2607	GO Process	GO:0071704	Organic substance metabolic process
trmD	511145.b2607	GO Process	GO:0090304	Nucleic acid metabolic process
trmD	511145.b2607	GO Process	GO:1901360	Organic cyclic compound metabolic process
trmD	511145.b2607	GO Function	GO:0000287	Magnesium ion binding
trmD	511145.b2607	GO Function	GO:0003824	Catalytic activity
trmD	511145.b2607	GO Function	GO:0005488	Binding
trmD	511145.b2607	GO Function	GO:0005515	Protein binding
trmD	511145.b2607	GO Function	GO:0008168	Methyltransferase activity
trmD	511145.b2607	GO Function	GO:0008173	RNA methyltransferase activity
trmD	511145.b2607	GO Function	GO:0008175	tRNA methyltransferase activity
trmD	511145.b2607	GO Function	GO:0008757	S-adenosylmethionine-dependent methyltransferase activity
trmD	511145.b2607	GO Function	GO:0009019	tRNA (guanine-N1-)-methyltransferase activity
trmD	511145.b2607	GO Function	GO:0016423	tRNA (guanine) methyltransferase activity
trmD	511145.b2607	GO Function	GO:0016740	Transferase activity
trmD	511145.b2607	GO Function	GO:0016741	Transferase activity, transferring one-carbon groups
trmD	511145.b2607	GO Function	GO:0042802	Identical protein binding
trmD	511145.b2607	GO Function	GO:0043167	Ion binding
trmD	511145.b2607	GO Function	GO:0043169	Cation binding
trmD	511145.b2607	GO Function	GO:0046872	Metal ion binding
trmD	511145.b2607	GO Function	GO:0052906	tRNA (guanine(37)-N(1))-methyltransferase activity
trmD	511145.b2607	GO Function	GO:0140098	Catalytic activity, acting on RNA
trmD	511145.b2607	GO Function	GO:0140101	Catalytic activity, acting on a tRNA
trmD	511145.b2607	GO Function	GO:0140640	Catalytic activity, acting on a nucleic acid
trmD	511145.b2607	GO Component	GO:0005622	Intracellular anatomical structure
trmD	511145.b2607	GO Component	GO:0005737	Cytoplasm
trmD	511145.b2607	GO Component	GO:0005829	Cytosol
trmD	511145.b2607	GO Component	GO:0110165	Cellular anatomical entity
trmD	511145.b2607	STRING clusters	CL:35	Translation, and Protein export
trmD	511145.b2607	STRING clusters	CL:37	Translation, and Protein export
trmD	511145.b2607	STRING clusters	CL:39	Mixed, incl. Ribonucleoprotein, and Translation regulator activity
trmD	511145.b2607	STRING clusters	CL:40	Ribonucleoprotein, and Translation factor activity, RNA binding
trmD	511145.b2607	STRING clusters	CL:41	Ribonucleoprotein, and Translation factor activity, RNA binding
trmD	511145.b2607	STRING clusters	CL:42	Ribosome, and Translation factor activity, RNA binding
trmD	511145.b2607	STRING clusters	CL:45	Ribosome, and Translation factor activity, RNA binding
trmD	511145.b2607	STRING clusters	CL:47	Ribosome, and Translation factor activity, RNA binding
trmD	511145.b2607	COMPARTMENTS	GOCC:0005622	Intracellular
trmD	511145.b2607	COMPARTMENTS	GOCC:0005737	Cytoplasm
trmD	511145.b2607	COMPARTMENTS	GOCC:0005829	Cytosol
trmD	511145.b2607	COMPARTMENTS	GOCC:0110165	Cellular anatomical entity
trmD	511145.b2607	UniProt Keywords	KW-0489	Methyltransferase
trmD	511145.b2607	UniProt Keywords	KW-0808	Transferase
trmD	511145.b2607	UniProt Keywords	KW-0819	tRNA processing
trmD	511145.b2607	UniProt Keywords	KW-0949	S-adenosyl-L-methionine
trmD	511145.b2607	UniProt Keywords	KW-0963	Cytoplasm
trmD	511145.b2607	Pfam	PF01746	tRNA (Guanine-1)-methyltransferase
trmD	511145.b2607	InterPro	IPR002649	tRNA (guanine-N1-)-methyltransferase, bacteria
trmD	511145.b2607	InterPro	IPR016009	tRNA methyltransferase TRMD/TRM10-type domain
trmD	511145.b2607	InterPro	IPR023148	tRNA (guanine-N(1)-)-methyltransferase, C-terminal domain superfamily
trmD	511145.b2607	InterPro	IPR029026	tRNA (guanine-N1-)-methyltransferase, N-terminal
trmD	511145.b2607	InterPro	IPR029028	Alpha/beta knot methyltransferases
trmI	511145.b2960	GO Process	GO:0001510	RNA methylation
trmI	511145.b2960	GO Process	GO:0006139	Nucleobase-containing compound metabolic process
trmI	511145.b2960	GO Process	GO:0006396	RNA processing
trmI	511145.b2960	GO Process	GO:0006399	tRNA metabolic process
trmI	511145.b2960	GO Process	GO:0006400	tRNA modification
trmI	511145.b2960	GO Process	GO:0006725	Cellular aromatic compound metabolic process
trmI	511145.b2960	GO Process	GO:0006807	Nitrogen compound metabolic process
trmI	511145.b2960	GO Process	GO:0008033	tRNA processing
trmI	511145.b2960	GO Process	GO:0008152	Metabolic process
trmI	511145.b2960	GO Process	GO:0009451	RNA modification
trmI	511145.b2960	GO Process	GO:0009987	Cellular process
trmI	511145.b2960	GO Process	GO:0010467	Gene expression
trmI	511145.b2960	GO Process	GO:0016070	RNA metabolic process
trmI	511145.b2960	GO Process	GO:0030488	tRNA methylation
trmI	511145.b2960	GO Process	GO:0032259	Methylation
trmI	511145.b2960	GO Process	GO:0034470	ncRNA processing
trmI	511145.b2960	GO Process	GO:0034641	Cellular nitrogen compound metabolic process
trmI	511145.b2960	GO Process	GO:0034660	ncRNA metabolic process
trmI	511145.b2960	GO Process	GO:0036265	RNA (guanine-N7)-methylation
trmI	511145.b2960	GO Process	GO:0043170	Macromolecule metabolic process
trmI	511145.b2960	GO Process	GO:0043412	Macromolecule modification
trmI	511145.b2960	GO Process	GO:0043414	Macromolecule methylation
trmI	511145.b2960	GO Process	GO:0044237	Cellular metabolic process
trmI	511145.b2960	GO Process	GO:0044238	Primary metabolic process
trmI	511145.b2960	GO Process	GO:0044260	Cellular macromolecule metabolic process
trmI	511145.b2960	GO Process	GO:0046483	Heterocycle metabolic process
trmI	511145.b2960	GO Process	GO:0071704	Organic substance metabolic process
trmI	511145.b2960	GO Process	GO:0090304	Nucleic acid metabolic process
trmI	511145.b2960	GO Process	GO:0106004	tRNA (guanine-N7)-methylation
trmI	511145.b2960	GO Process	GO:1901360	Organic cyclic compound metabolic process
trmI	511145.b2960	GO Function	GO:0003824	Catalytic activity
trmI	511145.b2960	GO Function	GO:0008168	Methyltransferase activity
trmI	511145.b2960	GO Function	GO:0008173	RNA methyltransferase activity
trmI	511145.b2960	GO Function	GO:0008175	tRNA methyltransferase activity
trmI	511145.b2960	GO Function	GO:0008176	tRNA (guanine-N7-)-methyltransferase activity
trmI	511145.b2960	GO Function	GO:0008757	S-adenosylmethionine-dependent methyltransferase activity
trmI	511145.b2960	GO Function	GO:0016423	tRNA (guanine) methyltransferase activity
trmI	511145.b2960	GO Function	GO:0016740	Transferase activity
trmI	511145.b2960	GO Function	GO:0016741	Transferase activity, transferring one-carbon groups
trmI	511145.b2960	GO Function	GO:0140098	Catalytic activity, acting on RNA
trmI	511145.b2960	GO Function	GO:0140101	Catalytic activity, acting on a tRNA
trmI	511145.b2960	GO Function	GO:0140640	Catalytic activity, acting on a nucleic acid
trmI	511145.b2960	GO Component	GO:0005622	Intracellular anatomical structure
trmI	511145.b2960	GO Component	GO:0032991	Protein-containing complex
trmI	511145.b2960	GO Component	GO:0034708	Methyltransferase complex
trmI	511145.b2960	GO Component	GO:0043527	tRNA methyltransferase complex
trmI	511145.b2960	GO Component	GO:0110165	Cellular anatomical entity
trmI	511145.b2960	GO Component	GO:1902494	Catalytic complex
trmI	511145.b2960	GO Component	GO:1990234	Transferase complex
trmI	511145.b2960	STRING clusters	CL:378	Mixed, incl. rRNA modification, and tRNA methylation
trmI	511145.b2960	STRING clusters	CL:379	rRNA modification, and tRNA methylation
trmI	511145.b2960	STRING clusters	CL:380	RNA methylation, and GTP binding domain
trmI	511145.b2960	STRING clusters	CL:381	RNA methylation
trmI	511145.b2960	STRING clusters	CL:382	RNA methyltransferase activity
trmI	511145.b2960	STRING clusters	CL:383	SpoU rRNA Methylase family, and Putative methyltransferase
trmI	511145.b2960	COMPARTMENTS	GOCC:0005622	Intracellular
trmI	511145.b2960	COMPARTMENTS	GOCC:0032991	Protein-containing complex
trmI	511145.b2960	COMPARTMENTS	GOCC:0034708	Methyltransferase complex
trmI	511145.b2960	COMPARTMENTS	GOCC:0043527	tRNA methyltransferase complex
trmI	511145.b2960	COMPARTMENTS	GOCC:1902494	Catalytic complex
trmI	511145.b2960	COMPARTMENTS	GOCC:1990234	Transferase complex
trmI	511145.b2960	UniProt Keywords	KW-0489	Methyltransferase
trmI	511145.b2960	UniProt Keywords	KW-0808	Transferase
trmI	511145.b2960	UniProt Keywords	KW-0819	tRNA processing
trmI	511145.b2960	UniProt Keywords	KW-0949	S-adenosyl-L-methionine
trmI	511145.b2960	Pfam	PF02390	Putative methyltransferase
trmI	511145.b2960	InterPro	IPR003358	tRNA (guanine-N-7) methyltransferase, Trmb type
trmI	511145.b2960	InterPro	IPR029063	S-adenosyl-L-methionine-dependent methyltransferase superfamily
tyrS	511145.b1637	GO Process	GO:0006082	Organic acid metabolic process
tyrS	511145.b1637	GO Process	GO:0006139	Nucleobase-containing compound metabolic process
tyrS	511145.b1637	GO Process	GO:0006399	tRNA metabolic process
tyrS	511145.b1637	GO Process	GO:0006412	Translation
tyrS	511145.b1637	GO Process	GO:0006418	tRNA aminoacylation for protein translation
tyrS	511145.b1637	GO Process	GO:0006437	tyrosyl-tRNA aminoacylation
tyrS	511145.b1637	GO Process	GO:0006518	Peptide metabolic process
tyrS	511145.b1637	GO Process	GO:0006520	Cellular amino acid metabolic process
tyrS	511145.b1637	GO Process	GO:0006725	Cellular aromatic compound metabolic process
tyrS	511145.b1637	GO Process	GO:0006807	Nitrogen compound metabolic process
tyrS	511145.b1637	GO Process	GO:0008152	Metabolic process
tyrS	511145.b1637	GO Process	GO:0009058	Biosynthetic process
tyrS	511145.b1637	GO Process	GO:0009059	Macromolecule biosynthetic process
tyrS	511145.b1637	GO Process	GO:0009987	Cellular process
tyrS	511145.b1637	GO Process	GO:0010467	Gene expression
tyrS	511145.b1637	GO Process	GO:0016070	RNA metabolic process
tyrS	511145.b1637	GO Process	GO:0019538	Protein metabolic process
tyrS	511145.b1637	GO Process	GO:0019752	Carboxylic acid metabolic process
tyrS	511145.b1637	GO Process	GO:0034641	Cellular nitrogen compound metabolic process
tyrS	511145.b1637	GO Process	GO:0034645	Cellular macromolecule biosynthetic process
tyrS	511145.b1637	GO Process	GO:0034660	ncRNA metabolic process
tyrS	511145.b1637	GO Process	GO:0043038	Amino acid activation
tyrS	511145.b1637	GO Process	GO:0043039	tRNA aminoacylation
tyrS	511145.b1637	GO Process	GO:0043043	Peptide biosynthetic process
tyrS	511145.b1637	GO Process	GO:0043170	Macromolecule metabolic process
tyrS	511145.b1637	GO Process	GO:0043436	Oxoacid metabolic process
tyrS	511145.b1637	GO Process	GO:0043603	Cellular amide metabolic process
tyrS	511145.b1637	GO Process	GO:0043604	Amide biosynthetic process
tyrS	511145.b1637	GO Process	GO:0044237	Cellular metabolic process
tyrS	511145.b1637	GO Process	GO:0044238	Primary metabolic process
tyrS	511145.b1637	GO Process	GO:0044249	Cellular biosynthetic process
tyrS	511145.b1637	GO Process	GO:0044260	Cellular macromolecule metabolic process
tyrS	511145.b1637	GO Process	GO:0044271	Cellular nitrogen compound biosynthetic process
tyrS	511145.b1637	GO Process	GO:0044281	Small molecule metabolic process
tyrS	511145.b1637	GO Process	GO:0046483	Heterocycle metabolic process
tyrS	511145.b1637	GO Process	GO:0071704	Organic substance metabolic process
tyrS	511145.b1637	GO Process	GO:0090304	Nucleic acid metabolic process
tyrS	511145.b1637	GO Process	GO:1901360	Organic cyclic compound metabolic process
tyrS	511145.b1637	GO Process	GO:1901564	Organonitrogen compound metabolic process
tyrS	511145.b1637	GO Process	GO:1901566	Organonitrogen compound biosynthetic process
tyrS	511145.b1637	GO Process	GO:1901576	Organic substance biosynthetic process
tyrS	511145.b1637	GO Function	GO:0000166	Nucleotide binding
tyrS	511145.b1637	GO Function	GO:0003676	Nucleic acid binding
tyrS	511145.b1637	GO Function	GO:0003723	RNA binding
tyrS	511145.b1637	GO Function	GO:0003824	Catalytic activity
tyrS	511145.b1637	GO Function	GO:0004812	aminoacyl-tRNA ligase activity
tyrS	511145.b1637	GO Function	GO:0004831	tyrosine-tRNA ligase activity
tyrS	511145.b1637	GO Function	GO:0005488	Binding
tyrS	511145.b1637	GO Function	GO:0005524	ATP binding
tyrS	511145.b1637	GO Function	GO:0016874	Ligase activity
tyrS	511145.b1637	GO Function	GO:0016875	Ligase activity, forming carbon-oxygen bonds
tyrS	511145.b1637	GO Function	GO:0017076	Purine nucleotide binding
tyrS	511145.b1637	GO Function	GO:0030554	Adenyl nucleotide binding
tyrS	511145.b1637	GO Function	GO:0032553	Ribonucleotide binding
tyrS	511145.b1637	GO Function	GO:0032555	Purine ribonucleotide binding
tyrS	511145.b1637	GO Function	GO:0032559	Adenyl ribonucleotide binding
tyrS	511145.b1637	GO Function	GO:0035639	Purine ribonucleoside triphosphate binding
tyrS	511145.b1637	GO Function	GO:0036094	Small molecule binding
tyrS	511145.b1637	GO Function	GO:0043167	Ion binding
tyrS	511145.b1637	GO Function	GO:0043168	Anion binding
tyrS	511145.b1637	GO Function	GO:0097159	Organic cyclic compound binding
tyrS	511145.b1637	GO Function	GO:0097367	Carbohydrate derivative binding
tyrS	511145.b1637	GO Function	GO:0140098	Catalytic activity, acting on RNA
tyrS	511145.b1637	GO Function	GO:0140101	Catalytic activity, acting on a tRNA
tyrS	511145.b1637	GO Function	GO:0140640	Catalytic activity, acting on a nucleic acid
tyrS	511145.b1637	GO Function	GO:1901265	Nucleoside phosphate binding
tyrS	511145.b1637	GO Function	GO:1901363	Heterocyclic compound binding
tyrS	511145.b1637	GO Component	GO:0005622	Intracellular anatomical structure
tyrS	511145.b1637	GO Component	GO:0005737	Cytoplasm
tyrS	511145.b1637	GO Component	GO:0005829	Cytosol
tyrS	511145.b1637	GO Component	GO:0016020	Membrane
tyrS	511145.b1637	GO Component	GO:0110165	Cellular anatomical entity
tyrS	511145.b1637	STRING clusters	CL:309	Aminoacyl-tRNA synthetase
tyrS	511145.b1637	STRING clusters	CL:311	Aminoacyl-tRNA synthetase
tyrS	511145.b1637	STRING clusters	CL:35	Translation, and Protein export
tyrS	511145.b1637	STRING clusters	CL:37	Translation, and Protein export
tyrS	511145.b1637	KEGG	eco00970	Aminoacyl-tRNA biosynthesis
tyrS	511145.b1637	COMPARTMENTS	GOCC:0005622	Intracellular
tyrS	511145.b1637	COMPARTMENTS	GOCC:0005737	Cytoplasm
tyrS	511145.b1637	COMPARTMENTS	GOCC:0005829	Cytosol
tyrS	511145.b1637	COMPARTMENTS	GOCC:0110165	Cellular anatomical entity
tyrS	511145.b1637	UniProt Keywords	KW-0007	Acetylation
tyrS	511145.b1637	UniProt Keywords	KW-0030	Aminoacyl-tRNA synthetase
tyrS	511145.b1637	UniProt Keywords	KW-0067	ATP-binding
tyrS	511145.b1637	UniProt Keywords	KW-0436	Ligase
tyrS	511145.b1637	UniProt Keywords	KW-0547	Nucleotide-binding
tyrS	511145.b1637	UniProt Keywords	KW-0648	Protein biosynthesis
tyrS	511145.b1637	UniProt Keywords	KW-0694	RNA-binding
tyrS	511145.b1637	UniProt Keywords	KW-0963	Cytoplasm
tyrS	511145.b1637	Pfam	PF00579	tRNA synthetases class I (W and Y)
tyrS	511145.b1637	InterPro	IPR001412	Aminoacyl-tRNA synthetase, class I, conserved site
tyrS	511145.b1637	InterPro	IPR002305	Aminoacyl-tRNA synthetase, class Ic
tyrS	511145.b1637	InterPro	IPR002307	Tyrosine-tRNA ligase
tyrS	511145.b1637	InterPro	IPR002942	RNA-binding S4 domain
tyrS	511145.b1637	InterPro	IPR014729	Rossmann-like alpha/beta/alpha sandwich fold
tyrS	511145.b1637	InterPro	IPR024088	Tyrosine-tRNA ligase, bacterial-type
tyrS	511145.b1637	InterPro	IPR024107	Tyrosine-tRNA ligase, bacterial-type, type 1
tyrS	511145.b1637	InterPro	IPR036986	RNA-binding S4 domain superfamily
tyrS	511145.b1637	SMART	SM00363	S4 RNA-binding domain
ubiD	511145.b3843	GO Process	GO:0006082	Organic acid metabolic process
ubiD	511145.b3843	GO Process	GO:0006743	Ubiquinone metabolic process
ubiD	511145.b3843	GO Process	GO:0006744	Ubiquinone biosynthetic process
ubiD	511145.b3843	GO Process	GO:0008152	Metabolic process
ubiD	511145.b3843	GO Process	GO:0009058	Biosynthetic process
ubiD	511145.b3843	GO Process	GO:0009987	Cellular process
ubiD	511145.b3843	GO Process	GO:0016043	Cellular component organization
ubiD	511145.b3843	GO Process	GO:0019752	Carboxylic acid metabolic process
ubiD	511145.b3843	GO Process	GO:0022607	Cellular component assembly
ubiD	511145.b3843	GO Process	GO:0032150	Ubiquinone biosynthetic process from chorismate
ubiD	511145.b3843	GO Process	GO:0034214	Protein hexamerization
ubiD	511145.b3843	GO Process	GO:0042180	Cellular ketone metabolic process
ubiD	511145.b3843	GO Process	GO:0042181	Ketone biosynthetic process
ubiD	511145.b3843	GO Process	GO:0043436	Oxoacid metabolic process
ubiD	511145.b3843	GO Process	GO:0043648	Dicarboxylic acid metabolic process
ubiD	511145.b3843	GO Process	GO:0043933	Protein-containing complex organization
ubiD	511145.b3843	GO Process	GO:0044085	Cellular component biogenesis
ubiD	511145.b3843	GO Process	GO:0044237	Cellular metabolic process
ubiD	511145.b3843	GO Process	GO:0044249	Cellular biosynthetic process
ubiD	511145.b3843	GO Process	GO:0044281	Small molecule metabolic process
ubiD	511145.b3843	GO Process	GO:0044283	Small molecule biosynthetic process
ubiD	511145.b3843	GO Process	GO:0046417	Chorismate metabolic process
ubiD	511145.b3843	GO Process	GO:0051259	Protein complex oligomerization
ubiD	511145.b3843	GO Process	GO:0065003	Protein-containing complex assembly
ubiD	511145.b3843	GO Process	GO:0071704	Organic substance metabolic process
ubiD	511145.b3843	GO Process	GO:0071840	Cellular component organization or biogenesis
ubiD	511145.b3843	GO Process	GO:1901576	Organic substance biosynthetic process
ubiD	511145.b3843	GO Process	GO:1901661	Quinone metabolic process
ubiD	511145.b3843	GO Process	GO:1901663	Quinone biosynthetic process
ubiD	511145.b3843	GO Function	GO:0000166	Nucleotide binding
ubiD	511145.b3843	GO Function	GO:0003824	Catalytic activity
ubiD	511145.b3843	GO Function	GO:0005488	Binding
ubiD	511145.b3843	GO Function	GO:0005515	Protein binding
ubiD	511145.b3843	GO Function	GO:0008694	3-octaprenyl-4-hydroxybenzoate carboxy-lyase activity
ubiD	511145.b3843	GO Function	GO:0016829	Lyase activity
ubiD	511145.b3843	GO Function	GO:0016830	Carbon-carbon lyase activity
ubiD	511145.b3843	GO Function	GO:0016831	Carboxy-lyase activity
ubiD	511145.b3843	GO Function	GO:0030145	Manganese ion binding
ubiD	511145.b3843	GO Function	GO:0032553	Ribonucleotide binding
ubiD	511145.b3843	GO Function	GO:0036094	Small molecule binding
ubiD	511145.b3843	GO Function	GO:0042802	Identical protein binding
ubiD	511145.b3843	GO Function	GO:0043167	Ion binding
ubiD	511145.b3843	GO Function	GO:0043168	Anion binding
ubiD	511145.b3843	GO Function	GO:0043169	Cation binding
ubiD	511145.b3843	GO Function	GO:0046872	Metal ion binding
ubiD	511145.b3843	GO Function	GO:0046914	Transition metal ion binding
ubiD	511145.b3843	GO Function	GO:0097159	Organic cyclic compound binding
ubiD	511145.b3843	GO Function	GO:0097367	Carbohydrate derivative binding
ubiD	511145.b3843	GO Function	GO:0120233	prenyl-FMNH2 binding
ubiD	511145.b3843	GO Function	GO:1901265	Nucleoside phosphate binding
ubiD	511145.b3843	GO Function	GO:1901363	Heterocyclic compound binding
ubiD	511145.b3843	GO Component	GO:0005622	Intracellular anatomical structure
ubiD	511145.b3843	GO Component	GO:0005737	Cytoplasm
ubiD	511145.b3843	GO Component	GO:0005829	Cytosol
ubiD	511145.b3843	GO Component	GO:0005886	Plasma membrane
ubiD	511145.b3843	GO Component	GO:0016020	Membrane
ubiD	511145.b3843	GO Component	GO:0071944	Cell periphery
ubiD	511145.b3843	GO Component	GO:0110165	Cellular anatomical entity
ubiD	511145.b3843	STRING clusters	CL:2534	Mixed, incl. Quinone metabolic process, and Tetrapyrrole biosynthetic process
ubiD	511145.b3843	STRING clusters	CL:2535	Ubiquinone metabolic process, and Tetrapyrrole biosynthetic process
ubiD	511145.b3843	STRING clusters	CL:2536	Ubiquinone metabolic process, and TAT protein transport complex
ubiD	511145.b3843	STRING clusters	CL:2537	Ubiquinone metabolic process, and TAT protein transport complex
ubiD	511145.b3843	STRING clusters	CL:2539	Ubiquinone biosynthesis
ubiD	511145.b3843	KEGG	eco00130	Ubiquinone and other terpenoid-quinone biosynthesis
ubiD	511145.b3843	KEGG	eco01100	Metabolic pathways
ubiD	511145.b3843	KEGG	eco01110	Biosynthesis of secondary metabolites
ubiD	511145.b3843	COMPARTMENTS	GOCC:0005622	Intracellular
ubiD	511145.b3843	COMPARTMENTS	GOCC:0005737	Cytoplasm
ubiD	511145.b3843	COMPARTMENTS	GOCC:0005829	Cytosol
ubiD	511145.b3843	COMPARTMENTS	GOCC:0110165	Cellular anatomical entity
ubiD	511145.b3843	UniProt Keywords	KW-0210	Decarboxylase
ubiD	511145.b3843	UniProt Keywords	KW-0285	Flavoprotein
ubiD	511145.b3843	UniProt Keywords	KW-0288	FMN
ubiD	511145.b3843	UniProt Keywords	KW-0456	Lyase
ubiD	511145.b3843	UniProt Keywords	KW-0464	Manganese
ubiD	511145.b3843	UniProt Keywords	KW-0472	Membrane
ubiD	511145.b3843	UniProt Keywords	KW-0479	Metal-binding
ubiD	511145.b3843	UniProt Keywords	KW-0831	Ubiquinone biosynthesis
ubiD	511145.b3843	UniProt Keywords	KW-1003	Cell membrane
ubiD	511145.b3843	Pfam	PF01977	3-octaprenyl-4-hydroxybenzoate carboxy-lyase
ubiD	511145.b3843	InterPro	IPR002830	UbiD decarboxylyase family
ubiD	511145.b3843	InterPro	IPR023677	UbiD decarboxylyase, bacteria
umpG	511145.b2744	GO Process	GO:0006139	Nucleobase-containing compound metabolic process
umpG	511145.b2744	GO Process	GO:0006220	Pyrimidine nucleotide metabolic process
umpG	511145.b2744	GO Process	GO:0006244	Pyrimidine nucleotide catabolic process
umpG	511145.b2744	GO Process	GO:0006725	Cellular aromatic compound metabolic process
umpG	511145.b2744	GO Process	GO:0006753	Nucleoside phosphate metabolic process
umpG	511145.b2744	GO Process	GO:0006793	Phosphorus metabolic process
umpG	511145.b2744	GO Process	GO:0006796	Phosphate-containing compound metabolic process
umpG	511145.b2744	GO Process	GO:0006807	Nitrogen compound metabolic process
umpG	511145.b2744	GO Process	GO:0008152	Metabolic process
umpG	511145.b2744	GO Process	GO:0009056	Catabolic process
umpG	511145.b2744	GO Process	GO:0009117	Nucleotide metabolic process
umpG	511145.b2744	GO Process	GO:0009123	Nucleoside monophosphate metabolic process
umpG	511145.b2744	GO Process	GO:0009125	Nucleoside monophosphate catabolic process
umpG	511145.b2744	GO Process	GO:0009129	Pyrimidine nucleoside monophosphate metabolic process
umpG	511145.b2744	GO Process	GO:0009131	Pyrimidine nucleoside monophosphate catabolic process
umpG	511145.b2744	GO Process	GO:0009158	Ribonucleoside monophosphate catabolic process
umpG	511145.b2744	GO Process	GO:0009161	Ribonucleoside monophosphate metabolic process
umpG	511145.b2744	GO Process	GO:0009166	Nucleotide catabolic process
umpG	511145.b2744	GO Process	GO:0009173	Pyrimidine ribonucleoside monophosphate metabolic process
umpG	511145.b2744	GO Process	GO:0009175	Pyrimidine ribonucleoside monophosphate catabolic process
umpG	511145.b2744	GO Process	GO:0009218	Pyrimidine ribonucleotide metabolic process
umpG	511145.b2744	GO Process	GO:0009222	Pyrimidine ribonucleotide catabolic process
umpG	511145.b2744	GO Process	GO:0009259	Ribonucleotide metabolic process
umpG	511145.b2744	GO Process	GO:0009261	Ribonucleotide catabolic process
umpG	511145.b2744	GO Process	GO:0009987	Cellular process
umpG	511145.b2744	GO Process	GO:0019439	Aromatic compound catabolic process
umpG	511145.b2744	GO Process	GO:0019637	Organophosphate metabolic process
umpG	511145.b2744	GO Process	GO:0019693	Ribose phosphate metabolic process
umpG	511145.b2744	GO Process	GO:0034641	Cellular nitrogen compound metabolic process
umpG	511145.b2744	GO Process	GO:0034655	Nucleobase-containing compound catabolic process
umpG	511145.b2744	GO Process	GO:0044237	Cellular metabolic process
umpG	511145.b2744	GO Process	GO:0044238	Primary metabolic process
umpG	511145.b2744	GO Process	GO:0044248	Cellular catabolic process
umpG	511145.b2744	GO Process	GO:0044270	Cellular nitrogen compound catabolic process
umpG	511145.b2744	GO Process	GO:0044281	Small molecule metabolic process
umpG	511145.b2744	GO Process	GO:0046049	UMP metabolic process
umpG	511145.b2744	GO Process	GO:0046050	UMP catabolic process
umpG	511145.b2744	GO Process	GO:0046434	Organophosphate catabolic process
umpG	511145.b2744	GO Process	GO:0046483	Heterocycle metabolic process
umpG	511145.b2744	GO Process	GO:0046700	Heterocycle catabolic process
umpG	511145.b2744	GO Process	GO:0055086	Nucleobase-containing small molecule metabolic process
umpG	511145.b2744	GO Process	GO:0071704	Organic substance metabolic process
umpG	511145.b2744	GO Process	GO:0072527	Pyrimidine-containing compound metabolic process
umpG	511145.b2744	GO Process	GO:0072529	Pyrimidine-containing compound catabolic process
umpG	511145.b2744	GO Process	GO:1901135	Carbohydrate derivative metabolic process
umpG	511145.b2744	GO Process	GO:1901136	Carbohydrate derivative catabolic process
umpG	511145.b2744	GO Process	GO:1901292	Nucleoside phosphate catabolic process
umpG	511145.b2744	GO Process	GO:1901360	Organic cyclic compound metabolic process
umpG	511145.b2744	GO Process	GO:1901361	Organic cyclic compound catabolic process
umpG	511145.b2744	GO Process	GO:1901564	Organonitrogen compound metabolic process
umpG	511145.b2744	GO Process	GO:1901565	Organonitrogen compound catabolic process
umpG	511145.b2744	GO Process	GO:1901575	Organic substance catabolic process
umpG	511145.b2744	GO Function	GO:0000166	Nucleotide binding
umpG	511145.b2744	GO Function	GO:0003824	Catalytic activity
umpG	511145.b2744	GO Function	GO:0004309	Exopolyphosphatase activity
umpG	511145.b2744	GO Function	GO:0005488	Binding
umpG	511145.b2744	GO Function	GO:0008252	Nucleotidase activity
umpG	511145.b2744	GO Function	GO:0008253	5-nucleotidase activity
umpG	511145.b2744	GO Function	GO:0008254	3-nucleotidase activity
umpG	511145.b2744	GO Function	GO:0016462	Pyrophosphatase activity
umpG	511145.b2744	GO Function	GO:0016787	Hydrolase activity
umpG	511145.b2744	GO Function	GO:0016788	Hydrolase activity, acting on ester bonds
umpG	511145.b2744	GO Function	GO:0016791	Phosphatase activity
umpG	511145.b2744	GO Function	GO:0016817	Hydrolase activity, acting on acid anhydrides
umpG	511145.b2744	GO Function	GO:0016818	Hydrolase activity, acting on acid anhydrides, in phosphorus-containing anhydrides
umpG	511145.b2744	GO Function	GO:0030145	Manganese ion binding
umpG	511145.b2744	GO Function	GO:0036094	Small molecule binding
umpG	511145.b2744	GO Function	GO:0042578	Phosphoric ester hydrolase activity
umpG	511145.b2744	GO Function	GO:0043167	Ion binding
umpG	511145.b2744	GO Function	GO:0043169	Cation binding
umpG	511145.b2744	GO Function	GO:0046872	Metal ion binding
umpG	511145.b2744	GO Function	GO:0046914	Transition metal ion binding
umpG	511145.b2744	GO Function	GO:0050484	GMP 5-nucleotidase activity
umpG	511145.b2744	GO Function	GO:0097159	Organic cyclic compound binding
umpG	511145.b2744	GO Function	GO:0106411	XMP 5-nucleosidase activity
umpG	511145.b2744	GO Function	GO:1901265	Nucleoside phosphate binding
umpG	511145.b2744	GO Function	GO:1901363	Heterocyclic compound binding
umpG	511145.b2744	GO Component	GO:0005622	Intracellular anatomical structure
umpG	511145.b2744	GO Component	GO:0005737	Cytoplasm
umpG	511145.b2744	GO Component	GO:0110165	Cellular anatomical entity
umpG	511145.b2744	STRING clusters	CL:1684	Mixed, incl. Nucleoside monophosphate biosynthetic process, and Pyrimidine metabolism
umpG	511145.b2744	STRING clusters	CL:1685	Nucleoside monophosphate biosynthetic process, and Nucleobase transport
umpG	511145.b2744	STRING clusters	CL:1784	Mixed, incl. Pyrimidine nucleotide salvage, and Nucleotidase activity
umpG	511145.b2744	KEGG	eco00230	Purine metabolism
umpG	511145.b2744	KEGG	eco00240	Pyrimidine metabolism
umpG	511145.b2744	KEGG	eco00760	Nicotinate and nicotinamide metabolism
umpG	511145.b2744	KEGG	eco01100	Metabolic pathways
umpG	511145.b2744	KEGG	eco01110	Biosynthesis of secondary metabolites
umpG	511145.b2744	UniProt Keywords	KW-0170	Cobalt
umpG	511145.b2744	UniProt Keywords	KW-0378	Hydrolase
umpG	511145.b2744	UniProt Keywords	KW-0460	Magnesium
umpG	511145.b2744	UniProt Keywords	KW-0464	Manganese
umpG	511145.b2744	UniProt Keywords	KW-0479	Metal-binding
umpG	511145.b2744	UniProt Keywords	KW-0533	Nickel
umpG	511145.b2744	UniProt Keywords	KW-0547	Nucleotide-binding
umpG	511145.b2744	UniProt Keywords	KW-0862	Zinc
umpG	511145.b2744	UniProt Keywords	KW-0963	Cytoplasm
umpG	511145.b2744	Pfam	PF01975	Survival protein SurE
umpG	511145.b2744	InterPro	IPR002828	Survival protein SurE-like phosphatase/nucleotidase
umpG	511145.b2744	InterPro	IPR030048	Survival protein SurE
umpG	511145.b2744	InterPro	IPR036523	SurE-like phosphatase/nucleotidase superfamily
ung	511145.b2580	GO Process	GO:0006139	Nucleobase-containing compound metabolic process
ung	511145.b2580	GO Process	GO:0006259	DNA metabolic process
ung	511145.b2580	GO Process	GO:0006281	DNA repair
ung	511145.b2580	GO Process	GO:0006284	Base-excision repair
ung	511145.b2580	GO Process	GO:0006285	Base-excision repair, AP site formation
ung	511145.b2580	GO Process	GO:0006725	Cellular aromatic compound metabolic process
ung	511145.b2580	GO Process	GO:0006807	Nitrogen compound metabolic process
ung	511145.b2580	GO Process	GO:0006950	Response to stress
ung	511145.b2580	GO Process	GO:0006974	Cellular response to DNA damage stimulus
ung	511145.b2580	GO Process	GO:0008152	Metabolic process
ung	511145.b2580	GO Process	GO:0009987	Cellular process
ung	511145.b2580	GO Process	GO:0033554	Cellular response to stress
ung	511145.b2580	GO Process	GO:0034641	Cellular nitrogen compound metabolic process
ung	511145.b2580	GO Process	GO:0043170	Macromolecule metabolic process
ung	511145.b2580	GO Process	GO:0044237	Cellular metabolic process
ung	511145.b2580	GO Process	GO:0044238	Primary metabolic process
ung	511145.b2580	GO Process	GO:0044260	Cellular macromolecule metabolic process
ung	511145.b2580	GO Process	GO:0046483	Heterocycle metabolic process
ung	511145.b2580	GO Process	GO:0050896	Response to stimulus
ung	511145.b2580	GO Process	GO:0051716	Cellular response to stimulus
ung	511145.b2580	GO Process	GO:0071704	Organic substance metabolic process
ung	511145.b2580	GO Process	GO:0090304	Nucleic acid metabolic process
ung	511145.b2580	GO Process	GO:0097510	Base-excision repair, AP site formation via deaminated base removal
ung	511145.b2580	GO Process	GO:1901360	Organic cyclic compound metabolic process
ung	511145.b2580	GO Function	GO:0003824	Catalytic activity
ung	511145.b2580	GO Function	GO:0004844	Uracil DNA N-glycosylase activity
ung	511145.b2580	GO Function	GO:0016787	Hydrolase activity
ung	511145.b2580	GO Function	GO:0016798	Hydrolase activity, acting on glycosyl bonds
ung	511145.b2580	GO Function	GO:0016799	Hydrolase activity, hydrolyzing N-glycosyl compounds
ung	511145.b2580	GO Function	GO:0019104	DNA N-glycosylase activity
ung	511145.b2580	GO Function	GO:0097506	Deaminated base DNA N-glycosylase activity
ung	511145.b2580	GO Function	GO:0140097	Catalytic activity, acting on DNA
ung	511145.b2580	GO Function	GO:0140640	Catalytic activity, acting on a nucleic acid
ung	511145.b2580	GO Component	GO:0005622	Intracellular anatomical structure
ung	511145.b2580	GO Component	GO:0005737	Cytoplasm
ung	511145.b2580	GO Component	GO:0110165	Cellular anatomical entity
ung	511145.b2580	STRING clusters	CL:689	DNA repair, and Chromosome
ung	511145.b2580	STRING clusters	CL:690	DNA repair, and Chromosome
ung	511145.b2580	STRING clusters	CL:691	DNA repair, and Chromosome
ung	511145.b2580	STRING clusters	CL:693	DNA damage, and Exodeoxyribonuclease activity
ung	511145.b2580	STRING clusters	CL:772	Base excision repair
ung	511145.b2580	KEGG	eco03410	Base excision repair
ung	511145.b2580	COMPARTMENTS	GOCC:0005622	Intracellular
ung	511145.b2580	COMPARTMENTS	GOCC:0005737	Cytoplasm
ung	511145.b2580	COMPARTMENTS	GOCC:0110165	Cellular anatomical entity
ung	511145.b2580	UniProt Keywords	KW-0227	DNA damage
ung	511145.b2580	UniProt Keywords	KW-0234	DNA repair
ung	511145.b2580	UniProt Keywords	KW-0378	Hydrolase
ung	511145.b2580	UniProt Keywords	KW-0963	Cytoplasm
ung	511145.b2580	InterPro	IPR002043	Uracil-DNA glycosylase family 1
ung	511145.b2580	InterPro	IPR005122	Uracil-DNA glycosylase-like
ung	511145.b2580	InterPro	IPR018085	Uracil-DNA glycosylase, active site
ung	511145.b2580	InterPro	IPR036895	Uracil-DNA glycosylase-like domain superfamily
ung	511145.b2580	SMART	SM00987	UreE urease accessory protein, C-terminal domain
yagE	511145.b0268	GO Process	GO:0005975	Carbohydrate metabolic process
yagE	511145.b0268	GO Process	GO:0006082	Organic acid metabolic process
yagE	511145.b0268	GO Process	GO:0008152	Metabolic process
yagE	511145.b0268	GO Process	GO:0009056	Catabolic process
yagE	511145.b0268	GO Process	GO:0009987	Cellular process
yagE	511145.b0268	GO Process	GO:0016052	Carbohydrate catabolic process
yagE	511145.b0268	GO Process	GO:0016054	Organic acid catabolic process
yagE	511145.b0268	GO Process	GO:0019520	Aldonic acid metabolic process
yagE	511145.b0268	GO Process	GO:0019752	Carboxylic acid metabolic process
yagE	511145.b0268	GO Process	GO:0032787	Monocarboxylic acid metabolic process
yagE	511145.b0268	GO Process	GO:0043436	Oxoacid metabolic process
yagE	511145.b0268	GO Process	GO:0044237	Cellular metabolic process
yagE	511145.b0268	GO Process	GO:0044238	Primary metabolic process
yagE	511145.b0268	GO Process	GO:0044248	Cellular catabolic process
yagE	511145.b0268	GO Process	GO:0044262	Cellular carbohydrate metabolic process
yagE	511145.b0268	GO Process	GO:0044275	Cellular carbohydrate catabolic process
yagE	511145.b0268	GO Process	GO:0044281	Small molecule metabolic process
yagE	511145.b0268	GO Process	GO:0044282	Small molecule catabolic process
yagE	511145.b0268	GO Process	GO:0046176	Aldonic acid catabolic process
yagE	511145.b0268	GO Process	GO:0046395	Carboxylic acid catabolic process
yagE	511145.b0268	GO Process	GO:0071704	Organic substance metabolic process
yagE	511145.b0268	GO Process	GO:0072329	Monocarboxylic acid catabolic process
yagE	511145.b0268	GO Process	GO:1901575	Organic substance catabolic process
yagE	511145.b0268	GO Function	GO:0003824	Catalytic activity
yagE	511145.b0268	GO Function	GO:0005488	Binding
yagE	511145.b0268	GO Function	GO:0005515	Protein binding
yagE	511145.b0268	GO Function	GO:0016829	Lyase activity
yagE	511145.b0268	GO Function	GO:0016830	Carbon-carbon lyase activity
yagE	511145.b0268	GO Function	GO:0016832	Aldehyde-lyase activity
yagE	511145.b0268	GO Function	GO:0042802	Identical protein binding
yagE	511145.b0268	GO Function	GO:0047440	2-dehydro-3-deoxy-D-pentonate aldolase activity
yagE	511145.b0268	GO Function	GO:0061677	2-dehydro-3-deoxy-D-gluconate aldolase activity
yagE	511145.b0268	GO Component	GO:0005622	Intracellular anatomical structure
yagE	511145.b0268	GO Component	GO:0005737	Cytoplasm
yagE	511145.b0268	GO Component	GO:0005829	Cytosol
yagE	511145.b0268	GO Component	GO:0110165	Cellular anatomical entity
yagE	511145.b0268	STRING clusters	CL:5419	Mixed, incl. Pentose and glucuronate interconversions, and Carbohydrate transport
yagE	511145.b0268	STRING clusters	CL:5420	Mixed, incl. Pentose and glucuronate interconversions, and Fucose metabolism
yagE	511145.b0268	STRING clusters	CL:5421	Mixed, incl. Pentose metabolic process, and Rhamnose metabolic process
yagE	511145.b0268	STRING clusters	CL:5485	Mixed, incl. Sorbitol transmembrane transport, and Xylonate dehydratase activity
yagE	511145.b0268	STRING clusters	CL:5505	Xylonate dehydratase activity, and 2-dehydro-3-deoxy-D-pentonate aldolase activity
yagE	511145.b0268	KEGG	eco00040	Pentose and glucuronate interconversions
yagE	511145.b0268	KEGG	eco01100	Metabolic pathways
yagE	511145.b0268	UniProt Keywords	KW-0456	Lyase
yagE	511145.b0268	UniProt Keywords	KW-0704	Schiff base
yagE	511145.b0268	UniProt Keywords	KW-0963	Cytoplasm
yagE	511145.b0268	InterPro	IPR002220	DapA-like
yagE	511145.b0268	InterPro	IPR013785	Aldolase-type TIM barrel
yagE	511145.b0268	InterPro	IPR020624	Schiff base-forming aldolase, conserved site
yagE	511145.b0268	InterPro	IPR020625	Schiff base-forming aldolase, active site
yagE	511145.b0268	SMART	SM01130	Dihydrodipicolinate synthetase family
yccX	511145.b0968	GO Process	GO:0006950	Response to stress
yccX	511145.b0968	GO Process	GO:0009266	Response to temperature stimulus
yccX	511145.b0968	GO Process	GO:0009408	Response to heat
yccX	511145.b0968	GO Process	GO:0009628	Response to abiotic stimulus
yccX	511145.b0968	GO Process	GO:0050896	Response to stimulus
yccX	511145.b0968	GO Function	GO:0003824	Catalytic activity
yccX	511145.b0968	GO Function	GO:0003998	Acylphosphatase activity
yccX	511145.b0968	GO Function	GO:0016787	Hydrolase activity
yccX	511145.b0968	GO Function	GO:0016817	Hydrolase activity, acting on acid anhydrides
yccX	511145.b0968	GO Function	GO:0016818	Hydrolase activity, acting on acid anhydrides, in phosphorus-containing anhydrides
yccX	511145.b0968	STRING clusters	CL:5419	Mixed, incl. Pentose and glucuronate interconversions, and Carbohydrate transport
yccX	511145.b0968	STRING clusters	CL:5567	Mixed, incl. Aldonic acid metabolic process, and Pentose and glucuronate interconversions
yccX	511145.b0968	STRING clusters	CL:5568	Mixed, incl. Pentose and glucuronate interconversions, and GntR, C-terminal
yccX	511145.b0968	STRING clusters	CL:5644	Mixed, incl. Lysozyme inhibitor activity, and Acylphosphatase
yccX	511145.b0968	KEGG	eco00620	Pyruvate metabolism
yccX	511145.b0968	KEGG	eco00627	Aminobenzoate degradation
yccX	511145.b0968	KEGG	eco01100	Metabolic pathways
yccX	511145.b0968	KEGG	eco01120	Microbial metabolism in diverse environments
yccX	511145.b0968	UniProt Keywords	KW-0378	Hydrolase
yccX	511145.b0968	UniProt Keywords	KW-1015	Disulfide bond
yccX	511145.b0968	Pfam	PF00708	Acylphosphatase
yccX	511145.b0968	InterPro	IPR001792	Acylphosphatase-like domain
yccX	511145.b0968	InterPro	IPR017968	Acylphosphatase, conserved site
yccX	511145.b0968	InterPro	IPR020456	Acylphosphatase
yccX	511145.b0968	InterPro	IPR028627	Acylphosphatase, bacteria
yccX	511145.b0968	InterPro	IPR036046	Acylphosphatase-like domain superfamily
yceA	511145.b1055	GO Process	GO:0002097	tRNA wobble base modification
yceA	511145.b1055	GO Process	GO:0002098	tRNA wobble uridine modification
yceA	511145.b1055	GO Process	GO:0006139	Nucleobase-containing compound metabolic process
yceA	511145.b1055	GO Process	GO:0006396	RNA processing
yceA	511145.b1055	GO Process	GO:0006399	tRNA metabolic process
yceA	511145.b1055	GO Process	GO:0006400	tRNA modification
yceA	511145.b1055	GO Process	GO:0006725	Cellular aromatic compound metabolic process
yceA	511145.b1055	GO Process	GO:0006807	Nitrogen compound metabolic process
yceA	511145.b1055	GO Process	GO:0008033	tRNA processing
yceA	511145.b1055	GO Process	GO:0008152	Metabolic process
yceA	511145.b1055	GO Process	GO:0009451	RNA modification
yceA	511145.b1055	GO Process	GO:0009987	Cellular process
yceA	511145.b1055	GO Process	GO:0010467	Gene expression
yceA	511145.b1055	GO Process	GO:0016070	RNA metabolic process
yceA	511145.b1055	GO Process	GO:0034470	ncRNA processing
yceA	511145.b1055	GO Process	GO:0034641	Cellular nitrogen compound metabolic process
yceA	511145.b1055	GO Process	GO:0034660	ncRNA metabolic process
yceA	511145.b1055	GO Process	GO:0043170	Macromolecule metabolic process
yceA	511145.b1055	GO Process	GO:0043412	Macromolecule modification
yceA	511145.b1055	GO Process	GO:0044237	Cellular metabolic process
yceA	511145.b1055	GO Process	GO:0044238	Primary metabolic process
yceA	511145.b1055	GO Process	GO:0046483	Heterocycle metabolic process
yceA	511145.b1055	GO Process	GO:0071704	Organic substance metabolic process
yceA	511145.b1055	GO Process	GO:0090304	Nucleic acid metabolic process
yceA	511145.b1055	GO Process	GO:1901360	Organic cyclic compound metabolic process
yceA	511145.b1055	GO Function	GO:0003824	Catalytic activity
yceA	511145.b1055	GO Function	GO:0016491	Oxidoreductase activity
yceA	511145.b1055	GO Function	GO:0016705	Oxidoreductase activity, acting on paired donors, with incorporation or reduction of molecular oxygen
yceA	511145.b1055	STRING clusters	CL:5690	Mixed, incl. MFS/sugar transport protein, and Glycoside hydrolase family 31
yceA	511145.b1055	Pfam	PF12368	Rhodanase C-terminal
yceA	511145.b1055	InterPro	IPR001763	Rhodanese-like domain
yceA	511145.b1055	InterPro	IPR020936	tRNA uridine(34) hydroxylase
yceA	511145.b1055	InterPro	IPR022111	Rhodanase, C-terminal
yceA	511145.b1055	InterPro	IPR036873	Rhodanese-like domain superfamily
yceA	511145.b1055	InterPro	IPR040503	tRNA uridine(34) hydroxylase, N-terminal
yceA	511145.b1055	SMART	SM00450	Rhodanese Homology Domain
ycfP	511145.b1108	GO Component	GO:0005622	Intracellular anatomical structure
ycfP	511145.b1108	GO Component	GO:0005737	Cytoplasm
ycfP	511145.b1108	GO Component	GO:0005829	Cytosol
ycfP	511145.b1108	GO Component	GO:0110165	Cellular anatomical entity
ycfP	511145.b1108	STRING clusters	CL:1197	Mixed, incl. SEC-C motif, and D-alanine catabolic process
ycfP	511145.b1108	STRING clusters	CL:897	Mixed, incl. Peptidoglycan metabolic process, and Cell division site
ycfP	511145.b1108	COMPARTMENTS	GOCC:0005622	Intracellular
ycfP	511145.b1108	COMPARTMENTS	GOCC:0005737	Cytoplasm
ycfP	511145.b1108	COMPARTMENTS	GOCC:0005829	Cytosol
ycfP	511145.b1108	COMPARTMENTS	GOCC:0110165	Cellular anatomical entity
ycfP	511145.b1108	Pfam	PF05728	Uncharacterised protein family (UPF0227)
ycfP	511145.b1108	InterPro	IPR008886	Uncharacterised protein family UPF0227/Esterase YqiA
ycfP	511145.b1108	InterPro	IPR022987	Uncharacterised protein family UPF0227
ycfP	511145.b1108	InterPro	IPR029058	Alpha/Beta hydrolase fold
ydjJ	511145.b1774	GO Function	GO:0003824	Catalytic activity
ydjJ	511145.b1774	GO Function	GO:0005488	Binding
ydjJ	511145.b1774	GO Function	GO:0008270	Zinc ion binding
ydjJ	511145.b1774	GO Function	GO:0016491	Oxidoreductase activity
ydjJ	511145.b1774	GO Function	GO:0016614	Oxidoreductase activity, acting on CH-OH group of donors
ydjJ	511145.b1774	GO Function	GO:0016616	Oxidoreductase activity, acting on the CH-OH group of donors, NAD or NADP as acceptor
ydjJ	511145.b1774	GO Function	GO:0043167	Ion binding
ydjJ	511145.b1774	GO Function	GO:0043169	Cation binding
ydjJ	511145.b1774	GO Function	GO:0046872	Metal ion binding
ydjJ	511145.b1774	GO Function	GO:0046914	Transition metal ion binding
ydjJ	511145.b1774	STRING clusters	CL:5950	Mixed, incl. Amine metabolic process, and Alcohol dehydrogenase-like, N-terminal
ydjJ	511145.b1774	STRING clusters	CL:5951	Mixed, incl. Ethanolamine catabolic process, and Alcohol dehydrogenase-like, N-terminal
ydjJ	511145.b1774	STRING clusters	CL:6027	Mixed, incl. Zinc-binding dehydrogenase, and KR domain
ydjJ	511145.b1774	STRING clusters	CL:6028	Mixed, incl. Zinc-binding dehydrogenase, and KR domain
ydjJ	511145.b1774	STRING clusters	CL:6029	Mixed, incl. The UbiC transcription regulator-associated (UTRA) domain is a conserved ligand-binding domain that has a similar fold to PUBMED:12757941. It is believed to modulate activity of bacterial transcription factors in response to binding small molecules., and Zinc-binding dehydrogenase
ydjJ	511145.b1774	STRING clusters	CL:6031	Zinc-binding dehydrogenase, and Fructose-bisphosphate aldolase class-II
ydjJ	511145.b1774	UniProt Keywords	KW-0479	Metal-binding
ydjJ	511145.b1774	UniProt Keywords	KW-0560	Oxidoreductase
ydjJ	511145.b1774	UniProt Keywords	KW-0862	Zinc
ydjJ	511145.b1774	Pfam	PF00107	Zinc-binding dehydrogenase
ydjJ	511145.b1774	Pfam	PF08240	Alcohol dehydrogenase GroES-like domain
ydjJ	511145.b1774	InterPro	IPR002328	Alcohol dehydrogenase, zinc-type, conserved site
ydjJ	511145.b1774	InterPro	IPR011032	GroES-like superfamily
ydjJ	511145.b1774	InterPro	IPR013149	Alcohol dehydrogenase-like, C-terminal
ydjJ	511145.b1774	InterPro	IPR013154	Alcohol dehydrogenase-like, N-terminal
ydjJ	511145.b1774	InterPro	IPR020843	Polyketide synthase, enoylreductase domain
ydjJ	511145.b1774	InterPro	IPR036291	NAD(P)-binding domain superfamily
ydjJ	511145.b1774	InterPro	IPR045306	Sorbitol dehydrogenase-like
yggP	511145.b4465	GO Function	GO:0003824	Catalytic activity
yggP	511145.b4465	GO Function	GO:0016491	Oxidoreductase activity
yggP	511145.b4465	STRING clusters	CL:5950	Mixed, incl. Amine metabolic process, and Alcohol dehydrogenase-like, N-terminal
yggP	511145.b4465	STRING clusters	CL:5951	Mixed, incl. Ethanolamine catabolic process, and Alcohol dehydrogenase-like, N-terminal
yggP	511145.b4465	STRING clusters	CL:6027	Mixed, incl. Zinc-binding dehydrogenase, and KR domain
yggP	511145.b4465	STRING clusters	CL:6028	Mixed, incl. Zinc-binding dehydrogenase, and KR domain
yggP	511145.b4465	KEGG	eco00051	Fructose and mannose metabolism
yggP	511145.b4465	KEGG	eco01100	Metabolic pathways
yggP	511145.b4465	UniProt Keywords	KW-0560	Oxidoreductase
yggP	511145.b4465	Pfam	PF00107	Zinc-binding dehydrogenase
yggP	511145.b4465	Pfam	PF08240	Alcohol dehydrogenase GroES-like domain
yggP	511145.b4465	InterPro	IPR011032	GroES-like superfamily
yggP	511145.b4465	InterPro	IPR013149	Alcohol dehydrogenase-like, C-terminal
yggP	511145.b4465	InterPro	IPR013154	Alcohol dehydrogenase-like, N-terminal
yggP	511145.b4465	InterPro	IPR036291	NAD(P)-binding domain superfamily
ygiQ	511145.b4469	GO Function	GO:0003824	Catalytic activity
ygiQ	511145.b4469	GO Function	GO:0005488	Binding
ygiQ	511145.b4469	GO Function	GO:0005506	Iron ion binding
ygiQ	511145.b4469	GO Function	GO:0043167	Ion binding
ygiQ	511145.b4469	GO Function	GO:0043169	Cation binding
ygiQ	511145.b4469	GO Function	GO:0046872	Metal ion binding
ygiQ	511145.b4469	GO Function	GO:0046914	Transition metal ion binding
ygiQ	511145.b4469	GO Function	GO:0051536	Iron-sulfur cluster binding
ygiQ	511145.b4469	GO Function	GO:0051539	4 iron, 4 sulfur cluster binding
ygiQ	511145.b4469	GO Function	GO:0051540	Metal cluster binding
ygiQ	511145.b4469	STRING clusters	CL:2689	Mixed, incl. Methyltransferase activity, and Condensin complex
ygiQ	511145.b4469	STRING clusters	CL:2690	Mixed, incl. Methyltransferase activity, and Condensin complex
ygiQ	511145.b4469	STRING clusters	CL:2692	Mixed, incl. rRNA (adenine-N6-)-methyltransferase activity, and Methyltransferase type 11
ygiQ	511145.b4469	STRING clusters	CL:2693	Mixed, incl. rRNA (adenine-N6-)-methyltransferase activity, and Methyltransferase type 11
ygiQ	511145.b4469	STRING clusters	CL:2695	Methyltransferase type 11, and SpoU rRNA Methylase family
ygiQ	511145.b4469	UniProt Keywords	KW-0004	4Fe-4S
ygiQ	511145.b4469	UniProt Keywords	KW-0408	Iron
ygiQ	511145.b4469	UniProt Keywords	KW-0411	Iron-sulfur
ygiQ	511145.b4469	UniProt Keywords	KW-0479	Metal-binding
ygiQ	511145.b4469	UniProt Keywords	KW-0949	S-adenosyl-L-methionine
ygiQ	511145.b4469	InterPro	IPR006638	Elp3/MiaA/NifB-like, radical SAM core domain
ygiQ	511145.b4469	InterPro	IPR007197	Radical SAM
ygiQ	511145.b4469	InterPro	IPR013704	UPF0313, N-terminal
ygiQ	511145.b4469	InterPro	IPR020612	Methylthiotransferase, conserved site
ygiQ	511145.b4469	InterPro	IPR022946	Uncharacterised protein family UPF0313
ygiQ	511145.b4469	InterPro	IPR023404	Radical SAM, alpha/beta horseshoe
ygiQ	511145.b4469	InterPro	IPR024560	UPF0313, C-terminal
ygiQ	511145.b4469	SMART	SM00729	Elongator protein 3, MiaB family, Radical SAM
yidC	511145.b3705	GO Process	GO:0006457	Protein folding
yidC	511145.b3705	GO Process	GO:0006810	Transport
yidC	511145.b3705	GO Process	GO:0008104	Protein localization
yidC	511145.b3705	GO Process	GO:0009987	Cellular process
yidC	511145.b3705	GO Process	GO:0015031	Protein transport
yidC	511145.b3705	GO Process	GO:0016043	Cellular component organization
yidC	511145.b3705	GO Process	GO:0022607	Cellular component assembly
yidC	511145.b3705	GO Process	GO:0032978	Protein insertion into membrane from inner side
yidC	511145.b3705	GO Process	GO:0033036	Macromolecule localization
yidC	511145.b3705	GO Process	GO:0043933	Protein-containing complex organization
yidC	511145.b3705	GO Process	GO:0043952	Protein transport by the Sec complex
yidC	511145.b3705	GO Process	GO:0044085	Cellular component biogenesis
yidC	511145.b3705	GO Process	GO:0045184	Establishment of protein localization
yidC	511145.b3705	GO Process	GO:0051179	Localization
yidC	511145.b3705	GO Process	GO:0051205	Protein insertion into membrane
yidC	511145.b3705	GO Process	GO:0051234	Establishment of localization
yidC	511145.b3705	GO Process	GO:0051641	Cellular localization
yidC	511145.b3705	GO Process	GO:0051668	Localization within membrane
yidC	511145.b3705	GO Process	GO:0055085	Transmembrane transport
yidC	511145.b3705	GO Process	GO:0061024	Membrane organization
yidC	511145.b3705	GO Process	GO:0065003	Protein-containing complex assembly
yidC	511145.b3705	GO Process	GO:0070727	Cellular macromolecule localization
yidC	511145.b3705	GO Process	GO:0071702	Organic substance transport
yidC	511145.b3705	GO Process	GO:0071705	Nitrogen compound transport
yidC	511145.b3705	GO Process	GO:0071806	Protein transmembrane transport
yidC	511145.b3705	GO Process	GO:0071840	Cellular component organization or biogenesis
yidC	511145.b3705	GO Process	GO:0072657	Protein localization to membrane
yidC	511145.b3705	GO Process	GO:0090150	Establishment of protein localization to membrane
yidC	511145.b3705	GO Function	GO:0032977	Membrane insertase activity
yidC	511145.b3705	GO Function	GO:0140104	Molecular carrier activity
yidC	511145.b3705	GO Function	GO:0140597	Protein carrier chaperone
yidC	511145.b3705	GO Component	GO:0005886	Plasma membrane
yidC	511145.b3705	GO Component	GO:0005887	Integral component of plasma membrane
yidC	511145.b3705	GO Component	GO:0016020	Membrane
yidC	511145.b3705	GO Component	GO:0016021	Integral component of membrane
yidC	511145.b3705	GO Component	GO:0031224	Intrinsic component of membrane
yidC	511145.b3705	GO Component	GO:0031226	Intrinsic component of plasma membrane
yidC	511145.b3705	GO Component	GO:0031522	Cell envelope Sec protein transport complex
yidC	511145.b3705	GO Component	GO:0032991	Protein-containing complex
yidC	511145.b3705	GO Component	GO:0071944	Cell periphery
yidC	511145.b3705	GO Component	GO:0110165	Cellular anatomical entity
yidC	511145.b3705	STRING clusters	CL:221	Protein transport by the Sec complex, and SRP54-type protein, GTPase domain
yidC	511145.b3705	STRING clusters	CL:223	Protein transport by the Sec complex
yidC	511145.b3705	STRING clusters	CL:225	Cell envelope Sec protein transport complex
yidC	511145.b3705	STRING clusters	CL:35	Translation, and Protein export
yidC	511145.b3705	STRING clusters	CL:37	Translation, and Protein export
yidC	511145.b3705	STRING clusters	CL:39	Mixed, incl. Ribonucleoprotein, and Translation regulator activity
yidC	511145.b3705	STRING clusters	CL:40	Ribonucleoprotein, and Translation factor activity, RNA binding
yidC	511145.b3705	STRING clusters	CL:41	Ribonucleoprotein, and Translation factor activity, RNA binding
yidC	511145.b3705	KEGG	eco02024	Quorum sensing
yidC	511145.b3705	KEGG	eco03060	Protein export
yidC	511145.b3705	KEGG	eco03070	Bacterial secretion system
yidC	511145.b3705	COMPARTMENTS	GOCC:0005886	Plasma membrane
yidC	511145.b3705	COMPARTMENTS	GOCC:0005887	Integral component of plasma membrane
yidC	511145.b3705	COMPARTMENTS	GOCC:0016020	Membrane
yidC	511145.b3705	COMPARTMENTS	GOCC:0016021	Integral component of membrane
yidC	511145.b3705	COMPARTMENTS	GOCC:0031224	Intrinsic component of membrane
yidC	511145.b3705	COMPARTMENTS	GOCC:0031226	Intrinsic component of plasma membrane
yidC	511145.b3705	COMPARTMENTS	GOCC:0031522	Cell envelope Sec protein transport complex
yidC	511145.b3705	COMPARTMENTS	GOCC:0032991	Protein-containing complex
yidC	511145.b3705	COMPARTMENTS	GOCC:0071944	Cell periphery
yidC	511145.b3705	COMPARTMENTS	GOCC:0110165	Cellular anatomical entity
yidC	511145.b3705	UniProt Keywords	KW-0143	Chaperone
yidC	511145.b3705	UniProt Keywords	KW-0472	Membrane
yidC	511145.b3705	UniProt Keywords	KW-0653	Protein transport
yidC	511145.b3705	UniProt Keywords	KW-0811	Translocation
yidC	511145.b3705	UniProt Keywords	KW-0812	Transmembrane
yidC	511145.b3705	UniProt Keywords	KW-0813	Transport
yidC	511145.b3705	UniProt Keywords	KW-0997	Cell inner membrane
yidC	511145.b3705	UniProt Keywords	KW-1003	Cell membrane
yidC	511145.b3705	UniProt Keywords	KW-1133	Transmembrane helix
yidC	511145.b3705	Pfam	PF02096	60Kd inner membrane protein
yidC	511145.b3705	Pfam	PF14849	YidC periplasmic domain
yidC	511145.b3705	InterPro	IPR001708	Membrane insertase YidC/ALB3/OXA1/COX18
yidC	511145.b3705	InterPro	IPR019998	Membrane insertase YidC
yidC	511145.b3705	InterPro	IPR028053	Membrane insertase YidC, N-terminal
yidC	511145.b3705	InterPro	IPR028055	Membrane insertase YidC/Oxa/ALB, C-terminal
yidC	511145.b3705	InterPro	IPR038221	YidC, periplasmic domain superfamily
yidC	511145.b3705	InterPro	IPR047196	Membrane insertase YidC/ALB, C-terminal
yihA	511145.b3865	GO Process	GO:0000910	Cytokinesis
yihA	511145.b3865	GO Process	GO:0000917	Division septum assembly
yihA	511145.b3865	GO Process	GO:0007049	Cell cycle
yihA	511145.b3865	GO Process	GO:0009987	Cellular process
yihA	511145.b3865	GO Process	GO:0016043	Cellular component organization
yihA	511145.b3865	GO Process	GO:0022402	Cell cycle process
yihA	511145.b3865	GO Process	GO:0022607	Cellular component assembly
yihA	511145.b3865	GO Process	GO:0032506	Cytokinetic process
yihA	511145.b3865	GO Process	GO:0044085	Cellular component biogenesis
yihA	511145.b3865	GO Process	GO:0051301	Cell division
yihA	511145.b3865	GO Process	GO:0071840	Cellular component organization or biogenesis
yihA	511145.b3865	GO Process	GO:0090529	Cell septum assembly
yihA	511145.b3865	GO Function	GO:0000166	Nucleotide binding
yihA	511145.b3865	GO Function	GO:0005488	Binding
yihA	511145.b3865	GO Function	GO:0005525	GTP binding
yihA	511145.b3865	GO Function	GO:0017076	Purine nucleotide binding
yihA	511145.b3865	GO Function	GO:0019001	Guanyl nucleotide binding
yihA	511145.b3865	GO Function	GO:0032553	Ribonucleotide binding
yihA	511145.b3865	GO Function	GO:0032555	Purine ribonucleotide binding
yihA	511145.b3865	GO Function	GO:0032561	Guanyl ribonucleotide binding
yihA	511145.b3865	GO Function	GO:0035639	Purine ribonucleoside triphosphate binding
yihA	511145.b3865	GO Function	GO:0036094	Small molecule binding
yihA	511145.b3865	GO Function	GO:0043167	Ion binding
yihA	511145.b3865	GO Function	GO:0043168	Anion binding
yihA	511145.b3865	GO Function	GO:0043169	Cation binding
yihA	511145.b3865	GO Function	GO:0046872	Metal ion binding
yihA	511145.b3865	GO Function	GO:0097159	Organic cyclic compound binding
yihA	511145.b3865	GO Function	GO:0097367	Carbohydrate derivative binding
yihA	511145.b3865	GO Function	GO:1901265	Nucleoside phosphate binding
yihA	511145.b3865	GO Function	GO:1901363	Heterocyclic compound binding
yihA	511145.b3865	GO Component	GO:0005622	Intracellular anatomical structure
yihA	511145.b3865	GO Component	GO:0005737	Cytoplasm
yihA	511145.b3865	GO Component	GO:0005829	Cytosol
yihA	511145.b3865	GO Component	GO:0110165	Cellular anatomical entity
yihA	511145.b3865	STRING clusters	CL:378	Mixed, incl. rRNA modification, and tRNA methylation
yihA	511145.b3865	STRING clusters	CL:379	rRNA modification, and tRNA methylation
yihA	511145.b3865	STRING clusters	CL:380	RNA methylation, and GTP binding domain
yihA	511145.b3865	STRING clusters	CL:416	Mixed, incl. GTP binding domain, and S4 RNA-binding domain
yihA	511145.b3865	COMPARTMENTS	GOCC:0005622	Intracellular
yihA	511145.b3865	COMPARTMENTS	GOCC:0005737	Cytoplasm
yihA	511145.b3865	COMPARTMENTS	GOCC:0005829	Cytosol
yihA	511145.b3865	COMPARTMENTS	GOCC:0110165	Cellular anatomical entity
yihA	511145.b3865	UniProt Keywords	KW-0131	Cell cycle
yihA	511145.b3865	UniProt Keywords	KW-0132	Cell division
yihA	511145.b3865	UniProt Keywords	KW-0342	GTP-binding
yihA	511145.b3865	UniProt Keywords	KW-0460	Magnesium
yihA	511145.b3865	UniProt Keywords	KW-0479	Metal-binding
yihA	511145.b3865	UniProt Keywords	KW-0547	Nucleotide-binding
yihA	511145.b3865	UniProt Keywords	KW-0717	Septation
yihA	511145.b3865	Pfam	PF01926	50S ribosome-binding GTPase
yihA	511145.b3865	InterPro	IPR006073	GTP binding domain
yihA	511145.b3865	InterPro	IPR019987	GTP-binding protein, ribosome biogenesis, YsxC
yihA	511145.b3865	InterPro	IPR027417	P-loop containing nucleoside triphosphate hydrolase
yihA	511145.b3865	InterPro	IPR030393	EngB-type guanine nucleotide-binding (G) domain
ykgF	511145.b0307	GO Process	GO:0006082	Organic acid metabolic process
ykgF	511145.b0307	GO Process	GO:0006089	Lactate metabolic process
ykgF	511145.b0307	GO Process	GO:0008152	Metabolic process
ykgF	511145.b0307	GO Process	GO:0009987	Cellular process
ykgF	511145.b0307	GO Process	GO:0019516	Lactate oxidation
ykgF	511145.b0307	GO Process	GO:0019752	Carboxylic acid metabolic process
ykgF	511145.b0307	GO Process	GO:0032787	Monocarboxylic acid metabolic process
ykgF	511145.b0307	GO Process	GO:0043436	Oxoacid metabolic process
ykgF	511145.b0307	GO Process	GO:0044237	Cellular metabolic process
ykgF	511145.b0307	GO Process	GO:0044281	Small molecule metabolic process
ykgF	511145.b0307	GO Process	GO:0071704	Organic substance metabolic process
ykgF	511145.b0307	GO Process	GO:1901615	Organic hydroxy compound metabolic process
ykgF	511145.b0307	GO Function	GO:0005488	Binding
ykgF	511145.b0307	GO Function	GO:0043167	Ion binding
ykgF	511145.b0307	GO Function	GO:0043169	Cation binding
ykgF	511145.b0307	GO Function	GO:0046872	Metal ion binding
ykgF	511145.b0307	GO Function	GO:0051536	Iron-sulfur cluster binding
ykgF	511145.b0307	GO Function	GO:0051539	4 iron, 4 sulfur cluster binding
ykgF	511145.b0307	GO Function	GO:0051540	Metal cluster binding
ykgF	511145.b0307	STRING clusters	CL:2403	Mixed, incl. Glycolate dehydrogenase activity, and LUD domain
ykgF	511145.b0307	STRING clusters	CL:2421	LUD domain, and L-lactate dehydrogenase activity
ykgF	511145.b0307	UniProt Keywords	KW-0004	4Fe-4S
ykgF	511145.b0307	UniProt Keywords	KW-0249	Electron transport
ykgF	511145.b0307	UniProt Keywords	KW-0408	Iron
ykgF	511145.b0307	UniProt Keywords	KW-0411	Iron-sulfur
ykgF	511145.b0307	UniProt Keywords	KW-0479	Metal-binding
ykgF	511145.b0307	UniProt Keywords	KW-0677	Repeat
ykgF	511145.b0307	UniProt Keywords	KW-0813	Transport
ykgF	511145.b0307	Pfam	PF02589	LUD domain
ykgF	511145.b0307	Pfam	PF13183	4Fe-4S dicluster domain
ykgF	511145.b0307	InterPro	IPR003741	LUD domain
ykgF	511145.b0307	InterPro	IPR004452	4Fe-4S binding protein
ykgF	511145.b0307	InterPro	IPR009051	Alpha-helical ferredoxin
ykgF	511145.b0307	InterPro	IPR017896	4Fe-4S ferredoxin-type, iron-sulphur binding domain
ykgF	511145.b0307	InterPro	IPR017900	4Fe-4S ferredoxin, iron-sulphur binding, conserved site
ykgF	511145.b0307	InterPro	IPR024185	5-formyltetrahydrofolate cyclo-ligase-like domain superfamily
ykgF	511145.b0307	InterPro	IPR024569	Lactate utilization protein B, C-terminal
ykgF	511145.b0307	InterPro	IPR037171	NagB/RpiA transferase-like
yraN	511145.b3148	GO Function	GO:0003676	Nucleic acid binding
yraN	511145.b3148	GO Function	GO:0005488	Binding
yraN	511145.b3148	GO Function	GO:0097159	Organic cyclic compound binding
yraN	511145.b3148	GO Function	GO:1901363	Heterocyclic compound binding
yraN	511145.b3148	STRING clusters	CL:897	Mixed, incl. Peptidoglycan metabolic process, and Cell division site
yraN	511145.b3148	STRING clusters	CL:898	Mixed, incl. Peptidoglycan metabolic process, and Cell division site
yraN	511145.b3148	STRING clusters	CL:899	Peptidoglycan metabolic process, and Cell septum
yraN	511145.b3148	Pfam	PF02021	Uncharacterised protein family UPF0102
yraN	511145.b3148	InterPro	IPR003509	Uncharacterised protein family UPF0102
yraN	511145.b3148	InterPro	IPR011335	Restriction endonuclease type II-like
yraN	511145.b3148	InterPro	IPR011856	tRNA endonuclease-like domain superfamily
ytfH	511145.b4212	GO Function	GO:0003676	Nucleic acid binding
ytfH	511145.b4212	GO Function	GO:0003677	DNA binding
ytfH	511145.b4212	GO Function	GO:0005488	Binding
ytfH	511145.b4212	GO Function	GO:0097159	Organic cyclic compound binding
ytfH	511145.b4212	GO Function	GO:1901363	Heterocyclic compound binding
ytfH	511145.b4212	STRING clusters	CL:6207	Mostly uncharacterized, incl. Degradation of aromatic compounds, and Uracil catabolic process
ytfH	511145.b4212	STRING clusters	CL:6208	Mostly uncharacterized, incl. 3-phenylpropionate catabolic process, and Uracil catabolic process
ytfH	511145.b4212	STRING clusters	CL:6318	Mixed, incl. LysE type translocator, and Major facilitator superfamily
ytfH	511145.b4212	STRING clusters	CL:6319	Mixed, incl. LysE type translocator, and EamA-like transporter family
ytfH	511145.b4212	STRING clusters	CL:6320	Mixed, incl. LysE type translocator, and EamA-like transporter family
ytfH	511145.b4212	STRING clusters	CL:6321	Mixed, incl. LysE type translocator, and EamA-like transporter family
ytfH	511145.b4212	STRING clusters	CL:6345	Mixed, incl. Putative zinc- or iron-chelating domain, and Cellular response to amino acid stimulus
ytfH	511145.b4212	UniProt Keywords	KW-0238	DNA-binding
ytfH	511145.b4212	UniProt Keywords	KW-0804	Transcription
ytfH	511145.b4212	UniProt Keywords	KW-0805	Transcription regulation
ytfH	511145.b4212	Pfam	PF01638	HxlR-like helix-turn-helix
ytfH	511145.b4212	InterPro	IPR002577	Helix-turn-helix, HxlR type
ytfH	511145.b4212	InterPro	IPR036388	Winged helix-like DNA-binding domain superfamily
ytfH	511145.b4212	InterPro	IPR036390	Winged helix DNA-binding domain superfamily
```
